# Efficient multi-station air quality prediction in Delhi with wavelet and optimization-based models

**DOI:** 10.1371/journal.pone.0330465

**Published:** 2025-08-19

**Authors:** Lakshmi Sankar, Krishnamoorthy Arasu

**Affiliations:** School of Computer Science Engineering, Vellore Institute of Technology, Vellore, India; Duy Tan University: Dai Hoc Duy Tan, VIET NAM

## Abstract

The swift decline in the air quality in South Asian mega cities, especially Delhi, presents significant threats to human health owing to elevated concentrations of particulate matter (PM2.5) resulting from dense populations, heavy traffic, and industrial emissions. Precise and efficient prediction of air quality is essential for successful mitigation and policy formulation. This research introduces an innovative predictive framework, AquaWave-BiLSTM, that combines sophisticated feature extraction and optimization methods to enhance multi-station air quality forecasting in Delhi. Hourly air quality and meteorological data were gathered from six monitoring sites from June 2018 to October 2019. The proposed model integrates Wavelet Transform for frequency pattern extraction, Principal Component Analysis (PCA) for dimensionality reduction, and A hybrid Aquila Optimizer and Arithmetic Optimization (AOAOA) for the selection of pertinent features. A Bidirectional Long Short-Term Memory (Bi-LSTM) network is utilized to simulate temporal interdependence. The AquaWave-BiLSTM framework demonstrated exceptional predictive accuracy, with a Mean Squared Error (MSE) of 0.00065, a Mean Absolute Error (MAE) of 0.04566, a Root Mean Square Error (RMSE) of 0.02523, and an R² value of 0.9494, surpassing conventional methodologies. Furthermore, the model exhibited computational efficiency with an average execution time of 20.57 seconds. The Wilcoxon Signed-Rank Test statistically validated the relevance of the suggested feature extraction and selection method for all monitoring stations. The AquaWave-BiLSTM framework enables efficient, interpretable air quality forecasting, with SHAP clarifying feature contributions.

## 1. Introduction

Environmental deterioration, including soil, water, and air, has been linked to economic development. Among these factors, air pollution has a negative influence on people’s health. Outdoor pollution and particles have a detrimental impact on both the mental and physical wellness of individuals [1]. The ambient air quality has seen a substantial decline due to a rapid rise in vehicle usage, rubbish burning, construction activities, industrial waste, fossil fuel emissions, and manufacturing processes. The WHO reports that around 4.2 million individuals succumb prematurely annually because they reveal ambient air pollution. The airborne pollutants, often referred to as air pollutants, contain Nitrogen dioxide (NO2), Carbon monoxide (CO), sulfur dioxide (SO2), Ozone (O3), and Particulate Matter (PM2.5 and PM10) [2]. Among them, PM2.5 is a term used to describe particles that have a diameter below 2.5 micrometers. It is the most significant contributor to health risks among all pollutants. These particles possess a micro size and low volume, enabling them to stay in the environment for extended durations. Research suggests that elevated levels of air pollution and dangerous particles might harm public health, negatively impact respiratory diseases, heart disease, and cancer in the lungs. In addition, excessive air pollution will cause haze, leading to reduced visibility in the sky, traffic accidents, aviation delays, and other related issues. Besides matter, many pollutants such as SOx, NOx, CO, organic volatile compounds, and aromatic polycyclic hydrocarbons, which are often known as the global burden of illness, have negative impacts [3]. Hence, the investigation of air pollution is crucial for the preservation of the ecosystem [4]. Recently, there has been a notable focus on air pollution in New Delhi, the national capital of India, and its impact on the health of the public. Delhi has emerged as one of the most heavily polluted urban areas globally. Acute air pollution occurs in New Delhi throughout the winter, with the Air Quality Index (AQI) declining to severe stages and sometimes reaching an emergency level [5] PM 2.5 is the primary pollutant of utmost importance in the Delhi area. These pollutants have been utilized in the computation of the air quality prediction. Meteorological features, including relative humidity, temperature, air pressure, wind direction, rainfall, sun radiation, and speed of the wind, significantly influence the concentration of pollutants in the atmosphere [6,7]. The air pollution in Delhi is influenced by a complex interplay of local and regional causes. Significant sources are traffic emissions from more than 11 million registered cars, industrial operations, construction dust, and the open incineration of biomass and garbage. Seasonal stubble burning in the adjacent states of Punjab and Haryana markedly exacerbates pollution levels, especially in October and November. Moreover, climatic factors like low wind velocities, elevated humidity, and recurrent temperature inversions in winter impede pollutant dispersion and intensify concentration levels. This work intends to develop customized forecasting models that can precisely capture the temporal and geographical dynamics of pollution due to specific area factors.

## 2. Literature survey

There are two primary groups of forecasting models for air pollution concentration: simulation-based and data-driven techniques, including statistics or machine learning techniques. The simulation-based technique integrates physician and chemical models to provide metrological and atmospheric factors, enabling the simulation of air pollution emission, transport, and chemical change. Nevertheless, these models are plagued by inaccuracies in their numerical representation, and the limited availability of data hinders the accurate parameterization of aerosol emissions. Data-driven methods use machine learning and statistical methods to identify correlations across predictors and dependent attributes sequentially. Hence, it is important to use various air quality observing indicators, climatic parameters, and sequential variables to anticipate PM2.5 levels accurately. This condition arose during the training and analysis of substantial volumes of input data in combination with machine learning techniques, hence triggering the contests associated with their use [8]. Notable research conducted by Ameer et al examined the effectiveness of four regression techniques: Decision tree, Gradient Boosting, Multilayer Perceptron, and Artificial Neural Network, which is used to forecast air quality levels [9,10]. The evaluation of these approaches was conducted by monitoring the concentration of PM2.5 particles in the air and determining the AQI In the same manner, deep learning methods such as recurrent neural networks [11] Long Short-Term Memory networks (LSTMs) were formed to overwhelm the constraints of conventional RNNs in capturing long-standing dependencies in sequences [12,13]. Implementing Gated Recurrent Unit (GRU) networks is comparatively easier, but they give equivalent efficiency to the LSTM networks [14]. In bi-directional RNNs, two hidden layers are connected in opposing directions to a common output layer, which can concurrently accept input from both past and future situations. Convolutional Neural Networks (CNNs) can extract spatiotemporal properties from input data, including the pertinent aspects of PM2.5 concentration across various monitoring stations. CNN and LSTM networks have shown considerable promise in predicting PM2.5 levels [15,16]. Zhang et al. [17] employed empirical mode decomposition with a Bi-LSTM network to enhance the accuracy of AQI prediction [17]. The Temporal Convolution Neural Network (TCN) is a overall technique used for the forecasting of convolutional sequences. It is capable of effectively capturing historical observations and related exogenous variables. This is achieved by considering the causal relationship between the convolutions in the architecture [18]. Nevertheless, the analytical capabilities of the deep learning models have seen a certain degree of improvement. However, when the topic develops intricately, the precision of predictions may be constrained by the configuration of the Unified network model. The hybrid deep learning model incorporates many system topologies to effectively compute intricate data and enhance its ability to adapt to variations in PM2.5 concentration [19,20].In 2023, Yonar and Yonar et al [21] used a combination of models, ANFIS, which included artificial neural networks and fuzzy inference systems [22], to predict several air pollution metrics in Istanbul. Conventional models fail to adequately represent both frequency and temporal relationships in air pollution data. They struggle to choose ideal characteristics, balance precision and processing expense, and manage long-term interdependence. Feature extraction methods, like Wavelet Transform (WT), facilitate the identification of temporal and frequency-based patterns in air quality data. Principal Component Analysis (PCA) is often utilized to decrease dimensionality while preserving essential variance. Research conducted by Mallat (1999) on wavelet transformations and S. A. Alsenan et al [23] on PCA validates their efficacy in extracting essential information and enhancing model efficiency. Liu et al. (2020) [24] further showed that integrating WT with PCA improves prediction efficacy in air pollution modeling. Natarajan S et al [25] designed an improved regression model specifically predicting the AQI. The Grey Wolf Optimization approach integrates with a Decision Tree Regression framework to enhance the accuracy of predictions Aarthi et al [26] proposed the BSMO method, which draws inspiration from the foraging activities of spider monkeys and employs a factor to determine important features from data. Gurumoorthy et al [27] implement Reinforcement Swarm Optimizer and Bi-GRU; the convergence rate of RSO may diminish as it nears the global optimum, especially in search regions with high dimensionality. This may result in overfitting, which can affect the performance of new, unfamiliar data. Panneerselvam et al [28] employed the CDAO model using the Arithmetic Optimization Algorithm (AOA) has a slower convergence rate, mostly because it employs division and multiplication extensively in the first search phase. Additionally, it has difficulties in maintaining a wide range of solutions and assuring the ongoing adaptability of the search agents, both of which are crucial for achieving a successful search. The Arithmetic Optimization Algorithm (AOA), although proficient in global search, encounters difficulties in smoothly transitioning between exploration and exploitation stages. Likewise, the Aquila Optimizer (AO), utilized by Al-Ganesh et al. in predictive models, has robust search capabilities but may become ensnared in inferior solutions in the absence of an efficient escape mechanism. To tackle these issues, a unique hybrid methodology, AOAOA [29] (Aquila Optimizer and Arithmetic Optimization Algorithm), was developed by integrating the advantages of AO and AOA. In contrast to traditional optimization methods like PSO, GA, and WOA, AOAOA adeptly balances exploration and exploitation, guaranteeing optimum feature selection while reducing the likelihood of entrapment in local optima. Although hybrid optimization approaches have been extensively investigated in fields including engineering design, path planning, and biomedical applications, their applicability in air quality prediction and feature selection optimization is still constrained. The suggested AOAOA technique increases computational efficiency and promotes feature selection accuracy, as evidenced by comparative performance assessments across various air quality monitoring stations. Bidirectional Long Short-Term Memory (Bi-LSTM), a deep learning model, has become well-known for its capacity to extract intricate temporal correlations from sequential data. In contrast to traditional LSTM, Bi-LSTM analyses input sequences in both forward and backward directions, improving pattern identification. Nonetheless, the elevated classical complexity of Bi-LSTM heightens the susceptibility to overfitting, especially when trained on datasets characterized by high feature dimensionality or constrained training samples. Overfitting transpires when the model retains specific patterns instead of generalizing, resulting in worse performance on novel data. To address this, the proposed AquaWave-BiLSTM model incorporates Wavelet Transform (WT), Principal Component Analysis PCA, and Aquila Optimizer and Arithmetic Optimization Algorithm AOAOA, which diminishes feature dimensionality and improves relevant feature selection. Furthermore, early stopping, dropout regularization, and Min-Max normalization are implemented to enhance training stability and generalization. The Wilcoxon Signed-Rank Test further substantiates the efficacy of feature selection across many monitoring stations, guaranteeing that the model identifies significant relationships without unnecessary complexity. The AquaWave-BiLSTM model guarantees accurate, efficient, and dependable air quality forecasts by tackling frequency-temporal relationships, feature selection, and computational efficiency while reducing the probability of overfitting.

The primary objective of this article is the following:

1. The initial phases of this research used Min-Max Scaler, a data pretreatment method that rescales data inputs to a standardized range of values from 0 to 1. This ensures that every parameter has an equal influence on predictive models and parameter has an equal influence on forecasting models and prevents any one characteristic from overpowering the model.
2. Subsequently, this study executed a correlation analysis to identify the most suitable meteorological parameters (Wind Direction, Temperature, Wind Speed, Relative Humidity, Solar Radiation, Barometric pressure, and historical PM 2.5) within the available sets of data.
3. Subsequently, Wavelet Transform and PCA were exploited for feature extraction and dimensionality reduction. The Wavelet Transform extracted frequency-based features from air quality data, whereas PCA decreased dimensionality by identifying the most pertinent components. This hybrid methodology improved the model’s capacity to understand complex patterns in air quality data.
4. Following this, the Aquila Optimizer (AO) and the Arithmetic Optimization Algorithm (AOAOA) were designed and used to determine distinctive features from the specified meteorological factor. This operation significantly decreases the complexity of the mode and the computational time. This approach utilizes a global optimization strategy that enhances efficiency, functions in several dimensions, and achieves effective search results. The hybrid approach combines the exploratory abilities of AO with the exploitative strategies of AOA, effectively addressing optimization challenges such as slow convergence, local optima issues, and optimizing the parameters for this Air quality predictive model.
5. Finally, the Bi-LSTM framework is employed for the prediction method since it successfully captures dependency over time in both directions of time series data.
6. This research assesses the consistency of feature extraction and selection techniques across several monitoring stations utilizing the Wilcoxon Signed-Rank Test. The findings underscore the need to integrate Wavelet+PCA with AOAOA to create an optimum feature subset, proposing an integrated approach to improve prediction accuracy and generality.
7. To progress the interpretability of the proposed predictive system, Shapley Additive explanations (SHAP) were employed to assess the contributions of specific meteorological and pollutant variables to PM2.5 forecasts. This study identified main predictors, uncovered station-specific variations, and offered practical insights into the determinants affecting PM2.5 dynamics.

The study is arranged systematically: Section 1: Introduction, Section 2: Literature Survey, Section 3: Datasets Description, Section 4: Proposed Methodology, Section 5: Evaluative Metrics and Experimental Setup, Section 6: Results and Discussion, Section 7: Conclusion.

## 3. Datasets description

The present research utilizes the hourly average air quality and atmospheric data obtained from the observing stations operated by the Central Pollution Control Board (CPCB) at specific sites in Delhi, India [8]. The city of Delhi, which serves as the administrative center of India, is recognized as the national capital of the country. The city is experiencing fast growth and is now ranked as the second most populous city in India. Additionally, it is recognized as one of the most polluted cities globally. For this research selected 6 highly polluted locations in Delhi and many adjacent districts referred to as the National Capital Region (NCR) [30]. The dataset was sourced from Sonawani, Shilpa, and Patil, Kailas (2021), entitled Delhi Multi-Site Air-Quality Data Set (Mendeley Data, V1, https://doi.org/10.17632/bzhzr9b64v.1). This publicly accessible dataset is a refined and augmented iteration of the original CPCB data, as curated and sustained by the first contributors. Consequently, extra imputation or outlier management was unnecessary in our investigation. The dataset encompasses the timeframe from June 1, 2018, to October 1, 2019, with hourly measurements. In this research, 20 attributes for our analysis were used data from the Central Pollution Control Board in India [31]. The dataset contains the concentration levels of several air pollution parameters measured by 6 government-installed monitoring stations in the Delhi–NCR area. Table 1 presents comprehensive information about the geography of the 6 data-collecting stations [7,32]. The Air Quality Index (AQI) is an excellent tool for expediting the delivery of air quality information to the public while calculating the AQI, a minimum of three out of the eight pollutants: PM2.5, CO, PM 10, NH3, O3, NO2, SO2 and Pb, must be included in the calculation. At least two of these pollutants must be considered PM10 and PM2.5 [33]. The research utilizes six climatic parameters: Temperature (Temp,o c), relative humidity (RH,%), wind direction (WD,o), wind speed (WS, m/s), solar radiation (SR, W/m 2), and atmospheric pressure (AP,mmHg). The climate parameters values may be obtained at the CPCB site of [31].

**Table 1:** Shows the location details regarding the data gathering in Delhi.

Multi-Station Name	District	Coordinates (Latitude, Longitude)
Ashok Vihar, New Delhi	Northwest	[28.6736,77.1811]
Dwarka, Delhi, India	Southwest	[28.6100,77.0500]
DC Stadium (Jawaharlal Nehru Stadium)	Central Delhi	[28.6200,77.0333]
Najafgarh, Delhi, India	Southwest	[28.5833,77.0333]
Nehru Nagar, Lajpat Nagar, New Delhi	South Delhi	[25.5539,77.2408]
Okhla, New Delhi	South Delhi	[28.5356,77.2778]

## 4. Proposed methodology

The framework for air quality prediction in this sequential data study consists of 5 stages are:

- Data normalization – Min- Max standardization.
- Correlation evaluation – Spearman correlation approach.
- Feature Extraction and Dimensionality Reduction – Wavelet Transform and PCA.
- Feature selection – AOAOA algorithm.
- Prediction – Bi-LSTM.

**Fig 1:**
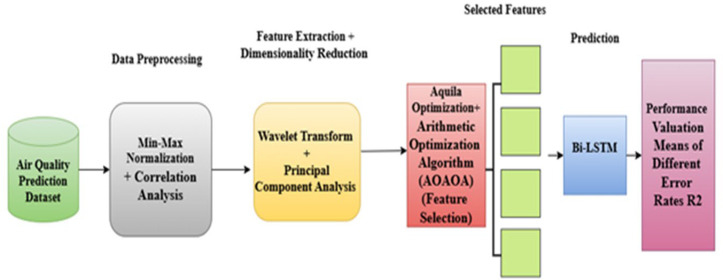
The general architecture of the proposed model of regression.

The graphic displaying the proposed regression model is demonstrated in Fig 1.

### 4.1 Data preprocessing

**Fig 2:**
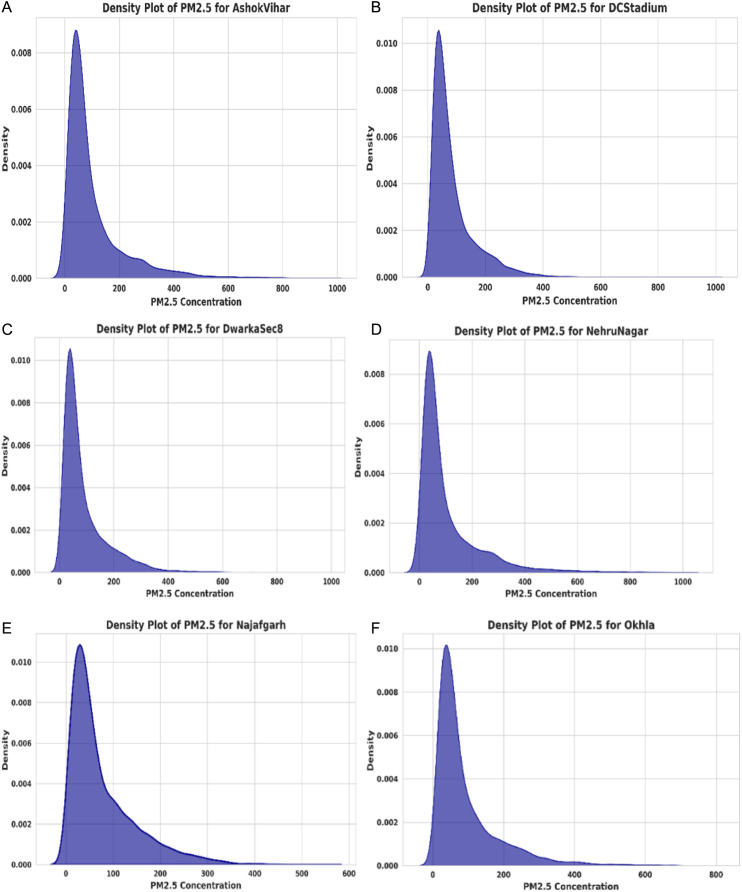
Density plot of PM2.5 for multi station.

**Fig 3:**
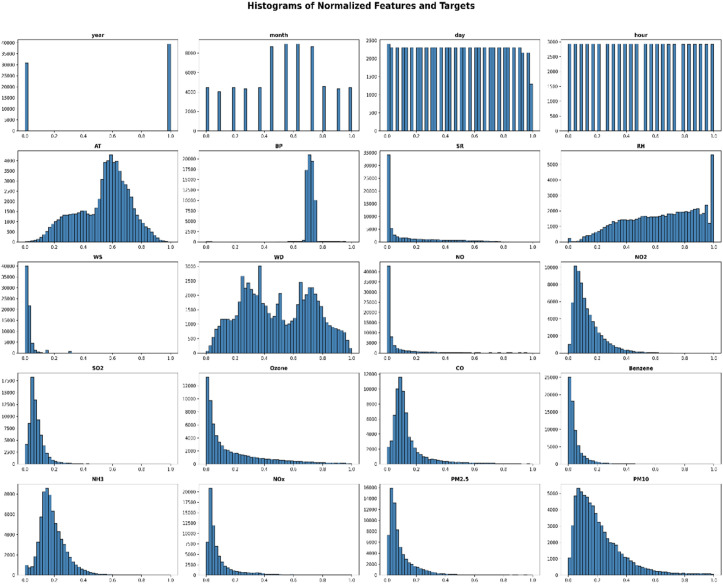
The mean normalized air quality data across multiple stations.


The study uses min-max normalization. It is commonly referred to as feature scaling and is a widely used strategy in deep learning and machine learning models. Min-max normalization preserved the original form of the datasets by establishing an association between the original data values. This limited range was achieved by reducing the standard deviations, which helped to minimize the influence of anomalies. The Min-max Normalization approach was used to minimize the variation among the corresponding values in the dataset [26]. Min-max normalization guarantees that characteristics with extensive numerical ranges (e.g., PM10) do not overshadow features with smaller scales (e.g., WS, NH₃) by scaling all values within a predetermined range, hence maintaining their relative significance. This study examines PM2.5 concentrations at six monitoring sites using density plots. Fig 2 illustrates the Density plot of PM2.5 for Multi Station. These graphs illustrate pollution intensity, distribution patterns, and variability, facilitating the identification of severe pollution occurrences and aiding air quality management choices. The dataset preprocessing steps, including loading, normalization, and station-wise density analysis, are detailed in S1 File in S1 Data. Fig 3 presents the Average Normalized Air Quality Data Across Multiple Stations. The equation for Min-Max Normalization is provided below,

$${\text{X}}_{\text{std}}=\frac{\text{X }-\;{\text{X}}_{\min}}{{\text{X}}_{\text{max }}-\;{\text{X}}_{\min}}$$ (1)

$$X_{scaled\;}=X_{std}\times (max-min)\;+\;min$$ (2)

### 4.2 Correlation between the concentration of PM2.5 and meteorological factors

**Fig 4:**
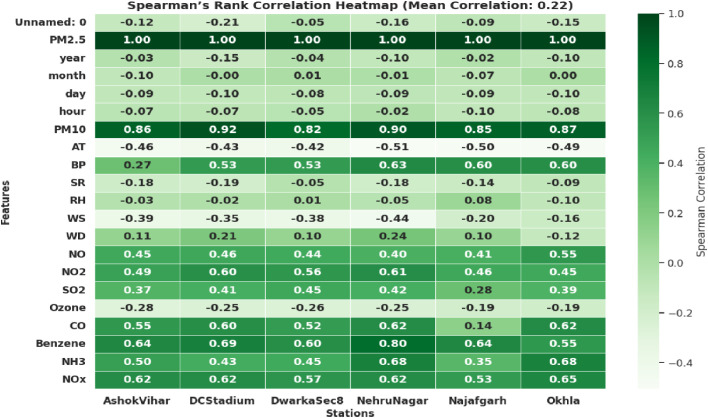
A Spearman’s ranking correlation coefficient represents the Mean correlation between PM2.5 and atmospheric components, for multi-stations.


Correlation evaluation is crucial for improving the precision of air quality forecasting models since meteorological conditions substantially influence the focus of PM2.5. Wind speed, humidity, and atmospheric pressure influence the reduction of pollutant concentration [7]; however, excessive humidity may exacerbate air quality [34]. The Spearman’s rank Coefficient (𝜌) is utilized to assess the association between PM2.5 and atmospheric parameters in numerous research regions. PM2.5 is significantly impacted by meteorological factors such as relative humidity, air pressure, temperature, wind direction, rainfall, sun radiation, and wind speed. The construction of photochemical smog in the atmosphere is affected by temperature, sunshine, and composition, while photochemical activity and thermal reactions in the surrounding air are determined by relative humidity and atmospheric pressure. Fig 4 represents Spearman’s rank coefficient employed to analyze the impact of atmospheric variables and PM2.5 [35].

$$\rho =\;\frac{6\;\sum_{k}\delta_{k}^{2}}{k(k^{2\;}-1)}$$ (3)

$$\delta_{K\;}=\text{ R }(\;{AQI}_{K\;})\;-\text{ R }(m_{k}\;)\;$$ (4)

**Fig 5:**
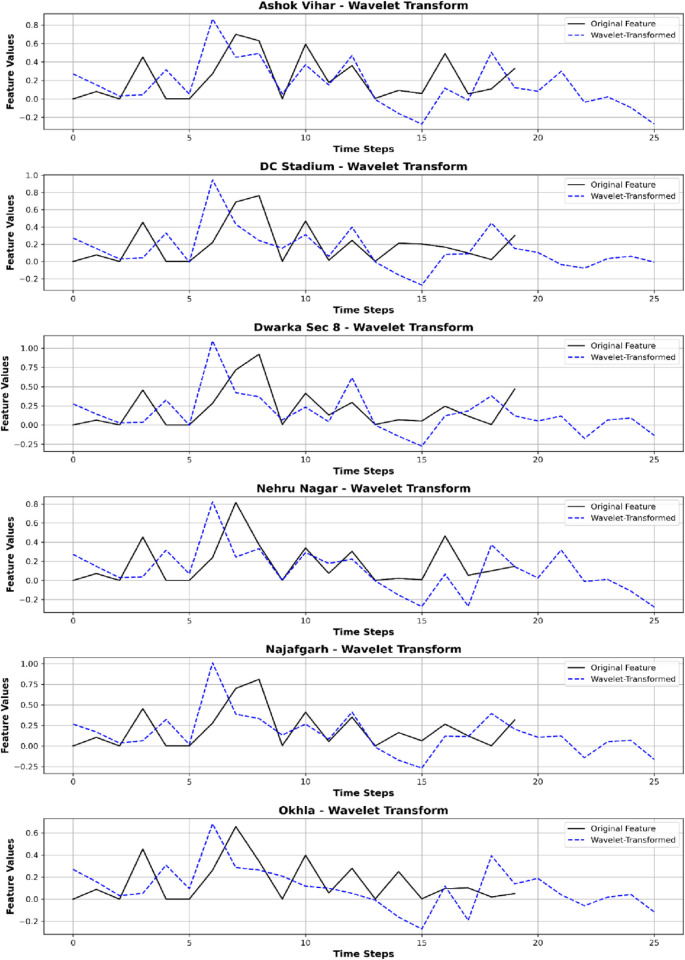
Comparison of original and wavelet-transformed features across six monitoring stations.

The symbol $\delta_{k}$ represents the difference R (${AQI}_{K}$) identifies the rank of the AQI, and R ($m_{k}$) represents the position of that special meteorological parameter among the other observations. When the ρ value is between 0 and 1, we see a positive association between x and y. A negative correlation arises when the result falls between −1 and 0. Table 2 provides Spearman’s rank coefficients between the PM2.5 and meteorological factors in Delhi. The meteorological elements that are taken into consideration include temperature AT, RH, WD, AP, SR, and WS [8]. PM2.5 Particles have a negative correlation with temperature and wind speed. Therefore, under colder circumstances, these particles tend to accumulate in the air, leading to a deterioration in the accuracy of air quality forecasts [36]. In this research, consider the influence of wind speed; greater wind speeds decrease the pollution levels of particles, resulting in improved air quality. Higher Barometric pressure levels contribute to increased concentration of pollutant particles, leading to poor air quality. Fig 5 illustrates a robust mean correlation between PM2.5 readings and metrological factors, including temperature, wind speed, humidity, and air pressure. This correlation will be used to create advanced deep-learning models capable of properly predicting AQI levels.

**Table 2:** Spearman’s rank means correlation coefficient between PM2.5 and Climatic Parameter across multiple stations.

AT	RH	WD	WS	AP	SR
−0.5	−0.42	0.098	−0.2	0.6	−0.14

### 4.3 Feature extraction and dimensionality reduction using wavelet transform and PCA for air quality prediction

Feature extraction and dimensionality reduction are essential operations that improve the efficiency and precision of predictive models employed in air-quality forecasting. This study employs Wavelet Transform (WT) on air quality datasets obtained from six separate monitoring stations—Ashok Vihar, DC Stadium, Dwarka Sec 8, Nehru Nagar, Najafgarh, and Okhla—to extract significant features before the implementation of PCA and BiLSTM networks. WT effectively disaggregates time-series air quality data into distinct frequency components, preserving both temporal and spectral attributes. The Discrete Wavelet Transform (DWT), employing the Daubechies (db4) wavelet, was utilized to derive approximation and detail coefficients, facilitating the differentiation between long-term trends and transient fluctuations in pollutant levels. The ‘db4’ wavelet was chosen after assessing various wavelet families (e.g., db2, sym4, coif1), as it exhibited an optimal balance between temporal and spectral localization, making it particularly adept at detecting unexpected shifts and fluctuating peaks frequently found in air quality time series. Its small framework and orthogonal characteristics render it ideal for multi-resolution studies. The features derived from wavelet analysis were further analyzed using PCA to reduce dimensionality while preserving critical information. PCA converts correlated data into orthogonal Principal Components (PCs) by the computation of the covariance matrix and subsequent eigenvalue decomposition. The variance ratio associated with the major components was examined utilizing a cumulative variance threshold approach. The initial 10 main components were chosen since they jointly accounted for more than 95% of the overall variation in the dataset. This guarantees little information loss while substantially decreasing feature dimensionality. The integration of Wavelet Transform with PCA enhances feature extraction by minimizing noise and redundancy, which is especially crucial for time series data characterized by strong correlation among input characteristics. This preprocessing step improves the prediction capacity of the BiLSTM model by mitigating overfitting. The findings indicate that this hybrid method markedly enhances model accuracy by adeptly addressing both temporal dynamics and structural trends across various air quality monitoring sites.

The discrete Wavelet Transform (DWT) and Principal Component Analysis (PCA) mathematical formulae are as follows:

DWT uses wavelet basis functions to break down a signal x(t) into approximation and detail coefficients.


$$C_{j,k}\;=\;\int x(t)\;\varphi_{j,}\text{ k}(\text{t})\text{ dt }$$ (5)

The wavelet function is defined as:


$$\varphi_{j\;,k(t)}=\;2^{\frac{j}{2}}\;\varphi \;(2^{j}\text{t }–\text{ k})$$ (6)

Where:

j = scale index(controls resolution)

k = translation index (controls position)

φ(t) = mother wavelet.

The approximation coefficients $A_{j\;}$(k) and details coefficients Dj (k) are computed as:


$$A_{j\;}(\text{k})\;=\;\sum x(n)\Phi j,k(n)$$ (7)

Dj (k)=∑x(n)φj,k(n) (8)

Where Φj,k is the scaling function.

PCA converts the data into a new coordinate system to optimize variance along the major components.


$$\text{Covariance\textbackslash{} Matrix}:\;C=\frac{1}{N}\;\sum {\mathrm{\backslash nolimits}}_{i=1}^{N}(X_{i}-\;\overset{―}{X})\;{(X_{i}-\;\overset{―}{X})\;}^{T}$$ (9)

Where $X_{i}\;$= input feature vector,

$\overset{―}{X}$= mean vector of data,

N = number of data points.


$$\text{Eigenvalue\textbackslash{} Decomposition}:\;C_{V}=\lambda_{V}$$ (10)

Where: v = eigenvectors (principal components),

λ = eigenvalues (variance explained by each component).


$$\text{Projection\textbackslash{} onto\textbackslash{} Principal\textbackslash{} Components}:\;z\;=\;V^{T}(X\;-\;\overset{―}{X})\;$$ (11)

Where: z = transformed feature vector in PCA space,

V = matrix of eigenvectors.

Fig 5 illustrates that the wavelet-transformed characteristics (blue dashed lines) offer a refined depiction of the original features (black lines). This process effectively captured both temporary fluctuations and persistent patterns, hence refining the datasets and enhancing pattern identification skills. It is significant to note that monitoring sites such as DC Stadium, Dwarka Sec 8, and Nehru Nagar had similar peak patterns, suggesting common external impacts, whereas Ashok Vihar and Okhla demonstrated more stable trends within the altered signals. In contrast, Najafgarh and Dwarka Sec 8 had significant swings, indicating rapid changes in air quality. Additionally, a slight delay was seen between the original data and the wavelet-transformed data, a typical occurrence resulting from wavelet decomposition. This preprocessing technique augments the feature set by preserving substantial differences while reducing the impact of random fluctuations.

**Fig 6:**
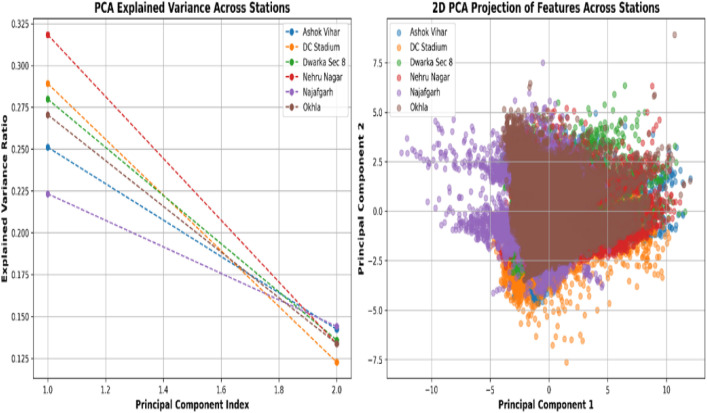
Principal component analysis (PCA) of air quality data across six monitoring stations.

**Fig 7:**
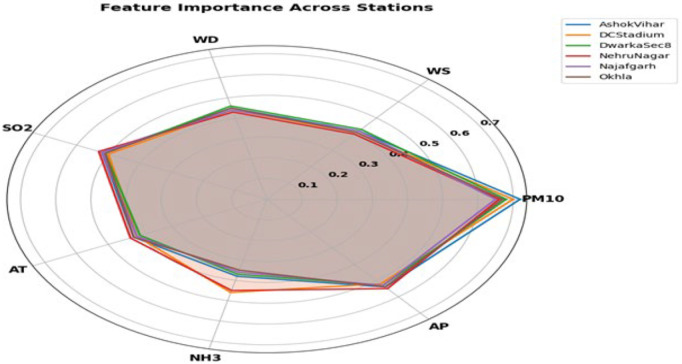
Radar chart of feature importance. The significance of seven factors for forecasting PM₂.₅ at six Delhi stations. Lines further from the center imply more importance.

Principal Component Analysis (PCA) was utilized to diminish dimensionality in air quality datasets while maintaining essential underlying patterns. Fig 7 (left) illustrates the explained variance, revealing that the first principal component (PC1) accounted for the most significant variance (~20–32%) across all monitoring stations, followed by the second (PC2), which captured approximately 12–18% of the variance. Nehru Nagar had the most explained variance for PC1, indicating superior linear separability in its data structure; conversely, Najafgarh demonstrated the least, reflecting increased complexity in its data distribution. Furthermore, in Fig 6 (right), the two-dimensional PCA projection of features demonstrates significant overlap across the different stations, suggesting that their feature distributions lack total individuality. While some natural clustering was evident, the definition of station-specific separations was insufficiently articulated, and instances of outliers were observed. DC Stadium exhibited a wider dispersion, signifying a higher level of unpredictability, whereas Najafgarh displayed a more concentrated distribution. These data indicate that PCA effectively reduces complexity and preserves significant patterns. Wavelet Transform (WT) identified critical trends. Still, Principal Component Analysis (PCA) decreased dimensionality while maintaining significant variation following a feature selection approach to optimize feature selection and improve predictive accuracy.

### 4.4. Feature selection

A metaheuristic algorithm hybridizes the Aquila Optimizer - Arithmetic Optimization Algorithm (AOAOA) to identify important characteristics after the extraction in air quality prediction. The problem of feature selection is presented as a complex optimization challenge to reduce unnecessary and redundant input components while maintaining the accuracy of the classifier. Traditional approaches to feature selection are criticized for their tendency to include an excessive number of irrelevant characteristics. On the other hand, the AOAOA algorithm addresses this problem by using an adaptive learning heuristic that carefully finds and picks highly relevant characteristics. The search space for the AOAOA includes all possible combinations of attributes that may be extracted that may be extracted from the dataset. This allows for a comprehensive and flexible method of selecting features [37].

#### 4.4.1 Aquila optimizer.

The Aquila Optimizer (AO) is a contemporary algorithm developed in 2021 by Abualigah et al [38]. It falls within the domain of swarm intelligence. The design is influenced by the varied hunting methods of the Aquila, a kind of eagle known for its exceptional agility and power. The AO algorithm is organized into four distinct stages that mirror the hunting behaviors of the Aquila [39].

Elevate with a vertical Dive: The phase of flight refers to the Aquila’s ability to fly at high altitudes while searching for prey from above. Within the algorithm, this corresponds to doing an extensive exploration of the potential solutions [40].

In Curve Flying with Short Glide: The Aquila aircraft flies at a lower altitude, indicating a more concentrated search in a potentially beneficial area according to the algorithm.

Flight at a low altitude with a gradual decrease in altitude:

Aquila’s strategy toward its aim is reflected in the algorithm via a deliberate and methodological search for the most favorable solution.

Walking and capturing: The last stage, in which the Aquila seizes its prey by walking, corresponds to the algorithm’s meticulous adjustment of the most optimal solution discovered.

The shift from the first phase of wide search to the subsequent phase of fine-tuning in the AO algorithm is defined by the current iteration about the maximum number of iterations permitted. If the present iteration is under or equal to than or equal to two-thirds of the greatest, the algorithm prioritizes exploration. After reaching that stage, it transitions to the act of taking advantage of something [41].

**Expanded exploration**: Aquila’s preliminary aerial hunt for prey at great heights. Once the most favorable hunting area is identified, the algorithm replicates Aquila’s rapid descent towards the selected prey. This technique is defined by a particular equation that directs the algorithm’s search pattern. During the “Expanded Exploration (X1)” procedure, the algorithm utilizes a comprehensive perspective to determine the search area and then focuses on the most optimal region, imitating Aquila’s effective hunting dive [42].


$$X_{1\;}(\text{t}+1)\;=\;X_{best\;}(\text{t})\;\times (1-\frac{t}{T}\;)\;+\;(X_{mean}(\text{t})\;-X_{best}(t)\text{ rand})\;$$ (12)

$X_{1\;}$(t+1) is denoted as a result of the next iteration of time at t, then is genetared using the initial search method $X_{1\;}$. $X_{best\;}$present the most favorable solution at time t. $(1-\frac{t}{T}$) is managing exploration. T and t refer to the highest number of iterations and the current iteration. $X_{m\;}$ it signifies the mean location value for the whole population. which may be calculated using this equation


$$X_{mean}(\text{t})\;=\;\frac{1}{N}\;\sum {\mathrm{\backslash nolimits}}_{I=1}^{N}X_{i\;}(\text{t})$$ (13)

**Narrowed exploration:** The Aquila mostly use this hunting method. Once Aquila detects the location of its target, it transitions from flying at an elevated height to remaining right across it. The target is aggressively seeking a favorable opportunity to initiate an assault.


$$X_{2\;}(\text{t}+1)\;=\;X_{best\;}(\text{t})\;\times \;Levy\;(D)\;+(\;X_{R}(\text{t})\;+\;(y\;-\;x)\;*\text{ rand})$$ (14)

$X_{2\;}$(t+1) is denoted because of the next iteration of time at t, $X_{best\;}$ present the most favorable solution at the time t, $X_{R}$(t) denotes a randomly selected answer from the range of integers between 1 and N. The term “rand” represents a random number that falls within the range of 0–1. The spiral form of the search is represented by x and y. It is represented as an equation below.


x = r ×sin(θ) (15)

y = r ×cos(θ) (16)

In addition, the Levy flight probability activity, denoted as Levy(D) is presented and σ is computed through an equation using the equations below.


$$\text{Levy }(\text{D})\;=\;S\;\times \;\frac{U\;\times \sigma }{{\left|V\right|}^{\frac{1}{\beta }}}$$ (17)

$$\;\sigma \;=\;\frac{\tau \;(\;1+\;\beta )\;\times \;\sin e\;(\frac{\beta \pi }{2})}{\tau \;(\frac{1+\beta }{2})\;\times \;\beta \times 2^{\left(\frac{\beta -1}{2}\right)}}$$ (18)

In this context, Levy(D) represents the Levy flight probability activity, here in D-dimensional space. The default value given to s is 0.1. Let u and v be chosen from the interval {0,1}. This has a fixed value of 0.5. and one of the parameters σ is in the Levy flight distribution function.

**Expanded exploitation:** The “Expanded Exploitation” phase refers to the third hunting tactic used by Aquila. This strategy entails a covert and close-to-the-ground approach, gradually descending near the reduce the proximity to the target before commencing an assault [43]. In this strategy, after Aquila has a rough estimation of the prey’s whereabouts, it begins a deliberate descent, executing a first attack to assess the prey’s response.


$$X_{3\;}(\text{t}+1)\;=\;{(\;X}_{best\;}(\text{t})\;\times \;(\;X_{mean}(\text{t}))\;\times \;\alpha \;-\;rand\;+((\;UB\;-\;LB)\;\times rand\;+LB\;)\times \delta \;$$ (19)

T represents the greatest possible count of repetitions. UB and LB stand for Upper Bound and Lower Bound, respectively. α and δ indicate static exploitation adjustability variables.

**Narrowed exploitation**: Now, the program replicates Aquila’s strategic drop to the earth’s surface strategic face, attentively tracking the unpredictable motions of the prey and getting ready for a decisive attack. The last stage of this procedure is the “Narrowed Exploitation” phase, which utilizes Aquila’s latest and most straightforward form of assault, referred to as the “walk and grab”. The last stage is precisely specified mathematically, encapsulating the intentional and strategic aspect of Aquila’s ultimate predatory maneuver.


$$X_{4\;}(\text{t}+1)\;=\text{ QF }\times \;{\;X}_{best\;}(\text{t})\;-\;\left(G_{1}\;\times X_{i}(\text{t})\times \;rand\right)-\;(\;G_{2\;}\times \;Levy\;(D)\;+rand\times \;G_{1})\;$$ (20)

$${\text{QF}}_{\text{t}}\;=\;t^{\frac{2\;\times rand\;-1}{{\left(1-T\right)}^{2}}}$$ (21)

$$G_{1}\;=\;2\;\times rand\;-1$$ (22)

$$G_{2\;}=\;2\;\times (1-\frac{t}{T}$$ (23)

The value of $G_{1}$ reflects Aquila’s changing movement throughout the chase, oscillating randomly within a given range to imitate the real approach. $G_{2\;}$ depicts the path of Aquila’s flight, which gradually decreases in a deliberate and systematic drop. The quality function, represented by the QF, is used to fine-tune the search mechanism, enhancing the algorithm’s efficiency as it tries to find the target.

#### 4.4.2 Arithmetic optimization algorithm.

The Arithmetic Optimization Algorithm (AOA) is an optimization technique that extracts inspiration from fundamental arithmetic principles. Employment is the operation of multiplying, dividing, subtracting, and adding to go through two crucial stages in the process of optimization: exploration and exploitation. During the exploration phase, the method employs multiplication and division procedures. These processes provide a wide range of values, which range of values, which help in conducting a thorough examination of potential solutions. This variety is essential for preventing premature commitment to suboptimal solutions. Conversely, the exploitation phase is dependent on the operations of subtraction and addition. These processes provide a more sophisticated method, improving the algorithm’s capacity to adapt and focus on the most promising possibilities, finally resulting in the optimal output [44].

Initialization: The starting population X of the AOA is produced arbitrarily. The population count is denoted as N × D, where N denotes the total number of individuals in the population, with D indicates the number of dimensions for each member of the population [43].

The AOA searching procedure determines the subsequent action using MOA functionality,


$$\text{MOA}(\text{ c })\;=\text{ Min }+\text{ c }\times \;\frac{Max\;-Min}{T}$$ (24)

The variable C denotes the current stage of iteration, whereas T represents the entire count of iterations. MOA(c) represents an operation that accelerates the process of iteration c. The minimum value is 0.2 and the maximum value is 1.

**Exploration and exploitation:** In the AOA, the exploration stage employs a global search method that applies Division (D), and multiplication (M) operations. These operators are selected for their capacity for creating a diverse range of computations in mathematics. The Division operator can disperse values, which makes it difficult to quickly converge on the optimal solution. On the other hand, the Multiplication operator can produce a narrowly focused set of values. The inherent capacity of these operators to either propagate out or focus values makes them especially important during the search phase of the methods, as they aid in thoroughly exploring the solution space.


$$\;xij\;(t+1)\;=\left\{ \begin{array}{c} best\;(x_{j}\;)\;÷(MOP\;(c)\;+\;\varepsilon \;)\;\times (({UB}_{j}\;-{LB}_{j\;})\;\times \;\mu \;+{LB}_{j}),\;r_{2\;}<0.5\\ best\;(x_{j}\;)\;\times (MOP\;(c)\;\times (({UB}_{j}\;-{LB}_{j\;})\;\times \;\mu \;+{LB}_{j}),\;otherwise \end{array}\right.\;$$ (25)

$$MOP\;(c)=\;1\;-\;\frac{C^{\frac{1}{\alpha }}}{T^{\frac{1}{\alpha }}}$$ (26)

Here ε  is a numeric with a low value and μ is a controlling variable that remains constant at 0.5 over the search operation. The upper and lower bounds of the jth location are denoted by ${UB}_{j\;}and\;{LB}_{j\;}$ accordingly [45]. The math Optimizer Probability (MOP) is a coefficient that represents the sensitivity of the coefficient, which is set at a fixed value of 5 and is used to determine the level of precision in improvement [46].

The exploitation technique in AOA employs arithmetic operators, namely subtraction and addition, to get a high level of outcomes using computational methods in the local search. This is possible owing to the low dispersion and ease of approaching the target associated with these operations.


$$xij\;(t+1)\;=\;\left\{ \begin{array}{c} best\;(x_{j}\;)\;-(MOP\;(c)\;\times (({UB}_{j}\;-{LB}_{j\;})\;\times \;\mu \;+{LB}_{j}),\;r_{3\;}<0.5\\ best\;(x_{j}\;)\;+(MOP\;(c)\;\times (({UB}_{j}\;-{LB}_{j\;})\;\times \;\mu \;+{LB}_{j}),otherwise \end{array}\right.\;$$ (27)

#### 4.4.3 Enhanced integration Of AOAOA: Rationale and algorithmic details.

**Fig 8:**
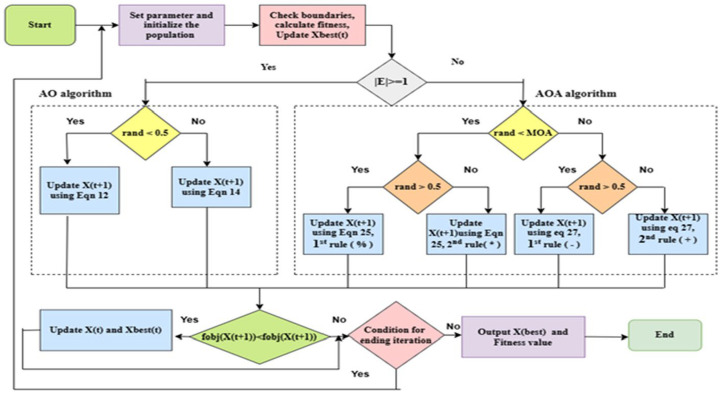
The sequence diagram illustrates the implementation of the hybrid AOAOA approach.

The rationale for integrating the Aquila Optimizer (AO) with the Arithmetic Optimization approach (AOA) arises from their synergistic search behaviors, which mitigate the shortcomings inherent in utilizing either approach independently. AO, drawing inspiration from the hunting techniques of the Aquila eagle, proficiently navigates the global search space, swiftly pinpointing potential areas while evading premature convergence to local minima. Nonetheless, its exploitation capability, refining the solution inside the designated area, may be constrained, occasionally leading to inferior outcomes. Conversely, AOA, utilizing arithmetic operations, offers robust local exploitation via its adaptive search mechanism, effectively honing candidate solutions but maybe exhibiting a deficiency in exploratory variety. This study integrates AO and AOA into the AOAOA architecture, utilizing the global exploration capabilities of AO and the local exploitation accuracy of AOA. This synergy guarantees a more balanced and efficient optimization method, enhancing feature selection precision while alleviating overfitting and decreasing computational expenses. Table 3 presents the pseudo-code for the hybridized AOAOA technique. When comparing Eq. 12,14,25, and 27, it can be shown that those people in AO swarms engage in a higher level of random searching compared to those in AOA swarms. Nevertheless, in the process of exploitation described by Eq. 19,20,25, and 27, alone in the AO swarms might demonstrate lower results compared to those in the AOA swarms. While both methods demonstrate strong optimization performance, the exploitation skill of people in AO swarms is unsatisfactory. Additionally, the exploration power of individuals in AOA swarms is less competent compared to alone in AO swarms. Hence, merging the exploration process alone in AO swarms and the exploitation process for people in AOA swarms would be more advantageous [29]. Fig 8 presents the sequence diagram that illustrates the implementation of the hybrid AOAOA approach.

**Table 3:** The section that follows the pseudo-code for the hybridized AOAOA technique.

1. Initialize the population size N. 2. Initialize the maximum number of iterations T. 3. Set dimension D. 4. Initialize the position of individuals x1(i=1,2,3. …. n 5. While (t <= T) 6. Update the MOA and MOP using Eqn (24,26) 7. Update x,y using Eqn (16and (17) 8. for i= 1:N 9. if |E|>= 1 10. if rand <0.5 11. Update the position of X(t +1using Eqn (12) 12. Else 13. Update the position of X(t+1using Eeq (14) 14. End if 15. Else 16. If rand < MOA // Exploration stage 17. if rand > 0.5 // Employ the Division operator (``÷ " ) 18. Update the position ofX(t+1)using the first rule of Eeq (25) 19. Else : // Employ the Multiplication operator (`` × " ) 20. Update the position of X(t+1using the second rule of Eqn (25) 21. End if 22. Else // Exploration stage 23. If rand > 0.5 // Employ the subtraction operator (``−`` ) 24. Update the position of X(t+1using the first rule of Eqn (27) 25. Else // Employ the addition operator (``+`` ) 26. Update the position of X(t+1using the second rule of Eqn (27) 27. End if 28. End if 29. Else if 30. End For 31. For i= 1: N //Check if the position goes out of the search space boundary 32. X(t) = enforce boundaries (X(t)) 33. Calculate the fitness of X(t) 34. Fitness = calculating fitness (X(t)) 35. # Update Xbest (t) if the new solution is better 36. $\;If\;fitness<best_{fitness}:$ 37. Xbest= X(t) 38. Best_fitness = fitness 39. Update Xbest (t) 40. t=t+1# Increment iteration 41. End while 42. # Return the best solution found 43. ReturnXbest(t)

Fig 7 depicts the feature significance scores for various air quality indicators across six monitoring stations, emphasizing the relative contribution of each feature to the predictive model. The radar graphic consists of seven axes that denote essential variables: PM10, wind speed (WS), relative humidity (RH), sulphur dioxide (SO₂), air temperature (AT), ammonia (NH₃), and air pressure (AP). Each colored line represents a distinct monitoring station — Ashok Vihar, DC Stadium, Dwarka Sec 8, Nehru Nagar, Najafgarh, and Okhla — illustrating the fluctuation in feature significance at each site. Regions nearer to the periphery denote elevated significance scores, whilst shaded areas depict the variability among stations. The research reveals that PM10 is the predominant factor across all locations, followed by SO₂, AP, and, to a lesser degree, WS and RH, emphasizing the significant impact of particulate matter and climatic variables on PM2.5 prediction. Conversely, NH₃ and AT consistently exhibit diminished significance ratings, indicating a relatively insignificant contribution to the predictive framework. This figure offers a distinct comparison analysis of feature importance, highlighting the primary influence of PM10 and associated meteorological elements in air quality evaluation.

### 4.5 Prediction

After feature selection, the retrieved features are fed into a prediction method that employs a Bi-LSTM network. This specific deep-learning approach is highly observed for its effectiveness in analyzing time series datasets about air quality. It has been experimentally proven to achieve the maximum level of accuracy in predictive modeling.

#### Bi-LSTM prediction.

**Fig 9:**
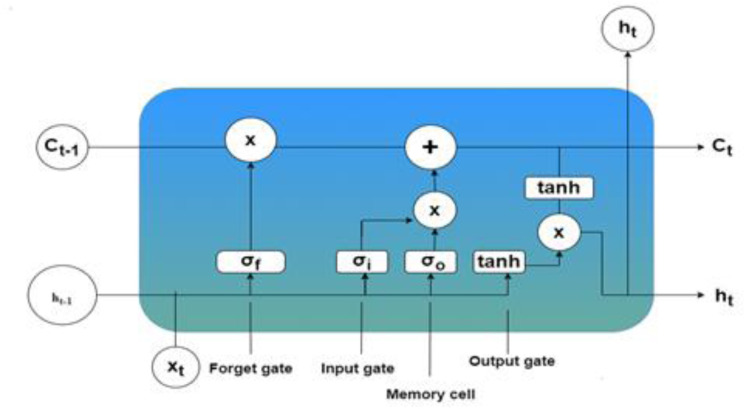
Generic network architecture of the LSTM.

RNNs and LST networks are specific types of RNNs. It is specifically intended to process sequential input and can generate. Diverse outputs for the same input by learning temporal relationships. The LSTM model is presented in Fig 9. The generic network architecture of the LSTM improves this ability by collecting extended relationships in sequential data via a sophisticated arrangement of gates inside its hidden layers. These gates regulate the transmission of information, enabling the network to either keep or delete input depending on its relevance to the prediction goal. LSTMs use three primary gates- forget, input, and output to order the memory cells of the network, which retain the temporal state of the network [47]. The forget gate is essential for selectively removing less relevant data, while the activation function provides weights to the input to decide its importance. Subsequent neurons receive and process high-weight information.

**Fig 10:**
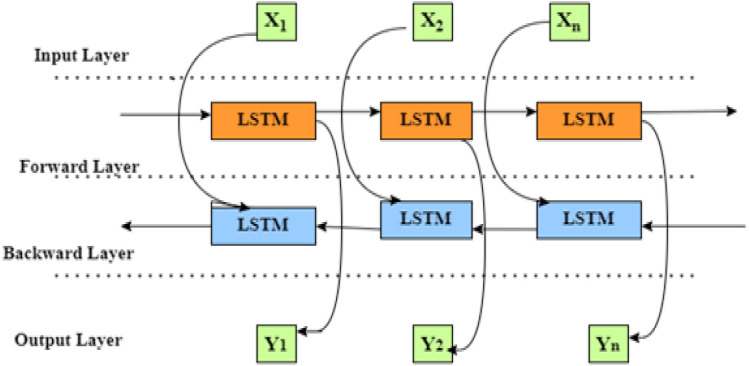
Air quality forecast is based on the Bi-LSTM framework.


The Bi-LSTM is a kind of bidirectional recurrent neural network that processes a sequence of information in both forward and backward directions. Fig 10 demonstrates that the Air quality forecast is based on the Bi-LSTM framework [48]. The cell state memory, which utilizes past and future information to predict output, the input sequence represented as X = (X_1,_ X_2,…………..,_ X_n),_ Bi-LSTM forward direction is represented as $\vec{h}=\;({\vec{h}}_{1},\;{\vec{h}}_{2},\;\ldots \ldots .{\vec{h}}_{n})$, and backward direction represented as $\;\overset{\leftarrow }{\overset{\leftarrow }{h}}=\;\overset{\leftarrow }{h_{1}},\;\overset{\leftarrow }{h_{2}},........,\overset{\leftarrow }{h_{n}}$) [49]. The ultimate cellular output, denoted as $y_{t}$ is generated by a process of combination ${\vec{h}}_{t}$ and $\;\overset{\leftarrow }{h_{t}}$The resulting final sequence is represented as $y\;=\;(y_{1},\;y_{2},........y_{n})$ [9].

$${\vec{h}}_{t}\;=\text{ H }(\;W_{ph}P_{t}\;+\;W_{hh\;}h_{t-1}+\;b_{h})$$ (28)

$$\;\overset{\leftarrow }{h_{t}}=\text{ H }(\;W_{ph}P_{t}\;+\;W_{hh\;}h_{t+1}+\;b_{h})$$ (29)

Where ht – hidden state at timestamp t,

$W_{ph}$: refers to the weight matrix connecting the input and hidden vector states.

$P_{t}$: refers to the input vector at a timestamp t.

$W_{hh\;}$The weight vector relating to two hidden states

$h_{t+1}$: the hidden state vector at a timestamp t + 1

$b_{h}$The bias vector is employed for the hidden state vectors.


$$q_{t}=\;W_{pq}{\vec{h}}_{t}+\;W_{pq}\;\overset{\leftarrow }{h_{t}}\;+\;B_{q}$$ (30)

The output values $q_{t}$ are obtained by adding the weight matrices of the input and output, which is achieved by succeeding the dot product of the forward hidden layers ${\vec{h}}_{t}$ and the backward hidden layers. The process involves creating layers by applying a hyperbolic tangent function and including a fixed bias $B_{q}$. Fig 10 displays the air quality forecast based on the Bi-LSTM framework.

### 4.6 Model development, experimental setup, and evaluative metrics

The proposed AquaWave-BiLSTM air quality prediction model includes feature extraction, feature selection, and predictive modeling utilizing a Bidirectional Long Short-Term Memory (BiLSTM) network. The dataset comprises air quality measurements from six monitoring stations: Ashok Vihar, DC Stadium, Dwarka Sec, Nehru Nagar, Najafgarh, and Okhla. Each station has 11,704 rows and 20 columns. A hybrid feature extraction method was utilized, integrating Wavelet Transform (WT) with Daubechies-4 (db4) at a decomposition level of 3 and main Component Analysis (PCA) to extract and diminish frequency-based features, choosing the top 10 main components. Feature selection was conducted via the Aquila Optimization–Arithmetic Optimization Algorithm (AOAOA), initialized with a population size of 50 and executed over 100 iterations. The values were determined after a grid search of several combinations (population sizes: 30, 50, 70; iterations: 50, 100, 150), with the chosen configuration yielding steady convergence and reliable prediction performance while maintaining computing efficiency. A feature significance criterion of 0.40 was experimentally established after evaluating various values (0.2 to 0.6) to retain relevant characteristics while reducing duplication. The AOAOA technique methodically improved candidate solutions while preserving an equilibrium between exploration and exploitation. The BiLSTM architecture consists of two BiLSTM layers, each containing 50 hidden units, followed by a dense output layer. The model utilized the Adam optimizer with a learning rate of 0.0005 and employed Mean Squared Error (MAE) as the loss function. The dataset underwent normalization via MinMaxScaler, and an 80:20 train-test split, cognizant of temporal factors, was used, using the first 80% for training and the subsequent 20% for testing, so maintain chronological integrity and avert data leakage for an authentic performance assessment. Ashok Vihar, Nehru Nagar, Okhla, DC Stadium, Dwarka Sec, and Najafgarh comprised 9,363 instances of training and 2,341 validation instances for each station. The model underwent training for 100 epochs with a batch size of 64, utilizing early stopping with a patience of 10 epochs and a learning rate reduction strategy to mitigate overfitting. Table 4 presents the Model’s architecture, compilation, training, and evaluation metrics. The hyperparameter selection for the AquaWave-BiLSTM model was informed by grid search, validation performance, and analogous work on time series prediction. The quantity of hidden units (50) in each BiLSTM layer was determined to strike a compromise between learning capability and the danger of overfitting, particularly considering the moderate dataset size (11,704 samples per station). A learning rate of 0.0005 facilitated smooth convergence and decreased the probability of bursting or disappearing gradients throughout training. A batch size of 64 facilitated reliable updates while preserving computational performance. The leading 10 principal components in PCA were chosen since they preserved over 95% of the cumulative variance, hence reducing information loss. The AOAOA settings, comprising a population size of 50 and 100 iterations, were chosen through empirical tuning and conform to the suggested defaults in optimization research. A feature selection criterion of 0.4 effectively balanced model simplicity with predictive accuracy.

**Table 4:** Model’s architecture, compilation, training, and evaluation metrics.

Category	Hyperparameter	Setting
**Feature Extraction**	Wavelet Type	db4
	Wavelet Decomposition Level	3
	Selected Coefficients (Wavelet)	50
	PCA Components	10
**Feature Selection**	Population Size (AOAOA)	50
	Iterations (AOAOA)	100
	Feature Selection Threshold	0.40
**BiLSTM Model**	Model Type	Bidirectional LSTM
	BiLSTM Units	100
	LSTM Activation Function	ReLU
	Dropout Rate	0.4
	Dense Layer Units	64
	Optimizer	adam
	Learning Rate	0.0005
	Loss Function	Mean Absolute Error (MAE)
**Training Strategy**	Batch Size	64
	Epochs	50
	Validation Split	0.2
	Train-Test Split	80% Train, 20% Test
	Early Stopping Patience	10 epochs
**Learning Rate Tuning**	Learning Rate Reduction Patience	5 epochs
	Learning Rate Reduction Factor	0.5
	Minimum Learning Rate	1e-6

The performance evaluation included Mean Squared Error (MSE), Mean Absolute Error (MAE), Root Mean Squared Error (RMSE), and R² Score, indicating that the hybrid feature extraction and selection approach markedly improved prediction accuracy at all monitoring stations. Furthermore, an analysis of feature significance was conducted to ascertain the most significant elements influencing differences in air quality. The study was performed using a Windows 10 operating system, including an Intel i5-8400 CPU (2.80 GHz), an NVIDIA GeForce GTX1060 graphics card (5 GB memory), and 24 GB of RAM. The whole development process, encompassing data processing, model construction, and assessment, was executed utilizing Python 3.6 with open-source modules including Pandas, NumPy, and PyTorch, so providing an effective and adaptable workflow.

#### Performance metrics.

Many evaluation metrics are employed to evaluate the accuracy of the models developed on regression. These metrics present empirical measurements of the precision and efficiency of the models.

**Mean Absolute Error:** is a statistical measure employed to determine the average difference between the actual and projected values in the test data. It offers an internal. The calculation involves determining the mean of the predictive errors, as demonstrated by the equation as follows [50].


$$MAE\;=\frac{1}{n}\;\sum_{i=1}^{n}|y_{i\;-}{y_{i\;}}^{\prime }|\;$$ (31)

**Root Mean Square Error:** is very responsive to inaccuracies in the significance of predicted outcomes within the dataset, therefore serving as a good metric for assessing the accuracy of predictions. A lower RMSE value indicates superior performance. The equation is used for determining this error.


$$\text{RMSE}=\sqrt{\frac{1}{n}\;{\sum {\mathrm{\backslash nolimits}}_{i=1}^{n}(y_{i\;-}{y_{i\;}}^{\prime })}^{2}\;}\;$$ (32)

**R**^**2**^ **Score:** is commonly denoted to as the value of the coefficient of determination, is a metric used to measure the variability of the desired variables within a model. The range is from 0 to 1, with a greater number indicating the model’s fit to the data source in a satisfactory manner.


$$R^{2}=\;1-\;\frac{\;{\sum_{i=1}^{n}(y_{i\;-}{y_{i\;}}^{\prime })}^{2}}{\;{\sum_{i=1}^{n}(y_{i\;-}{y_{i\;}}^{\prime })}^{2}}$$ (33)

**Mean Squared Error**: measures the accuracy of an approach by calculating the average of the expected and actual values. A small MSE indicates that a model has a higher level of efficiency in predicting the data.


$$MSE=\;\frac{{\sum_{i=1}^{n}(y_{i\;-}{y_{i\;}}^{\prime })}^{2}}{n}\;$$ (34)

## 5. Results

**Fig 11:**
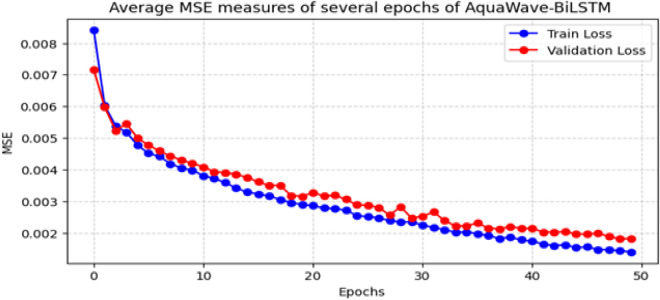
Graphical representation of MSE measures of several epochs of AquaWave –BiLSTM.

**Fig 12:**
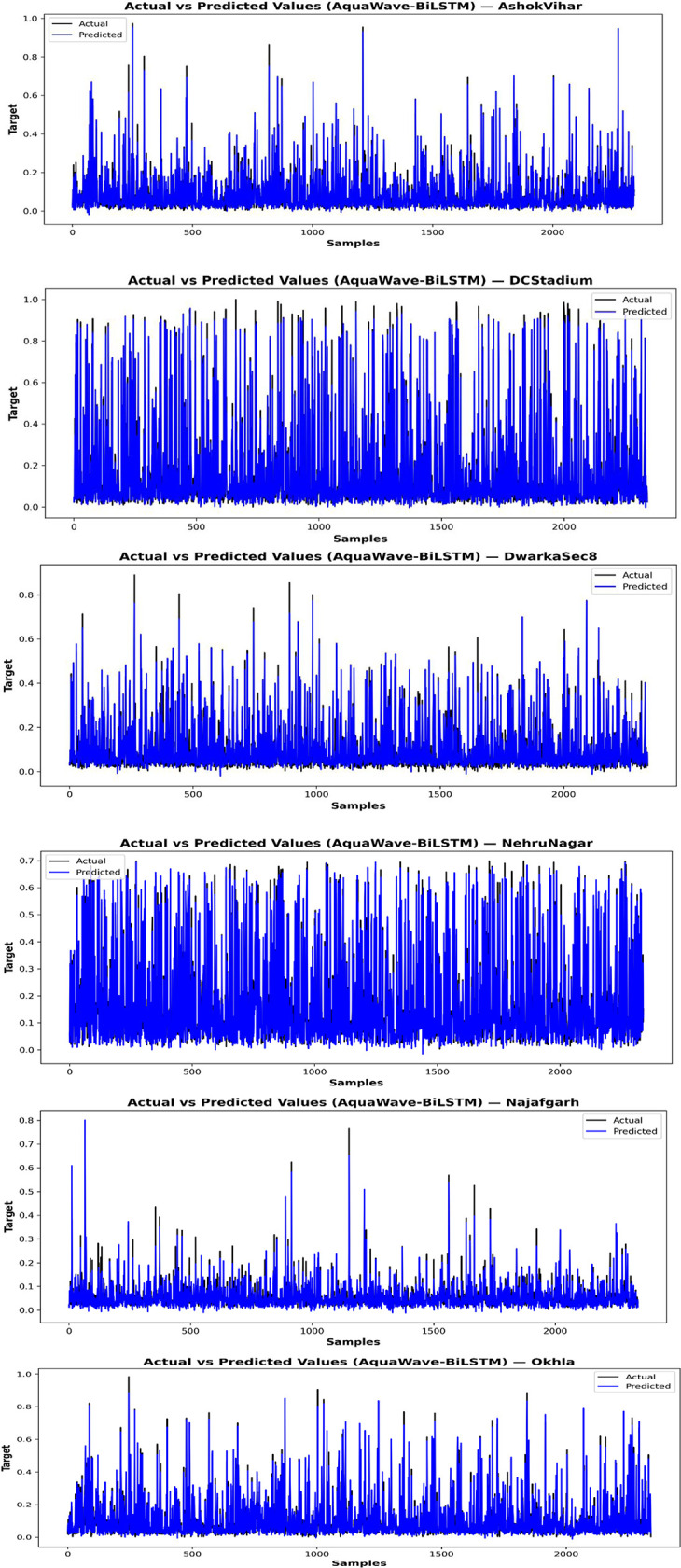
Graphical representation: actual vs. predicted PM2.5 for multiple stations.

The AquaWave-BiLSTM model incorporates Wavelet Transform to extract essential frequency-based data and employs PCA for dimensionality reduction, hence improving its capacity to discern intricate air quality patterns. The AOAOA were employed to further optimize feature selection, achieving an appropriate balance between exploration and exploitation. This method preserved just the most pertinent characteristics, decreasing computing complexity while enhancing forecast accuracy. Fig 11 depicts the progression of MSE throughout several training rounds. The continual reduction in MSE indicates the model’s efficient learning process. In contrast to traditional models susceptible to overfitting superfluous features, the refined feature selection process facilitated consistent convergence between training and validation losses. This illustrates the model’s capacity to generalize effectively without necessitating premature cessation at a predetermined period. Additionally, a comparison study demonstrates that the optimized AquaWave-BiLSTM model has enhanced stability, sustaining reduced MSE values during both training and validation stages. The use of sophisticated optimization methods guarantees that the AquaWave-BiLSTM model maintains computational efficiency and resilience in air quality predictions. The AquaWave-BiLSTM framework for multi-station air quality forecasting attained an average MSE of 0.00065, MAE of 0.04566, RMSE of 0.02523, and R² of 0.9494. The complete execution of the AquaWave-BiLSTM model, encompassing the Colab notebook, code, and intermediate results, is available in S2 File in S1 Data for replication and reference. Fig 12 illustrates the actual vs forecasts for several stations, showcasing the model’s capacity to capture variations in air quality trends at various monitoring sites. Despite rare underestimations during strong pollution spikes, the model exhibits consistent performance over most of the test range. These findings further corroborate the efficacy of the suggested feature selection and refining methodologies, guaranteeing a dependable and scalable forecasting strategy for air quality evaluation.

### 5.1 Impact of feature extraction on model performance

The efficacy of the AquaWave-BiLSTM model was assessed both with and without feature extraction to illustrate the influence of Wavelet Transform, PCA, and AOAOA feature selection on air quality forecasting. The analysis demonstrates a notable enhancement in model accuracy when implementing feature extraction before feature selection.

#### With feature extraction (Wavelet transform + PCA + AOAOA Feature selection).

This method utilized Wavelet Transform to identify essential frequency-based patterns, while PCA decreased dimensionality by preserving just the most significant features. The AO and AOA optimization methods subsequently identified the most pertinent features, balancing exploration and exploitation to improve computing efficiency. This diminished model complexity curtailed duplicate characteristics and enhanced predictive stability. The BiLSTM model demonstrated a decreased Mean Squared Error (MSE) and elevated R² values, signifying improved generalization and less overfitting at all monitoring stations.

#### Without feature extraction (AOAOA feature selection on raw data).

**Fig 13:**
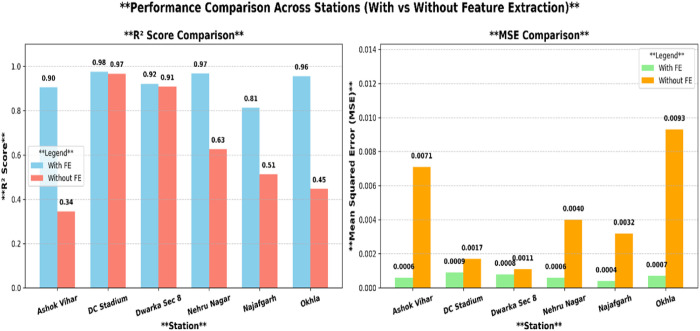
Visual representation of R² score and MSE comparison across stations with and without feature extraction.

Conversely, in the absence of feature extraction, the model utilized AO and AOA for feature selection straight from raw data, which encompassed redundant and less pertinent variables. Although AOAOA optimized feature selection, the existence of unprocessed data resulted in elevated MSE values and diminished R² scores, indicating a reduction in prediction ability. The lack of Wavelet Transform and PCA led to heightened computational complexity and an elevated danger of overfitting, as the model endeavored to learn from noisy or less relevant patterns. Fig 13 illustrate the R² Score and MSE Comparison across stations with and without feature extraction, demonstrating the effectiveness of the AquaWave-BiLSTM model in multi-station air quality forecasting. These findings validate that the combination of feature extraction with AOAOA optimization augments air quality forecasting precision by eliminating superfluous variables, mitigating overfitting, and enhancing model efficacy.

The above plot presents the comparative evaluation of the Bi-LSTM model’s effectiveness across six monitoring stations, assessed with and without feature extraction. The left panel displays the R² score comparison, illustrating the range to which the model accounts for the variable in air quality. The right panel depicts the Mean Squared Error (MSE), measuring prediction errors. Feature extraction—comprising Wavelet Transform, PCA, and AOAOA optimization—substantially increases efficiency for most stations (e.g., AshokVihar, NehruNagar, Najafgarh, Okhla), as indicated by improved R² scores and much lower MSE values. Without extraction, efficiency drops owing to duplicated or noisy input features. These findings confirm the significance of preprocessing in improving temporal prediction precision across diverse air quality monitoring locations.

### 5.2 Feature selection comparison for air quality prediction: AOAOA vs. other optimization techniques

**Fig 14:**
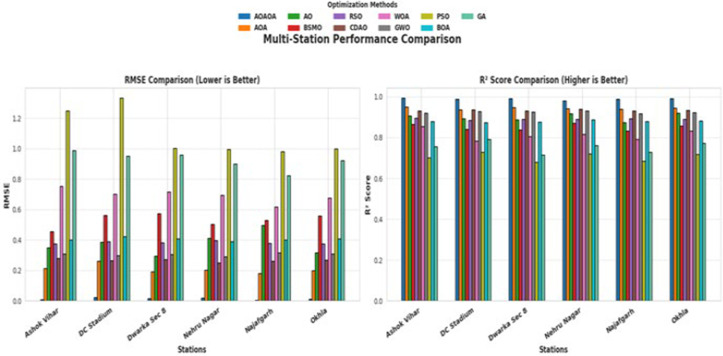
Multi-station performance comparison of feature selection methods for air quality prediction: AOAOA vs. competing algorithms.

This Fig 14 illustrates a comparative analysis of several feature selection algorithms- AOAOA, AOA, AO, BSMO, RSO, CDAO, WOA, GWO, PSO, and GA regarding their efficacy in air quality forecasting techniques, assessed across six monitoring stations: Ashok Vihar, DC Stadium, Dwaraka Sec, Nehru Nagar, N Najafgarh, and Okhla. The RMSE comparison reveals that AOAOA attains one of the lowest RMSE values, underscoring its high forecasting precision. CDAO and GWO have comparably low RMSEs, suggesting competitive efficacy. In contrast, PSO and GA regularly provide the greatest RMSE values, indicating a limited ability to capture pertinent information for precise air quality prediction. The comparison of R² Scores further corroborates these facts. AOAOA, CDAO, and GWO are identified as leading performers, with elevated R² values that signify robust predictive correlation and consistency across all stations. Conversely, PSO, GA, and WOA have markedly lower R² values, indicating diminished generalization ability and model adequacy. This visual study substantiates the efficacy of the AOAOA–BiLSTM framework. The AOAOA approach adeptly equilibrates exploration and exploitation [51], alleviating local optima and diminishing the likelihood of overfitting. It surpasses alternative methods in both prediction accuracy and computing efficiency, attaining an **average RMSE of 0.0252, a R² of 0.9494, a training duration of 20.57 seconds, and an inference duration of 1.1169 seconds** per batch across all stations. This comparative evaluation establishes that AOAOA, succeeded by CDAO and BSMO, demonstrates enhanced efficacy in air quality prediction tasks. These results endorse the amalgamation of ordered feature selection with deep learning models for adaptable, precise, and efficient environmental forecasting systems.

### 5.3 Comparative analysis of machine and deep learning algorithms for multi-station air quality data

The proposed AquaWave-BiLSTM system has been analyzed with a range of classifiers to predict air quality [52]. The classifiers consist of SVM, RF, RNN, LSTM, GRU, Bi-LSTM, Bi-GRU, and Bi-LSTM Attention, AOAOA-BI-LSTM mechanism. These were compared to the recently released model via benchmarking. Within the domain of sequential forecasting, the Bi-LSTM architecture stands out through its distinct gates, namely the reset and update gates. Such gates play a crucial role in reducing computing loss, enhancing the model’s ability to store data for a long time, and addressing the gradient vanishing problem. The AquaWave-BiLSTM model attained enhanced accuracy at a marginally increased computational expense, thereby balancing accuracy and efficiency in air quality forecasting. Table 5 contrasts computation durations for diverse prediction tasks, using several machine learning and deep learning methods applied to multi-station air quality datasets. Despite the AquaWave-BiLSTM model’s slightly greater computing demand compared to baseline models, its superior predictive performance warrants the trade-off. Furthermore, using improved technology can enhance computational efficiency, decrease expenses while preserve the accuracy-efficiency equilibrium.

**Table 5:** Analysis of several machines and deep learning algorithms for multi-station air quality datasets.

Multi-station	Models	Prediction of PM2.5				Computational Cost for each station (seconds)
		MSE	MAE	RMSE	R^2^	
**Ashok Vihar**	SVM	1.4401	1.5523	1.3556	0.6132	11.21
	RF	0.9900	1.3669	1.2932	0.6589	13.44
	RNN	0.8711	1.2431	1.0234	0.7266	14.58
	LSTM	0.6543	0.6232	0.9001	0.7542	15.16
	GRU	0.5462	0.5123	0.7698	0.8023	16.67
	BI-LSTM	0.4323	0.3900	0.5211	0.8152	26.67
	BI-LSTM ATTEN	0.2135	0.2451	0.4865	0.8563	22.66
	AquaWave-BiLSTM	**0.0006**	**0.0154**	**0.0248**	**0.9436**	**20.98**
**DC Stadium**	SVM	1.6623	1.2575	1.4553	0.6430	11.35
	RF	0.1011	1.3896	1.3010	0.6930	14.21
	RNN	0.8932	1.2232	1.0933	0.7321	15.66
	LSTM	0.6639	0.6351	0.9351	0.7752	17.24
	GRU	0.5561	0.5439	0.7531	0.7963	17.24
	BI-LSTM	0.4210	0.3721	0.5321	0.8522	17.15
	BI-LSTM ATTEN	0.2593	0.2635	0.3951	0.8874	24.22
	AquaWave-BiLSTM	**0.0009**	**0.0194**	**0.0301**	**0.9823**	**18.43**
**Dwarka Sec**	SVM	1.2201	1.3572	1.5654	0.6652	11.63
	RF	0.9839	1.3782	1.3139	0.7052	14.99
	RNN	0.9031	1.1134	0.9938	0.7522	15.15
	LSTM	0.6235	0.6135	0.8932	0.7999	16.42
	GRU	0.5663	0.4949	0.7938	0.8172	16.61
	BI-LSTM	0.4132	0.4056	0.5031	0.8472	18.12
	BI-LSTM ATTEN	0.1931	0.1935	0.3735	0.8695	23.55
	AquaWave-BiLSTM	**0.0008**	**0.0179**	**0.0290**	**0.9396**	**19.73**
**Nehru Nagar**	SVM	1.3220	1.4278	1.6321	0.5930	11.71
	RF	0.1135	1.3569	1.2809	0.6930	13.62
	RNN	0.8631	1.0431	1.1132	0.7132	15.44
	LSTM	0.5931	0.6031	0.8357	0.7689	16.53
	GRU	0.5321	0.5036	0.7131	0.8056	99.20
	BI-LSTM	0.4059	0.4056	0.5031	0.8632	20.89
	BI-LSTM ATTEN	0.1832	0.1886	0.4195	0.8965	22.11
	AquaWave-BiLSTM	**0.0006**	**0.0179**	**0.0246**	**0.9805**	**21.38**
**Najafgarh102.58**	SVM	1.2856	1.4542	1.5963	0.6392	12.06
	RF	0.9569	1.4539	1.2981	0.7096	13.67
	RNN	0.8690	1.1431	0.9834	0.6952	14.74
	LSTM	0.6011	0.5949	0.8357	0.7711	16.09
	GRU	0.5031	0.4835	0.6905	0.8123	16.62
	BI-LSTM	0.4639	0.4482	0.4821	0.8574	20.93
	BI-LSTM ATTEN	0.2093	0.2038	0.3525	0.8652	25.25
	AquaWave-BiLSTM	**0.0004**	**0.0122**	**0.0189**	**0.8911**	**22.38**
**Okhla**	SVM	1.1616	1.2528	1.3838	0.6230	12.52
	RF	0.1121	1.4463	1.2893	0.6892	14.34
	RNN	0.8832	1.2158	1.0882	0.7015	15.05
	LSTM	0.6011	0.5949	0.8357	0.7852	15.20
	GRU	0.5132	0.5235	0.7452	0.8078	15.98
	BI-LSTM	0.4432	0.4221	0.5584	0.9001	18.10
	BI-LSTM ATTEN	0.2384	0.2246	04352	0.9125	23.59
	AquaWave-BiLSTM	**0.0007**	**0.0166**	**0.0240**	**0.9657**	**20.15**

### 5.4 Feature importance analysis using SHAP

**Fig 15:**
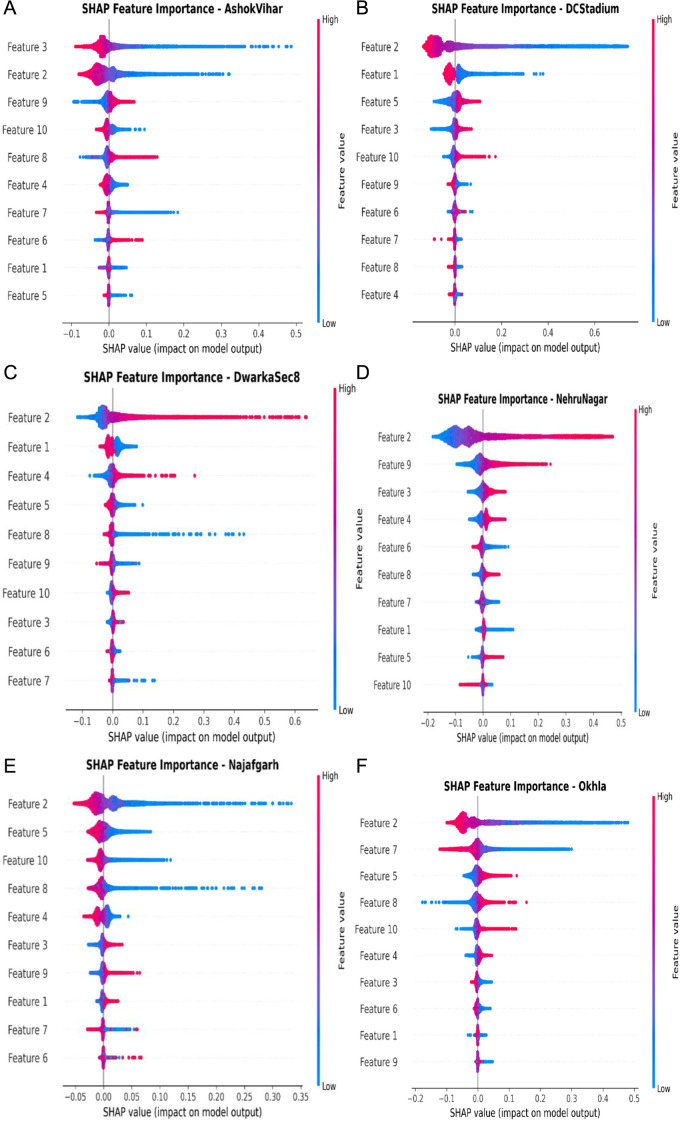
SHAP summary plots of the top 10 features contributing to PM2.5 prediction at each station: (a) AshokVihar, (b) DCStadium, (c) DwarkaSec8, (d) Najafgarh, (e) NehruNagar, and (f)Okhla.

To improve the interpretability of the AquaWave-BiLSTM framework and to ascertain the impact of meteorological and pollutant factors on PM2.5 prediction, this study performed a feature significance analysis utilizing Shapley Additive Explanations (SHAP) [53] SHAP values were calculated for each monitoring station using a Random Forest surrogate model trained on the chosen feature set [54]. The SHAP summary charts in Fig 15 depict the ranking contributions of the ten most significant characteristics at each station. Wavelet and PCA are used to modify and compress the original features into significant components, from which the most pertinent ones are discerned utilizing AOAOA. The chosen components are utilized to train the predictive model, while SHAP is applied to elucidate and measure their influence on the model’s output. This research correlated the SHAP features with the original meteorological and pollutant data by associating each SHAP-ranked feature with its own PCA component and determining the top three contributing wavelet-transformed variables for that component. Table 6 delineates, for each station, the highest-ranked SHAP feature, its corresponding PCA component, and the most significant original variables. The SHAP-based feature significance analysis and associated visualizations for model interpretability are provided in S3 File in S1 Data.

**Table 6:** Top-ranked SHAP feature (Rank 1) at each station, its PCA component, and the three most significant meteorological and pollutant factors influencing PM2.5 prediction. PM₁₀ emerged as the primary predictor across stations, with RH, AT, AP, and WS also making substantial contributions.

Station	SHAP Rank	SHAP Features	PCA Component	Top Variables
AshoVihar	1	Feature 3	Component 3	PM10, AT, NOx
DCStadium	1	Feature 2	Component 2	PM10, RH, NO2
DwarkaSec8	1	Feature 2	Component 2	PM10, WS, CO
Najafgarh	1	Feature 2	Component 2	PM10, AP, NO
NehruNagar	1	Feature 2	Component 2	PM10, RH, SO2
Okhla	1	Feature 2	Component 2	PM10, AT, NO2

PM₁₀ consistently emerged as the primary predictor across stations, significantly influencing Ashok Vihar, Najafgarh, and Okhla. Meteorological factors like RH, AT, AP, and WS significantly influenced the forecasts. At the Okhla station, SHAP Feature 2 (PCA Component 2) was predominantly affected by PM10, relative humidity (RH), and nitrogen dioxide (NO₂). At Ashok Vihar, SHAP Feature 3 (PCA Component 3) was mostly influenced by PM10, AT, and NOₓ. This investigation underscores the intricate relationship between particles and meteorological elements in influencing PM2.5 concentrations.

### 5.5 Statistical assessment of feature extraction and selection techniques utilizing the wilcoxon signed-rank test for multi-station analysis

This study utilized the Wilcoxon Signed-Rank Test to evaluate the efficacy of several feature extraction and selection methodologies across six air quality monitoring stations: Ashok Vihar, DC Stadium, Dwarka Sec 8, Nehru Nagar, Najafgarh, and Okhla. The main aim was to assess the efficacy of a hybrid feature extraction method that combines WT and PCA against two feature selection techniques: AOAOA and Random Selection. The Wilcoxon Signed-Rank Test, a non-parametric technique suitable for paired, non-normally distributed data like air quality measurements, was employed to assess the statistical significance of variations in predictive performance. The Shapiro–Wilk test validated the non-normality of the MSE differences (p < 0.05), hence validating the use of this non-parametric test. At all six monitoring sites, the suggested WT + PCA technique consistently attained the lowest MSE values in comparison to AOAOA and Random Selection. In comparison of WT + PCA with AOAOA, the Wilcoxon test produced test statistics of 0.0 and a p-value of 0.0312, signifying a statistically significant enhancement at the 0.05 threshold. The comparison between WT + PCA and Random Selection yielded a test statistic of 0.0 and a p-value of 0.0156, further substantiating the superiority of the suggested strategy. A test value of 0.0 indicates that the suggested strategy surpassed all alternatives in every paired comparison.

To further delineate the variability and robustness of the techniques, in this study calculated the mean ± standard deviation (SD) and 95% confidence intervals (CI) of the mean squared error (MSE) across all stations. The WT + PCA method attained an average MSE of 0.00067 ± 0.00017 (95% CI: [0.00050, 0.00084]), indicating exceptional accuracy and dependability. The AOAOA strategy produced a greater and more varied MSE of 0.0044 ± 0.0031 (95% CI: [0.0012, 0.0076]), but the Random Selection method exhibited the poorest performance, with an MSE of 0.0069 ± 0.0037 (95% CI: [0.0031, 0.0107]). These findings highlight the exceptional and reliable efficacy of the proposed hybrid feature extraction approach, demonstrated by its reduced mean error, more concentrated distribution, and smaller confidence interval relative to the alternatives Table 7.

**Table 7:** Station-wise MSE comparison across proposed and baseline methods.

Multi-Station	Proposed (Aquvawave + BiLSTM)	AOAOA	Random
Ashok Vihar	0.0006	0.0071	0.0095
DC Stadium	0.0009	0.0017	0.0032
Dwarka Sec	0.0008	0.0011	0.0038
Nehru Nager	0.0006	0.0040	0.0056
Najafgarh	0.0004	0.0032	0.0061
Okhla	0.0007	0.0093	0.0134

**Fig 16:**
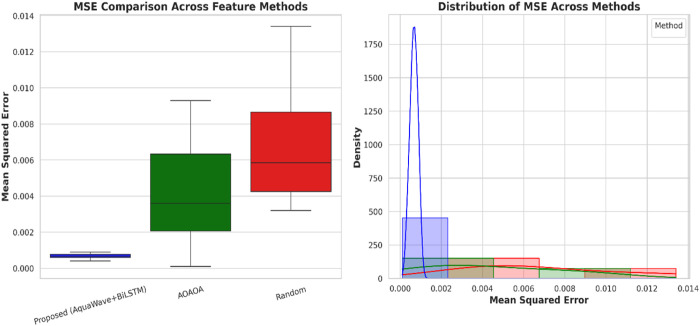
Comparison of average mean squared error (MSE) across feature extraction and selection methods for multiple stations.

Fig 16 depicts the MSE effectiveness of three feature processing algorithms utilizing boxplots and KDE-overlaid histograms. The AquaWave+BiLSTM approach (Wavelet + PCA + AOAOA) demonstrates the lowest and most consistent MSE, seen by its narrow distribution and low median in the boxplot. The KDE figure further validates the robustness of this method, with MSE values closely clustered around zero. Conversely, AOAOA-only and Random selection demonstrate more error variability. These visualizations confirm the efficacy of integrating Wavelet Transform, PCA, and AOAOA in enhancing precision in predictions for air quality forecasting.

## 6. Discussion

The suggested AOAOA–Bi-LSTM model shows remarkable accuracy in air quality forecasting by using critical meteorological variables, such as PM10, WS, WD, RH, NO₂, AT, NH₃, and AP. The variables were chosen via a sophisticated feature selection technique to preserve the most pertinent properties. The incorporation of wavelet transforms (WT) augmented the prediction framework by splitting time-series air quality data into separate frequency components, maintaining both temporal and spectral attributes. Principal component analysis (PCA) indicated that the first PC1 represented 20–32% of the variance among monitoring stations, whereas the second principal component (PC2) included 12–18%, underscoring prevailing trends in air quality fluctuations. This work rigorously investigated the impact of feature extraction and selection on model performance, contrasting predictive effectiveness with and without these methodologies. AOAOA was evaluated against alternative optimization techniques, exhibiting enhanced feature selection efficacy while minimizing computing complexity. The Bi-LSTM model, utilizing sophisticated gated mechanisms, successfully alleviated gradient vanishing problems and maintained long-term dependencies, surpassing traditional ML and DL models in multi-station air quality forecasting. By tackling prevalent issues like overfitting, AOAOA successfully removed superfluous and less useful characteristics, hence improving generalization and maintaining consistent performance. The comparative examination with alternative prediction models further confirmed the robustness of the AOAOA–Bi–LSTM strategy. Despite the AquaWave-BiLSTM model’s modest computing demands compared to baseline models, its superior predictive performance warrants the trade-off. The SHAP-based research validated the significant influence of PM₁₀ and essential meteorological variables (RH, AT, AP, WS) in forecasting PM2.5 at all sites. This corresponds with prior research indicating that particle precursors and climatic factors substantially affect PM2.5 dynamics. Station-specific variations were observed: RH and NO₂ had a greater influence at Okhla, AT and NOₓ at Ashok Vihar, and WS and CO at DC Stadium. The results substantiate the prediction capability of the AquaWave-BiLSTM framework and provide practical insights for addressing certain contaminants and climatic variables throughout Delhi. The Wilcoxon Signed-Rank Test was utilized to statistically evaluate feature extraction and selection methods across several monitoring stations. The results proved a statistically significant enhancement with the suggested Wavelet+PCA method compared to AOAOA-based selection (p = 0.0312), underscoring the advantages of systematic feature extraction. The technique demonstrated enhanced stability and reduced MSE variability among stations, hence affirming its resilience for air quality forecasting. These findings underscore the significance of integrating Wavelet+PCA with AOAOA to formulate an optimum feature subset, highlighting a hybrid methodology for enhanced prediction accuracy and generalizability.

### 6.1 Limitations and future work

The AquaWave-BiLSTM model exhibits robust forecasting capabilities, but certain limitations persist. This study utilized data from six monitoring stations in Delhi over a 16-month duration, capturing short-term seasonal fluctuations while neglecting long-term trends and interannual variability. Future studies may employ multi-year, multi-city datasets to improve generalizability and assess effectiveness across various meteorological and pollution conditions. Moreover, the assessment utilized a basic 80:20 chronological division, which may inadequately reflect temporal connections within the data. Future studies might investigate advanced validation procedures, including rolling window and walk-forward methodologies. Ultimately, integrating supplementary pollution sources and augmenting.

## 7. Conclusion

This research presents the AquaWave-BiLSTM framework for multi-station air quality prediction in Delhi, utilizing sophisticated feature extraction, selection, and deep learning methodologies. The model adeptly captures complex temporal and frequency patterns while minimizing dimensionality through the integration of Wavelet Transform and Principal Component Analysis (PCA). Exploration and exploitation are balanced in the hybrid Aquila–Arithmetic Optimization Algorithm (AOAOA), which improves feature selection. The Bidirectional Long Short-Term Memory (Bi-LSTM) network effectively captures temporal relationships, resulting in enhanced predictive performance relative to conventional machine learning and deep learning models. The AquaWave-BiLSTM attained significant predictive accuracy (MSE: 0.00065, MAE: 0.04566, RMSE: 0.02523, R²: 0.9494) while exhibiting notable computing economy, underscoring its practical use for real-time urban air quality forecasting. The Wilcoxon Signed-Rank Test statistically validated the efficacy of the suggested feature extraction and selection methodology. The use of SHAP analysis enhanced interpretability, elucidating the relative significance of contaminants and climatic variables affecting PM2.5 forecasts.

## Supporting information

S1 Data S1 File. Colab notebook demonstrating the data loading process, feature normalization techniques, and density distribution plots for each monitoring station. S2 File. Colab notebook for AquaWave-BiLSTM model analysis, and results. S3 File. Colab notebook containing SHAP visualizations and interpretability analysis related to PM2.5 prediction.
(ZIP)
