# Supplementary material for: Efficient multi-station air quality prediction in Delhi with wavelet and optimization-based models
Source: PLoS One. 2025 Aug 19;20(8):e0330465. doi: 10.1371/journal.pone.0330465 (PMC12364328; doi:10.1371/journal.pone.0330465)
Supplement: S1 Data — S2 File. Colab notebook for AquaWave-BiLSTM model analysis, and results. S3 File. Colab notebook containing SHAP visualizations and interpretability analysis related to PM2.5 prediction. (ZIP) [file pone.0330465.s001.zip › S1-File .pdf]

DATASET LOADING

```
import pandas as pd

stations = {
    'AshokVihar': '/content/AshokVihar_Hourly.csv',
    'DCStadium': '/content/DCStadium_Hourly.csv',
    'DwarkaSec8': '/content/DwarkaSec8_Hourly.csv',
    'NehruNagar': '/content/NehruNagar_Hourly.csv',
    'Najafgarh': '/content/Najafgarh_Hourly.csv',
    'Okhla': '/content/Okhla_Hourly.csv'
}

for station, file_path in stations.items():
    df = pd.read_csv(file_path)
    print(f"{station}: {df.shape[0]} rows, {df.shape[1]} columns")
```

```
AshokVihar: 11705 rows, 21 columns
DCStadium: 11704 rows, 21 columns
DwarkaSec8: 11704 rows, 21 columns
NehruNagar: 11705 rows, 21 columns
Najafgarh: 11704 rows, 21 columns
Okhla: 11705 rows, 21 columns
```

DATA PREPROCESSING

```
import pandas as pd
import numpy as np
import matplotlib.pyplot as plt
import seaborn as sns
from sklearn.preprocessing import MinMaxScaler
import os

# Define file paths
file_paths = [
    '/content/AshokVihar_Hourly.csv',
    '/content/DCStadium_Hourly.csv',
    '/content/DwarkaSec8_Hourly.csv',
    '/content/Najafgarh_Hourly.csv',
    '/content/NehruNagar_Hourly.csv',
    '/content/Okhla_Hourly.csv'
]

# List to hold dataframes
dfs = []

# Load each file into a DataFrame and append to the [illegible]
for file_path in file_paths:
    df = pd.read_csv(file_path)
    df['source_file'] = os.path.basename(file_path)  # Add a column to identify the source file
    dfs.append(df)
```

What can I help you build?

```
# Concatenate all DataFrames into a single DataFrame
combined_df = pd.concat(dfs, ignore_index=True)

# Display basic information about the dataset
print(combined_df.info())

# Define features and target for normalization
features = combined_df.drop(columns=['PM2.5', 'PM10', 'Unnamed: 0', 'source_file'])
target = combined_df[['PM2.5', 'PM10']]

# Normalize the data
scaler_X = MinMaxScaler()
scaler_y = MinMaxScaler()

normalized_features = scaler_X.fit_transform(features)
normalized_target = scaler_y.fit_transform(target)

# Convert back to DataFrame for plotting
normalized_features_df = pd.DataFrame(normalized_features, columns=features.columns)
normalized_target_df = pd.DataFrame(normalized_target, columns=['PM2.5', 'PM10'])

# Combine normalized features and target
normalized_combined_df = pd.concat([normalized_features_df, normalized_target_df], axis=1)

# Histograms for each feature and target
normalized_combined_df.hist(bins=50, figsize=(20, 15))
plt.suptitle('Histograms of Normalized Features and Targets')
plt.show()
```

```
<class 'pandas.core.frame.DataFrame'>
RangeIndex: 70227 entries, 0 to 70226
Data columns (total 22 columns):
 #   Column       Non-Null Count  Dtype  
---  ------       --------------  -----  
 0   Unnamed: 0   70227 non-null  int64  
 1   PM2.5        70227 non-null  float64
 2   year         70227 non-null  float64
 3   month        70227 non-null  float64
 4   day          70227 non-null  float64
 5   hour         70227 non-null  float64
 6   PM10         70227 non-null  float64
 7   AT           70227 non-null  float64
 8   BP           70227 non-null  float64
 9   SR           70227 non-null  float64
 10  RH           70227 non-null  float64
 11  WS           70227 non-null  float64
 12  WD           70227 non-null  float64
 13  NO           70227 non-null  float64
 14  NO2          70227 non-null  float64
 15  SO2          70227 non-null  float64
 16  Ozone        70227 non-null  float64
 17  CO           70227 non-null  float64
 18  Benzene      70227 non-null  float64
 19  NH3          70227 non-null  float64
 20  NOx          70227 non-null  float64
 21  source_file  70227 non-null  object 
dtypes: float64(20), int64(1), object(1)
memory usage: 11.8+ MB
None
```

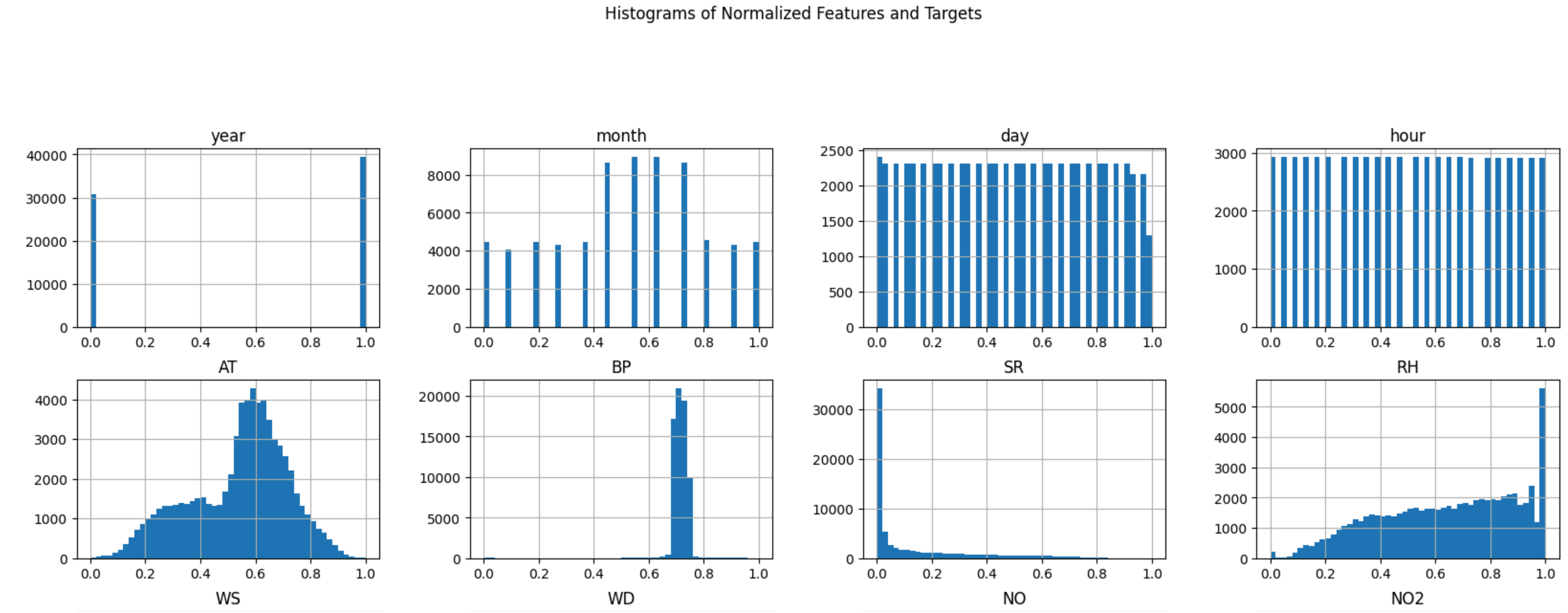

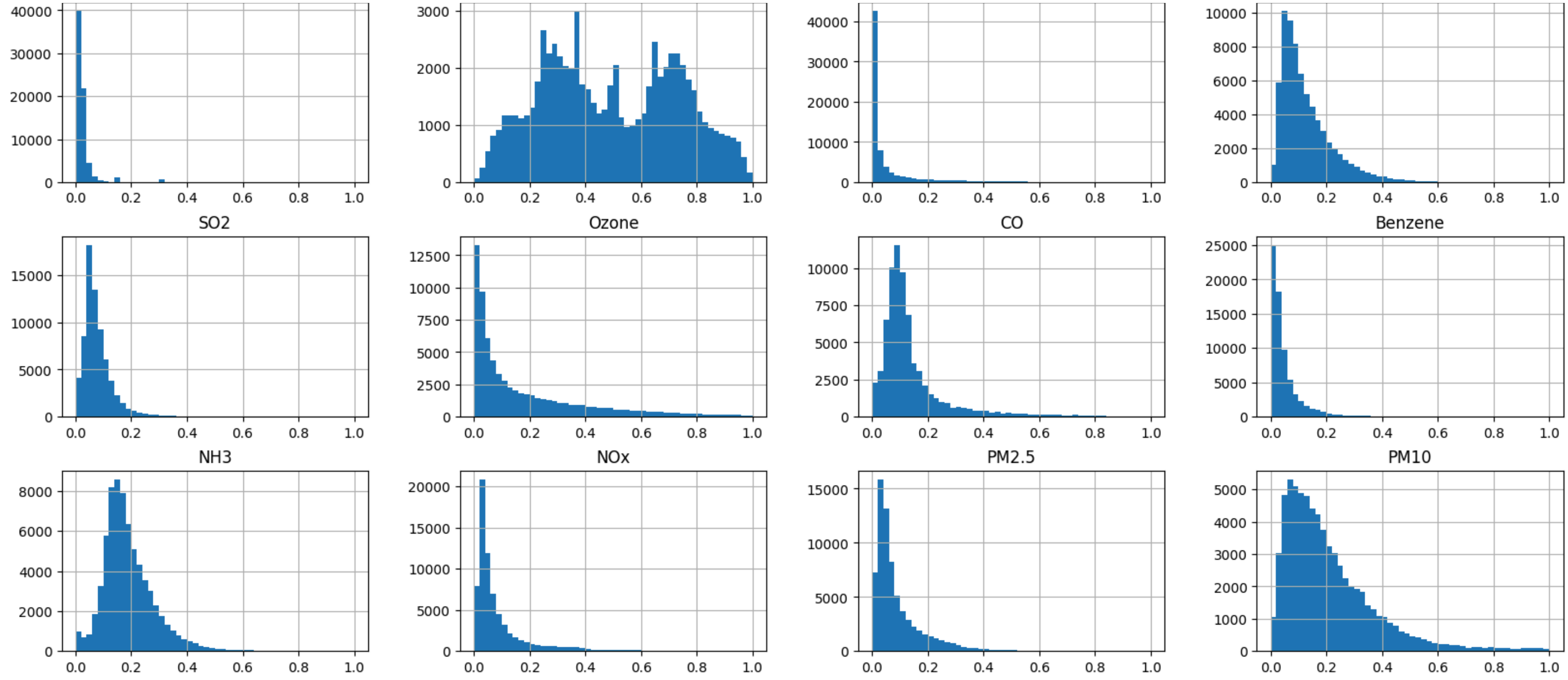
SO2
Ozone
CO
Benzene
NH3
NOx
PM2.5
PM10

```
import pandas as pd
import matplotlib.pyplot as plt
import seaborn as sns
import os

# Set seaborn style
sns.set_style("whitegrid")

# Define file paths for your six stations
station_files = {
    'AshokVihar': '/content/AshokVihar_Hourly.csv',
    'DCStadium': '/content/DCStadium_Hourly.csv',
    'DwarkaSec8': '/content/DwarkaSec8_Hourly.csv',
    'NehruNagar': '/content/NehruNagar_Hourly.csv',
    'Najafgarh': '/content/Najafgarh_Hourly.csv',
    'Okhla': '/content/Okhla_Hourly.csv'
}

# Read and concatenate all stations into one DataFrame
dfs = []
for station, path in station_files.items():
    if os.path.exists(path):
        df = pd.read_csv(path)
        if 'PM2.5' not in df.columns:
            print(f"⚠️ PM2.5 column not found in {station}, available columns: {df.columns.tolist()}")
            continue
        df['source_file'] = station  # add station name column
        dfs.append(df)
    else:
        print(f"❌ File not found: {path}")

# Concatenate
combined_df = pd.concat(dfs, ignore_index=True)

print(f"✅ Combined data shape: {combined_df.shape}")
print(f"✅ Columns: {combined_df.columns.tolist()}")

# Unique stations
stations = combined_df['source_file'].unique()

# Loop and plot
for station in stations:
    df_station = combined_df[combined_df['source_file'] == station]

    # Drop missing or constant values
    df_station = df_station.dropna(subset=['PM2.5'])
    if df_station['PM2.5'].nunique() <= 1:
        print(f"⚠️ Skipping {station}: PM2.5 has no variation.")
        continue

    plt.figure(figsize=(8, 5), dpi=300)
    sns.kdeplot(
        data=df_station,
        x='PM2.5',
```

```
        fill=True,
        alpha=0.6,
        color='darkblue'
    )

    plt.xlabel('PM2.5 Concentration', fontsize=12, fontweight='bold')
    plt.ylabel('Density', fontsize=12, fontweight='bold')
    plt.title(f'Density Plot of PM2.5 for {station}', fontsize=14, fontweight='bold')
    plt.xticks(fontsize=10, fontweight='bold')
    plt.yticks(fontsize=10, fontweight='bold')
    plt.tight_layout()
    plt.show()
```

```
⚠ PM2.5 column not found in Najafgarh, available columns: ['51.0', '32.45', '975.42', '3.17', '22.35', '0.95', '257.95', '25.2', '55.83', '22.62', '72.22', '0.38', '0.88',
✅ Combined data shape: (58523, 22)
✅ Columns: ['Unnamed: 0', 'PM2.5', 'year', 'month', 'day', 'hour', 'PM10', 'AT', 'BP', 'SR', 'RH', 'WS', 'WD', 'NO', 'NO2', 'SO2', 'Ozone', 'CO', 'Benzene', 'NH3', 'NOx',
```

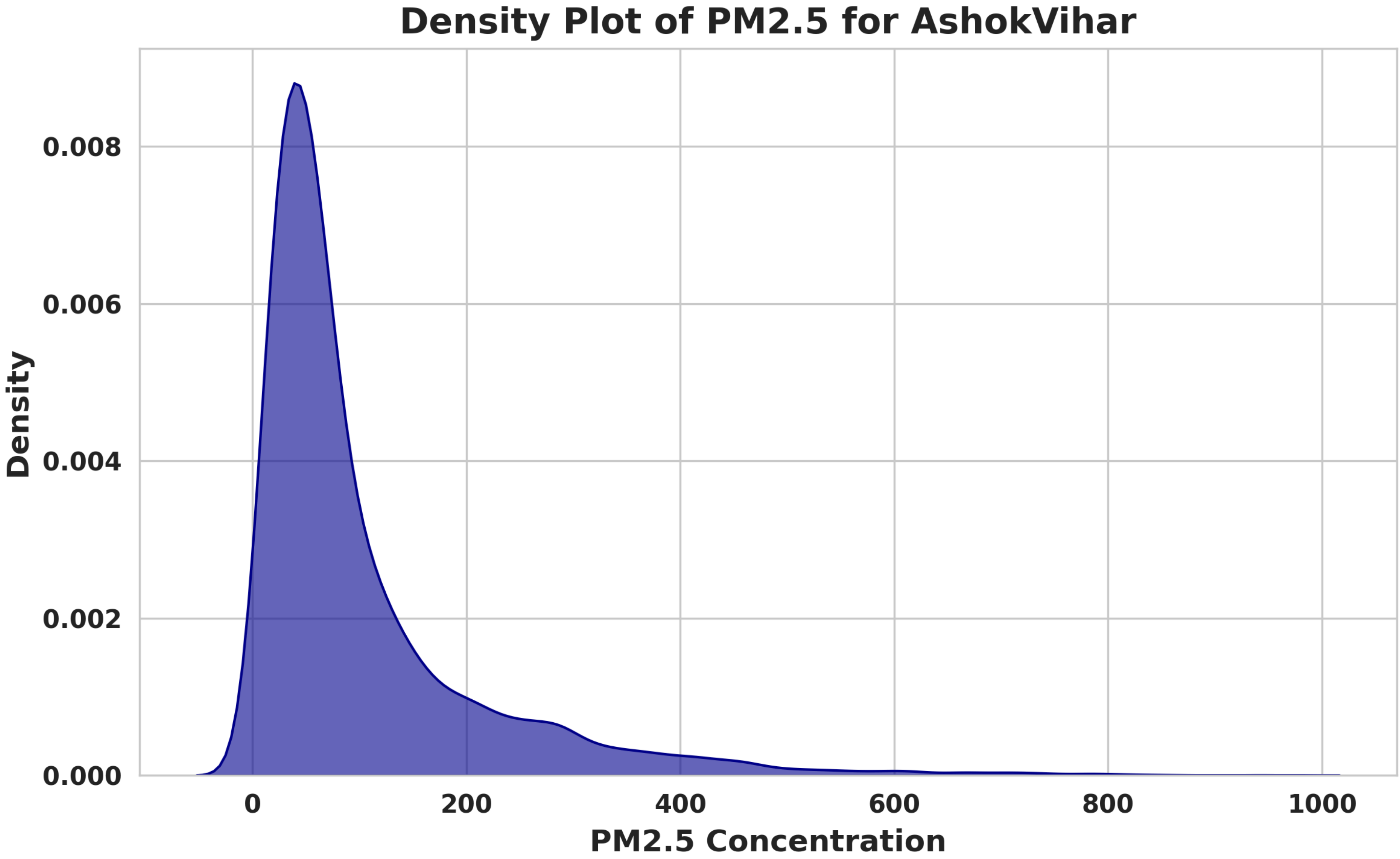

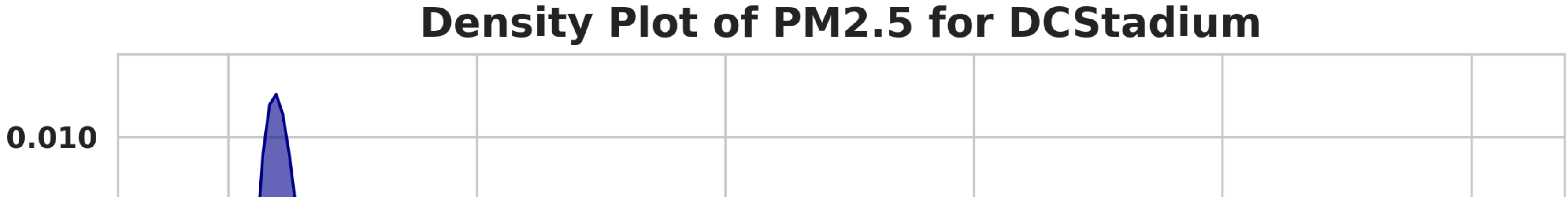

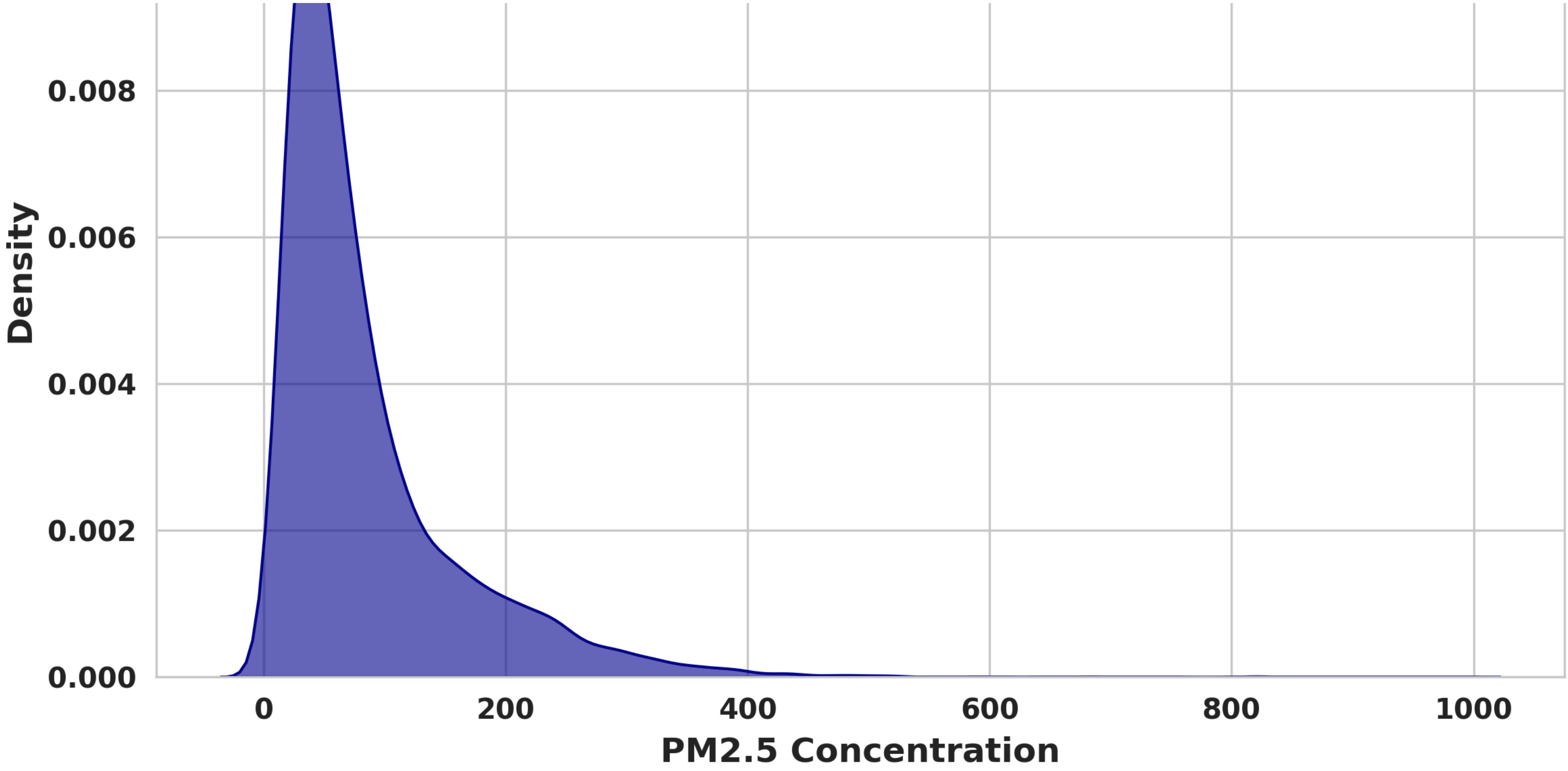
0.008
0.006
0.004
0.002
0.000
Density
0
200
400
600
800
1000
PM2.5 Concentration

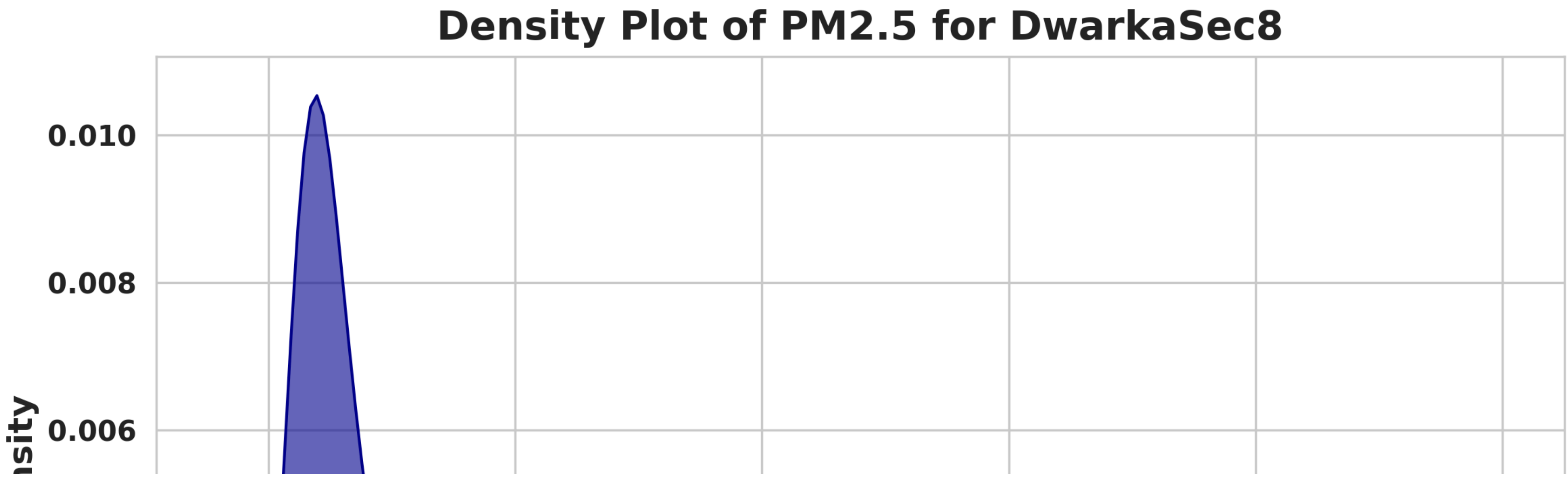
Density Plot of PM2.5 for DwarkaSec8
0.010
0.008
0.006

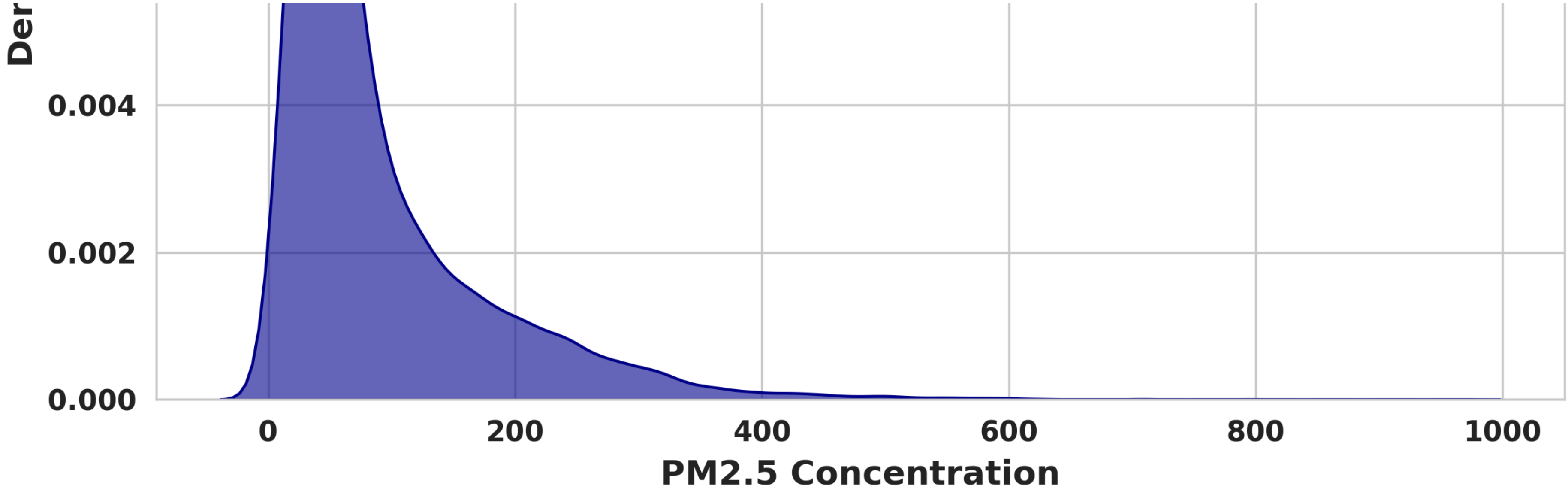
Der
0.004
0.002
0.000
0
200
400
600
800
1000
PM2.5 Concentration

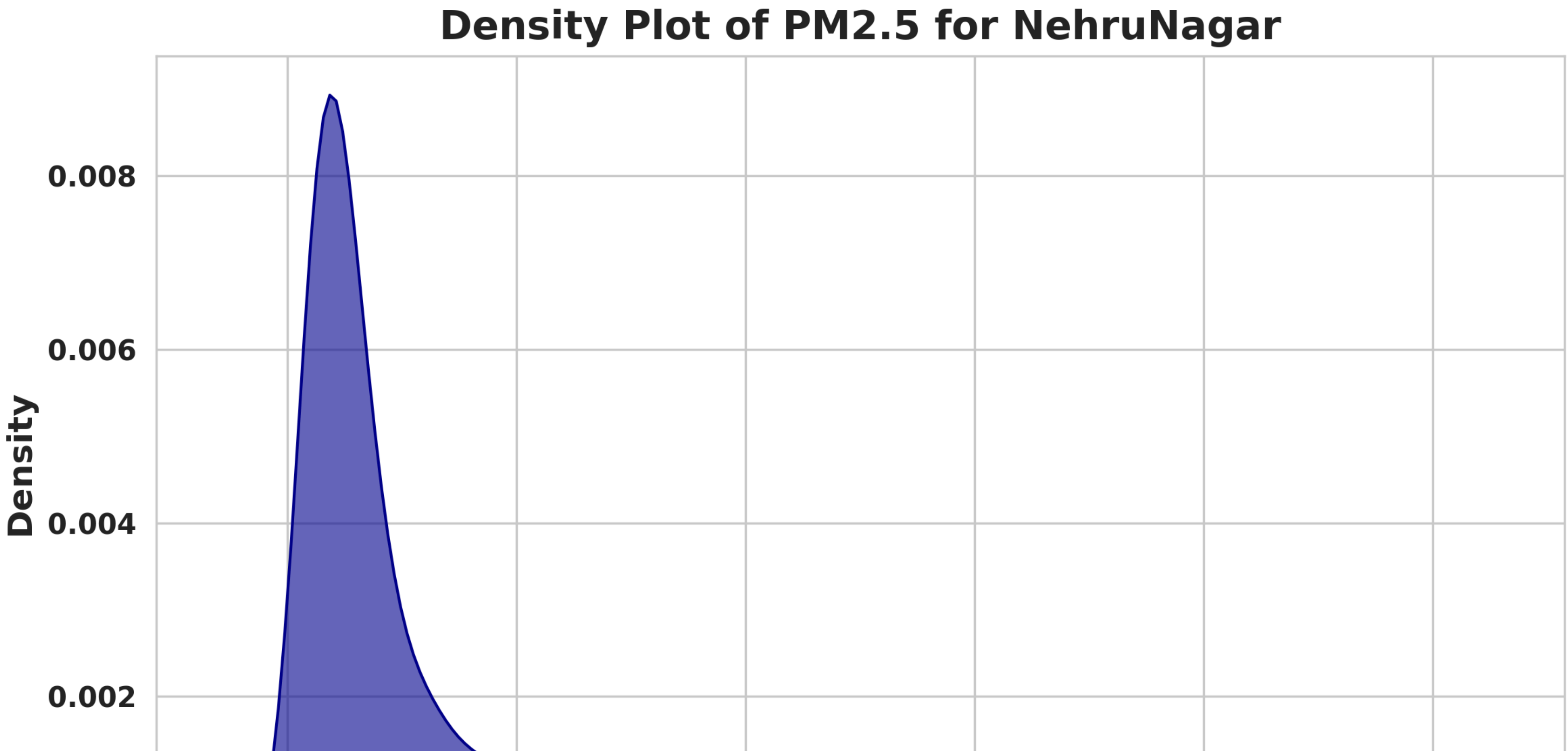
Density Plot of PM2.5 for NehruNagar
0.008
0.006
0.004
0.002
Density

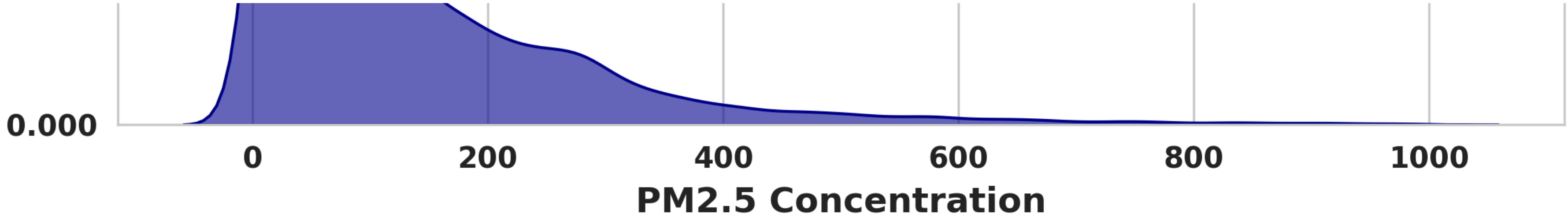
0.000
0
200
400
600
800
1000
PM2.5 Concentration

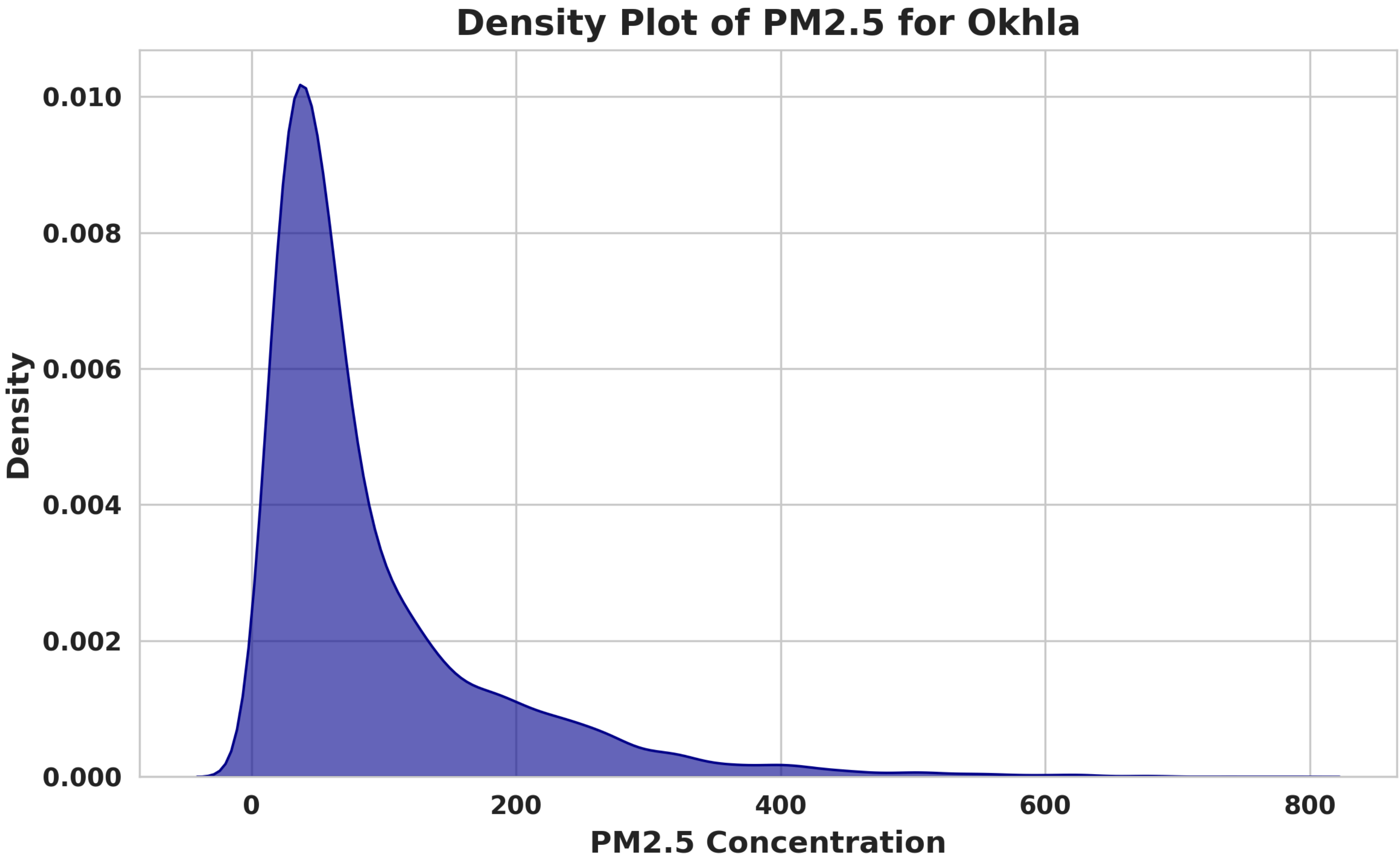
Density Plot of PM2.5 for Okhla
Density
0.010
0.008
0.006
0.004
0.002
0.000
0
200
400
600
800
PM2.5 Concentration

```
import pandas as pd
import numpy as np
import seaborn as sns
import matplotlib.pyplot as plt

stations = {
    'AshokVihar': '/content/AshokVihar_Hourly.csv',
    'DCStadium': '/content/DCStadium_Hourly.csv',
    'DwarkaSec8': '/content/DwarkaSec8_Hourly.csv',
    'NehruNagar': '/content/NehruNagar_Hourly.csv',
    'Najafgarh': '/content/Najafgarh_Hourly.csv',
    'Okhla': '/content/Okhla_Hourly.csv'
}

all_corrs = []

for station, path in stations.items():
    df = pd.read_csv(path)
    # Optional: drop non-numeric columns, adjust as needed
    df_numeric = df.select_dtypes(include=[np.number])
    corr = df_numeric.corr(method='spearman')['PM2.5'].rename(station)
    all_corrs.append(corr)

# Combine all stations into a single DataFrame
corr_df = pd.concat(all_corrs, axis=1)

# Calculate mean correlation for title
mean_corr = corr_df.mean().mean()

# Plotting
plt.figure(figsize=(12, 8))
sns.set(font_scale=1.1)
ax = sns.heatmap(
    corr_df,
    annot=True,
    fmt=".2f",
    cmap='Greens',
    cbar_kws={'label': 'Spearman Correlation'},
    linewidths=0.5
)
```
