# Supplementary material for: Efficient multi-station air quality prediction in Delhi with wavelet and optimization-based models
Source: PLoS One. 2025 Aug 19;20(8):e0330465. doi: 10.1371/journal.pone.0330465 (PMC12364328; doi:10.1371/journal.pone.0330465)
Supplement: S1 Data — S2 File. Colab notebook for AquaWave-BiLSTM model analysis, and results. S3 File. Colab notebook containing SHAP visualizations and interpretability analysis related to PM2.5 prediction. (ZIP) [file pone.0330465.s001.zip › S2-File.pdf]

```
!pip install PyWavelets
```

```
Collecting PyWavelets
  Downloading pywavelets-1.8.0-cp311-cp311-manylinux_2_17_x86_64.manylinux2014_x86_64.whl.metadata (9.0 kB)
Requirement already satisfied: numpy<3,>=1.23 in /usr/local/lib/python3.11/dist-packages (from PyWavelets) (2.0.2)
Downloading pywavelets-1.8.0-cp311-cp311-manylinux_2_17_x86_64.manylinux2014_x86_64.whl (4.5 MB)
   ━━━━━━━━━━━━━━━━━━━━━━━━━━━━━━━━━━━━━━━━ 4.5/4.5 MB 35.2 MB/s eta 0:00:00
Installing collected packages: PyWavelets
Successfully installed PyWavelets-1.8.0
```

Feature Extraction and Dimensionality Reduction Using Wavelet Transform and PCA

```
import numpy as np
import pandas as pd
import matplotlib.pyplot as plt
import pywt
from sklearn.preprocessing import MinMaxScaler

# ============================
# Load Data for Multiple Stations
# ============================
file_paths = {
    'AshokVihar': '/content/AshokVihar_Hourly.csv',
    'DCStadium': '/content/DCStadium_Hourly.csv',
    'DwarkaSec8': '/content/DwarkaSec8_Hourly.csv',
    'NehruNagar': '/content/NehruNagar_Hourly.csv',
    'Najafgarh': '/content/Najafgarh_Hourly.csv',
    'Okhla': '/content/Okhla_Hourly.csv'
}

wavelet_features = {}  # Store wavelet-transformed data

# ============================
# Wavelet Transform Function (Fixed)
# ============================
def apply_wavelet_transform(X, wavelet='db4', num_features=50):
    features = []
    for sample in X:
        max_level = pywt.dwt_max_level(len(sample), wavelet)  # Auto-adjust level
        level = min(3, max_level)  # Avoid boundary effects
        coeffs = pywt.wavedec(sample, wavelet, level=level)
        flattened_coeffs = np.concatenate([c.flatten() for c in coeffs])
```

```
        features.append(flattened_coeffs[:num_features])  # Select only first 'num_features'
    return np.array(features)

# ============================
# Process Each Station
# ============================
for station, path in file_paths.items():
    df = pd.read_csv(path)

    # Normalize Data
    scaler = MinMaxScaler()
    X = scaler.fit_transform(df.iloc[:, :-1].values)  # Features

    # Apply Wavelet Transform
    X_wavelet = apply_wavelet_transform(X)
    wavelet_features[station] = X_wavelet

# ============================
# Visualization - Wavelet Transform for Each Station
# ============================
fig, axes = plt.subplots(len(file_paths), 1, figsize=(12, len(file_paths) * 3))

for i, (station, X_wavelet) in enumerate(wavelet_features.items()):
    ax = axes[i]  # Assign subplot
    ax.plot(X[0], label="Original Feature", color='black')
    ax.plot(X_wavelet[0], label="Wavelet-Transformed", linestyle='dashed', color='blue')
    ax.set_title(f'{station} - Wavelet Transform')
    ax.set_xlabel('Time Steps')
    ax.set_ylabel('Feature Values')
    ax.legend()
    ax.grid(True)

plt.tight_layout()
plt.show()
```

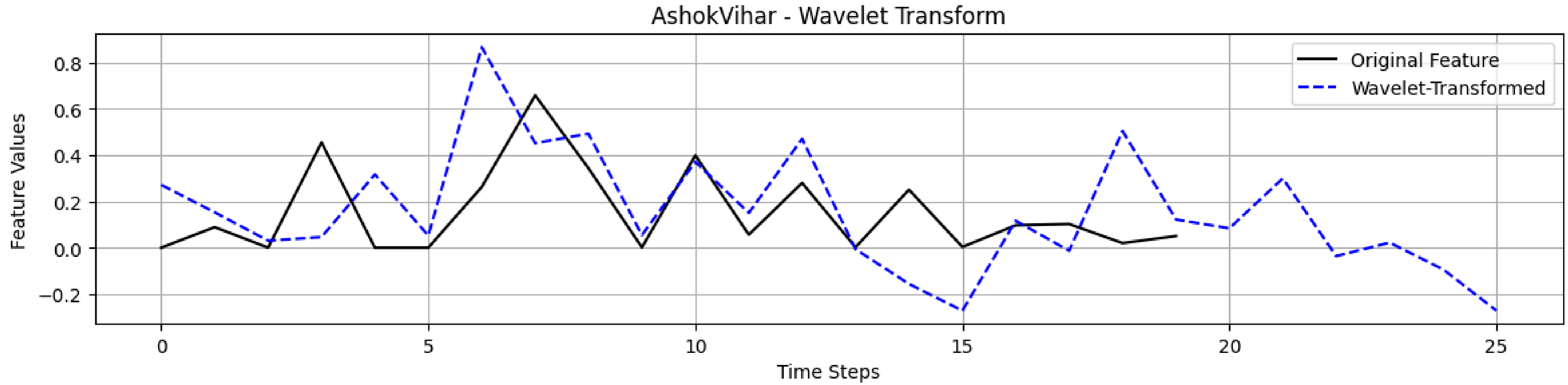
AshokVihar - Wavelet Transform
Feature Values
0.8
0.6
0.4
0.2
0.0
−0.2
0
5
10
15
20
25
Time Steps
Original Feature
Wavelet-Transformed

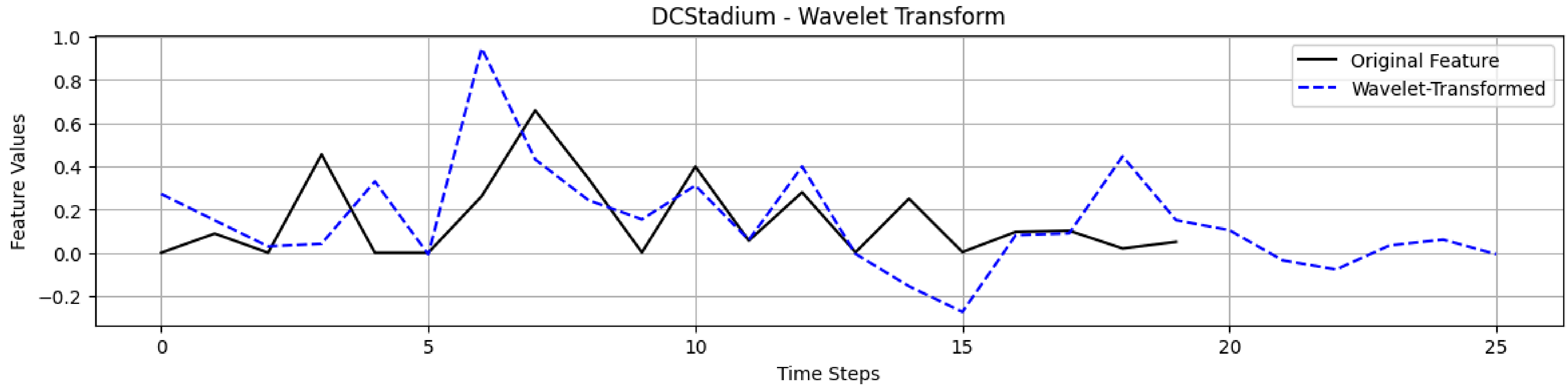
DCStadium - Wavelet Transform
Feature Values
1.0
0.8
0.6
0.4
0.2
0.0
−0.2
0
5
10
15
20
25
Time Steps
Original Feature
Wavelet-Transformed

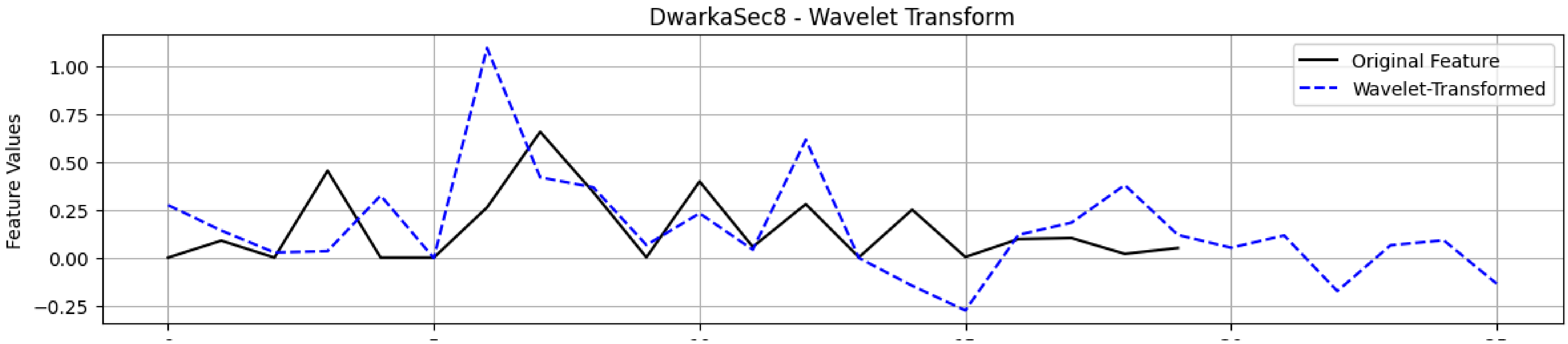
DwarkaSec8 - Wavelet Transform
Feature Values
1.00
0.75
0.50
0.25
0.00
−0.25
Original Feature
Wavelet-Transformed

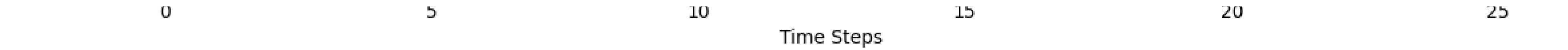
0
5
10
15
20
25
Time Steps

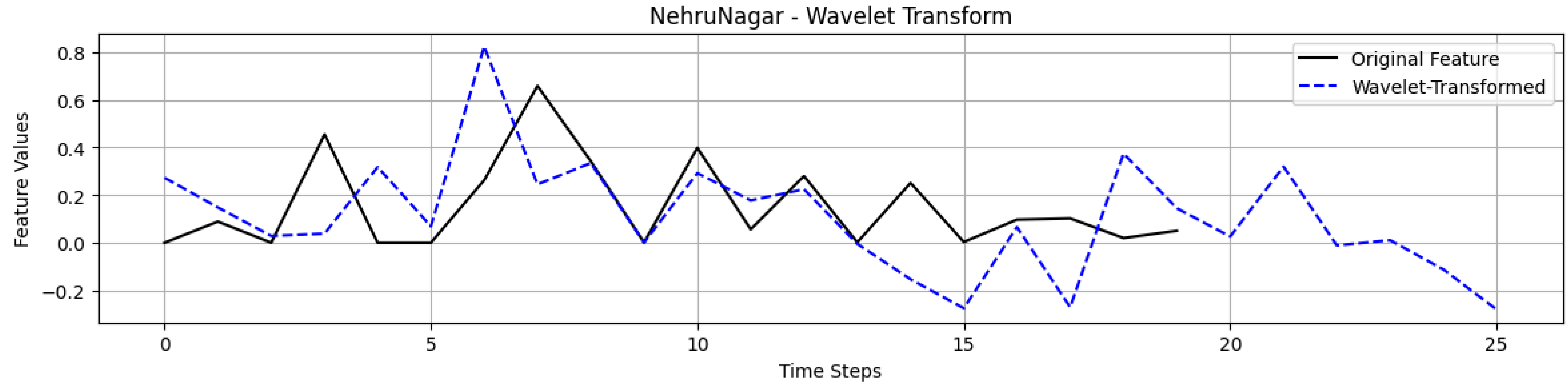
NehruNagar - Wavelet Transform
Original Feature
Wavelet-Transformed
Feature Values
0.8
0.6
0.4
0.2
0.0
−0.2
0
5
10
15
20
25
Time Steps

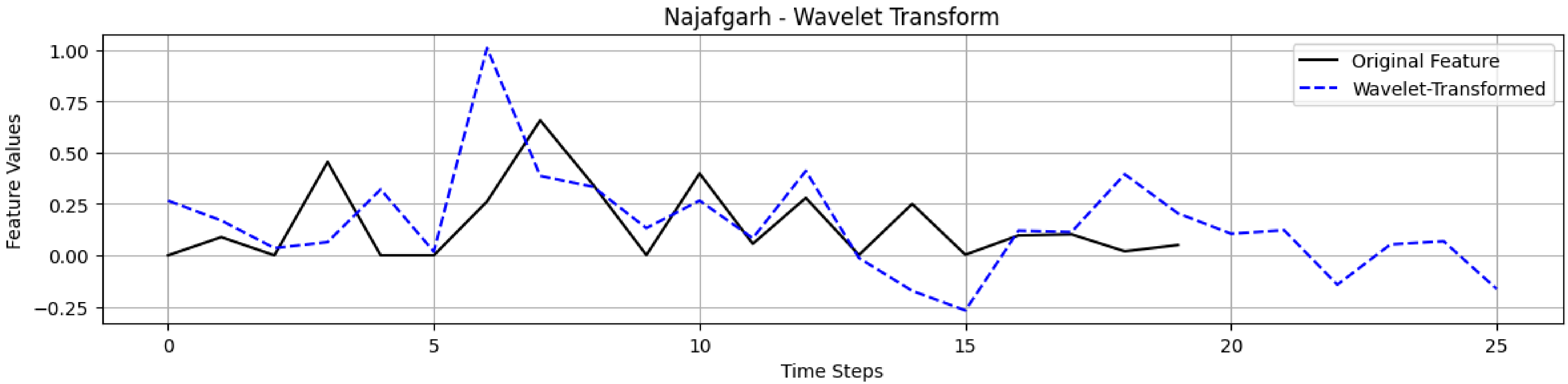
Najafgarh - Wavelet Transform
Original Feature
Wavelet-Transformed
Feature Values
1.00
0.75
0.50
0.25
0.00
−0.25
0
5
10
15
20
25
Time Steps

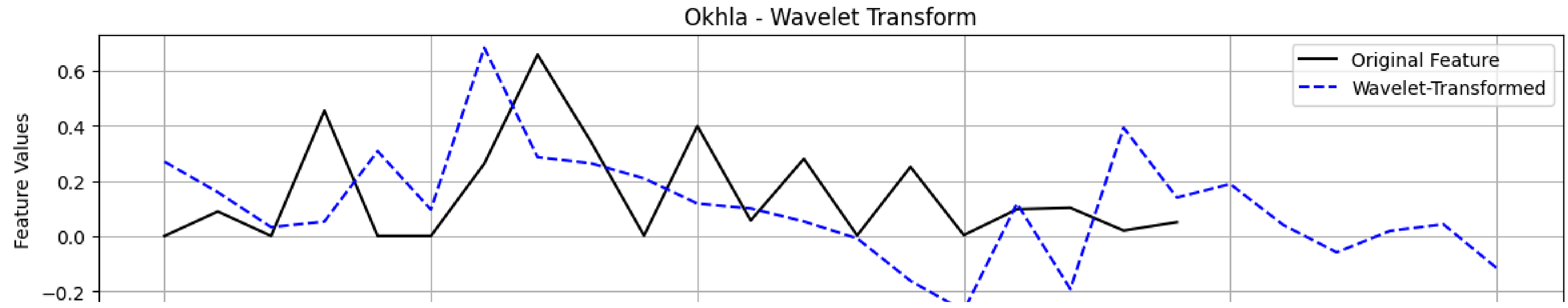
Okhla - Wavelet Transform
Original Feature
Wavelet-Transformed
Feature Values
0.6
0.4
0.2
0.0
−0.2

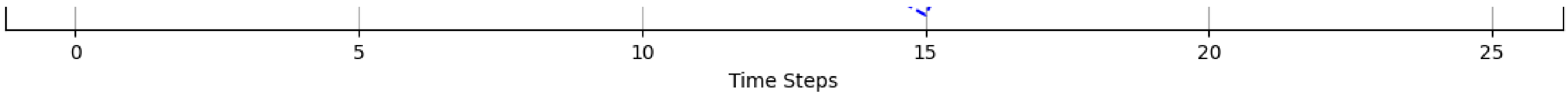
0
5
10
15
20
25
Time Steps

```
import numpy as np
import pandas as pd
import matplotlib.pyplot as plt
from sklearn.preprocessing import StandardScaler
from sklearn.decomposition import PCA

# ============================
# Load Data for Multiple Stations
# ============================
file_paths = {
    'AshokVihar': '/content/AshokVihar_Hourly.csv',
    'DCStadium': '/content/DCStadium_Hourly.csv',
    'DwarkaSec8': '/content/DwarkaSec8_Hourly.csv',
    'NehruNagar': '/content/NehruNagar_Hourly.csv',
    'Najafgarh': '/content/Najafgarh_Hourly.csv',
    'Okhla': '/content/Okhla_Hourly.csv'
}

pca_results = {}  # Store PCA-transformed data and PCA models

# ============================
# PCA Function
# ============================
def apply_pca(X, n_components=2):
    scaler = StandardScaler()
    X_scaled = scaler.fit_transform(X)
    pca = PCA(n_components=n_components)
    X_pca = pca.fit_transform(X_scaled)
    return X_pca, pca

# ============================
# Process Each Station
# ============================
colors = ['b', 'g', 'r', 'c', 'm', 'y']

for station, path in file_paths.items():
    df = pd.read_csv(path)

    # Standardize Data
    scaler = StandardScaler()
    X = scaler.fit_transform(df.iloc[:, :-1].values)  # Features

    # Apply PCA
    X_pca, pca = apply_pca(X)
```

```
    # Store results
    pca_results[station] = (X_pca, pca)

# ============================
# Create Subplots for PCA Visualization
# ============================
fig, axes = plt.subplots(1, 2, figsize=(14, 6))

# **Plot 1: PCA Explained Variance Across All Stations**
for station, (_, pca) in pca_results.items():
    axes[0].plot(range(1, len(pca.explained_variance_ratio_) + 1),
                 pca.explained_variance_ratio_, marker='o', linestyle='--', label=station)

axes[0].set_title('PCA Explained Variance Across Stations')
axes[0].set_xlabel('Principal Component Index')
axes[0].set_ylabel('Explained Variance Ratio')
axes[0].legend()
axes[0].grid(True)

# **Plot 2: 2D PCA Projection for Each Station**
for station, (X_pca, _) in pca_results.items():
    axes[1].scatter(X_pca[:, 0], X_pca[:, 1], alpha=0.5, label=station)

axes[1].set_title('2D PCA Projection of Features Across Stations')
axes[1].set_xlabel('Principal Component 1')
axes[1].set_ylabel('Principal Component 2')
axes[1].legend()
axes[1].grid(True)

plt.tight_layout()
plt.show()
```

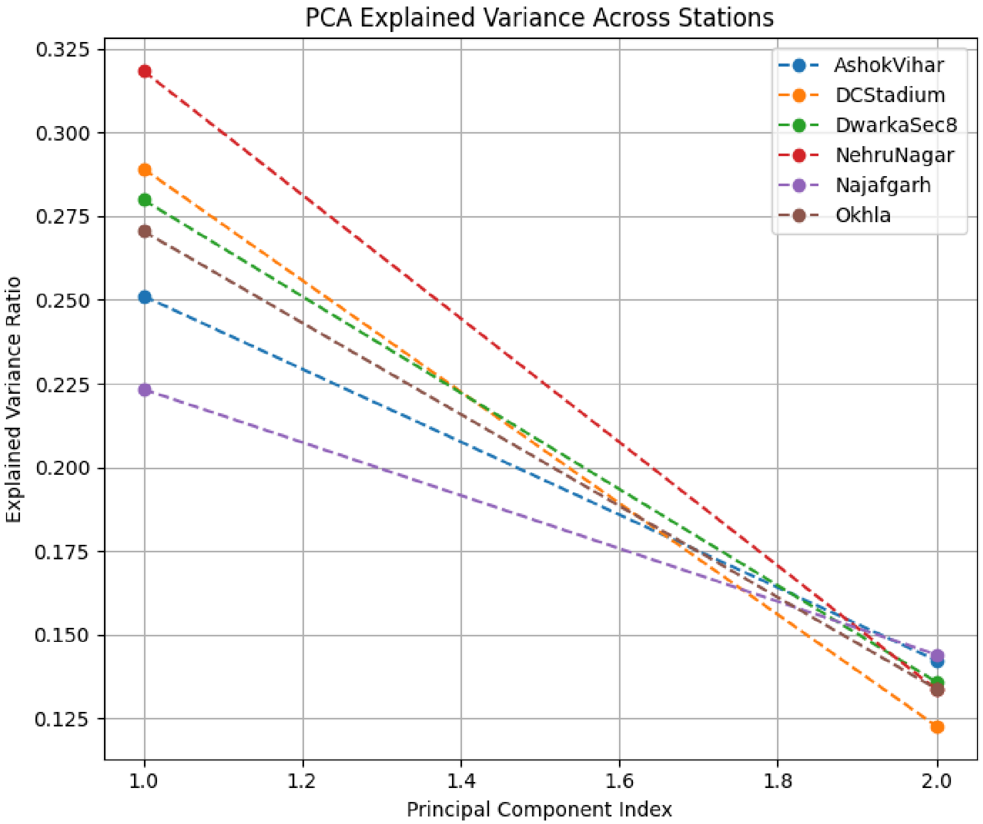

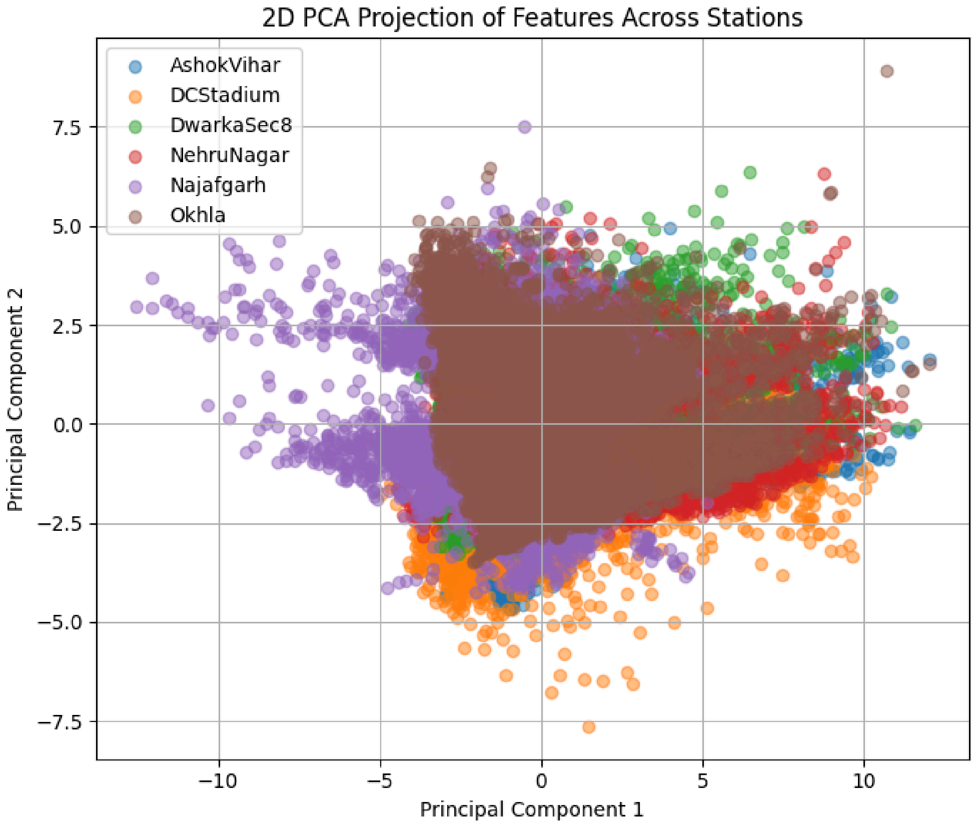


```
!pip install PyWavelets
```

```
Collecting PyWavelets
  Downloading pywavelets-1.8.0-cp311-cp311-manylinux_2_17_x86_64.manylinux2014_x86_64.whl.metadata (9.0 kB)
Requirement already satisfied: numpy<3,>=1.23 in /usr/local/lib/python3.11/dist-packages (from PyWavelets) (1.26.4)
Downloading pywavelets-1.8.0-cp311-cp311-manylinux_2_17_x86_64.manylinux2014_x86_64.whl (4.5 MB)
   ━━━━━━━━━━━━━━━━━━━━━━━━━━━━━━━━━━━━━━━━ 4.5/4.5 MB 34.1 MB/s eta 0:00:00
Installing collected packages: PyWavelets
Successfully installed PyWavelets-1.8.0
```

Start coding or generate with AI.

```
!pip install PyWavelets
```

```
Collecting PyWavelets
  Downloading pywavelets-1.8.0-cp311-cp311-manylinux_2_17_x86_64.manylinux2014_x86_64.whl.metadata (9.0 kB)
Requirement already satisfied: numpy<3,>=1.23 in /usr/local/lib/python3.11/dist-packages (from PyWavelets) (1.26.4)
Downloading pywavelets-1.8.0-cp311-cp311-manylinux_2_17_x86_64.manylinux2014_x86_64.whl (4.5 MB)
   ━━━━━━━━━━━━━━━━━━━━━━━━━━━━━━━━━━━━━━━━ 4.5/4.5 MB 29.8 MB/s eta 0:00:00
Installing collected packages: PyWavelets
Successfully installed PyWavelets-1.8.0
```

Hybrid AOAOA Optimizer

```
import numpy as np
import pandas as pd
import matplotlib.pyplot as plt
from sklearn.decomposition import PCA
from sklearn.preprocessing import StandardScaler, MinMaxScaler
from sklearn.model_selection import train_test_split
from sklearn.metrics import mean_squared_error, mean_absolute_error, r2_score
import tensorflow as tf
from tensorflow.keras.models import Sequential
from tensorflow.keras.layers import LSTM, Dense, Bidirectional
import pywt

# ============================
# Hybrid AOAOA Optimizer
# ============================
class HybridOptimizer:
    def __init__(self, objective_function, lower_bound, upper_bound, population_size, iterations):
        self.objective_function = objective_function
        self.lower_bound = np.array(lower_bound)
        self.upper_bound = np.array(upper_bound)
        self.population_size = population_size
        self.iterations = iterations
        self.population = np.random.uniform(low=self.lower_bound, high=self.upper_bound, size=(population_size, len(lower_bound)))
        self.best_solution = None
        self.best_fitness = float('inf')

    def optimize(self):
        for _ in range(self.iterations):
            for i in range(self.population_size):
                perturbation = np.random.uniform(-0.1, 0.1, size=self.population.shape[1])
                candidate_solution_aquila = self.population[i] + perturbation
                candidate_solution_aquila = np.clip(candidate_solution_aquila, self.lower_bound, self.upper_bound)
```

```
                fitness_aquila = self.objective_function(candidate_solution_aquila)

                if fitness_aquila < self.best_fitness:
                    self.best_fitness = fitness_aquila
                    self.best_solution = candidate_solution_aquila

            for i in range(self.population_size):
                partner_idx = np.random.randint(self.population_size)
                partner = self.population[partner_idx]
                candidate_solution_arithmetic = (self.population[i] + partner) / 2
                candidate_solution_arithmetic = np.clip(candidate_solution_arithmetic, self.lower_bound, self.upper_bound)
                fitness_arithmetic = self.objective_function(candidate_solution_arithmetic)

                if fitness_arithmetic < self.best_fitness:
                    self.best_fitness = fitness_arithmetic
                    self.best_solution = candidate_solution_arithmetic

        return self.best_solution

# ============================
# Feature Extraction
# ============================
def extract_wavelet_features(X, wavelet='db4', level=3, num_features=50):
    features = []
    for sample in X:
        coeffs = pywt.wavedec(sample, wavelet, level=level)
        flattened_coeffs = np.concatenate([c.flatten() for c in coeffs])
        features.append(flattened_coeffs[:num_features])
    return np.array(features)

def apply_pca(X, n_components=10):
    scaler = StandardScaler()
    X_scaled = scaler.fit_transform(X)
    pca = PCA(n_components=n_components)
    return pca.fit_transform(X_scaled)

def extract_combined_features(X):
    X_wavelet = extract_wavelet_features(X)
    X_pca = apply_pca(X_wavelet)
    return X_pca

# ============================
# Bi-LSTM Model Definition
# ============================
```

```
def build_lstm_model(input_shape):
    model = Sequential([
        Bidirectional(LSTM(50, return_sequences=True, input_shape=input_shape)),
        Bidirectional(LSTM(50, return_sequences=False)),
        Dense(1)
    ])
    model.compile(optimizer='adam', loss='mean_squared_error')
    return model

# ============================
# Training & Evaluation
# ============================
def evaluate_model(X, y):
    if X.shape[1] == 0:
        raise ValueError("No features selected! Adjust AOAOA feature selection.")
    X_train, X_test, y_train, y_test = train_test_split(X, y, test_size=0.2, random_state=42)
    X_train = X_train.reshape(X_train.shape[0], X_train.shape[1], 1)
    X_test = X_test.reshape(X_test.shape[0], X_test.shape[1], 1)

    model = build_lstm_model((X_train.shape[1], 1))
    model.fit(X_train, y_train, epochs=50, batch_size=64, validation_data=(X_test, y_test), verbose=1)
    y_pred = model.predict(X_test)

    mse = mean_squared_error(y_test, y_pred)
    mae = mean_absolute_error(y_test, y_pred)
    rmse = np.sqrt(mse)
    r2 = r2_score(y_test, y_pred)

    return mse, mae, rmse, r2, y_test, y_pred

# ============================
# Multi-Station Processing
# ============================
stations = {
    'AshokVihar': '/content/AshokVihar_Hourly.csv',
    'DCStadium': '/content/DCStadium_Hourly.csv',
    'DwarkaSec8': '/content/DwarkaSec8_Hourly.csv',
    'NehruNagar': '/content/NehruNagar_Hourly.csv',
    'Najafgarh': '/content/Najafgarh_Hourly.csv',
    'Okhla': '/content/Okhla_Hourly.csv'
}

threshold = 0.40  # Adjust this value as needed
results = {}
```

```
for station, file_path in stations.items():
    print(f"\nProcessing Station: {station}")

    # Load Data
    df = pd.read_csv(file_path)

    # Preprocessing
    scaler = MinMaxScaler()
    X_full = scaler.fit_transform(df.iloc[:, :-1].values)
    y = scaler.fit_transform(df.iloc[:, -1].values.reshape(-1, 1))

    # Feature Extraction
    X_extracted = extract_combined_features(X_full)

    # Feature Selection with Hybrid AOAOA
    objective_function = lambda x: np.sum(x**2)
    hybrid_optimizer = HybridOptimizer(objective_function, lower_bound=[-1] * X_extracted.shape[1],
                                       upper_bound=[1] * X_extracted.shape[1],
                                       population_size=50, iterations=100)
    selected_features = hybrid_optimizer.optimize()

    # Select features above threshold
    X_selected = X_extracted[:, selected_features > threshold]

    # Final Model Evaluation
    X_final = X_selected if X_selected.shape[1] > 0 else X_extracted
    mse, mae, rmse, r2, y_test, y_pred = evaluate_model(X_final, y)

    # Store results
    results[station] = {"MSE": mse, "MAE": mae, "RMSE": rmse, "R² Score": r2}

    # Plot Feature Importance
    plt.figure(figsize=(10, 5))
    feature_importance = np.abs(selected_features)
    plt.bar(range(len(feature_importance)), feature_importance)
    plt.xlabel('Feature Index')
    plt.ylabel('Importance Score')
    plt.title(f'Feature Importance for {station}')
    plt.show()

    # Plot Actual vs Predicted
    plt.figure(figsize=(10, 5))
    plt.plot(y_test, label="Actual Values", color='black')
```

```
    plt.plot(y_pred, label="Predicted Values", color='blue')
    plt.xlabel('Samples')
    plt.ylabel('Values')
    plt.title(f'Actual vs. Predicted Values (Bi-LSTM) - {station}')
    plt.legend()
    plt.show()

# Print Final Results
print("\nFinal Model Evaluation Across Stations:")
for station, metrics in results.items():
    print(f"\nStation: {station}")
    for metric, value in metrics.items():
        print(f"{metric}: {value:.4f}")
```

```
Processing Station: AshokVihar
/usr/local/lib/python3.11/dist-packages/pywt/_multilevel.py:43: UserWarning: Level value of 3 is too high: all coefficients will experience boundary effe
  warnings.warn(
Epoch 1/50
/usr/local/lib/python3.11/dist-packages/keras/src/layers/rnn/rnn.py:200: UserWarning: Do not pass an `input_shape`/`input_dim` argument to a layer. When
  super().__init__(**kwargs)
147/147 ━━━━━━━━━━━━━━━━━━━━ 21s 43ms/step - loss: 0.0050 - val_loss: 0.0027
Epoch 2/50
147/147 ━━━━━━━━━━━━━━━━━━━━ 9s 35ms/step - loss: 0.0026 - val_loss: 0.0026
Epoch 3/50
147/147 ━━━━━━━━━━━━━━━━━━━━ 10s 34ms/step - loss: 0.0021 - val_loss: 0.0018
Epoch 4/50
147/147 ━━━━━━━━━━━━━━━━━━━━ 6s 41ms/step - loss: 0.0019 - val_loss: 0.0016
Epoch 5/50
147/147 ━━━━━━━━━━━━━━━━━━━━ 9s 35ms/step - loss: 0.0018 - val_loss: 0.0014
Epoch 6/50
147/147 ━━━━━━━━━━━━━━━━━━━━ 10s 34ms/step - loss: 0.0015 - val_loss: 0.0015
Epoch 7/50
147/147 ━━━━━━━━━━━━━━━━━━━━ 7s 47ms/step - loss: 0.0013 - val_loss: 0.0015
Epoch 8/50
147/147 ━━━━━━━━━━━━━━━━━━━━ 5s 33ms/step - loss: 0.0013 - val_loss: 0.0013
Epoch 9/50
147/147 ━━━━━━━━━━━━━━━━━━━━ 5s 36ms/step - loss: 0.0013 - val_loss: 0.0013
Epoch 10/50
147/147 ━━━━━━━━━━━━━━━━━━━━ 10s 32ms/step - loss: 0.0013 - val_loss: 0.0012
Epoch 11/50
147/147 ━━━━━━━━━━━━━━━━━━━━ 7s 46ms/step - loss: 0.0013 - val_loss: 0.0011
Epoch 12/50
147/147 ━━━━━━━━━━━━━━━━━━━━ 5s 35ms/step - loss: 0.0012 - val_loss: 0.0011
Epoch 13/50
147/147 ━━━━━━━━━━━━━━━━━━━━ 6s 44ms/step - loss: 0.0012 - val_loss: 0.0011
Epoch 14/50
147/147 ━━━━━━━━━━━━━━━━━━━━ 9s 35ms/step - loss: 0.0011 - val_loss: 0.0012
Epoch 15/50
147/147 ━━━━━━━━━━━━━━━━━━━━ 10s 33ms/step - loss: 0.0012 - val_loss: 0.0013
Epoch 16/50
147/147 ━━━━━━━━━━━━━━━━━━━━ 7s 46ms/step - loss: 0.0012 - val_loss: 0.0010
Epoch 17/50
147/147 ━━━━━━━━━━━━━━━━━━━━ 5s 34ms/step - loss: 0.0010 - val_loss: 0.0010
Epoch 18/50
147/147 ━━━━━━━━━━━━━━━━━━━━ 6s 38ms/step - loss: 9.9015e-04 - val_loss: 0.0011
Epoch 19/50
147/147 ━━━━━━━━━━━━━━━━━━━━ 10s 35ms/step - loss: 9.0943e-04 - val_loss: 0.0010
Epoch 20/50
147/147 ━━━━━━━━━━━━━━━━━━━━ 7s 45ms/step - loss: 9.7096e-04 - val_loss: 0.0010
Epoch 21/50
147/147 ━━━━━━━━━━━━━━━━━━━━ 9s 40ms/step - loss: 9.0710e-04 - val_loss: 9.1676e-04
Epoch 22/50
```

```
147/147 ━━━━━━━━━━━━━━━━━━━━ 9s 34ms/step - loss: 8.5566e-04 - val_loss: 8.7452e-04
Epoch 23/50
147/147 ━━━━━━━━━━━━━━━━━━━━ 6s 44ms/step - loss: 8.7860e-04 - val_loss: 9.1311e-04
Epoch 24/50
147/147 ━━━━━━━━━━━━━━━━━━━━ 5s 35ms/step - loss: 8.2333e-04 - val_loss: 8.8105e-04
Epoch 25/50
147/147 ━━━━━━━━━━━━━━━━━━━━ 6s 42ms/step - loss: 8.5571e-04 - val_loss: 8.7409e-04
Epoch 26/50
147/147 ━━━━━━━━━━━━━━━━━━━━ 5s 35ms/step - loss: 7.6096e-04 - val_loss: 9.5505e-04
Epoch 27/50
147/147 ━━━━━━━━━━━━━━━━━━━━ 12s 44ms/step - loss: 8.0295e-04 - val_loss: 7.9894e-04
Epoch 28/50
147/147 ━━━━━━━━━━━━━━━━━━━━ 10s 41ms/step - loss: 7.7363e-04 - val_loss: 8.5071e-04
Epoch 29/50
147/147 ━━━━━━━━━━━━━━━━━━━━ 9s 35ms/step - loss: 7.3364e-04 - val_loss: 8.0228e-04
Epoch 30/50
147/147 ━━━━━━━━━━━━━━━━━━━━ 10s 33ms/step - loss: 7.5724e-04 - val_loss: 7.5254e-04
Epoch 31/50
147/147 ━━━━━━━━━━━━━━━━━━━━ 7s 45ms/step - loss: 7.2114e-04 - val_loss: 8.3790e-04
Epoch 32/50
147/147 ━━━━━━━━━━━━━━━━━━━━ 5s 36ms/step - loss: 6.7267e-04 - val_loss: 8.6476e-04
Epoch 33/50
147/147 ━━━━━━━━━━━━━━━━━━━━ 11s 42ms/step - loss: 7.3792e-04 - val_loss: 7.3454e-04
Epoch 34/50
147/147 ━━━━━━━━━━━━━━━━━━━━ 11s 46ms/step - loss: 6.0954e-04 - val_loss: 6.6948e-04
Epoch 35/50
147/147 ━━━━━━━━━━━━━━━━━━━━ 5s 33ms/step - loss: 5.7259e-04 - val_loss: 7.5022e-04
Epoch 36/50
147/147 ━━━━━━━━━━━━━━━━━━━━ 6s 39ms/step - loss: 6.2708e-04 - val_loss: 7.1176e-04
Epoch 37/50
147/147 ━━━━━━━━━━━━━━━━━━━━ 6s 39ms/step - loss: 6.1966e-04 - val_loss: 7.4061e-04
Epoch 38/50
147/147 ━━━━━━━━━━━━━━━━━━━━ 11s 46ms/step - loss: 6.0248e-04 - val_loss: 7.2235e-04
Epoch 39/50
147/147 ━━━━━━━━━━━━━━━━━━━━ 5s 33ms/step - loss: 5.7292e-04 - val_loss: 8.1419e-04
Epoch 40/50
147/147 ━━━━━━━━━━━━━━━━━━━━ 7s 44ms/step - loss: 5.9283e-04 - val_loss: 6.4540e-04
Epoch 41/50
147/147 ━━━━━━━━━━━━━━━━━━━━ 9s 36ms/step - loss: 5.6348e-04 - val_loss: 6.9703e-04
Epoch 42/50
147/147 ━━━━━━━━━━━━━━━━━━━━ 6s 44ms/step - loss: 5.0917e-04 - val_loss: 6.4120e-04
Epoch 43/50
147/147 ━━━━━━━━━━━━━━━━━━━━ 5s 33ms/step - loss: 5.2102e-04 - val_loss: 6.3135e-04
Epoch 44/50
147/147 ━━━━━━━━━━━━━━━━━━━━ 7s 45ms/step - loss: 5.1599e-04 - val_loss: 6.1281e-04
Epoch 45/50
147/147 ━━━━━━━━━━━━━━━━━━━━ 9s 35ms/step - loss: 4.9235e-04 - val_loss: 6.1656e-04
Epoch 46/50
147/147 ━━━━━━━━━━━━━━━━━━━━ 7s 46ms/step - loss: 4.8343e-04 - val_loss: 6.3667e-04
```

```
Epoch 47/50
147/147 ━━━━━━━━━━━━━━━━━━━━ 9s 36ms/step - loss: 4.8270e-04 - val_loss: 5.8894e-04
Epoch 48/50
147/147 ━━━━━━━━━━━━━━━━━━━━ 6s 40ms/step - loss: 4.7975e-04 - val_loss: 6.1239e-04
Epoch 49/50
147/147 ━━━━━━━━━━━━━━━━━━━━ 11s 44ms/step - loss: 4.7334e-04 - val_loss: 5.8132e-04
Epoch 50/50
147/147 ━━━━━━━━━━━━━━━━━━━━ 5s 35ms/step - loss: 4.5962e-04 - val_loss: 6.2070e-04
74/74 ━━━━━━━━━━━━━━━━━━━━ 2s 17ms/step
```

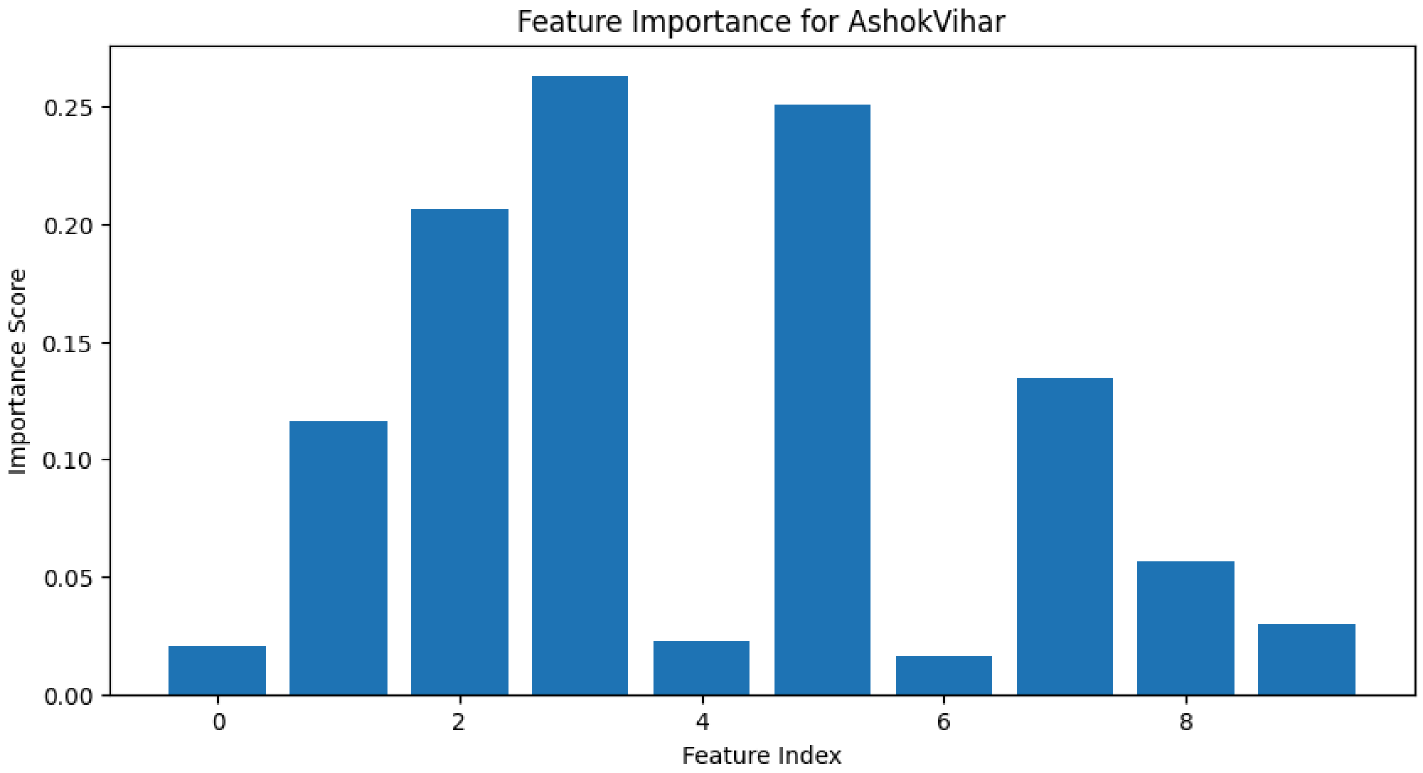

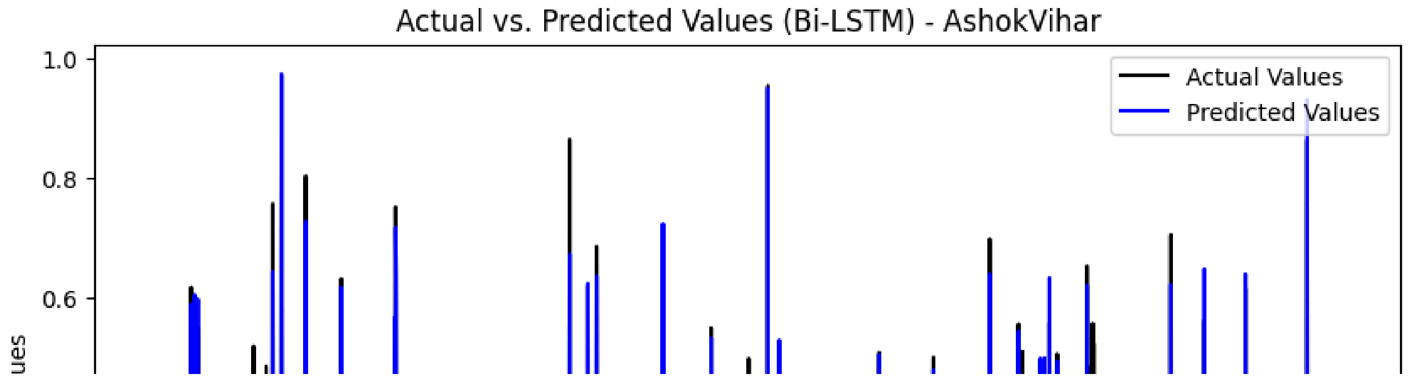

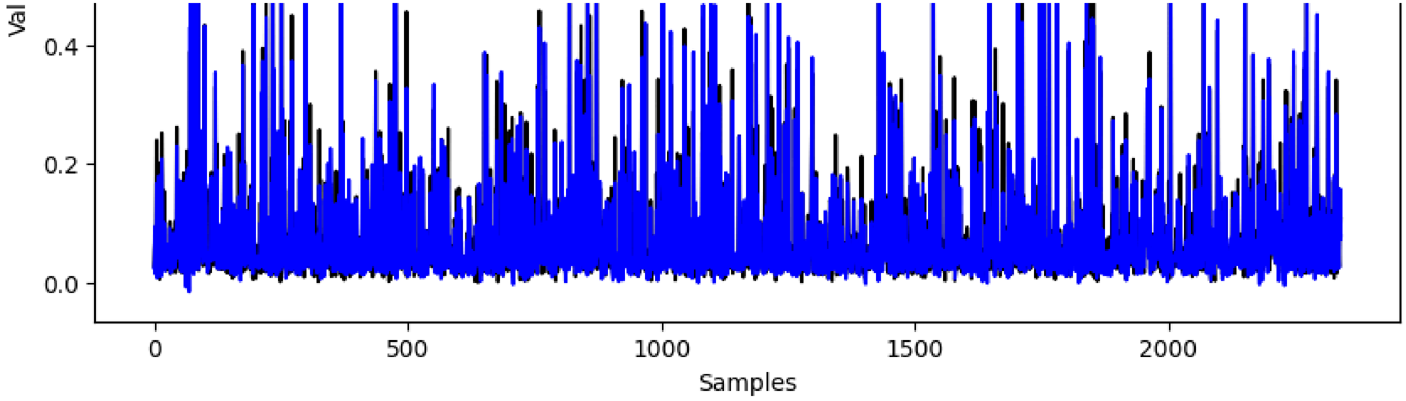


```
Processing Station: DCStadium
/usr/local/lib/python3.11/dist-packages/pywt/_multilevel.py:43: UserWarning: Level value of 3 is too high: all coefficients will experience boundary effe
  warnings.warn(
Epoch 1/50
/usr/local/lib/python3.11/dist-packages/keras/src/layers/rnn/rnn.py:200: UserWarning: Do not pass an `input_shape`/`input_dim` argument to a layer. When
  super().__init__(**kwargs)
147/147 ━━━━━━━━━━━━━━━━━━━━ 15s 46ms/step - loss: 0.0250 - val_loss: 0.0105
Epoch 2/50
147/147 ━━━━━━━━━━━━━━━━━━━━ 5s 34ms/step - loss: 0.0078 - val_loss: 0.0028
Epoch 3/50
147/147 ━━━━━━━━━━━━━━━━━━━━ 6s 43ms/step - loss: 0.0028 - val_loss: 0.0032
Epoch 4/50
147/147 ━━━━━━━━━━━━━━━━━━━━ 5s 32ms/step - loss: 0.0025 - val_loss: 0.0021
Epoch 5/50
147/147 ━━━━━━━━━━━━━━━━━━━━ 6s 42ms/step - loss: 0.0021 - val_loss: 0.0020
Epoch 6/50
147/147 ━━━━━━━━━━━━━━━━━━━━ 5s 33ms/step - loss: 0.0023 - val_loss: 0.0018
Epoch 7/50
147/147 ━━━━━━━━━━━━━━━━━━━━ 5s 33ms/step - loss: 0.0022 - val_loss: 0.0018
Epoch 8/50
147/147 ━━━━━━━━━━━━━━━━━━━━ 7s 44ms/step - loss: 0.0019 - val_loss: 0.0020
Epoch 9/50
147/147 ━━━━━━━━━━━━━━━━━━━━ 5s 32ms/step - loss: 0.0019 - val_loss: 0.0017
Epoch 10/50
147/147 ━━━━━━━━━━━━━━━━━━━━ 7s 43ms/step - loss: 0.0018 - val_loss: 0.0016
Epoch 11/50
147/147 ━━━━━━━━━━━━━━━━━━━━ 9s 31ms/step - loss: 0.0018 - val_loss: 0.0016
Epoch 12/50
147/147 ━━━━━━━━━━━━━━━━━━━━ 6s 43ms/step - loss: 0.0016 - val_loss: 0.0015
Epoch 13/50
147/147 ━━━━━━━━━━━━━━━━━━━━ 5s 32ms/step - loss: 0.0019 - val_loss: 0.0015
Epoch 14/50
147/147 ━━━━━━━━━━━━━━━━━━━━ 6s 41ms/step - loss: 0.0017 - val_loss: 0.0015
Epoch 15/50
```

```
147/147 ━━━━━━━━━━━━━━━━━━━━ 9s 34ms/step - loss: 0.0015 - val_loss: 0.0014
Epoch 16/50
147/147 ━━━━━━━━━━━━━━━━━━━━ 7s 45ms/step - loss: 0.0015 - val_loss: 0.0014
Epoch 17/50
147/147 ━━━━━━━━━━━━━━━━━━━━ 9s 34ms/step - loss: 0.0015 - val_loss: 0.0014
Epoch 18/50
147/147 ━━━━━━━━━━━━━━━━━━━━ 6s 43ms/step - loss: 0.0015 - val_loss: 0.0014
Epoch 19/50
147/147 ━━━━━━━━━━━━━━━━━━━━ 5s 32ms/step - loss: 0.0013 - val_loss: 0.0013
Epoch 20/50
147/147 ━━━━━━━━━━━━━━━━━━━━ 6s 41ms/step - loss: 0.0014 - val_loss: 0.0013
Epoch 21/50
147/147 ━━━━━━━━━━━━━━━━━━━━ 5s 32ms/step - loss: 0.0013 - val_loss: 0.0012
Epoch 22/50
147/147 ━━━━━━━━━━━━━━━━━━━━ 5s 34ms/step - loss: 0.0012 - val_loss: 0.0016
Epoch 23/50
147/147 ━━━━━━━━━━━━━━━━━━━━ 7s 45ms/step - loss: 0.0013 - val_loss: 0.0014
Epoch 24/50
147/147 ━━━━━━━━━━━━━━━━━━━━ 5s 34ms/step - loss: 0.0012 - val_loss: 0.0011
Epoch 25/50
147/147 ━━━━━━━━━━━━━━━━━━━━ 7s 45ms/step - loss: 0.0011 - val_loss: 0.0013
Epoch 26/50
147/147 ━━━━━━━━━━━━━━━━━━━━ 9s 34ms/step - loss: 0.0011 - val_loss: 0.0011
Epoch 27/50
147/147 ━━━━━━━━━━━━━━━━━━━━ 7s 46ms/step - loss: 0.0012 - val_loss: 0.0012
Epoch 28/50
147/147 ━━━━━━━━━━━━━━━━━━━━ 9s 37ms/step - loss: 0.0011 - val_loss: 0.0014
Epoch 29/50
147/147 ━━━━━━━━━━━━━━━━━━━━ 6s 40ms/step - loss: 0.0011 - val_loss: 0.0012
Epoch 30/50
147/147 ━━━━━━━━━━━━━━━━━━━━ 5s 33ms/step - loss: 0.0010 - val_loss: 9.9844e-04
Epoch 31/50
147/147 ━━━━━━━━━━━━━━━━━━━━ 6s 41ms/step - loss: 9.0488e-04 - val_loss: 0.0010
Epoch 32/50
147/147 ━━━━━━━━━━━━━━━━━━━━ 5s 34ms/step - loss: 0.0011 - val_loss: 9.7190e-04
Epoch 33/50
147/147 ━━━━━━━━━━━━━━━━━━━━ 6s 40ms/step - loss: 9.5491e-04 - val_loss: 0.0010
Epoch 34/50
147/147 ━━━━━━━━━━━━━━━━━━━━ 5s 34ms/step - loss: 0.0010 - val_loss: 0.0010
Epoch 35/50
147/147 ━━━━━━━━━━━━━━━━━━━━ 5s 33ms/step - loss: 9.9467e-04 - val_loss: 9.5736e-04
Epoch 36/50
147/147 ━━━━━━━━━━━━━━━━━━━━ 7s 46ms/step - loss: 8.6171e-04 - val_loss: 9.9285e-04
Epoch 37/50
147/147 ━━━━━━━━━━━━━━━━━━━━ 8s 34ms/step - loss: 8.4436e-04 - val_loss: 0.0011
Epoch 38/50
147/147 ━━━━━━━━━━━━━━━━━━━━ 6s 40ms/step - loss: 9.6895e-04 - val_loss: 0.0010
Epoch 39/50
147/147 ━━━━━━━━━━━━━━━━━━━━ 11s 42ms/step - loss: 7.8654e-04 - val_loss: 9.4700e-04
[illegible]
```

```
Epoch 40/50
147/147 ━━━━━━━━━━━━━━━━━━━━ 5s 32ms/step - loss: 8.4355e-04 - val_loss: 9.9127e-04
Epoch 41/50
147/147 ━━━━━━━━━━━━━━━━━━━━ 5s 34ms/step - loss: 8.7694e-04 - val_loss: 9.5036e-04
Epoch 42/50
147/147 ━━━━━━━━━━━━━━━━━━━━ 6s 42ms/step - loss: 8.3598e-04 - val_loss: 9.5562e-04
Epoch 43/50
147/147 ━━━━━━━━━━━━━━━━━━━━ 10s 38ms/step - loss: 8.2370e-04 - val_loss: 9.0984e-04
Epoch 44/50
147/147 ━━━━━━━━━━━━━━━━━━━━ 9s 32ms/step - loss: 8.4602e-04 - val_loss: 9.7411e-04
Epoch 45/50
147/147 ━━━━━━━━━━━━━━━━━━━━ 7s 43ms/step - loss: 8.5634e-04 - val_loss: 9.4784e-04
Epoch 46/50
147/147 ━━━━━━━━━━━━━━━━━━━━ 5s 31ms/step - loss: 8.2532e-04 - val_loss: 9.2229e-04
Epoch 47/50
147/147 ━━━━━━━━━━━━━━━━━━━━ 6s 38ms/step - loss: 7.2706e-04 - val_loss: 9.0844e-04
Epoch 48/50
147/147 ━━━━━━━━━━━━━━━━━━━━ 9s 32ms/step - loss: 7.6359e-04 - val_loss: 8.9790e-04
Epoch 49/50
147/147 ━━━━━━━━━━━━━━━━━━━━ 7s 45ms/step - loss: 7.6283e-04 - val_loss: 8.7538e-04
Epoch 50/50
147/147 ━━━━━━━━━━━━━━━━━━━━ 5s 34ms/step - loss: 7.7321e-04 - val_loss: 8.2615e-04
74/74 ━━━━━━━━━━━━━━━━━━━━ 2s 17ms/step
```

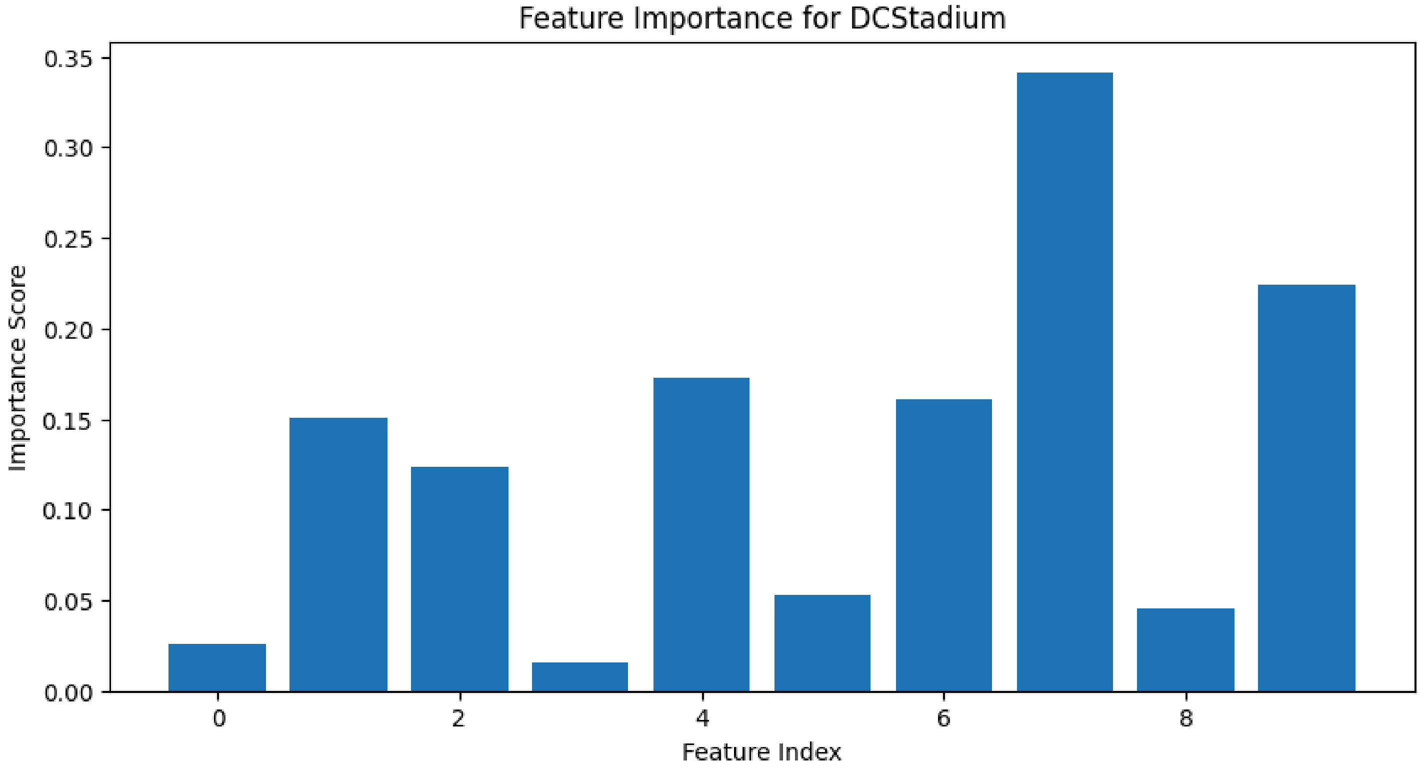

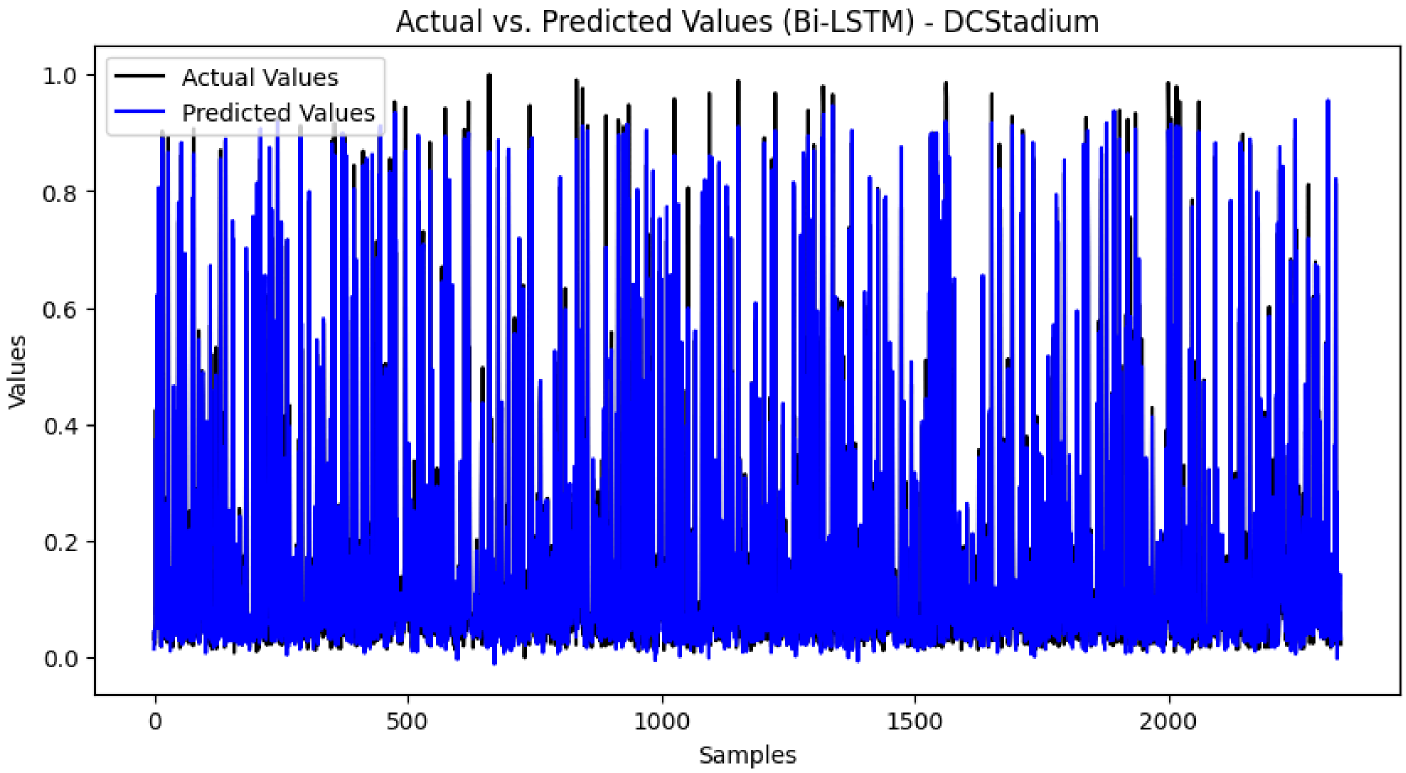


```
Processing Station: DwarkaSec8
/usr/local/lib/python3.11/dist-packages/pywt/_multilevel.py:43: UserWarning: Level value of 3 is too high: all coefficients will experience boundary effe
  warnings.warn(
Epoch 1/50
/usr/local/lib/python3.11/dist-packages/keras/src/layers/rnn/rnn.py:200: UserWarning: Do not pass an `input_shape`/`input_dim` argument to a layer. When
  super().__init__(**kwargs)
147/147 ━━━━━━━━━━━━━━━━━━━━ 16s 48ms/step - loss: 0.0099 - val_loss: 0.0036
Epoch 2/50
147/147 ━━━━━━━━━━━━━━━━━━━━ 5s 32ms/step - loss: 0.0032 - val_loss: 0.0020
Epoch 3/50
147/147 ━━━━━━━━━━━━━━━━━━━━ 5s 33ms/step - loss: 0.0020 - val_loss: 0.0017
Epoch 4/50
147/147 ━━━━━━━━━━━━━━━━━━━━ 6s 44ms/step - loss: 0.0017 - val_loss: 0.0015
Epoch 5/50
147/147 ━━━━━━━━━━━━━━━━━━━━ 5s 34ms/step - loss: 0.0015 - val_loss: 0.0015
Epoch 6/50
147/147 ━━━━━━━━━━━━━━━━━━━━ 7s 44ms/step - loss: 0.0015 - val_loss: 0.0014
Epoch 7/50
147/147 ━━━━━━━━━━━━━━━━━━━━ 5s 32ms/step - loss: 0.0014 - val_loss: 0.0014
Epoch 8/50
147/147 ━━━━━━━━━━━━━━━━━━━━ 5s 33ms/step - loss: 0.0013 - val_loss: 0.0013
```

```
[illegible]
Epoch 9/50
147/147 ━━━━━━━━━━━━━━━━━━━━ 6s 42ms/step - loss: 0.0012 - val_loss: 0.0013
Epoch 10/50
147/147 ━━━━━━━━━━━━━━━━━━━━ 5s 33ms/step - loss: 0.0013 - val_loss: 0.0014
Epoch 11/50
147/147 ━━━━━━━━━━━━━━━━━━━━ 6s 44ms/step - loss: 0.0012 - val_loss: 0.0013
Epoch 12/50
147/147 ━━━━━━━━━━━━━━━━━━━━ 9s 33ms/step - loss: 0.0012 - val_loss: 0.0014
Epoch 13/50
147/147 ━━━━━━━━━━━━━━━━━━━━ 7s 44ms/step - loss: 0.0011 - val_loss: 0.0011
Epoch 14/50
147/147 ━━━━━━━━━━━━━━━━━━━━ 9s 33ms/step - loss: 0.0010 - val_loss: 0.0012
Epoch 15/50
147/147 ━━━━━━━━━━━━━━━━━━━━ 6s 41ms/step - loss: 0.0010 - val_loss: 0.0011
Epoch 16/50
147/147 ━━━━━━━━━━━━━━━━━━━━ 5s 35ms/step - loss: 0.0011 - val_loss: 0.0011
Epoch 17/50
147/147 ━━━━━━━━━━━━━━━━━━━━ 10s 32ms/step - loss: 0.0010 - val_loss: 0.0011
Epoch 18/50
147/147 ━━━━━━━━━━━━━━━━━━━━ 6s 40ms/step - loss: 0.0010 - val_loss: 0.0011
Epoch 19/50
147/147 ━━━━━━━━━━━━━━━━━━━━ 6s 39ms/step - loss: 9.4165e-04 - val_loss: 0.0011
Epoch 20/50
147/147 ━━━━━━━━━━━━━━━━━━━━ 5s 34ms/step - loss: 9.5437e-04 - val_loss: 0.0011
Epoch 21/50
147/147 ━━━━━━━━━━━━━━━━━━━━ 7s 45ms/step - loss: 9.7489e-04 - val_loss: 9.5404e-04
Epoch 22/50
147/147 ━━━━━━━━━━━━━━━━━━━━ 5s 32ms/step - loss: 8.4778e-04 - val_loss: 0.0011
Epoch 23/50
147/147 ━━━━━━━━━━━━━━━━━━━━ 6s 43ms/step - loss: 8.5270e-04 - val_loss: 0.0010
Epoch 24/50
147/147 ━━━━━━━━━━━━━━━━━━━━ 5s 34ms/step - loss: 8.4608e-04 - val_loss: 0.0011
Epoch 25/50
147/147 ━━━━━━━━━━━━━━━━━━━━ 12s 44ms/step - loss: 8.7773e-04 - val_loss: 9.6097e-04
Epoch 26/50
147/147 ━━━━━━━━━━━━━━━━━━━━ 5s 34ms/step - loss: 8.2467e-04 - val_loss: 9.0129e-04
Epoch 27/50
147/147 ━━━━━━━━━━━━━━━━━━━━ 6s 44ms/step - loss: 8.4716e-04 - val_loss: 9.7565e-04
Epoch 28/50
147/147 ━━━━━━━━━━━━━━━━━━━━ 9s 34ms/step - loss: 8.1480e-04 - val_loss: 9.3495e-04
Epoch 29/50
147/147 ━━━━━━━━━━━━━━━━━━━━ 7s 45ms/step - loss: 7.6727e-04 - val_loss: 9.2930e-04
Epoch 30/50
147/147 ━━━━━━━━━━━━━━━━━━━━ 9s 34ms/step - loss: 7.6383e-04 - val_loss: 9.7134e-04
Epoch 31/50
147/147 ━━━━━━━━━━━━━━━━━━━━ 6s 42ms/step - loss: 8.0659e-04 - val_loss: 9.2024e-04
Epoch 32/50
147/147 ━━━━━━━━━━━━━━━━━━━━ 5s 34ms/step - loss: 7.6881e-04 - val_loss: 8.7095e-04
Epoch 33/50
```

```
147/147 ━━━━━━━━━━━━━━━━━━━━ 7s 47ms/step - loss: 7.6933e-04 - val_loss: 8.3967e-04
Epoch 34/50
147/147 ━━━━━━━━━━━━━━━━━━━━ 8s 31ms/step - loss: 7.0825e-04 - val_loss: 8.7872e-04
Epoch 35/50
147/147 ━━━━━━━━━━━━━━━━━━━━ 13s 86ms/step - loss: 7.0203e-04 - val_loss: 8.3683e-04
Epoch 36/50
147/147 ━━━━━━━━━━━━━━━━━━━━ 13s 34ms/step - loss: 7.3177e-04 - val_loss: 8.9462e-04
Epoch 37/50
147/147 ━━━━━━━━━━━━━━━━━━━━ 7s 46ms/step - loss: 6.7938e-04 - val_loss: 8.7744e-04
Epoch 38/50
147/147 ━━━━━━━━━━━━━━━━━━━━ 5s 35ms/step - loss: 7.0882e-04 - val_loss: 8.5936e-04
Epoch 39/50
147/147 ━━━━━━━━━━━━━━━━━━━━ 6s 41ms/step - loss: 6.7362e-04 - val_loss: 8.3124e-04
Epoch 40/50
147/147 ━━━━━━━━━━━━━━━━━━━━ 9s 33ms/step - loss: 6.6975e-04 - val_loss: 9.1891e-04
Epoch 41/50
147/147 ━━━━━━━━━━━━━━━━━━━━ 7s 46ms/step - loss: 7.0089e-04 - val_loss: 8.1752e-04
Epoch 42/50
147/147 ━━━━━━━━━━━━━━━━━━━━ 8s 33ms/step - loss: 6.3664e-04 - val_loss: 8.8697e-04
Epoch 43/50
147/147 ━━━━━━━━━━━━━━━━━━━━ 7s 46ms/step - loss: 6.6667e-04 - val_loss: 8.3898e-04
Epoch 44/50
147/147 ━━━━━━━━━━━━━━━━━━━━ 9s 37ms/step - loss: 6.2346e-04 - val_loss: 7.7500e-04
Epoch 45/50
147/147 ━━━━━━━━━━━━━━━━━━━━ 6s 41ms/step - loss: 6.6902e-04 - val_loss: 7.9995e-04
Epoch 46/50
147/147 ━━━━━━━━━━━━━━━━━━━━ 11s 46ms/step - loss: 6.3158e-04 - val_loss: 7.7646e-04
Epoch 47/50
147/147 ━━━━━━━━━━━━━━━━━━━━ 5s 32ms/step - loss: 6.1065e-04 - val_loss: 8.2635e-04
Epoch 48/50
147/147 ━━━━━━━━━━━━━━━━━━━━ 6s 36ms/step - loss: 6.2063e-04 - val_loss: 7.8996e-04
Epoch 49/50
147/147 ━━━━━━━━━━━━━━━━━━━━ 10s 34ms/step - loss: 5.8639e-04 - val_loss: 7.2360e-04
Epoch 50/50
147/147 ━━━━━━━━━━━━━━━━━━━━ 7s 44ms/step - loss: 5.7019e-04 - val_loss: 7.2499e-04
74/74 ━━━━━━━━━━━━━━━━━━━━ 2s 17ms/step
```

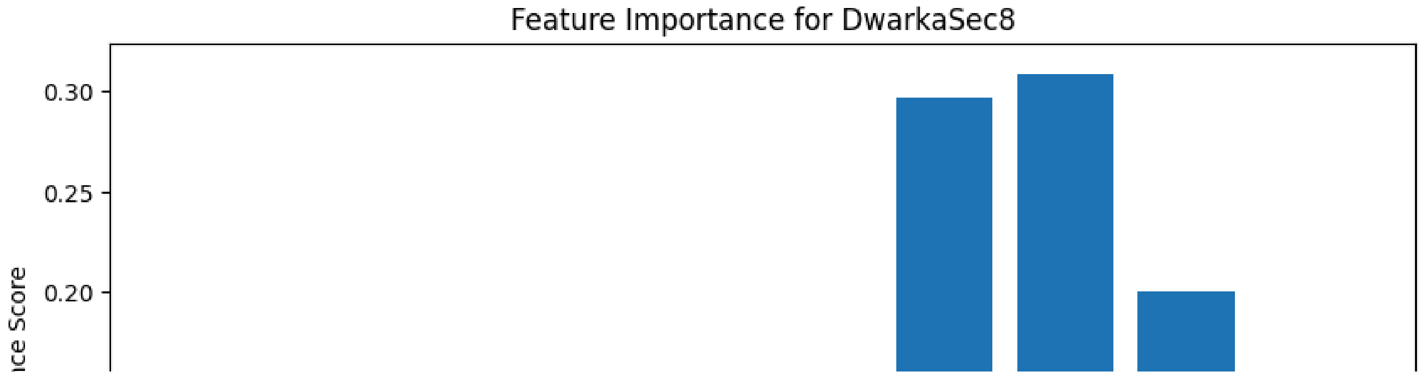

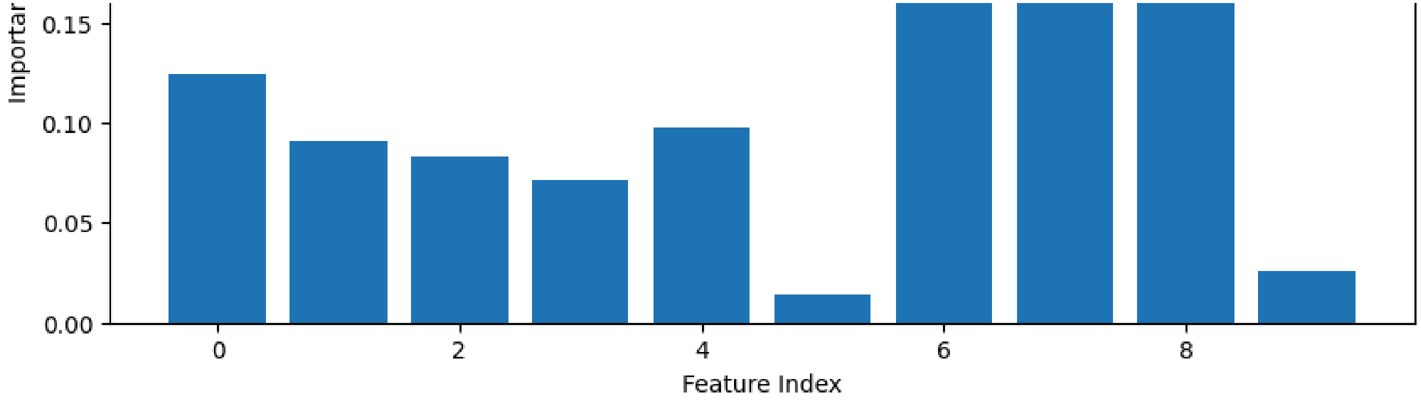

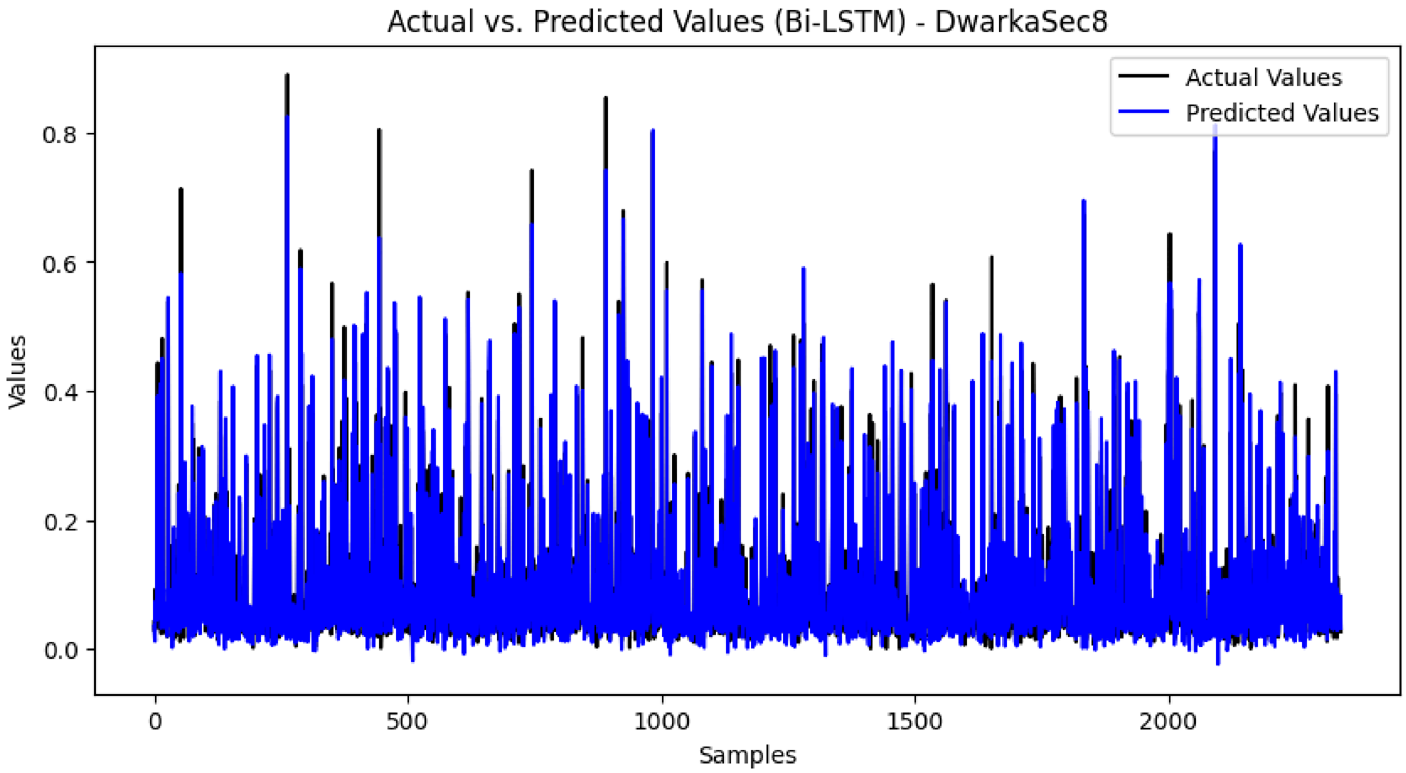


```
Processing Station: NehruNagar
/usr/local/lib/python3.11/dist-packages/pywt/_multilevel.py:43: UserWarning: Level value of 3 is too high: all coefficients will experience boundary effe
  warnings.warn(
Epoch 1/50
/usr/local/lib/python3.11/dist-packages/keras/src/layers/rnn/rnn.py:200: UserWarning: Do not pass an `input_shape`/`input_dim` argument to a layer. When
  super().__init__(**kwargs)
147/147 ━━━━━━━━━━━━━━━━━━━━ 15s 42ms/step - loss: 0.0174 - val_loss: 0.0055
```

```
Epoch 2/50
147/147 ━━━━━━━━━━━━━━━━━━━━ 9s 35ms/step - loss: 0.0047 - val_loss: 0.0041
Epoch 3/50
147/147 ━━━━━━━━━━━━━━━━━━━━ 7s 46ms/step - loss: 0.0031 - val_loss: 0.0023
Epoch 4/50
147/147 ━━━━━━━━━━━━━━━━━━━━ 5s 35ms/step - loss: 0.0021 - val_loss: 0.0020
Epoch 5/50
147/147 ━━━━━━━━━━━━━━━━━━━━ 11s 40ms/step - loss: 0.0018 - val_loss: 0.0025
Epoch 6/50
147/147 ━━━━━━━━━━━━━━━━━━━━ 5s 35ms/step - loss: 0.0018 - val_loss: 0.0019
Epoch 7/50
147/147 ━━━━━━━━━━━━━━━━━━━━ 7s 49ms/step - loss: 0.0018 - val_loss: 0.0020
Epoch 8/50
147/147 ━━━━━━━━━━━━━━━━━━━━ 5s 35ms/step - loss: 0.0016 - val_loss: 0.0019
Epoch 9/50
147/147 ━━━━━━━━━━━━━━━━━━━━ 10s 33ms/step - loss: 0.0016 - val_loss: 0.0017
Epoch 10/50
147/147 ━━━━━━━━━━━━━━━━━━━━ 5s 35ms/step - loss: 0.0017 - val_loss: 0.0017
Epoch 11/50
147/147 ━━━━━━━━━━━━━━━━━━━━ 11s 37ms/step - loss: 0.0015 - val_loss: 0.0016
Epoch 12/50
147/147 ━━━━━━━━━━━━━━━━━━━━ 7s 49ms/step - loss: 0.0015 - val_loss: 0.0017
Epoch 13/50
147/147 ━━━━━━━━━━━━━━━━━━━━ 5s 36ms/step - loss: 0.0014 - val_loss: 0.0015
Epoch 14/50
147/147 ━━━━━━━━━━━━━━━━━━━━ 10s 36ms/step - loss: 0.0014 - val_loss: 0.0014
Epoch 15/50
147/147 ━━━━━━━━━━━━━━━━━━━━ 11s 41ms/step - loss: 0.0013 - val_loss: 0.0013
Epoch 16/50
147/147 ━━━━━━━━━━━━━━━━━━━━ 5s 36ms/step - loss: 0.0012 - val_loss: 0.0011
Epoch 17/50
147/147 ━━━━━━━━━━━━━━━━━━━━ 7s 48ms/step - loss: 0.0010 - val_loss: 0.0011
Epoch 18/50
147/147 ━━━━━━━━━━━━━━━━━━━━ 5s 33ms/step - loss: 0.0011 - val_loss: 0.0010
Epoch 19/50
147/147 ━━━━━━━━━━━━━━━━━━━━ 6s 41ms/step - loss: 9.4353e-04 - val_loss: 0.0011
Epoch 20/50
147/147 ━━━━━━━━━━━━━━━━━━━━ 5s 36ms/step - loss: 9.2026e-04 - val_loss: 0.0011
Epoch 21/50
147/147 ━━━━━━━━━━━━━━━━━━━━ 12s 48ms/step - loss: 9.5789e-04 - val_loss: 8.7074e-04
Epoch 22/50
147/147 ━━━━━━━━━━━━━━━━━━━━ 8s 34ms/step - loss: 8.3566e-04 - val_loss: 8.6169e-04
Epoch 23/50
147/147 ━━━━━━━━━━━━━━━━━━━━ 7s 46ms/step - loss: 8.3620e-04 - val_loss: 8.6539e-04
Epoch 24/50
147/147 ━━━━━━━━━━━━━━━━━━━━ 5s 34ms/step - loss: 8.2085e-04 - val_loss: 8.3722e-04
Epoch 25/50
147/147 ━━━━━━━━━━━━━━━━━━━━ 7s 47ms/step - loss: 7.7866e-04 - val_loss: 8.9533e-04
Epoch 26/50
[illegible]
```

```
147/147 ━━━━━━━━━━━━━━━━━━━━ 8s 33ms/step - loss: 7.7312e-04 - val_loss: 8.2656e-04
Epoch 27/50
147/147 ━━━━━━━━━━━━━━━━━━━━ 7s 46ms/step - loss: 8.6454e-04 - val_loss: 8.5830e-04
Epoch 28/50
147/147 ━━━━━━━━━━━━━━━━━━━━ 9s 35ms/step - loss: 7.4203e-04 - val_loss: 9.7388e-04
Epoch 29/50
147/147 ━━━━━━━━━━━━━━━━━━━━ 10s 35ms/step - loss: 7.2556e-04 - val_loss: 8.9812e-04
Epoch 30/50
147/147 ━━━━━━━━━━━━━━━━━━━━ 7s 47ms/step - loss: 7.2285e-04 - val_loss: 7.8642e-04
Epoch 31/50
147/147 ━━━━━━━━━━━━━━━━━━━━ 5s 33ms/step - loss: 7.0638e-04 - val_loss: 8.1145e-04
Epoch 32/50
147/147 ━━━━━━━━━━━━━━━━━━━━ 6s 41ms/step - loss: 6.6287e-04 - val_loss: 7.9750e-04
Epoch 33/50
147/147 ━━━━━━━━━━━━━━━━━━━━ 9s 34ms/step - loss: 6.8237e-04 - val_loss: 7.6142e-04
Epoch 34/50
147/147 ━━━━━━━━━━━━━━━━━━━━ 7s 45ms/step - loss: 6.6609e-04 - val_loss: 7.5366e-04
Epoch 35/50
147/147 ━━━━━━━━━━━━━━━━━━━━ 5s 35ms/step - loss: 6.0920e-04 - val_loss: 7.5124e-04
Epoch 36/50
147/147 ━━━━━━━━━━━━━━━━━━━━ 10s 34ms/step - loss: 6.1470e-04 - val_loss: 7.4699e-04
Epoch 37/50
147/147 ━━━━━━━━━━━━━━━━━━━━ 5s 33ms/step - loss: 5.9748e-04 - val_loss: 7.4161e-04
Epoch 38/50
147/147 ━━━━━━━━━━━━━━━━━━━━ 7s 44ms/step - loss: 6.4519e-04 - val_loss: 7.1349e-04
Epoch 39/50
147/147 ━━━━━━━━━━━━━━━━━━━━ 5s 34ms/step - loss: 5.7668e-04 - val_loss: 6.9356e-04
Epoch 40/50
147/147 ━━━━━━━━━━━━━━━━━━━━ 7s 47ms/step - loss: 5.6499e-04 - val_loss: 6.6103e-04
Epoch 41/50
147/147 ━━━━━━━━━━━━━━━━━━━━ 9s 36ms/step - loss: 5.5628e-04 - val_loss: 6.4884e-04
Epoch 42/50
147/147 ━━━━━━━━━━━━━━━━━━━━ 10s 35ms/step - loss: 6.0216e-04 - val_loss: 6.6529e-04
Epoch 43/50
147/147 ━━━━━━━━━━━━━━━━━━━━ 7s 45ms/step - loss: 6.3054e-04 - val_loss: 6.7282e-04
Epoch 44/50
147/147 ━━━━━━━━━━━━━━━━━━━━ 5s 33ms/step - loss: 5.8521e-04 - val_loss: 6.9031e-04
Epoch 45/50
147/147 ━━━━━━━━━━━━━━━━━━━━ 5s 33ms/step - loss: 5.3226e-04 - val_loss: 8.6865e-04
Epoch 46/50
147/147 ━━━━━━━━━━━━━━━━━━━━ 7s 44ms/step - loss: 6.3202e-04 - val_loss: 6.2127e-04
Epoch 47/50
147/147 ━━━━━━━━━━━━━━━━━━━━ 11s 47ms/step - loss: 5.2907e-04 - val_loss: 6.5820e-04
Epoch 48/50
147/147 ━━━━━━━━━━━━━━━━━━━━ 8s 34ms/step - loss: 5.5914e-04 - val_loss: 6.2870e-04
Epoch 49/50
147/147 ━━━━━━━━━━━━━━━━━━━━ 7s 45ms/step - loss: 4.7730e-04 - val_loss: 6.8123e-04
Epoch 50/50
147/147 ━━━━━━━━━━━━━━━━━━━━ 5s 35ms/step - loss: 5.3003e-04 - val_loss: 6.3233e-04
74/74 ━━━━━━━━━━━━━━━━━━━━ 2s 17ms/step
```

```
74/74 ━━━━━━━━━━━━━━━━━━━━ 2s 17ms/step
```

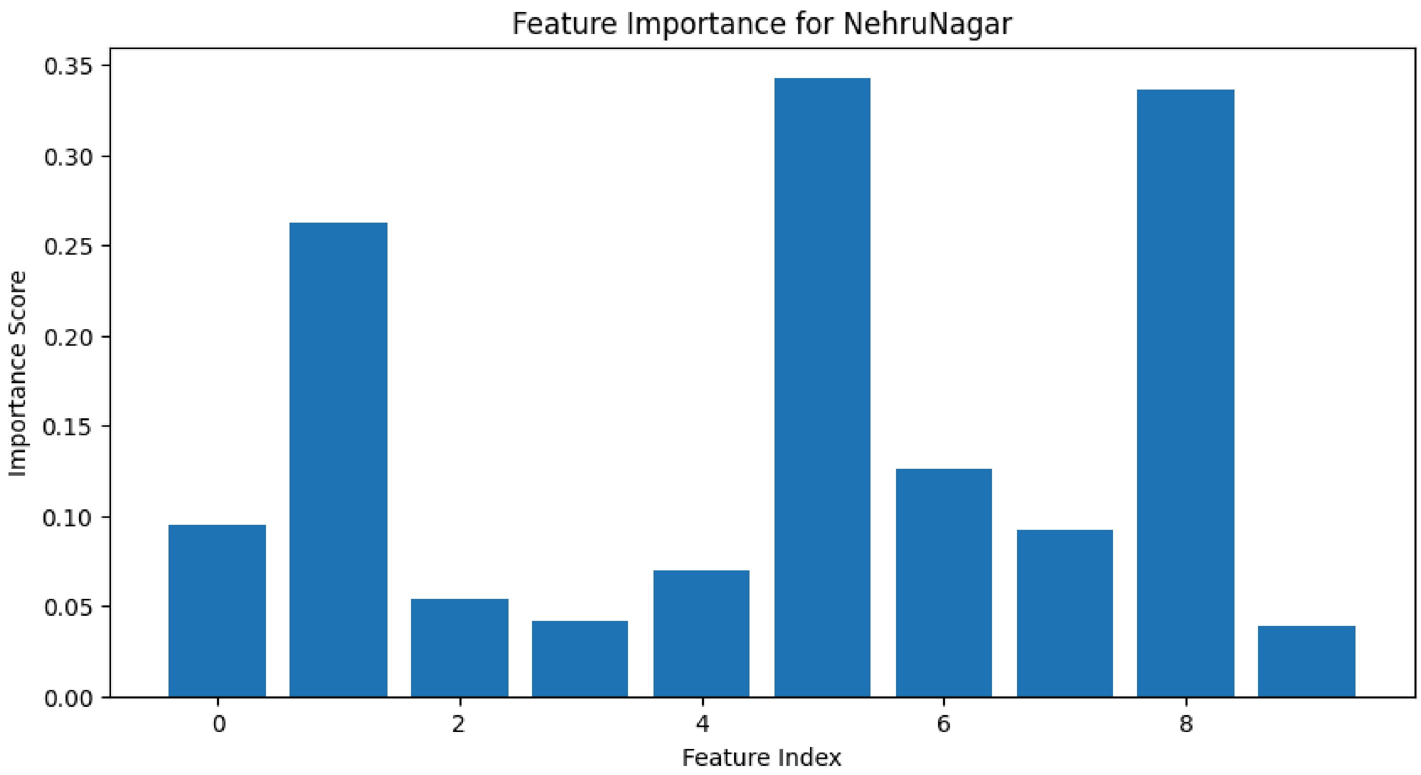

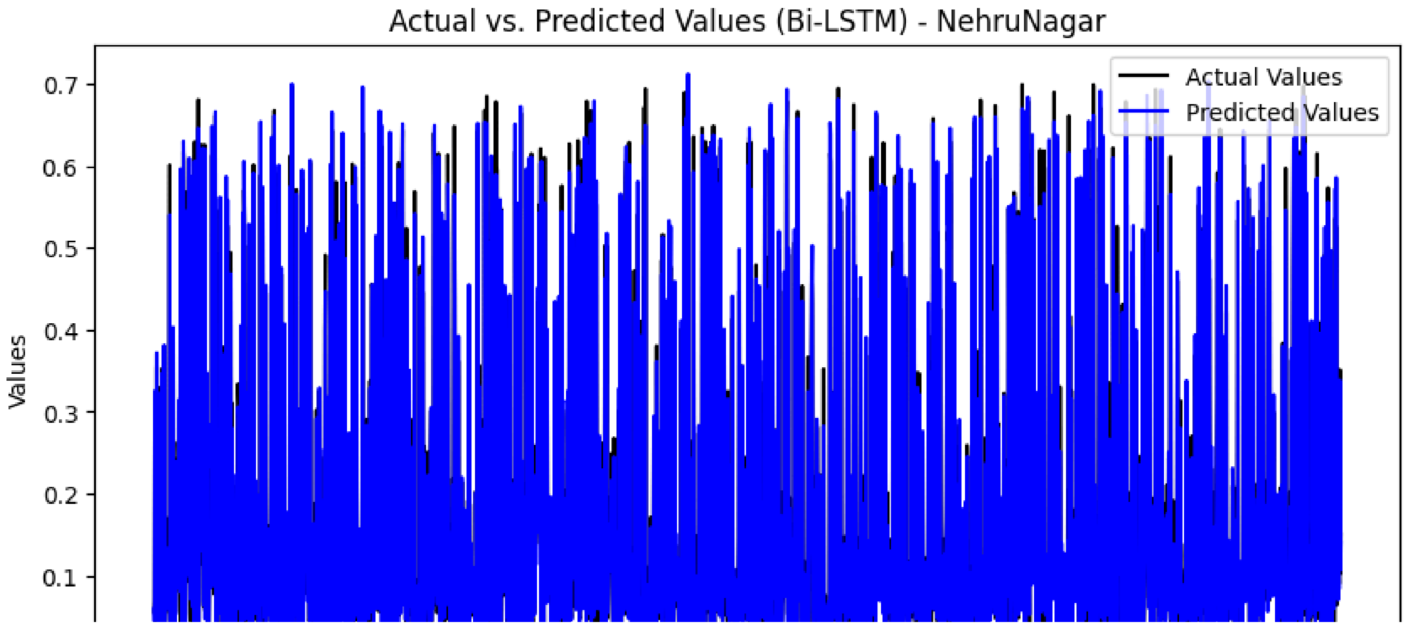

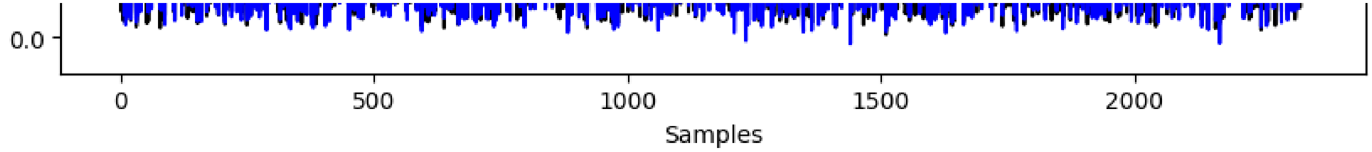


```
Processing Station: Najafgarh
/usr/local/lib/python3.11/dist-packages/pywt/_multilevel.py:43: UserWarning: Level value of 3 is too high: all coefficients will experience boundary effe
  warnings.warn(
Epoch 1/50
/usr/local/lib/python3.11/dist-packages/keras/src/layers/rnn/rnn.py:200: UserWarning: Do not pass an `input_shape`/`input_dim` argument to a layer. When
  super().__init__(**kwargs)
147/147 ━━━━━━━━━━━━━━━━━━━━ 15s 47ms/step - loss: 0.0029 - val_loss: 0.0019
Epoch 2/50
147/147 ━━━━━━━━━━━━━━━━━━━━ 5s 36ms/step - loss: 0.0022 - val_loss: 0.0015
Epoch 3/50
147/147 ━━━━━━━━━━━━━━━━━━━━ 7s 46ms/step - loss: 0.0018 - val_loss: 0.0013
Epoch 4/50
147/147 ━━━━━━━━━━━━━━━━━━━━ 10s 41ms/step - loss: 0.0013 - val_loss: 0.0012
Epoch 5/50
147/147 ━━━━━━━━━━━━━━━━━━━━ 6s 39ms/step - loss: 0.0015 - val_loss: 0.0012
Epoch 6/50
147/147 ━━━━━━━━━━━━━━━━━━━━ 5s 34ms/step - loss: 0.0012 - val_loss: 0.0012
Epoch 7/50
147/147 ━━━━━━━━━━━━━━━━━━━━ 7s 48ms/step - loss: 0.0012 - val_loss: 0.0012
Epoch 8/50
147/147 ━━━━━━━━━━━━━━━━━━━━ 5s 36ms/step - loss: 0.0011 - val_loss: 0.0011
Epoch 9/50
147/147 ━━━━━━━━━━━━━━━━━━━━ 10s 36ms/step - loss: 0.0011 - val_loss: 9.9777e-04
Epoch 10/50
147/147 ━━━━━━━━━━━━━━━━━━━━ 10s 37ms/step - loss: 0.0010 - val_loss: 9.2752e-04
Epoch 11/50
147/147 ━━━━━━━━━━━━━━━━━━━━ 5s 36ms/step - loss: 0.0010 - val_loss: 8.6163e-04
Epoch 12/50
147/147 ━━━━━━━━━━━━━━━━━━━━ 7s 46ms/step - loss: 9.6905e-04 - val_loss: 9.9758e-04
Epoch 13/50
147/147 ━━━━━━━━━━━━━━━━━━━━ 9s 37ms/step - loss: 8.6951e-04 - val_loss: 8.2360e-04
Epoch 14/50
147/147 ━━━━━━━━━━━━━━━━━━━━ 6s 41ms/step - loss: 8.2304e-04 - val_loss: 8.4595e-04
Epoch 15/50
147/147 ━━━━━━━━━━━━━━━━━━━━ 5s 36ms/step - loss: 9.5744e-04 - val_loss: 7.3680e-04
Epoch 16/50
147/147 ━━━━━━━━━━━━━━━━━━━━ 10s 34ms/step - loss: 6.7283e-04 - val_loss: 7.6590e-04
Epoch 17/50
147/147 ━━━━━━━━━━━━━━━━━━━━ 6s 43ms/step - loss: 7.2395e-04 - val_loss: 6.7877e-04
Epoch 18/50
147/147 ━━━━━━━━━━━━━━━━━━━━ 9s 35ms/step - loss: 6.5925e-04 - val_loss: 6.9564e-04
Epoch 19/50
147/147 ━━━━━━━━━━━━━━━━━━━━ 7s 45ms/step - loss: 7.3403e-04 - val_loss: 6.1876e-04
```

```
[illegible]
Epoch 20/50
147/147 ━━━━━━━━━━━━━━━━━━━━ 5s 34ms/step - loss: 6.1898e-04 - val_loss: 6.0344e-04
Epoch 21/50
147/147 ━━━━━━━━━━━━━━━━━━━━ 6s 39ms/step - loss: 6.0869e-04 - val_loss: 5.4940e-04
Epoch 22/50
147/147 ━━━━━━━━━━━━━━━━━━━━ 6s 40ms/step - loss: 5.4598e-04 - val_loss: 5.4777e-04
Epoch 23/50
147/147 ━━━━━━━━━━━━━━━━━━━━ 5s 37ms/step - loss: 5.2884e-04 - val_loss: 5.4755e-04
Epoch 24/50
147/147 ━━━━━━━━━━━━━━━━━━━━ 7s 47ms/step - loss: 5.4936e-04 - val_loss: 5.2352e-04
Epoch 25/50
147/147 ━━━━━━━━━━━━━━━━━━━━ 5s 34ms/step - loss: 5.4023e-04 - val_loss: 5.1735e-04
Epoch 26/50
147/147 ━━━━━━━━━━━━━━━━━━━━ 7s 45ms/step - loss: 5.2893e-04 - val_loss: 5.1442e-04
Epoch 27/50
147/147 ━━━━━━━━━━━━━━━━━━━━ 5s 36ms/step - loss: 4.8854e-04 - val_loss: 5.0643e-04
Epoch 28/50
147/147 ━━━━━━━━━━━━━━━━━━━━ 5s 36ms/step - loss: 4.6059e-04 - val_loss: 5.0542e-04
Epoch 29/50
147/147 ━━━━━━━━━━━━━━━━━━━━ 6s 43ms/step - loss: 5.6126e-04 - val_loss: 4.7498e-04
Epoch 30/50
147/147 ━━━━━━━━━━━━━━━━━━━━ 5s 34ms/step - loss: 4.8768e-04 - val_loss: 5.5842e-04
Epoch 31/50
147/147 ━━━━━━━━━━━━━━━━━━━━ 7s 48ms/step - loss: 4.6895e-04 - val_loss: 4.4071e-04
Epoch 32/50
147/147 ━━━━━━━━━━━━━━━━━━━━ 5s 36ms/step - loss: 4.3095e-04 - val_loss: 4.0107e-04
Epoch 33/50
147/147 ━━━━━━━━━━━━━━━━━━━━ 6s 42ms/step - loss: 4.2761e-04 - val_loss: 4.6968e-04
Epoch 34/50
147/147 ━━━━━━━━━━━━━━━━━━━━ 6s 37ms/step - loss: 4.2248e-04 - val_loss: 4.1968e-04
Epoch 35/50
147/147 ━━━━━━━━━━━━━━━━━━━━ 5s 34ms/step - loss: 4.1849e-04 - val_loss: 3.9630e-04
Epoch 36/50
147/147 ━━━━━━━━━━━━━━━━━━━━ 7s 45ms/step - loss: 4.5261e-04 - val_loss: 4.0514e-04
Epoch 37/50
147/147 ━━━━━━━━━━━━━━━━━━━━ 5s 34ms/step - loss: 4.0228e-04 - val_loss: 3.9720e-04
Epoch 38/50
147/147 ━━━━━━━━━━━━━━━━━━━━ 7s 44ms/step - loss: 3.8436e-04 - val_loss: 3.7480e-04
Epoch 39/50
147/147 ━━━━━━━━━━━━━━━━━━━━ 9s 36ms/step - loss: 3.7660e-04 - val_loss: 3.6007e-04
Epoch 40/50
147/147 ━━━━━━━━━━━━━━━━━━━━ 7s 48ms/step - loss: 3.7046e-04 - val_loss: 3.8506e-04
Epoch 41/50
147/147 ━━━━━━━━━━━━━━━━━━━━ 5s 35ms/step - loss: 3.5033e-04 - val_loss: 3.5720e-04
Epoch 42/50
147/147 ━━━━━━━━━━━━━━━━━━━━ 7s 45ms/step - loss: 3.5378e-04 - val_loss: 3.8261e-04
Epoch 43/50
147/147 ━━━━━━━━━━━━━━━━━━━━ 5s 35ms/step - loss: 3.5843e-04 - val_loss: 3.4474e-04
Epoch 44/50
```

```
147/147 ━━━━━━━━━━━━━━━━━━━━ 5s 36ms/step - loss: 3.2770e-04 - val_loss: 3.5324e-04
Epoch 45/50
147/147 ━━━━━━━━━━━━━━━━━━━━ 7s 47ms/step - loss: 3.4329e-04 - val_loss: 3.4437e-04
Epoch 46/50
147/147 ━━━━━━━━━━━━━━━━━━━━ 9s 42ms/step - loss: 3.1828e-04 - val_loss: 3.4021e-04
Epoch 47/50
147/147 ━━━━━━━━━━━━━━━━━━━━ 6s 41ms/step - loss: 3.3438e-04 - val_loss: 3.8315e-04
Epoch 48/50
147/147 ━━━━━━━━━━━━━━━━━━━━ 5s 34ms/step - loss: 3.2427e-04 - val_loss: 3.3257e-04
Epoch 49/50
147/147 ━━━━━━━━━━━━━━━━━━━━ 7s 48ms/step - loss: 3.3174e-04 - val_loss: 3.4336e-04
Epoch 50/50
147/147 ━━━━━━━━━━━━━━━━━━━━ 5s 36ms/step - loss: 3.2142e-04 - val_loss: 3.4186e-04
74/74 ━━━━━━━━━━━━━━━━━━━━ 6s 26ms/step
```

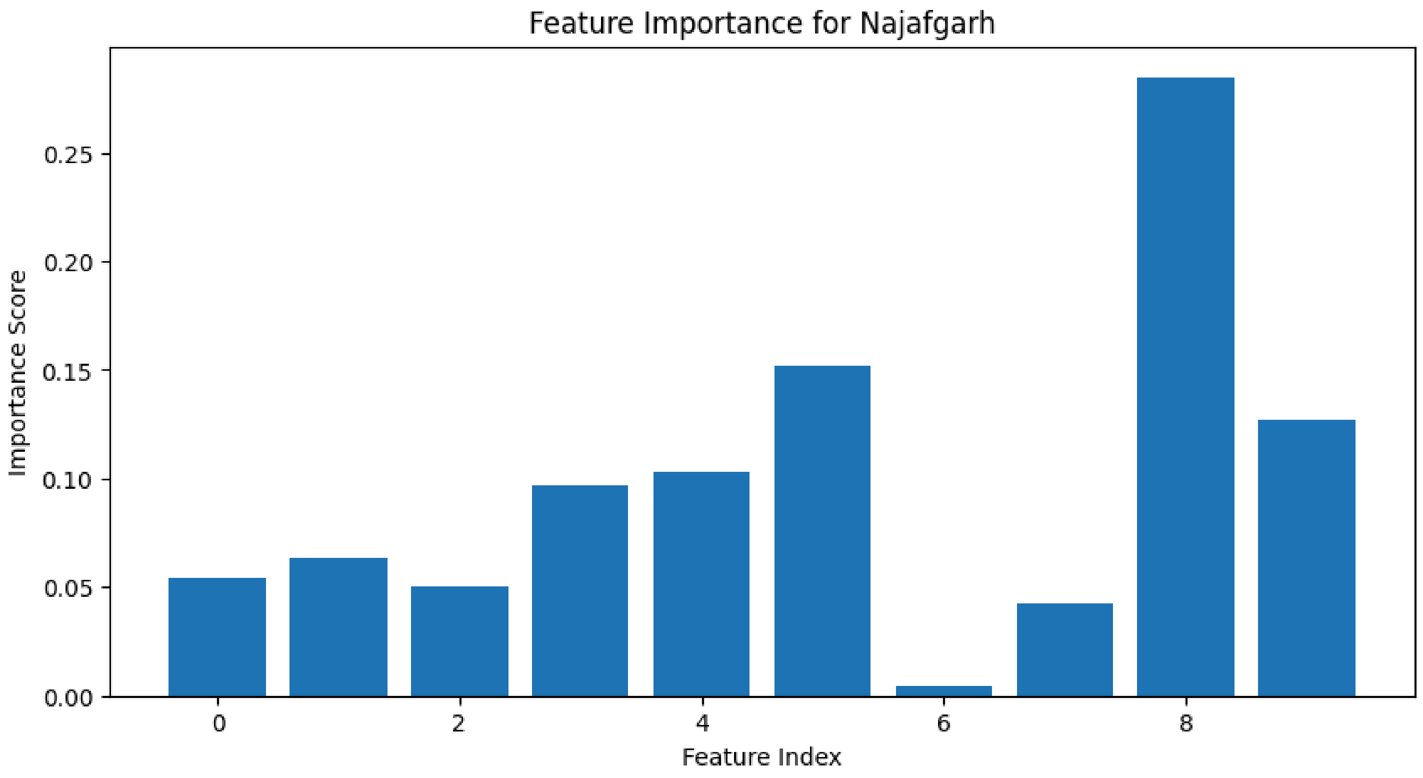

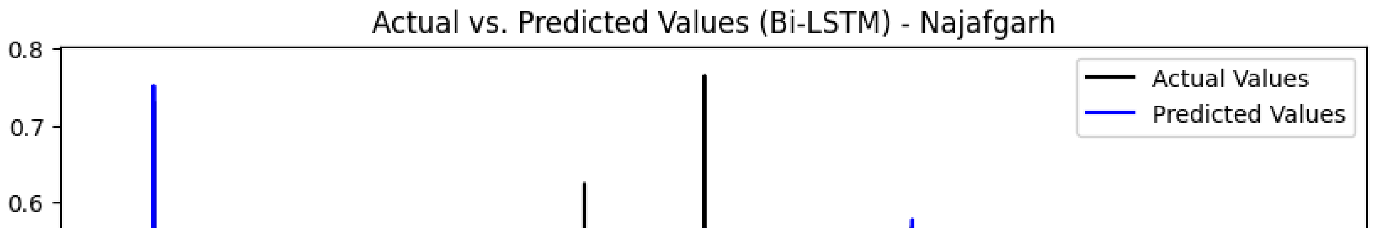

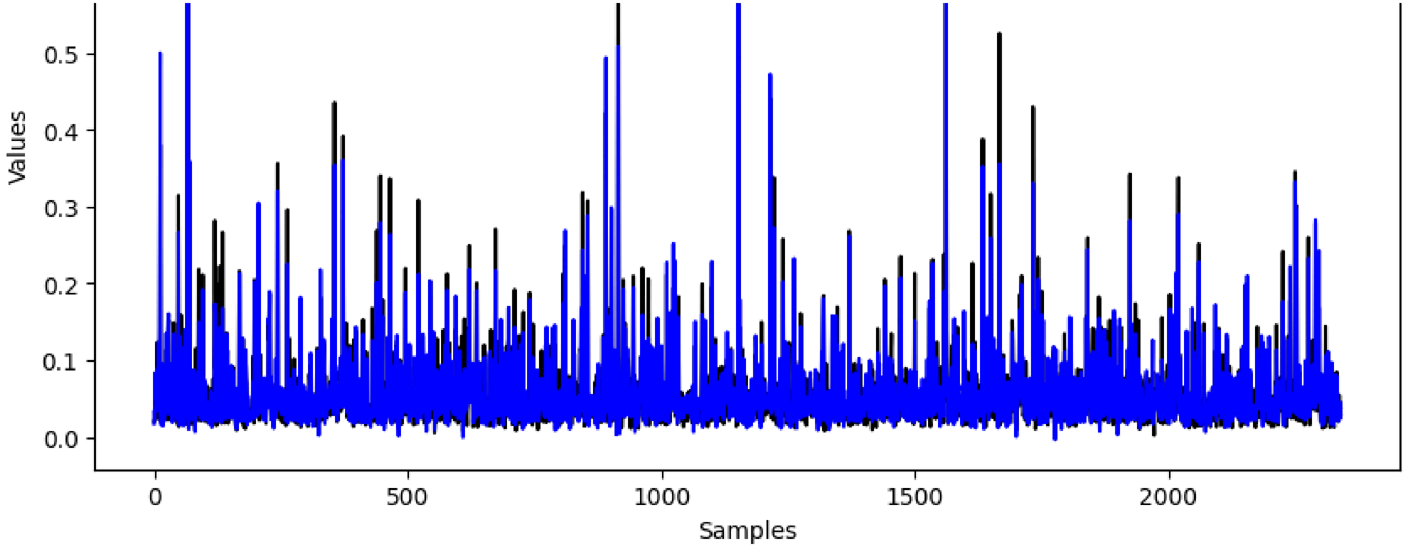


```
Processing Station: Okhla
/usr/local/lib/python3.11/dist-packages/pywt/_multilevel.py:43: UserWarning: Level value of 3 is too high: all coefficients will experience boundary effe
  warnings.warn(
Epoch 1/50
/usr/local/lib/python3.11/dist-packages/keras/src/layers/rnn/rnn.py:200: UserWarning: Do not pass an `input_shape`/`input_dim` argument to a layer. When
  super().__init__(**kwargs)
147/147 ━━━━━━━━━━━━━━━━━━━━ 16s 44ms/step - loss: 0.0108 - val_loss: 0.0068
Epoch 2/50
147/147 ━━━━━━━━━━━━━━━━━━━━ 9s 38ms/step - loss: 0.0057 - val_loss: 0.0046
Epoch 3/50
147/147 ━━━━━━━━━━━━━━━━━━━━ 8s 52ms/step - loss: 0.0037 - val_loss: 0.0028
Epoch 4/50
147/147 ━━━━━━━━━━━━━━━━━━━━ 6s 39ms/step - loss: 0.0025 - val_loss: 0.0025
Epoch 5/50
147/147 ━━━━━━━━━━━━━━━━━━━━ 7s 49ms/step - loss: 0.0021 - val_loss: 0.0018
Epoch 6/50
147/147 ━━━━━━━━━━━━━━━━━━━━ 9s 38ms/step - loss: 0.0018 - val_loss: 0.0021
Epoch 7/50
147/147 ━━━━━━━━━━━━━━━━━━━━ 8s 53ms/step - loss: 0.0019 - val_loss: 0.0018
Epoch 8/50
147/147 ━━━━━━━━━━━━━━━━━━━━ 8s 39ms/step - loss: 0.0015 - val_loss: 0.0013
Epoch 9/50
147/147 ━━━━━━━━━━━━━━━━━━━━ 10s 39ms/step - loss: 0.0016 - val_loss: 0.0013
Epoch 10/50
147/147 ━━━━━━━━━━━━━━━━━━━━ 10s 37ms/step - loss: 0.0014 - val_loss: 0.0012
Epoch 11/50
147/147 ━━━━━━━━━━━━━━━━━━━━ 11s 41ms/step - loss: 0.0012 - val_loss: 0.0011
Epoch 12/50
147/147 ━━━━━━━━━━━━━━━━━━━━ 6s 40ms/step - loss: 0.0013 - val_loss: 0.0012
```

```
Epoch 13/50
147/147 ━━━━━━━━━━━━━━━━━━━━ 7s 48ms/step - loss: 0.0011 - val_loss: 9.5171e-04
Epoch 14/50
147/147 ━━━━━━━━━━━━━━━━━━━━ 6s 39ms/step - loss: 0.0011 - val_loss: 9.2502e-04
Epoch 15/50
147/147 ━━━━━━━━━━━━━━━━━━━━ 7s 50ms/step - loss: 9.7000e-04 - val_loss: 0.0010
Epoch 16/50
147/147 ━━━━━━━━━━━━━━━━━━━━ 6s 39ms/step - loss: 9.4814e-04 - val_loss: 0.0011
Epoch 17/50
147/147 ━━━━━━━━━━━━━━━━━━━━ 10s 37ms/step - loss: 9.4511e-04 - val_loss: 8.2109e-04
Epoch 18/50
147/147 ━━━━━━━━━━━━━━━━━━━━ 7s 49ms/step - loss: 8.6321e-04 - val_loss: 8.2673e-04
Epoch 19/50
147/147 ━━━━━━━━━━━━━━━━━━━━ 6s 38ms/step - loss: 8.1662e-04 - val_loss: 8.2687e-04
Epoch 20/50
147/147 ━━━━━━━━━━━━━━━━━━━━ 7s 49ms/step - loss: 8.6642e-04 - val_loss: 7.5244e-04
Epoch 21/50
147/147 ━━━━━━━━━━━━━━━━━━━━ 9s 38ms/step - loss: 8.2573e-04 - val_loss: 7.3961e-04
Epoch 22/50
147/147 ━━━━━━━━━━━━━━━━━━━━ 7s 51ms/step - loss: 8.1306e-04 - val_loss: 7.1818e-04
Epoch 23/50
147/147 ━━━━━━━━━━━━━━━━━━━━ 6s 39ms/step - loss: 8.2577e-04 - val_loss: 7.9732e-04
Epoch 24/50
147/147 ━━━━━━━━━━━━━━━━━━━━ 7s 51ms/step - loss: 7.5850e-04 - val_loss: 7.1942e-04
Epoch 25/50
147/147 ━━━━━━━━━━━━━━━━━━━━ 6s 38ms/step - loss: 7.0400e-04 - val_loss: 7.4815e-04
Epoch 26/50
147/147 ━━━━━━━━━━━━━━━━━━━━ 11s 45ms/step - loss: 7.0469e-04 - val_loss: 7.3601e-04
Epoch 27/50
147/147 ━━━━━━━━━━━━━━━━━━━━ 6s 39ms/step - loss: 7.4223e-04 - val_loss: 7.6252e-04
Epoch 28/50
147/147 ━━━━━━━━━━━━━━━━━━━━ 10s 39ms/step - loss: 6.7342e-04 - val_loss: 6.7618e-04
Epoch 29/50
147/147 ━━━━━━━━━━━━━━━━━━━━ 8s 53ms/step - loss: 6.0580e-04 - val_loss: 6.9458e-04
Epoch 30/50
147/147 ━━━━━━━━━━━━━━━━━━━━ 6s 38ms/step - loss: 6.3690e-04 - val_loss: 6.7546e-04
Epoch 31/50
147/147 ━━━━━━━━━━━━━━━━━━━━ 8s 51ms/step - loss: 6.2195e-04 - val_loss: 7.6841e-04
Epoch 32/50
147/147 ━━━━━━━━━━━━━━━━━━━━ 6s 39ms/step - loss: 6.4256e-04 - val_loss: 6.4388e-04
Epoch 33/50
147/147 ━━━━━━━━━━━━━━━━━━━━ 10s 40ms/step - loss: 5.7940e-04 - val_loss: 6.5919e-04
Epoch 34/50
147/147 ━━━━━━━━━━━━━━━━━━━━ 12s 50ms/step - loss: 5.8002e-04 - val_loss: 6.7866e-04
Epoch 35/50
147/147 ━━━━━━━━━━━━━━━━━━━━ 6s 37ms/step - loss: 6.2093e-04 - val_loss: 6.4414e-04
Epoch 36/50
147/147 ━━━━━━━━━━━━━━━━━━━━ 8s 52ms/step - loss: 5.8560e-04 - val_loss: 6.4933e-04
Epoch 37/50
[illegible]
```

```
147/147 ━━━━━━━━━━━━━━━━━━━━ 8s 39ms/step - loss: 6.1106e-04 - val_loss: 6.6578e-04
Epoch 38/50
147/147 ━━━━━━━━━━━━━━━━━━━━ 8s 54ms/step - loss: 5.9190e-04 - val_loss: 6.0490e-04
Epoch 39/50
147/147 ━━━━━━━━━━━━━━━━━━━━ 9s 42ms/step - loss: 5.4838e-04 - val_loss: 6.1044e-04
Epoch 40/50
147/147 ━━━━━━━━━━━━━━━━━━━━ 7s 45ms/step - loss: 5.1985e-04 - val_loss: 5.7928e-04
Epoch 41/50
147/147 ━━━━━━━━━━━━━━━━━━━━ 6s 40ms/step - loss: 5.3893e-04 - val_loss: 6.1422e-04
Epoch 42/50
147/147 ━━━━━━━━━━━━━━━━━━━━ 8s 52ms/step - loss: 5.6835e-04 - val_loss: 6.3304e-04
Epoch 43/50
147/147 ━━━━━━━━━━━━━━━━━━━━ 6s 38ms/step - loss: 4.9934e-04 - val_loss: 7.2319e-04
Epoch 44/50
147/147 ━━━━━━━━━━━━━━━━━━━━ 7s 50ms/step - loss: 5.6378e-04 - val_loss: 6.9908e-04
Epoch 45/50
147/147 ━━━━━━━━━━━━━━━━━━━━ 9s 40ms/step - loss: 5.0534e-04 - val_loss: 7.2469e-04
Epoch 46/50
147/147 ━━━━━━━━━━━━━━━━━━━━ 10s 39ms/step - loss: 5.1643e-04 - val_loss: 5.6802e-04
Epoch 47/50
147/147 ━━━━━━━━━━━━━━━━━━━━ 10s 39ms/step - loss: 4.6646e-04 - val_loss: 5.8409e-04
Epoch 48/50
147/147 ━━━━━━━━━━━━━━━━━━━━ 7s 50ms/step - loss: 5.1787e-04 - val_loss: 6.2648e-04
Epoch 49/50
147/147 ━━━━━━━━━━━━━━━━━━━━ 9s 39ms/step - loss: 4.8322e-04 - val_loss: 5.7567e-04
Epoch 50/50
147/147 ━━━━━━━━━━━━━━━━━━━━ 10s 38ms/step - loss: 4.3990e-04 - val_loss: 5.8477e-04
74/74 ━━━━━━━━━━━━━━━━━━━━ 2s 19ms/step
```

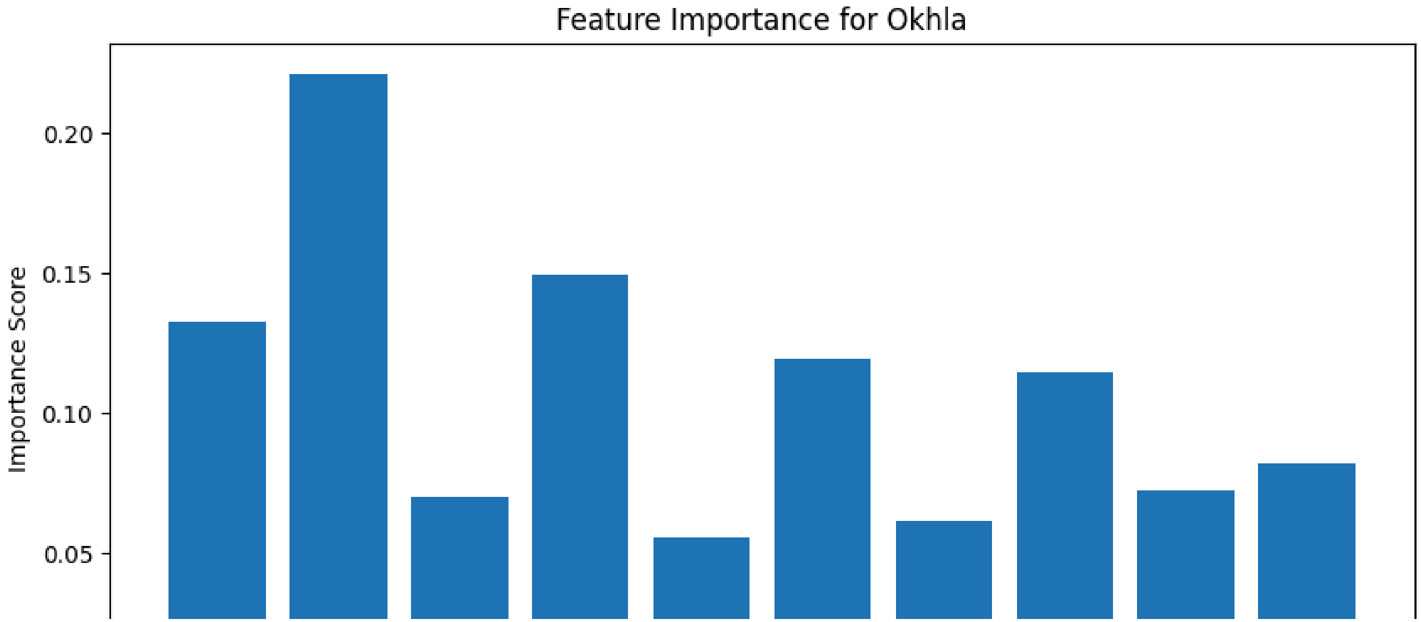

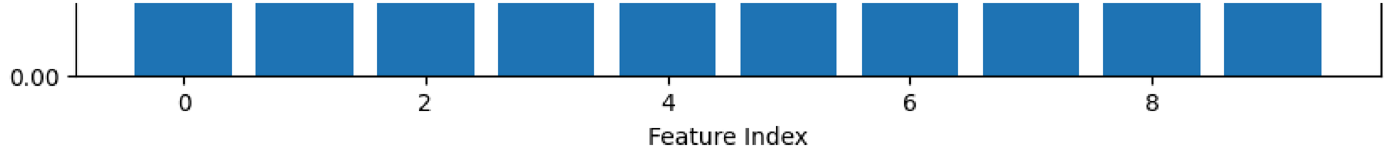

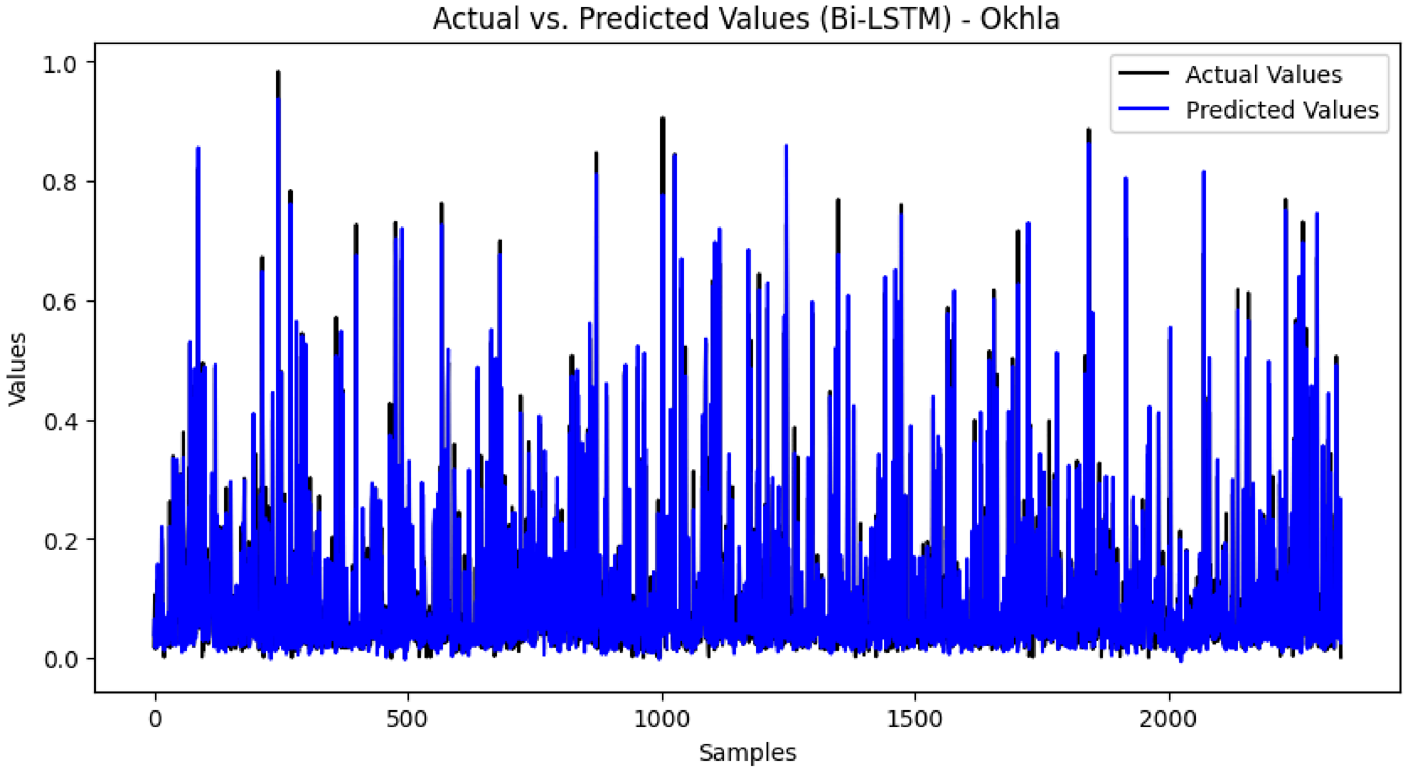


```
Final Model Evaluation Across Stations:

Station: AshokVihar
MSE: 0.0006
MAE: 0.0154
RMSE: 0.0249
R² Score: 0.9430

Station: DCStadium
MSE: 0.0008
MAE: 0.0183
RMSE: 0.0287
R² Score: 0.9838

Station: DwarkaSec8
MSE: 0.0007
```

```
MSE: 0.0007
MAE: 0.0178
RMSE: 0.0269
R² Score: 0.9425

Station: NehruNagar
MSE: 0.0006
MAE: 0.0181
RMSE: 0.0251
R² Score: 0.9796

Station: Najafgarh
MSE: 0.0003
MAE: 0.0119
RMSE: 0.0185
R² Score: 0.8958

Station: Okhla
MSE: 0.0006
MAE: 0.0154
RMSE: 0.0242
R² Score: 0.9653
```

Double-click (or enter) to edit

```
!pip install PyWavelets
```

```
Collecting PyWavelets
  Downloading pywavelets-1.8.0-cp311-cp311-manylinux_2_17_x86_64.manylinux2014_x86_64.whl.metadata (9.0 kB)
Requirement already satisfied: numpy<3,>=1.23 in /usr/local/lib/python3.11/dist-packages (from PyWavelets) (2.0.2)
Downloading pywavelets-1.8.0-cp311-cp311-manylinux_2_17_x86_64.manylinux2014_x86_64.whl (4.5 MB)
   ━━━━━━━━━━━━━━━━━━━━━━━━━━━━━━━━━━━━━━━━ 4.5/4.5 MB 78.0 MB/s eta 0:00:00
Installing collected packages: PyWavelets
Successfully installed PyWavelets-1.8.0
```

AquilaOptimizer

```
import numpy as np
import pandas as pd
import matplotlib.pyplot as plt
from sklearn.decomposition import PCA
from sklearn.preprocessing import StandardScaler, MinMaxScaler
from sklearn.model_selection import train_test_split
from sklearn.metrics import mean_squared_error, mean_absolute_error, r2_score
import tensorflow as tf
from tensorflow.keras.models import Sequential
from tensorflow.keras.layers import LSTM, Dense, Bidirectional
import pywt

# ============================
# Aquila Optimizer
# ============================
class AquilaOptimizer:
    def __init__(self, objective_function, lower_bound, upper_bound, population_size, iterations):
        self.objective_function = objective_function
        self.lower_bound = np.array(lower_bound)
        self.upper_bound = np.array(upper_bound)
        self.population_size = population_size
        self.iterations = iterations
        self.dimension = len(lower_bound)
        self.population = np.random.uniform(self.lower_bound, self.upper_bound, (self.population_size, self.dimension))
        self.best_solution = None
        self.best_fitness = float('inf')
```

```
    def optimize(self):
        for t in range(self.iterations):
            for i in range(self.population_size):
                alpha = 0.1  # exploration factor
                r = np.random.uniform(-1, 1, self.dimension)
                candidate = self.population[i] + alpha * r * (self.best_solution - self.population[i]) if self.best_solution is not None else self.population
                candidate = np.clip(candidate, self.lower_bound, self.upper_bound)
                fitness = self.objective_function(candidate)

                if fitness < self.best_fitness:
                    self.best_fitness = fitness
                    self.best_solution = candidate

        return self.best_solution

# ============================
# Feature Extraction
# ============================
def extract_wavelet_features(X, wavelet='db4', level=3, num_features=50):
    features = []
    for sample in X:
        coeffs = pywt.wavedec(sample, wavelet, level=level)
        flattened_coeffs = np.concatenate([c.flatten() for c in coeffs])
        features.append(flattened_coeffs[:num_features])
    return np.array(features)

def apply_pca(X, n_components=10):
    scaler = StandardScaler()
    X_scaled = scaler.fit_transform(X)
    pca = PCA(n_components=n_components)
    return pca.fit_transform(X_scaled)

def extract_combined_features(X):
    X_wavelet = extract_wavelet_features(X)
    X_pca = apply_pca(X_wavelet)
    return X_pca

# ============================
# Bi-LSTM Model Definition
# ============================
def build_lstm_model(input_shape):
    model = Sequential([
        Bidirectional(LSTM(50, return_sequences=True, input_shape=input_shape)),
```

```
        Bidirectional(LSTM(50, return_sequences=False)),
        Dense(1)
    ])
    model.compile(optimizer='adam', loss='mean_squared_error')
    return model

# ============================
# Training & Evaluation
# ============================
def evaluate_model(X, y):
    if X.shape[1] == 0:
        raise ValueError("No features selected! Adjust Aquila feature selection.")
    X_train, X_test, y_train, y_test = train_test_split(X, y, test_size=0.2, random_state=42)
    X_train = X_train.reshape(X_train.shape[0], X_train.shape[1], 1)
    X_test = X_test.reshape(X_test.shape[0], X_test.shape[1], 1)

    model = build_lstm_model((X_train.shape[1], 1))
    model.fit(X_train, y_train, epochs=50, batch_size=64, validation_data=(X_test, y_test), verbose=1)
    y_pred = model.predict(X_test)

    mse = mean_squared_error(y_test, y_pred)
    mae = mean_absolute_error(y_test, y_pred)
    rmse = np.sqrt(mse)
    r2 = r2_score(y_test, y_pred)

    return mse, mae, rmse, r2, y_test, y_pred

# ============================
# Multi-Station Processing
# ============================
stations = {
    'AshokVihar': '/content/AshokVihar_Hourly.csv',
    'DCStadium': '/content/DCStadium_Hourly.csv',
    'DwarkaSec8': '/content/DwarkaSec8_Hourly.csv',
    'NehruNagar': '/content/NehruNagar_Hourly.csv',
    'Najafgarh': '/content/Najafgarh_Hourly.csv',
    'Okhla': '/content/Okhla_Hourly.csv'
}

threshold = 0.40  # Adjust this value as needed
results = {}

for station, file_path in stations.items():
    print(f"\nProcessing Station: {station}")
```

```
    # Load Data
    df = pd.read_csv(file_path)

    # Preprocessing
    scaler = MinMaxScaler()
    X_full = scaler.fit_transform(df.iloc[:, :-1].values)
    y = scaler.fit_transform(df.iloc[:, -1].values.reshape(-1, 1))

    # Feature Extraction
    X_extracted = extract_combined_features(X_full)

    # Feature Selection with Aquila Optimizer
    objective_function = lambda x: np.sum(x**2)
    aquila_optimizer = AquilaOptimizer(objective_function, lower_bound=[-1] * X_extracted.shape[1],
                                       upper_bound=[1] * X_extracted.shape[1],
                                       population_size=50, iterations=100)
    selected_features = aquila_optimizer.optimize()

    # Select features above threshold
    X_selected = X_extracted[:, selected_features > threshold]

    # Final Model Evaluation
    X_final = X_selected if X_selected.shape[1] > 0 else X_extracted
    mse, mae, rmse, r2, y_test, y_pred = evaluate_model(X_final, y)

    # Store results
    results[station] = {"MSE": mse, "MAE": mae, "RMSE": rmse, "R² Score": r2}

    # Plot Feature Importance
    plt.figure(figsize=(10, 5))
    feature_importance = np.abs(selected_features)
    plt.bar(range(len(feature_importance)), feature_importance)
    plt.xlabel('Feature Index')
    plt.ylabel('Importance Score')
    plt.title(f'Feature Importance for {station}')
    plt.show()

    # Plot Actual vs Predicted
    plt.figure(figsize=(10, 5))
    plt.plot(y_test, label="Actual Values", color='black')
    plt.plot(y_pred, label="Predicted Values", color='blue')
    plt.xlabel('Samples')
    plt.ylabel('Values')
```

```
    plt.title(f'Actual vs. Predicted Values Aquila optimizer-(Bi-LSTM) - {station}')
    plt.legend()
    plt.show()

# Print Final Results
print("\nFinal Model Evaluation Across Stations:")
for station, metrics in results.items():
    print(f"\nStation: {station}")
    for metric, value in metrics.items():
        print(f"{metric}: {value:.4f}")
```

```
Processing Station: AshokVihar
/usr/local/lib/python3.11/dist-packages/pywt/_multilevel.py:43: UserWarning: Level value of 3 is too high: all coefficients will experience boundary effe
  warnings.warn(
/usr/local/lib/python3.11/dist-packages/keras/src/layers/rnn/rnn.py:200: UserWarning: Do not pass an `input_shape`/`input_dim` argument to a layer. When
  super().__init__(**kwargs)
Epoch 1/50
147/147 ━━━━━━━━━━━━━━━━━━━━ 13s 15ms/step - loss: 0.0131 - val_loss: 0.0105
Epoch 2/50
147/147 ━━━━━━━━━━━━━━━━━━━━ 2s 9ms/step - loss: 0.0104 - val_loss: 0.0104
Epoch 3/50
147/147 ━━━━━━━━━━━━━━━━━━━━ 1s 9ms/step - loss: 0.0106 - val_loss: 0.0104
Epoch 4/50
147/147 ━━━━━━━━━━━━━━━━━━━━ 3s 10ms/step - loss: 0.0119 - val_loss: 0.0104
Epoch 5/50
147/147 ━━━━━━━━━━━━━━━━━━━━ 1s 9ms/step - loss: 0.0114 - val_loss: 0.0104
Epoch 6/50
147/147 ━━━━━━━━━━━━━━━━━━━━ 3s 9ms/step - loss: 0.0105 - val_loss: 0.0103
Epoch 7/50
147/147 ━━━━━━━━━━━━━━━━━━━━ 3s 9ms/step - loss: 0.0108 - val_loss: 0.0103
Epoch 8/50
147/147 ━━━━━━━━━━━━━━━━━━━━ 1s 9ms/step - loss: 0.0109 - val_loss: 0.0105
Epoch 9/50
147/147 ━━━━━━━━━━━━━━━━━━━━ 1s 10ms/step - loss: 0.0100 - val_loss: 0.0103
Epoch 10/50
147/147 ━━━━━━━━━━━━━━━━━━━━ 2s 13ms/step - loss: 0.0104 - val_loss: 0.0103
Epoch 11/50
147/147 ━━━━━━━━━━━━━━━━━━━━ 2s 9ms/step - loss: 0.0108 - val_loss: 0.0105
Epoch 12/50
147/147 ━━━━━━━━━━━━━━━━━━━━ 1s 10ms/step - loss: 0.0105 - val_loss: 0.0103
Epoch 13/50
147/147 ━━━━━━━━━━━━━━━━━━━━ 2s 9ms/step - loss: 0.0110 - val_loss: 0.0104
Epoch 14/50
147/147 ━━━━━━━━━━━━━━━━━━━━ 3s 9ms/step - loss: 0.0108 - val_loss: 0.0103
Epoch 15/50
147/147 ━━━━━━━━━━━━━━━━━━━━ 3s 12ms/step - loss: 0.0107 - val_loss: 0.0103
Epoch 16/50
147/147 ━━━━━━━━━━━━━━━━━━━━ 2s 9ms/step - loss: 0.0108 - val_loss: 0.0103
Epoch 17/50
147/147 ━━━━━━━━━━━━━━━━━━━━ 1s 9ms/step - loss: 0.0094 - val_loss: 0.0103
Epoch 18/50
147/147 ━━━━━━━━━━━━━━━━━━━━ 1s 9ms/step - loss: 0.0108 - val_loss: 0.0102
Epoch 19/50
147/147 ━━━━━━━━━━━━━━━━━━━━ 3s 9ms/step - loss: 0.0110 - val_loss: 0.0102
Epoch 20/50
147/147 ━━━━━━━━━━━━━━━━━━━━ 1s 9ms/step - loss: 0.0107 - val_loss: 0.0103
Epoch 21/50
147/147 ━━━━━━━━━━━━━━━━━━━━ 3s 10ms/step - loss: 0.0102 - val_loss: 0.0104
Epoch 22/50
```

```
147/147 ━━━━━━━━━━━━━━━━━━━━ 3s 10ms/step - loss: 0.0108 - val_loss: 0.0103
Epoch 23/50
147/147 ━━━━━━━━━━━━━━━━━━━━ 2s 9ms/step - loss: 0.0105 - val_loss: 0.0103
Epoch 24/50
147/147 ━━━━━━━━━━━━━━━━━━━━ 1s 9ms/step - loss: 0.0113 - val_loss: 0.0103
Epoch 25/50
147/147 ━━━━━━━━━━━━━━━━━━━━ 3s 9ms/step - loss: 0.0110 - val_loss: 0.0103
Epoch 26/50
147/147 ━━━━━━━━━━━━━━━━━━━━ 1s 9ms/step - loss: 0.0097 - val_loss: 0.0103
Epoch 27/50
147/147 ━━━━━━━━━━━━━━━━━━━━ 3s 13ms/step - loss: 0.0105 - val_loss: 0.0102
Epoch 28/50
147/147 ━━━━━━━━━━━━━━━━━━━━ 2s 9ms/step - loss: 0.0107 - val_loss: 0.0103
Epoch 29/50
147/147 ━━━━━━━━━━━━━━━━━━━━ 1s 9ms/step - loss: 0.0105 - val_loss: 0.0103
Epoch 30/50
147/147 ━━━━━━━━━━━━━━━━━━━━ 3s 9ms/step - loss: 0.0100 - val_loss: 0.0102
Epoch 31/50
147/147 ━━━━━━━━━━━━━━━━━━━━ 1s 9ms/step - loss: 0.0107 - val_loss: 0.0102
Epoch 32/50
147/147 ━━━━━━━━━━━━━━━━━━━━ 3s 9ms/step - loss: 0.0109 - val_loss: 0.0102
Epoch 33/50
147/147 ━━━━━━━━━━━━━━━━━━━━ 3s 10ms/step - loss: 0.0103 - val_loss: 0.0102
Epoch 34/50
147/147 ━━━━━━━━━━━━━━━━━━━━ 2s 10ms/step - loss: 0.0099 - val_loss: 0.0102
Epoch 35/50
147/147 ━━━━━━━━━━━━━━━━━━━━ 1s 9ms/step - loss: 0.0109 - val_loss: 0.0102
Epoch 36/50
147/147 ━━━━━━━━━━━━━━━━━━━━ 1s 9ms/step - loss: 0.0109 - val_loss: 0.0102
Epoch 37/50
147/147 ━━━━━━━━━━━━━━━━━━━━ 1s 9ms/step - loss: 0.0107 - val_loss: 0.0102
Epoch 38/50
147/147 ━━━━━━━━━━━━━━━━━━━━ 3s 9ms/step - loss: 0.0106 - val_loss: 0.0102
Epoch 39/50
147/147 ━━━━━━━━━━━━━━━━━━━━ 2s 13ms/step - loss: 0.0102 - val_loss: 0.0103
Epoch 40/50
147/147 ━━━━━━━━━━━━━━━━━━━━ 2s 14ms/step - loss: 0.0103 - val_loss: 0.0103
Epoch 41/50
147/147 ━━━━━━━━━━━━━━━━━━━━ 2s 9ms/step - loss: 0.0107 - val_loss: 0.0102
Epoch 42/50
147/147 ━━━━━━━━━━━━━━━━━━━━ 1s 9ms/step - loss: 0.0104 - val_loss: 0.0102
Epoch 43/50
147/147 ━━━━━━━━━━━━━━━━━━━━ 1s 9ms/step - loss: 0.0106 - val_loss: 0.0102
Epoch 44/50
147/147 ━━━━━━━━━━━━━━━━━━━━ 1s 9ms/step - loss: 0.0108 - val_loss: 0.0102
Epoch 45/50
147/147 ━━━━━━━━━━━━━━━━━━━━ 3s 9ms/step - loss: 0.0112 - val_loss: 0.0102
Epoch 46/50
147/147 ━━━━━━━━━━━━━━━━━━━━ 2s 13ms/step - loss: 0.0113 - val_loss: 0.0102
```

```
Epoch 47/50
147/147 ━━━━━━━━━━━━━━━━━━━━ 2s 10ms/step - loss: 0.0103 - val_loss: 0.0102
Epoch 48/50
147/147 ━━━━━━━━━━━━━━━━━━━━ 2s 9ms/step - loss: 0.0104 - val_loss: 0.0102
Epoch 49/50
147/147 ━━━━━━━━━━━━━━━━━━━━ 3s 9ms/step - loss: 0.0102 - val_loss: 0.0104
Epoch 50/50
147/147 ━━━━━━━━━━━━━━━━━━━━ 3s 10ms/step - loss: 0.0104 - val_loss: 0.0102
74/74 ━━━━━━━━━━━━━━━━━━━━ 1s 11ms/step
```

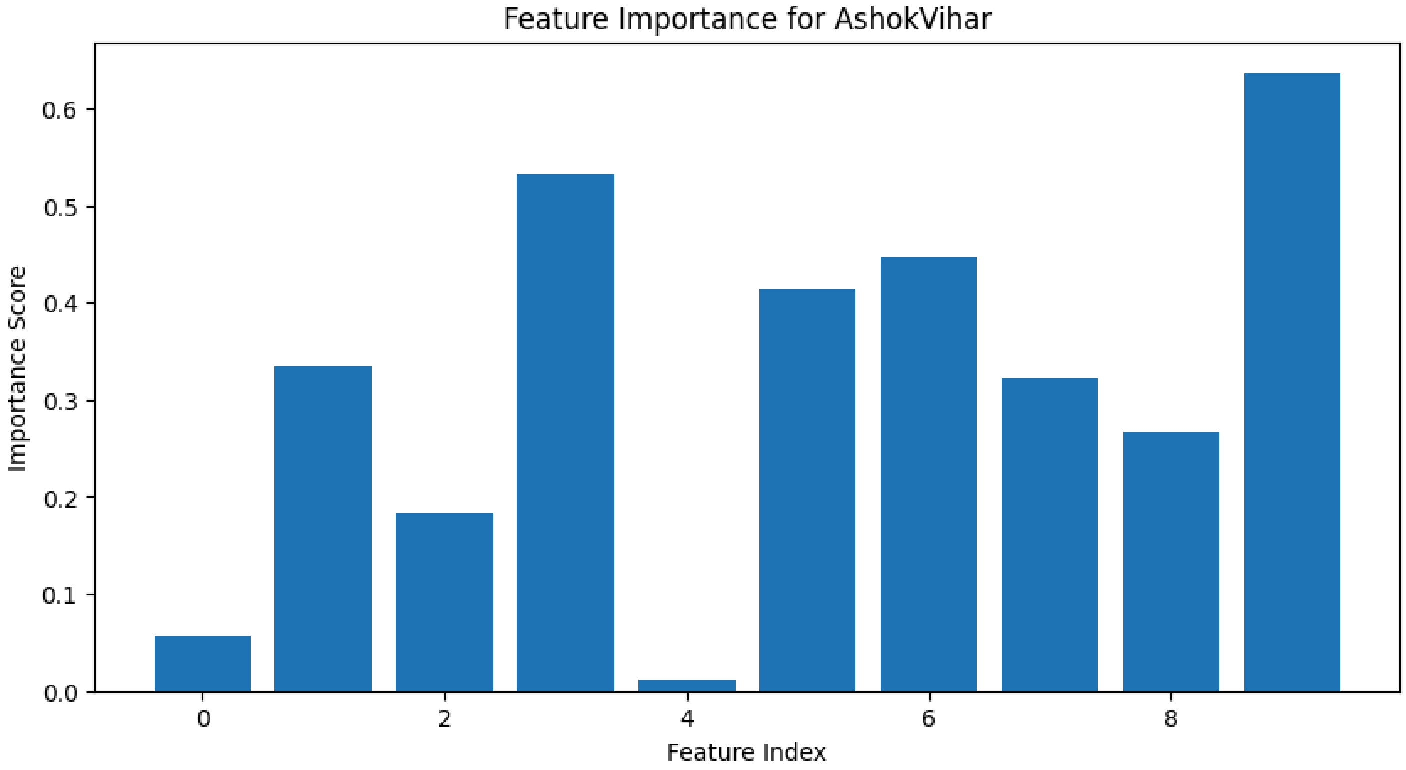

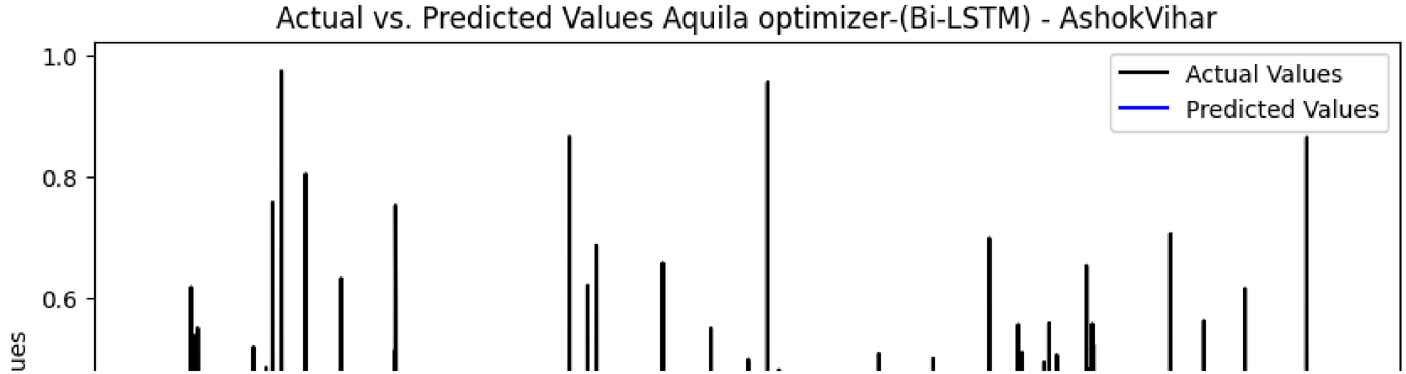

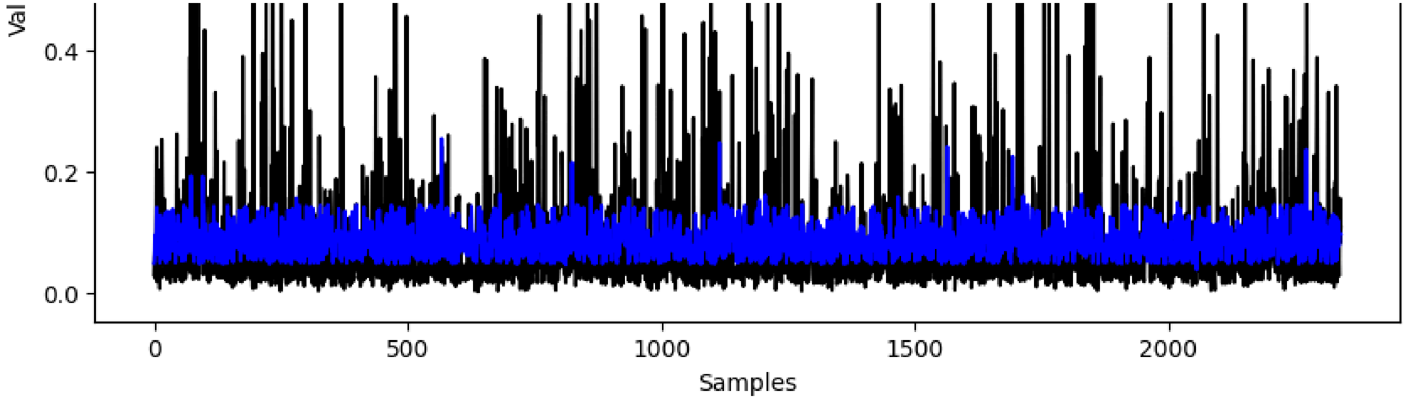


```
Processing Station: DCStadium
/usr/local/lib/python3.11/dist-packages/pywt/_multilevel.py:43: UserWarning: Level value of 3 is too high: all coefficients will experience boundary effe
  warnings.warn(
Epoch 1/50
/usr/local/lib/python3.11/dist-packages/keras/src/layers/rnn/rnn.py:200: UserWarning: Do not pass an `input_shape`/`input_dim` argument to a layer. When
  super().__init__(**kwargs)
147/147 ━━━━━━━━━━━━━━━━━━━━ 6s 13ms/step - loss: 0.0471 - val_loss: 0.0356
Epoch 2/50
147/147 ━━━━━━━━━━━━━━━━━━━━ 2s 9ms/step - loss: 0.0360 - val_loss: 0.0293
Epoch 3/50
147/147 ━━━━━━━━━━━━━━━━━━━━ 3s 10ms/step - loss: 0.0291 - val_loss: 0.0238
Epoch 4/50
147/147 ━━━━━━━━━━━━━━━━━━━━ 2s 13ms/step - loss: 0.0244 - val_loss: 0.0231
Epoch 5/50
147/147 ━━━━━━━━━━━━━━━━━━━━ 2s 9ms/step - loss: 0.0236 - val_loss: 0.0230
Epoch 6/50
147/147 ━━━━━━━━━━━━━━━━━━━━ 3s 10ms/step - loss: 0.0235 - val_loss: 0.0217
Epoch 7/50
147/147 ━━━━━━━━━━━━━━━━━━━━ 2s 9ms/step - loss: 0.0212 - val_loss: 0.0225
Epoch 8/50
147/147 ━━━━━━━━━━━━━━━━━━━━ 1s 9ms/step - loss: 0.0212 - val_loss: 0.0207
Epoch 9/50
147/147 ━━━━━━━━━━━━━━━━━━━━ 1s 9ms/step - loss: 0.0218 - val_loss: 0.0223
Epoch 10/50
147/147 ━━━━━━━━━━━━━━━━━━━━ 3s 11ms/step - loss: 0.0210 - val_loss: 0.0208
Epoch 11/50
147/147 ━━━━━━━━━━━━━━━━━━━━ 1s 9ms/step - loss: 0.0215 - val_loss: 0.0206
Epoch 12/50
147/147 ━━━━━━━━━━━━━━━━━━━━ 1s 9ms/step - loss: 0.0216 - val_loss: 0.0202
Epoch 13/50
147/147 ━━━━━━━━━━━━━━━━━━━━ 3s 9ms/step - loss: 0.0223 - val_loss: 0.0200
Epoch 14/50
147/147 ━━━━━━━━━━━━━━━━━━━━ 1s 9ms/step - loss: 0.0209 - val_loss: 0.0201
Epoch 15/50
```

```
147/147 ━━━━━━━━━━━━━━━━━━━━ 3s 9ms/step - loss: 0.0215 - val_loss: 0.0198
Epoch 16/50
147/147 ━━━━━━━━━━━━━━━━━━━━ 2s 13ms/step - loss: 0.0212 - val_loss: 0.0204
Epoch 17/50
147/147 ━━━━━━━━━━━━━━━━━━━━ 2s 10ms/step - loss: 0.0219 - val_loss: 0.0200
Epoch 18/50
147/147 ━━━━━━━━━━━━━━━━━━━━ 2s 9ms/step - loss: 0.0205 - val_loss: 0.0199
Epoch 19/50
147/147 ━━━━━━━━━━━━━━━━━━━━ 1s 9ms/step - loss: 0.0211 - val_loss: 0.0201
Epoch 20/50
147/147 ━━━━━━━━━━━━━━━━━━━━ 3s 9ms/step - loss: 0.0204 - val_loss: 0.0201
Epoch 21/50
147/147 ━━━━━━━━━━━━━━━━━━━━ 2s 9ms/step - loss: 0.0214 - val_loss: 0.0196
Epoch 22/50
147/147 ━━━━━━━━━━━━━━━━━━━━ 2s 12ms/step - loss: 0.0208 - val_loss: 0.0199
Epoch 23/50
147/147 ━━━━━━━━━━━━━━━━━━━━ 2s 9ms/step - loss: 0.0214 - val_loss: 0.0197
Epoch 24/50
147/147 ━━━━━━━━━━━━━━━━━━━━ 1s 9ms/step - loss: 0.0208 - val_loss: 0.0196
Epoch 25/50
147/147 ━━━━━━━━━━━━━━━━━━━━ 3s 10ms/step - loss: 0.0214 - val_loss: 0.0204
Epoch 26/50
147/147 ━━━━━━━━━━━━━━━━━━━━ 1s 9ms/step - loss: 0.0205 - val_loss: 0.0205
Epoch 27/50
147/147 ━━━━━━━━━━━━━━━━━━━━ 3s 9ms/step - loss: 0.0217 - val_loss: 0.0197
Epoch 28/50
147/147 ━━━━━━━━━━━━━━━━━━━━ 2s 12ms/step - loss: 0.0203 - val_loss: 0.0212
Epoch 29/50
147/147 ━━━━━━━━━━━━━━━━━━━━ 2s 9ms/step - loss: 0.0207 - val_loss: 0.0192
Epoch 30/50
147/147 ━━━━━━━━━━━━━━━━━━━━ 1s 9ms/step - loss: 0.0194 - val_loss: 0.0215
Epoch 31/50
147/147 ━━━━━━━━━━━━━━━━━━━━ 3s 9ms/step - loss: 0.0212 - val_loss: 0.0197
Epoch 32/50
147/147 ━━━━━━━━━━━━━━━━━━━━ 1s 9ms/step - loss: 0.0202 - val_loss: 0.0195
Epoch 33/50
147/147 ━━━━━━━━━━━━━━━━━━━━ 3s 9ms/step - loss: 0.0207 - val_loss: 0.0190
Epoch 34/50
147/147 ━━━━━━━━━━━━━━━━━━━━ 3s 13ms/step - loss: 0.0204 - val_loss: 0.0192
Epoch 35/50
147/147 ━━━━━━━━━━━━━━━━━━━━ 1s 9ms/step - loss: 0.0207 - val_loss: 0.0193
Epoch 36/50
147/147 ━━━━━━━━━━━━━━━━━━━━ 1s 9ms/step - loss: 0.0203 - val_loss: 0.0213
Epoch 37/50
147/147 ━━━━━━━━━━━━━━━━━━━━ 1s 9ms/step - loss: 0.0208 - val_loss: 0.0195
Epoch 38/50
147/147 ━━━━━━━━━━━━━━━━━━━━ 2s 9ms/step - loss: 0.0208 - val_loss: 0.0191
Epoch 39/50
147/147 ━━━━━━━━━━━━━━━━━━━━ 3s 9ms/step - loss: 0.0204 - val_loss: 0.0193
[illegible]
```

```
Epoch 40/50
147/147 ━━━━━━━━━━━━━━━━━━━━ 2s 12ms/step - loss: 0.0201 - val_loss: 0.0188
Epoch 41/50
147/147 ━━━━━━━━━━━━━━━━━━━━ 2s 12ms/step - loss: 0.0210 - val_loss: 0.0189
Epoch 42/50
147/147 ━━━━━━━━━━━━━━━━━━━━ 1s 9ms/step - loss: 0.0196 - val_loss: 0.0194
Epoch 43/50
147/147 ━━━━━━━━━━━━━━━━━━━━ 1s 9ms/step - loss: 0.0196 - val_loss: 0.0190
Epoch 44/50
147/147 ━━━━━━━━━━━━━━━━━━━━ 3s 10ms/step - loss: 0.0182 - val_loss: 0.0200
Epoch 45/50
147/147 ━━━━━━━━━━━━━━━━━━━━ 2s 9ms/step - loss: 0.0198 - val_loss: 0.0193
Epoch 46/50
147/147 ━━━━━━━━━━━━━━━━━━━━ 3s 13ms/step - loss: 0.0204 - val_loss: 0.0187
Epoch 47/50
147/147 ━━━━━━━━━━━━━━━━━━━━ 2s 9ms/step - loss: 0.0188 - val_loss: 0.0188
Epoch 48/50
147/147 ━━━━━━━━━━━━━━━━━━━━ 3s 9ms/step - loss: 0.0199 - val_loss: 0.0185
Epoch 49/50
147/147 ━━━━━━━━━━━━━━━━━━━━ 3s 9ms/step - loss: 0.0197 - val_loss: 0.0187
Epoch 50/50
147/147 ━━━━━━━━━━━━━━━━━━━━ 3s 9ms/step - loss: 0.0199 - val_loss: 0.0194
74/74 ━━━━━━━━━━━━━━━━━━━━ 1s 10ms/step
```

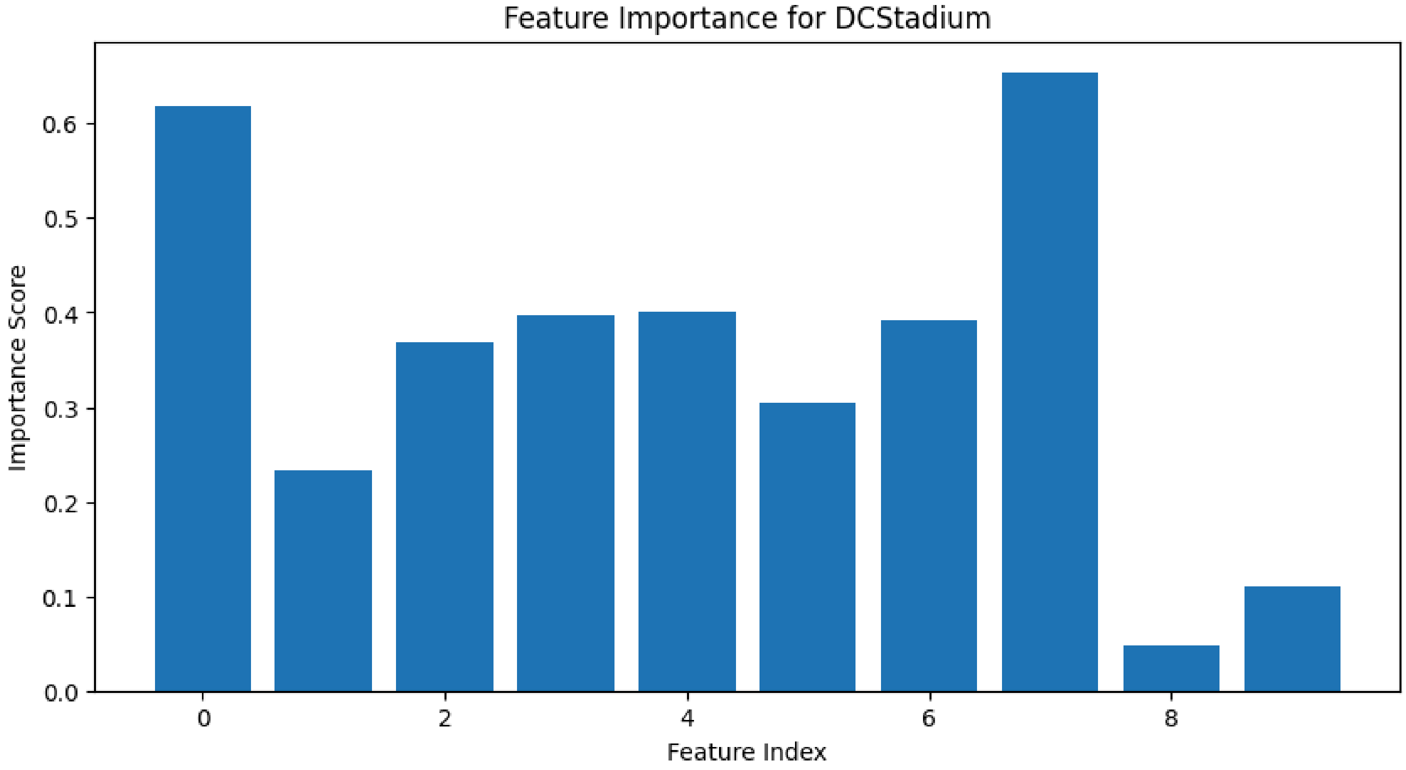

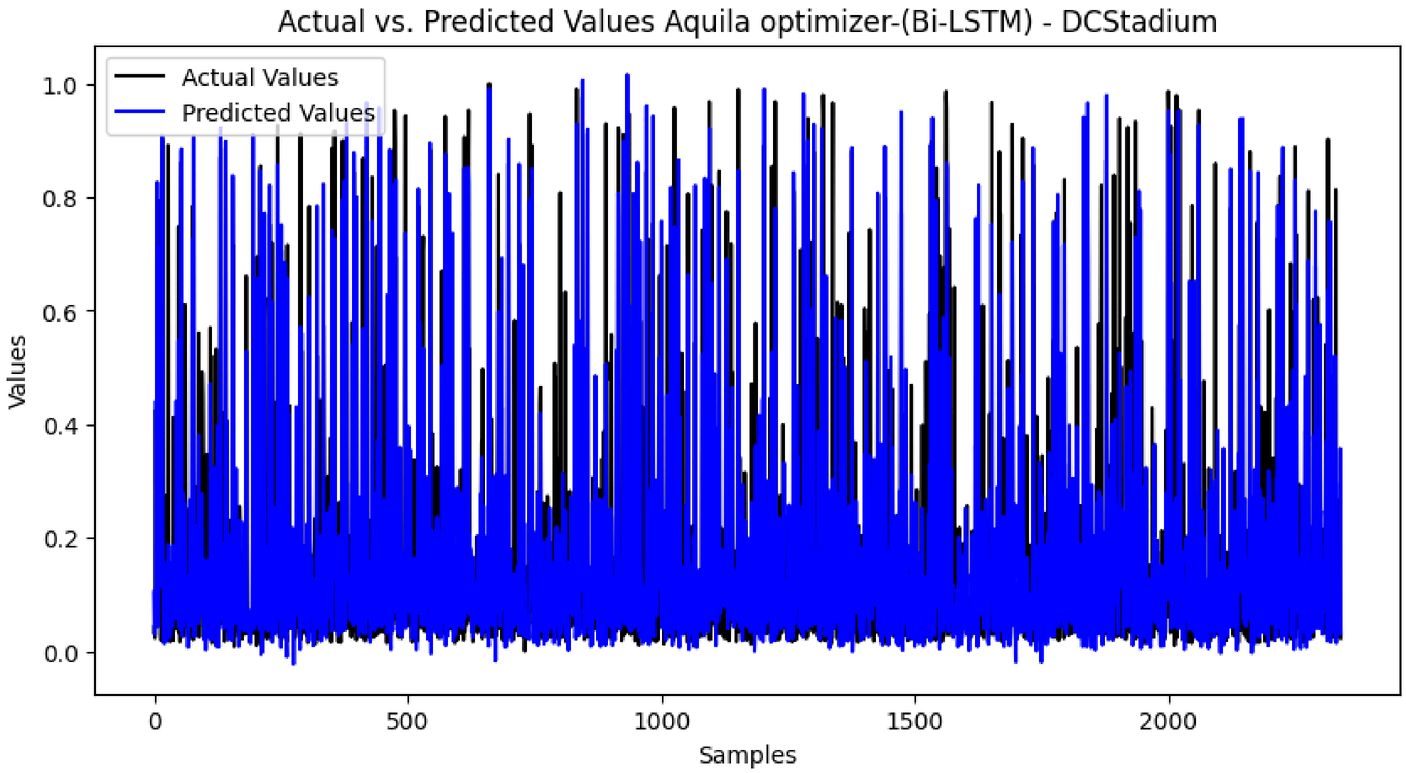


```
Processing Station: DwarkaSec8
/usr/local/lib/python3.11/dist-packages/pywt/_multilevel.py:43: UserWarning: Level value of 3 is too high: all coefficients will experience boundary effe
  warnings.warn(
Epoch 1/50
/usr/local/lib/python3.11/dist-packages/keras/src/layers/rnn/rnn.py:200: UserWarning: Do not pass an `input_shape`/`input_dim` argument to a layer. When
  super().__init__(**kwargs)
147/147 ━━━━━━━━━━━━━━━━━━━━ 6s 13ms/step - loss: 0.0073 - val_loss: 0.0033
Epoch 2/50
147/147 ━━━━━━━━━━━━━━━━━━━━ 2s 10ms/step - loss: 0.0030 - val_loss: 0.0020
Epoch 3/50
147/147 ━━━━━━━━━━━━━━━━━━━━ 3s 13ms/step - loss: 0.0020 - val_loss: 0.0017
Epoch 4/50
147/147 ━━━━━━━━━━━━━━━━━━━━ 2s 12ms/step - loss: 0.0015 - val_loss: 0.0016
Epoch 5/50
147/147 ━━━━━━━━━━━━━━━━━━━━ 2s 10ms/step - loss: 0.0015 - val_loss: 0.0016
Epoch 6/50
147/147 ━━━━━━━━━━━━━━━━━━━━ 2s 11ms/step - loss: 0.0014 - val_loss: 0.0014
Epoch 7/50
147/147 ━━━━━━━━━━━━━━━━━━━━ 2s 11ms/step - loss: 0.0013 - val_loss: 0.0014
Epoch 8/50
147/147 ━━━━━━━━━━━━━━━━━━━━ 3s 11ms/step - loss: 0.0013 - val_loss: 0.0015
```

```
[illegible]
Epoch 9/50
147/147 ━━━━━━━━━━━━━━━━━━━━ 3s 15ms/step - loss: 0.0012 - val_loss: 0.0013
Epoch 10/50
147/147 ━━━━━━━━━━━━━━━━━━━━ 2s 10ms/step - loss: 0.0011 - val_loss: 0.0012
Epoch 11/50
147/147 ━━━━━━━━━━━━━━━━━━━━ 1s 10ms/step - loss: 0.0011 - val_loss: 0.0013
Epoch 12/50
147/147 ━━━━━━━━━━━━━━━━━━━━ 1s 10ms/step - loss: 0.0011 - val_loss: 0.0012
Epoch 13/50
147/147 ━━━━━━━━━━━━━━━━━━━━ 3s 10ms/step - loss: 0.0011 - val_loss: 0.0011
Epoch 14/50
147/147 ━━━━━━━━━━━━━━━━━━━━ 1s 10ms/step - loss: 0.0010 - val_loss: 0.0011
Epoch 15/50
147/147 ━━━━━━━━━━━━━━━━━━━━ 2s 11ms/step - loss: 0.0010 - val_loss: 0.0011
Epoch 16/50
147/147 ━━━━━━━━━━━━━━━━━━━━ 3s 13ms/step - loss: 0.0010 - val_loss: 0.0011
Epoch 17/50
147/147 ━━━━━━━━━━━━━━━━━━━━ 2s 10ms/step - loss: 9.4827e-04 - val_loss: 0.0010
Epoch 18/50
147/147 ━━━━━━━━━━━━━━━━━━━━ 2s 10ms/step - loss: 9.9578e-04 - val_loss: 0.0012
Epoch 19/50
147/147 ━━━━━━━━━━━━━━━━━━━━ 3s 10ms/step - loss: 0.0010 - val_loss: 0.0011
Epoch 20/50
147/147 ━━━━━━━━━━━━━━━━━━━━ 2s 10ms/step - loss: 0.0010 - val_loss: 0.0012
Epoch 21/50
147/147 ━━━━━━━━━━━━━━━━━━━━ 2s 10ms/step - loss: 0.0010 - val_loss: 9.7659e-04
Epoch 22/50
147/147 ━━━━━━━━━━━━━━━━━━━━ 3s 13ms/step - loss: 9.2916e-04 - val_loss: 0.0011
Epoch 23/50
147/147 ━━━━━━━━━━━━━━━━━━━━ 1s 10ms/step - loss: 8.7430e-04 - val_loss: 9.8756e-04
Epoch 24/50
147/147 ━━━━━━━━━━━━━━━━━━━━ 1s 10ms/step - loss: 8.9808e-04 - val_loss: 9.6434e-04
Epoch 25/50
147/147 ━━━━━━━━━━━━━━━━━━━━ 3s 10ms/step - loss: 8.3742e-04 - val_loss: 9.4717e-04
Epoch 26/50
147/147 ━━━━━━━━━━━━━━━━━━━━ 3s 11ms/step - loss: 8.9275e-04 - val_loss: 9.9203e-04
Epoch 27/50
147/147 ━━━━━━━━━━━━━━━━━━━━ 2s 14ms/step - loss: 8.2234e-04 - val_loss: 0.0010
Epoch 28/50
147/147 ━━━━━━━━━━━━━━━━━━━━ 2s 12ms/step - loss: 8.9401e-04 - val_loss: 0.0010
Epoch 29/50
147/147 ━━━━━━━━━━━━━━━━━━━━ 1s 10ms/step - loss: 8.4625e-04 - val_loss: 0.0010
Epoch 30/50
147/147 ━━━━━━━━━━━━━━━━━━━━ 3s 10ms/step - loss: 7.8990e-04 - val_loss: 9.3014e-04
Epoch 31/50
147/147 ━━━━━━━━━━━━━━━━━━━━ 1s 10ms/step - loss: 8.0295e-04 - val_loss: 0.0010
Epoch 32/50
147/147 ━━━━━━━━━━━━━━━━━━━━ 3s 10ms/step - loss: 8.4449e-04 - val_loss: 9.6262e-04
Epoch 33/50
```

```
147/147 ━━━━━━━━━━━━━━━━━━━━ 2s 14ms/step - loss: 8.0220e-04 - val_loss: 9.7331e-04
Epoch 34/50
147/147 ━━━━━━━━━━━━━━━━━━━━ 2s 10ms/step - loss: 8.1145e-04 - val_loss: 9.1753e-04
Epoch 35/50
147/147 ━━━━━━━━━━━━━━━━━━━━ 1s 10ms/step - loss: 7.4095e-04 - val_loss: 8.9841e-04
Epoch 36/50
147/147 ━━━━━━━━━━━━━━━━━━━━ 3s 11ms/step - loss: 7.3473e-04 - val_loss: 9.5316e-04
Epoch 37/50
147/147 ━━━━━━━━━━━━━━━━━━━━ 2s 10ms/step - loss: 8.0396e-04 - val_loss: 8.6646e-04
Epoch 38/50
147/147 ━━━━━━━━━━━━━━━━━━━━ 3s 11ms/step - loss: 6.9882e-04 - val_loss: 9.2462e-04
Epoch 39/50
147/147 ━━━━━━━━━━━━━━━━━━━━ 2s 15ms/step - loss: 7.0725e-04 - val_loss: 9.3461e-04
Epoch 40/50
147/147 ━━━━━━━━━━━━━━━━━━━━ 2s 11ms/step - loss: 6.9490e-04 - val_loss: 8.5813e-04
Epoch 41/50
147/147 ━━━━━━━━━━━━━━━━━━━━ 2s 10ms/step - loss: 6.8819e-04 - val_loss: 8.5943e-04
Epoch 42/50
147/147 ━━━━━━━━━━━━━━━━━━━━ 3s 11ms/step - loss: 7.1401e-04 - val_loss: 8.5633e-04
Epoch 43/50
147/147 ━━━━━━━━━━━━━━━━━━━━ 2s 10ms/step - loss: 6.5758e-04 - val_loss: 0.0013
Epoch 44/50
147/147 ━━━━━━━━━━━━━━━━━━━━ 3s 12ms/step - loss: 7.4408e-04 - val_loss: 7.9967e-04
Epoch 45/50
147/147 ━━━━━━━━━━━━━━━━━━━━ 2s 11ms/step - loss: 6.2087e-04 - val_loss: 7.7848e-04
Epoch 46/50
147/147 ━━━━━━━━━━━━━━━━━━━━ 2s 10ms/step - loss: 6.1877e-04 - val_loss: 8.3615e-04
Epoch 47/50
147/147 ━━━━━━━━━━━━━━━━━━━━ 2s 11ms/step - loss: 6.8490e-04 - val_loss: 8.1257e-04
Epoch 48/50
147/147 ━━━━━━━━━━━━━━━━━━━━ 3s 11ms/step - loss: 6.1901e-04 - val_loss: 7.5058e-04
Epoch 49/50
147/147 ━━━━━━━━━━━━━━━━━━━━ 3s 11ms/step - loss: 5.7741e-04 - val_loss: 8.7839e-04
Epoch 50/50
147/147 ━━━━━━━━━━━━━━━━━━━━ 3s 12ms/step - loss: 6.6723e-04 - val_loss: 7.6393e-04
74/74 ━━━━━━━━━━━━━━━━━━━━ 1s 8ms/step
```

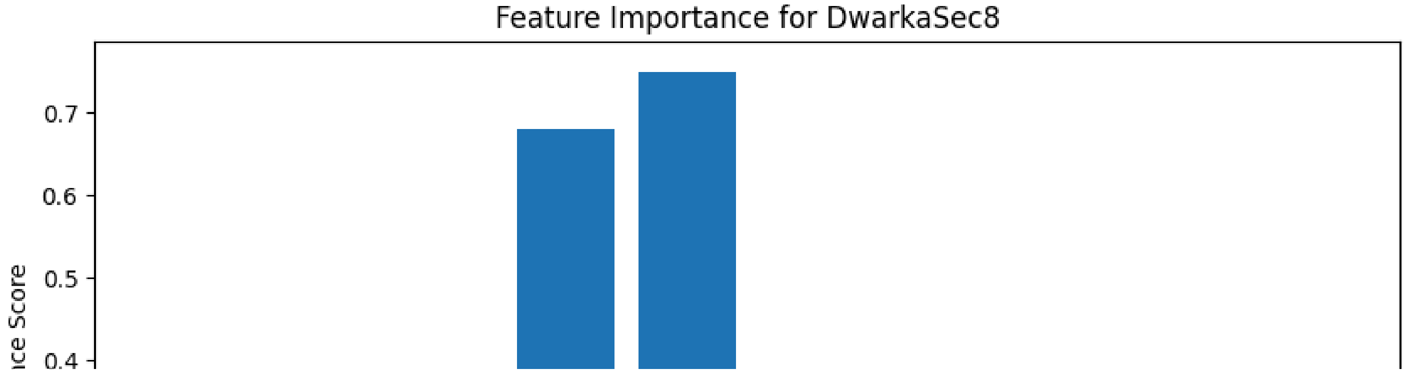

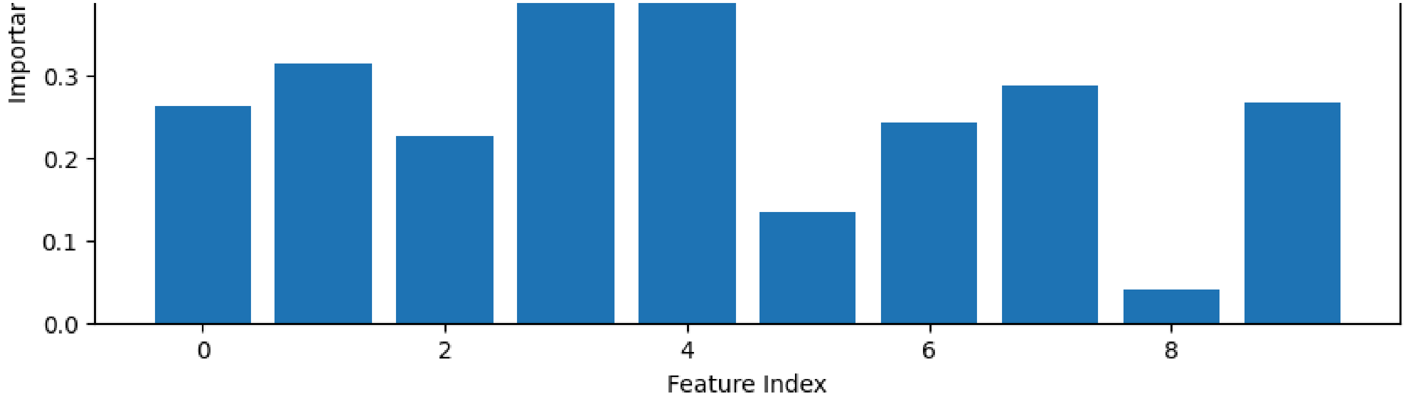

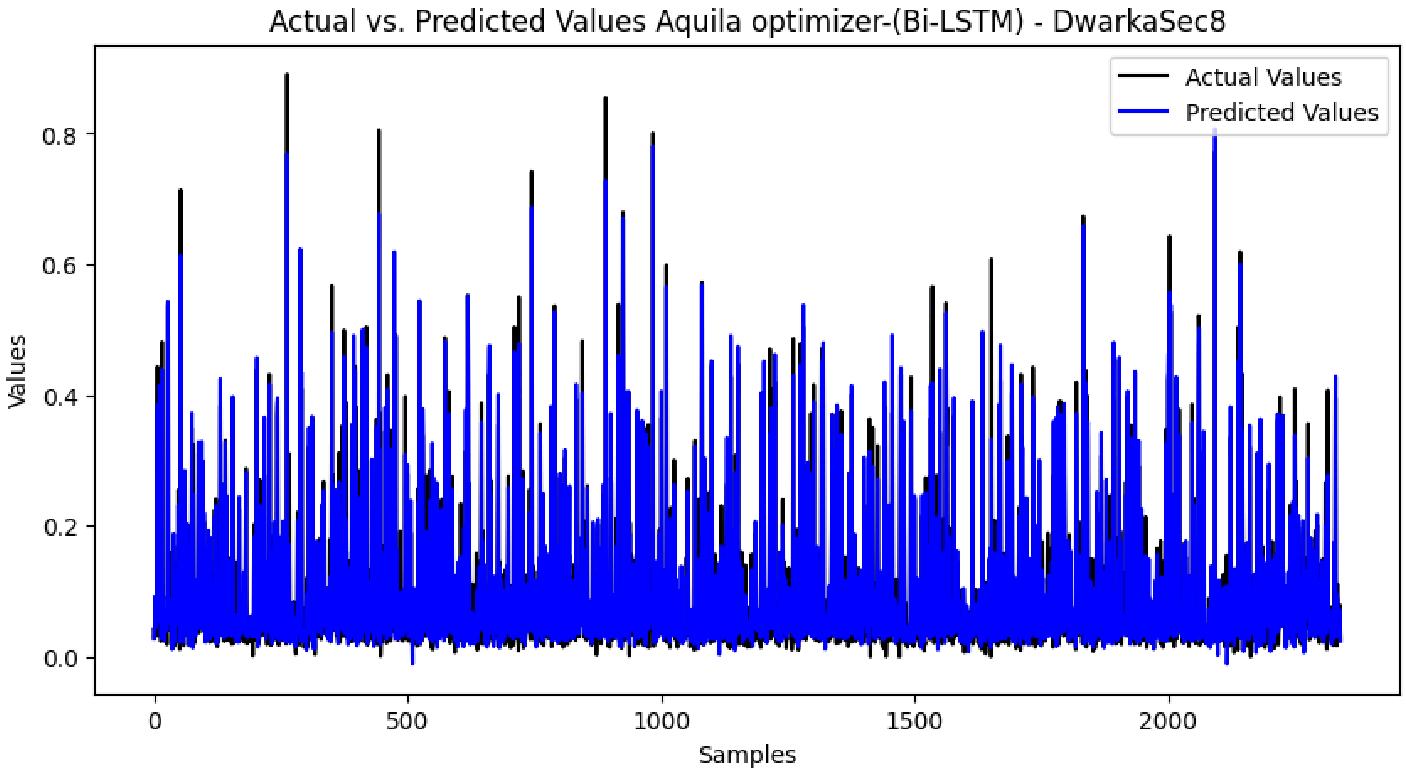


```
Processing Station: NehruNagar
/usr/local/lib/python3.11/dist-packages/pywt/_multilevel.py:43: UserWarning: Level value of 3 is too high: all coefficients will experience boundary effe
  warnings.warn(
Epoch 1/50
/usr/local/lib/python3.11/dist-packages/keras/src/layers/rnn/rnn.py:200: UserWarning: Do not pass an `input_shape`/`input_dim` argument to a layer. When
  super().__init__(**kwargs)
147/147 ━━━━━━━━━━━━━━━━━━━━ 5s 12ms/step - loss: 0.0321 - val_loss: 0.0244
```

```
Epoch 2/50
147/147 ━━━━━━━━━━━━━━━━━━━━ 1s 10ms/step - loss: 0.0227 - val_loss: 0.0241
Epoch 3/50
147/147 ━━━━━━━━━━━━━━━━━━━━ 3s 11ms/step - loss: 0.0235 - val_loss: 0.0242
Epoch 4/50
147/147 ━━━━━━━━━━━━━━━━━━━━ 2s 10ms/step - loss: 0.0226 - val_loss: 0.0242
Epoch 5/50
147/147 ━━━━━━━━━━━━━━━━━━━━ 1s 9ms/step - loss: 0.0227 - val_loss: 0.0243
Epoch 6/50
147/147 ━━━━━━━━━━━━━━━━━━━━ 1s 9ms/step - loss: 0.0232 - val_loss: 0.0242
Epoch 7/50
147/147 ━━━━━━━━━━━━━━━━━━━━ 1s 10ms/step - loss: 0.0237 - val_loss: 0.0240
Epoch 8/50
147/147 ━━━━━━━━━━━━━━━━━━━━ 2s 11ms/step - loss: 0.0238 - val_loss: 0.0243
Epoch 9/50
147/147 ━━━━━━━━━━━━━━━━━━━━ 3s 13ms/step - loss: 0.0215 - val_loss: 0.0240
Epoch 10/50
147/147 ━━━━━━━━━━━━━━━━━━━━ 2s 10ms/step - loss: 0.0226 - val_loss: 0.0241
Epoch 11/50
147/147 ━━━━━━━━━━━━━━━━━━━━ 3s 9ms/step - loss: 0.0218 - val_loss: 0.0242
Epoch 12/50
147/147 ━━━━━━━━━━━━━━━━━━━━ 1s 10ms/step - loss: 0.0232 - val_loss: 0.0239
Epoch 13/50
147/147 ━━━━━━━━━━━━━━━━━━━━ 1s 9ms/step - loss: 0.0225 - val_loss: 0.0241
Epoch 14/50
147/147 ━━━━━━━━━━━━━━━━━━━━ 1s 9ms/step - loss: 0.0226 - val_loss: 0.0239
Epoch 15/50
147/147 ━━━━━━━━━━━━━━━━━━━━ 3s 13ms/step - loss: 0.0226 - val_loss: 0.0241
Epoch 16/50
147/147 ━━━━━━━━━━━━━━━━━━━━ 2s 9ms/step - loss: 0.0225 - val_loss: 0.0242
Epoch 17/50
147/147 ━━━━━━━━━━━━━━━━━━━━ 3s 10ms/step - loss: 0.0230 - val_loss: 0.0240
Epoch 18/50
147/147 ━━━━━━━━━━━━━━━━━━━━ 2s 9ms/step - loss: 0.0221 - val_loss: 0.0240
Epoch 19/50
147/147 ━━━━━━━━━━━━━━━━━━━━ 3s 9ms/step - loss: 0.0226 - val_loss: 0.0239
Epoch 20/50
147/147 ━━━━━━━━━━━━━━━━━━━━ 3s 15ms/step - loss: 0.0217 - val_loss: 0.0240
Epoch 21/50
147/147 ━━━━━━━━━━━━━━━━━━━━ 2s 10ms/step - loss: 0.0226 - val_loss: 0.0238
Epoch 22/50
147/147 ━━━━━━━━━━━━━━━━━━━━ 3s 9ms/step - loss: 0.0222 - val_loss: 0.0239
Epoch 23/50
147/147 ━━━━━━━━━━━━━━━━━━━━ 1s 9ms/step - loss: 0.0228 - val_loss: 0.0241
Epoch 24/50
147/147 ━━━━━━━━━━━━━━━━━━━━ 3s 9ms/step - loss: 0.0227 - val_loss: 0.0240
Epoch 25/50
147/147 ━━━━━━━━━━━━━━━━━━━━ 2s 11ms/step - loss: 0.0228 - val_loss: 0.0238
Epoch 26/50
[illegible]
```

```
147/147 ━━━━━━━━━━━━━━━━━━━━ 3s 11ms/step - loss: 0.0232 - val_loss: 0.0240
Epoch 27/50
147/147 ━━━━━━━━━━━━━━━━━━━━ 2s 9ms/step - loss: 0.0223 - val_loss: 0.0239
Epoch 28/50
147/147 ━━━━━━━━━━━━━━━━━━━━ 1s 10ms/step - loss: 0.0229 - val_loss: 0.0239
Epoch 29/50
147/147 ━━━━━━━━━━━━━━━━━━━━ 3s 9ms/step - loss: 0.0220 - val_loss: 0.0240
Epoch 30/50
147/147 ━━━━━━━━━━━━━━━━━━━━ 1s 9ms/step - loss: 0.0219 - val_loss: 0.0238
Epoch 31/50
147/147 ━━━━━━━━━━━━━━━━━━━━ 1s 10ms/step - loss: 0.0225 - val_loss: 0.0239
Epoch 32/50
147/147 ━━━━━━━━━━━━━━━━━━━━ 2s 13ms/step - loss: 0.0227 - val_loss: 0.0239
Epoch 33/50
147/147 ━━━━━━━━━━━━━━━━━━━━ 2s 10ms/step - loss: 0.0219 - val_loss: 0.0239
Epoch 34/50
147/147 ━━━━━━━━━━━━━━━━━━━━ 1s 9ms/step - loss: 0.0217 - val_loss: 0.0239
Epoch 35/50
147/147 ━━━━━━━━━━━━━━━━━━━━ 1s 9ms/step - loss: 0.0222 - val_loss: 0.0239
Epoch 36/50
147/147 ━━━━━━━━━━━━━━━━━━━━ 1s 10ms/step - loss: 0.0222 - val_loss: 0.0239
Epoch 37/50
147/147 ━━━━━━━━━━━━━━━━━━━━ 3s 9ms/step - loss: 0.0225 - val_loss: 0.0239
Epoch 38/50
147/147 ━━━━━━━━━━━━━━━━━━━━ 3s 13ms/step - loss: 0.0223 - val_loss: 0.0238
Epoch 39/50
147/147 ━━━━━━━━━━━━━━━━━━━━ 2s 12ms/step - loss: 0.0224 - val_loss: 0.0240
Epoch 40/50
147/147 ━━━━━━━━━━━━━━━━━━━━ 2s 9ms/step - loss: 0.0221 - val_loss: 0.0238
Epoch 41/50
147/147 ━━━━━━━━━━━━━━━━━━━━ 1s 9ms/step - loss: 0.0219 - val_loss: 0.0240
Epoch 42/50
147/147 ━━━━━━━━━━━━━━━━━━━━ 3s 9ms/step - loss: 0.0229 - val_loss: 0.0239
Epoch 43/50
147/147 ━━━━━━━━━━━━━━━━━━━━ 3s 9ms/step - loss: 0.0210 - val_loss: 0.0238
Epoch 44/50
147/147 ━━━━━━━━━━━━━━━━━━━━ 2s 12ms/step - loss: 0.0226 - val_loss: 0.0238
Epoch 45/50
147/147 ━━━━━━━━━━━━━━━━━━━━ 2s 9ms/step - loss: 0.0224 - val_loss: 0.0239
Epoch 46/50
147/147 ━━━━━━━━━━━━━━━━━━━━ 3s 9ms/step - loss: 0.0226 - val_loss: 0.0238
Epoch 47/50
147/147 ━━━━━━━━━━━━━━━━━━━━ 1s 10ms/step - loss: 0.0227 - val_loss: 0.0239
Epoch 48/50
147/147 ━━━━━━━━━━━━━━━━━━━━ 3s 10ms/step - loss: 0.0218 - val_loss: 0.0239
Epoch 49/50
147/147 ━━━━━━━━━━━━━━━━━━━━ 2s 11ms/step - loss: 0.0218 - val_loss: 0.0239
Epoch 50/50
147/147 ━━━━━━━━━━━━━━━━━━━━ 2s 12ms/step - loss: 0.0218 - val_loss: 0.0239
74/74 ━━━━━━━━━━━━━━━━━━━━ 1s 10ms/step
```

74/74 1s 16ms/step

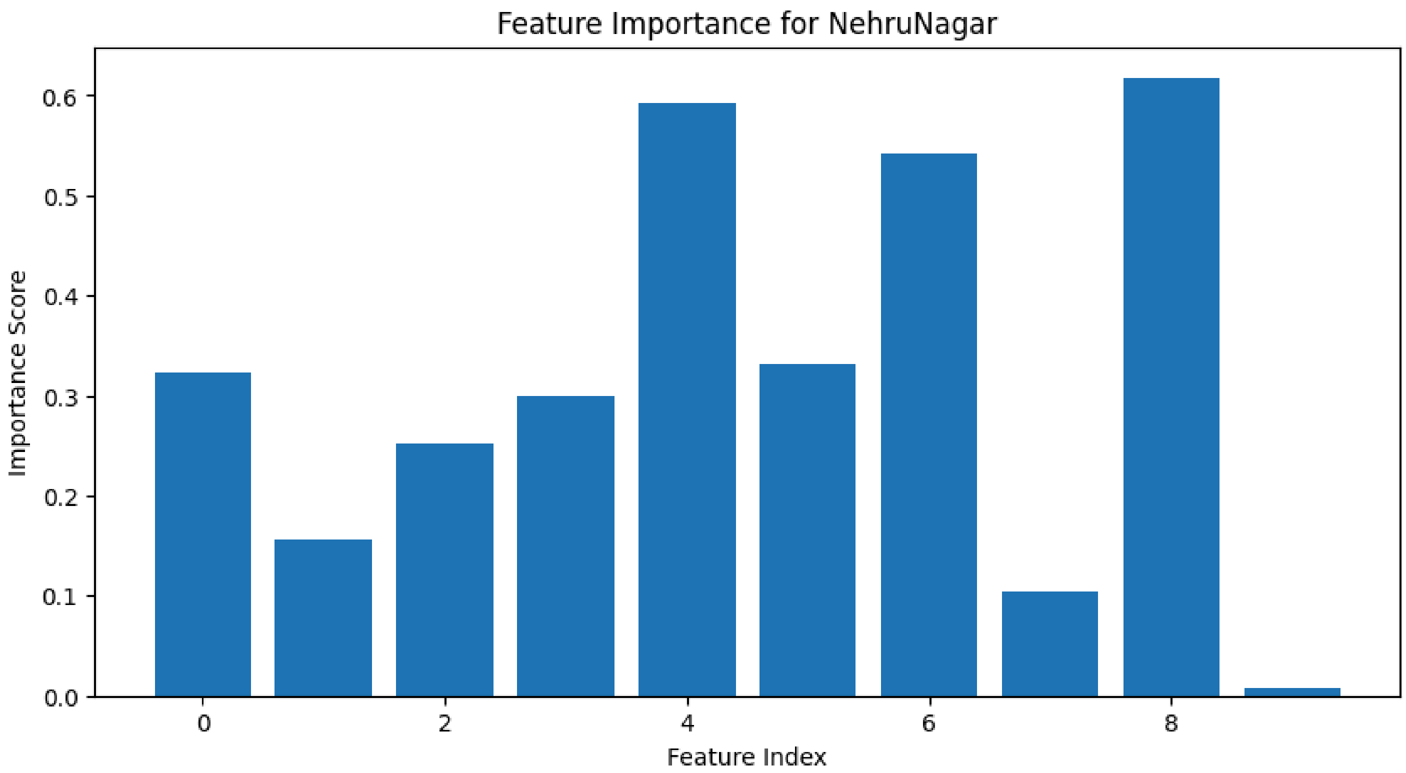

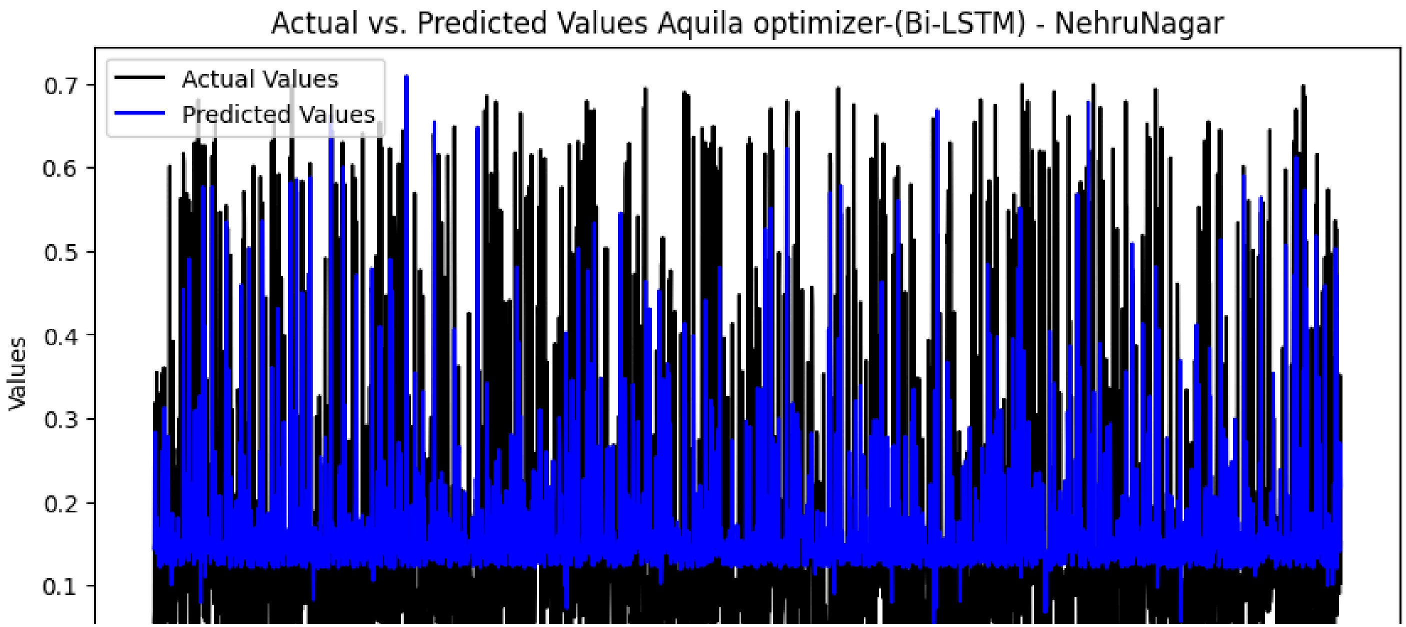

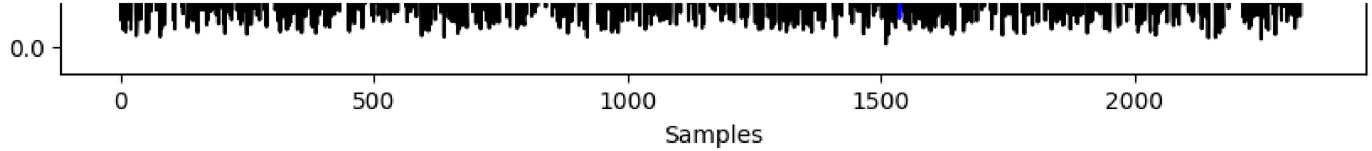


```
Processing Station: Najafgarh
/usr/local/lib/python3.11/dist-packages/pywt/_multilevel.py:43: UserWarning: Level value of 3 is too high: all coefficients will experience boundary effe
  warnings.warn(
Epoch 1/50
/usr/local/lib/python3.11/dist-packages/keras/src/layers/rnn/rnn.py:200: UserWarning: Do not pass an `input_shape`/`input_dim` argument to a layer. When
  super().__init__(**kwargs)
147/147 ━━━━━━━━━━━━━━━━━━━━ 5s 12ms/step - loss: 0.0031 - val_loss: 0.0021
Epoch 2/50
147/147 ━━━━━━━━━━━━━━━━━━━━ 1s 10ms/step - loss: 0.0027 - val_loss: 0.0021
Epoch 3/50
147/147 ━━━━━━━━━━━━━━━━━━━━ 1s 10ms/step - loss: 0.0025 - val_loss: 0.0020
Epoch 4/50
147/147 ━━━━━━━━━━━━━━━━━━━━ 2s 12ms/step - loss: 0.0024 - val_loss: 0.0021
Epoch 5/50
147/147 ━━━━━━━━━━━━━━━━━━━━ 2s 9ms/step - loss: 0.0023 - val_loss: 0.0020
Epoch 6/50
147/147 ━━━━━━━━━━━━━━━━━━━━ 1s 10ms/step - loss: 0.0026 - val_loss: 0.0019
Epoch 7/50
147/147 ━━━━━━━━━━━━━━━━━━━━ 3s 9ms/step - loss: 0.0021 - val_loss: 0.0019
Epoch 8/50
147/147 ━━━━━━━━━━━━━━━━━━━━ 3s 9ms/step - loss: 0.0024 - val_loss: 0.0019
Epoch 9/50
147/147 ━━━━━━━━━━━━━━━━━━━━ 1s 9ms/step - loss: 0.0020 - val_loss: 0.0018
Epoch 10/50
147/147 ━━━━━━━━━━━━━━━━━━━━ 2s 12ms/step - loss: 0.0020 - val_loss: 0.0017
Epoch 11/50
147/147 ━━━━━━━━━━━━━━━━━━━━ 2s 10ms/step - loss: 0.0020 - val_loss: 0.0017
Epoch 12/50
147/147 ━━━━━━━━━━━━━━━━━━━━ 2s 9ms/step - loss: 0.0019 - val_loss: 0.0016
Epoch 13/50
147/147 ━━━━━━━━━━━━━━━━━━━━ 2s 10ms/step - loss: 0.0020 - val_loss: 0.0016
Epoch 14/50
147/147 ━━━━━━━━━━━━━━━━━━━━ 1s 9ms/step - loss: 0.0021 - val_loss: 0.0015
Epoch 15/50
147/147 ━━━━━━━━━━━━━━━━━━━━ 3s 10ms/step - loss: 0.0020 - val_loss: 0.0014
Epoch 16/50
147/147 ━━━━━━━━━━━━━━━━━━━━ 3s 13ms/step - loss: 0.0017 - val_loss: 0.0015
Epoch 17/50
147/147 ━━━━━━━━━━━━━━━━━━━━ 2s 11ms/step - loss: 0.0018 - val_loss: 0.0015
Epoch 18/50
147/147 ━━━━━━━━━━━━━━━━━━━━ 1s 10ms/step - loss: 0.0017 - val_loss: 0.0015
Epoch 19/50
147/147 ━━━━━━━━━━━━━━━━━━━━ 1s 10ms/step - loss: 0.0019 - val_loss: 0.0015
```

```
[illegible]
Epoch 20/50
147/147 ━━━━━━━━━━━━━━━━━━━━ 1s 9ms/step - loss: 0.0017 - val_loss: 0.0015
Epoch 21/50
147/147 ━━━━━━━━━━━━━━━━━━━━ 1s 9ms/step - loss: 0.0017 - val_loss: 0.0015
Epoch 22/50
147/147 ━━━━━━━━━━━━━━━━━━━━ 3s 12ms/step - loss: 0.0017 - val_loss: 0.0014
Epoch 23/50
147/147 ━━━━━━━━━━━━━━━━━━━━ 2s 10ms/step - loss: 0.0017 - val_loss: 0.0014
Epoch 24/50
147/147 ━━━━━━━━━━━━━━━━━━━━ 1s 9ms/step - loss: 0.0019 - val_loss: 0.0014
Epoch 25/50
147/147 ━━━━━━━━━━━━━━━━━━━━ 3s 10ms/step - loss: 0.0018 - val_loss: 0.0014
Epoch 26/50
147/147 ━━━━━━━━━━━━━━━━━━━━ 3s 9ms/step - loss: 0.0017 - val_loss: 0.0014
Epoch 27/50
147/147 ━━━━━━━━━━━━━━━━━━━━ 3s 11ms/step - loss: 0.0018 - val_loss: 0.0014
Epoch 28/50
147/147 ━━━━━━━━━━━━━━━━━━━━ 2s 10ms/step - loss: 0.0016 - val_loss: 0.0015
Epoch 29/50
147/147 ━━━━━━━━━━━━━━━━━━━━ 1s 9ms/step - loss: 0.0016 - val_loss: 0.0014
Epoch 30/50
147/147 ━━━━━━━━━━━━━━━━━━━━ 3s 10ms/step - loss: 0.0016 - val_loss: 0.0014
Epoch 31/50
147/147 ━━━━━━━━━━━━━━━━━━━━ 1s 9ms/step - loss: 0.0015 - val_loss: 0.0013
Epoch 32/50
147/147 ━━━━━━━━━━━━━━━━━━━━ 1s 9ms/step - loss: 0.0015 - val_loss: 0.0014
Epoch 33/50
147/147 ━━━━━━━━━━━━━━━━━━━━ 3s 11ms/step - loss: 0.0015 - val_loss: 0.0013
Epoch 34/50
147/147 ━━━━━━━━━━━━━━━━━━━━ 2s 13ms/step - loss: 0.0016 - val_loss: 0.0014
Epoch 35/50
147/147 ━━━━━━━━━━━━━━━━━━━━ 2s 10ms/step - loss: 0.0014 - val_loss: 0.0013
Epoch 36/50
147/147 ━━━━━━━━━━━━━━━━━━━━ 3s 10ms/step - loss: 0.0015 - val_loss: 0.0014
Epoch 37/50
147/147 ━━━━━━━━━━━━━━━━━━━━ 3s 10ms/step - loss: 0.0015 - val_loss: 0.0013
Epoch 38/50
147/147 ━━━━━━━━━━━━━━━━━━━━ 3s 10ms/step - loss: 0.0015 - val_loss: 0.0013
Epoch 39/50
147/147 ━━━━━━━━━━━━━━━━━━━━ 3s 12ms/step - loss: 0.0014 - val_loss: 0.0013
Epoch 40/50
147/147 ━━━━━━━━━━━━━━━━━━━━ 2s 10ms/step - loss: 0.0014 - val_loss: 0.0013
Epoch 41/50
147/147 ━━━━━━━━━━━━━━━━━━━━ 1s 10ms/step - loss: 0.0015 - val_loss: 0.0013
Epoch 42/50
147/147 ━━━━━━━━━━━━━━━━━━━━ 3s 9ms/step - loss: 0.0015 - val_loss: 0.0012
Epoch 43/50
147/147 ━━━━━━━━━━━━━━━━━━━━ 3s 9ms/step - loss: 0.0013 - val_loss: 0.0013
Epoch 44/50
```

```
147/147 ━━━━━━━━━━━━━━━━━━━━ 3s 13ms/step - loss: 0.0016 - val_loss: 0.0013
Epoch 45/50
147/147 ━━━━━━━━━━━━━━━━━━━━ 1s 9ms/step - loss: 0.0014 - val_loss: 0.0012
Epoch 46/50
147/147 ━━━━━━━━━━━━━━━━━━━━ 1s 10ms/step - loss: 0.0014 - val_loss: 0.0012
Epoch 47/50
147/147 ━━━━━━━━━━━━━━━━━━━━ 1s 9ms/step - loss: 0.0014 - val_loss: 0.0012
Epoch 48/50
147/147 ━━━━━━━━━━━━━━━━━━━━ 1s 9ms/step - loss: 0.0014 - val_loss: 0.0012
Epoch 49/50
147/147 ━━━━━━━━━━━━━━━━━━━━ 1s 9ms/step - loss: 0.0015 - val_loss: 0.0012
Epoch 50/50
147/147 ━━━━━━━━━━━━━━━━━━━━ 3s 9ms/step - loss: 0.0014 - val_loss: 0.0013
74/74 ━━━━━━━━━━━━━━━━━━━━ 2s 11ms/step
```

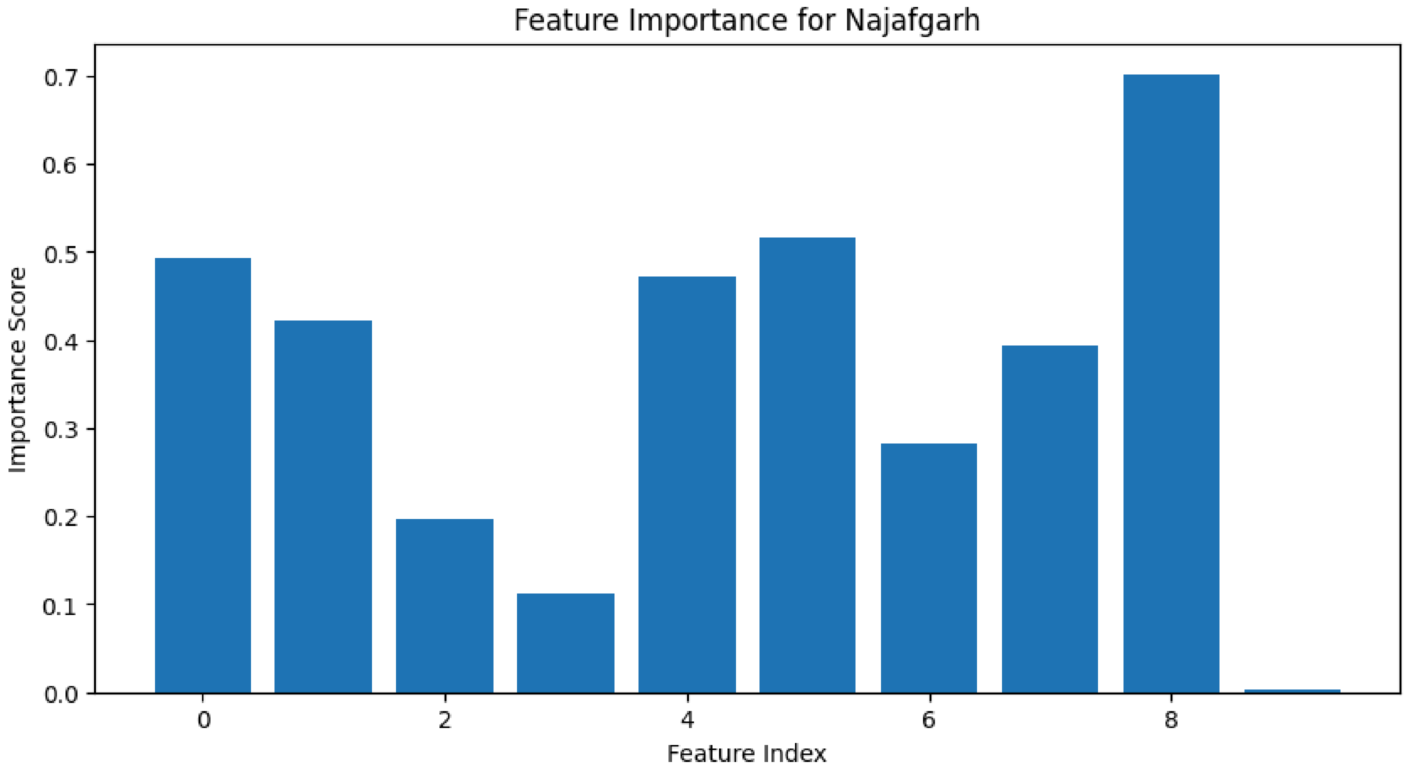

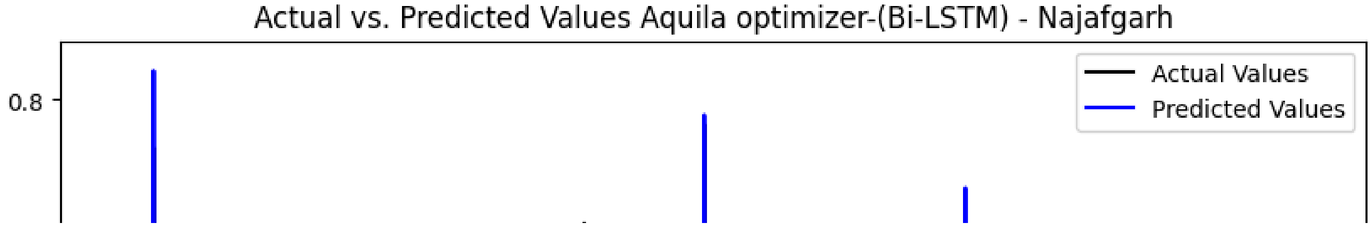

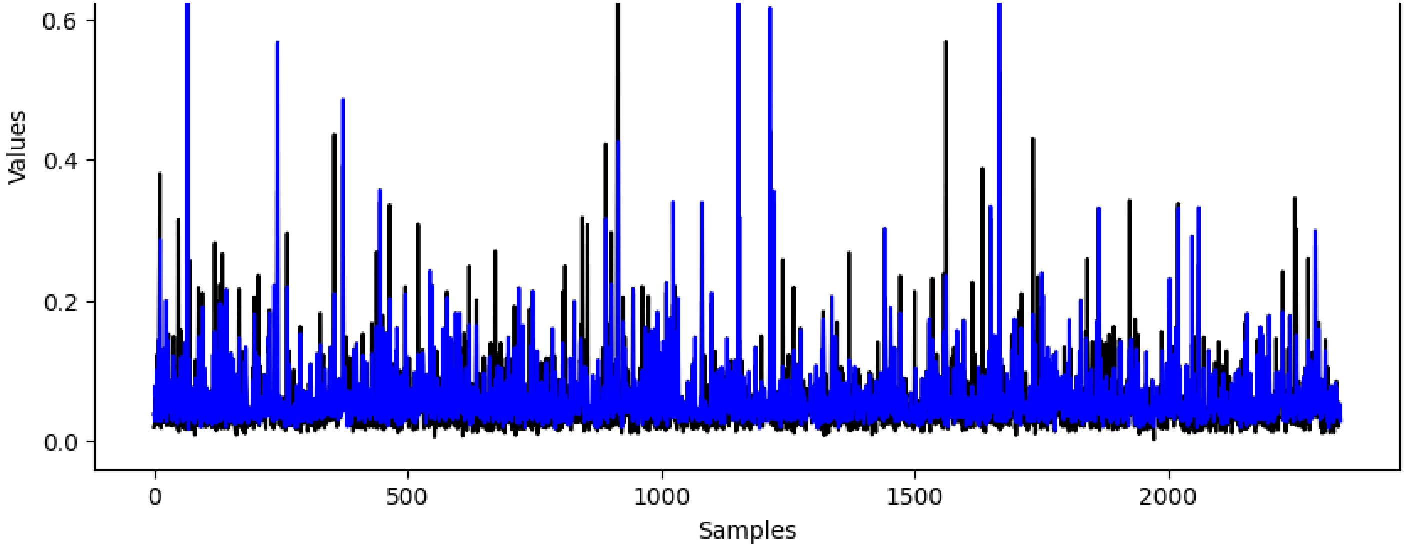


```
Processing Station: Okhla
/usr/local/lib/python3.11/dist-packages/pywt/_multilevel.py:43: UserWarning: Level value of 3 is too high: all coefficients will experience boundary effe
  warnings.warn(
Epoch 1/50
/usr/local/lib/python3.11/dist-packages/keras/src/layers/rnn/rnn.py:200: UserWarning: Do not pass an `input_shape`/`input_dim` argument to a layer. When
  super().__init__(**kwargs)
147/147 ━━━━━━━━━━━━━━━━━━━━ 5s 12ms/step - loss: 0.0182 - val_loss: 0.0164
Epoch 2/50
147/147 ━━━━━━━━━━━━━━━━━━━━ 2s 10ms/step - loss: 0.0167 - val_loss: 0.0162
Epoch 3/50
147/147 ━━━━━━━━━━━━━━━━━━━━ 3s 14ms/step - loss: 0.0159 - val_loss: 0.0162
Epoch 4/50
147/147 ━━━━━━━━━━━━━━━━━━━━ 2s 9ms/step - loss: 0.0172 - val_loss: 0.0164
Epoch 5/50
147/147 ━━━━━━━━━━━━━━━━━━━━ 1s 9ms/step - loss: 0.0164 - val_loss: 0.0162
Epoch 6/50
147/147 ━━━━━━━━━━━━━━━━━━━━ 1s 9ms/step - loss: 0.0165 - val_loss: 0.0161
Epoch 7/50
147/147 ━━━━━━━━━━━━━━━━━━━━ 3s 9ms/step - loss: 0.0157 - val_loss: 0.0168
Epoch 8/50
147/147 ━━━━━━━━━━━━━━━━━━━━ 3s 9ms/step - loss: 0.0167 - val_loss: 0.0162
Epoch 9/50
147/147 ━━━━━━━━━━━━━━━━━━━━ 3s 13ms/step - loss: 0.0168 - val_loss: 0.0160
Epoch 10/50
147/147 ━━━━━━━━━━━━━━━━━━━━ 2s 9ms/step - loss: 0.0156 - val_loss: 0.0160
Epoch 11/50
147/147 ━━━━━━━━━━━━━━━━━━━━ 3s 9ms/step - loss: 0.0161 - val_loss: 0.0160
Epoch 12/50
147/147 ━━━━━━━━━━━━━━━━━━━━ 2s 9ms/step - loss: 0.0162 - val_loss: 0.0160
```

```
Epoch 13/50
147/147 ━━━━━━━━━━━━━━━━━━━━ 3s 9ms/step - loss: 0.0165 - val_loss: 0.0160
Epoch 14/50
147/147 ━━━━━━━━━━━━━━━━━━━━ 2s 14ms/step - loss: 0.0157 - val_loss: 0.0160
Epoch 15/50
147/147 ━━━━━━━━━━━━━━━━━━━━ 2s 9ms/step - loss: 0.0156 - val_loss: 0.0160
Epoch 16/50
147/147 ━━━━━━━━━━━━━━━━━━━━ 1s 9ms/step - loss: 0.0160 - val_loss: 0.0160
Epoch 17/50
147/147 ━━━━━━━━━━━━━━━━━━━━ 3s 10ms/step - loss: 0.0154 - val_loss: 0.0160
Epoch 18/50
147/147 ━━━━━━━━━━━━━━━━━━━━ 1s 9ms/step - loss: 0.0165 - val_loss: 0.0162
Epoch 19/50
147/147 ━━━━━━━━━━━━━━━━━━━━ 3s 10ms/step - loss: 0.0161 - val_loss: 0.0160
Epoch 20/50
147/147 ━━━━━━━━━━━━━━━━━━━━ 2s 13ms/step - loss: 0.0156 - val_loss: 0.0161
Epoch 21/50
147/147 ━━━━━━━━━━━━━━━━━━━━ 2s 10ms/step - loss: 0.0150 - val_loss: 0.0160
Epoch 22/50
147/147 ━━━━━━━━━━━━━━━━━━━━ 1s 10ms/step - loss: 0.0161 - val_loss: 0.0160
Epoch 23/50
147/147 ━━━━━━━━━━━━━━━━━━━━ 3s 10ms/step - loss: 0.0162 - val_loss: 0.0160
Epoch 24/50
147/147 ━━━━━━━━━━━━━━━━━━━━ 1s 10ms/step - loss: 0.0166 - val_loss: 0.0160
Epoch 25/50
147/147 ━━━━━━━━━━━━━━━━━━━━ 3s 10ms/step - loss: 0.0170 - val_loss: 0.0159
Epoch 26/50
147/147 ━━━━━━━━━━━━━━━━━━━━ 3s 13ms/step - loss: 0.0154 - val_loss: 0.0160
Epoch 27/50
147/147 ━━━━━━━━━━━━━━━━━━━━ 1s 10ms/step - loss: 0.0162 - val_loss: 0.0161
Epoch 28/50
147/147 ━━━━━━━━━━━━━━━━━━━━ 1s 10ms/step - loss: 0.0168 - val_loss: 0.0160
Epoch 29/50
147/147 ━━━━━━━━━━━━━━━━━━━━ 2s 10ms/step - loss: 0.0160 - val_loss: 0.0160
Epoch 30/50
147/147 ━━━━━━━━━━━━━━━━━━━━ 1s 10ms/step - loss: 0.0163 - val_loss: 0.0160
Epoch 31/50
147/147 ━━━━━━━━━━━━━━━━━━━━ 3s 10ms/step - loss: 0.0164 - val_loss: 0.0159
Epoch 32/50
147/147 ━━━━━━━━━━━━━━━━━━━━ 2s 11ms/step - loss: 0.0159 - val_loss: 0.0159
Epoch 33/50
147/147 ━━━━━━━━━━━━━━━━━━━━ 3s 12ms/step - loss: 0.0158 - val_loss: 0.0160
Epoch 34/50
147/147 ━━━━━━━━━━━━━━━━━━━━ 2s 10ms/step - loss: 0.0158 - val_loss: 0.0160
Epoch 35/50
147/147 ━━━━━━━━━━━━━━━━━━━━ 1s 10ms/step - loss: 0.0164 - val_loss: 0.0160
Epoch 36/50
147/147 ━━━━━━━━━━━━━━━━━━━━ 3s 11ms/step - loss: 0.0153 - val_loss: 0.0160
Epoch 37/50
[illegible]
```

```
147/147 ━━━━━━━━━━━━━━━━━━━━ 2s 10ms/step - loss: 0.0157 - val_loss: 0.0160
Epoch 38/50
147/147 ━━━━━━━━━━━━━━━━━━━━ 2s 15ms/step - loss: 0.0157 - val_loss: 0.0160
Epoch 39/50
147/147 ━━━━━━━━━━━━━━━━━━━━ 2s 10ms/step - loss: 0.0156 - val_loss: 0.0159
Epoch 40/50
147/147 ━━━━━━━━━━━━━━━━━━━━ 2s 10ms/step - loss: 0.0151 - val_loss: 0.0159
Epoch 41/50
147/147 ━━━━━━━━━━━━━━━━━━━━ 3s 11ms/step - loss: 0.0156 - val_loss: 0.0159
Epoch 42/50
147/147 ━━━━━━━━━━━━━━━━━━━━ 1s 9ms/step - loss: 0.0166 - val_loss: 0.0160
Epoch 43/50
147/147 ━━━━━━━━━━━━━━━━━━━━ 2s 11ms/step - loss: 0.0153 - val_loss: 0.0160
Epoch 44/50
147/147 ━━━━━━━━━━━━━━━━━━━━ 3s 14ms/step - loss: 0.0156 - val_loss: 0.0160
Epoch 45/50
147/147 ━━━━━━━━━━━━━━━━━━━━ 2s 9ms/step - loss: 0.0161 - val_loss: 0.0159
Epoch 46/50
147/147 ━━━━━━━━━━━━━━━━━━━━ 1s 10ms/step - loss: 0.0149 - val_loss: 0.0159
Epoch 47/50
147/147 ━━━━━━━━━━━━━━━━━━━━ 1s 9ms/step - loss: 0.0157 - val_loss: 0.0160
Epoch 48/50
147/147 ━━━━━━━━━━━━━━━━━━━━ 1s 10ms/step - loss: 0.0159 - val_loss: 0.0160
Epoch 49/50
147/147 ━━━━━━━━━━━━━━━━━━━━ 3s 10ms/step - loss: 0.0156 - val_loss: 0.0159
Epoch 50/50
147/147 ━━━━━━━━━━━━━━━━━━━━ 2s 11ms/step - loss: 0.0158 - val_loss: 0.0160
74/74 ━━━━━━━━━━━━━━━━━━━━ 1s 10ms/step
```

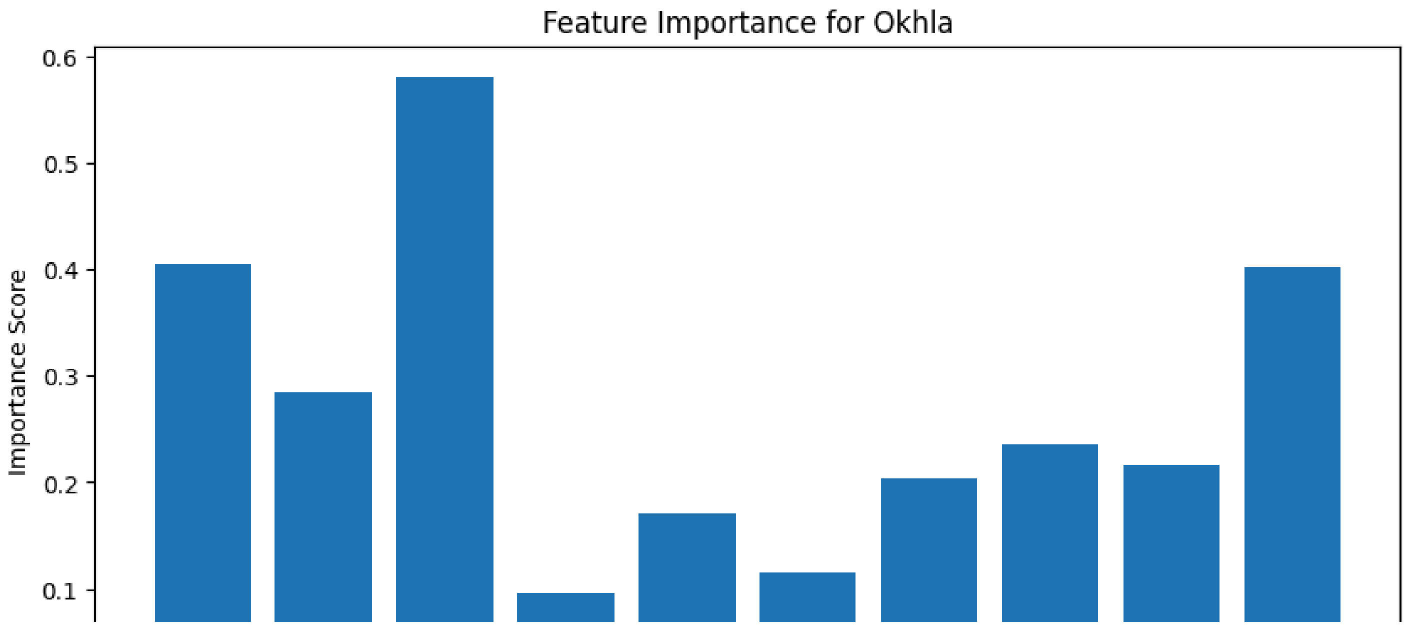

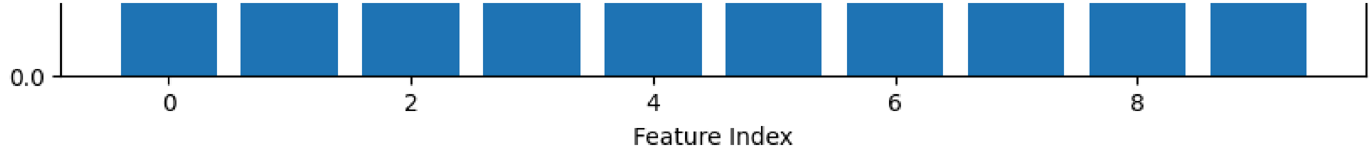

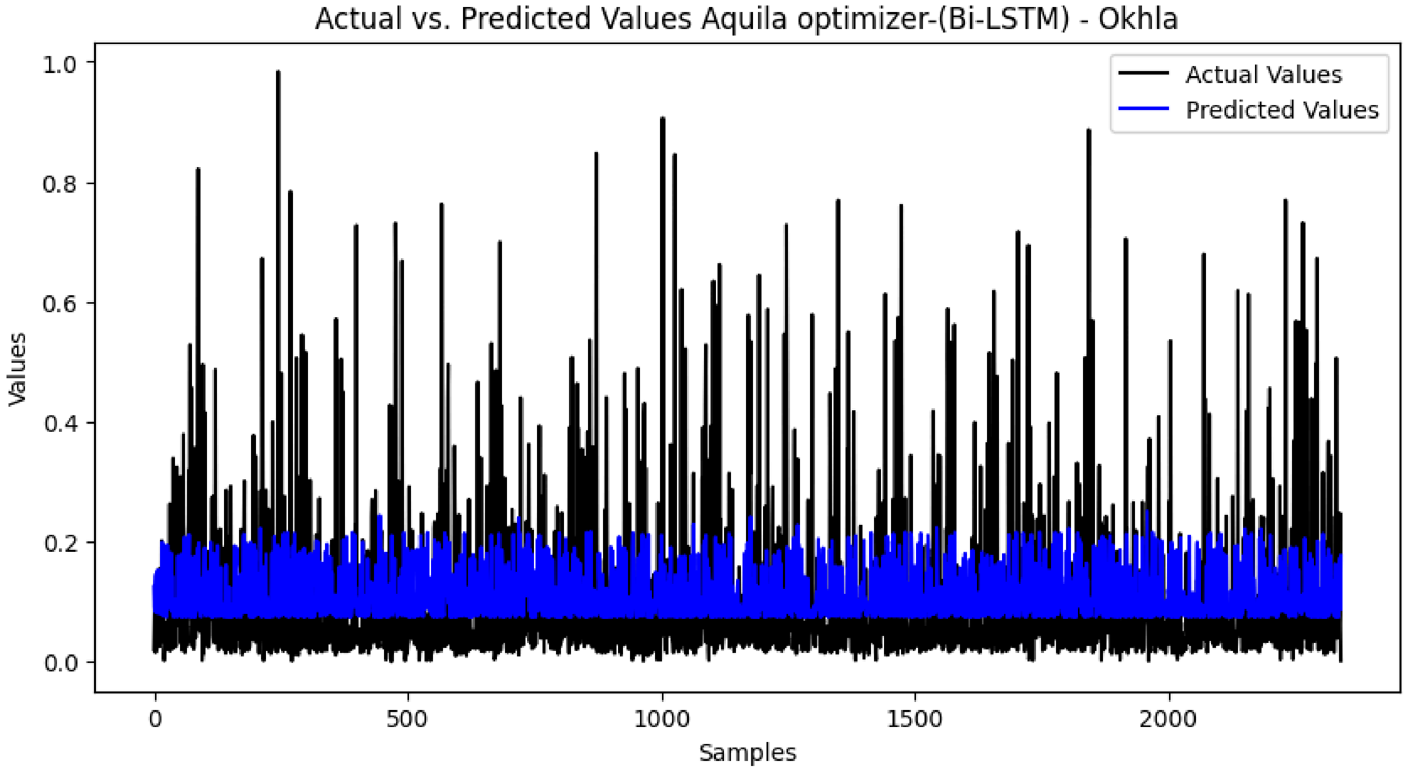


```
Final Model Evaluation Across Stations:

Station: AshokVihar
MSE: 0.0102
MAE: 0.0631
RMSE: 0.1011
R² Score: 0.0616

Station: DCStadium
MSE: 0.0194
MAE: 0.0923
RMSE: 0.1393
R² Score: 0.6198

Station: DwarkaSec8
MSE: 0.0008
```

```
MSE: 0.[illegible]
MAE: 0.0179
RMSE: 0.0276
R² Score: 0.9395

Station: NehruNagar
MSE: 0.0239
MAE: 0.1123
RMSE: 0.1547
R² Score: 0.2282

Station: Najafgarh
MSE: 0.0013
MAE: 0.0218
RMSE: 0.0357
R² Score: 0.6115

Station: Okhla
MSE: 0.0160
MAE: 0.0833
RMSE: 0.1266
R² Score: 0.0504
```

Start coding or generate with AI.

Arithmetic Optimizer

```
import numpy as np
import pandas as pd
import matplotlib.pyplot as plt
from sklearn.decomposition import PCA
from sklearn.preprocessing import StandardScaler, MinMaxScaler
from sklearn.model_selection import train_test_split
from sklearn.metrics import mean_squared_error, mean_absolute_error, r2_score
import tensorflow as tf
from tensorflow.keras.models import Sequential
from tensorflow.keras.layers import LSTM, Dense, Bidirectional
import pywt

# ============================
# Arithmetic Optimizer
# ============================
class ArithmeticOptimizer:
    def __init__(self, objective_function, lower_bound, upper_bound, population_size, iterations):
        self.objective_function = objective_function
        self.lower_bound = np.array(lower_bound)
        self.upper_bound = np.array(upper_bound)
        self.population_size = population_size
        self.iterations = iterations
        self.population = np.random.uniform(low=self.lower_bound, high=self.upper_bound,
                                            size=(population_size, len(lower_bound)))
        self.best_solution = None
        self.best_fitness = float('inf')

    def optimize(self):
        for _ in range(self.iterations):
            for i in range(self.population_size):
                partner_idx = np.random.randint(self.population_size)
                partner = self.population[partner_idx]
                candidate_solution = (self.population[i] + partner) / 2
                candidate_solution = np.clip(candidate_solution, self.lower_bound, self.upper_bound)
                fitness = self.objective_function(candidate_solution)

                if fitness < self.best_fitness:
```

```
                    self.best_fitness = fitness
                    self.best_solution = candidate_solution

        return self.best_solution

# ============================
# Feature Extraction
# ============================
def extract_wavelet_features(X, wavelet='db4', level=3, num_features=50):
    features = []
    for sample in X:
        coeffs = pywt.wavedec(sample, wavelet, level=level)
        flattened_coeffs = np.concatenate([c.flatten() for c in coeffs])
        features.append(flattened_coeffs[:num_features])
    return np.array(features)

def apply_pca(X, n_components=10):
    scaler = StandardScaler()
    X_scaled = scaler.fit_transform(X)
    pca = PCA(n_components=n_components)
    return pca.fit_transform(X_scaled)

def extract_combined_features(X):
    X_wavelet = extract_wavelet_features(X)
    X_pca = apply_pca(X_wavelet)
    return X_pca

# ============================
# Bi-LSTM Model Definition
# ============================
def build_lstm_model(input_shape):
    model = Sequential([
        Bidirectional(LSTM(50, return_sequences=True, input_shape=input_shape)),
        Bidirectional(LSTM(50, return_sequences=False)),
        Dense(1)
    ])
    model.compile(optimizer='adam', loss='mean_squared_error')
    return model

# ============================
# Training & Evaluation
# ============================
def evaluate_model(X, y):
    if X.shape[1] == 0:
```

```
        raise ValueError("No features selected! Adjust feature selection threshold.")
    X_train, X_test, y_train, y_test = train_test_split(X, y, test_size=0.2, random_state=42)
    X_train = X_train.reshape(X_train.shape[0], X_train.shape[1], 1)
    X_test = X_test.reshape(X_test.shape[0], X_test.shape[1], 1)

    model = build_lstm_model((X_train.shape[1], 1))
    model.fit(X_train, y_train, epochs=20, batch_size=64, validation_data=(X_test, y_test), verbose=1)
    y_pred = model.predict(X_test)

    mse = mean_squared_error(y_test, y_pred)
    mae = mean_absolute_error(y_test, y_pred)
    rmse = np.sqrt(mse)
    r2 = r2_score(y_test, y_pred)

    return mse, mae, rmse, r2, y_test, y_pred

# ============================
# Multi-Station Processing
# ============================
stations = {
    'AshokVihar': '/content/AshokVihar_Hourly.csv',
    'DCStadium': '/content/DCStadium_Hourly.csv',
    'DwarkaSec8': '/content/DwarkaSec8_Hourly.csv',
    'NehruNagar': '/content/NehruNagar_Hourly.csv',
    'Najafgarh': '/content/Najafgarh_Hourly.csv',
    'Okhla': '/content/Okhla_Hourly.csv'
}

threshold = 0.40  # Feature selection threshold
results = {}

for station, file_path in stations.items():
    print(f"\nProcessing Station: {station}")

    # Load Data
    df = pd.read_csv(file_path)

    # Preprocessing
    scaler = MinMaxScaler()
    X_full = scaler.fit_transform(df.iloc[:, :-1].values)
    y = scaler.fit_transform(df.iloc[:, -1].values.reshape(-1, 1))

    # Feature Extraction
    X_extracted = extract_combined_features(X_full)
```

```
    # Feature Selection with Arithmetic Optimization
    objective_function = lambda x: np.sum(x**2)
    arith_optimizer = ArithmeticOptimizer(objective_function,
                                          lower_bound=[-1] * X_extracted.shape[1],
                                          upper_bound=[1] * X_extracted.shape[1],
                                          population_size=50,
                                          iterations=100)
    selected_features = arith_optimizer.optimize()

    # Select features above threshold
    X_selected = X_extracted[:, selected_features > threshold]

    # Final Model Evaluation
    X_final = X_selected if X_selected.shape[1] > 0 else X_extracted
    mse, mae, rmse, r2, y_test, y_pred = evaluate_model(X_final, y)

    # Store results
    results[station] = {"MSE": mse, "MAE": mae, "RMSE": rmse, "R² Score": r2}

    # Plot Feature Importance
    plt.figure(figsize=(10, 5))
    feature_importance = np.abs(selected_features)
    plt.bar(range(len(feature_importance)), feature_importance)
    plt.xlabel('Feature Index')
    plt.ylabel('Importance Score')
    plt.title(f'Feature Importance for {station}')
    plt.show()

    # Plot Actual vs Predicted
    plt.figure(figsize=(10, 5))
    plt.plot(y_test, label="Actual Values", color='black')
    plt.plot(y_pred, label="Predicted Values", color='blue')
    plt.xlabel('Samples')
    plt.ylabel('Values')
    plt.title(f'Actual vs. Predicted Values Arithmetic optimization-(Bi-LSTM) - {station}')
    plt.legend()
    plt.show()

# Print Final Results
print("\nFinal Model Evaluation Across Stations:")
for station, metrics in results.items():
    print(f"\nStation: {station}")
    for metric, value in metrics.items():
```

```
        print(f"{metric}: {value:.4f}")
```

```
Processing Station: AshokVihar
/usr/local/lib/python3.11/dist-packages/pywt/_multilevel.py:43: UserWarning: Level value of 3 is too high: all coefficients will experience boundary effe
  warnings.warn(
Epoch 1/20
/usr/local/lib/python3.11/dist-packages/keras/src/layers/rnn/rnn.py:200: UserWarning: Do not pass an `input_shape`/`input_dim` argument to a layer. When
  super().__init__(**kwargs)
147/147 ━━━━━━━━━━━━━━━━━━━━ 6s 16ms/step - loss: 0.0050 - val_loss: 0.0027
Epoch 2/20
147/147 ━━━━━━━━━━━━━━━━━━━━ 2s 14ms/step - loss: 0.0027 - val_loss: 0.0023
Epoch 3/20
147/147 ━━━━━━━━━━━━━━━━━━━━ 2s 12ms/step - loss: 0.0019 - val_loss: 0.0024
Epoch 4/20
147/147 ━━━━━━━━━━━━━━━━━━━━ 2s 12ms/step - loss: 0.0020 - val_loss: 0.0016
Epoch 5/20
147/147 ━━━━━━━━━━━━━━━━━━━━ 2s 11ms/step - loss: 0.0016 - val_loss: 0.0016
Epoch 6/20
147/147 ━━━━━━━━━━━━━━━━━━━━ 2s 12ms/step - loss: 0.0014 - val_loss: 0.0014
Epoch 7/20
147/147 ━━━━━━━━━━━━━━━━━━━━ 2s 10ms/step - loss: 0.0014 - val_loss: 0.0013
Epoch 8/20
147/147 ━━━━━━━━━━━━━━━━━━━━ 2s 14ms/step - loss: 0.0012 - val_loss: 0.0017
Epoch 9/20
147/147 ━━━━━━━━━━━━━━━━━━━━ 2s 14ms/step - loss: 0.0013 - val_loss: 0.0013
Epoch 10/20
147/147 ━━━━━━━━━━━━━━━━━━━━ 2s 12ms/step - loss: 0.0012 - val_loss: 0.0012
Epoch 11/20
147/147 ━━━━━━━━━━━━━━━━━━━━ 2s 12ms/step - loss: 0.0012 - val_loss: 0.0014
Epoch 12/20
147/147 ━━━━━━━━━━━━━━━━━━━━ 1s 10ms/step - loss: 0.0011 - val_loss: 0.0013
Epoch 13/20
147/147 ━━━━━━━━━━━━━━━━━━━━ 1s 10ms/step - loss: 0.0011 - val_loss: 0.0011
Epoch 14/20
147/147 ━━━━━━━━━━━━━━━━━━━━ 1s 10ms/step - loss: 0.0010 - val_loss: 0.0011
Epoch 15/20
147/147 ━━━━━━━━━━━━━━━━━━━━ 3s 15ms/step - loss: 0.0012 - val_loss: 0.0011
Epoch 16/20
147/147 ━━━━━━━━━━━━━━━━━━━━ 2s 10ms/step - loss: 0.0011 - val_loss: 9.8758e-04
Epoch 17/20
147/147 ━━━━━━━━━━━━━━━━━━━━ 3s 10ms/step - loss: 0.0010 - val_loss: 0.0012
Epoch 18/20
147/147 ━━━━━━━━━━━━━━━━━━━━ 2s 11ms/step - loss: 0.0010 - val_loss: 9.0494e-04
Epoch 19/20
147/147 ━━━━━━━━━━━━━━━━━━━━ 2s 10ms/step - loss: 9.1996e-04 - val_loss: 9.4763e-04
Epoch 20/20
147/147 ━━━━━━━━━━━━━━━━━━━━ 3s 14ms/step - loss: 9.7431e-04 - val_loss: 0.0010
74/74 ━━━━━━━━━━━━━━━━━━━━ 1s 8ms/step
```

Feature Importance for AshokVihar

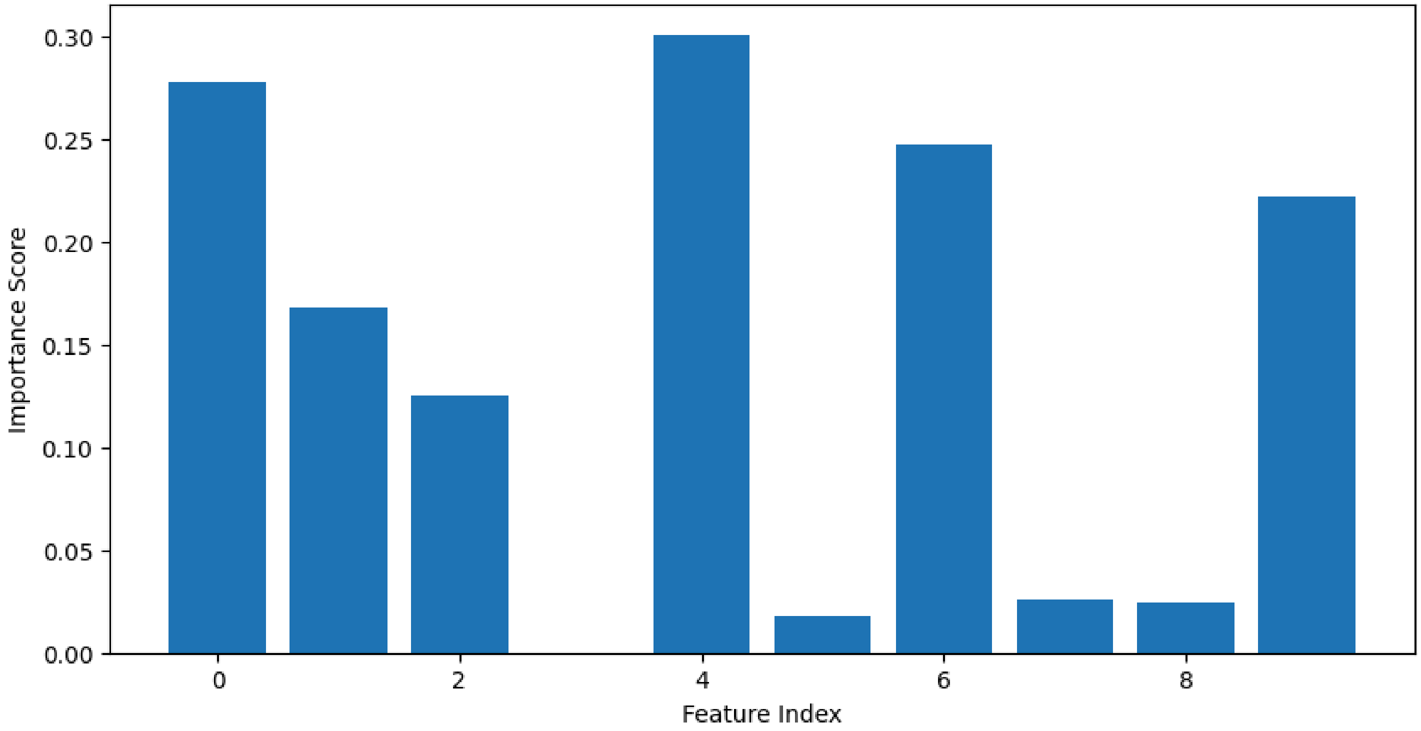
Importance Score
0.30
0.25
0.20
0.15
0.10
0.05
0.00
0
2
4
6
8
Feature Index

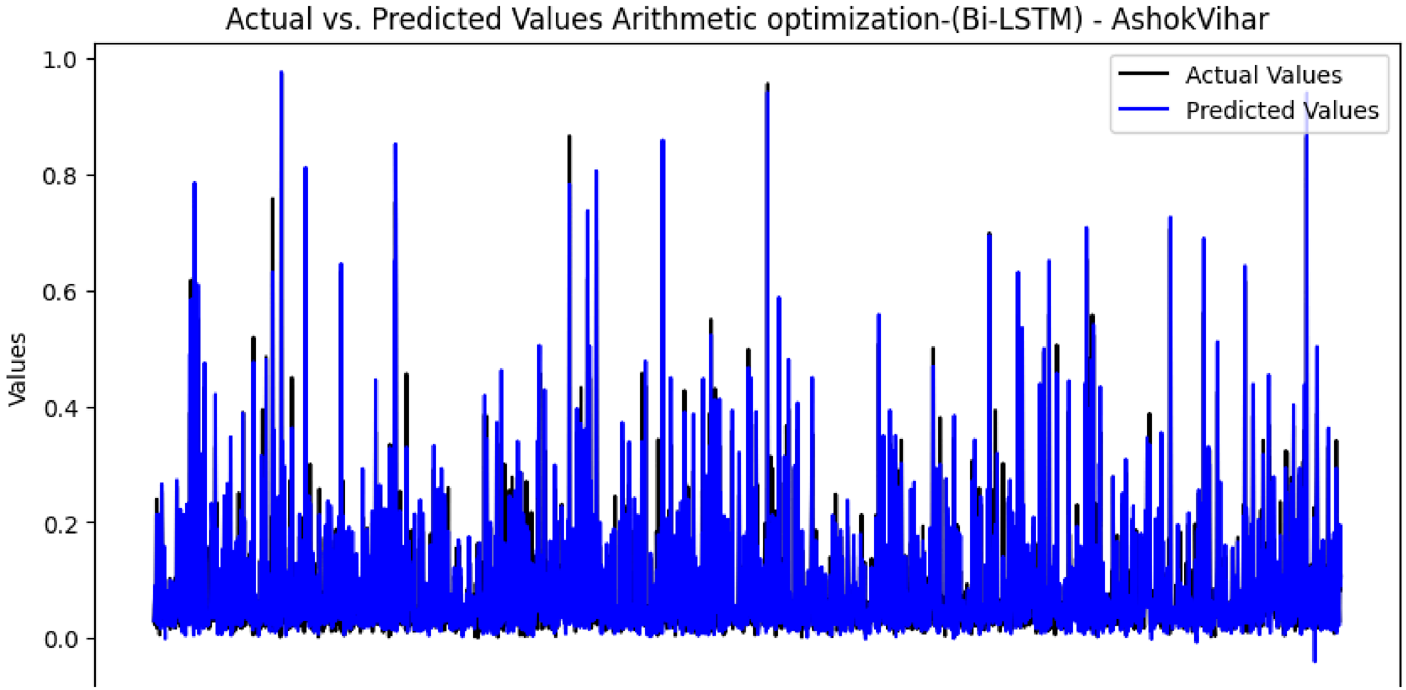
Actual vs. Predicted Values Arithmetic optimization-(Bi-LSTM) - AshokVihar
Values
1.0
0.8
0.6
0.4
0.2
0.0
Actual Values
Predicted Values

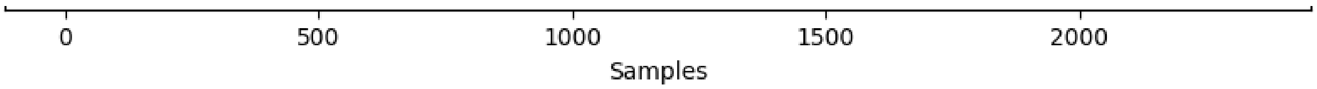


```
Processing Station: DCStadium
/usr/local/lib/python3.11/dist-packages/pywt/_multilevel.py:43: UserWarning: Level value of 3 is too high: all coefficients will experience boundary effe
  warnings.warn(
Epoch 1/20
/usr/local/lib/python3.11/dist-packages/keras/src/layers/rnn/rnn.py:200: UserWarning: Do not pass an `input_shape`/`input_dim` argument to a layer. When
  super().__init__(**kwargs)
147/147 ━━━━━━━━━━━━━━━━━━━━ 6s 14ms/step - loss: 0.0230 - val_loss: 0.0099
Epoch 2/20
147/147 ━━━━━━━━━━━━━━━━━━━━ 2s 11ms/step - loss: 0.0073 - val_loss: 0.0029
Epoch 3/20
147/147 ━━━━━━━━━━━━━━━━━━━━ 3s 14ms/step - loss: 0.0029 - val_loss: 0.0021
Epoch 4/20
147/147 ━━━━━━━━━━━━━━━━━━━━ 1s 10ms/step - loss: 0.0023 - val_loss: 0.0022
Epoch 5/20
147/147 ━━━━━━━━━━━━━━━━━━━━ 3s 10ms/step - loss: 0.0021 - val_loss: 0.0019
Epoch 6/20
147/147 ━━━━━━━━━━━━━━━━━━━━ 3s 10ms/step - loss: 0.0022 - val_loss: 0.0018
Epoch 7/20
147/147 ━━━━━━━━━━━━━━━━━━━━ 3s 11ms/step - loss: 0.0020 - val_loss: 0.0018
Epoch 8/20
147/147 ━━━━━━━━━━━━━━━━━━━━ 3s 14ms/step - loss: 0.0020 - val_loss: 0.0021
Epoch 9/20
147/147 ━━━━━━━━━━━━━━━━━━━━ 2s 11ms/step - loss: 0.0020 - val_loss: 0.0018
Epoch 10/20
147/147 ━━━━━━━━━━━━━━━━━━━━ 2s 10ms/step - loss: 0.0018 - val_loss: 0.0018
Epoch 11/20
147/147 ━━━━━━━━━━━━━━━━━━━━ 2s 11ms/step - loss: 0.0017 - val_loss: 0.0015
Epoch 12/20
147/147 ━━━━━━━━━━━━━━━━━━━━ 2s 11ms/step - loss: 0.0017 - val_loss: 0.0014
Epoch 13/20
147/147 ━━━━━━━━━━━━━━━━━━━━ 2s 11ms/step - loss: 0.0016 - val_loss: 0.0016
Epoch 14/20
147/147 ━━━━━━━━━━━━━━━━━━━━ 3s 12ms/step - loss: 0.0017 - val_loss: 0.0014
Epoch 15/20
147/147 ━━━━━━━━━━━━━━━━━━━━ 2s 10ms/step - loss: 0.0016 - val_loss: 0.0014
Epoch 16/20
147/147 ━━━━━━━━━━━━━━━━━━━━ 3s 11ms/step - loss: 0.0016 - val_loss: 0.0014
Epoch 17/20
147/147 ━━━━━━━━━━━━━━━━━━━━ 2s 11ms/step - loss: 0.0016 - val_loss: 0.0014
Epoch 18/20
147/147 ━━━━━━━━━━━━━━━━━━━━ 1s 10ms/step - loss: 0.0014 - val_loss: 0.0012
Epoch 19/20
147/147 ━━━━━━━━━━━━━━━━━━━━ 1s 10ms/step - loss: 0.0013 - val_loss: 0.0015
Epoch 20/20
147/147 ━━━━━━━━━━━━━━━━━━━━ 2s 13ms/step - loss: 0.0014 - val_loss: 0.0012
```

```
74/74 ━━━━━━━━━━━━━━━━━━━━ 1s 8ms/step
```

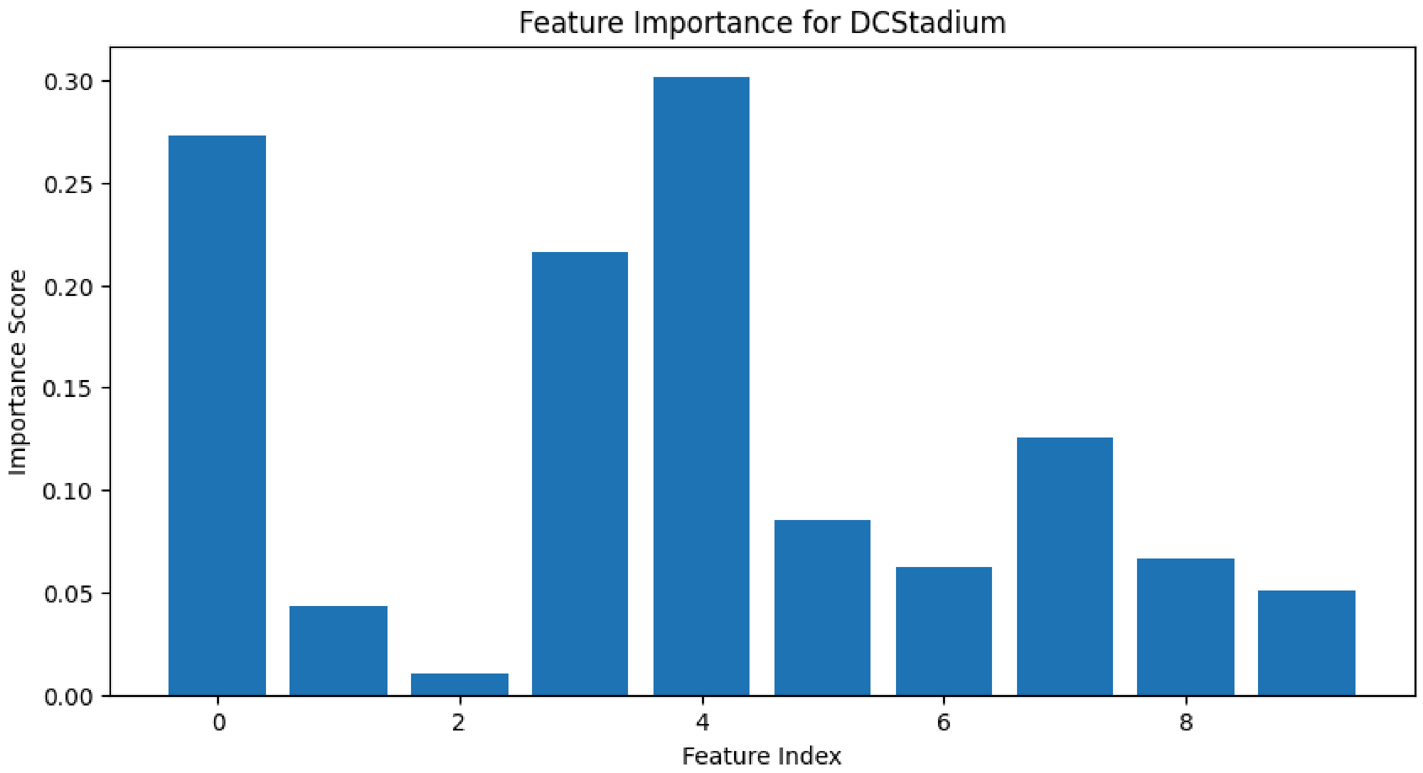

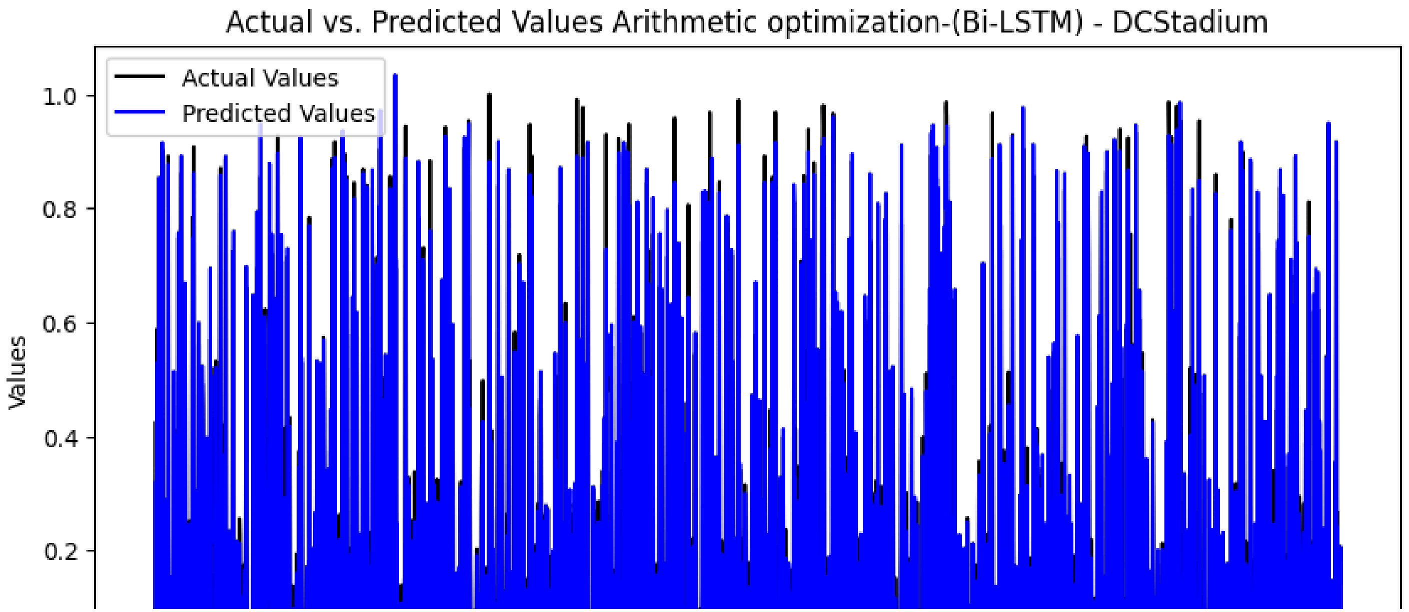

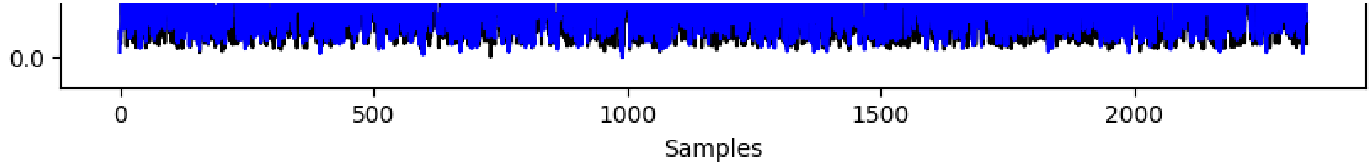


```
Processing Station: DwarkaSec8
/usr/local/lib/python3.11/dist-packages/pywt/_multilevel.py:43: UserWarning: Level value of 3 is too high: all coefficients will experience boundary effe
  warnings.warn(
Epoch 1/20
/usr/local/lib/python3.11/dist-packages/keras/src/layers/rnn/rnn.py:200: UserWarning: Do not pass an `input_shape`/`input_dim` argument to a layer. When
  super().__init__(**kwargs)
147/147 ━━━━━━━━━━━━━━━━━━━━ 6s 14ms/step - loss: 0.0079 - val_loss: 0.0036
Epoch 2/20
147/147 ━━━━━━━━━━━━━━━━━━━━ 2s 11ms/step - loss: 0.0031 - val_loss: 0.0020
Epoch 3/20
147/147 ━━━━━━━━━━━━━━━━━━━━ 3s 13ms/step - loss: 0.0018 - val_loss: 0.0016
Epoch 4/20
147/147 ━━━━━━━━━━━━━━━━━━━━ 2s 11ms/step - loss: 0.0016 - val_loss: 0.0015
Epoch 5/20
147/147 ━━━━━━━━━━━━━━━━━━━━ 2s 10ms/step - loss: 0.0015 - val_loss: 0.0014
Epoch 6/20
147/147 ━━━━━━━━━━━━━━━━━━━━ 3s 11ms/step - loss: 0.0014 - val_loss: 0.0014
Epoch 7/20
147/147 ━━━━━━━━━━━━━━━━━━━━ 1s 10ms/step - loss: 0.0014 - val_loss: 0.0013
Epoch 8/20
147/147 ━━━━━━━━━━━━━━━━━━━━ 3s 14ms/step - loss: 0.0013 - val_loss: 0.0013
Epoch 9/20
147/147 ━━━━━━━━━━━━━━━━━━━━ 2s 10ms/step - loss: 0.0012 - val_loss: 0.0013
Epoch 10/20
147/147 ━━━━━━━━━━━━━━━━━━━━ 3s 10ms/step - loss: 0.0012 - val_loss: 0.0013
Epoch 11/20
147/147 ━━━━━━━━━━━━━━━━━━━━ 1s 10ms/step - loss: 0.0011 - val_loss: 0.0012
Epoch 12/20
147/147 ━━━━━━━━━━━━━━━━━━━━ 3s 10ms/step - loss: 0.0012 - val_loss: 0.0011
Epoch 13/20
147/147 ━━━━━━━━━━━━━━━━━━━━ 2s 11ms/step - loss: 0.0011 - val_loss: 0.0011
Epoch 14/20
147/147 ━━━━━━━━━━━━━━━━━━━━ 2s 15ms/step - loss: 0.0010 - val_loss: 0.0012
Epoch 15/20
147/147 ━━━━━━━━━━━━━━━━━━━━ 2s 11ms/step - loss: 0.0011 - val_loss: 0.0012
Epoch 16/20
147/147 ━━━━━━━━━━━━━━━━━━━━ 1s 10ms/step - loss: 9.8918e-04 - val_loss: 0.0011
Epoch 17/20
147/147 ━━━━━━━━━━━━━━━━━━━━ 2s 11ms/step - loss: 9.2680e-04 - val_loss: 0.0012
Epoch 18/20
147/147 ━━━━━━━━━━━━━━━━━━━━ 1s 10ms/step - loss: 9.5896e-04 - val_loss: 9.6640e-04
Epoch 19/20
[illegible]
```

```
147/147 ━━━━━━━━━━━━━━━━━━━━ 3s 11ms/step - loss: 9.0210e-04 - val_loss: 9.7973e-04
Epoch 20/20
147/147 ━━━━━━━━━━━━━━━━━━━━ 3s 14ms/step - loss: 9.4187e-04 - val_loss: 9.8704e-04
74/74 ━━━━━━━━━━━━━━━━━━━━ 1s 7ms/step
```

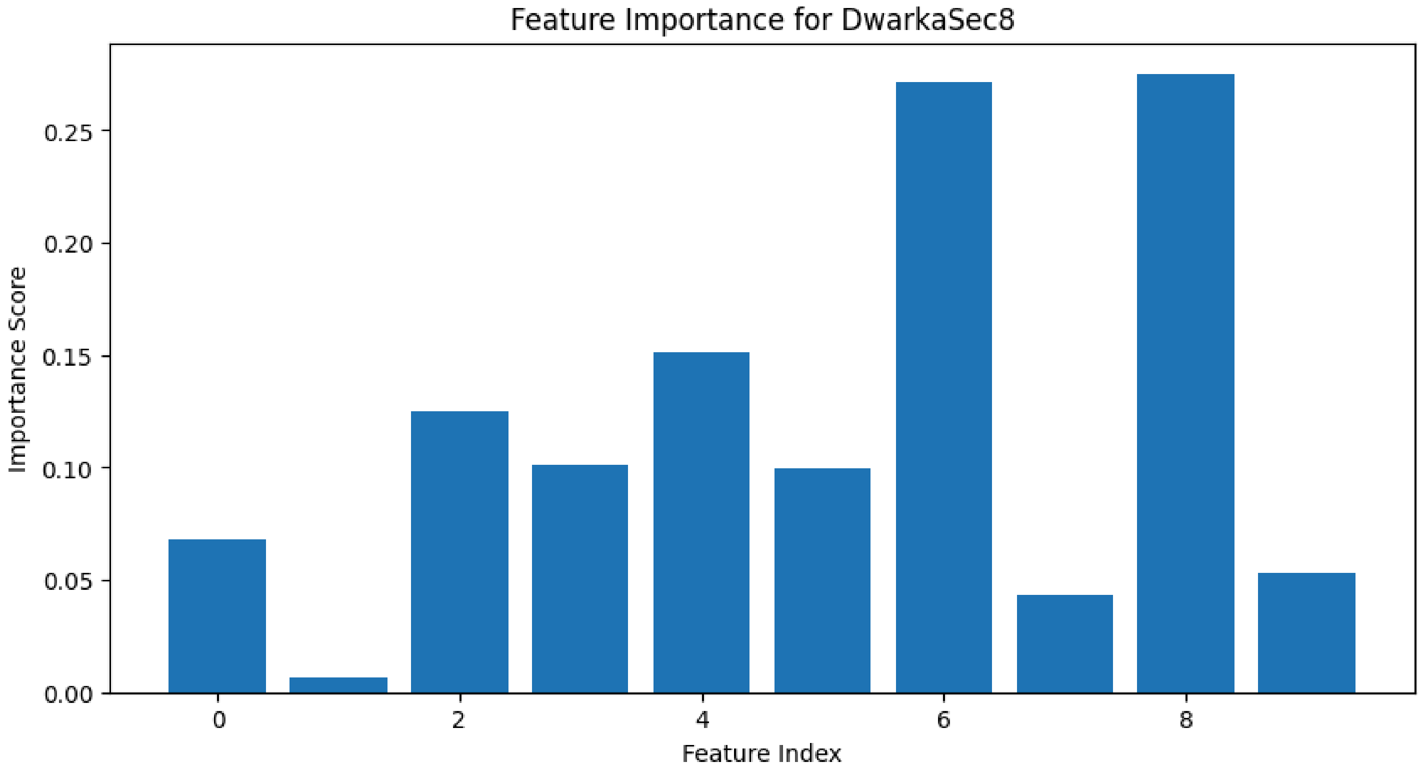

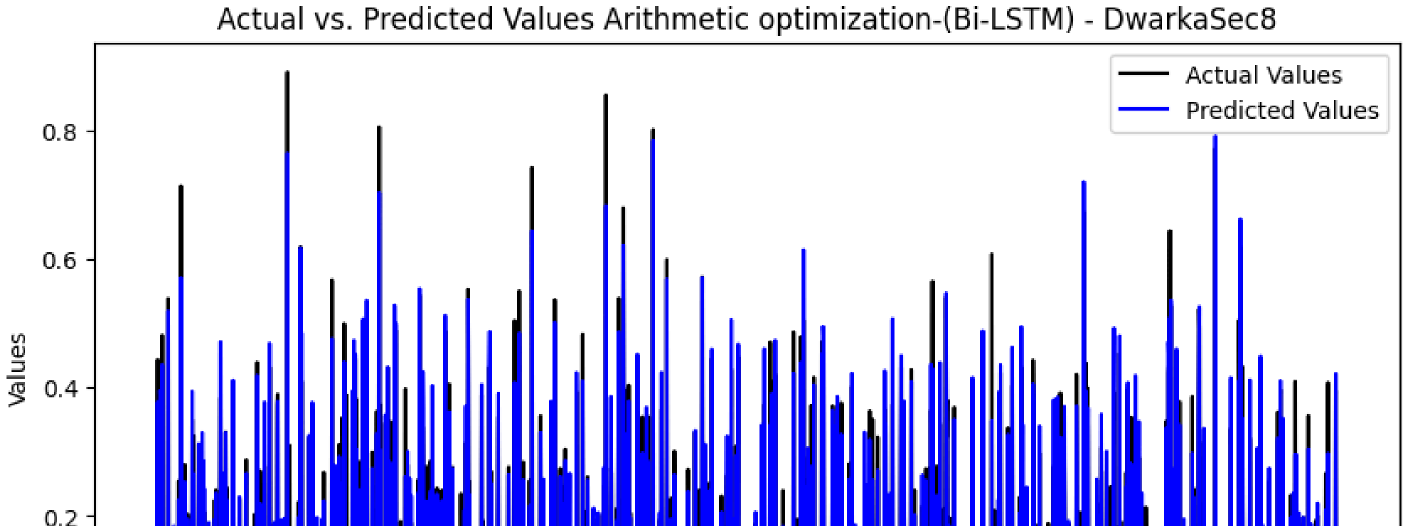

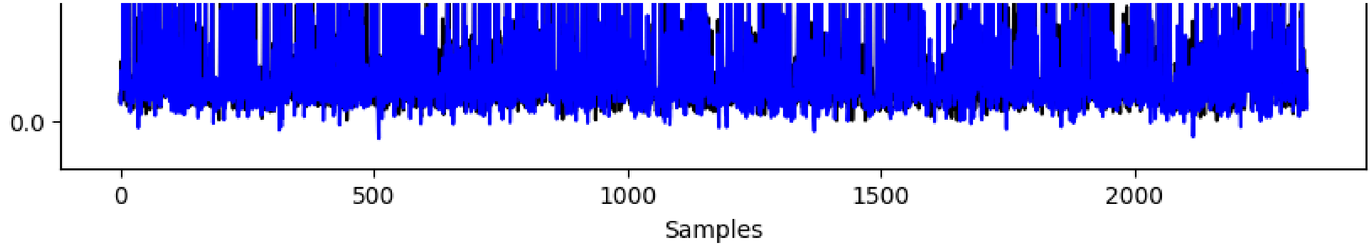


```
Processing Station: NehruNagar
/usr/local/lib/python3.11/dist-packages/pywt/_multilevel.py:43: UserWarning: Level value of 3 is too high: all coefficients will experience boundary effe
  warnings.warn(
Epoch 1/20
/usr/local/lib/python3.11/dist-packages/keras/src/layers/rnn/rnn.py:200: UserWarning: Do not pass an `input_shape`/`input_dim` argument to a layer. When
  super().__init__(**kwargs)
147/147 ━━━━━━━━━━━━━━━━━━━━ 6s 14ms/step - loss: 0.0127 - val_loss: 0.0041
Epoch 2/20
147/147 ━━━━━━━━━━━━━━━━━━━━ 1s 10ms/step - loss: 0.0037 - val_loss: 0.0025
Epoch 3/20
147/147 ━━━━━━━━━━━━━━━━━━━━ 3s 15ms/step - loss: 0.0023 - val_loss: 0.0023
Epoch 4/20
147/147 ━━━━━━━━━━━━━━━━━━━━ 2s 10ms/step - loss: 0.0020 - val_loss: 0.0019
Epoch 5/20
147/147 ━━━━━━━━━━━━━━━━━━━━ 2s 10ms/step - loss: 0.0018 - val_loss: 0.0019
Epoch 6/20
147/147 ━━━━━━━━━━━━━━━━━━━━ 3s 11ms/step - loss: 0.0016 - val_loss: 0.0019
Epoch 7/20
147/147 ━━━━━━━━━━━━━━━━━━━━ 3s 11ms/step - loss: 0.0016 - val_loss: 0.0017
Epoch 8/20
147/147 ━━━━━━━━━━━━━━━━━━━━ 3s 13ms/step - loss: 0.0016 - val_loss: 0.0018
Epoch 9/20
147/147 ━━━━━━━━━━━━━━━━━━━━ 2s 14ms/step - loss: 0.0015 - val_loss: 0.0017
Epoch 10/20
147/147 ━━━━━━━━━━━━━━━━━━━━ 1s 10ms/step - loss: 0.0015 - val_loss: 0.0016
Epoch 11/20
147/147 ━━━━━━━━━━━━━━━━━━━━ 1s 10ms/step - loss: 0.0014 - val_loss: 0.0015
Epoch 12/20
147/147 ━━━━━━━━━━━━━━━━━━━━ 2s 11ms/step - loss: 0.0014 - val_loss: 0.0015
Epoch 13/20
147/147 ━━━━━━━━━━━━━━━━━━━━ 2s 10ms/step - loss: 0.0012 - val_loss: 0.0015
Epoch 14/20
147/147 ━━━━━━━━━━━━━━━━━━━━ 2s 10ms/step - loss: 0.0013 - val_loss: 0.0014
Epoch 15/20
147/147 ━━━━━━━━━━━━━━━━━━━━ 2s 13ms/step - loss: 0.0011 - val_loss: 0.0012
Epoch 16/20
147/147 ━━━━━━━━━━━━━━━━━━━━ 2s 10ms/step - loss: 0.0011 - val_loss: 0.0013
Epoch 17/20
147/147 ━━━━━━━━━━━━━━━━━━━━ 2s 10ms/step - loss: 0.0011 - val_loss: 0.0012
Epoch 18/20
```

```
Epoch 18/20
147/147 ━━━━━━━━━━━━━━━━━━━━ 1s 10ms/step - loss: 0.0010 - val_loss: 0.0010
Epoch 19/20
147/147 ━━━━━━━━━━━━━━━━━━━━ 2s 10ms/step - loss: 9.5542e-04 - val_loss: 0.0011
Epoch 20/20
147/147 ━━━━━━━━━━━━━━━━━━━━ 3s 11ms/step - loss: 9.7507e-04 - val_loss: 9.9488e-04
74/74 ━━━━━━━━━━━━━━━━━━━━ 1s 11ms/step
```

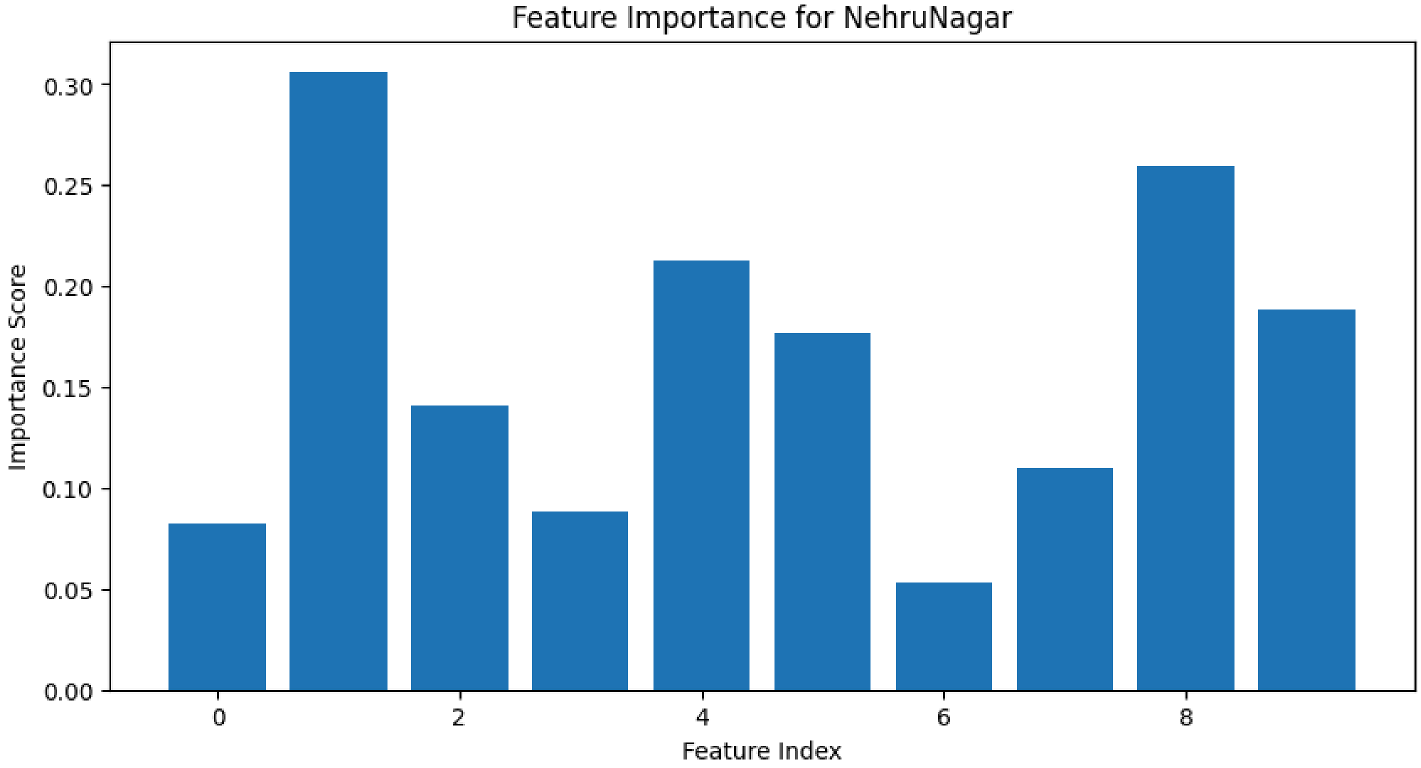

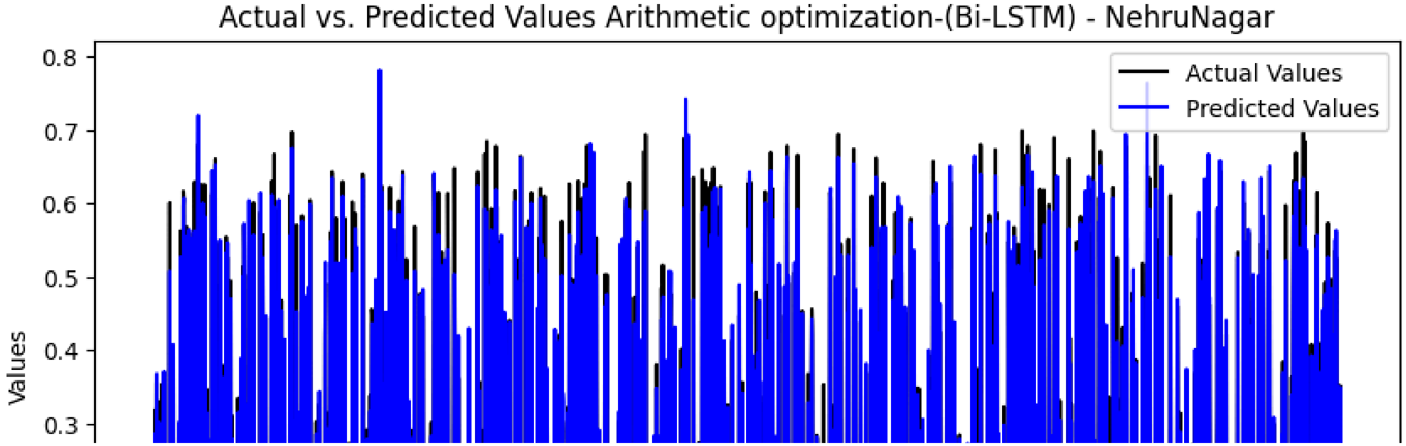

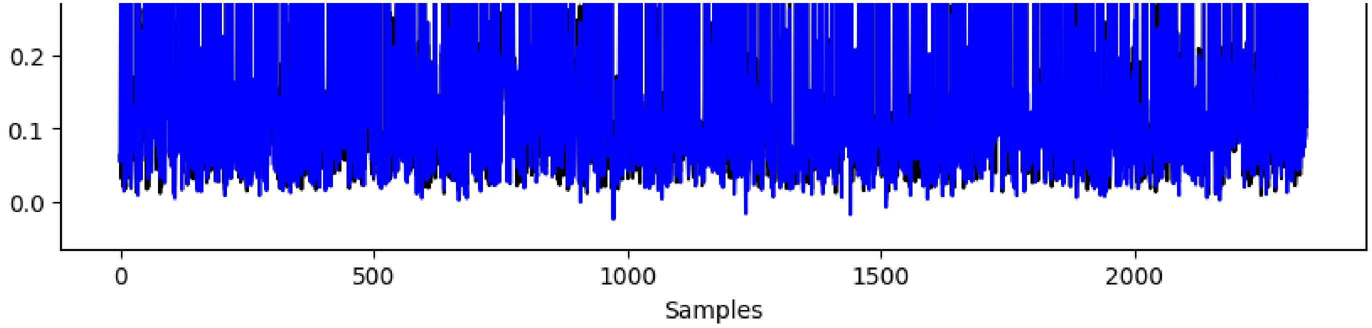


```
Processing Station: Najafgarh
/usr/local/lib/python3.11/dist-packages/pywt/_multilevel.py:43: UserWarning: Level value of 3 is too high: all coefficients will experience boundary effe
  warnings.warn(
Epoch 1/20
/usr/local/lib/python3.11/dist-packages/keras/src/layers/rnn/rnn.py:200: UserWarning: Do not pass an `input_shape`/`input_dim` argument to a layer. When
  super().__init__(**kwargs)
147/147 ━━━━━━━━━━━━━━━━━━━━ 6s 14ms/step - loss: 0.0033 - val_loss: 0.0019
Epoch 2/20
147/147 ━━━━━━━━━━━━━━━━━━━━ 2s 11ms/step - loss: 0.0021 - val_loss: 0.0015
Epoch 3/20
147/147 ━━━━━━━━━━━━━━━━━━━━ 3s 13ms/step - loss: 0.0018 - val_loss: 0.0013
Epoch 4/20
147/147 ━━━━━━━━━━━━━━━━━━━━ 2s 11ms/step - loss: 0.0013 - val_loss: 0.0013
Epoch 5/20
147/147 ━━━━━━━━━━━━━━━━━━━━ 2s 10ms/step - loss: 0.0015 - val_loss: 0.0012
Epoch 6/20
147/147 ━━━━━━━━━━━━━━━━━━━━ 2s 10ms/step - loss: 0.0012 - val_loss: 0.0011
Epoch 7/20
147/147 ━━━━━━━━━━━━━━━━━━━━ 3s 11ms/step - loss: 0.0012 - val_loss: 0.0014
Epoch 8/20
147/147 ━━━━━━━━━━━━━━━━━━━━ 3s 12ms/step - loss: 0.0011 - val_loss: 0.0013
Epoch 9/20
147/147 ━━━━━━━━━━━━━━━━━━━━ 2s 12ms/step - loss: 0.0011 - val_loss: 9.6148e-04
Epoch 10/20
147/147 ━━━━━━━━━━━━━━━━━━━━ 2s 10ms/step - loss: 0.0010 - val_loss: 0.0010
Epoch 11/20
147/147 ━━━━━━━━━━━━━━━━━━━━ 2s 10ms/step - loss: 8.5259e-04 - val_loss: 0.0011
Epoch 12/20
147/147 ━━━━━━━━━━━━━━━━━━━━ 2s 10ms/step - loss: 9.3634e-04 - val_loss: 8.3534e-04
Epoch 13/20
147/147 ━━━━━━━━━━━━━━━━━━━━ 3s 11ms/step - loss: 8.2936e-04 - val_loss: 8.3087e-04
Epoch 14/20
147/147 ━━━━━━━━━━━━━━━━━━━━ 2s 11ms/step - loss: 8.4756e-04 - val_loss: 8.6167e-04
Epoch 15/20
147/147 ━━━━━━━━━━━━━━━━━━━━ 3s 12ms/step - loss: 8.1735e-04 - val_loss: 8.0296e-04
Epoch 16/20
147/147 ━━━━━━━━━━━━━━━━━━━━ 2s 10ms/step - loss: 8.5990e-04 - val_loss: 8.3423e-04
```

```
Epoch 17/20
147/147 ━━━━━━━━━━━━━━━━━━━━ 3s 10ms/step - loss: 8.1763e-04 - val_loss: 7.2486e-04
Epoch 18/20
147/147 ━━━━━━━━━━━━━━━━━━━━ 3s 10ms/step - loss: 7.8481e-04 - val_loss: 7.0283e-04
Epoch 19/20
147/147 ━━━━━━━━━━━━━━━━━━━━ 3s 14ms/step - loss: 6.2719e-04 - val_loss: 7.1846e-04
Epoch 20/20
147/147 ━━━━━━━━━━━━━━━━━━━━ 2s 14ms/step - loss: 6.5571e-04 - val_loss: 6.1428e-04
74/74 ━━━━━━━━━━━━━━━━━━━━ 1s 8ms/step
```

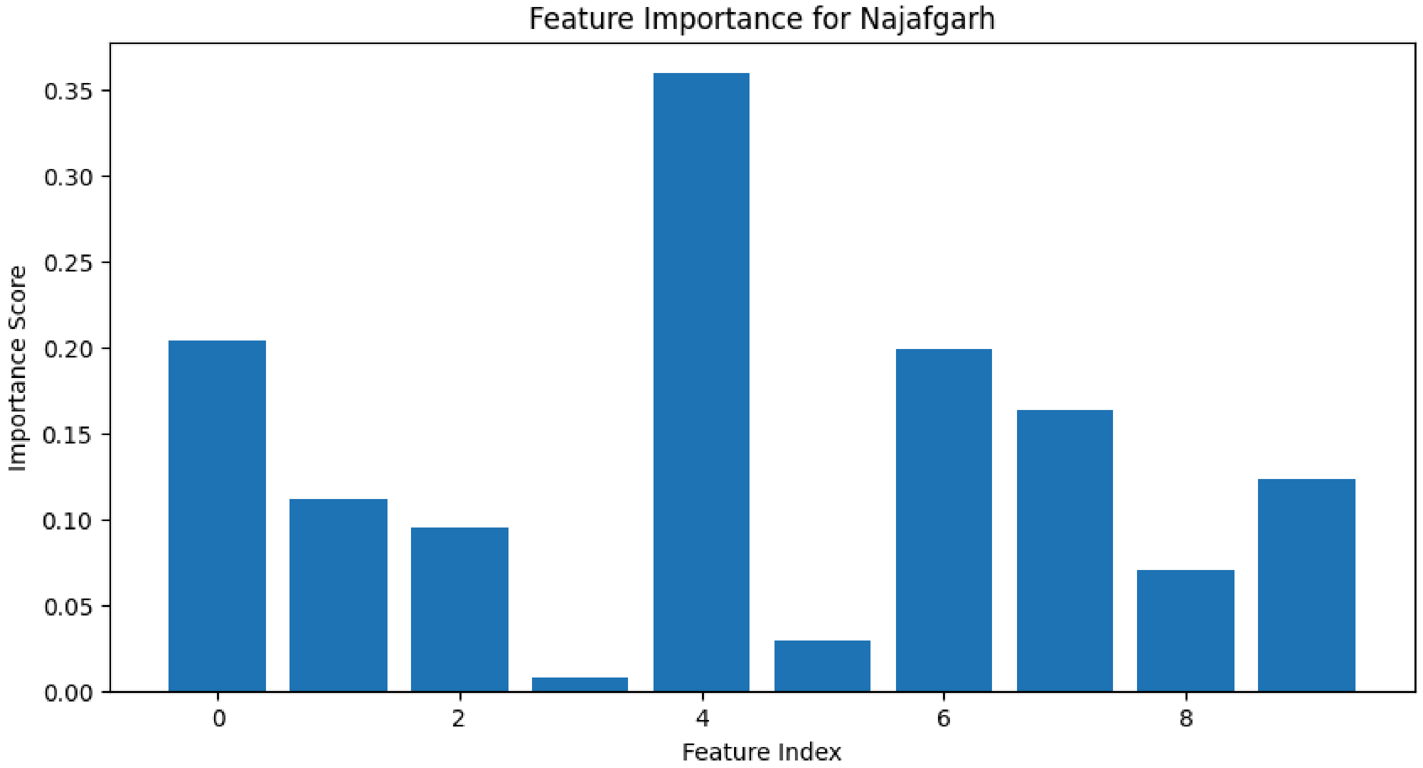

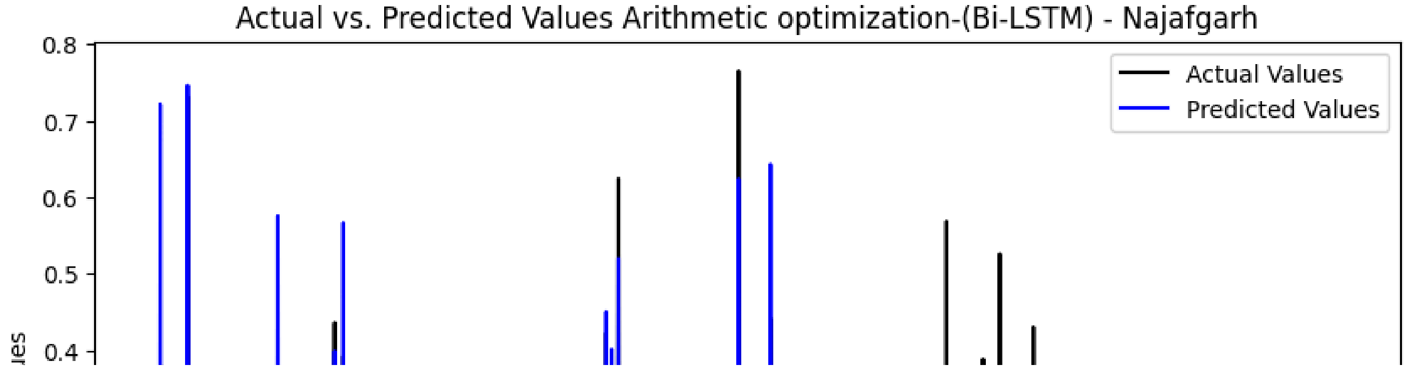

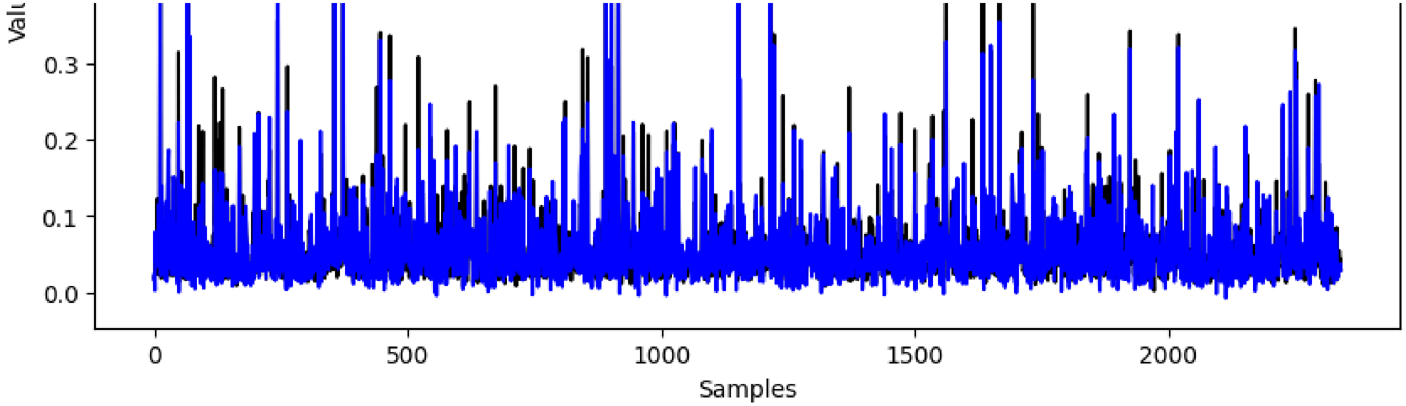


```
Processing Station: Okhla
/usr/local/lib/python3.11/dist-packages/pywt/_multilevel.py:43: UserWarning: Level value of 3 is too high: all coefficients will experience boundary effe
  warnings.warn(
Epoch 1/20
/usr/local/lib/python3.11/dist-packages/keras/src/layers/rnn/rnn.py:200: UserWarning: Do not pass an `input_shape`/`input_dim` argument to a layer. When
  super().__init__(**kwargs)
147/147 ━━━━━━━━━━━━━━━━━━━━ 6s 15ms/step - loss: 0.0129 - val_loss: 0.0068
Epoch 2/20
147/147 ━━━━━━━━━━━━━━━━━━━━ 2s 13ms/step - loss: 0.0063 - val_loss: 0.0041
Epoch 3/20
147/147 ━━━━━━━━━━━━━━━━━━━━ 2s 12ms/step - loss: 0.0037 - val_loss: 0.0026
Epoch 4/20
147/147 ━━━━━━━━━━━━━━━━━━━━ 2s 12ms/step - loss: 0.0026 - val_loss: 0.0021
Epoch 5/20
147/147 ━━━━━━━━━━━━━━━━━━━━ 2s 11ms/step - loss: 0.0022 - val_loss: 0.0022
Epoch 6/20
147/147 ━━━━━━━━━━━━━━━━━━━━ 3s 11ms/step - loss: 0.0019 - val_loss: 0.0016
Epoch 7/20
147/147 ━━━━━━━━━━━━━━━━━━━━ 3s 12ms/step - loss: 0.0017 - val_loss: 0.0016
Epoch 8/20
147/147 ━━━━━━━━━━━━━━━━━━━━ 2s 14ms/step - loss: 0.0015 - val_loss: 0.0015
Epoch 9/20
147/147 ━━━━━━━━━━━━━━━━━━━━ 2s 11ms/step - loss: 0.0014 - val_loss: 0.0013
Epoch 10/20
147/147 ━━━━━━━━━━━━━━━━━━━━ 3s 12ms/step - loss: 0.0014 - val_loss: 0.0012
Epoch 11/20
147/147 ━━━━━━━━━━━━━━━━━━━━ 2s 11ms/step - loss: 0.0012 - val_loss: 0.0011
Epoch 12/20
147/147 ━━━━━━━━━━━━━━━━━━━━ 3s 11ms/step - loss: 0.0011 - val_loss: 0.0010
Epoch 13/20
147/147 ━━━━━━━━━━━━━━━━━━━━ 3s 15ms/step - loss: 0.0010 - val_loss: 9.1861e-04
Epoch 14/20
147/147 ━━━━━━━━━━━━━━━━━━━━ 2s 11ms/step - loss: 0.0010 - val_loss: 8.4055e-04
Epoch 15/20
```

```
147/147 ━━━━━━━━━━━━━━━━━━━━ 2s 12ms/step - loss: 9.1043e-04 - val_loss: 8.0913e-04
Epoch 16/20
147/147 ━━━━━━━━━━━━━━━━━━━━ 3s 12ms/step - loss: 8.6322e-04 - val_loss: 8.1892e-04
Epoch 17/20
147/147 ━━━━━━━━━━━━━━━━━━━━ 3s 12ms/step - loss: 9.5316e-04 - val_loss: 8.8176e-04
Epoch 18/20
147/147 ━━━━━━━━━━━━━━━━━━━━ 3s 12ms/step - loss: 8.4786e-04 - val_loss: 8.1069e-04
Epoch 19/20
147/147 ━━━━━━━━━━━━━━━━━━━━ 2s 15ms/step - loss: 7.6565e-04 - val_loss: 7.5322e-04
Epoch 20/20
147/147 ━━━━━━━━━━━━━━━━━━━━ 2s 12ms/step - loss: 7.8140e-04 - val_loss: 7.5681e-04
74/74 ━━━━━━━━━━━━━━━━━━━━ 1s 8ms/step
```

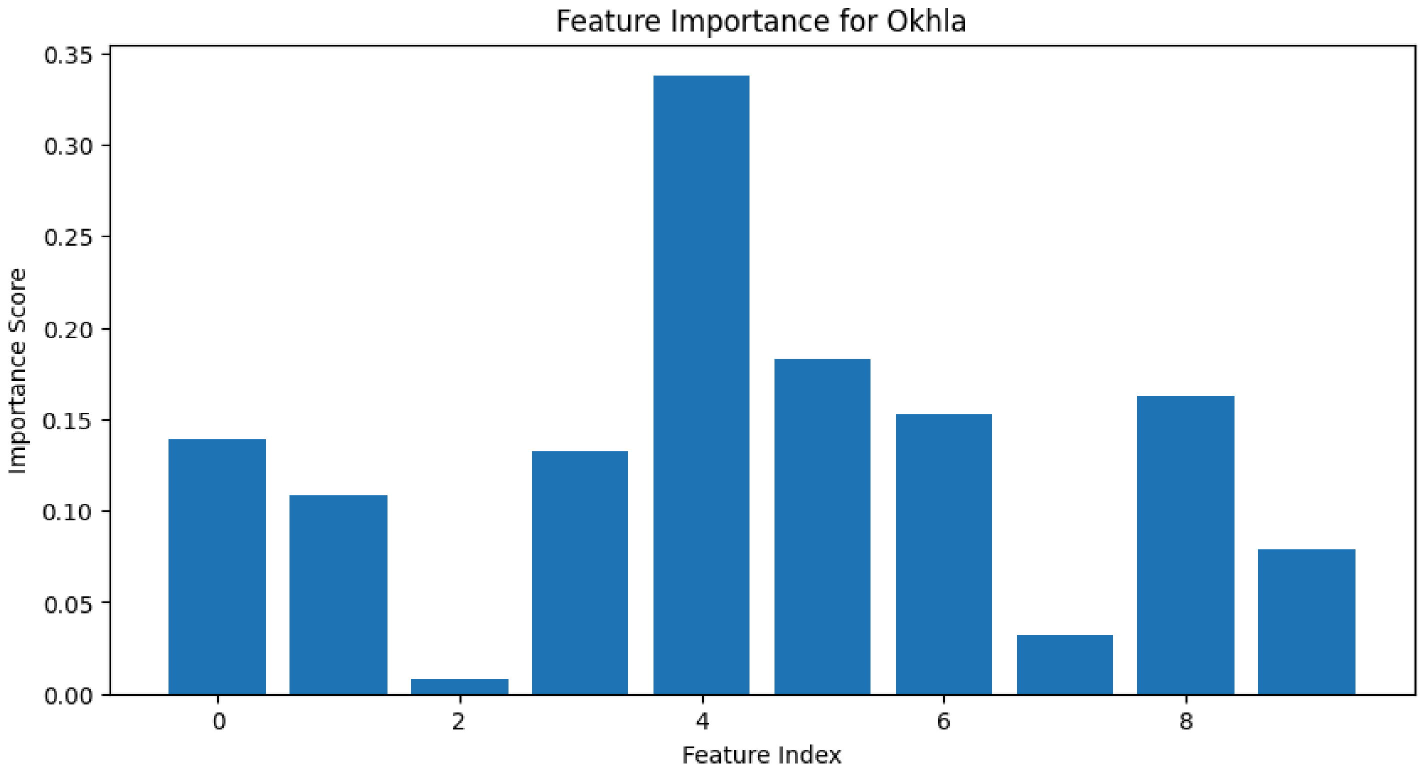

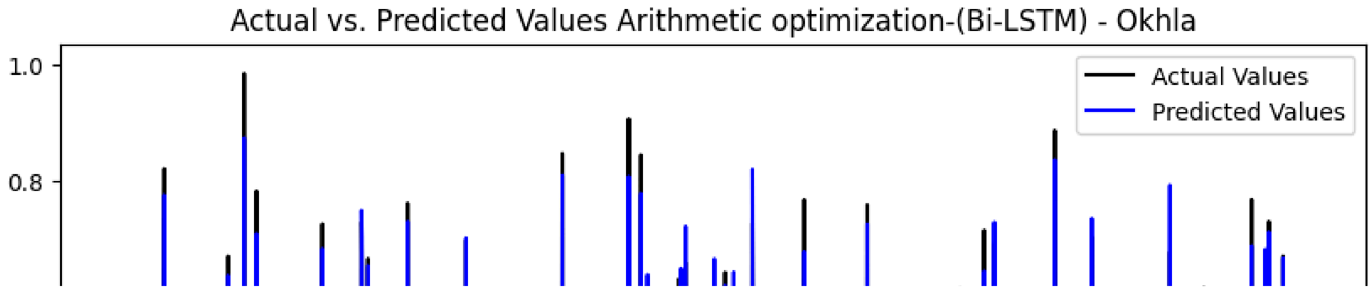

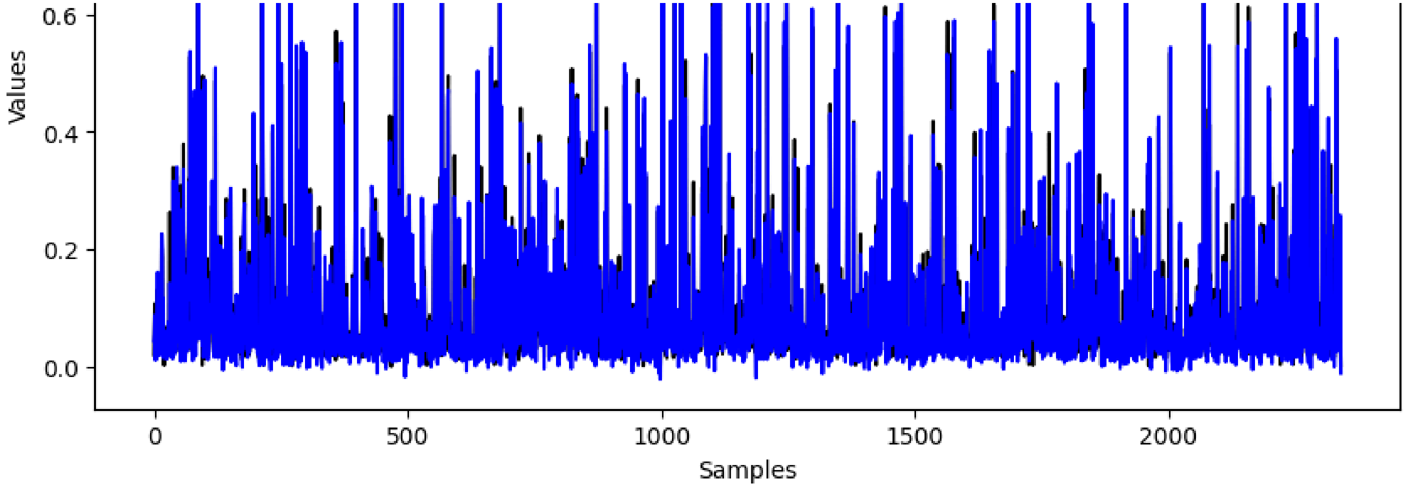


```
Final Model Evaluation Across Stations:

Station: AshokVihar
MSE: 0.0010
MAE: 0.0208
RMSE: 0.0322
R² Score: 0.9049

Station: DCStadium
MSE: 0.0012
MAE: 0.0242
RMSE: 0.0351
R² Score: 0.9758

Station: DwarkaSec8
MSE: 0.0010
MAE: 0.0211
RMSE: 0.0314
R² Score: 0.9218

Station: NehruNagar
MSE: 0.0010
MAE: 0.0225
RMSE: 0.0315
R² Score: 0.9679

Station: Najafgarh
MSE: 0.0006
MAE: 0.0152
RMSE: 0.0248
R² Score: 0.8128
```

```
Station: Okhla
MSE: 0.0008
MAE: 0.0189
RMSE: 0.0275
R² Score: 0.9552
```

Hybrid AOAOA Optimize with training and validation loss for each station

```
import numpy as np
import pandas as pd
import matplotlib.pyplot as plt
from sklearn.decomposition import PCA
from sklearn.preprocessing import StandardScaler, MinMaxScaler
from sklearn.model_selection import train_test_split
from sklearn.metrics import mean_squared_error, mean_absolute_error, r2_score
import tensorflow as tf
from tensorflow.keras.models import Sequential
from tensorflow.keras.layers import LSTM, Dense, Bidirectional
import pywt

# ============================
# Hybrid AOAOA Optimizer
# ============================
class HybridOptimizer:
    def __init__(self, objective_function, lower_bound, upper_bound, population_size, iterations):
        self.objective_function = objective_function
        self.lower_bound = np.array(lower_bound)
        self.upper_bound = np.array(upper_bound)
        self.population_size = population_size
        self.iterations = iterations
        self.population = np.random.uniform(low=self.lower_bound, high=self.upper_bound,
                                            size=(population_size, len(lower_bound)))
        self.best_solution = None
        self.best_fitness = float('inf')

    def optimize(self):
        for _ in range(self.iterations):
            for i in range(self.population_size):
                perturbation = np.random.uniform(-0.1, 0.1, size=self.population.shape[1])
                candidate_solution_aquila = self.population[i] + perturbation
                candidate_solution_aquila = np.clip(candidate_solution_aquila, self.lower_bound, self.upper_bound)
                fitness_aquila = self.objective_function(candidate_solution_aquila)

                if fitness_aquila < self.best_fitness:
                    self.best_fitness = fitness_aquila
                    self.best_solution = candidate_solution_aquila

            for i in range(self.population_size):
```

```
                partner_idx = np.random.randint(self.population_size)
                partner = self.population[partner_idx]
                candidate_solution_arithmetic = (self.population[i] + partner) / 2
                candidate_solution_arithmetic = np.clip(candidate_solution_arithmetic, self.lower_bound, self.upper_bound)
                fitness_arithmetic = self.objective_function(candidate_solution_arithmetic)

                if fitness_arithmetic < self.best_fitness:
                    self.best_fitness = fitness_arithmetic
                    self.best_solution = candidate_solution_arithmetic

        return self.best_solution

# ============================
# Feature Extraction
# ============================
def extract_wavelet_features(X, wavelet='db4', level=3, num_features=50):
    features = []
    for sample in X:
        coeffs = pywt.wavedec(sample, wavelet, level=level)
        flattened_coeffs = np.concatenate([c.flatten() for c in coeffs])
        features.append(flattened_coeffs[:num_features])
    return np.array(features)

def apply_pca(X, n_components=10):
    scaler = StandardScaler()
    X_scaled = scaler.fit_transform(X)
    pca = PCA(n_components=n_components)
    return pca.fit_transform(X_scaled)

def extract_combined_features(X):
    X_wavelet = extract_wavelet_features(X)
    X_pca = apply_pca(X_wavelet)
    return X_pca

# ============================
# Bi-LSTM Model Definition
# ============================
def build_lstm_model(input_shape):
    model = Sequential([
        Bidirectional(LSTM(50, return_sequences=True, input_shape=input_shape)),
        Bidirectional(LSTM(50, return_sequences=False)),
        Dense(1)
    ])
    model.compile(optimizer='adam', loss='mean_squared_error')
```

```
    return model

# ============================
# Training & Evaluation
# ============================
def evaluate_model(X, y, station_name):
    if X.shape[1] == 0:
        raise ValueError("No features selected! Adjust AOAOA feature selection.")
    X_train, X_test, y_train, y_test = train_test_split(X, y, test_size=0.2, random_state=42)
    X_train = X_train.reshape(X_train.shape[0], X_train.shape[1], 1)
    X_test = X_test.reshape(X_test.shape[0], X_test.shape[1], 1)

    model = build_lstm_model((X_train.shape[1], 1))
    history = model.fit(X_train, y_train, epochs=50, batch_size=64,
                        validation_data=(X_test, y_test), verbose=1)

    # Plot training vs validation loss
    plt.figure(figsize=(8, 5))
    plt.plot(history.history['loss'], label='Training Loss')
    plt.plot(history.history['val_loss'], label='Validation Loss')
    plt.title(f'Training vs Validation Loss - {station_name}')
    plt.xlabel('Epoch')
    plt.ylabel('Loss (MSE)')
    plt.legend()
    plt.grid(True)
    plt.tight_layout()
    plt.show()

    y_pred = model.predict(X_test)

    mse = mean_squared_error(y_test, y_pred)
    mae = mean_absolute_error(y_test, y_pred)
    rmse = np.sqrt(mse)
    r2 = r2_score(y_test, y_pred)

    return mse, mae, rmse, r2, y_test, y_pred

# ============================
# Multi-Station Processing
# ============================
stations = {
    'AshokVihar': '/content/AshokVihar_Hourly.csv',
    'DCStadium': '/content/DCStadium_Hourly.csv',
    'DwarkaSec8': '/content/DwarkaSec8_Hourly.csv',
```

```
    'NehruNagar': '/content/NehruNagar_Hourly.csv',
    'Najafgarh': '/content/Najafgarh_Hourly.csv',
    'Okhla': '/content/Okhla_Hourly.csv'
}

threshold = 0.40  # Adjust this value as needed
results = {}

for station, file_path in stations.items():
    print(f"\nProcessing Station: {station}")

    # Load Data
    df = pd.read_csv(file_path)

    # Preprocessing
    scaler = MinMaxScaler()
    X_full = scaler.fit_transform(df.iloc[:, :-1].values)
    y = scaler.fit_transform(df.iloc[:, -1].values.reshape(-1, 1))

    # Feature Extraction
    X_extracted = extract_combined_features(X_full)

    # Feature Selection with Hybrid AOAOA
    objective_function = lambda x: np.sum(x**2)
    hybrid_optimizer = HybridOptimizer(objective_function,
                                       lower_bound=[-1] * X_extracted.shape[1],
                                       upper_bound=[1] * X_extracted.shape[1],
                                       population_size=50,
                                       iterations=100)
    selected_features = hybrid_optimizer.optimize()

    # Select features above threshold
    X_selected = X_extracted[:, selected_features > threshold]

    # Final Model Evaluation
    X_final = X_selected if X_selected.shape[1] > 0 else X_extracted
    mse, mae, rmse, r2, y_test, y_pred = evaluate_model(X_final, y, station)

    # Store results
    results[station] = {"MSE": mse, "MAE": mae, "RMSE": rmse, "R² Score": r2}

    # Plot Feature Importance
    plt.figure(figsize=(10, 5))
    feature_importance = np.abs(selected_features)
```

```
    plt.bar(range(len(feature_importance)), feature_importance)
    plt.xlabel('Feature Index')
    plt.ylabel('Importance Score')
    plt.title(f'Feature Importance for {station}')
    plt.tight_layout()
    plt.show()

    # Plot Actual vs Predicted
    plt.figure(figsize=(10, 5))
    plt.plot(y_test, label="Actual Values", color='black')
    plt.plot(y_pred, label="Predicted Values", color='blue')
    plt.xlabel('Samples')
    plt.ylabel('Values')
    plt.title(f'Actual vs. Predicted Values (Bi-LSTM) - {station}')
    plt.legend()
    plt.tight_layout()
    plt.show()

# Print Final Results
print("\nFinal Model Evaluation Across Stations:")
for station, metrics in results.items():
    print(f"\nStation: {station}")
    for metric, value in metrics.items():
        print(f"{metric}: {value:.4f}")
```

```
Processing Station: AshokVihar
/usr/local/lib/python3.11/dist-packages/pywt/_multilevel.py:43: UserWarning: Level value of 3 is too high: all coefficients will experience boundary effe
  warnings.warn(
Epoch 1/50
/usr/local/lib/python3.11/dist-packages/keras/src/layers/rnn/rnn.py:200: UserWarning: Do not pass an `input_shape`/`input_dim` argument to a layer. When
  super().__init__(**kwargs)
147/147 ━━━━━━━━━━━━━━━━━━━━ 6s 14ms/step - loss: 0.0051 - val_loss: 0.0030
Epoch 2/50
147/147 ━━━━━━━━━━━━━━━━━━━━ 2s 13ms/step - loss: 0.0028 - val_loss: 0.0028
Epoch 3/50
147/147 ━━━━━━━━━━━━━━━━━━━━ 1s 10ms/step - loss: 0.0022 - val_loss: 0.0020
Epoch 4/50
147/147 ━━━━━━━━━━━━━━━━━━━━ 1s 10ms/step - loss: 0.0019 - val_loss: 0.0019
Epoch 5/50
147/147 ━━━━━━━━━━━━━━━━━━━━ 2s 10ms/step - loss: 0.0019 - val_loss: 0.0015
Epoch 6/50
147/147 ━━━━━━━━━━━━━━━━━━━━ 2s 10ms/step - loss: 0.0014 - val_loss: 0.0014
Epoch 7/50
147/147 ━━━━━━━━━━━━━━━━━━━━ 3s 11ms/step - loss: 0.0013 - val_loss: 0.0013
Epoch 8/50
147/147 ━━━━━━━━━━━━━━━━━━━━ 2s 14ms/step - loss: 0.0013 - val_loss: 0.0012
Epoch 9/50
147/147 ━━━━━━━━━━━━━━━━━━━━ 2s 10ms/step - loss: 0.0013 - val_loss: 0.0011
Epoch 10/50
147/147 ━━━━━━━━━━━━━━━━━━━━ 3s 10ms/step - loss: 0.0013 - val_loss: 0.0012
Epoch 11/50
147/147 ━━━━━━━━━━━━━━━━━━━━ 3s 10ms/step - loss: 0.0012 - val_loss: 0.0011
Epoch 12/50
147/147 ━━━━━━━━━━━━━━━━━━━━ 3s 10ms/step - loss: 0.0011 - val_loss: 0.0012
Epoch 13/50
147/147 ━━━━━━━━━━━━━━━━━━━━ 2s 13ms/step - loss: 0.0012 - val_loss: 0.0011
Epoch 14/50
147/147 ━━━━━━━━━━━━━━━━━━━━ 2s 11ms/step - loss: 0.0011 - val_loss: 0.0011
Epoch 15/50
147/147 ━━━━━━━━━━━━━━━━━━━━ 2s 11ms/step - loss: 0.0012 - val_loss: 0.0011
Epoch 16/50
147/147 ━━━━━━━━━━━━━━━━━━━━ 1s 10ms/step - loss: 0.0011 - val_loss: 9.6676e-04
Epoch 17/50
147/147 ━━━━━━━━━━━━━━━━━━━━ 3s 10ms/step - loss: 0.0011 - val_loss: 0.0011
Epoch 18/50
147/147 ━━━━━━━━━━━━━━━━━━━━ 3s 11ms/step - loss: 0.0010 - val_loss: 0.0010
Epoch 19/50
147/147 ━━━━━━━━━━━━━━━━━━━━ 3s 14ms/step - loss: 9.2326e-04 - val_loss: 0.0011
Epoch 20/50
147/147 ━━━━━━━━━━━━━━━━━━━━ 2s 11ms/step - loss: 9.7686e-04 - val_loss: 9.5169e-04
Epoch 21/50
147/147 ━━━━━━━━━━━━━━━━━━━━ 2s 11ms/step - loss: 9.6281e-04 - val_loss: 0.0012
Epoch 22/50
```

```
147/147 ━━━━━━━━━━━━━━━━━━━━ 2s 11ms/step - loss: 9.0885e-04 - val_loss: 9.2603e-04
Epoch 23/50
147/147 ━━━━━━━━━━━━━━━━━━━━ 2s 11ms/step - loss: 8.4369e-04 - val_loss: 8.4830e-04
Epoch 24/50
147/147 ━━━━━━━━━━━━━━━━━━━━ 1s 10ms/step - loss: 8.1348e-04 - val_loss: 8.4769e-04
Epoch 25/50
147/147 ━━━━━━━━━━━━━━━━━━━━ 2s 13ms/step - loss: 8.5026e-04 - val_loss: 9.5265e-04
Epoch 26/50
147/147 ━━━━━━━━━━━━━━━━━━━━ 2s 14ms/step - loss: 8.5755e-04 - val_loss: 8.8231e-04
Epoch 27/50
147/147 ━━━━━━━━━━━━━━━━━━━━ 2s 11ms/step - loss: 7.7781e-04 - val_loss: 8.9664e-04
Epoch 28/50
147/147 ━━━━━━━━━━━━━━━━━━━━ 2s 10ms/step - loss: 7.2269e-04 - val_loss: 8.0015e-04
Epoch 29/50
147/147 ━━━━━━━━━━━━━━━━━━━━ 3s 10ms/step - loss: 6.8096e-04 - val_loss: 7.3599e-04
Epoch 30/50
147/147 ━━━━━━━━━━━━━━━━━━━━ 3s 10ms/step - loss: 6.9115e-04 - val_loss: 9.6950e-04
Epoch 31/50
147/147 ━━━━━━━━━━━━━━━━━━━━ 3s 14ms/step - loss: 7.2969e-04 - val_loss: 7.1601e-04
Epoch 32/50
147/147 ━━━━━━━━━━━━━━━━━━━━ 2s 11ms/step - loss: 6.7008e-04 - val_loss: 8.6329e-04
Epoch 33/50
147/147 ━━━━━━━━━━━━━━━━━━━━ 2s 11ms/step - loss: 7.2020e-04 - val_loss: 7.3718e-04
Epoch 34/50
147/147 ━━━━━━━━━━━━━━━━━━━━ 2s 10ms/step - loss: 6.0350e-04 - val_loss: 7.4319e-04
Epoch 35/50
147/147 ━━━━━━━━━━━━━━━━━━━━ 3s 11ms/step - loss: 6.4117e-04 - val_loss: 7.2114e-04
Epoch 36/50
147/147 ━━━━━━━━━━━━━━━━━━━━ 2s 11ms/step - loss: 6.5047e-04 - val_loss: 6.8075e-04
Epoch 37/50
147/147 ━━━━━━━━━━━━━━━━━━━━ 2s 16ms/step - loss: 5.7883e-04 - val_loss: 7.3321e-04
Epoch 38/50
147/147 ━━━━━━━━━━━━━━━━━━━━ 2s 10ms/step - loss: 6.1252e-04 - val_loss: 6.6859e-04
Epoch 39/50
147/147 ━━━━━━━━━━━━━━━━━━━━ 3s 11ms/step - loss: 5.4135e-04 - val_loss: 7.2588e-04
Epoch 40/50
147/147 ━━━━━━━━━━━━━━━━━━━━ 2s 10ms/step - loss: 5.5360e-04 - val_loss: 6.6786e-04
Epoch 41/50
147/147 ━━━━━━━━━━━━━━━━━━━━ 1s 10ms/step - loss: 5.5784e-04 - val_loss: 7.3324e-04
Epoch 42/50
147/147 ━━━━━━━━━━━━━━━━━━━━ 2s 11ms/step - loss: 5.3780e-04 - val_loss: 6.6291e-04
Epoch 43/50
147/147 ━━━━━━━━━━━━━━━━━━━━ 2s 14ms/step - loss: 5.3856e-04 - val_loss: 7.1924e-04
Epoch 44/50
147/147 ━━━━━━━━━━━━━━━━━━━━ 2s 12ms/step - loss: 5.2327e-04 - val_loss: 6.5609e-04
Epoch 45/50
147/147 ━━━━━━━━━━━━━━━━━━━━ 2s 10ms/step - loss: 4.9588e-04 - val_loss: 6.1083e-04
Epoch 46/50
147/147 ━━━━━━━━━━━━━━━━━━━━ 3s 10ms/step - loss: 5.0996e-04 - val_loss: 6.2007e-04
```

```
Epoch 47/50
147/147 ━━━━━━━━━━━━━━━━━━━━ 2s 11ms/step - loss: 5.0529e-04 - val_loss: 6.4165e-04
Epoch 48/50
147/147 ━━━━━━━━━━━━━━━━━━━━ 1s 10ms/step - loss: 4.9883e-04 - val_loss: 6.0372e-04
Epoch 49/50
147/147 ━━━━━━━━━━━━━━━━━━━━ 3s 16ms/step - loss: 4.7165e-04 - val_loss: 5.9711e-04
Epoch 50/50
147/147 ━━━━━━━━━━━━━━━━━━━━ 2s 10ms/step - loss: 4.6111e-04 - val_loss: 5.9826e-04
```

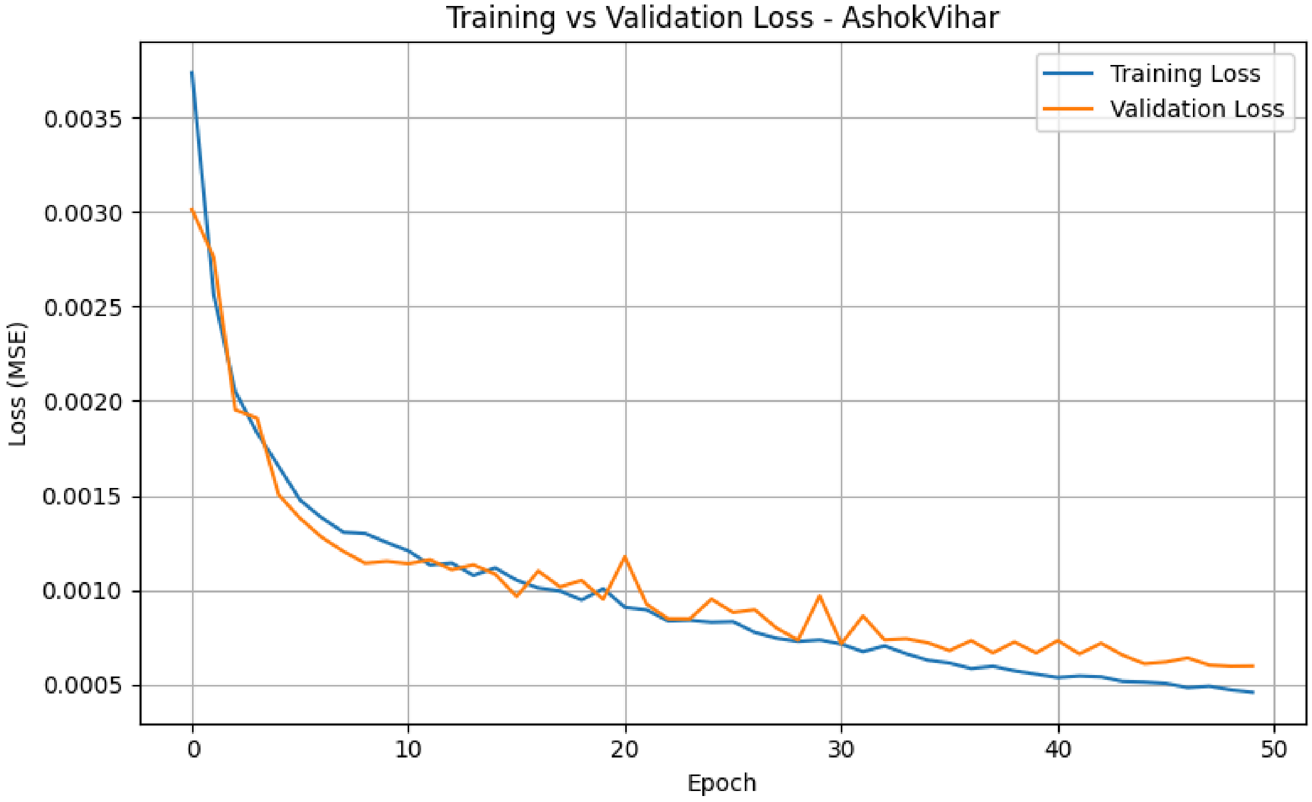


```
74/74 ━━━━━━━━━━━━━━━━━━━━ 1s 8ms/step
```

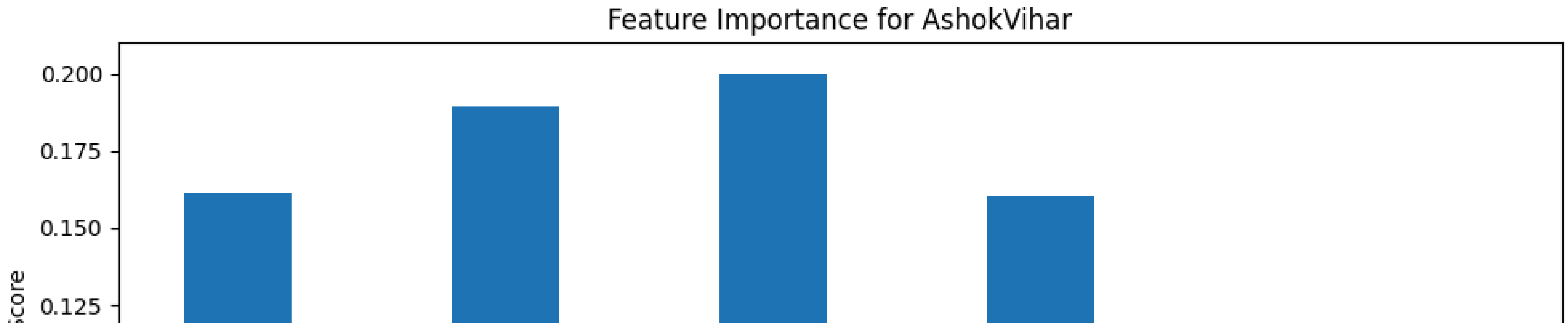

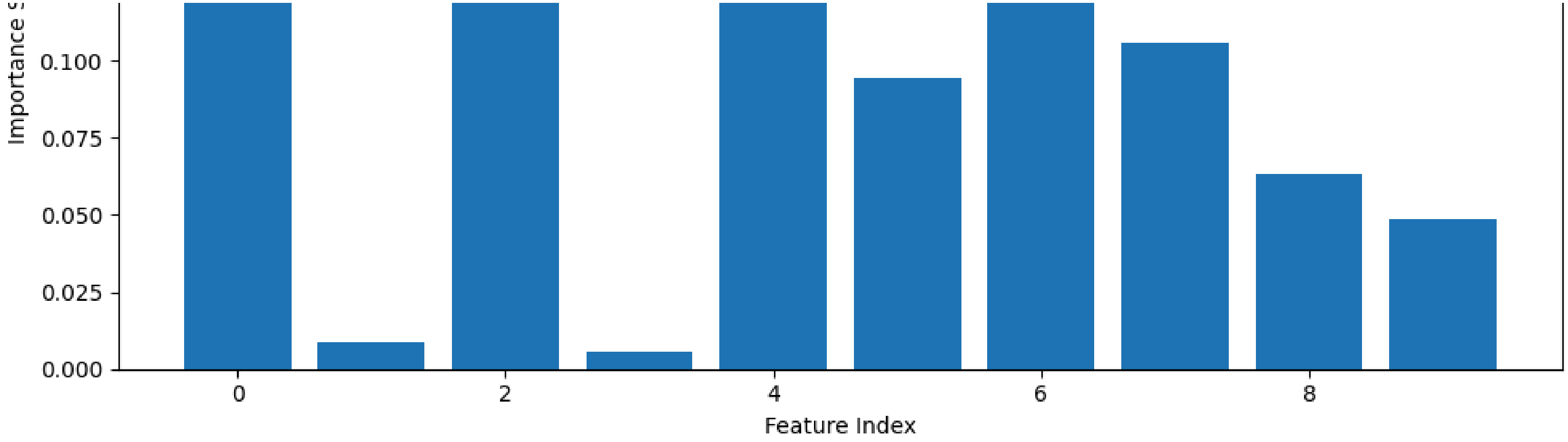

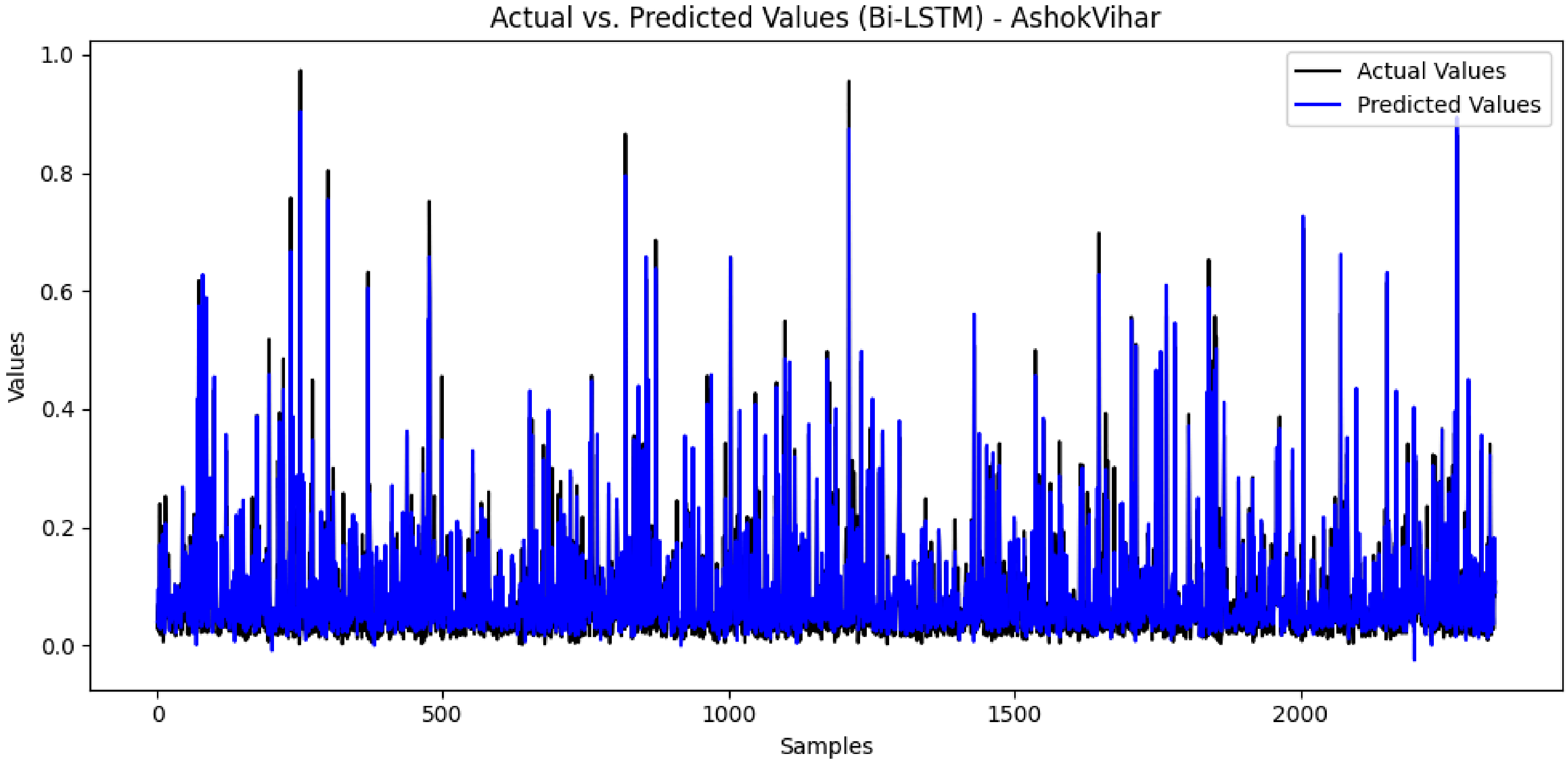


```
Processing Station: DCStadium
/usr/local/lib/python3.11/dist-packages/pywt/_multilevel.py:43: UserWarning: Level value of 3 is too high: all coefficients will experience boundary effe
  warnings.warn(
Epoch 1/50
```

```
Epoch 1/50
/usr/local/lib/python3.11/dist-packages/keras/src/layers/rnn/rnn.py:200: UserWarning: Do not pass an `input_shape`/`input_dim` argument to a layer. When
  super().__init__(**kwargs)
147/147 ━━━━━━━━━━━━━━━━━━━━ 6s 17ms/step - loss: 0.0244 - val_loss: 0.0098
Epoch 2/50
147/147 ━━━━━━━━━━━━━━━━━━━━ 2s 14ms/step - loss: 0.0081 - val_loss: 0.0034
Epoch 3/50
147/147 ━━━━━━━━━━━━━━━━━━━━ 2s 11ms/step - loss: 0.0032 - val_loss: 0.0022
Epoch 4/50
147/147 ━━━━━━━━━━━━━━━━━━━━ 2s 10ms/step - loss: 0.0023 - val_loss: 0.0020
Epoch 5/50
147/147 ━━━━━━━━━━━━━━━━━━━━ 2s 11ms/step - loss: 0.0021 - val_loss: 0.0019
Epoch 6/50
147/147 ━━━━━━━━━━━━━━━━━━━━ 3s 11ms/step - loss: 0.0021 - val_loss: 0.0018
Epoch 7/50
147/147 ━━━━━━━━━━━━━━━━━━━━ 2s 14ms/step - loss: 0.0021 - val_loss: 0.0017
Epoch 8/50
147/147 ━━━━━━━━━━━━━━━━━━━━ 2s 10ms/step - loss: 0.0018 - val_loss: 0.0020
Epoch 9/50
147/147 ━━━━━━━━━━━━━━━━━━━━ 2s 11ms/step - loss: 0.0019 - val_loss: 0.0018
Epoch 10/50
147/147 ━━━━━━━━━━━━━━━━━━━━ 3s 13ms/step - loss: 0.0017 - val_loss: 0.0016
Epoch 11/50
147/147 ━━━━━━━━━━━━━━━━━━━━ 2s 11ms/step - loss: 0.0018 - val_loss: 0.0016
Epoch 12/50
147/147 ━━━━━━━━━━━━━━━━━━━━ 3s 11ms/step - loss: 0.0016 - val_loss: 0.0017
Epoch 13/50
147/147 ━━━━━━━━━━━━━━━━━━━━ 2s 15ms/step - loss: 0.0016 - val_loss: 0.0015
Epoch 14/50
147/147 ━━━━━━━━━━━━━━━━━━━━ 2s 10ms/step - loss: 0.0014 - val_loss: 0.0016
Epoch 15/50
147/147 ━━━━━━━━━━━━━━━━━━━━ 2s 11ms/step - loss: 0.0014 - val_loss: 0.0014
Epoch 16/50
147/147 ━━━━━━━━━━━━━━━━━━━━ 2s 11ms/step - loss: 0.0016 - val_loss: 0.0015
Epoch 17/50
147/147 ━━━━━━━━━━━━━━━━━━━━ 2s 11ms/step - loss: 0.0014 - val_loss: 0.0013
Epoch 18/50
147/147 ━━━━━━━━━━━━━━━━━━━━ 2s 11ms/step - loss: 0.0014 - val_loss: 0.0012
Epoch 19/50
147/147 ━━━━━━━━━━━━━━━━━━━━ 2s 11ms/step - loss: 0.0013 - val_loss: 0.0013
Epoch 20/50
147/147 ━━━━━━━━━━━━━━━━━━━━ 2s 15ms/step - loss: 0.0012 - val_loss: 0.0014
Epoch 21/50
147/147 ━━━━━━━━━━━━━━━━━━━━ 2s 10ms/step - loss: 0.0012 - val_loss: 0.0015
Epoch 22/50
147/147 ━━━━━━━━━━━━━━━━━━━━ 3s 10ms/step - loss: 0.0012 - val_loss: 0.0011
Epoch 23/50
147/147 ━━━━━━━━━━━━━━━━━━━━ 2s 11ms/step - loss: 0.0012 - val_loss: 0.0011
Epoch 24/50
147/147 ━━━━━━━━━━━━━━━━━━━━ 2s 12ms/step - loss: 0.0011 - val_loss: 0.0012
```

```
Epoch 25/50
147/147 ━━━━━━━━━━━━━━━━━━━━ 3s 11ms/step - loss: 0.0011 - val_loss: 0.0012
Epoch 26/50
147/147 ━━━━━━━━━━━━━━━━━━━━ 2s 15ms/step - loss: 0.0011 - val_loss: 0.0012
Epoch 27/50
147/147 ━━━━━━━━━━━━━━━━━━━━ 2s 11ms/step - loss: 0.0012 - val_loss: 0.0011
Epoch 28/50
147/147 ━━━━━━━━━━━━━━━━━━━━ 2s 10ms/step - loss: 0.0010 - val_loss: 0.0010
Epoch 29/50
147/147 ━━━━━━━━━━━━━━━━━━━━ 3s 10ms/step - loss: 0.0011 - val_loss: 0.0010
Epoch 30/50
147/147 ━━━━━━━━━━━━━━━━━━━━ 2s 12ms/step - loss: 0.0011 - val_loss: 0.0011
Epoch 31/50
147/147 ━━━━━━━━━━━━━━━━━━━━ 2s 10ms/step - loss: 0.0010 - val_loss: 0.0012
Epoch 32/50
147/147 ━━━━━━━━━━━━━━━━━━━━ 3s 16ms/step - loss: 9.2894e-04 - val_loss: 0.0014
Epoch 33/50
147/147 ━━━━━━━━━━━━━━━━━━━━ 2s 11ms/step - loss: 0.0013 - val_loss: 0.0011
Epoch 34/50
147/147 ━━━━━━━━━━━━━━━━━━━━ 2s 12ms/step - loss: 0.0010 - val_loss: 0.0011
Epoch 35/50
147/147 ━━━━━━━━━━━━━━━━━━━━ 2s 10ms/step - loss: 9.6911e-04 - val_loss: 0.0012
Epoch 36/50
147/147 ━━━━━━━━━━━━━━━━━━━━ 3s 10ms/step - loss: 9.2048e-04 - val_loss: 0.0011
Epoch 37/50
147/147 ━━━━━━━━━━━━━━━━━━━━ 2s 11ms/step - loss: 9.1005e-04 - val_loss: 9.9777e-04
Epoch 38/50
147/147 ━━━━━━━━━━━━━━━━━━━━ 2s 13ms/step - loss: 9.4350e-04 - val_loss: 9.7009e-04
Epoch 39/50
147/147 ━━━━━━━━━━━━━━━━━━━━ 2s 15ms/step - loss: 8.9046e-04 - val_loss: 9.7887e-04
Epoch 40/50
147/147 ━━━━━━━━━━━━━━━━━━━━ 2s 11ms/step - loss: 8.1948e-04 - val_loss: 9.9988e-04
Epoch 41/50
147/147 ━━━━━━━━━━━━━━━━━━━━ 3s 10ms/step - loss: 9.6002e-04 - val_loss: 9.5025e-04
Epoch 42/50
147/147 ━━━━━━━━━━━━━━━━━━━━ 2s 11ms/step - loss: 8.6865e-04 - val_loss: 9.2921e-04
Epoch 43/50
147/147 ━━━━━━━━━━━━━━━━━━━━ 2s 11ms/step - loss: 9.3275e-04 - val_loss: 9.5002e-04
Epoch 44/50
147/147 ━━━━━━━━━━━━━━━━━━━━ 3s 13ms/step - loss: 8.8165e-04 - val_loss: 9.9960e-04
Epoch 45/50
147/147 ━━━━━━━━━━━━━━━━━━━━ 2s 14ms/step - loss: 8.9162e-04 - val_loss: 9.7761e-04
Epoch 46/50
147/147 ━━━━━━━━━━━━━━━━━━━━ 2s 11ms/step - loss: 8.5669e-04 - val_loss: 9.8559e-04
Epoch 47/50
147/147 ━━━━━━━━━━━━━━━━━━━━ 2s 11ms/step - loss: 8.5904e-04 - val_loss: 8.5569e-04
Epoch 48/50
147/147 ━━━━━━━━━━━━━━━━━━━━ 3s 11ms/step - loss: 8.3409e-04 - val_loss: 8.8657e-04
Epoch 49/50
```

```
147/147 ━━━━━━━━━━━━━━━━━━━━ 2s 10ms/step - loss: 7.2934e-04 - val_loss: 9.1872e-04
Epoch 50/50
147/147 ━━━━━━━━━━━━━━━━━━━━ 2s 13ms/step - loss: 7.5739e-04 - val_loss: 9.4018e-04
```

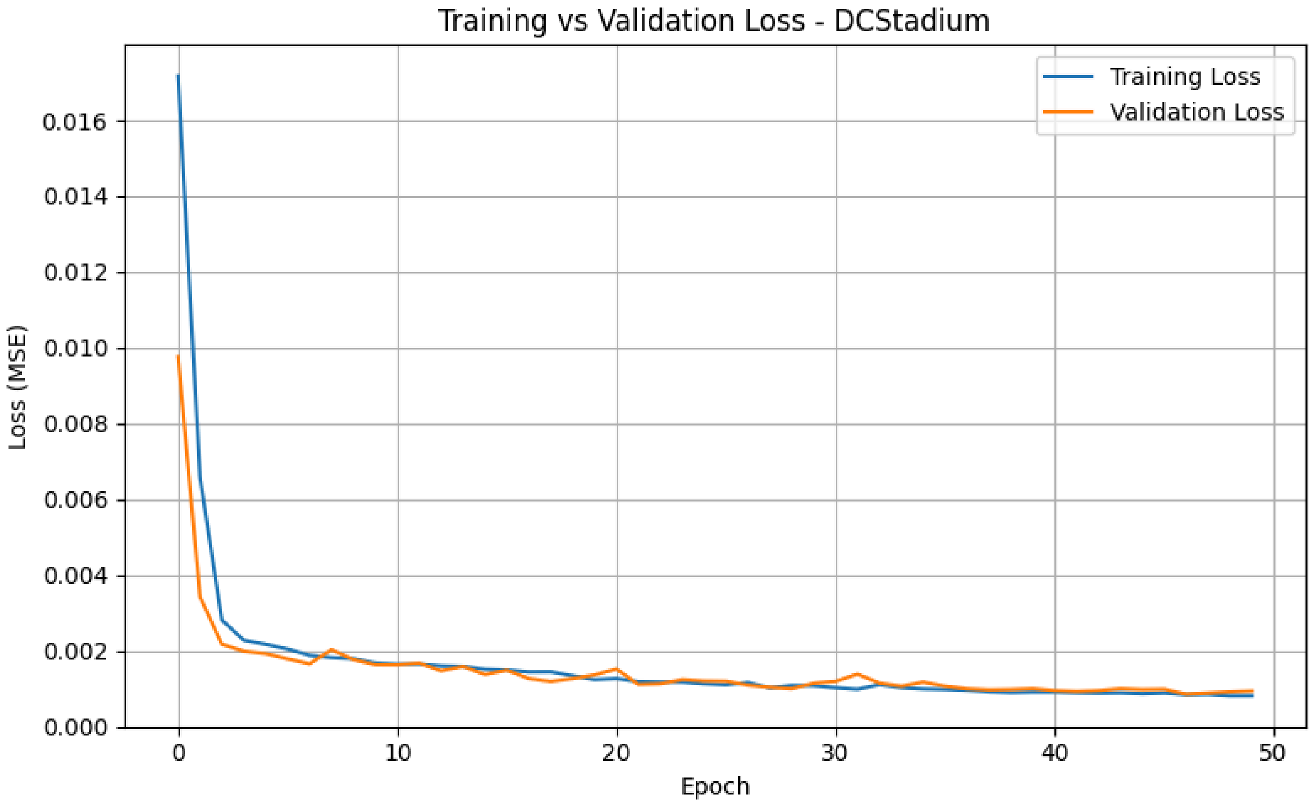


```
74/74 ━━━━━━━━━━━━━━━━━━━━ 1s 11ms/step
```

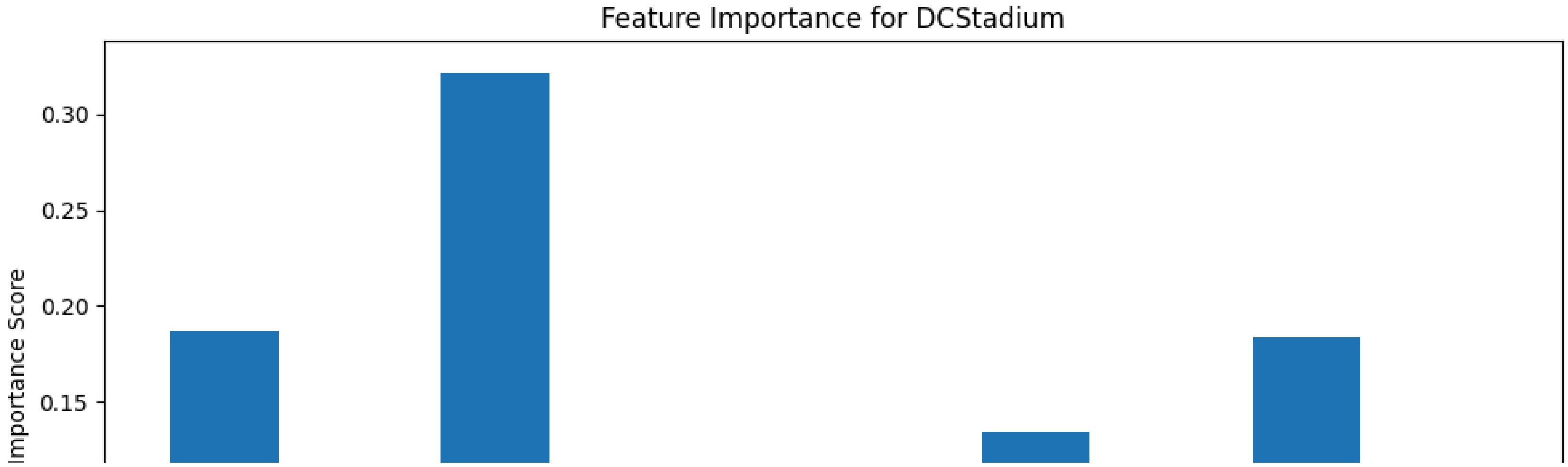

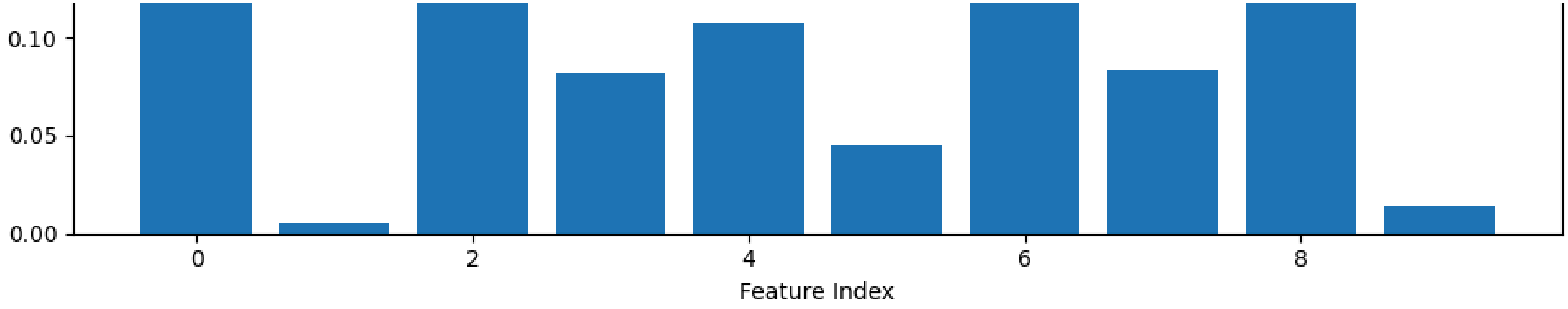

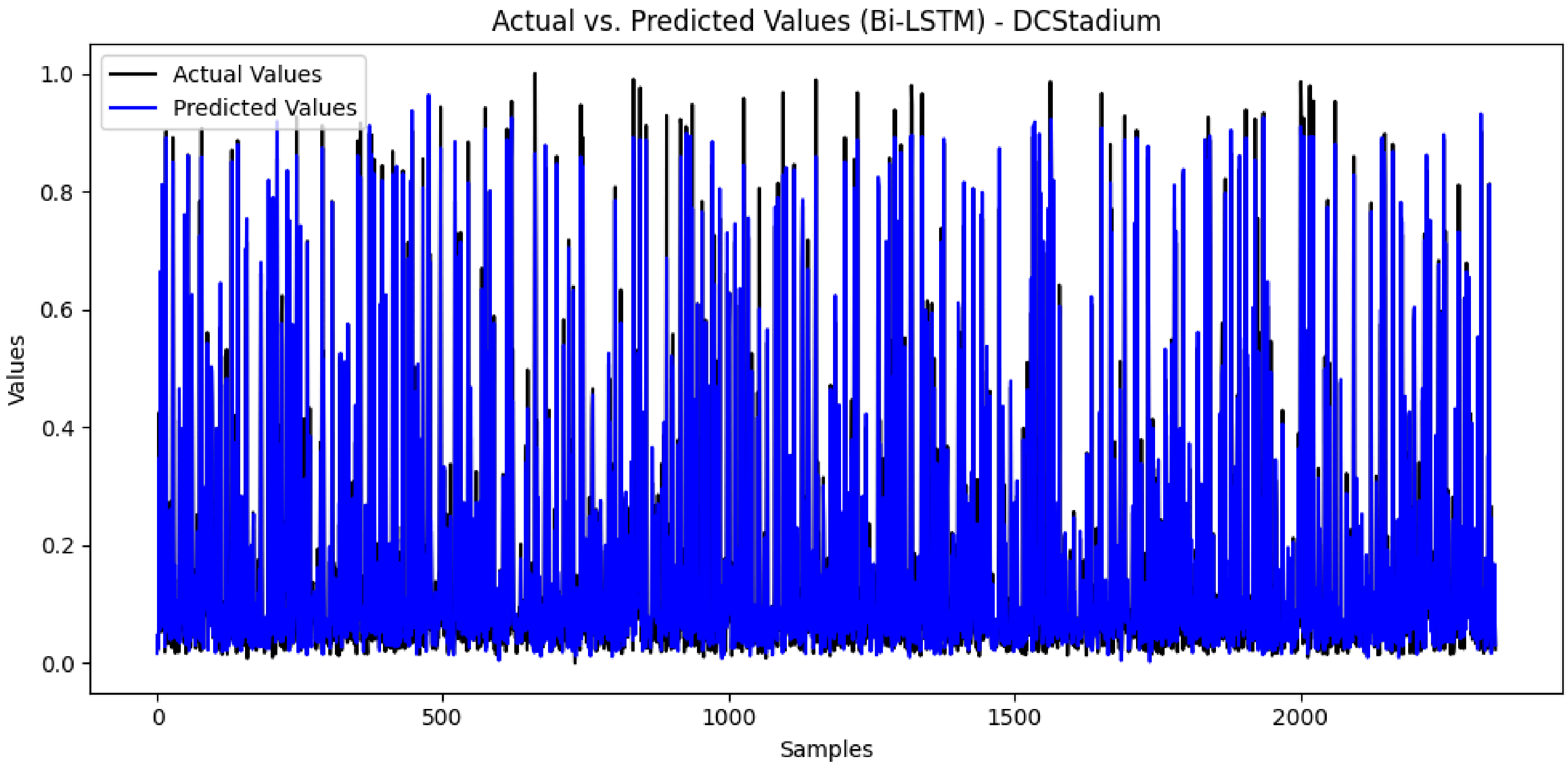


```
Processing Station: DwarkaSec8
/usr/local/lib/python3.11/dist-packages/pywt/_multilevel.py:43: UserWarning: Level value of 3 is too high: all coefficients will experience boundary effe
  warnings.warn(
Epoch 1/50
/usr/local/lib/python3.11/dist-packages/keras/src/layers/rnn/rnn.py:200: UserWarning: Do not pass an `input_shape`/`input_dim` argument to a layer. When
  super().__init__(**kwargs)
147/147 ━━━━━━━━━━━━━━━━━━━━ 50s 15ms/step - loss: 0.0072 - val_loss: 0.0035
Epoch 2/50
147/147 ━━━━━━━━━━━━━━━━━━━━ 2s 12ms/step - loss: 0.0030 - val_loss: 0.0021
```

```
Epoch 3/50
147/147 ━━━━━━━━━━━━━━━━━━━━ 2s 11ms/step - loss: 0.0020 - val_loss: 0.0017
Epoch 4/50
147/147 ━━━━━━━━━━━━━━━━━━━━ 2s 15ms/step - loss: 0.0017 - val_loss: 0.0016
Epoch 5/50
147/147 ━━━━━━━━━━━━━━━━━━━━ 2s 12ms/step - loss: 0.0015 - val_loss: 0.0014
Epoch 6/50
147/147 ━━━━━━━━━━━━━━━━━━━━ 2s 11ms/step - loss: 0.0014 - val_loss: 0.0014
Epoch 7/50
147/147 ━━━━━━━━━━━━━━━━━━━━ 2s 11ms/step - loss: 0.0013 - val_loss: 0.0013
Epoch 8/50
147/147 ━━━━━━━━━━━━━━━━━━━━ 2s 11ms/step - loss: 0.0013 - val_loss: 0.0013
Epoch 9/50
147/147 ━━━━━━━━━━━━━━━━━━━━ 3s 11ms/step - loss: 0.0012 - val_loss: 0.0012
Epoch 10/50
147/147 ━━━━━━━━━━━━━━━━━━━━ 2s 15ms/step - loss: 0.0013 - val_loss: 0.0012
Epoch 11/50
147/147 ━━━━━━━━━━━━━━━━━━━━ 2s 14ms/step - loss: 0.0011 - val_loss: 0.0012
Epoch 12/50
147/147 ━━━━━━━━━━━━━━━━━━━━ 2s 11ms/step - loss: 0.0011 - val_loss: 0.0012
Epoch 13/50
147/147 ━━━━━━━━━━━━━━━━━━━━ 2s 11ms/step - loss: 0.0011 - val_loss: 0.0012
Epoch 14/50
147/147 ━━━━━━━━━━━━━━━━━━━━ 2s 11ms/step - loss: 0.0010 - val_loss: 0.0012
Epoch 15/50
147/147 ━━━━━━━━━━━━━━━━━━━━ 3s 11ms/step - loss: 0.0010 - val_loss: 0.0011
Epoch 16/50
147/147 ━━━━━━━━━━━━━━━━━━━━ 3s 16ms/step - loss: 9.7279e-04 - val_loss: 0.0011
Epoch 17/50
147/147 ━━━━━━━━━━━━━━━━━━━━ 2s 12ms/step - loss: 9.5683e-04 - val_loss: 0.0011
Epoch 18/50
147/147 ━━━━━━━━━━━━━━━━━━━━ 2s 12ms/step - loss: 0.0010 - val_loss: 0.0010
Epoch 19/50
147/147 ━━━━━━━━━━━━━━━━━━━━ 2s 11ms/step - loss: 9.5053e-04 - val_loss: 0.0010
Epoch 20/50
147/147 ━━━━━━━━━━━━━━━━━━━━ 3s 11ms/step - loss: 9.5828e-04 - val_loss: 9.6449e-04
Epoch 21/50
147/147 ━━━━━━━━━━━━━━━━━━━━ 2s 12ms/step - loss: 8.6532e-04 - val_loss: 9.8803e-04
Epoch 22/50
147/147 ━━━━━━━━━━━━━━━━━━━━ 3s 16ms/step - loss: 8.9810e-04 - val_loss: 0.0010
Epoch 23/50
147/147 ━━━━━━━━━━━━━━━━━━━━ 2s 11ms/step - loss: 8.7878e-04 - val_loss: 9.2782e-04
Epoch 24/50
147/147 ━━━━━━━━━━━━━━━━━━━━ 2s 12ms/step - loss: 8.7755e-04 - val_loss: 9.5793e-04
Epoch 25/50
147/147 ━━━━━━━━━━━━━━━━━━━━ 2s 11ms/step - loss: 8.4419e-04 - val_loss: 9.3225e-04
Epoch 26/50
147/147 ━━━━━━━━━━━━━━━━━━━━ 2s 12ms/step - loss: 8.3253e-04 - val_loss: 9.5592e-04
Epoch 27/50
```

```
147/147 ━━━━━━━━━━━━━━━━━━━━ 2s 12ms/step - loss: 8.4219e-04 - val_loss: 9.7888e-04
Epoch 28/50
147/147 ━━━━━━━━━━━━━━━━━━━━ 3s 14ms/step - loss: 8.5147e-04 - val_loss: 8.9028e-04
Epoch 29/50
147/147 ━━━━━━━━━━━━━━━━━━━━ 2s 13ms/step - loss: 7.7611e-04 - val_loss: 8.7397e-04
Epoch 30/50
147/147 ━━━━━━━━━━━━━━━━━━━━ 2s 12ms/step - loss: 7.6072e-04 - val_loss: 9.0936e-04
Epoch 31/50
147/147 ━━━━━━━━━━━━━━━━━━━━ 2s 11ms/step - loss: 7.3752e-04 - val_loss: 0.0011
Epoch 32/50
147/147 ━━━━━━━━━━━━━━━━━━━━ 3s 11ms/step - loss: 7.7839e-04 - val_loss: 8.9162e-04
Epoch 33/50
147/147 ━━━━━━━━━━━━━━━━━━━━ 3s 13ms/step - loss: 7.3872e-04 - val_loss: 9.0162e-04
Epoch 34/50
147/147 ━━━━━━━━━━━━━━━━━━━━ 2s 14ms/step - loss: 7.8912e-04 - val_loss: 9.2239e-04
Epoch 35/50
147/147 ━━━━━━━━━━━━━━━━━━━━ 2s 11ms/step - loss: 7.3229e-04 - val_loss: 8.3609e-04
Epoch 36/50
147/147 ━━━━━━━━━━━━━━━━━━━━ 3s 11ms/step - loss: 7.4205e-04 - val_loss: 8.5541e-04
Epoch 37/50
147/147 ━━━━━━━━━━━━━━━━━━━━ 2s 11ms/step - loss: 7.4985e-04 - val_loss: 8.0890e-04
Epoch 38/50
147/147 ━━━━━━━━━━━━━━━━━━━━ 3s 11ms/step - loss: 7.0271e-04 - val_loss: 8.2867e-04
Epoch 39/50
147/147 ━━━━━━━━━━━━━━━━━━━━ 3s 16ms/step - loss: 6.6579e-04 - val_loss: 8.1682e-04
Epoch 40/50
147/147 ━━━━━━━━━━━━━━━━━━━━ 2s 12ms/step - loss: 6.9822e-04 - val_loss: 7.7847e-04
Epoch 41/50
147/147 ━━━━━━━━━━━━━━━━━━━━ 2s 12ms/step - loss: 6.6052e-04 - val_loss: 8.3858e-04
Epoch 42/50
147/147 ━━━━━━━━━━━━━━━━━━━━ 2s 12ms/step - loss: 6.6972e-04 - val_loss: 8.4767e-04
Epoch 43/50
147/147 ━━━━━━━━━━━━━━━━━━━━ 3s 12ms/step - loss: 6.6927e-04 - val_loss: 7.8044e-04
Epoch 44/50
147/147 ━━━━━━━━━━━━━━━━━━━━ 2s 11ms/step - loss: 5.9373e-04 - val_loss: 7.9786e-04
Epoch 45/50
147/147 ━━━━━━━━━━━━━━━━━━━━ 3s 15ms/step - loss: 5.8679e-04 - val_loss: 7.8414e-04
Epoch 46/50
147/147 ━━━━━━━━━━━━━━━━━━━━ 2s 12ms/step - loss: 6.0666e-04 - val_loss: 7.8269e-04
Epoch 47/50
147/147 ━━━━━━━━━━━━━━━━━━━━ 2s 11ms/step - loss: 5.8250e-04 - val_loss: 8.0693e-04
Epoch 48/50
147/147 ━━━━━━━━━━━━━━━━━━━━ 3s 11ms/step - loss: 6.1275e-04 - val_loss: 7.5063e-04
Epoch 49/50
147/147 ━━━━━━━━━━━━━━━━━━━━ 2s 11ms/step - loss: 6.0530e-04 - val_loss: 8.4836e-04
Epoch 50/50
147/147 ━━━━━━━━━━━━━━━━━━━━ 2s 12ms/step - loss: 6.0393e-04 - val_loss: 7.6264e-04
```

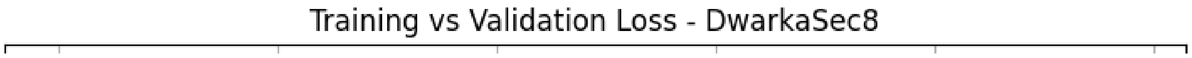

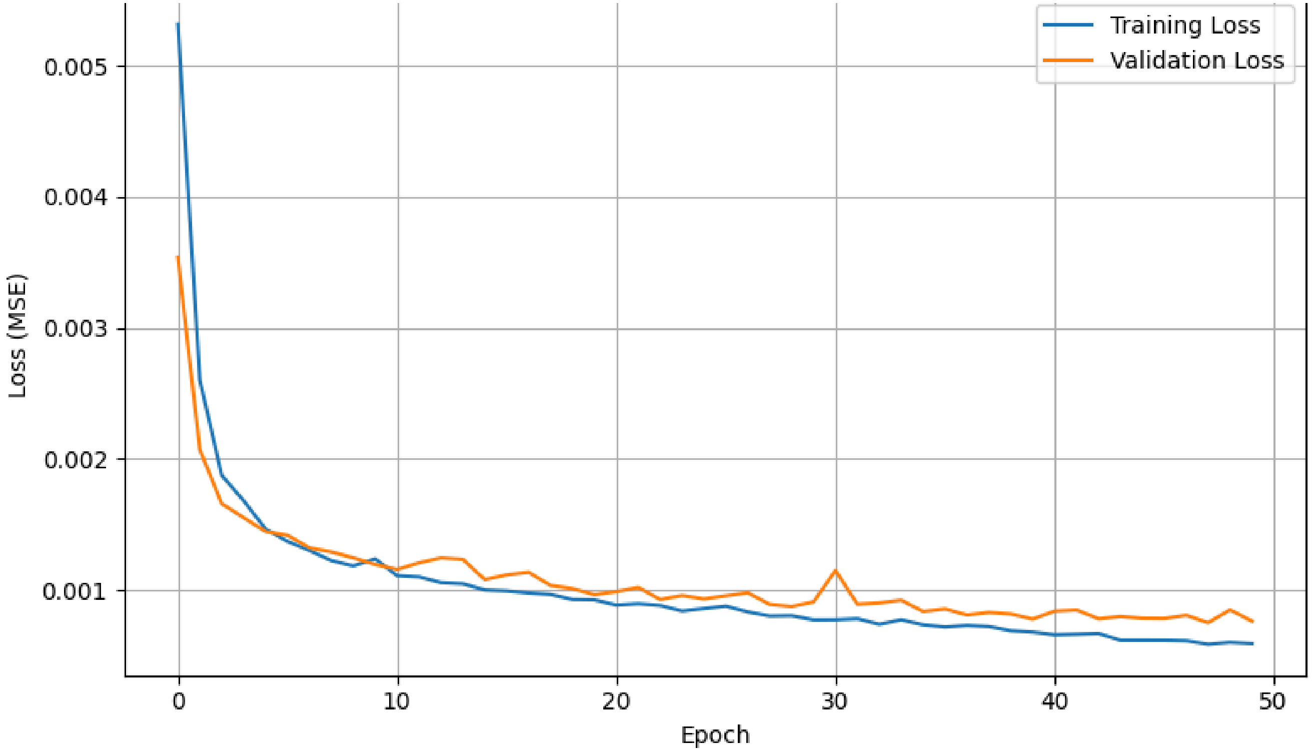


```
74/74 ━━━━━━━━━━━━━━━━━━━━ 1s 8ms/step
```

Feature Importance for DwarkaSec8

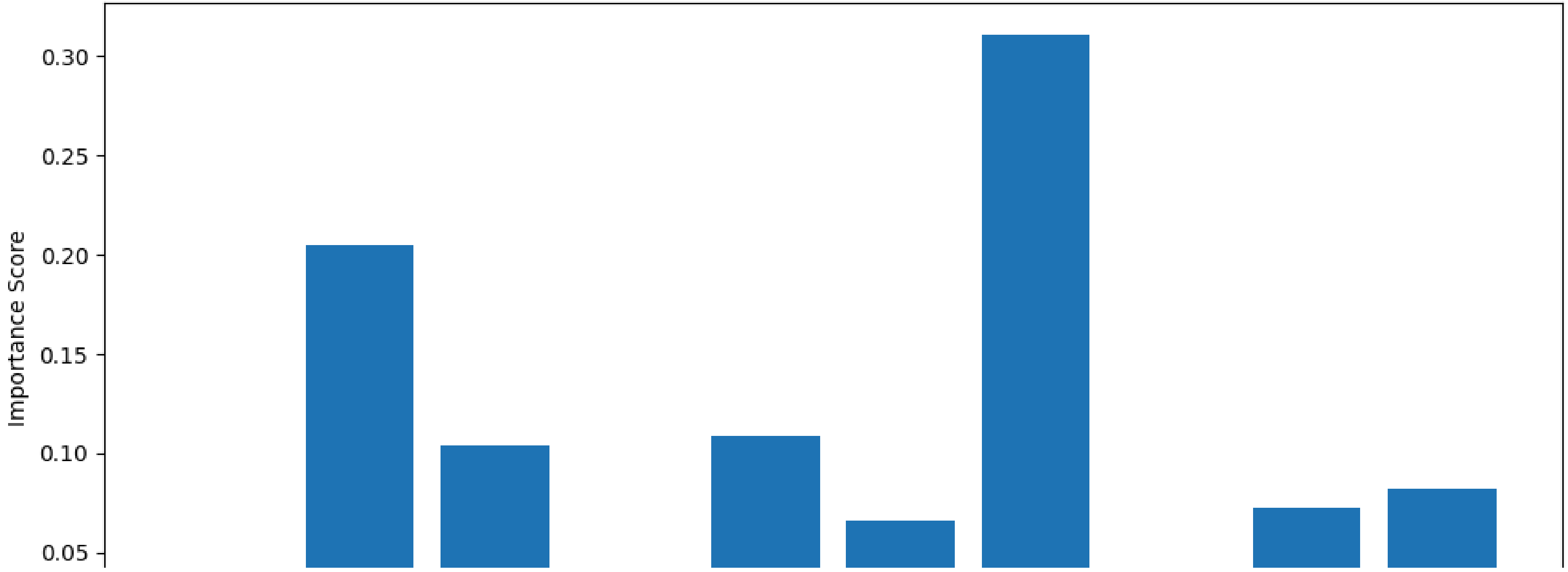

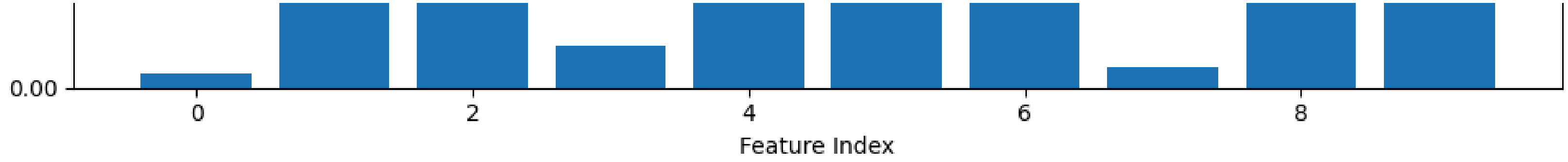

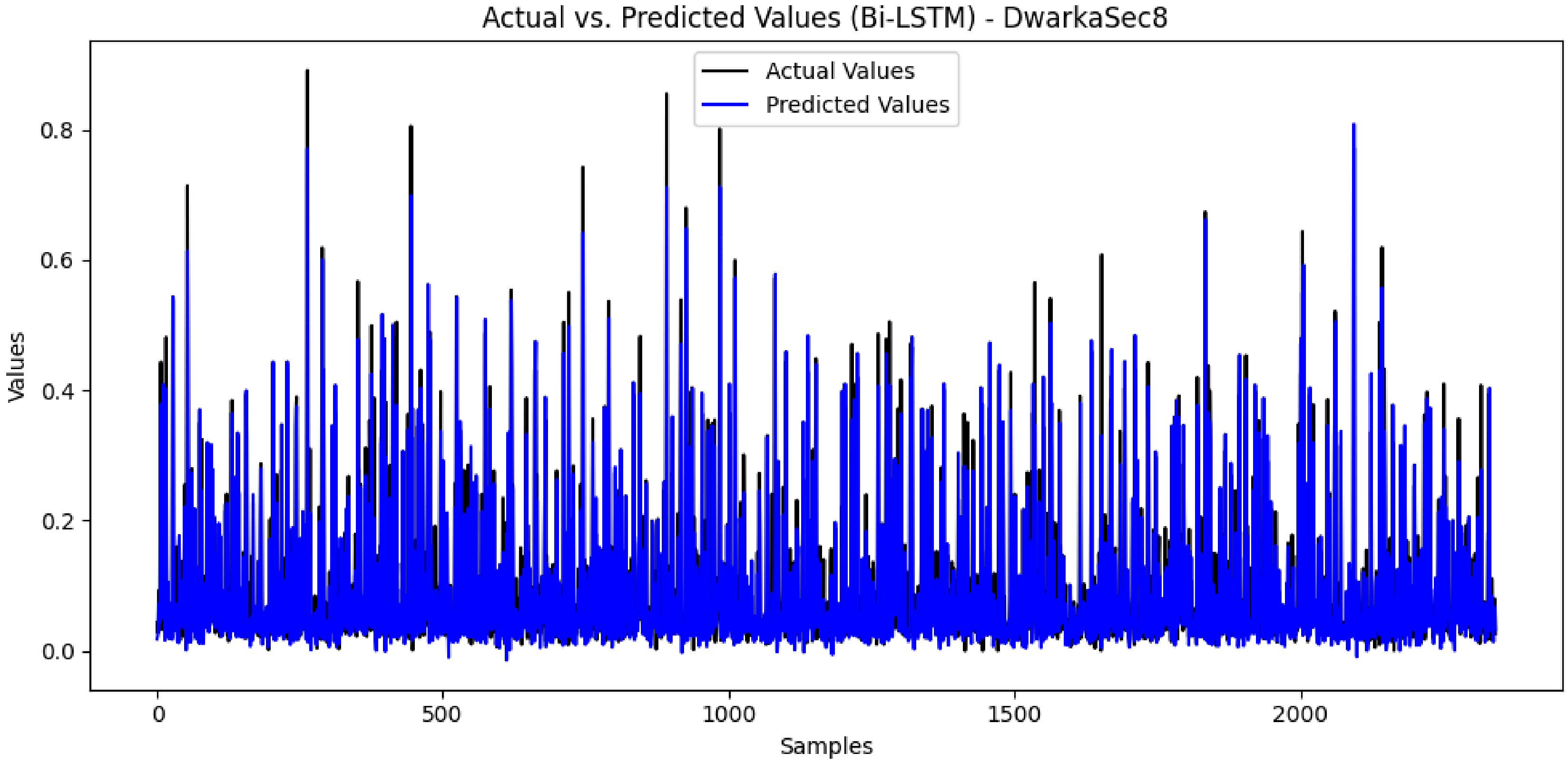


```
Processing Station: NehruNagar
/usr/local/lib/python3.11/dist-packages/pywt/_multilevel.py:43: UserWarning: Level value of 3 is too high: all coefficients will experience boundary effe
  warnings.warn(
Epoch 1/50
/usr/local/lib/python3.11/dist-packages/keras/src/layers/rnn/rnn.py:200: UserWarning: Do not pass an `input_shape`/`input_dim` argument to a layer. When
  super().__init__(**kwargs)
147/147 ━━━━━━━━━━━━━━━━━━━━ 6s 14ms/step - loss: 0.0161 - val_loss: 0.0047
Epoch 2/50
147/147 ━━━━━━━━━━━━━━━━━━━━ 2s 11ms/step - loss: 0.0041 - val_loss: 0.0033
Epoch 3/50
147/147 ━━━━━━━━━━━━━━━━━━━━ 2s 15ms/step - loss: 0.0024 - val_loss: 0.0021
Epoch 4/50
147/147 ━━━━━━━━━━━━━━━━━━━━ 2s 14ms/step - loss: 0.0019 - val_loss: 0.0020
Epoch 5/50
```

```
147/147 ━━━━━━━━━━━━━━━━━━━━ 2s 12ms/step - loss: 0.0019 - val_loss: 0.0020
Epoch 6/50
147/147 ━━━━━━━━━━━━━━━━━━━━ 2s 11ms/step - loss: 0.0017 - val_loss: 0.0019
Epoch 7/50
147/147 ━━━━━━━━━━━━━━━━━━━━ 2s 12ms/step - loss: 0.0018 - val_loss: 0.0017
Epoch 8/50
147/147 ━━━━━━━━━━━━━━━━━━━━ 2s 11ms/step - loss: 0.0016 - val_loss: 0.0017
Epoch 9/50
147/147 ━━━━━━━━━━━━━━━━━━━━ 2s 12ms/step - loss: 0.0015 - val_loss: 0.0018
Epoch 10/50
147/147 ━━━━━━━━━━━━━━━━━━━━ 2s 15ms/step - loss: 0.0015 - val_loss: 0.0015
Epoch 11/50
147/147 ━━━━━━━━━━━━━━━━━━━━ 2s 13ms/step - loss: 0.0013 - val_loss: 0.0016
Epoch 12/50
147/147 ━━━━━━━━━━━━━━━━━━━━ 2s 12ms/step - loss: 0.0014 - val_loss: 0.0017
Epoch 13/50
147/147 ━━━━━━━━━━━━━━━━━━━━ 2s 11ms/step - loss: 0.0013 - val_loss: 0.0015
Epoch 14/50
147/147 ━━━━━━━━━━━━━━━━━━━━ 2s 11ms/step - loss: 0.0013 - val_loss: 0.0015
Epoch 15/50
147/147 ━━━━━━━━━━━━━━━━━━━━ 3s 12ms/step - loss: 0.0012 - val_loss: 0.0013
Epoch 16/50
147/147 ━━━━━━━━━━━━━━━━━━━━ 2s 16ms/step - loss: 0.0012 - val_loss: 0.0013
Epoch 17/50
147/147 ━━━━━━━━━━━━━━━━━━━━ 2s 11ms/step - loss: 0.0011 - val_loss: 0.0012
Epoch 18/50
147/147 ━━━━━━━━━━━━━━━━━━━━ 2s 11ms/step - loss: 0.0011 - val_loss: 0.0012
Epoch 19/50
147/147 ━━━━━━━━━━━━━━━━━━━━ 3s 12ms/step - loss: 9.6715e-04 - val_loss: 0.0011
Epoch 20/50
147/147 ━━━━━━━━━━━━━━━━━━━━ 2s 11ms/step - loss: 9.9984e-04 - val_loss: 0.0011
Epoch 21/50
147/147 ━━━━━━━━━━━━━━━━━━━━ 2s 11ms/step - loss: 9.6671e-04 - val_loss: 0.0010
Epoch 22/50
147/147 ━━━━━━━━━━━━━━━━━━━━ 2s 13ms/step - loss: 9.0545e-04 - val_loss: 9.4980e-04
Epoch 23/50
147/147 ━━━━━━━━━━━━━━━━━━━━ 2s 15ms/step - loss: 8.8365e-04 - val_loss: 9.5376e-04
Epoch 24/50
147/147 ━━━━━━━━━━━━━━━━━━━━ 2s 12ms/step - loss: 8.3489e-04 - val_loss: 9.2824e-04
Epoch 25/50
147/147 ━━━━━━━━━━━━━━━━━━━━ 2s 11ms/step - loss: 8.0141e-04 - val_loss: 8.7930e-04
Epoch 26/50
147/147 ━━━━━━━━━━━━━━━━━━━━ 2s 11ms/step - loss: 8.5277e-04 - val_loss: 8.4463e-04
Epoch 27/50
147/147 ━━━━━━━━━━━━━━━━━━━━ 2s 12ms/step - loss: 7.8384e-04 - val_loss: 8.5775e-04
Epoch 28/50
147/147 ━━━━━━━━━━━━━━━━━━━━ 3s 14ms/step - loss: 7.6247e-04 - val_loss: 8.1393e-04
Epoch 29/50
147/147 ━━━━━━━━━━━━━━━━━━━━ 3s 14ms/step - loss: 7.0732e-04 - val_loss: 7.7329e-04
```

```
Epoch 30/50
147/147 ━━━━━━━━━━━━━━━━━━━━ 2s 12ms/step - loss: 6.9008e-04 - val_loss: 7.5997e-04
Epoch 31/50
147/147 ━━━━━━━━━━━━━━━━━━━━ 2s 12ms/step - loss: 6.4699e-04 - val_loss: 7.5958e-04
Epoch 32/50
147/147 ━━━━━━━━━━━━━━━━━━━━ 2s 11ms/step - loss: 6.8405e-04 - val_loss: 8.0197e-04
Epoch 33/50
147/147 ━━━━━━━━━━━━━━━━━━━━ 2s 11ms/step - loss: 6.6939e-04 - val_loss: 7.7563e-04
Epoch 34/50
147/147 ━━━━━━━━━━━━━━━━━━━━ 3s 14ms/step - loss: 6.8950e-04 - val_loss: 7.6788e-04
Epoch 35/50
147/147 ━━━━━━━━━━━━━━━━━━━━ 2s 15ms/step - loss: 6.1357e-04 - val_loss: 6.9068e-04
Epoch 36/50
147/147 ━━━━━━━━━━━━━━━━━━━━ 2s 11ms/step - loss: 6.0377e-04 - val_loss: 7.0679e-04
Epoch 37/50
147/147 ━━━━━━━━━━━━━━━━━━━━ 2s 11ms/step - loss: 6.0247e-04 - val_loss: 7.2992e-04
Epoch 38/50
147/147 ━━━━━━━━━━━━━━━━━━━━ 2s 11ms/step - loss: 6.0419e-04 - val_loss: 6.6893e-04
Epoch 39/50
147/147 ━━━━━━━━━━━━━━━━━━━━ 3s 11ms/step - loss: 5.3962e-04 - val_loss: 6.5593e-04
Epoch 40/50
147/147 ━━━━━━━━━━━━━━━━━━━━ 2s 13ms/step - loss: 5.3921e-04 - val_loss: 6.8192e-04
Epoch 41/50
147/147 ━━━━━━━━━━━━━━━━━━━━ 2s 15ms/step - loss: 5.5038e-04 - val_loss: 6.8856e-04
Epoch 42/50
147/147 ━━━━━━━━━━━━━━━━━━━━ 2s 12ms/step - loss: 5.0990e-04 - val_loss: 6.9136e-04
Epoch 43/50
147/147 ━━━━━━━━━━━━━━━━━━━━ 2s 11ms/step - loss: 5.5359e-04 - val_loss: 6.1752e-04
Epoch 44/50
147/147 ━━━━━━━━━━━━━━━━━━━━ 3s 11ms/step - loss: 5.2003e-04 - val_loss: 6.5469e-04
Epoch 45/50
147/147 ━━━━━━━━━━━━━━━━━━━━ 3s 12ms/step - loss: 4.7334e-04 - val_loss: 6.6416e-04
Epoch 46/50
147/147 ━━━━━━━━━━━━━━━━━━━━ 2s 13ms/step - loss: 4.7354e-04 - val_loss: 6.3735e-04
Epoch 47/50
147/147 ━━━━━━━━━━━━━━━━━━━━ 2s 15ms/step - loss: 5.8005e-04 - val_loss: 5.5645e-04
Epoch 48/50
147/147 ━━━━━━━━━━━━━━━━━━━━ 2s 11ms/step - loss: 4.2763e-04 - val_loss: 5.9908e-04
Epoch 49/50
147/147 ━━━━━━━━━━━━━━━━━━━━ 2s 11ms/step - loss: 4.5083e-04 - val_loss: 5.2794e-04
Epoch 50/50
147/147 ━━━━━━━━━━━━━━━━━━━━ 3s 11ms/step - loss: 4.5491e-04 - val_loss: 5.9846e-04
```

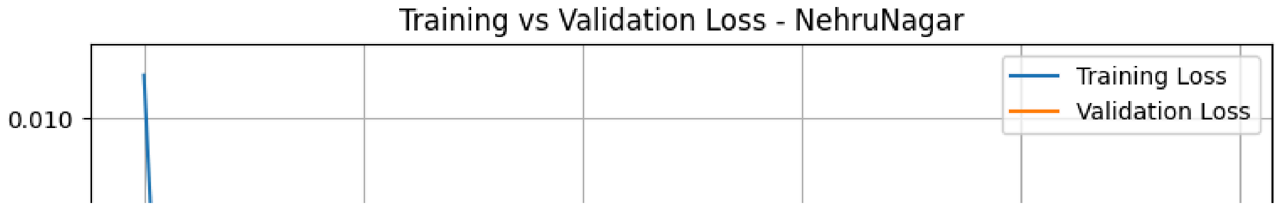

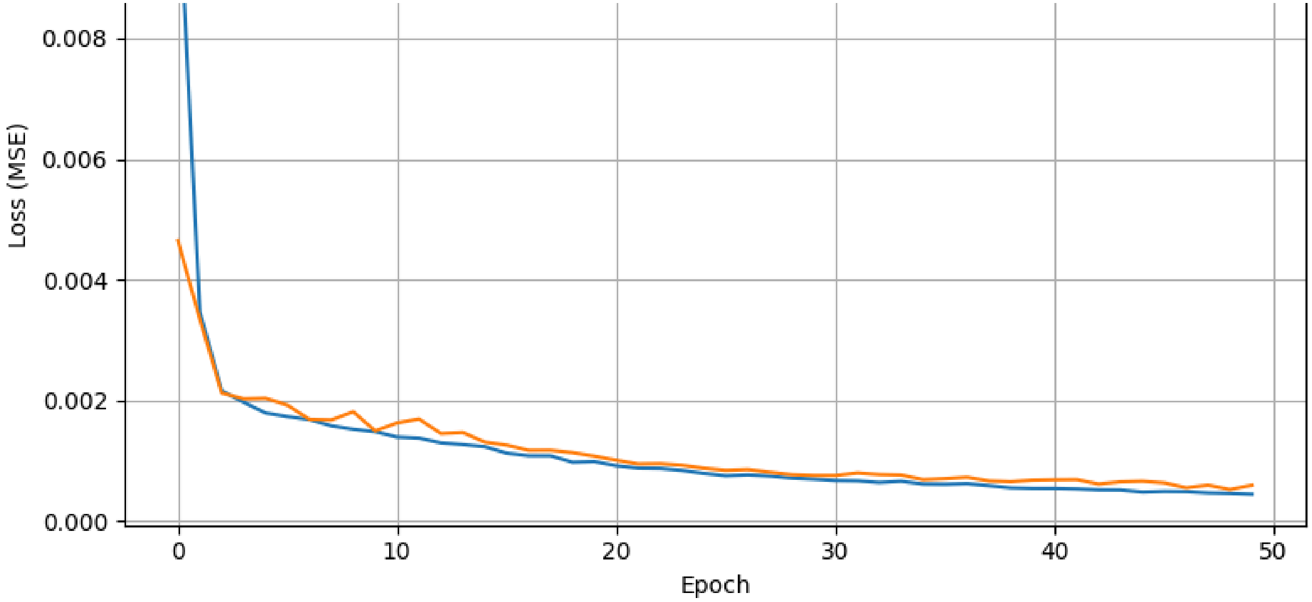


```
74/74 ━━━━━━━━━━━━━━━━━━━━ 1s 9ms/step
```

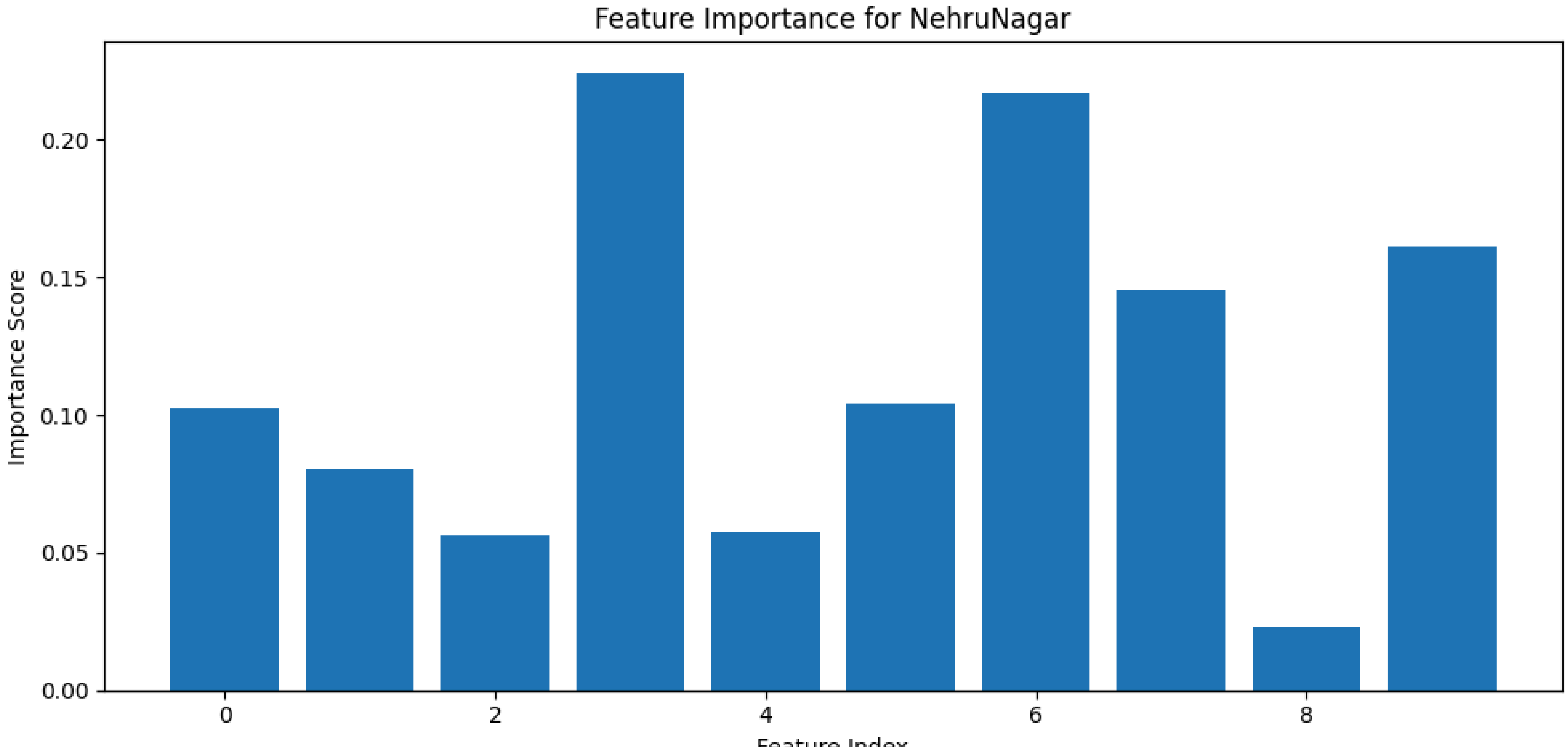

Feature Index

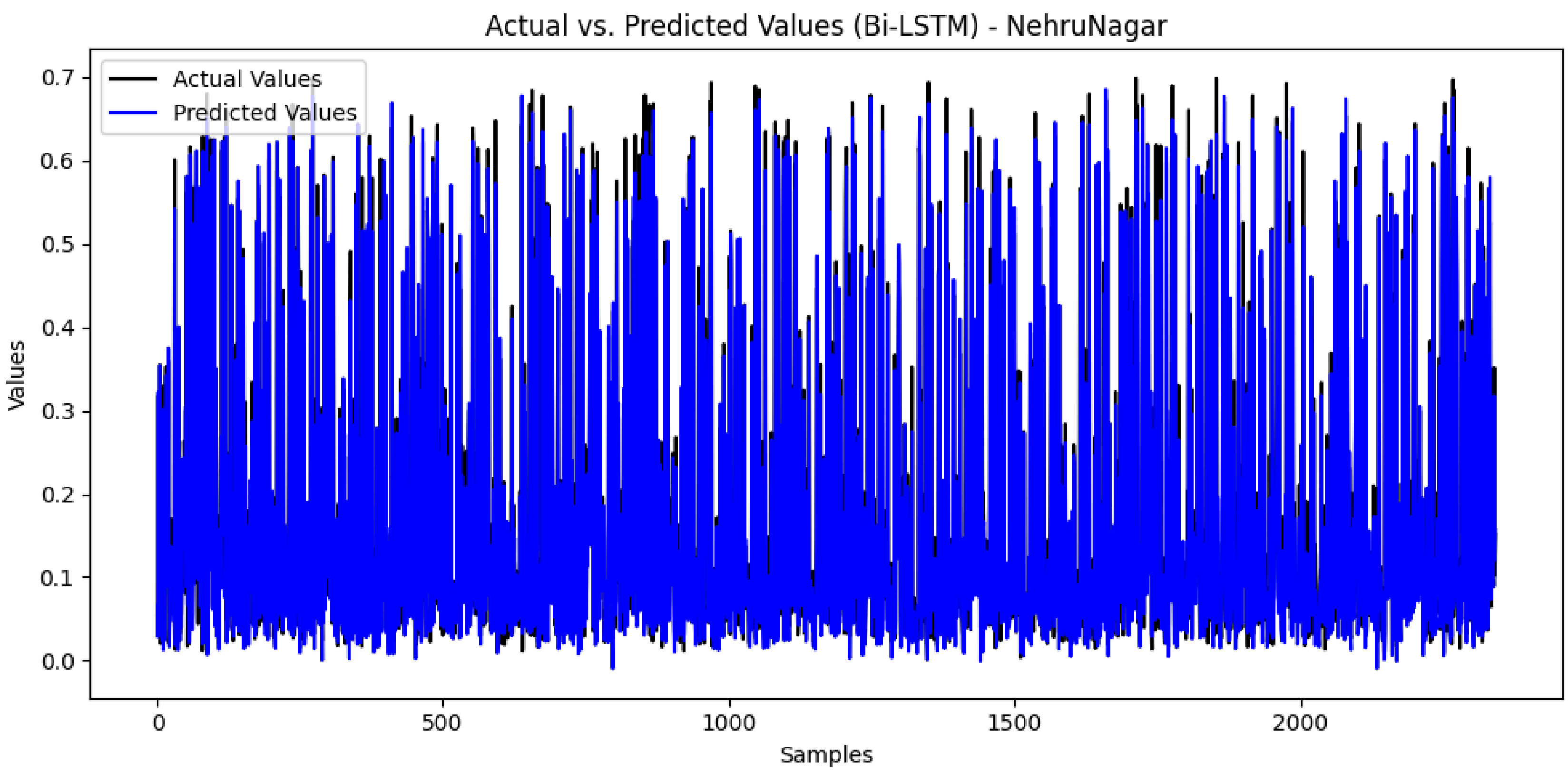


```
Processing Station: Najafgarh
/usr/local/lib/python3.11/dist-packages/pywt/_multilevel.py:43: UserWarning: Level value of 3 is too high: all coefficients will experience boundary effe
  warnings.warn(
Epoch 1/50
/usr/local/lib/python3.11/dist-packages/keras/src/layers/rnn/rnn.py:200: UserWarning: Do not pass an `input_shape`/`input_dim` argument to a layer. When
  super().__init__(**kwargs)
147/147 ━━━━━━━━━━━━━━━━━━━━ 7s 15ms/step - loss: 0.0040 - val_loss: 0.0019
Epoch 2/50
147/147 ━━━━━━━━━━━━━━━━━━━━ 2s 11ms/step - loss: 0.0024 - val_loss: 0.0016
Epoch 3/50
147/147 ━━━━━━━━━━━━━━━━━━━━ 2s 12ms/step - loss: 0.0017 - val_loss: 0.0013
Epoch 4/50
147/147 ━━━━━━━━━━━━━━━━━━━━ 3s 13ms/step - loss: 0.0015 - val_loss: 0.0012
Epoch 5/50
147/147 ━━━━━━━━━━━━━━━━━━━━ 2s 14ms/step - loss: 0.0013 - val_loss: 0.0012
Epoch 6/50
147/147 ━━━━━━━━━━━━━━━━━━━━ 2s 13ms/step - loss: 0.0014 - val_loss: 0.0014
Epoch 7/50
147/147 ━━━━━━━━━━━━━━━━━━━━ 2s 12ms/step - loss: 0.0013 - val_loss: 0.0012
```

```
Epoch 8/50
147/147 ━━━━━━━━━━━━━━━━━━━━ 2s 11ms/step - loss: 0.0011 - val_loss: 0.0010
Epoch 9/50
147/147 ━━━━━━━━━━━━━━━━━━━━ 2s 12ms/step - loss: 9.7740e-04 - val_loss: 0.0010
Epoch 10/50
147/147 ━━━━━━━━━━━━━━━━━━━━ 2s 11ms/step - loss: 0.0011 - val_loss: 9.3009e-04
Epoch 11/50
147/147 ━━━━━━━━━━━━━━━━━━━━ 3s 16ms/step - loss: 0.0011 - val_loss: 9.0361e-04
Epoch 12/50
147/147 ━━━━━━━━━━━━━━━━━━━━ 2s 12ms/step - loss: 9.5381e-04 - val_loss: 8.8509e-04
Epoch 13/50
147/147 ━━━━━━━━━━━━━━━━━━━━ 2s 11ms/step - loss: 9.1065e-04 - val_loss: 7.9623e-04
Epoch 14/50
147/147 ━━━━━━━━━━━━━━━━━━━━ 2s 11ms/step - loss: 8.9612e-04 - val_loss: 9.4128e-04
Epoch 15/50
147/147 ━━━━━━━━━━━━━━━━━━━━ 3s 14ms/step - loss: 8.7668e-04 - val_loss: 8.0692e-04
Epoch 16/50
147/147 ━━━━━━━━━━━━━━━━━━━━ 4s 24ms/step - loss: 7.8852e-04 - val_loss: 8.3003e-04
Epoch 17/50
147/147 ━━━━━━━━━━━━━━━━━━━━ 3s 12ms/step - loss: 7.3614e-04 - val_loss: 7.6576e-04
Epoch 18/50
147/147 ━━━━━━━━━━━━━━━━━━━━ 2s 11ms/step - loss: 6.5672e-04 - val_loss: 7.9162e-04
Epoch 19/50
147/147 ━━━━━━━━━━━━━━━━━━━━ 3s 11ms/step - loss: 6.8614e-04 - val_loss: 7.0832e-04
Epoch 20/50
147/147 ━━━━━━━━━━━━━━━━━━━━ 3s 14ms/step - loss: 6.4589e-04 - val_loss: 6.0970e-04
Epoch 21/50
147/147 ━━━━━━━━━━━━━━━━━━━━ 2s 15ms/step - loss: 5.7204e-04 - val_loss: 6.3878e-04
Epoch 22/50
147/147 ━━━━━━━━━━━━━━━━━━━━ 2s 13ms/step - loss: 5.2362e-04 - val_loss: 7.2470e-04
Epoch 23/50
147/147 ━━━━━━━━━━━━━━━━━━━━ 2s 12ms/step - loss: 5.6709e-04 - val_loss: 5.4760e-04
Epoch 24/50
147/147 ━━━━━━━━━━━━━━━━━━━━ 2s 12ms/step - loss: 5.0357e-04 - val_loss: 5.7252e-04
Epoch 25/50
147/147 ━━━━━━━━━━━━━━━━━━━━ 2s 11ms/step - loss: 5.9855e-04 - val_loss: 5.5793e-04
Epoch 26/50
147/147 ━━━━━━━━━━━━━━━━━━━━ 2s 12ms/step - loss: 5.0995e-04 - val_loss: 5.1274e-04
Epoch 27/50
147/147 ━━━━━━━━━━━━━━━━━━━━ 3s 12ms/step - loss: 5.4958e-04 - val_loss: 5.2306e-04
Epoch 28/50
147/147 ━━━━━━━━━━━━━━━━━━━━ 2s 15ms/step - loss: 5.1681e-04 - val_loss: 4.9522e-04
Epoch 29/50
147/147 ━━━━━━━━━━━━━━━━━━━━ 2s 11ms/step - loss: 4.8275e-04 - val_loss: 4.7531e-04
Epoch 30/50
147/147 ━━━━━━━━━━━━━━━━━━━━ 3s 12ms/step - loss: 4.7013e-04 - val_loss: 5.1032e-04
Epoch 31/50
147/147 ━━━━━━━━━━━━━━━━━━━━ 2s 12ms/step - loss: 4.3539e-04 - val_loss: 4.7312e-04
Epoch 32/50
```

```
147/147 ━━━━━━━━━━━━━━━━━━━━ 3s 12ms/step - loss: 4.6580e-04 - val_loss: 4.6168e-04
Epoch 33/50
147/147 ━━━━━━━━━━━━━━━━━━━━ 3s 15ms/step - loss: 4.6979e-04 - val_loss: 4.2426e-04
Epoch 34/50
147/147 ━━━━━━━━━━━━━━━━━━━━ 2s 14ms/step - loss: 4.1959e-04 - val_loss: 4.5352e-04
Epoch 35/50
147/147 ━━━━━━━━━━━━━━━━━━━━ 2s 12ms/step - loss: 4.1660e-04 - val_loss: 4.3465e-04
Epoch 36/50
147/147 ━━━━━━━━━━━━━━━━━━━━ 3s 12ms/step - loss: 4.2468e-04 - val_loss: 4.5191e-04
Epoch 37/50
147/147 ━━━━━━━━━━━━━━━━━━━━ 3s 12ms/step - loss: 3.8109e-04 - val_loss: 4.1132e-04
Epoch 38/50
147/147 ━━━━━━━━━━━━━━━━━━━━ 3s 14ms/step - loss: 4.4948e-04 - val_loss: 4.3781e-04
Epoch 39/50
147/147 ━━━━━━━━━━━━━━━━━━━━ 2s 15ms/step - loss: 3.9750e-04 - val_loss: 3.9748e-04
Epoch 40/50
147/147 ━━━━━━━━━━━━━━━━━━━━ 2s 11ms/step - loss: 3.5494e-04 - val_loss: 4.1458e-04
Epoch 41/50
147/147 ━━━━━━━━━━━━━━━━━━━━ 4s 20ms/step - loss: 3.8396e-04 - val_loss: 4.1158e-04
Epoch 42/50
147/147 ━━━━━━━━━━━━━━━━━━━━ 4s 14ms/step - loss: 3.8148e-04 - val_loss: 4.2592e-04
Epoch 43/50
147/147 ━━━━━━━━━━━━━━━━━━━━ 2s 16ms/step - loss: 3.5119e-04 - val_loss: 3.9933e-04
Epoch 44/50
147/147 ━━━━━━━━━━━━━━━━━━━━ 2s 12ms/step - loss: 3.8740e-04 - val_loss: 3.7716e-04
Epoch 45/50
147/147 ━━━━━━━━━━━━━━━━━━━━ 2s 11ms/step - loss: 3.4013e-04 - val_loss: 4.0399e-04
Epoch 46/50
147/147 ━━━━━━━━━━━━━━━━━━━━ 3s 12ms/step - loss: 3.6451e-04 - val_loss: 3.9228e-04
Epoch 47/50
147/147 ━━━━━━━━━━━━━━━━━━━━ 2s 12ms/step - loss: 3.3137e-04 - val_loss: 4.0459e-04
Epoch 48/50
147/147 ━━━━━━━━━━━━━━━━━━━━ 3s 12ms/step - loss: 3.3600e-04 - val_loss: 3.7320e-04
Epoch 49/50
147/147 ━━━━━━━━━━━━━━━━━━━━ 2s 15ms/step - loss: 3.2893e-04 - val_loss: 3.5074e-04
Epoch 50/50
147/147 ━━━━━━━━━━━━━━━━━━━━ 2s 12ms/step - loss: 3.7353e-04 - val_loss: 3.5759e-04
```

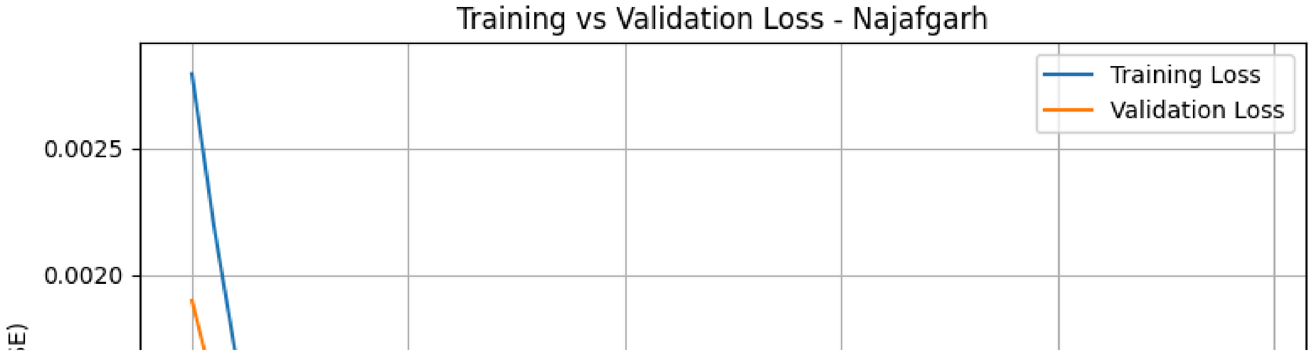

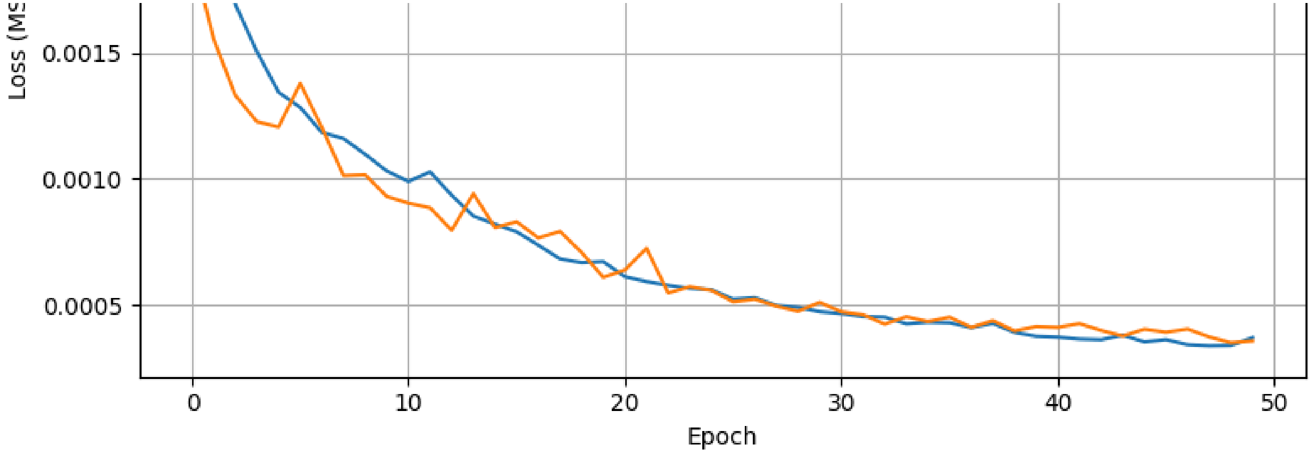


```
74/74 ━━━━━━━━━━━━━━━━━━━━ 1s 8ms/step
```

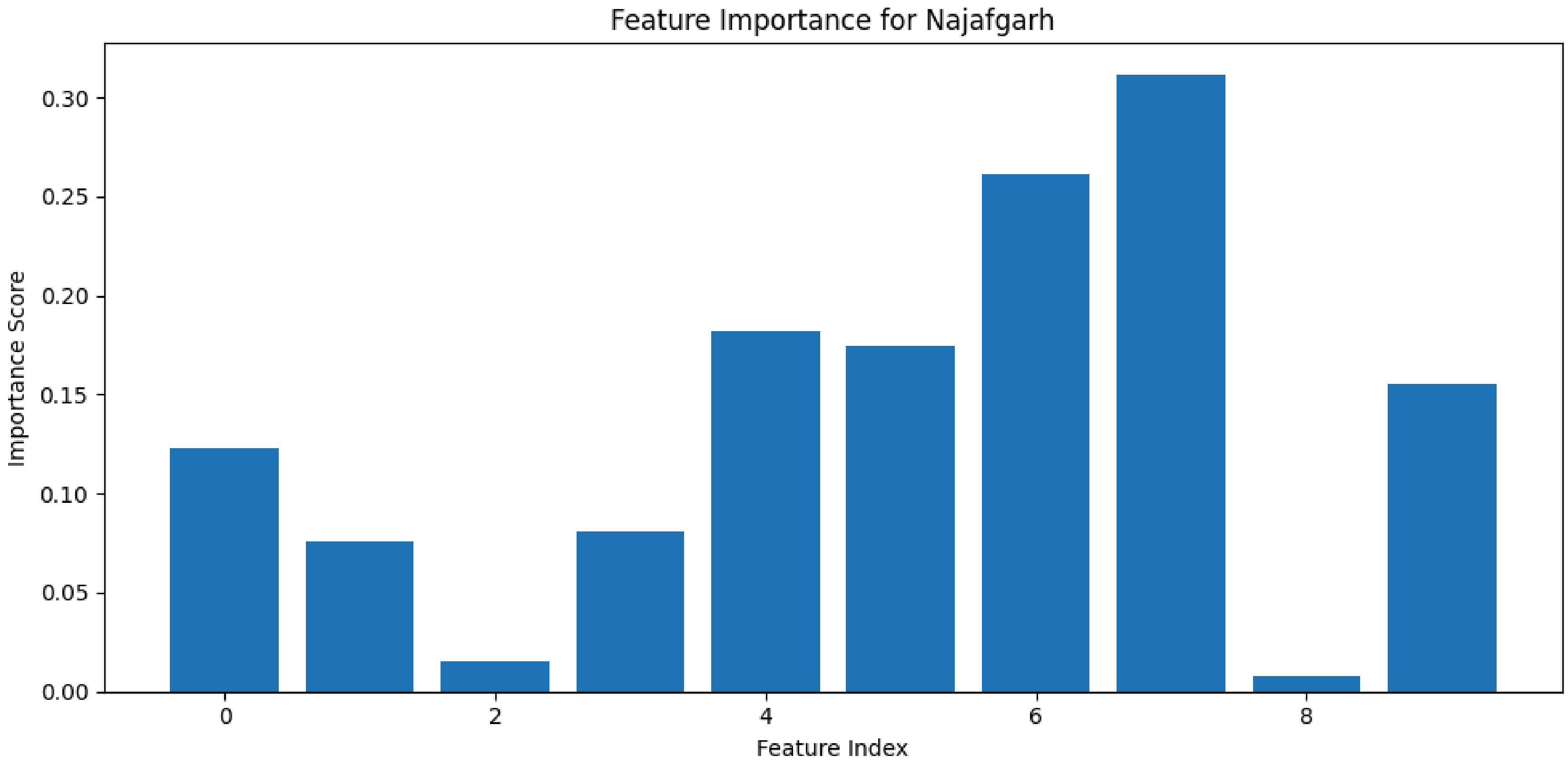

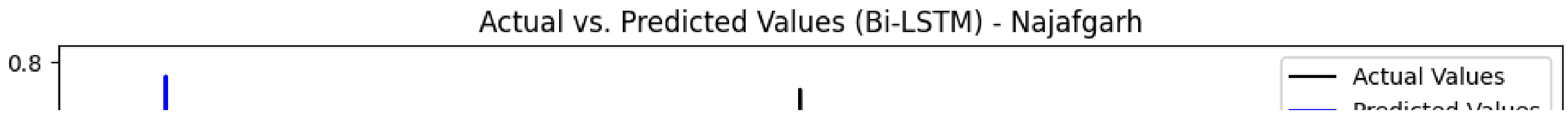

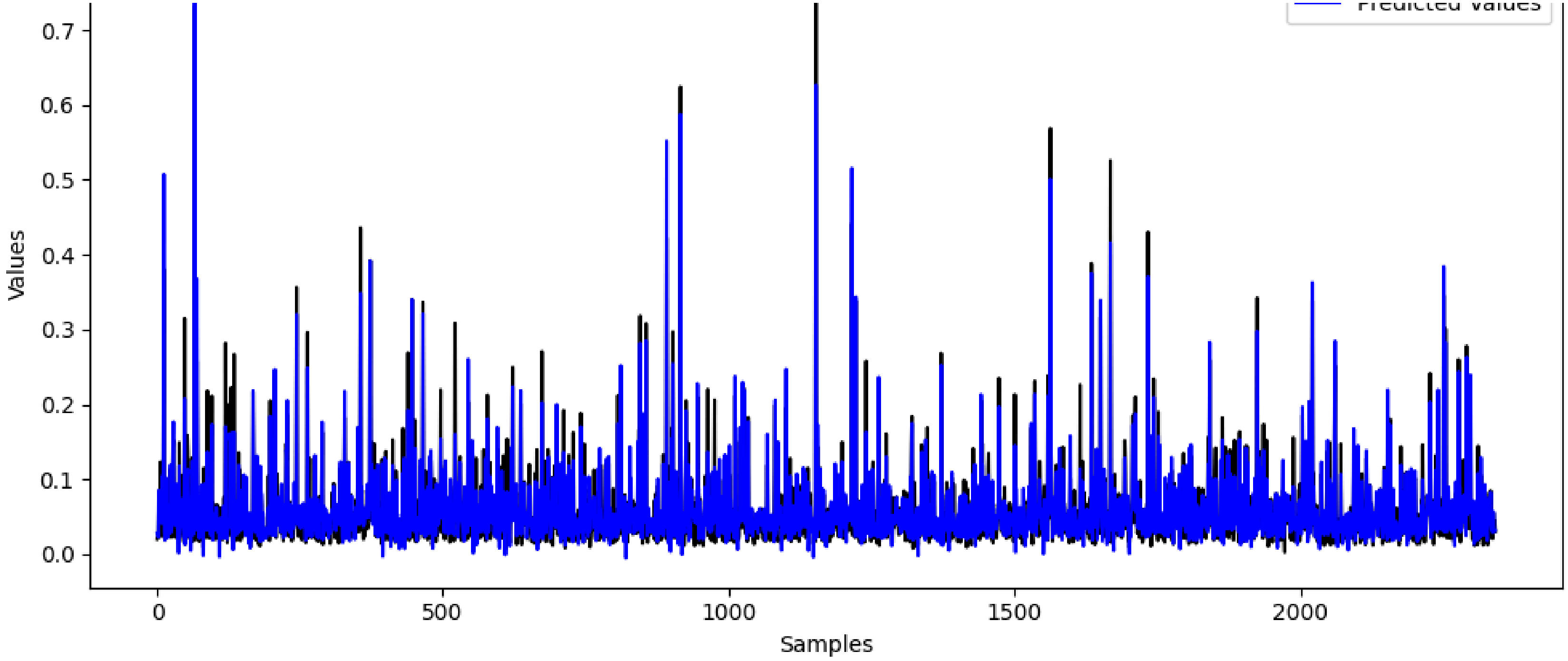


```
Processing Station: Okhla
/usr/local/lib/python3.11/dist-packages/pywt/_multilevel.py:43: UserWarning: Level value of 3 is too high: all coefficients will experience boundary effe
  warnings.warn(
Epoch 1/50
/usr/local/lib/python3.11/dist-packages/keras/src/layers/rnn/rnn.py:200: UserWarning: Do not pass an `input_shape`/`input_dim` argument to a layer. When
  super().__init__(**kwargs)
147/147 ━━━━━━━━━━━━━━━━━━━━ 6s 18ms/step - loss: 0.0111 - val_loss: 0.0068
Epoch 2/50
147/147 ━━━━━━━━━━━━━━━━━━━━ 4s 12ms/step - loss: 0.0061 - val_loss: 0.0036
Epoch 3/50
147/147 ━━━━━━━━━━━━━━━━━━━━ 2s 11ms/step - loss: 0.0034 - val_loss: 0.0027
Epoch 4/50
147/147 ━━━━━━━━━━━━━━━━━━━━ 3s 11ms/step - loss: 0.0026 - val_loss: 0.0021
Epoch 5/50
147/147 ━━━━━━━━━━━━━━━━━━━━ 3s 14ms/step - loss: 0.0022 - val_loss: 0.0017
Epoch 6/50
147/147 ━━━━━━━━━━━━━━━━━━━━ 2s 15ms/step - loss: 0.0018 - val_loss: 0.0017
Epoch 7/50
147/147 ━━━━━━━━━━━━━━━━━━━━ 2s 12ms/step - loss: 0.0018 - val_loss: 0.0016
Epoch 8/50
147/147 ━━━━━━━━━━━━━━━━━━━━ 2s 11ms/step - loss: 0.0016 - val_loss: 0.0013
Epoch 9/50
147/147 ━━━━━━━━━━━━━━━━━━━━ 2s 11ms/step - loss: 0.0014 - val_loss: 0.0012
Epoch 10/50
```

```
147/147 ━━━━━━━━━━━━━━━━━━━━ 2s 12ms/step - loss: 0.0013 - val_loss: 0.0011
Epoch 11/50
147/147 ━━━━━━━━━━━━━━━━━━━━ 2s 11ms/step - loss: 0.0012 - val_loss: 0.0011
Epoch 12/50
147/147 ━━━━━━━━━━━━━━━━━━━━ 2s 15ms/step - loss: 0.0011 - val_loss: 0.0014
Epoch 13/50
147/147 ━━━━━━━━━━━━━━━━━━━━ 2s 13ms/step - loss: 0.0012 - val_loss: 0.0010
Epoch 14/50
147/147 ━━━━━━━━━━━━━━━━━━━━ 2s 11ms/step - loss: 0.0010 - val_loss: 9.3523e-04
Epoch 15/50
147/147 ━━━━━━━━━━━━━━━━━━━━ 2s 11ms/step - loss: 9.1416e-04 - val_loss: 0.0010
Epoch 16/50
147/147 ━━━━━━━━━━━━━━━━━━━━ 3s 12ms/step - loss: 0.0010 - val_loss: 8.8097e-04
Epoch 17/50
147/147 ━━━━━━━━━━━━━━━━━━━━ 2s 11ms/step - loss: 8.5751e-04 - val_loss: 7.9664e-04
Epoch 18/50
147/147 ━━━━━━━━━━━━━━━━━━━━ 2s 13ms/step - loss: 8.9528e-04 - val_loss: 7.9471e-04
Epoch 19/50
147/147 ━━━━━━━━━━━━━━━━━━━━ 2s 12ms/step - loss: 7.6943e-04 - val_loss: 8.5785e-04
Epoch 20/50
147/147 ━━━━━━━━━━━━━━━━━━━━ 2s 12ms/step - loss: 8.2702e-04 - val_loss: 8.3622e-04
Epoch 21/50
147/147 ━━━━━━━━━━━━━━━━━━━━ 2s 11ms/step - loss: 7.7988e-04 - val_loss: 8.7090e-04
Epoch 22/50
147/147 ━━━━━━━━━━━━━━━━━━━━ 3s 12ms/step - loss: 8.6956e-04 - val_loss: 7.4476e-04
Epoch 23/50
147/147 ━━━━━━━━━━━━━━━━━━━━ 2s 12ms/step - loss: 7.2399e-04 - val_loss: 8.5201e-04
Epoch 24/50
147/147 ━━━━━━━━━━━━━━━━━━━━ 3s 15ms/step - loss: 7.8030e-04 - val_loss: 7.5872e-04
Epoch 25/50
147/147 ━━━━━━━━━━━━━━━━━━━━ 2s 13ms/step - loss: 7.0014e-04 - val_loss: 6.7582e-04
Epoch 26/50
147/147 ━━━━━━━━━━━━━━━━━━━━ 2s 11ms/step - loss: 6.9449e-04 - val_loss: 7.4663e-04
Epoch 27/50
147/147 ━━━━━━━━━━━━━━━━━━━━ 3s 12ms/step - loss: 6.8235e-04 - val_loss: 6.6361e-04
Epoch 28/50
147/147 ━━━━━━━━━━━━━━━━━━━━ 2s 11ms/step - loss: 6.4945e-04 - val_loss: 8.1503e-04
Epoch 29/50
147/147 ━━━━━━━━━━━━━━━━━━━━ 3s 12ms/step - loss: 6.4724e-04 - val_loss: 7.1157e-04
Epoch 30/50
147/147 ━━━━━━━━━━━━━━━━━━━━ 3s 13ms/step - loss: 7.2992e-04 - val_loss: 6.5692e-04
Epoch 31/50
147/147 ━━━━━━━━━━━━━━━━━━━━ 2s 12ms/step - loss: 6.3400e-04 - val_loss: 6.1713e-04
Epoch 32/50
147/147 ━━━━━━━━━━━━━━━━━━━━ 2s 11ms/step - loss: 6.0276e-04 - val_loss: 6.3679e-04
Epoch 33/50
147/147 ━━━━━━━━━━━━━━━━━━━━ 2s 11ms/step - loss: 7.2924e-04 - val_loss: 6.0970e-04
Epoch 34/50
147/147 ━━━━━━━━━━━━━━━━━━━━ 2s 12ms/step - loss: 5.8013e-04 - val_loss: 6.0126e-04
```

```
Epoch 35/50
147/147 ━━━━━━━━━━━━━━━━━━━━ 3s 15ms/step - loss: 6.0125e-04 - val_loss: 6.2575e-04
Epoch 36/50
147/147 ━━━━━━━━━━━━━━━━━━━━ 2s 13ms/step - loss: 5.8058e-04 - val_loss: 6.8873e-04
Epoch 37/50
147/147 ━━━━━━━━━━━━━━━━━━━━ 2s 11ms/step - loss: 6.1030e-04 - val_loss: 7.1339e-04
Epoch 38/50
147/147 ━━━━━━━━━━━━━━━━━━━━ 3s 12ms/step - loss: 5.6889e-04 - val_loss: 6.0692e-04
Epoch 39/50
147/147 ━━━━━━━━━━━━━━━━━━━━ 3s 12ms/step - loss: 5.4799e-04 - val_loss: 5.8203e-04
Epoch 40/50
147/147 ━━━━━━━━━━━━━━━━━━━━ 3s 16ms/step - loss: 5.2926e-04 - val_loss: 5.9871e-04
Epoch 41/50
147/147 ━━━━━━━━━━━━━━━━━━━━ 2s 13ms/step - loss: 5.4182e-04 - val_loss: 7.1349e-04
Epoch 42/50
147/147 ━━━━━━━━━━━━━━━━━━━━ 2s 12ms/step - loss: 5.9661e-04 - val_loss: 6.0539e-04
Epoch 43/50
147/147 ━━━━━━━━━━━━━━━━━━━━ 2s 11ms/step - loss: 4.9505e-04 - val_loss: 7.4008e-04
Epoch 44/50
147/147 ━━━━━━━━━━━━━━━━━━━━ 3s 12ms/step - loss: 5.0956e-04 - val_loss: 6.0369e-04
Epoch 45/50
147/147 ━━━━━━━━━━━━━━━━━━━━ 3s 12ms/step - loss: 4.7468e-04 - val_loss: 6.0342e-04
Epoch 46/50
147/147 ━━━━━━━━━━━━━━━━━━━━ 5s 29ms/step - loss: 5.5001e-04 - val_loss: 5.7385e-04
Epoch 47/50
147/147 ━━━━━━━━━━━━━━━━━━━━ 3s 13ms/step - loss: 4.7045e-04 - val_loss: 5.3514e-04
Epoch 48/50
147/147 ━━━━━━━━━━━━━━━━━━━━ 4s 20ms/step - loss: 4.8441e-04 - val_loss: 6.3151e-04
Epoch 49/50
147/147 ━━━━━━━━━━━━━━━━━━━━ 4s 26ms/step - loss: 4.9114e-04 - val_loss: 6.1542e-04
Epoch 50/50
147/147 ━━━━━━━━━━━━━━━━━━━━ 2s 13ms/step - loss: 4.4508e-04 - val_loss: 7.1879e-04
```

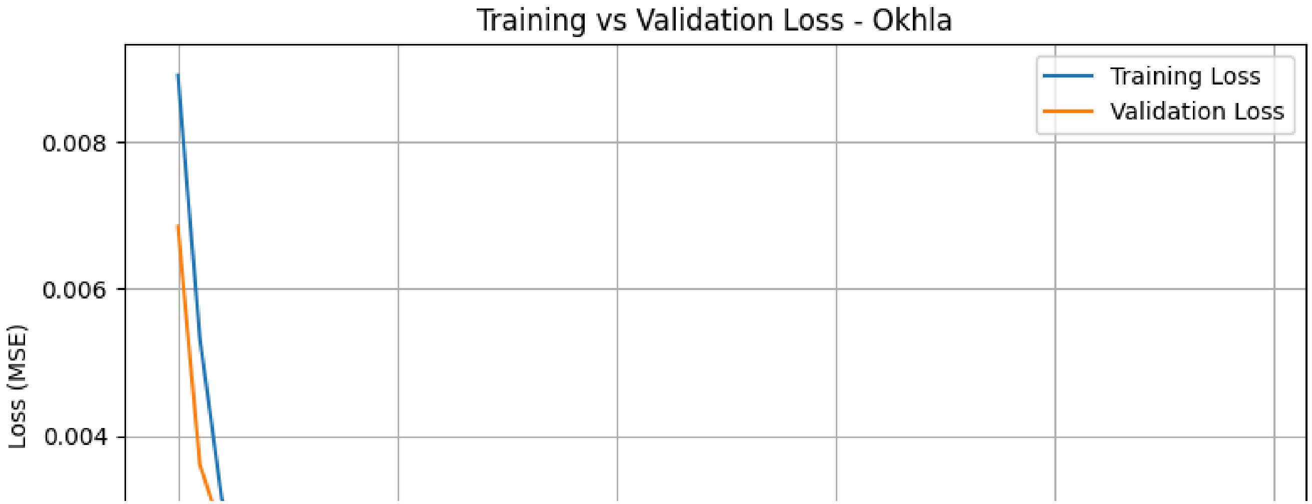

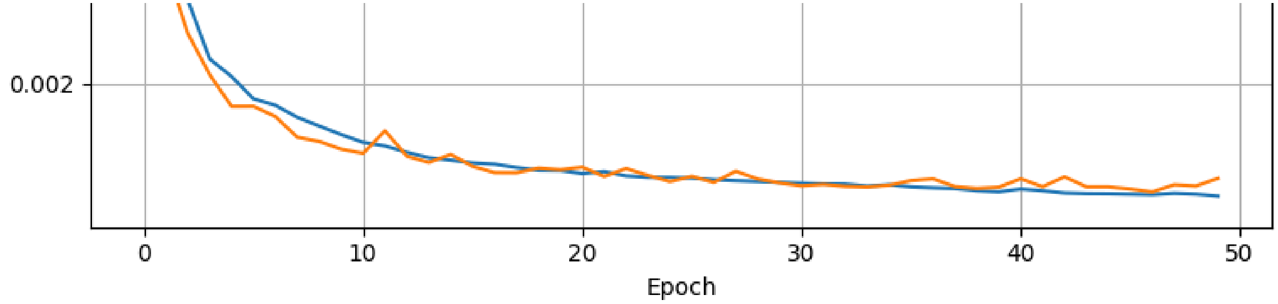

74/74 ━━━━━━━━━━━━━━━━━━━━ 1s 8ms/step

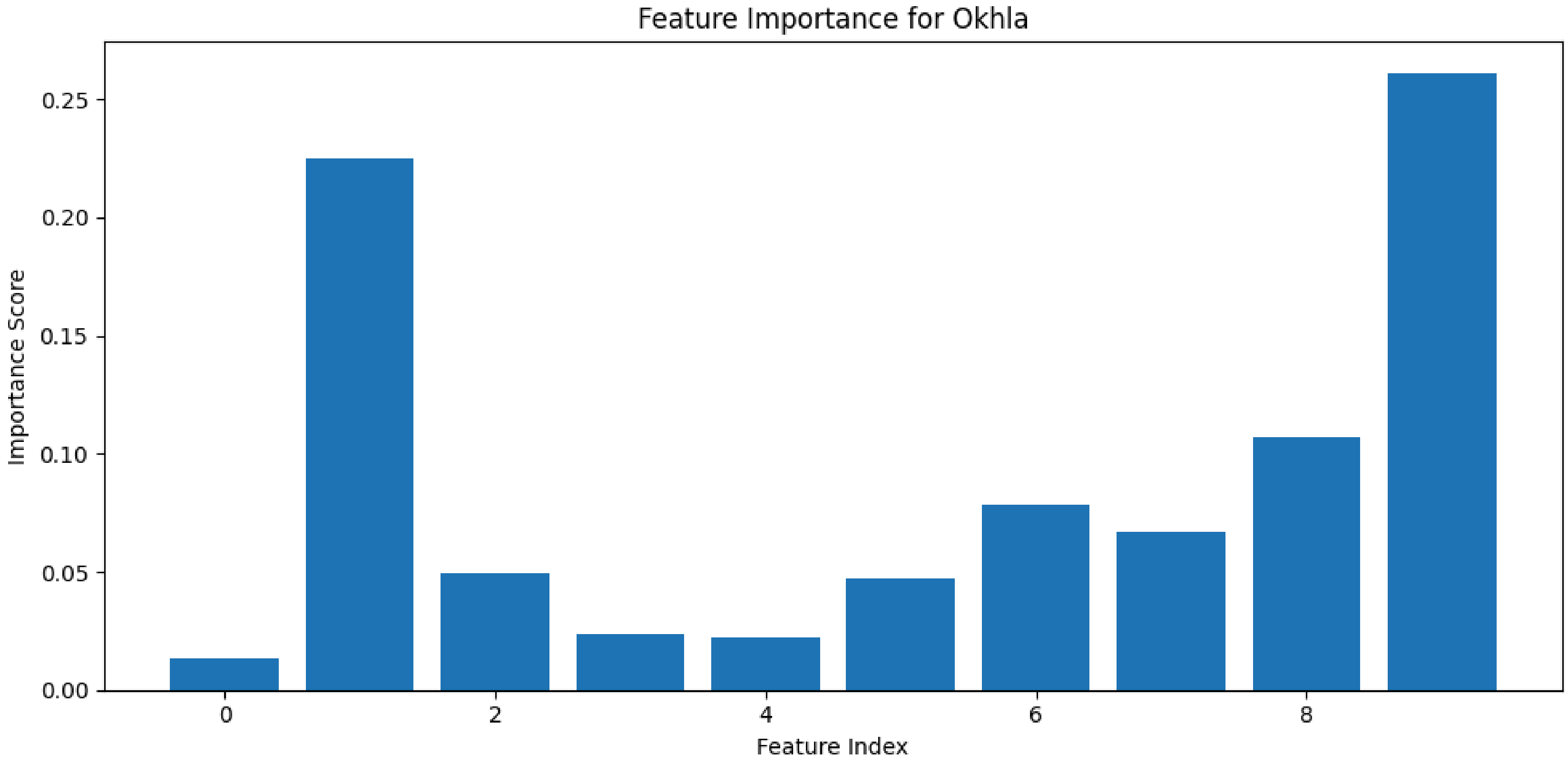

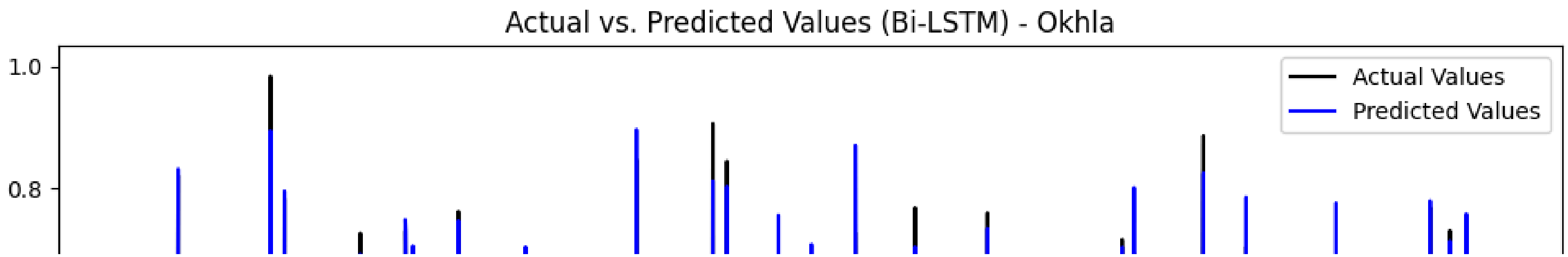

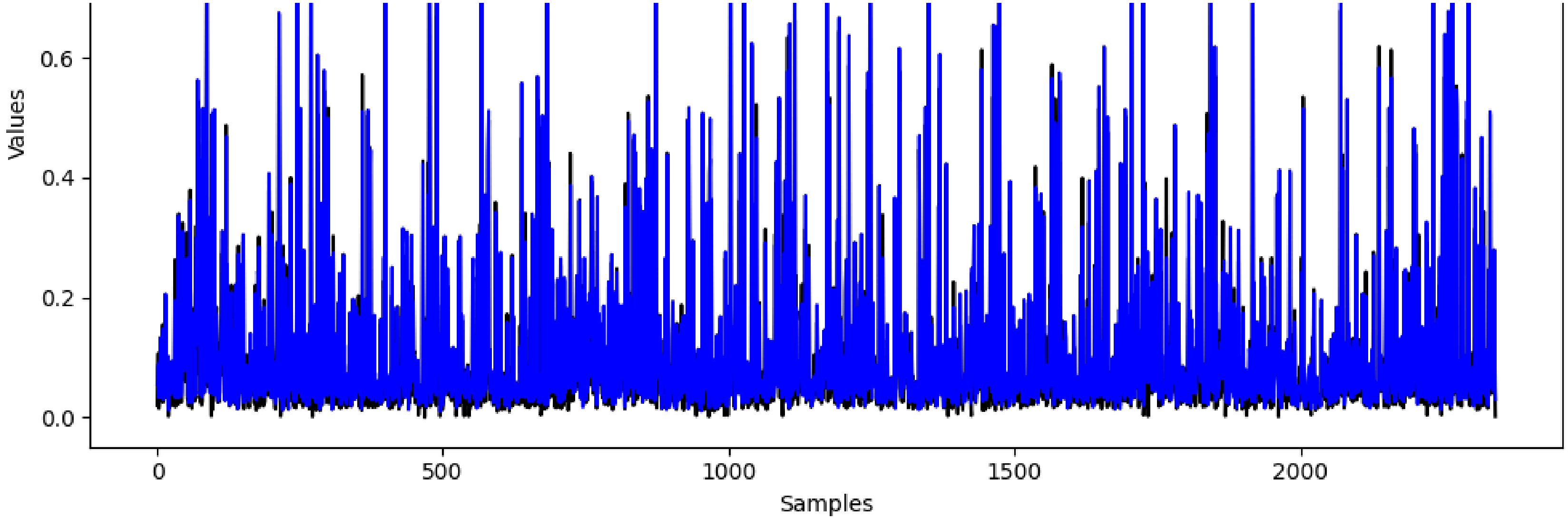


```
Final Model Evaluation Across Stations:

Station: AshokVihar
MSE: 0.0006
MAE: 0.0174
RMSE: 0.0245
R² Score: 0.9451

Station: DCStadium
MSE: 0.0009
MAE: 0.0202
RMSE: 0.0307
R² Score: 0.9816

Station: DwarkaSec8
MSE: 0.0008
MAE: 0.0175
RMSE: 0.0276
R² Score: 0.9396

Station: NehruNagar
MSE: 0.0006
MAE: 0.0178
RMSE: 0.0245
R² Score: 0.9807

Station: Najafgarh
MSE: 0.0004
MAE: 0.0128
```

```
RMSE: 0.0189
R² Score: 0.8911

Station: Okhla
MSE: 0.0007
MAE: 0.0182
RMSE: 0.0268
R² Score: 0.9574
```

*Without Feature extraction *

```
import numpy as np
import pandas as pd
import matplotlib.pyplot as plt
from sklearn.preprocessing import MinMaxScaler
from sklearn.model_selection import train_test_split
from sklearn.metrics import mean_squared_error, mean_absolute_error, r2_score
import tensorflow as tf
from tensorflow.keras.models import Sequential
from tensorflow.keras.layers import LSTM, Dense, Bidirectional

# ============================
# Hybrid AOAOA Optimizer
# ============================
class HybridOptimizer:
    def __init__(self, objective_function, lower_bound, upper_bound, population_size, iterations):
        self.objective_function = objective_function
        self.lower_bound = np.array(lower_bound)
        self.upper_bound = np.array(upper_bound)
        self.population_size = population_size
        self.iterations = iterations
        self.population = np.random.uniform(low=self.lower_bound, high=self.upper_bound,
                                            size=(population_size, len(lower_bound)))
        self.best_solution = None
        self.best_fitness = float('inf')

    def optimize(self):
        for _ in range(self.iterations):
            for i in range(self.population_size):
                perturbation = np.random.uniform(-0.1, 0.1, size=self.population.shape[1])
                candidate_solution_aquila = self.population[i] + perturbation
                candidate_solution_aquila = np.clip(candidate_solution_aquila, self.lower_bound, self.upper_bound)
                fitness_aquila = self.objective_function(candidate_solution_aquila)

                if fitness_aquila < self.best_fitness:
                    self.best_fitness = fitness_aquila
                    self.best_solution = candidate_solution_aquila

            for i in range(self.population_size):
                partner_idx = np.random.randint(self.population_size)
                partner = self.population[partner_idx]
```

```
                candidate_solution_arithmetic = (self.population[i] + partner) / 2
                candidate_solution_arithmetic = np.clip(candidate_solution_arithmetic, self.lower_bound, self.upper_bound)
                fitness_arithmetic = self.objective_function(candidate_solution_arithmetic)

                if fitness_arithmetic < self.best_fitness:
                    self.best_fitness = fitness_arithmetic
                    self.best_solution = candidate_solution_arithmetic

        return self.best_solution

# ============================
# Bi-LSTM Model Definition
# ============================
def build_lstm_model(input_shape):
    model = Sequential([
        Bidirectional(LSTM(50, return_sequences=True, input_shape=input_shape)),
        Bidirectional(LSTM(50, return_sequences=False)),
        Dense(1)
    ])
    model.compile(optimizer='adam', loss='mean_squared_error')
    return model

# ============================
# Training & Evaluation
# ============================
def evaluate_model(X, y, station_name):
    if X.shape[1] == 0:
        raise ValueError("No features selected! Adjust AOAOA feature selection.")
    X_train, X_test, y_train, y_test = train_test_split(X, y, test_size=0.2, random_state=42)
    X_train = X_train.reshape(X_train.shape[0], X_train.shape[1], 1)
    X_test = X_test.reshape(X_test.shape[0], X_test.shape[1], 1)

    model = build_lstm_model((X_train.shape[1], 1))
    history = model.fit(X_train, y_train, epochs=50, batch_size=64,
                        validation_data=(X_test, y_test), verbose=1)

    # Training vs Validation Loss Plot
    plt.figure(figsize=(8, 5))
    plt.plot(history.history['loss'], label='Training Loss')
    plt.plot(history.history['val_loss'], label='Validation Loss')
    plt.title(f'Training vs Validation Loss - {station_name}')
    plt.xlabel('Epoch')
    plt.ylabel('Loss (MSE)')
    plt.legend()
```

```
    plt.grid(True)
    plt.tight_layout()
    plt.show()

    y_pred = model.predict(X_test)

    mse = mean_squared_error(y_test, y_pred)
    mae = mean_absolute_error(y_test, y_pred)
    rmse = np.sqrt(mse)
    r2 = r2_score(y_test, y_pred)

    return mse, mae, rmse, r2, y_test, y_pred

# ============================
# Multi-Station Processing
# ============================
stations = {
    'AshokVihar': '/content/AshokVihar_Hourly.csv',
    'DCStadium': '/content/DCStadium_Hourly.csv',
    'DwarkaSec8': '/content/DwarkaSec8_Hourly.csv',
    'NehruNagar': '/content/NehruNagar_Hourly.csv',
    'Najafgarh': '/content/Najafgarh_Hourly.csv',
    'Okhla': '/content/Okhla_Hourly.csv'
}

threshold = 0.40
results = {}

for station, file_path in stations.items():
    print(f"\nProcessing Station: {station}")

    # Load Data
    df = pd.read_csv(file_path)

    # Preprocessing
    scaler = MinMaxScaler()
    X_full = scaler.fit_transform(df.iloc[:, :-1].values)
    y = scaler.fit_transform(df.iloc[:, -1].values.reshape(-1, 1))

    # Feature Selection using original (preprocessed) features
    objective_function = lambda x: np.sum(x**2)
    hybrid_optimizer = HybridOptimizer(objective_function,
                                       lower_bound=[-1] * X_full.shape[1],
                                       upper_bound=[1] * X_full.shape[1],
```

```
                                    population_size=50,
                                    iterations=100)
    selected_features = hybrid_optimizer.optimize()

    # Select features above threshold
    X_selected = X_full[:, selected_features > threshold]

    # Final Model Evaluation
    X_final = X_selected if X_selected.shape[1] > 0 else X_full
    mse, mae, rmse, r2, y_test, y_pred = evaluate_model(X_final, y, station)

    # Store results
    results[station] = {"MSE": mse, "MAE": mae, "RMSE": rmse, "R² Score": r2}

    # Plot Feature Importance
    plt.figure(figsize=(10, 5))
    feature_importance = np.abs(selected_features)
    plt.bar(range(len(feature_importance)), feature_importance)
    plt.xlabel('Feature Index')
    plt.ylabel('Importance Score')
    plt.title(f'Feature Importance for {station}')
    plt.tight_layout()
    plt.show()

    # Plot Actual vs Predicted
    plt.figure(figsize=(10, 5))
    plt.plot(y_test, label="Actual Values", color='black')
    plt.plot(y_pred, label="Predicted Values", color='blue')
    plt.xlabel('Samples')
    plt.ylabel('Values')
    plt.title(f'Actual vs. Predicted Values (Bi-LSTM) - {station}')
    plt.legend()
    plt.tight_layout()
    plt.show()

# Print Final Results
print("\nFinal Model Evaluation Across Stations:")
for station, metrics in results.items():
    print(f"\nStation: {station}")
    for metric, value in metrics.items():
        print(f"{metric}: {value:.4f}")
```

```
Processing Station: AshokVihar
Epoch 1/50
/usr/local/lib/python3.11/dist-packages/keras/src/layers/rnn/rnn.py:200: UserWarning: Do not pass an `input_shape`/`input_dim` argument to a layer. When
  super().__init__(**kwargs)
147/147 ━━━━━━━━━━━━━━━━━━━━ 10s 23ms/step - loss: 0.0116 - val_loss: 0.0088
Epoch 2/50
147/147 ━━━━━━━━━━━━━━━━━━━━ 4s 18ms/step - loss: 0.0089 - val_loss: 0.0085
Epoch 3/50
147/147 ━━━━━━━━━━━━━━━━━━━━ 3s 21ms/step - loss: 0.0086 - val_loss: 0.0086
Epoch 4/50
147/147 ━━━━━━━━━━━━━━━━━━━━ 3s 9ms/step - loss: 0.0090 - val_loss: 0.0086
Epoch 5/50
147/147 ━━━━━━━━━━━━━━━━━━━━ 1s 9ms/step - loss: 0.0090 - val_loss: 0.0084
Epoch 6/50
147/147 ━━━━━━━━━━━━━━━━━━━━ 3s 9ms/step - loss: 0.0088 - val_loss: 0.0084
Epoch 7/50
147/147 ━━━━━━━━━━━━━━━━━━━━ 3s 9ms/step - loss: 0.0086 - val_loss: 0.0084
Epoch 8/50
147/147 ━━━━━━━━━━━━━━━━━━━━ 2s 11ms/step - loss: 0.0082 - val_loss: 0.0083
Epoch 9/50
147/147 ━━━━━━━━━━━━━━━━━━━━ 2s 12ms/step - loss: 0.0081 - val_loss: 0.0083
Epoch 10/50
147/147 ━━━━━━━━━━━━━━━━━━━━ 2s 9ms/step - loss: 0.0088 - val_loss: 0.0085
Epoch 11/50
147/147 ━━━━━━━━━━━━━━━━━━━━ 1s 9ms/step - loss: 0.0085 - val_loss: 0.0082
Epoch 12/50
147/147 ━━━━━━━━━━━━━━━━━━━━ 3s 10ms/step - loss: 0.0082 - val_loss: 0.0083
Epoch 13/50
147/147 ━━━━━━━━━━━━━━━━━━━━ 1s 9ms/step - loss: 0.0085 - val_loss: 0.0084
Epoch 14/50
147/147 ━━━━━━━━━━━━━━━━━━━━ 3s 11ms/step - loss: 0.0089 - val_loss: 0.0082
Epoch 15/50
147/147 ━━━━━━━━━━━━━━━━━━━━ 2s 12ms/step - loss: 0.0081 - val_loss: 0.0082
Epoch 16/50
147/147 ━━━━━━━━━━━━━━━━━━━━ 2s 9ms/step - loss: 0.0080 - val_loss: 0.0081
Epoch 17/50
147/147 ━━━━━━━━━━━━━━━━━━━━ 1s 9ms/step - loss: 0.0090 - val_loss: 0.0082
Epoch 18/50
147/147 ━━━━━━━━━━━━━━━━━━━━ 3s 9ms/step - loss: 0.0078 - val_loss: 0.0081
Epoch 19/50
147/147 ━━━━━━━━━━━━━━━━━━━━ 1s 9ms/step - loss: 0.0081 - val_loss: 0.0082
Epoch 20/50
147/147 ━━━━━━━━━━━━━━━━━━━━ 2s 10ms/step - loss: 0.0084 - val_loss: 0.0081
Epoch 21/50
147/147 ━━━━━━━━━━━━━━━━━━━━ 2s 11ms/step - loss: 0.0086 - val_loss: 0.0082
Epoch 22/50
147/147 ━━━━━━━━━━━━━━━━━━━━ 2s 9ms/step - loss: 0.0084 - val_loss: 0.0081
Epoch 23/50
```

```
147/147 ━━━━━━━━━━━━━━━━━━━━ 1s 9ms/step - loss: 0.0082 - val_loss: 0.0081
Epoch 24/50
147/147 ━━━━━━━━━━━━━━━━━━━━ 3s 9ms/step - loss: 0.0082 - val_loss: 0.0082
Epoch 25/50
147/147 ━━━━━━━━━━━━━━━━━━━━ 3s 9ms/step - loss: 0.0092 - val_loss: 0.0081
Epoch 26/50
147/147 ━━━━━━━━━━━━━━━━━━━━ 3s 11ms/step - loss: 0.0084 - val_loss: 0.0081
Epoch 27/50
147/147 ━━━━━━━━━━━━━━━━━━━━ 2s 13ms/step - loss: 0.0081 - val_loss: 0.0080
Epoch 28/50
147/147 ━━━━━━━━━━━━━━━━━━━━ 1s 9ms/step - loss: 0.0083 - val_loss: 0.0080
Epoch 29/50
147/147 ━━━━━━━━━━━━━━━━━━━━ 1s 9ms/step - loss: 0.0083 - val_loss: 0.0079
Epoch 30/50
147/147 ━━━━━━━━━━━━━━━━━━━━ 1s 9ms/step - loss: 0.0084 - val_loss: 0.0079
Epoch 31/50
147/147 ━━━━━━━━━━━━━━━━━━━━ 3s 9ms/step - loss: 0.0083 - val_loss: 0.0079
Epoch 32/50
147/147 ━━━━━━━━━━━━━━━━━━━━ 1s 9ms/step - loss: 0.0083 - val_loss: 0.0079
Epoch 33/50
147/147 ━━━━━━━━━━━━━━━━━━━━ 1s 9ms/step - loss: 0.0082 - val_loss: 0.0079
Epoch 34/50
147/147 ━━━━━━━━━━━━━━━━━━━━ 2s 13ms/step - loss: 0.0079 - val_loss: 0.0078
Epoch 35/50
147/147 ━━━━━━━━━━━━━━━━━━━━ 2s 11ms/step - loss: 0.0081 - val_loss: 0.0078
Epoch 36/50
147/147 ━━━━━━━━━━━━━━━━━━━━ 1s 9ms/step - loss: 0.0080 - val_loss: 0.0076
Epoch 37/50
147/147 ━━━━━━━━━━━━━━━━━━━━ 1s 9ms/step - loss: 0.0079 - val_loss: 0.0077
Epoch 38/50
147/147 ━━━━━━━━━━━━━━━━━━━━ 1s 10ms/step - loss: 0.0080 - val_loss: 0.0076
Epoch 39/50
147/147 ━━━━━━━━━━━━━━━━━━━━ 2s 9ms/step - loss: 0.0080 - val_loss: 0.0077
Epoch 40/50
147/147 ━━━━━━━━━━━━━━━━━━━━ 3s 10ms/step - loss: 0.0077 - val_loss: 0.0076
Epoch 41/50
147/147 ━━━━━━━━━━━━━━━━━━━━ 2s 13ms/step - loss: 0.0083 - val_loss: 0.0075
Epoch 42/50
147/147 ━━━━━━━━━━━━━━━━━━━━ 1s 9ms/step - loss: 0.0072 - val_loss: 0.0075
Epoch 43/50
147/147 ━━━━━━━━━━━━━━━━━━━━ 3s 9ms/step - loss: 0.0073 - val_loss: 0.0074
Epoch 44/50
147/147 ━━━━━━━━━━━━━━━━━━━━ 3s 9ms/step - loss: 0.0077 - val_loss: 0.0077
Epoch 45/50
147/147 ━━━━━━━━━━━━━━━━━━━━ 3s 9ms/step - loss: 0.0076 - val_loss: 0.0072
Epoch 46/50
147/147 ━━━━━━━━━━━━━━━━━━━━ 2s 11ms/step - loss: 0.0071 - val_loss: 0.0073
Epoch 47/50
147/147 ━━━━━━━━━━━━━━━━━━━━ 2s 14ms/step - loss: 0.0069 - val_loss: 0.0077
```

```
Epoch 48/50
147/147 ━━━━━━━━━━━━━━━━━━━━ 1s 9ms/step - loss: 0.0075 - val_loss: 0.0073
Epoch 49/50
147/147 ━━━━━━━━━━━━━━━━━━━━ 1s 9ms/step - loss: 0.0067 - val_loss: 0.0071
Epoch 50/50
147/147 ━━━━━━━━━━━━━━━━━━━━ 1s 9ms/step - loss: 0.0072 - val_loss: 0.0071
```

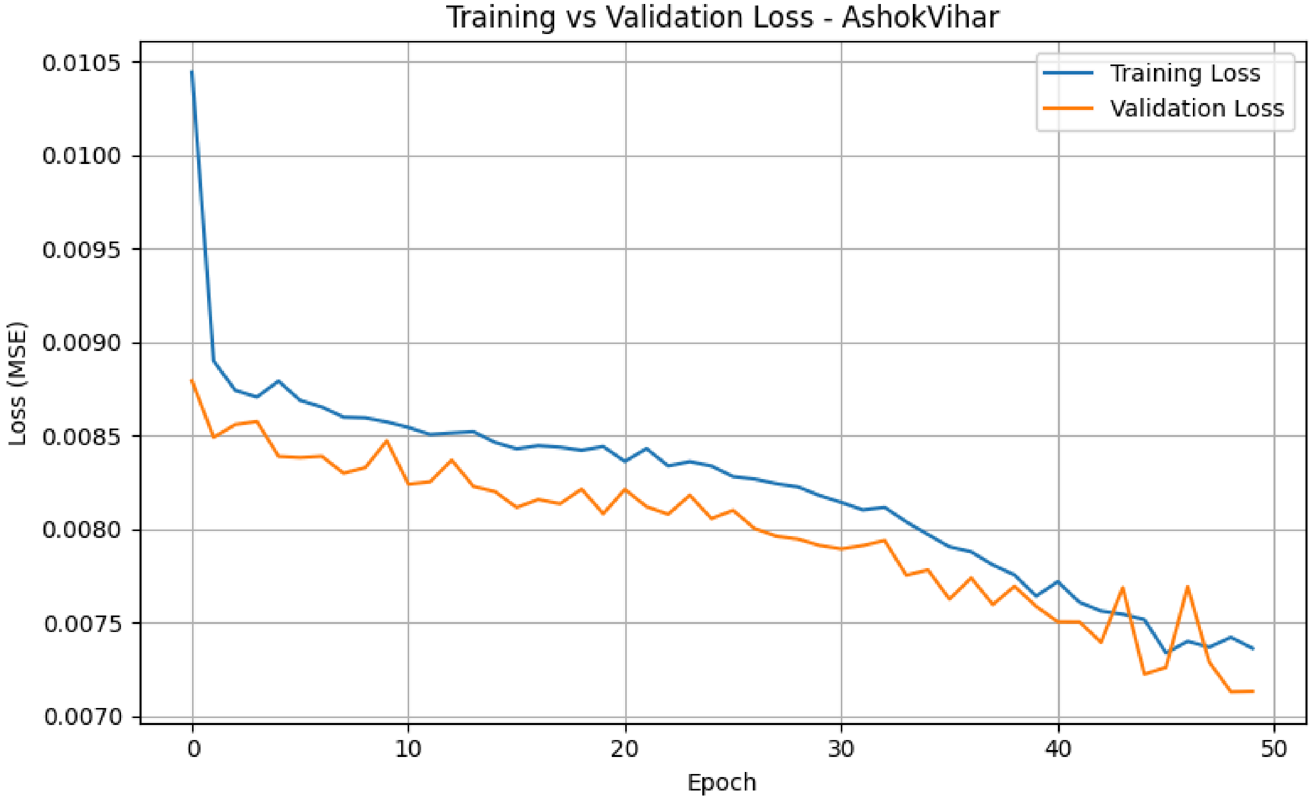


```
74/74 ━━━━━━━━━━━━━━━━━━━━ 1s 7ms/step
```

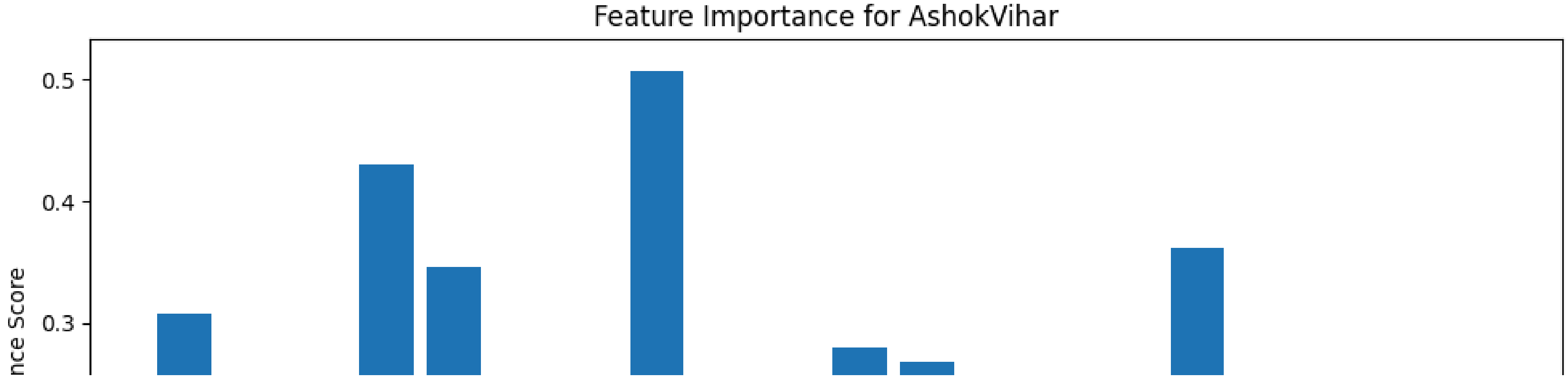

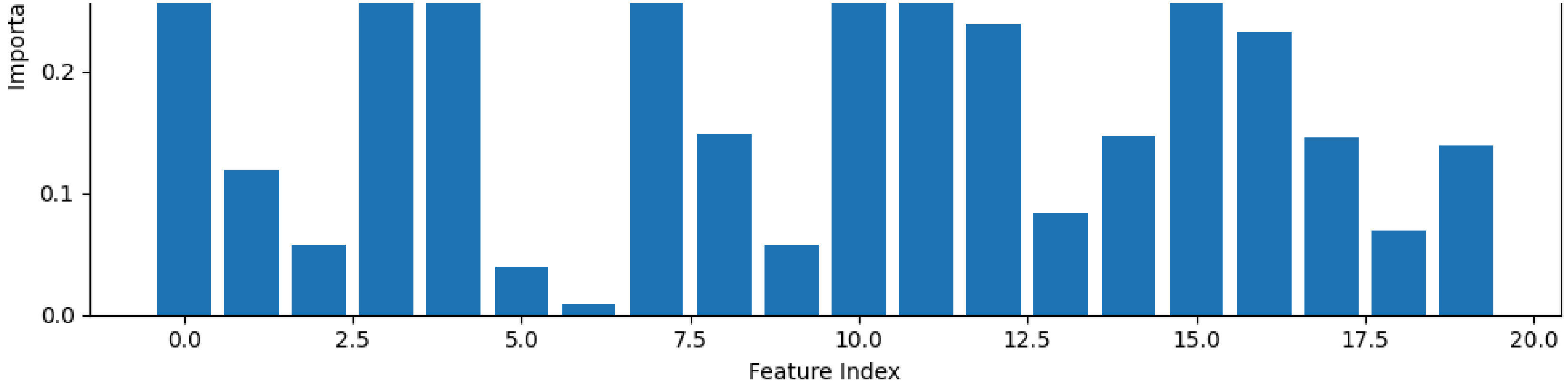

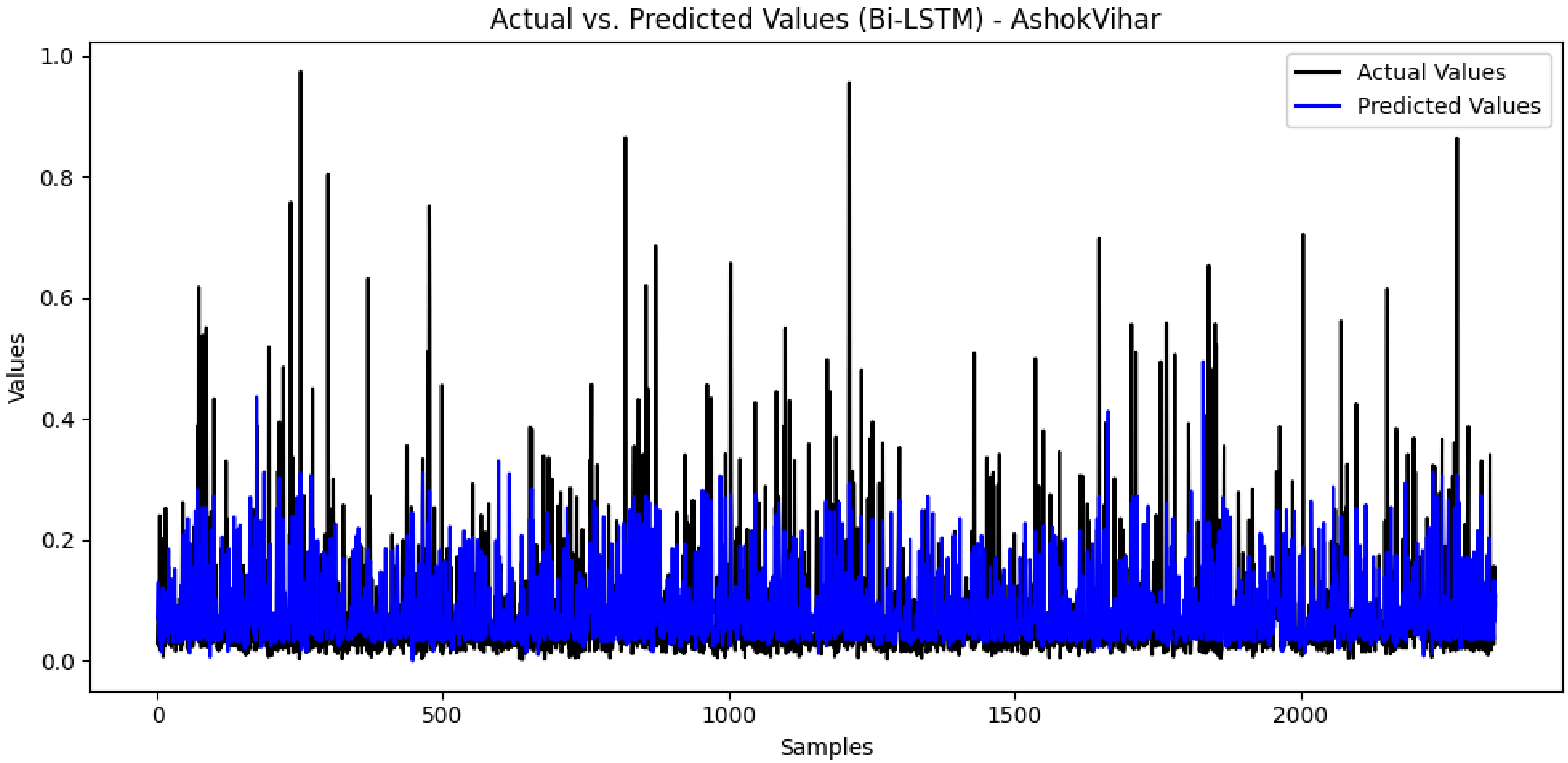


```
Processing Station: DCStadium
Epoch 1/50
/usr/local/lib/python3.11/dist-packages/keras/src/layers/rnn/rnn.py:200: UserWarning: Do not pass an `input_shape`/`input_dim` argument to a layer. When
  super().__init__(**kwargs)
147/147 ━━━━━━━━━━━━━━━━━━━━ 6s 15ms/step - loss: 0.0318 - val_loss: 0.0018
Epoch 2/50
```

```
[illegible]
147/147 ━━━━━━━━━━━━━━━━━━━━ 1s 9ms/step - loss: 0.0016 - val_loss: 0.0017
Epoch 3/50
147/147 ━━━━━━━━━━━━━━━━━━━━ 3s 9ms/step - loss: 0.0015 - val_loss: 0.0017
Epoch 4/50
147/147 ━━━━━━━━━━━━━━━━━━━━ 3s 9ms/step - loss: 0.0015 - val_loss: 0.0016
Epoch 5/50
147/147 ━━━━━━━━━━━━━━━━━━━━ 1s 9ms/step - loss: 0.0016 - val_loss: 0.0015
Epoch 6/50
147/147 ━━━━━━━━━━━━━━━━━━━━ 3s 13ms/step - loss: 0.0015 - val_loss: 0.0016
Epoch 7/50
147/147 ━━━━━━━━━━━━━━━━━━━━ 2s 9ms/step - loss: 0.0016 - val_loss: 0.0016
Epoch 8/50
147/147 ━━━━━━━━━━━━━━━━━━━━ 3s 9ms/step - loss: 0.0015 - val_loss: 0.0015
Epoch 9/50
147/147 ━━━━━━━━━━━━━━━━━━━━ 1s 9ms/step - loss: 0.0017 - val_loss: 0.0017
Epoch 10/50
147/147 ━━━━━━━━━━━━━━━━━━━━ 3s 9ms/step - loss: 0.0015 - val_loss: 0.0016
Epoch 11/50
147/147 ━━━━━━━━━━━━━━━━━━━━ 1s 9ms/step - loss: 0.0015 - val_loss: 0.0015
Epoch 12/50
147/147 ━━━━━━━━━━━━━━━━━━━━ 2s 13ms/step - loss: 0.0015 - val_loss: 0.0015
Epoch 13/50
147/147 ━━━━━━━━━━━━━━━━━━━━ 2s 12ms/step - loss: 0.0015 - val_loss: 0.0015
Epoch 14/50
147/147 ━━━━━━━━━━━━━━━━━━━━ 1s 9ms/step - loss: 0.0015 - val_loss: 0.0015
Epoch 15/50
147/147 ━━━━━━━━━━━━━━━━━━━━ 1s 9ms/step - loss: 0.0016 - val_loss: 0.0015
Epoch 16/50
147/147 ━━━━━━━━━━━━━━━━━━━━ 3s 9ms/step - loss: 0.0014 - val_loss: 0.0016
Epoch 17/50
147/147 ━━━━━━━━━━━━━━━━━━━━ 1s 9ms/step - loss: 0.0017 - val_loss: 0.0015
Epoch 18/50
147/147 ━━━━━━━━━━━━━━━━━━━━ 2s 10ms/step - loss: 0.0015 - val_loss: 0.0015
Epoch 19/50
147/147 ━━━━━━━━━━━━━━━━━━━━ 2s 10ms/step - loss: 0.0015 - val_loss: 0.0015
Epoch 20/50
147/147 ━━━━━━━━━━━━━━━━━━━━ 2s 13ms/step - loss: 0.0017 - val_loss: 0.0015
Epoch 21/50
147/147 ━━━━━━━━━━━━━━━━━━━━ 1s 9ms/step - loss: 0.0016 - val_loss: 0.0015
Epoch 22/50
147/147 ━━━━━━━━━━━━━━━━━━━━ 2s 10ms/step - loss: 0.0015 - val_loss: 0.0015
Epoch 23/50
147/147 ━━━━━━━━━━━━━━━━━━━━ 2s 9ms/step - loss: 0.0015 - val_loss: 0.0015
Epoch 24/50
147/147 ━━━━━━━━━━━━━━━━━━━━ 2s 12ms/step - loss: 0.0016 - val_loss: 0.0015
Epoch 25/50
147/147 ━━━━━━━━━━━━━━━━━━━━ 1s 9ms/step - loss: 0.0015 - val_loss: 0.0015
Epoch 26/50
147/147 ━━━━━━━━━━━━━━━━━━━━ 3s 12ms/step - loss: 0.0015 - val_loss: 0.0015
```

```
Epoch 27/50
147/147 ━━━━━━━━━━━━━━━━━━━━ 2s 11ms/step - loss: 0.0016 - val_loss: 0.0015
Epoch 28/50
147/147 ━━━━━━━━━━━━━━━━━━━━ 1s 9ms/step - loss: 0.0016 - val_loss: 0.0015
Epoch 29/50
147/147 ━━━━━━━━━━━━━━━━━━━━ 1s 9ms/step - loss: 0.0015 - val_loss: 0.0015
Epoch 30/50
147/147 ━━━━━━━━━━━━━━━━━━━━ 1s 9ms/step - loss: 0.0015 - val_loss: 0.0016
Epoch 31/50
147/147 ━━━━━━━━━━━━━━━━━━━━ 1s 9ms/step - loss: 0.0016 - val_loss: 0.0015
Epoch 32/50
147/147 ━━━━━━━━━━━━━━━━━━━━ 1s 9ms/step - loss: 0.0016 - val_loss: 0.0015
Epoch 33/50
147/147 ━━━━━━━━━━━━━━━━━━━━ 3s 9ms/step - loss: 0.0016 - val_loss: 0.0015
Epoch 34/50
147/147 ━━━━━━━━━━━━━━━━━━━━ 2s 13ms/step - loss: 0.0016 - val_loss: 0.0015
Epoch 35/50
147/147 ━━━━━━━━━━━━━━━━━━━━ 2s 10ms/step - loss: 0.0015 - val_loss: 0.0015
Epoch 36/50
147/147 ━━━━━━━━━━━━━━━━━━━━ 2s 9ms/step - loss: 0.0015 - val_loss: 0.0015
Epoch 37/50
147/147 ━━━━━━━━━━━━━━━━━━━━ 3s 9ms/step - loss: 0.0014 - val_loss: 0.0015
Epoch 38/50
147/147 ━━━━━━━━━━━━━━━━━━━━ 1s 9ms/step - loss: 0.0016 - val_loss: 0.0015
Epoch 39/50
147/147 ━━━━━━━━━━━━━━━━━━━━ 1s 9ms/step - loss: 0.0016 - val_loss: 0.0015
Epoch 40/50
147/147 ━━━━━━━━━━━━━━━━━━━━ 1s 10ms/step - loss: 0.0016 - val_loss: 0.0015
Epoch 41/50
147/147 ━━━━━━━━━━━━━━━━━━━━ 3s 10ms/step - loss: 0.0015 - val_loss: 0.0015
Epoch 42/50
147/147 ━━━━━━━━━━━━━━━━━━━━ 2s 9ms/step - loss: 0.0014 - val_loss: 0.0015
Epoch 43/50
147/147 ━━━━━━━━━━━━━━━━━━━━ 1s 9ms/step - loss: 0.0014 - val_loss: 0.0015
Epoch 44/50
147/147 ━━━━━━━━━━━━━━━━━━━━ 3s 9ms/step - loss: 0.0015 - val_loss: 0.0015
Epoch 45/50
147/147 ━━━━━━━━━━━━━━━━━━━━ 1s 9ms/step - loss: 0.0015 - val_loss: 0.0015
Epoch 46/50
147/147 ━━━━━━━━━━━━━━━━━━━━ 2s 11ms/step - loss: 0.0016 - val_loss: 0.0015
Epoch 47/50
147/147 ━━━━━━━━━━━━━━━━━━━━ 2s 14ms/step - loss: 0.0015 - val_loss: 0.0015
Epoch 48/50
147/147 ━━━━━━━━━━━━━━━━━━━━ 2s 9ms/step - loss: 0.0015 - val_loss: 0.0015
Epoch 49/50
147/147 ━━━━━━━━━━━━━━━━━━━━ 1s 9ms/step - loss: 0.0016 - val_loss: 0.0015
Epoch 50/50
147/147 ━━━━━━━━━━━━━━━━━━━━ 1s 9ms/step - loss: 0.0015 - val_loss: 0.0017
```

Training vs Validation Loss - DCStadium

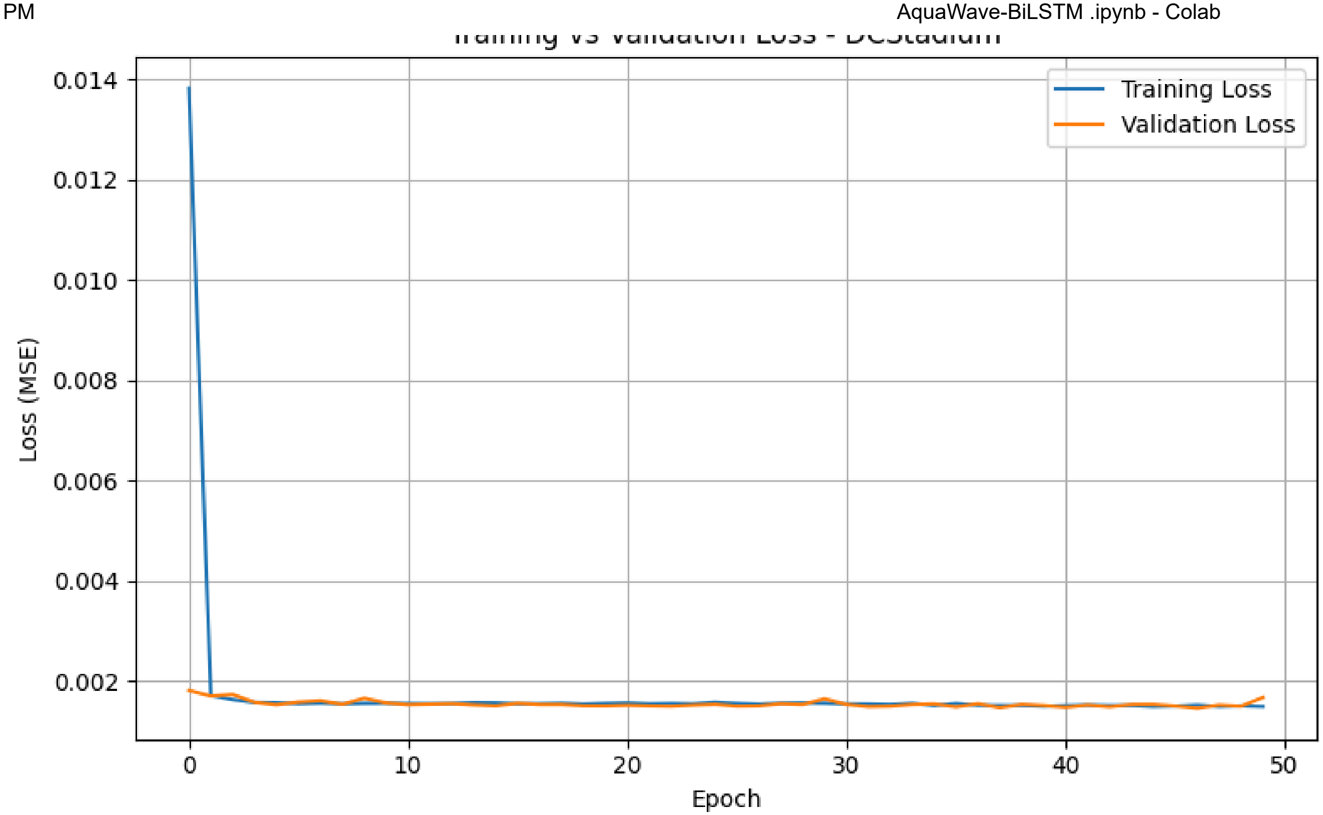


```
74/74 ━━━━━━━━━━━━━━━━━━━━ 1s 7ms/step
```

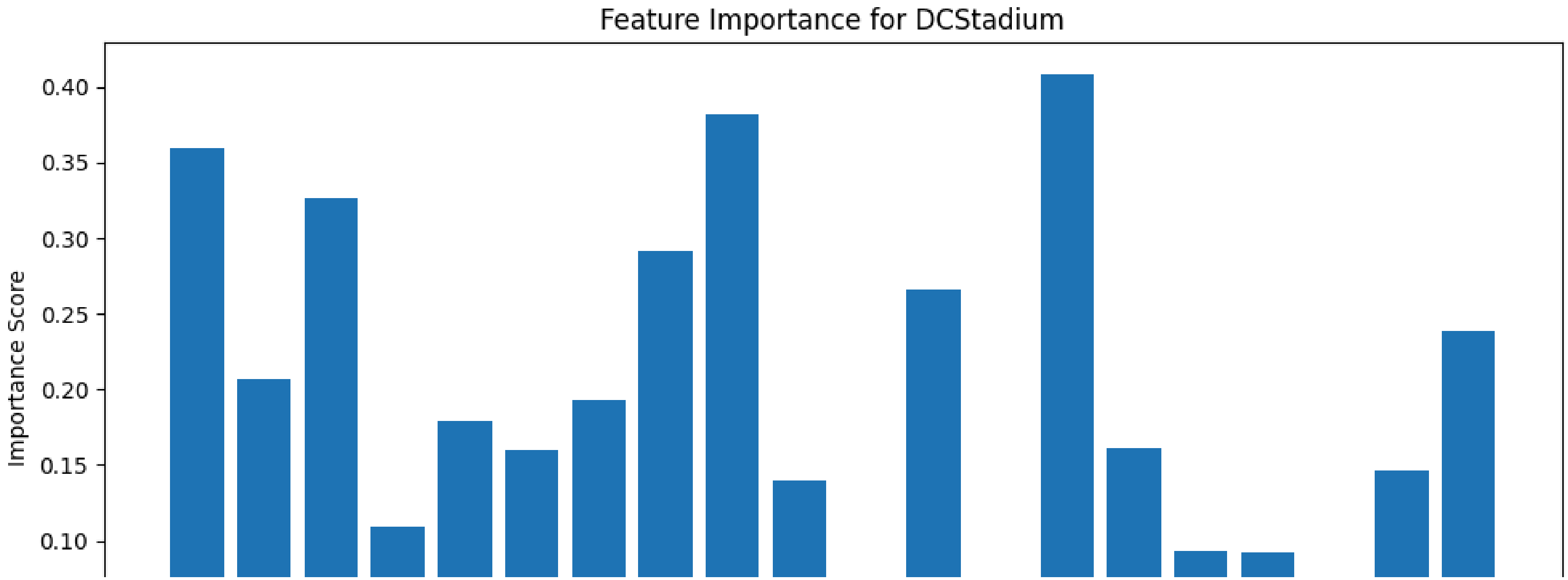

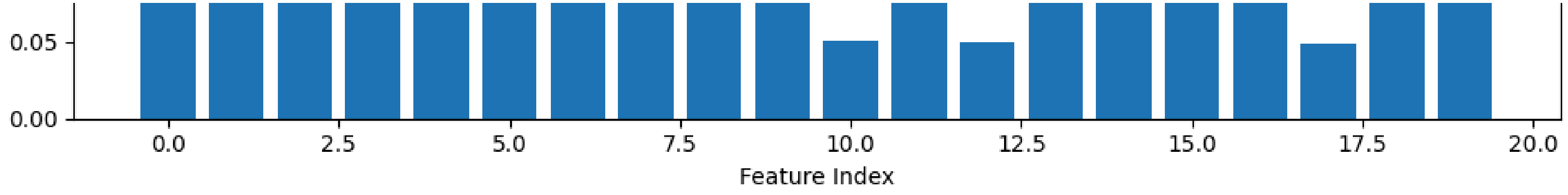

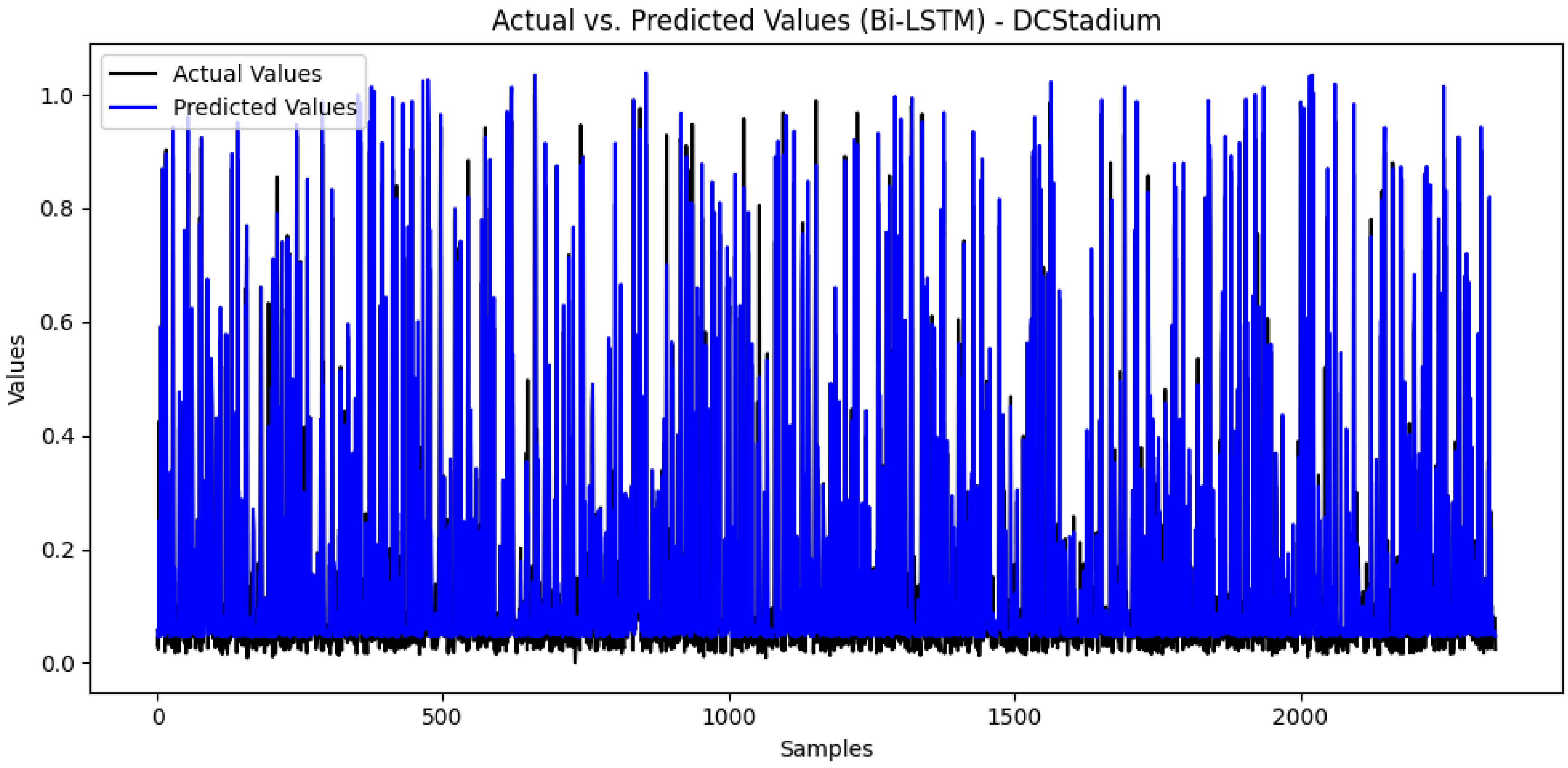


```
Processing Station: DwarkaSec8
Epoch 1/50
/usr/local/lib/python3.11/dist-packages/keras/src/layers/rnn/rnn.py:200: UserWarning: Do not pass an `input_shape`/`input_dim` argument to a layer. When
  super().__init__(**kwargs)
147/147 ━━━━━━━━━━━━━━━━━━━━ 7s 19ms/step - loss: 0.0107 - val_loss: 0.0079
Epoch 2/50
147/147 ━━━━━━━━━━━━━━━━━━━━ 2s 12ms/step - loss: 0.0066 - val_loss: 0.0059
Epoch 3/50
147/147 ━━━━━━━━━━━━━━━━━━━━ 2s 12ms/step - loss: 0.0059 - val_loss: 0.0058
Epoch 4/50
147/147 ━━━━━━━━━━━━━━━━━━━━ 2s 12ms/step - loss: 0.0055 - val_loss: 0.0050
Epoch 5/50
147/147 ━━━━━━━━━━━━━━━━━━━━ 2s 11ms/step - loss: 0.0053 - val_loss: 0.0050
```

```
Epoch 6/50
147/147 ━━━━━━━━━━━━━━━━━━━━ 2s 13ms/step - loss: 0.0047 - val_loss: 0.0047
Epoch 7/50
147/147 ━━━━━━━━━━━━━━━━━━━━ 2s 15ms/step - loss: 0.0046 - val_loss: 0.0045
Epoch 8/50
147/147 ━━━━━━━━━━━━━━━━━━━━ 2s 12ms/step - loss: 0.0041 - val_loss: 0.0032
Epoch 9/50
147/147 ━━━━━━━━━━━━━━━━━━━━ 3s 12ms/step - loss: 0.0034 - val_loss: 0.0031
Epoch 10/50
147/147 ━━━━━━━━━━━━━━━━━━━━ 3s 12ms/step - loss: 0.0030 - val_loss: 0.0031
Epoch 11/50
147/147 ━━━━━━━━━━━━━━━━━━━━ 2s 11ms/step - loss: 0.0027 - val_loss: 0.0026
Epoch 12/50
147/147 ━━━━━━━━━━━━━━━━━━━━ 2s 15ms/step - loss: 0.0024 - val_loss: 0.0026
Epoch 13/50
147/147 ━━━━━━━━━━━━━━━━━━━━ 2s 14ms/step - loss: 0.0024 - val_loss: 0.0024
Epoch 14/50
147/147 ━━━━━━━━━━━━━━━━━━━━ 2s 11ms/step - loss: 0.0021 - val_loss: 0.0020
Epoch 15/50
147/147 ━━━━━━━━━━━━━━━━━━━━ 2s 11ms/step - loss: 0.0021 - val_loss: 0.0019
Epoch 16/50
147/147 ━━━━━━━━━━━━━━━━━━━━ 2s 11ms/step - loss: 0.0017 - val_loss: 0.0014
Epoch 17/50
147/147 ━━━━━━━━━━━━━━━━━━━━ 2s 12ms/step - loss: 0.0013 - val_loss: 0.0013
Epoch 18/50
147/147 ━━━━━━━━━━━━━━━━━━━━ 2s 13ms/step - loss: 0.0011 - val_loss: 0.0011
Epoch 19/50
147/147 ━━━━━━━━━━━━━━━━━━━━ 3s 13ms/step - loss: 8.3968e-04 - val_loss: 8.1424e-04
Epoch 20/50
147/147 ━━━━━━━━━━━━━━━━━━━━ 2s 11ms/step - loss: 8.0732e-04 - val_loss: 7.1865e-04
Epoch 21/50
147/147 ━━━━━━━━━━━━━━━━━━━━ 2s 12ms/step - loss: 5.9318e-04 - val_loss: 0.0019
Epoch 22/50
147/147 ━━━━━━━━━━━━━━━━━━━━ 2s 11ms/step - loss: 0.0010 - val_loss: 5.3579e-04
Epoch 23/50
147/147 ━━━━━━━━━━━━━━━━━━━━ 3s 12ms/step - loss: 4.5563e-04 - val_loss: 5.9950e-04
Epoch 24/50
147/147 ━━━━━━━━━━━━━━━━━━━━ 2s 14ms/step - loss: 4.3004e-04 - val_loss: 3.2933e-04
Epoch 25/50
147/147 ━━━━━━━━━━━━━━━━━━━━ 2s 14ms/step - loss: 3.4334e-04 - val_loss: 3.2046e-04
Epoch 26/50
147/147 ━━━━━━━━━━━━━━━━━━━━ 2s 11ms/step - loss: 3.4251e-04 - val_loss: 3.8743e-04
Epoch 27/50
147/147 ━━━━━━━━━━━━━━━━━━━━ 2s 11ms/step - loss: 3.2042e-04 - val_loss: 2.5578e-04
Epoch 28/50
147/147 ━━━━━━━━━━━━━━━━━━━━ 2s 11ms/step - loss: 2.3515e-04 - val_loss: 2.2771e-04
Epoch 29/50
147/147 ━━━━━━━━━━━━━━━━━━━━ 3s 12ms/step - loss: 2.8000e-04 - val_loss: 2.8198e-04
Epoch 30/50
```

```
147/147 ━━━━━━━━━━━━━━━━━━━━ 3s 14ms/step - loss: 2.7022e-04 - val_loss: 2.3958e-04
Epoch 31/50
147/147 ━━━━━━━━━━━━━━━━━━━━ 2s 16ms/step - loss: 2.3116e-04 - val_loss: 3.1478e-04
Epoch 32/50
147/147 ━━━━━━━━━━━━━━━━━━━━ 2s 12ms/step - loss: 2.9977e-04 - val_loss: 2.0140e-04
Epoch 33/50
147/147 ━━━━━━━━━━━━━━━━━━━━ 2s 12ms/step - loss: 2.3452e-04 - val_loss: 2.0269e-04
Epoch 34/50
147/147 ━━━━━━━━━━━━━━━━━━━━ 3s 12ms/step - loss: 2.5843e-04 - val_loss: 2.0511e-04
Epoch 35/50
147/147 ━━━━━━━━━━━━━━━━━━━━ 3s 12ms/step - loss: 2.2216e-04 - val_loss: 1.7417e-04
Epoch 36/50
147/147 ━━━━━━━━━━━━━━━━━━━━ 3s 15ms/step - loss: 2.1980e-04 - val_loss: 2.3015e-04
Epoch 37/50
147/147 ━━━━━━━━━━━━━━━━━━━━ 2s 12ms/step - loss: 2.3685e-04 - val_loss: 1.6830e-04
Epoch 38/50
147/147 ━━━━━━━━━━━━━━━━━━━━ 2s 13ms/step - loss: 2.2259e-04 - val_loss: 1.8560e-04
Epoch 39/50
147/147 ━━━━━━━━━━━━━━━━━━━━ 2s 12ms/step - loss: 2.1133e-04 - val_loss: 1.6721e-04
Epoch 40/50
147/147 ━━━━━━━━━━━━━━━━━━━━ 2s 12ms/step - loss: 2.1871e-04 - val_loss: 1.6891e-04
Epoch 41/50
147/147 ━━━━━━━━━━━━━━━━━━━━ 3s 14ms/step - loss: 1.7157e-04 - val_loss: 4.8148e-04
Epoch 42/50
147/147 ━━━━━━━━━━━━━━━━━━━━ 3s 16ms/step - loss: 2.5052e-04 - val_loss: 1.6439e-04
Epoch 43/50
147/147 ━━━━━━━━━━━━━━━━━━━━ 2s 12ms/step - loss: 1.7884e-04 - val_loss: 3.5374e-04
Epoch 44/50
147/147 ━━━━━━━━━━━━━━━━━━━━ 2s 13ms/step - loss: 2.7446e-04 - val_loss: 1.6014e-04
Epoch 45/50
147/147 ━━━━━━━━━━━━━━━━━━━━ 2s 13ms/step - loss: 1.9898e-04 - val_loss: 1.4583e-04
Epoch 46/50
147/147 ━━━━━━━━━━━━━━━━━━━━ 2s 13ms/step - loss: 1.7505e-04 - val_loss: 1.7395e-04
Epoch 47/50
147/147 ━━━━━━━━━━━━━━━━━━━━ 3s 15ms/step - loss: 2.8102e-04 - val_loss: 1.6580e-04
Epoch 48/50
147/147 ━━━━━━━━━━━━━━━━━━━━ 2s 13ms/step - loss: 1.7712e-04 - val_loss: 1.4268e-04
Epoch 49/50
147/147 ━━━━━━━━━━━━━━━━━━━━ 2s 12ms/step - loss: 2.1436e-04 - val_loss: 1.4272e-04
Epoch 50/50
147/147 ━━━━━━━━━━━━━━━━━━━━ 3s 12ms/step - loss: 1.7874e-04 - val_loss: 1.4286e-04
```

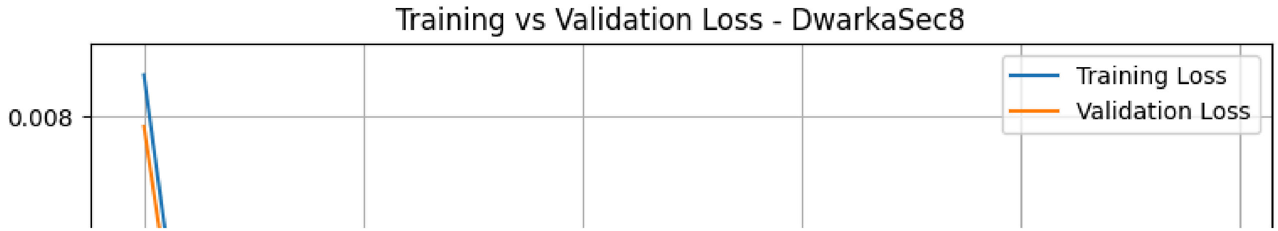

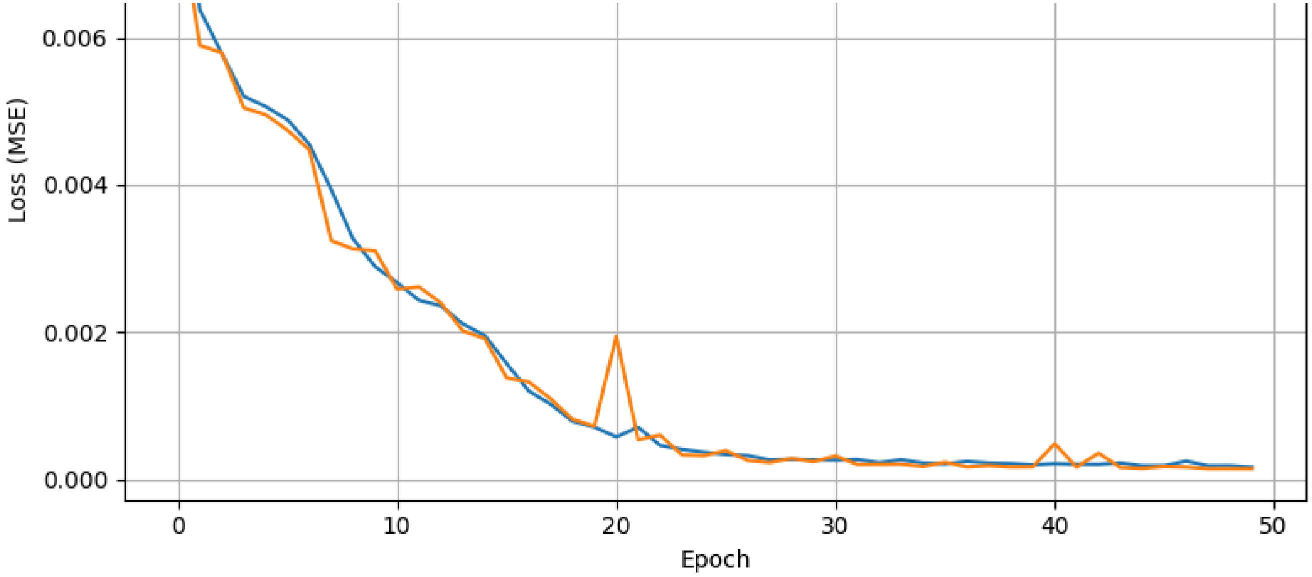


```
74/74 ━━━━━━━━━━━━━━━━━━━━ 1s 8ms/step
```

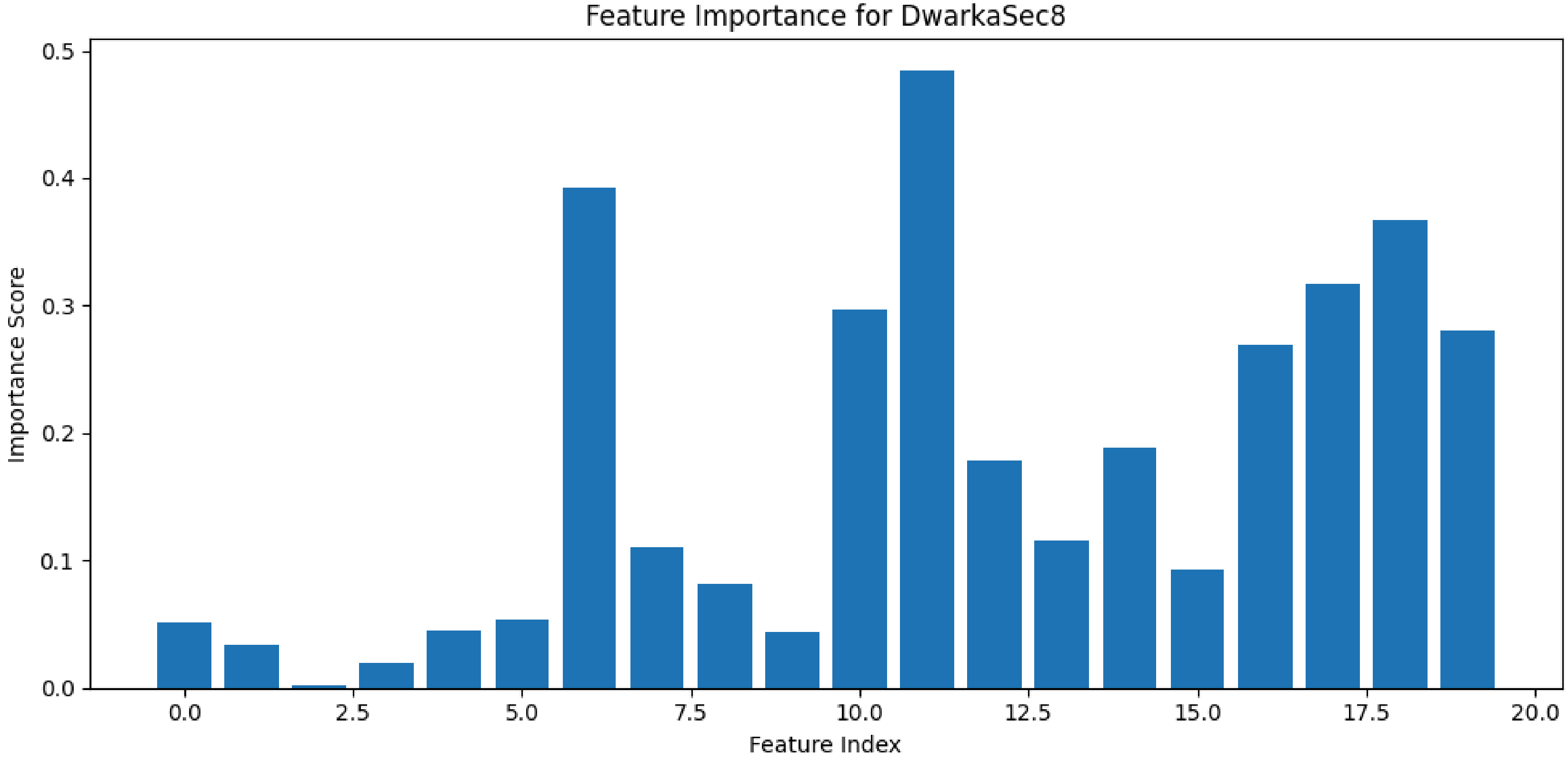

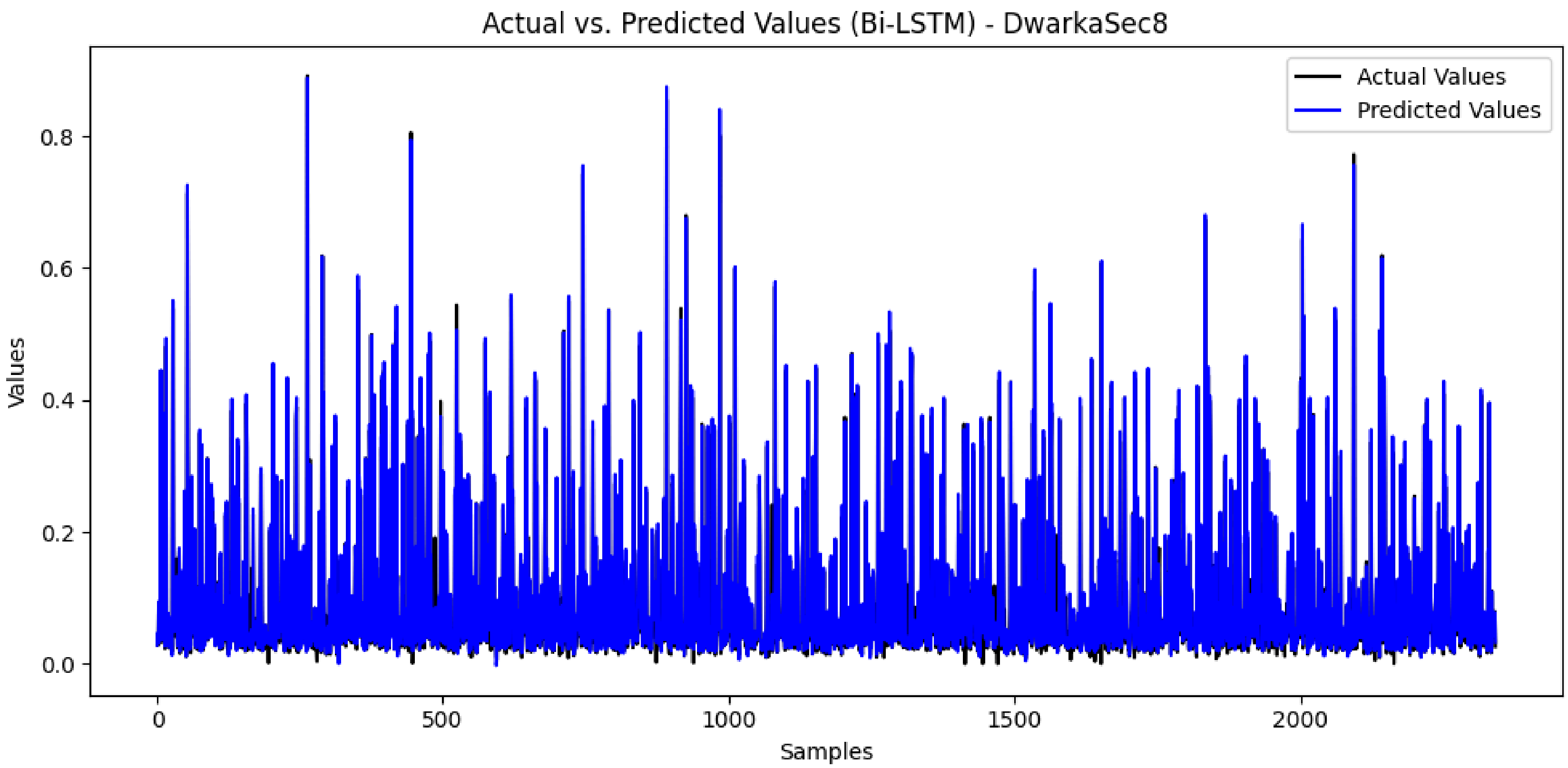


```
Processing Station: NehruNagar
Epoch 1/50
/usr/local/lib/python3.11/dist-packages/keras/src/layers/rnn/rnn.py:200: UserWarning: Do not pass an `input_shape`/`input_dim` argument to a layer. When
  super().__init__(**kwargs)
147/147 ━━━━━━━━━━━━━━━━━━━━ 7s 14ms/step - loss: 0.0342 - val_loss: 0.0306
Epoch 2/50
147/147 ━━━━━━━━━━━━━━━━━━━━ 2s 11ms/step - loss: 0.0282 - val_loss: 0.0304
Epoch 3/50
147/147 ━━━━━━━━━━━━━━━━━━━━ 2s 10ms/step - loss: 0.0302 - val_loss: 0.0298
Epoch 4/50
147/147 ━━━━━━━━━━━━━━━━━━━━ 2s 10ms/step - loss: 0.0281 - val_loss: 0.0258
Epoch 5/50
147/147 ━━━━━━━━━━━━━━━━━━━━ 2s 12ms/step - loss: 0.0227 - val_loss: 0.0248
Epoch 6/50
147/147 ━━━━━━━━━━━━━━━━━━━━ 2s 14ms/step - loss: 0.0226 - val_loss: 0.0241
Epoch 7/50
147/147 ━━━━━━━━━━━━━━━━━━━━ 2s 10ms/step - loss: 0.0225 - val_loss: 0.0242
Epoch 8/50
147/147 ━━━━━━━━━━━━━━━━━━━━ 1s 10ms/step - loss: 0.0229 - val_loss: 0.0242
Epoch 9/50
```

```
147/147 ━━━━━━━━━━━━━━━━━━━━ 1s 9ms/step - loss: 0.0228 - val_loss: 0.0240
Epoch 10/50
147/147 ━━━━━━━━━━━━━━━━━━━━ 3s 9ms/step - loss: 0.0225 - val_loss: 0.0242
Epoch 11/50
147/147 ━━━━━━━━━━━━━━━━━━━━ 1s 9ms/step - loss: 0.0227 - val_loss: 0.0248
Epoch 12/50
147/147 ━━━━━━━━━━━━━━━━━━━━ 3s 15ms/step - loss: 0.0231 - val_loss: 0.0240
Epoch 13/50
147/147 ━━━━━━━━━━━━━━━━━━━━ 2s 11ms/step - loss: 0.0227 - val_loss: 0.0243
Epoch 14/50
147/147 ━━━━━━━━━━━━━━━━━━━━ 2s 10ms/step - loss: 0.0225 - val_loss: 0.0249
Epoch 15/50
147/147 ━━━━━━━━━━━━━━━━━━━━ 2s 10ms/step - loss: 0.0225 - val_loss: 0.0242
Epoch 16/50
147/147 ━━━━━━━━━━━━━━━━━━━━ 3s 11ms/step - loss: 0.0223 - val_loss: 0.0244
Epoch 17/50
147/147 ━━━━━━━━━━━━━━━━━━━━ 3s 14ms/step - loss: 0.0223 - val_loss: 0.0241
Epoch 18/50
147/147 ━━━━━━━━━━━━━━━━━━━━ 2s 15ms/step - loss: 0.0217 - val_loss: 0.0244
Epoch 19/50
147/147 ━━━━━━━━━━━━━━━━━━━━ 2s 11ms/step - loss: 0.0226 - val_loss: 0.0246
Epoch 20/50
147/147 ━━━━━━━━━━━━━━━━━━━━ 2s 11ms/step - loss: 0.0236 - val_loss: 0.0240
Epoch 21/50
147/147 ━━━━━━━━━━━━━━━━━━━━ 3s 12ms/step - loss: 0.0223 - val_loss: 0.0241
Epoch 22/50
147/147 ━━━━━━━━━━━━━━━━━━━━ 2s 11ms/step - loss: 0.0223 - val_loss: 0.0242
Epoch 23/50
147/147 ━━━━━━━━━━━━━━━━━━━━ 3s 15ms/step - loss: 0.0225 - val_loss: 0.0241
Epoch 24/50
147/147 ━━━━━━━━━━━━━━━━━━━━ 2s 12ms/step - loss: 0.0220 - val_loss: 0.0241
Epoch 25/50
147/147 ━━━━━━━━━━━━━━━━━━━━ 2s 11ms/step - loss: 0.0219 - val_loss: 0.0241
Epoch 26/50
147/147 ━━━━━━━━━━━━━━━━━━━━ 2s 11ms/step - loss: 0.0229 - val_loss: 0.0241
Epoch 27/50
147/147 ━━━━━━━━━━━━━━━━━━━━ 3s 12ms/step - loss: 0.0214 - val_loss: 0.0243
Epoch 28/50
147/147 ━━━━━━━━━━━━━━━━━━━━ 3s 13ms/step - loss: 0.0232 - val_loss: 0.0243
Epoch 29/50
147/147 ━━━━━━━━━━━━━━━━━━━━ 3s 15ms/step - loss: 0.0218 - val_loss: 0.0248
Epoch 30/50
147/147 ━━━━━━━━━━━━━━━━━━━━ 2s 11ms/step - loss: 0.0222 - val_loss: 0.0241
Epoch 31/50
147/147 ━━━━━━━━━━━━━━━━━━━━ 2s 12ms/step - loss: 0.0216 - val_loss: 0.0242
Epoch 32/50
147/147 ━━━━━━━━━━━━━━━━━━━━ 2s 11ms/step - loss: 0.0221 - val_loss: 0.0240
Epoch 33/50
147/147 ━━━━━━━━━━━━━━━━━━━━ 2s 12ms/step - loss: 0.0223 - val_loss: 0.0242
```

```
Epoch 34/50
147/147 ━━━━━━━━━━━━━━━━━━━━ 2s 12ms/step - loss: 0.0219 - val_loss: 0.0240
Epoch 35/50
147/147 ━━━━━━━━━━━━━━━━━━━━ 2s 15ms/step - loss: 0.0219 - val_loss: 0.0242
Epoch 36/50
147/147 ━━━━━━━━━━━━━━━━━━━━ 2s 10ms/step - loss: 0.0224 - val_loss: 0.0242
Epoch 37/50
147/147 ━━━━━━━━━━━━━━━━━━━━ 3s 11ms/step - loss: 0.0221 - val_loss: 0.0243
Epoch 38/50
147/147 ━━━━━━━━━━━━━━━━━━━━ 2s 12ms/step - loss: 0.0222 - val_loss: 0.0246
Epoch 39/50
147/147 ━━━━━━━━━━━━━━━━━━━━ 3s 12ms/step - loss: 0.0227 - val_loss: 0.0240
Epoch 40/50
147/147 ━━━━━━━━━━━━━━━━━━━━ 2s 13ms/step - loss: 0.0224 - val_loss: 0.0242
Epoch 41/50
147/147 ━━━━━━━━━━━━━━━━━━━━ 2s 16ms/step - loss: 0.0217 - val_loss: 0.0240
Epoch 42/50
147/147 ━━━━━━━━━━━━━━━━━━━━ 2s 11ms/step - loss: 0.0227 - val_loss: 0.0241
Epoch 43/50
147/147 ━━━━━━━━━━━━━━━━━━━━ 3s 11ms/step - loss: 0.0220 - val_loss: 0.0240
Epoch 44/50
147/147 ━━━━━━━━━━━━━━━━━━━━ 3s 11ms/step - loss: 0.0221 - val_loss: 0.0240
Epoch 45/50
147/147 ━━━━━━━━━━━━━━━━━━━━ 2s 11ms/step - loss: 0.0220 - val_loss: 0.0241
Epoch 46/50
147/147 ━━━━━━━━━━━━━━━━━━━━ 2s 16ms/step - loss: 0.0223 - val_loss: 0.0242
Epoch 47/50
147/147 ━━━━━━━━━━━━━━━━━━━━ 2s 13ms/step - loss: 0.0220 - val_loss: 0.0239
Epoch 48/50
147/147 ━━━━━━━━━━━━━━━━━━━━ 2s 11ms/step - loss: 0.0216 - val_loss: 0.0239
Epoch 49/50
147/147 ━━━━━━━━━━━━━━━━━━━━ 2s 10ms/step - loss: 0.0221 - val_loss: 0.0240
Epoch 50/50
147/147 ━━━━━━━━━━━━━━━━━━━━ 2s 9ms/step - loss: 0.0226 - val_loss: 0.0240
```

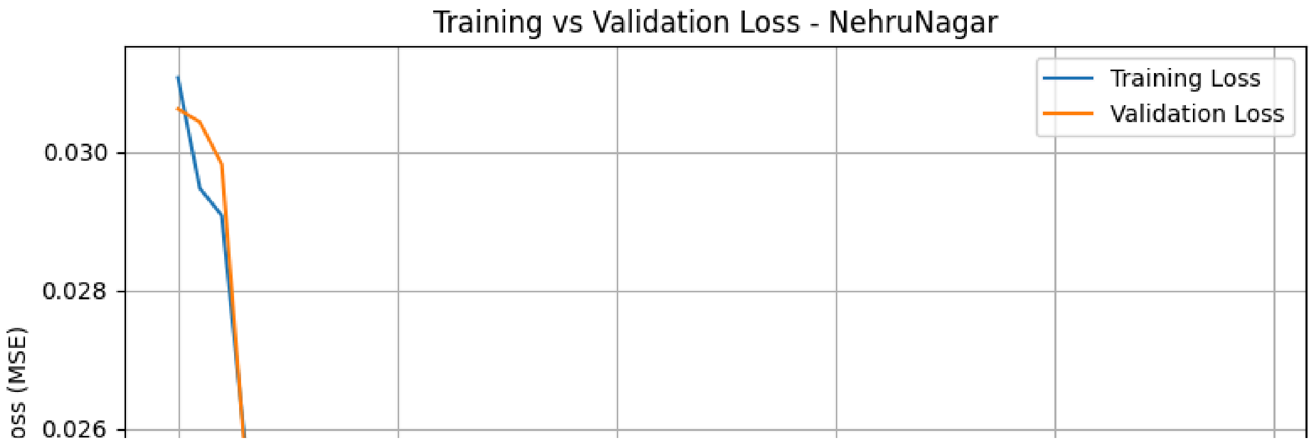

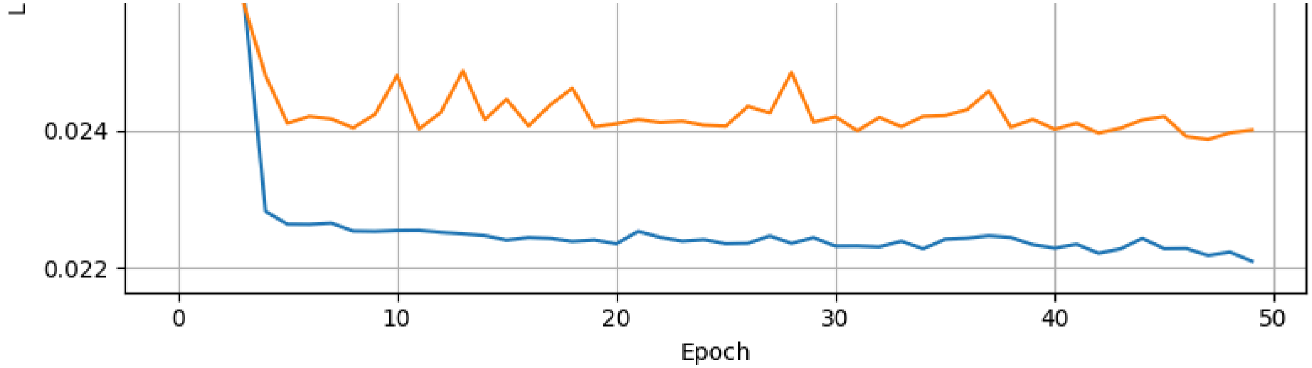


```
74/74 ━━━━━━━━━━━━━━━━━━━━ 1s 7ms/step
```

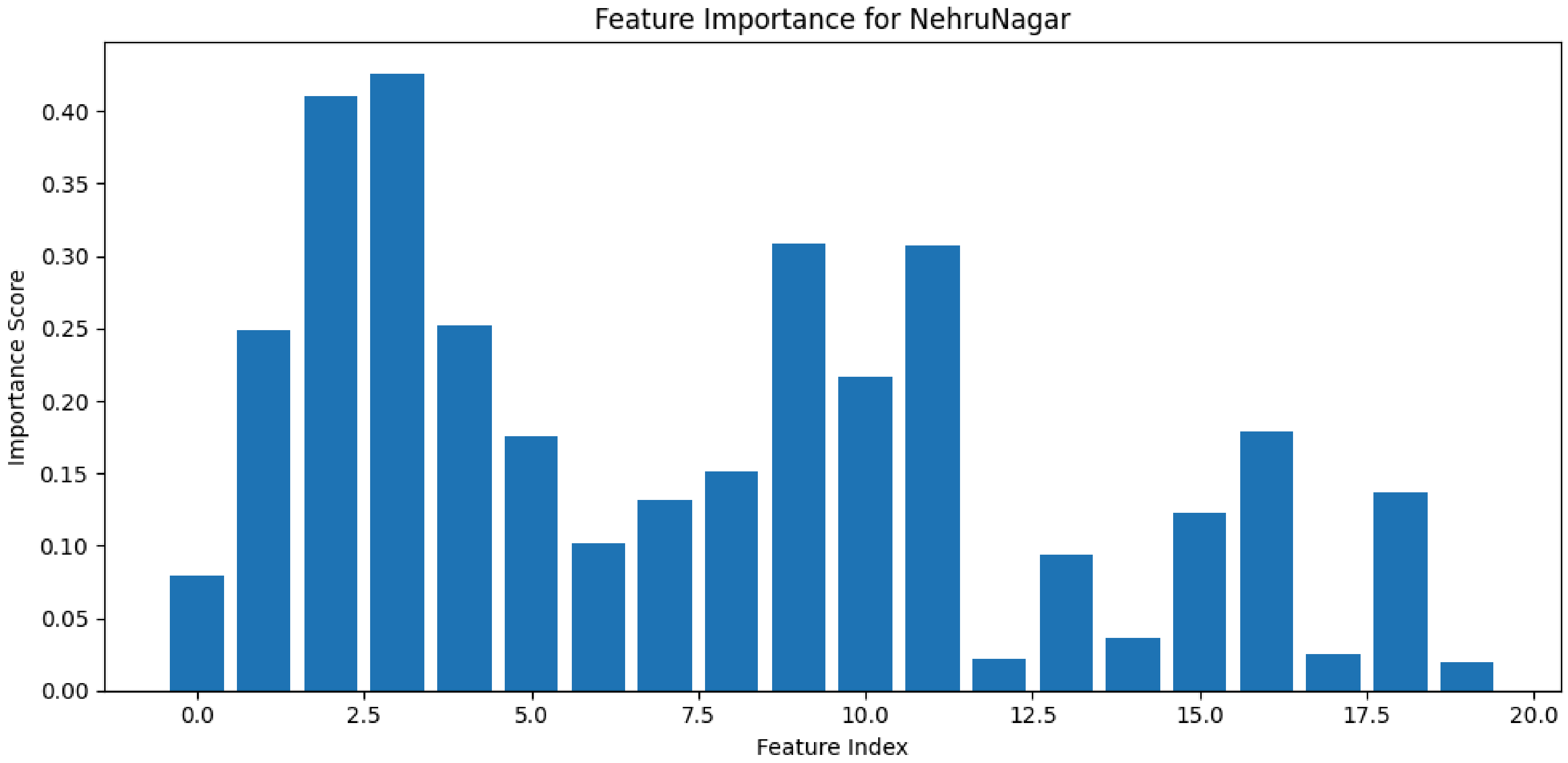

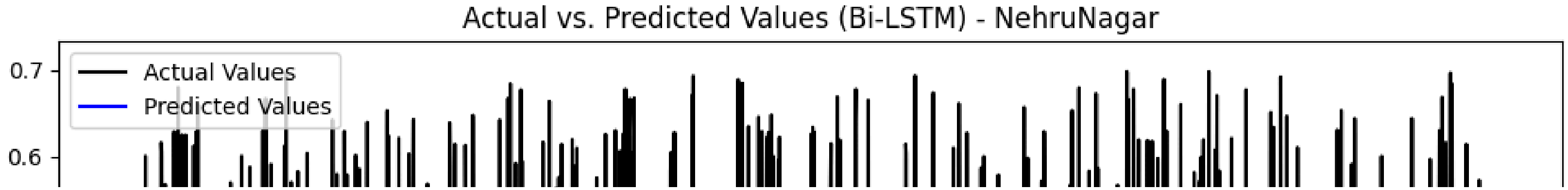

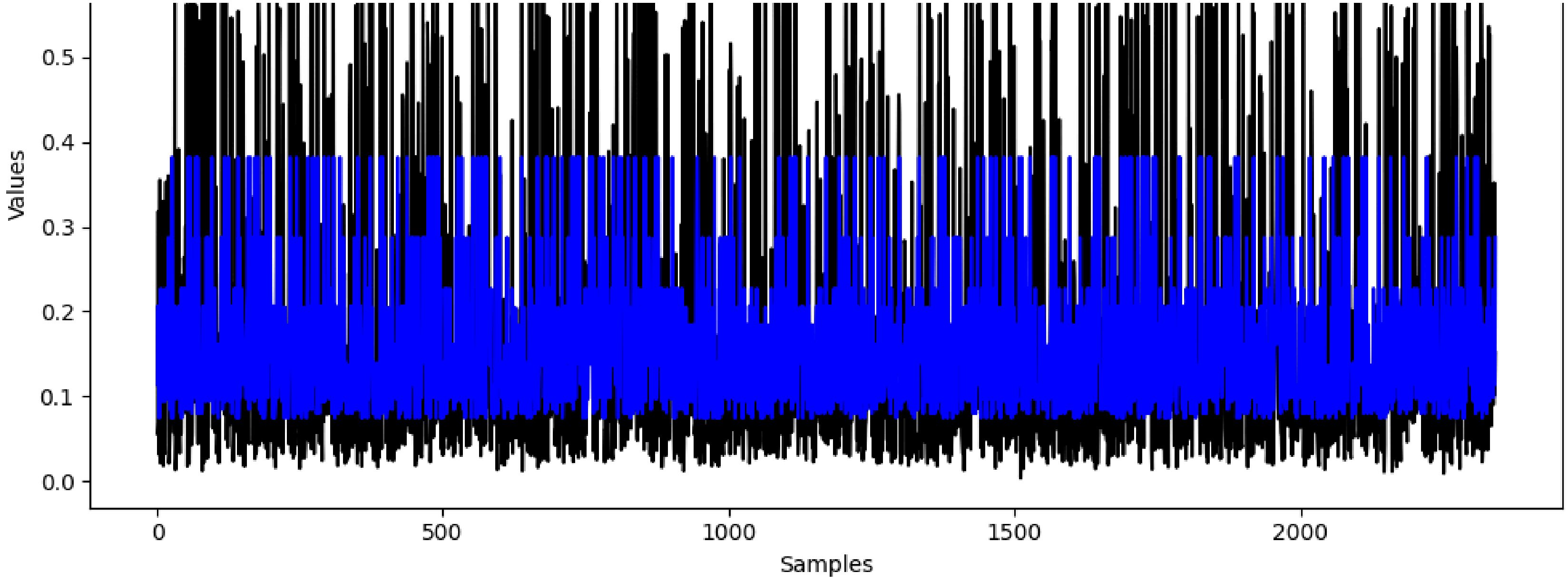


```
Processing Station: Najafgarh
Epoch 1/50
/usr/local/lib/python3.11/dist-packages/keras/src/layers/rnn/rnn.py:200: UserWarning: Do not pass an `input_shape`/`input_dim` argument to a layer. When
  super().__init__(**kwargs)
147/147 ━━━━━━━━━━━━━━━━━━━━ 8s 14ms/step - loss: 0.0040 - val_loss: 0.0032
Epoch 2/50
147/147 ━━━━━━━━━━━━━━━━━━━━ 2s 11ms/step - loss: 0.0039 - val_loss: 0.0032
Epoch 3/50
147/147 ━━━━━━━━━━━━━━━━━━━━ 2s 10ms/step - loss: 0.0036 - val_loss: 0.0033
Epoch 4/50
147/147 ━━━━━━━━━━━━━━━━━━━━ 2s 13ms/step - loss: 0.0038 - val_loss: 0.0032
Epoch 5/50
147/147 ━━━━━━━━━━━━━━━━━━━━ 2s 12ms/step - loss: 0.0041 - val_loss: 0.0032
Epoch 6/50
147/147 ━━━━━━━━━━━━━━━━━━━━ 2s 12ms/step - loss: 0.0040 - val_loss: 0.0032
Epoch 7/50
147/147 ━━━━━━━━━━━━━━━━━━━━ 3s 12ms/step - loss: 0.0038 - val_loss: 0.0032
Epoch 8/50
147/147 ━━━━━━━━━━━━━━━━━━━━ 2s 10ms/step - loss: 0.0038 - val_loss: 0.0032
Epoch 9/50
147/147 ━━━━━━━━━━━━━━━━━━━━ 2s 10ms/step - loss: 0.0038 - val_loss: 0.0033
Epoch 10/50
147/147 ━━━━━━━━━━━━━━━━━━━━ 2s 12ms/step - loss: 0.0036 - val_loss: 0.0032
Epoch 11/50
147/147 ━━━━━━━━━━━━━━━━━━━━ 2s 10ms/step - loss: 0.0036 - val_loss: 0.0032
Epoch 12/50
147/147 ━━━━━━━━━━━━━━━━━━━━ 1s 10ms/step - loss: 0.0040 - val_loss: 0.0032
```

```
Epoch 13/50
147/147 ━━━━━━━━━━━━━━━━━━━━ 1s 10ms/step - loss: 0.0036 - val_loss: 0.0033
Epoch 14/50
147/147 ━━━━━━━━━━━━━━━━━━━━ 3s 10ms/step - loss: 0.0037 - val_loss: 0.0032
Epoch 15/50
147/147 ━━━━━━━━━━━━━━━━━━━━ 3s 11ms/step - loss: 0.0033 - val_loss: 0.0033
Epoch 16/50
147/147 ━━━━━━━━━━━━━━━━━━━━ 2s 13ms/step - loss: 0.0035 - val_loss: 0.0032
Epoch 17/50
147/147 ━━━━━━━━━━━━━━━━━━━━ 2s 12ms/step - loss: 0.0039 - val_loss: 0.0033
Epoch 18/50
147/147 ━━━━━━━━━━━━━━━━━━━━ 2s 12ms/step - loss: 0.0039 - val_loss: 0.0032
Epoch 19/50
147/147 ━━━━━━━━━━━━━━━━━━━━ 2s 11ms/step - loss: 0.0039 - val_loss: 0.0032
Epoch 20/50
147/147 ━━━━━━━━━━━━━━━━━━━━ 2s 9ms/step - loss: 0.0044 - val_loss: 0.0032
Epoch 21/50
147/147 ━━━━━━━━━━━━━━━━━━━━ 1s 10ms/step - loss: 0.0036 - val_loss: 0.0032
Epoch 22/50
147/147 ━━━━━━━━━━━━━━━━━━━━ 3s 14ms/step - loss: 0.0037 - val_loss: 0.0033
Epoch 23/50
147/147 ━━━━━━━━━━━━━━━━━━━━ 2s 11ms/step - loss: 0.0032 - val_loss: 0.0033
Epoch 24/50
147/147 ━━━━━━━━━━━━━━━━━━━━ 2s 10ms/step - loss: 0.0040 - val_loss: 0.0032
Epoch 25/50
147/147 ━━━━━━━━━━━━━━━━━━━━ 1s 9ms/step - loss: 0.0039 - val_loss: 0.0032
Epoch 26/50
147/147 ━━━━━━━━━━━━━━━━━━━━ 3s 9ms/step - loss: 0.0040 - val_loss: 0.0032
Epoch 27/50
147/147 ━━━━━━━━━━━━━━━━━━━━ 3s 11ms/step - loss: 0.0038 - val_loss: 0.0032
Epoch 28/50
147/147 ━━━━━━━━━━━━━━━━━━━━ 2s 11ms/step - loss: 0.0040 - val_loss: 0.0032
Epoch 29/50
147/147 ━━━━━━━━━━━━━━━━━━━━ 2s 9ms/step - loss: 0.0041 - val_loss: 0.0032
Epoch 30/50
147/147 ━━━━━━━━━━━━━━━━━━━━ 1s 10ms/step - loss: 0.0039 - val_loss: 0.0032
Epoch 31/50
147/147 ━━━━━━━━━━━━━━━━━━━━ 3s 10ms/step - loss: 0.0034 - val_loss: 0.0032
Epoch 32/50
147/147 ━━━━━━━━━━━━━━━━━━━━ 1s 10ms/step - loss: 0.0039 - val_loss: 0.0032
Epoch 33/50
147/147 ━━━━━━━━━━━━━━━━━━━━ 2s 11ms/step - loss: 0.0041 - val_loss: 0.0032
Epoch 34/50
147/147 ━━━━━━━━━━━━━━━━━━━━ 3s 11ms/step - loss: 0.0040 - val_loss: 0.0032
Epoch 35/50
147/147 ━━━━━━━━━━━━━━━━━━━━ 2s 11ms/step - loss: 0.0030 - val_loss: 0.0032
Epoch 36/50
147/147 ━━━━━━━━━━━━━━━━━━━━ 2s 11ms/step - loss: 0.0038 - val_loss: 0.0033
Epoch 37/50
```

```
147/147 ━━━━━━━━━━━━━━━━━━━━ 2s 12ms/step - loss: 0.0037 - val_loss: 0.0032
Epoch 38/50
147/147 ━━━━━━━━━━━━━━━━━━━━ 2s 11ms/step - loss: 0.0040 - val_loss: 0.0032
Epoch 39/50
147/147 ━━━━━━━━━━━━━━━━━━━━ 3s 14ms/step - loss: 0.0034 - val_loss: 0.0032
Epoch 40/50
147/147 ━━━━━━━━━━━━━━━━━━━━ 2s 13ms/step - loss: 0.0035 - val_loss: 0.0032
Epoch 41/50
147/147 ━━━━━━━━━━━━━━━━━━━━ 2s 11ms/step - loss: 0.0036 - val_loss: 0.0032
Epoch 42/50
147/147 ━━━━━━━━━━━━━━━━━━━━ 2s 12ms/step - loss: 0.0038 - val_loss: 0.0032
Epoch 43/50
147/147 ━━━━━━━━━━━━━━━━━━━━ 2s 11ms/step - loss: 0.0039 - val_loss: 0.0032
Epoch 44/50
147/147 ━━━━━━━━━━━━━━━━━━━━ 2s 11ms/step - loss: 0.0039 - val_loss: 0.0033
Epoch 45/50
147/147 ━━━━━━━━━━━━━━━━━━━━ 2s 12ms/step - loss: 0.0038 - val_loss: 0.0032
Epoch 46/50
147/147 ━━━━━━━━━━━━━━━━━━━━ 3s 15ms/step - loss: 0.0035 - val_loss: 0.0032
Epoch 47/50
147/147 ━━━━━━━━━━━━━━━━━━━━ 2s 12ms/step - loss: 0.0041 - val_loss: 0.0032
Epoch 48/50
147/147 ━━━━━━━━━━━━━━━━━━━━ 2s 12ms/step - loss: 0.0036 - val_loss: 0.0033
Epoch 49/50
147/147 ━━━━━━━━━━━━━━━━━━━━ 3s 13ms/step - loss: 0.0034 - val_loss: 0.0032
Epoch 50/50
147/147 ━━━━━━━━━━━━━━━━━━━━ 3s 13ms/step - loss: 0.0040 - val_loss: 0.0032
```

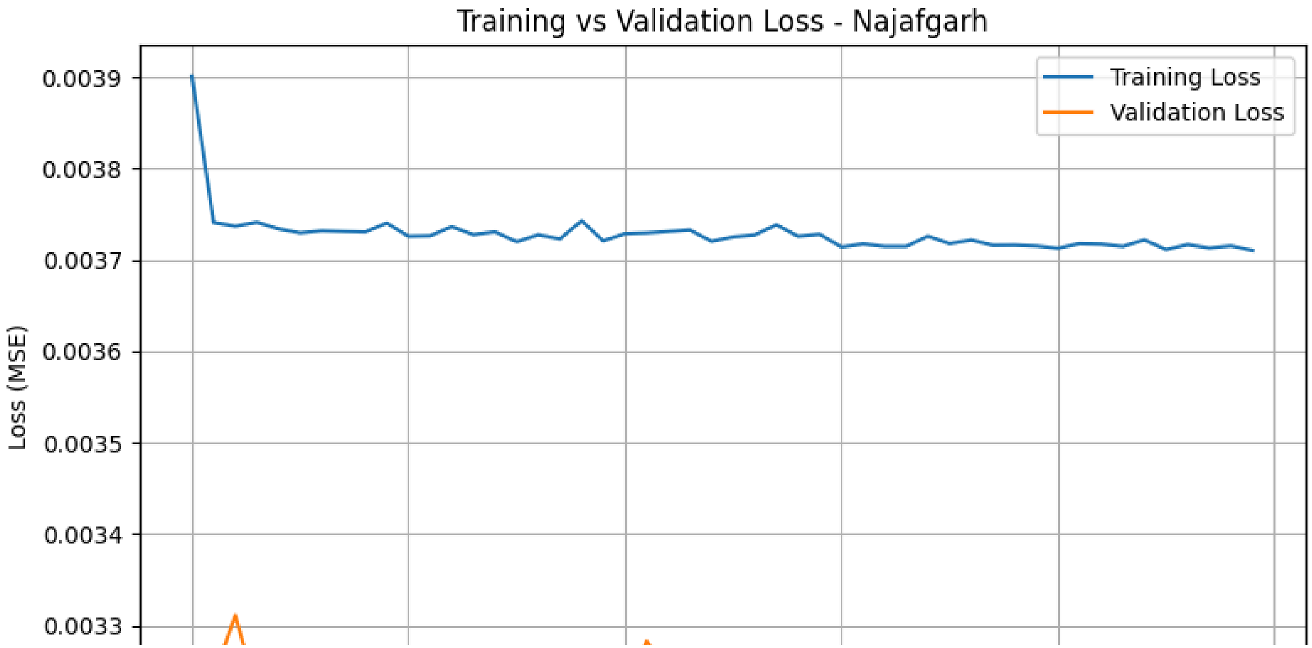

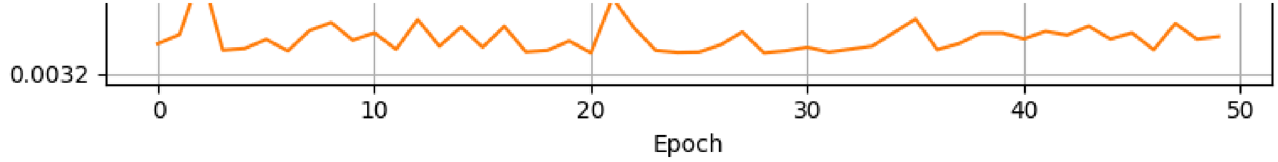


```
74/74 ━━━━━━━━━━━━━━━━━━━━ 1s 12ms/step
```

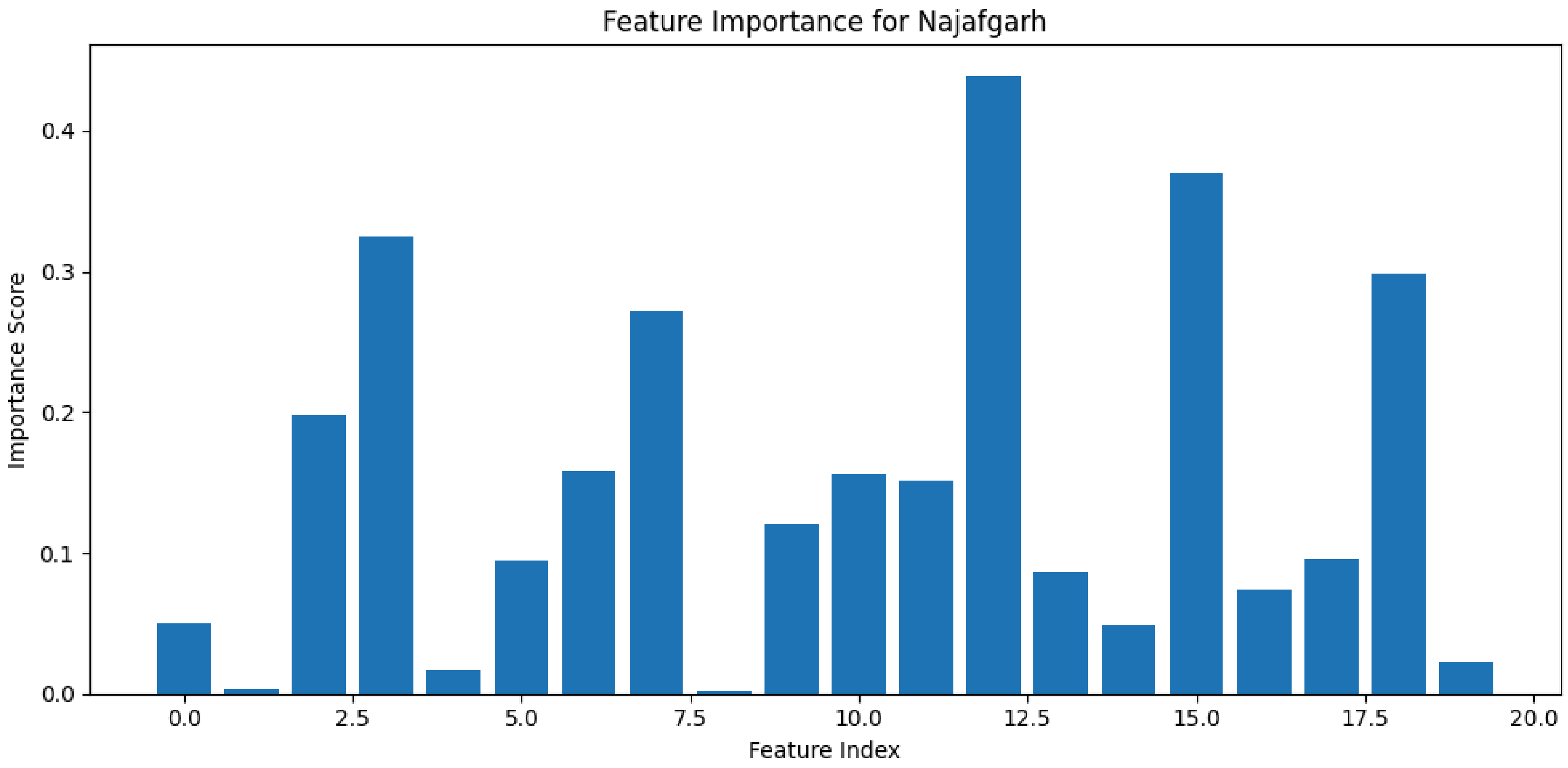

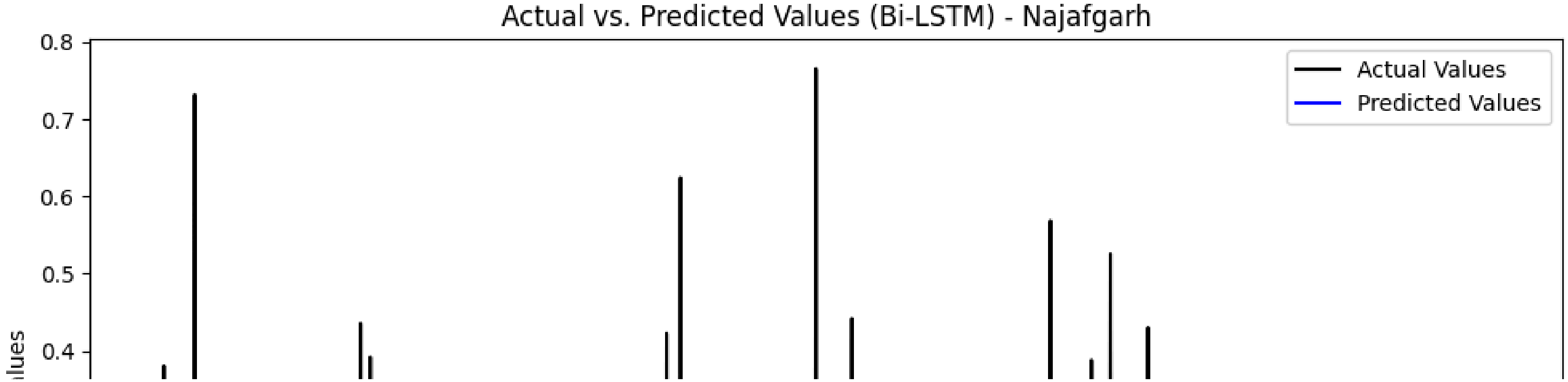

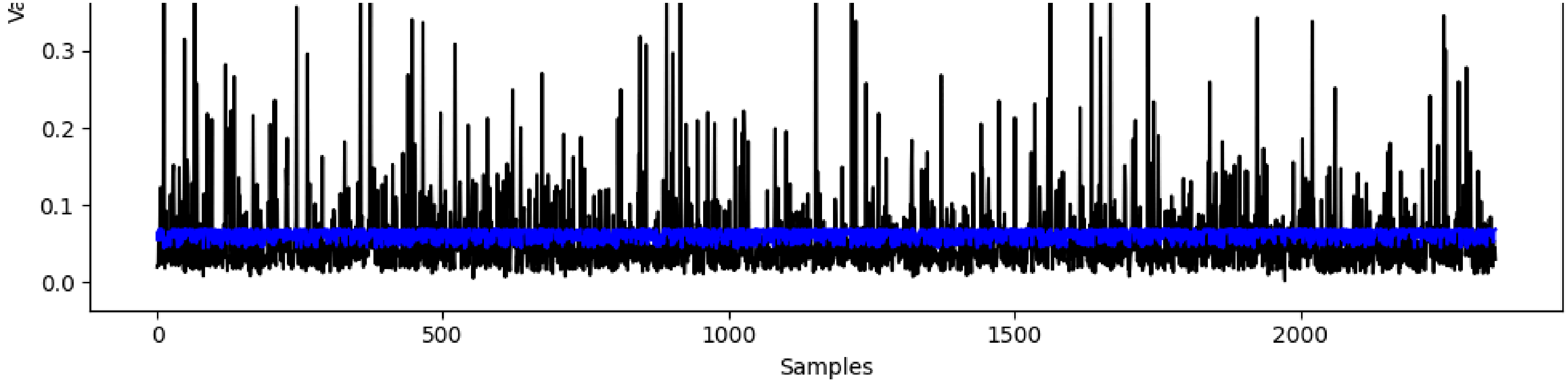


```
Processing Station: Okhla
Epoch 1/50
/usr/local/lib/python3.11/dist-packages/keras/src/layers/rnn/rnn.py:200: UserWarning: Do not pass an `input_shape`/`input_dim` argument to a layer. When
  super().__init__(**kwargs)
147/147 ━━━━━━━━━━━━━━━━━━━━ 7s 16ms/step - loss: 0.0180 - val_loss: 0.0152
Epoch 2/50
147/147 ━━━━━━━━━━━━━━━━━━━━ 2s 12ms/step - loss: 0.0153 - val_loss: 0.0126
Epoch 3/50
147/147 ━━━━━━━━━━━━━━━━━━━━ 2s 12ms/step - loss: 0.0128 - val_loss: 0.0123
Epoch 4/50
147/147 ━━━━━━━━━━━━━━━━━━━━ 3s 17ms/step - loss: 0.0123 - val_loss: 0.0123
Epoch 5/50
147/147 ━━━━━━━━━━━━━━━━━━━━ 2s 13ms/step - loss: 0.0121 - val_loss: 0.0121
Epoch 6/50
147/147 ━━━━━━━━━━━━━━━━━━━━ 2s 11ms/step - loss: 0.0140 - val_loss: 0.0119
Epoch 7/50
147/147 ━━━━━━━━━━━━━━━━━━━━ 2s 12ms/step - loss: 0.0118 - val_loss: 0.0119
Epoch 8/50
147/147 ━━━━━━━━━━━━━━━━━━━━ 3s 12ms/step - loss: 0.0122 - val_loss: 0.0126
Epoch 9/50
147/147 ━━━━━━━━━━━━━━━━━━━━ 3s 12ms/step - loss: 0.0121 - val_loss: 0.0118
Epoch 10/50
147/147 ━━━━━━━━━━━━━━━━━━━━ 2s 17ms/step - loss: 0.0125 - val_loss: 0.0118
Epoch 11/50
147/147 ━━━━━━━━━━━━━━━━━━━━ 2s 14ms/step - loss: 0.0116 - val_loss: 0.0118
Epoch 12/50
147/147 ━━━━━━━━━━━━━━━━━━━━ 2s 12ms/step - loss: 0.0113 - val_loss: 0.0121
Epoch 13/50
147/147 ━━━━━━━━━━━━━━━━━━━━ 2s 12ms/step - loss: 0.0116 - val_loss: 0.0119
Epoch 14/50
147/147 ━━━━━━━━━━━━━━━━━━━━ 2s 12ms/step - loss: 0.0114 - val_loss: 0.0119
Epoch 15/50
147/147 ━━━━━━━━━━━━━━━━━━━━ 2s 12ms/step - loss: 0.0124 - val_loss: 0.0120
Epoch 16/50
```

```
147/147 ━━━━━━━━━━━━━━━━━━━━ 3s 15ms/step - loss: 0.0115 - val_loss: 0.0118
Epoch 17/50
147/147 ━━━━━━━━━━━━━━━━━━━━ 3s 15ms/step - loss: 0.0126 - val_loss: 0.0121
Epoch 18/50
147/147 ━━━━━━━━━━━━━━━━━━━━ 2s 11ms/step - loss: 0.0123 - val_loss: 0.0124
Epoch 19/50
147/147 ━━━━━━━━━━━━━━━━━━━━ 2s 11ms/step - loss: 0.0120 - val_loss: 0.0119
Epoch 20/50
147/147 ━━━━━━━━━━━━━━━━━━━━ 3s 13ms/step - loss: 0.0119 - val_loss: 0.0120
Epoch 21/50
147/147 ━━━━━━━━━━━━━━━━━━━━ 2s 12ms/step - loss: 0.0113 - val_loss: 0.0120
Epoch 22/50
147/147 ━━━━━━━━━━━━━━━━━━━━ 2s 14ms/step - loss: 0.0118 - val_loss: 0.0121
Epoch 23/50
147/147 ━━━━━━━━━━━━━━━━━━━━ 2s 14ms/step - loss: 0.0116 - val_loss: 0.0119
Epoch 24/50
147/147 ━━━━━━━━━━━━━━━━━━━━ 1s 10ms/step - loss: 0.0113 - val_loss: 0.0118
Epoch 25/50
147/147 ━━━━━━━━━━━━━━━━━━━━ 2s 11ms/step - loss: 0.0115 - val_loss: 0.0119
Epoch 26/50
147/147 ━━━━━━━━━━━━━━━━━━━━ 3s 11ms/step - loss: 0.0120 - val_loss: 0.0121
Epoch 27/50
147/147 ━━━━━━━━━━━━━━━━━━━━ 3s 11ms/step - loss: 0.0106 - val_loss: 0.0116
Epoch 28/50
147/147 ━━━━━━━━━━━━━━━━━━━━ 3s 14ms/step - loss: 0.0102 - val_loss: 0.0114
Epoch 29/50
147/147 ━━━━━━━━━━━━━━━━━━━━ 2s 13ms/step - loss: 0.0098 - val_loss: 0.0106
Epoch 30/50
147/147 ━━━━━━━━━━━━━━━━━━━━ 2s 11ms/step - loss: 0.0098 - val_loss: 0.0100
Epoch 31/50
147/147 ━━━━━━━━━━━━━━━━━━━━ 3s 11ms/step - loss: 0.0093 - val_loss: 0.0102
Epoch 32/50
147/147 ━━━━━━━━━━━━━━━━━━━━ 3s 13ms/step - loss: 0.0091 - val_loss: 0.0099
Epoch 33/50
147/147 ━━━━━━━━━━━━━━━━━━━━ 2s 10ms/step - loss: 0.0100 - val_loss: 0.0098
Epoch 34/50
147/147 ━━━━━━━━━━━━━━━━━━━━ 3s 12ms/step - loss: 0.0087 - val_loss: 0.0095
Epoch 35/50
147/147 ━━━━━━━━━━━━━━━━━━━━ 2s 10ms/step - loss: 0.0090 - val_loss: 0.0094
Epoch 36/50
147/147 ━━━━━━━━━━━━━━━━━━━━ 1s 10ms/step - loss: 0.0094 - val_loss: 0.0101
Epoch 37/50
147/147 ━━━━━━━━━━━━━━━━━━━━ 3s 10ms/step - loss: 0.0089 - val_loss: 0.0093
Epoch 38/50
147/147 ━━━━━━━━━━━━━━━━━━━━ 3s 10ms/step - loss: 0.0084 - val_loss: 0.0093
Epoch 39/50
147/147 ━━━━━━━━━━━━━━━━━━━━ 3s 13ms/step - loss: 0.0085 - val_loss: 0.0105
Epoch 40/50
147/147 ━━━━━━━━━━━━━━━━━━━━ 2s 11ms/step - loss: 0.0090 - val_loss: 0.0095
```

```
Epoch 41/50
147/147 ━━━━━━━━━━━━━━━━━━━━ 2s 10ms/step - loss: 0.0090 - val_loss: 0.0096
Epoch 42/50
147/147 ━━━━━━━━━━━━━━━━━━━━ 1s 10ms/step - loss: 0.0084 - val_loss: 0.0096
Epoch 43/50
147/147 ━━━━━━━━━━━━━━━━━━━━ 3s 10ms/step - loss: 0.0084 - val_loss: 0.0097
Epoch 44/50
147/147 ━━━━━━━━━━━━━━━━━━━━ 3s 13ms/step - loss: 0.0085 - val_loss: 0.0092
Epoch 45/50
147/147 ━━━━━━━━━━━━━━━━━━━━ 2s 15ms/step - loss: 0.0085 - val_loss: 0.0100
Epoch 46/50
147/147 ━━━━━━━━━━━━━━━━━━━━ 2s 10ms/step - loss: 0.0087 - val_loss: 0.0098
Epoch 47/50
147/147 ━━━━━━━━━━━━━━━━━━━━ 3s 10ms/step - loss: 0.0086 - val_loss: 0.0093
Epoch 48/50
147/147 ━━━━━━━━━━━━━━━━━━━━ 3s 10ms/step - loss: 0.0082 - val_loss: 0.0093
Epoch 49/50
147/147 ━━━━━━━━━━━━━━━━━━━━ 2s 11ms/step - loss: 0.0084 - val_loss: 0.0092
Epoch 50/50
147/147 ━━━━━━━━━━━━━━━━━━━━ 2s 12ms/step - loss: 0.0084 - val_loss: 0.0093
```

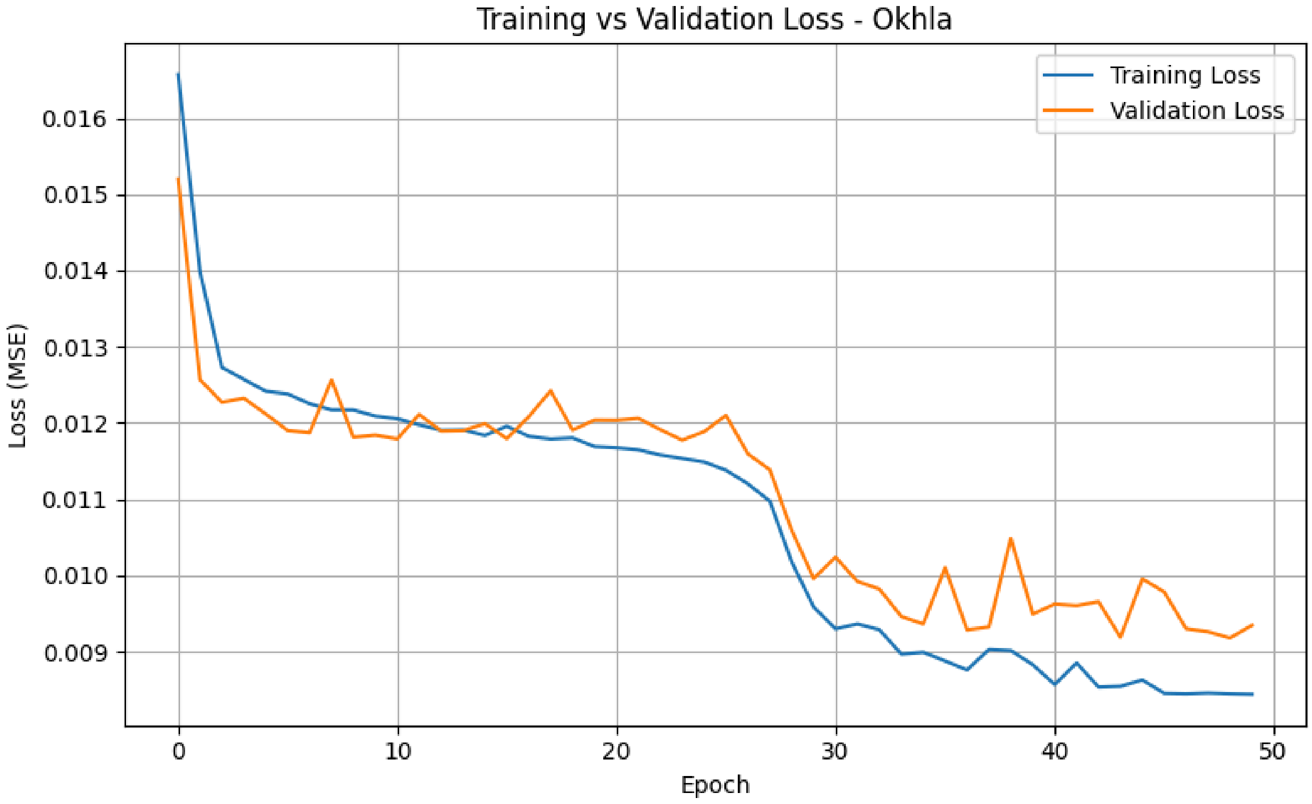


```
74/74 ━━━━━━━━━━━━━━━━━━━━ 1s 8ms/step
```

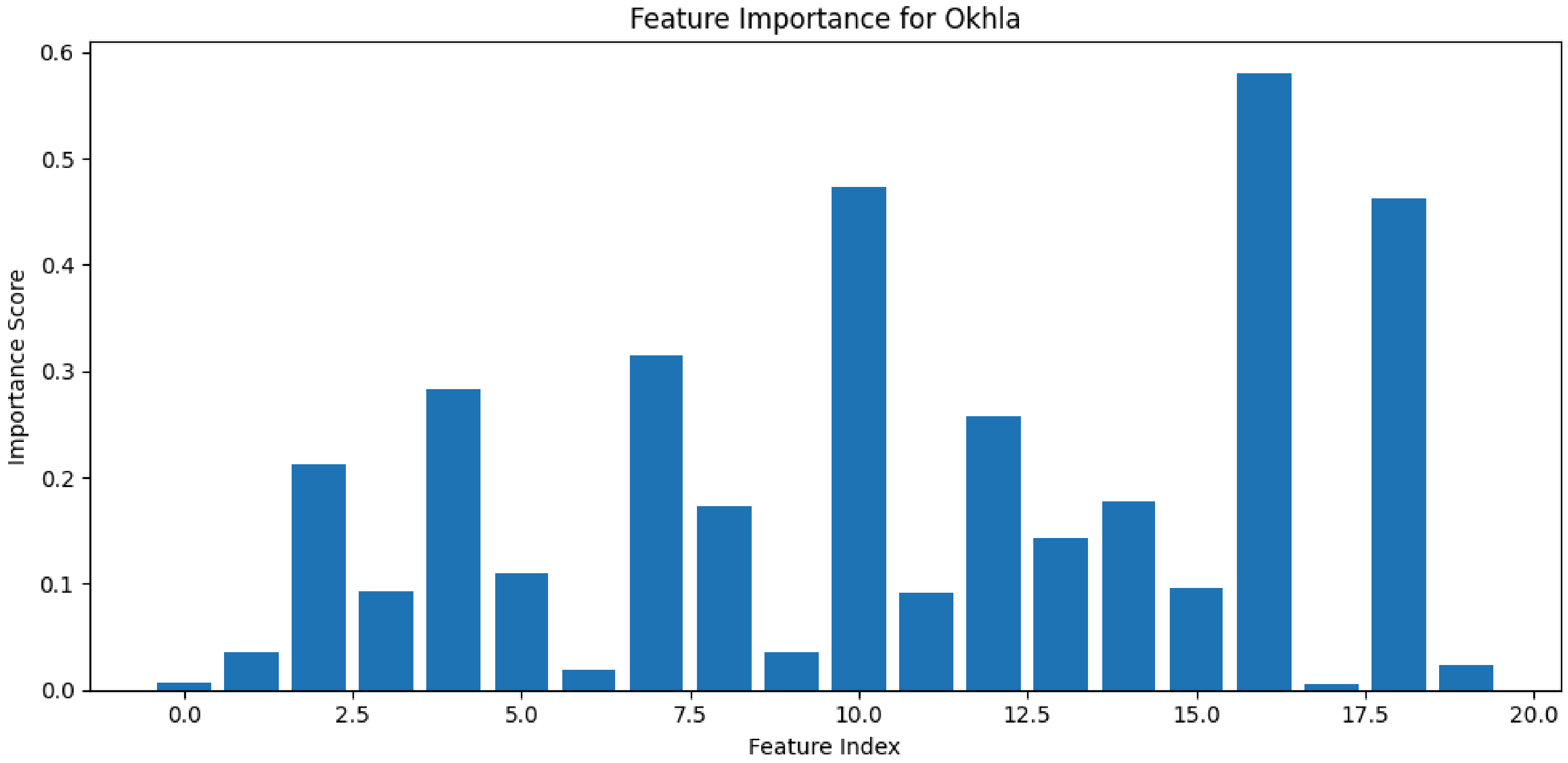
Feature Importance for Okhla
Importance Score
0.0
0.1
0.2
0.3
0.4
0.5
0.6
0.0
2.5
5.0
7.5
10.0
12.5
15.0
17.5
20.0
Feature Index

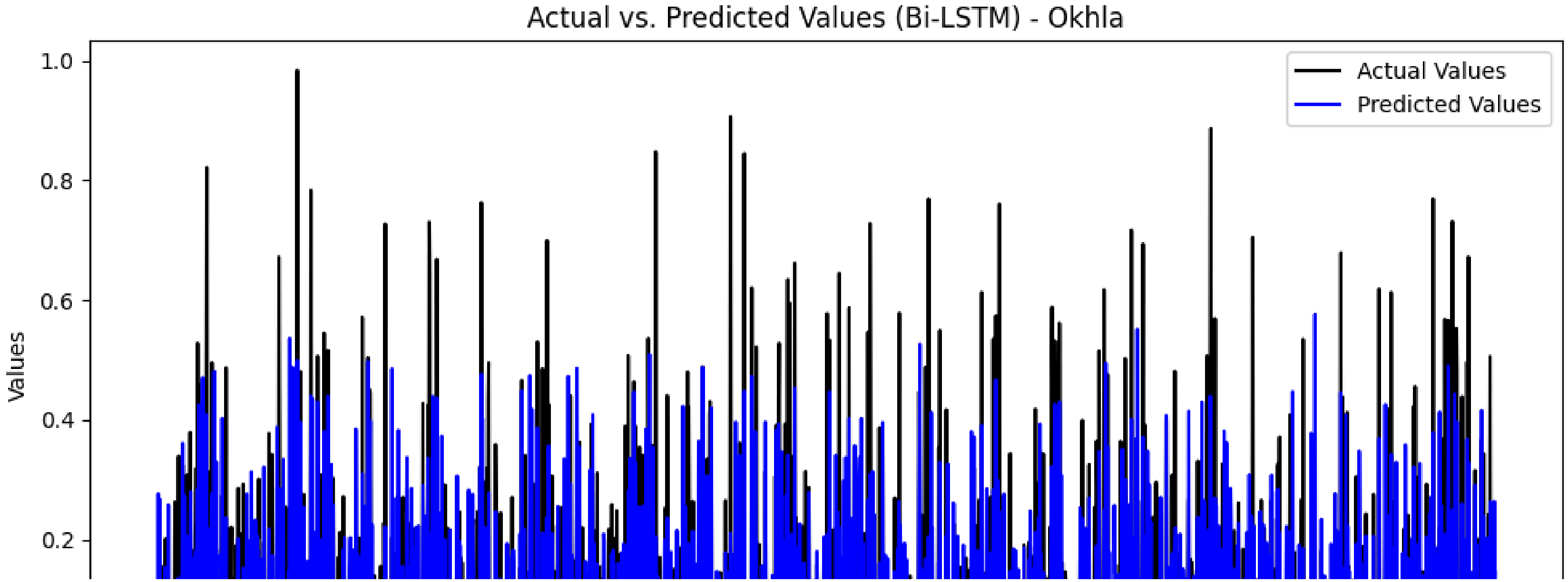
Actual vs. Predicted Values (Bi-LSTM) - Okhla
Values
0.2
0.4
0.6
0.8
1.0
Actual Values
Predicted Values

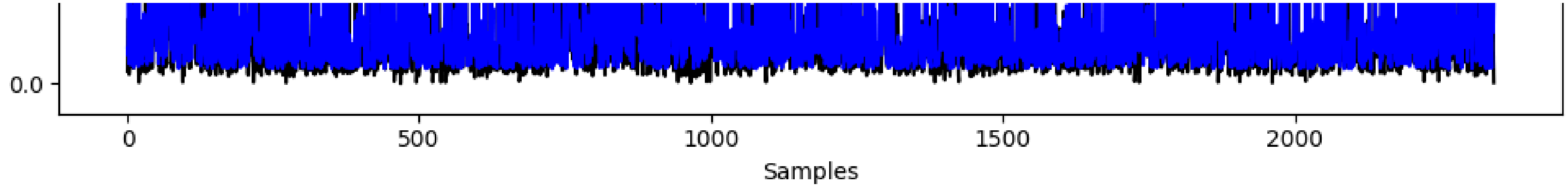


```
Final Model Evaluation Across Stations:

Station: AshokVihar
MSE: 0.0071
MAE: 0.0497
RMSE: 0.0845
R² Score: 0.3450

Station: DCStadium
MSE: 0.0017
MAE: 0.0282
RMSE: 0.0409
R² Score: 0.9672

Station: DwarkaSec8
MSE: 0.0001
MAE: 0.0073
RMSE: 0.0120
R² Score: 0.9887

Station: NehruNagar
MSE: 0.0240
MAE: 0.1116
RMSE: 0.1549
R² Score: 0.2257

Station: Najafgarh
MSE: 0.0032
MAE: 0.0354
RMSE: 0.0569
R² Score: 0.0127
```
