# Supplementary material for: Efficient multi-station air quality prediction in Delhi with wavelet and optimization-based models
Source: PLoS One. 2025 Aug 19;20(8):e0330465. doi: 10.1371/journal.pone.0330465 (PMC12364328; doi:10.1371/journal.pone.0330465)
Supplement: S1 Data — S2 File. Colab notebook for AquaWave-BiLSTM model analysis, and results. S3 File. Colab notebook containing SHAP visualizations and interpretability analysis related to PM2.5 prediction. (ZIP) [file pone.0330465.s001.zip › S3-File.pdf]

```
import numpy as np
import pandas as pd
import matplotlib.pyplot as plt
import os
import pywt
import shap

from sklearn.decomposition import PCA
from sklearn.preprocessing import StandardScaler, MinMaxScaler
from sklearn.model_selection import train_test_split
from sklearn.metrics import mean_squared_error, mean_absolute_error, r2_score
from sklearn.ensemble import RandomForestRegressor

import tensorflow as tf
from tensorflow.keras.models import Sequential
from tensorflow.keras.layers import LSTM, Dense, Bidirectional

# ============================================
# Hybrid AOAOA Optimizer
# ============================================
class HybridOptimizer:
    def __init__(self, objective_function, lower_bound, upper_bound, population_size, iterations):
        self.objective_function = objective_function
        self.lower_bound = np.array(lower_bound)
        self.upper_bound = np.array(upper_bound)
        self.population_size = population_size
        self.iterations = iterations
        self.population = np.random.uniform(low=self.lower_bound, high=self.upper_bound, size=(population_size, len(lower_bound)))
        self.best_solution = None
        self.best_fitness = float('inf')

    def optimize(self):
        for _ in range(self.iterations):
            for i in range(self.population_size):
                perturbation = np.random.uniform(-0.1, 0.1, size=self.population.shape[1])
                candidate_solution_aquila = self.population[i] + perturbation
                candidate_solution_aquila = np.clip(candidate_solution_aquila, self.lower_bound, self.upper_bound)
                fitness_aquila = self.objective_function(candidate_solution_aquila)

                if fitness_aquila < self.best_fitness:
                    self.best_fitness = fitness_aquila
                    self.best_solution = candidate_solution_aquila

            for i in range(self.population_size):
                partner_idx = np.random.randint(self.population_size)
                partner = self.population[partner_idx]
                candidate_solution_arithmetic = (self.population[i] + partner) / 2
                candidate_solution_arithmetic = np.clip(candidate_solution_arithmetic, self.lower_bound, self.upper_bound)
                fitness_arithmetic = self.objective_function(candidate_solution_arithmetic)

                if fitness_arithmetic < self.best_fitness:
                    self.best_fitness = fitness_arithmetic
                    self.best_solution = candidate_solution_arithmetic
```

```
        return self.best_solution

# ============================================
# Feature Extraction
# ============================================
def extract_wavelet_features(X, wavelet='db4', level=3, num_features=50):
    features = []
    for sample in X:
        coeffs = pywt.wavedec(sample, wavelet, level=level)
        flattened_coeffs = np.concatenate([c.flatten() for c in coeffs])
        features.append(flattened_coeffs[:num_features])
    return np.array(features)

def apply_pca(X, n_components=10):
    scaler = StandardScaler()
    X_scaled = scaler.fit_transform(X)
    pca = PCA(n_components=n_components)
    return pca.fit_transform(X_scaled)

def extract_combined_features(X):
    X_wavelet = extract_wavelet_features(X)
    X_pca = apply_pca(X_wavelet)
    return X_pca

# ============================================
# Bi-LSTM Model
# ============================================
def build_lstm_model(input_shape):
    model = Sequential([
        Bidirectional(LSTM(50, return_sequences=True, input_shape=input_shape)),
        Bidirectional(LSTM(50, return_sequences=False)),
        Dense(1)
    ])
    model.compile(optimizer='adam', loss='mean_squared_error')
    return model

# ============================================
# Training & Evaluation
# ============================================
def evaluate_model(X, y):
    if X.shape[1] == 0:
        raise ValueError("No features selected! Adjust AOAOA feature selection.")
    X_train, X_test, y_train, y_test = train_test_split(X, y, test_size=0.2, random_state=42)
    X_train = X_train.reshape(X_train.shape[0], X_train.shape[1], 1)
    X_test = X_test.reshape(X_test.shape[0], X_test.shape[1], 1)

    model = build_lstm_model((X_train.shape[1], 1))
    model.fit(X_train, y_train, epochs=50, batch_size=64, validation_data=(X_test, y_test), verbose=1)
    y_pred = model.predict(X_test)

    mse = mean_squared_error(y_test, y_pred)
    mae = mean_absolute_error(y_test, y_pred)
    rmse = np.sqrt(mse)
    r2 = r2_score(y_test, y_pred)
```

```
        return mse, mae, rmse, r2, y_test, y_pred

    # ============================================
    # Run Across All Stations
    # ============================================
    stations = {
        'AshokVihar': '/content/AshokVihar_Hourly.csv',
        'DCStadium': '/content/DCStadium_Hourly.csv',
        'DwarkaSec8': '/content/DwarkaSec8_Hourly.csv',
        'NehruNagar': '/content/NehruNagar_Hourly.csv',
        'Najafgarh': '/content/Najafgarh_Hourly.csv',
        'Okhla': '/content/Okhla_Hourly.csv'
    }

    threshold = 0.40
    results = {}
    os.makedirs("shap_plots", exist_ok=True)

    for station, file_path in stations.items():
        print(f"\nProcessing Station: {station}")

        df = pd.read_csv(file_path)

        scaler = MinMaxScaler()
        X_full = scaler.fit_transform(df.iloc[:, :-1].values)
        y = scaler.fit_transform(df.iloc[:, -1].values.reshape(-1, 1)).ravel()

        # Feature Extraction
        X_extracted = extract_combined_features(X_full)

        # Feature Selection with Hybrid AOAOA
        objective_function = lambda x: np.sum(x**2)
        hybrid_optimizer = HybridOptimizer(objective_function, lower_bound=[-1]*X_extracted.shape[1],
                                           upper_bound=[1]*X_extracted.shape[1],
                                           population_size=50, iterations=100)
        selected_features = hybrid_optimizer.optimize()

        X_selected = X_extracted[:, selected_features > threshold]
        if X_selected.shape[1] > 0:
            X_final = X_selected
            selected_feature_indices = np.where(selected_features > threshold)[0]
        else:
            X_final = X_extracted
            selected_feature_indices = np.arange(X_extracted.shape[1])

        # Dummy feature names for components (post PCA)
        selected_feature_names = [f"Component {i+1}" for i in range(X_final.shape[1])]
        print(f"Selected features ({len(selected_feature_names)}): {selected_feature_names}")

        # Final Model Evaluation
        mse, mae, rmse, r2, y_test, y_pred = evaluate_model(X_final, y)

        results[station] = {"MSE": mse, "MAE": mae, "RMSE": rmse, "R² Score": r2}

        # AOAOA Feature Importance Plot
```

```
        plt.figure(figsize=(10, 5))
        feature_importance = np.abs(selected_features)
        plt.bar(range(len(feature_importance)), feature_importance)
        plt.xlabel('Feature Index')
        plt.ylabel('Importance Score')
        plt.title(f'Feature Importance (AOAOA) - {station}')
        plt.tight_layout()
        plt.savefig(f"shap_plots/{station}_AOAOA_FeatureImportance.png")
        plt.close()

        # SHAP with RF surrogate
        print(f"Computing SHAP for {station}...")
        rf = RandomForestRegressor(n_estimators=100, random_state=42)
        rf.fit(X_final, y)

        explainer = shap.Explainer(rf, X_final)
        shap_values = explainer(X_final, check_additivity=False)

        # SHAP Summary Plot with proper labels
        plt.title(f"SHAP Feature Importance - {station}")
        shap.summary_plot(
            shap_values,
            X_final,
            feature_names=selected_feature_names,
            show=False
        )
        plt.savefig(f"shap_plots/{station}_SHAP_Summary.png", bbox_inches='tight')
        plt.close()

        # Actual vs Predicted Plot
        plt.figure(figsize=(10, 5))
        plt.plot(y_test, label="Actual", color='black')
        plt.plot(y_pred, label="Predicted", color='blue')
        plt.xlabel('Samples')
        plt.ylabel('PM2.5')
        plt.title(f'Actual vs Predicted (Bi-LSTM) - {station}')
        plt.legend()
        plt.tight_layout()
        plt.savefig(f"shap_plots/{station}_Actual_vs_Predicted.png")
        plt.close()

    # ============================================
    # Results Summary
    # ============================================
    print("\nFinal Model Evaluation Across Stations:")
    for station, metrics in results.items():
        print(f"\nStation: {station}")
        for metric, value in metrics.items():
            print(f"{metric}: {value:.4f}")
```

```
Epoch 47/50
147/147 ━━━━━━━━━━━━━━━━━━━━ 3s 17ms/step - loss: 3.3792e-04 - val_loss: 4.0325e-04
Epoch 48/50
147/147 ━━━━━━━━━━━━━━━━━━━━ 2s 17ms/step - loss: 3.2339e-04 - val_loss: 3.3191e-04
Epoch 49/50
147/147 ━━━━━━━━━━━━━━━━━━━━ 3s 21ms/step - loss: 3.1232e-04 - val_loss: 3.6534e-04
Epoch 50/50
147/147 ━━━━━━━━━━━━━━━━━━━━ 4s 17ms/step - loss: 3.1902e-04 - val_loss: 3.3090e-04
74/74 ━━━━━━━━━━━━━━━━━━━━ 1s 9ms/step
Computing SHAP for Najafgarh...
100%|===================| 11699/11704 [18:56<00:00]
Processing Station: Okhla
/usr/local/lib/python3.11/dist-packages/pywt/_multilevel.py:43: UserWarning: Level value of 3 is too high: all coefficients will experience boundary effects.
  warnings.warn(
Selected features (10): ['Component 1', 'Component 2', 'Component 3', 'Component 4', 'Component 5', 'Component 6', 'Component 7', 'Component 8', 'Component 9', 'Component 1
Epoch 1/50
/usr/local/lib/python3.11/dist-packages/keras/src/layers/rnn/rnn.py:200: UserWarning: Do not pass an `input_shape`/`input_dim` argument to a layer. When using Sequential mo
  super().__init__(**kwargs)
147/147 ━━━━━━━━━━━━━━━━━━━━ 11s 30ms/step - loss: 0.0111 - val_loss: 0.0069
Epoch 2/50
147/147 ━━━━━━━━━━━━━━━━━━━━ 3s 22ms/step - loss: 0.0062 - val_loss: 0.0037
Epoch 3/50
147/147 ━━━━━━━━━━━━━━━━━━━━ 5s 20ms/step - loss: 0.0036 - val_loss: 0.0033
Epoch 4/50
147/147 ━━━━━━━━━━━━━━━━━━━━ 4s 26ms/step - loss: 0.0027 - val_loss: 0.0022
Epoch 5/50
147/147 ━━━━━━━━━━━━━━━━━━━━ 3s 21ms/step - loss: 0.0023 - val_loss: 0.0019
Epoch 6/50
147/147 ━━━━━━━━━━━━━━━━━━━━ 3s 22ms/step - loss: 0.0019 - val_loss: 0.0016
Epoch 7/50
147/147 ━━━━━━━━━━━━━━━━━━━━ 6s 26ms/step - loss: 0.0016 - val_loss: 0.0014
Epoch 8/50
147/147 ━━━━━━━━━━━━━━━━━━━━ 3s 21ms/step - loss: 0.0016 - val_loss: 0.0014
Epoch 9/50
147/147 ━━━━━━━━━━━━━━━━━━━━ 3s 21ms/step - loss: 0.0015 - val_loss: 0.0013
Epoch 10/50
147/147 ━━━━━━━━━━━━━━━━━━━━ 6s 24ms/step - loss: 0.0013 - val_loss: 0.0011
Epoch 11/50
147/147 ━━━━━━━━━━━━━━━━━━━━ 5s 22ms/step - loss: 0.0013 - val_loss: 0.0012
Epoch 12/50
147/147 ━━━━━━━━━━━━━━━━━━━━ 6s 26ms/step - loss: 0.0011 - val_loss: 9.8708e-04
Epoch 13/50
147/147 ━━━━━━━━━━━━━━━━━━━━ 3s 22ms/step - loss: 0.0010 - val_loss: 9.6632e-04
Epoch 14/50
147/147 ━━━━━━━━━━━━━━━━━━━━ 3s 22ms/step - loss: 0.0011 - val_loss: 9.2079e-04
Epoch 15/50
147/147 ━━━━━━━━━━━━━━━━━━━━ 6s 26ms/step - loss: 8.9923e-04 - val_loss: 8.6579e-04
Epoch 16/50
147/147 ━━━━━━━━━━━━━━━━━━━━ 3s 22ms/step - loss: 9.4513e-04 - val_loss: 8.3216e-04
Epoch 17/50
147/147 ━━━━━━━━━━━━━━━━━━━━ 5s 20ms/step - loss: 8.7105e-04 - val_loss: 8.0994e-04
Epoch 18/50
147/147 ━━━━━━━━━━━━━━━━━━━━ 4s 26ms/step - loss: 8.7373e-04 - val_loss: 7.5534e-04
```

```python
import numpy as np
import pandas as pd
import matplotlib.pyplot as plt
import os
import pywt
```

```
import shap

from sklearn.decomposition import PCA
from sklearn.preprocessing import StandardScaler, MinMaxScaler
from sklearn.model_selection import train_test_split
from sklearn.metrics import mean_squared_error, mean_absolute_error, r2_score
from sklearn.ensemble import RandomForestRegressor

import tensorflow as tf
from tensorflow.keras.models import Sequential
from tensorflow.keras.layers import LSTM, Dense, Bidirectional

# ============================
# Hybrid AOAOA Optimizer
# ============================
class HybridOptimizer:
    def __init__(self, objective_function, lower_bound, upper_bound, population_size, iterations):
        self.objective_function = objective_function
        self.lower_bound = np.array(lower_bound)
        self.upper_bound = np.array(upper_bound)
        self.population_size = population_size
        self.iterations = iterations
        self.population = np.random.uniform(
            low=self.lower_bound, high=self.upper_bound,
            size=(population_size, len(lower_bound))
        )
        self.best_solution = None
        self.best_fitness = float('inf')

    def optimize(self):
        for _ in range(self.iterations):
            for i in range(self.population_size):
                perturbation = np.random.uniform(-0.1, 0.1, size=self.population.shape[1])
                candidate_solution_aquila = self.population[i] + perturbation
                candidate_solution_aquila = np.clip(candidate_solution_aquila, self.lower_bound, self.upper_bound)
                fitness_aquila = self.objective_function(candidate_solution_aquila)

                if fitness_aquila < self.best_fitness:
                    self.best_fitness = fitness_aquila
                    self.best_solution = candidate_solution_aquila

            for i in range(self.population_size):
                partner_idx = np.random.randint(self.population_size)
                partner = self.population[partner_idx]
                candidate_solution_arithmetic = (self.population[i] + partner) / 2
                candidate_solution_arithmetic = np.clip(candidate_solution_arithmetic, self.lower_bound, self.upper_bound)
                fitness_arithmetic = self.objective_function(candidate_solution_arithmetic)

                if fitness_arithmetic < self.best_fitness:
                    self.best_fitness = fitness_arithmetic
                    self.best_solution = candidate_solution_arithmetic

        return self.best_solution

# ============================
```

```
# Feature Extraction
# ============================
def extract_wavelet_features(X, wavelet='db4', level=3, num_features=50):
    features = []
    for sample in X:
        coeffs = pywt.wavedec(sample, wavelet, level=level)
        flattened_coeffs = np.concatenate([c.flatten() for c in coeffs])
        features.append(flattened_coeffs[:num_features])
    return np.array(features)

def apply_pca(X, n_components=10):
    scaler = StandardScaler()
    X_scaled = scaler.fit_transform(X)
    pca = PCA(n_components=n_components)
    return pca.fit_transform(X_scaled)

def extract_combined_features(X):
    X_wavelet = extract_wavelet_features(X)
    X_pca = apply_pca(X_wavelet)
    return X_pca

# ============================
# Bi-LSTM Model
# ============================
def build_lstm_model(input_shape):
    model = Sequential([
        Bidirectional(LSTM(50, return_sequences=True, input_shape=input_shape)),
        Bidirectional(LSTM(50, return_sequences=False)),
        Dense(1)
    ])
    model.compile(optimizer='adam', loss='mean_squared_error')
    return model

# ============================
# Training & Evaluation
# ============================
def evaluate_model(X, y):
    if X.shape[1] == 0:
        raise ValueError("No features selected!")
    X_train, X_test, y_train, y_test = train_test_split(X, y, test_size=0.2, random_state=42)
    X_train = X_train.reshape(X_train.shape[0], X_train.shape[1], 1)
    X_test = X_test.reshape(X_test.shape[0], X_test.shape[1], 1)

    model = build_lstm_model((X_train.shape[1], 1))
    model.fit(X_train, y_train, epochs=50, batch_size=64, validation_data=(X_test, y_test), verbose=1)
    y_pred = model.predict(X_test)

    mse = mean_squared_error(y_test, y_pred)
    mae = mean_absolute_error(y_test, y_pred)
    rmse = np.sqrt(mse)
    r2 = r2_score(y_test, y_pred)

    return mse, mae, rmse, r2, y_test, y_pred

# ============================
```

```
# Run only for Okhla
# ===========================

station = 'Okhla'
file_path = '/content/Okhla_Hourly.csv'

threshold = 0.40
os.makedirs("shap_plots", exist_ok=True)

print(f"\nProcessing Station: {station}")

df = pd.read_csv(file_path)

scaler = MinMaxScaler()
X_full = scaler.fit_transform(df.iloc[:, :-1].values)
y = scaler.fit_transform(df.iloc[:, -1].values.reshape(-1, 1)).ravel()

# Feature Extraction
X_extracted = extract_combined_features(X_full)

# Feature Selection with Hybrid AOAOA
objective_function = lambda x: np.sum(x**2)
hybrid_optimizer = HybridOptimizer(objective_function, lower_bound=[-1]*X_extracted.shape[1],
                                   upper_bound=[1]*X_extracted.shape[1],
                                   population_size=50, iterations=100)
selected_features = hybrid_optimizer.optimize()

X_selected = X_extracted[:, selected_features > threshold]
X_final = X_selected if X_selected.shape[1] > 0 else X_extracted

# Final Model Evaluation
mse, mae, rmse, r2, y_test, y_pred = evaluate_model(X_final, y)

print(f"\nModel Evaluation for {station}:")
print(f"MSE: {mse:.4f}")
print(f"MAE: {mae:.4f}")
print(f"RMSE: {rmse:.4f}")
print(f"R² Score: {r2:.4f}")

# AOAOA Feature Importance Plot
plt.figure(figsize=(10, 5))
feature_importance = np.abs(selected_features)
plt.bar(range(len(feature_importance)), feature_importance)
plt.xlabel('Feature Index')
plt.ylabel('Importance Score')
plt.title(f'Feature Importance (AOAOA) - {station}')
plt.tight_layout()
plt.savefig(f"shap_plots/{station}_AOAOA_FeatureImportance.png")
plt.close()

# SHAP with RF surrogate
print(f"Computing SHAP for {station}...")
rf = RandomForestRegressor(n_estimators=100, random_state=42)
rf.fit(X_final, y)
```

```
explainer = shap.Explainer(rf, X_final)
shap_values = explainer(X_final, check_additivity=False)

plt.title(f"SHAP Feature Importance - {station}")
shap.summary_plot(shap_values, X_final, show=False)
plt.savefig(f"shap_plots/{station}_SHAP_Summary.png", bbox_inches='tight')
plt.close()

# Actual vs Predicted Plot
plt.figure(figsize=(10, 5))
plt.plot(y_test, label="Actual", color='black')
plt.plot(y_pred, label="Predicted", color='blue')
plt.xlabel('Samples')
plt.ylabel('PM2.5')
plt.title(f'Actual vs Predicted (Bi-LSTM) - {station}')
plt.legend()
plt.tight_layout()
plt.savefig(f"shap_plots/{station}_Actual_vs_Predicted.png")
plt.close()

print(f"\n✅ Finished processing {station}. Results and plots are saved in 'shap_plots/' folder.")
```

```
Epoch 42/50
147/147 ━━━━━━━━━━━━━━━━━━━━ 5s 34ms/step - loss: 4.9020e-04 - val_loss: 5.8241e-04
Epoch 43/50
147/147 ━━━━━━━━━━━━━━━━━━━━ 4s 27ms/step - loss: 4.5940e-04 - val_loss: 5.7586e-04
Epoch 44/50
147/147 ━━━━━━━━━━━━━━━━━━━━ 5s 31ms/step - loss: 5.3394e-04 - val_loss: 5.7052e-04
Epoch 45/50
147/147 ━━━━━━━━━━━━━━━━━━━━ 5s 31ms/step - loss: 4.6570e-04 - val_loss: 5.8017e-04
Epoch 46/50
147/147 ━━━━━━━━━━━━━━━━━━━━ 4s 27ms/step - loss: 4.4434e-04 - val_loss: 6.1905e-04
Epoch 47/50
147/147 ━━━━━━━━━━━━━━━━━━━━ 5s 35ms/step - loss: 4.5595e-04 - val_loss: 6.4466e-04
Epoch 48/50
147/147 ━━━━━━━━━━━━━━━━━━━━ 4s 29ms/step - loss: 4.5654e-04 - val_loss: 5.2186e-04
Epoch 49/50
147/147 ━━━━━━━━━━━━━━━━━━━━ 4s 27ms/step - loss: 5.0867e-04 - val_loss: 5.4793e-04
Epoch 50/50
147/147 ━━━━━━━━━━━━━━━━━━━━ 5s 35ms/step - loss: 4.5011e-04 - val_loss: 5.0547e-04
74/74 ━━━━━━━━━━━━━━━━━━━━ 2s 15ms/step

Model Evaluation for Okhla:
MSE: 0.0005
```

Start coding or generate with AI.

```
import numpy as np
import pandas as pd
import matplotlib.pyplot as plt
from sklearn.decomposition import PCA
from sklearn.preprocessing import StandardScaler, MinMaxScaler
from sklearn.model_selection import train_test_split
from sklearn.metrics import mean_squared_error, mean_absolute_error, r2_score
import tensorflow as tf
from tensorflow.keras.models import Sequential
from tensorflow.keras.layers import LSTM, Dense, Bidirectional
import pywt

# ============================
# Hybrid AOAOA Optimizer
# ============================
class HybridOptimizer:
    def __init__(self, objective_function, lower_bound, upper_bound, population_size, iterations):
        self.objective_function = objective_function
        self.lower_bound = np.array(lower_bound)
        self.upper_bound = np.array(upper_bound)
        self.population_size = population_size
        self.iterations = iterations
        self.population = np.random.uniform(low=self.lower_bound, high=self.upper_bound, size=(population_size, len(lower_bound)))
        self.best_solution = None
        self.best_fitness = float('inf')

    def optimize(self):
        for _ in range(self.iterations):
            for i in range(self.population_size):
                perturbation = np.random.uniform(-0.1, 0.1, size=self.population.shape[1])
                candidate_solution_aquila = self.population[i] + perturbation
```

```
                candidate_solution_aquila = np.clip(candidate_solution_aquila, self.lower_bound, self.upper_bound)
                fitness_aquila = self.objective_function(candidate_solution_aquila)

                if fitness_aquila < self.best_fitness:
                    self.best_fitness = fitness_aquila
                    self.best_solution = candidate_solution_aquila

            for i in range(self.population_size):
                partner_idx = np.random.randint(self.population_size)
                partner = self.population[partner_idx]
                candidate_solution_arithmetic = (self.population[i] + partner) / 2
                candidate_solution_arithmetic = np.clip(candidate_solution_arithmetic, self.lower_bound, self.upper_bound)
                fitness_arithmetic = self.objective_function(candidate_solution_arithmetic)

                if fitness_arithmetic < self.best_fitness:
                    self.best_fitness = fitness_arithmetic
                    self.best_solution = candidate_solution_arithmetic

        return self.best_solution

# ============================
# Feature Extraction
# ============================
def extract_wavelet_features(X, wavelet='db4', level=3, num_features=50):
    features = []
    for sample in X:
        coeffs = pywt.wavedec(sample, wavelet, level=level)
        flattened_coeffs = np.concatenate([c.flatten() for c in coeffs])
        features.append(flattened_coeffs[:num_features])
    return np.array(features)

def apply_pca(X, n_components=10):
    scaler = StandardScaler()
    X_scaled = scaler.fit_transform(X)
    pca = PCA(n_components=n_components)
    return pca.fit_transform(X_scaled)

def extract_combined_features(X):
    X_wavelet = extract_wavelet_features(X)
    X_pca = apply_pca(X_wavelet)
    return X_pca

# ============================
# Bi-LSTM Model Definition
# ============================
def build_lstm_model(input_shape):
    model = Sequential([
        Bidirectional(LSTM(50, return_sequences=True, input_shape=input_shape)),
        Bidirectional(LSTM(50, return_sequences=False)),
        Dense(1)
    ])
    model.compile(optimizer='adam', loss='mean_squared_error')
    return model

# ============================
```

```
# Training & Evaluation
# ============================
def evaluate_model(X, y):
    if X.shape[1] == 0:
        raise ValueError("No features selected! Adjust AOAOA feature selection.")
    X_train, X_test, y_train, y_test = train_test_split(X, y, test_size=0.2, random_state=42)
    X_train = X_train.reshape(X_train.shape[0], X_train.shape[1], 1)
    X_test = X_test.reshape(X_test.shape[0], X_test.shape[1], 1)

    model = build_lstm_model((X_train.shape[1], 1))
    model.fit(X_train, y_train, epochs=50, batch_size=64, validation_data=(X_test, y_test), verbose=1)
    y_pred = model.predict(X_test)

    mse = mean_squared_error(y_test, y_pred)
    mae = mean_absolute_error(y_test, y_pred)
    rmse = np.sqrt(mse)
    r2 = r2_score(y_test, y_pred)

    return mse, mae, rmse, r2, y_test, y_pred

# ============================
# Multi-Station Processing
# ============================
stations = {
    'AshokVihar': '/content/AshokVihar_Hourly.csv',
    'DCStadium': '/content/DCStadium_Hourly.csv',
    'DwarkaSec8': '/content/DwarkaSec8_Hourly.csv',
    'NehruNagar': '/content/NehruNagar_Hourly.csv',
    'Najafgarh': '/content/Najafgarh_Hourly.csv',
    'Okhla': '/content/Okhla_Hourly.csv'
}

threshold = 0.40  # Adjust this value as needed
results = {}

for station, file_path in stations.items():
    print(f"\nProcessing Station: {station}")

    # Load Data
    df = pd.read_csv(file_path)

    # Preprocessing
    scaler = MinMaxScaler()
    X_full = scaler.fit_transform(df.iloc[:, :-1].values)
    y = scaler.fit_transform(df.iloc[:, -1].values.reshape(-1, 1))

    # Feature Extraction
    X_extracted = extract_combined_features(X_full)

    # Feature Selection with Hybrid AOAOA
    objective_function = lambda x: np.sum(x**2)
    hybrid_optimizer = HybridOptimizer(objective_function, lower_bound=[-1] * X_extracted.shape[1],
                                       upper_bound=[1] * X_extracted.shape[1],
                                       population_size=50, iterations=100)
    selected_features = hybrid_optimizer.optimize()
```

```
        # Select features above threshold
        X_selected = X_extracted[:, selected_features > threshold]

        # Final Model Evaluation
        X_final = X_selected if X_selected.shape[1] > 0 else X_extracted
        mse, mae, rmse, r2, y_test, y_pred = evaluate_model(X_final, y)

        # Store results
        results[station] = {"MSE": mse, "MAE": mae, "RMSE": rmse, "R² Score": r2}

        # Plot Feature Importance
        plt.figure(figsize=(10, 5))
        feature_importance = np.abs(selected_features)
        plt.bar(range(len(feature_importance)), feature_importance)
        plt.xlabel('Feature Index')
        plt.ylabel('Importance Score')
        plt.title(f'Feature Importance for {station}')
        plt.show()
    # Plot Actual vs Predicted
        plt.figure(figsize=(10,5), dpi=300)
        plt.plot(y_test, label="Actual", color='black')
        plt.plot(y_pred, label="Predicted", color='blue')
        plt.xlabel("Samples", fontsize=12, fontweight='bold')
        plt.ylabel("Target", fontsize=12, fontweight='bold')
        plt.title(f"Actual vs Predicted Values (AquaWave-BiLSTM) – {station}", fontsize=14, fontweight='bold')
        plt.legend()
        plt.tight_layout()
        plt.savefig(f"AOAOA_FeatureImportance_Plots/{station}_Actual_vs_Predicted.png", bbox_inches='tight')
        plt.show()


    # Print Final Results
    print("\nFinal Model Evaluation Across Stations:")
    for station, metrics in results.items():
        print(f"\nStation: {station}")
        for metric, value in metrics.items():
            print(f"{metric}: {value:.4f}")
```

```
Processing Station: AshokVihar
/usr/local/lib/python3.11/dist-packages/pywt/_multilevel.py:43: UserWarning: Level value of 3 is too high: all coefficients will experience boundary effects.
  warnings.warn(
Epoch 1/50
/usr/local/lib/python3.11/dist-packages/keras/src/layers/rnn/rnn.py:200: UserWarning: Do not pass an `input_shape`/`input_dim` argument to a layer. When using Sequential mod
  super().__init__(**kwargs)
147/147 ━━━━━━━━━━━━━━━━━━━━ 6s 14ms/step - loss: 0.0060 - val_loss: 0.0029
Epoch 2/50
147/147 ━━━━━━━━━━━━━━━━━━━━ 2s 10ms/step - loss: 0.0028 - val_loss: 0.0022
Epoch 3/50
147/147 ━━━━━━━━━━━━━━━━━━━━ 1s 10ms/step - loss: 0.0022 - val_loss: 0.0019
Epoch 4/50
147/147 ━━━━━━━━━━━━━━━━━━━━ 3s 11ms/step - loss: 0.0018 - val_loss: 0.0017
Epoch 5/50
147/147 ━━━━━━━━━━━━━━━━━━━━ 2s 10ms/step - loss: 0.0016 - val_loss: 0.0016
Epoch 6/50
147/147 ━━━━━━━━━━━━━━━━━━━━ 3s 10ms/step - loss: 0.0015 - val_loss: 0.0015
Epoch 7/50
147/147 ━━━━━━━━━━━━━━━━━━━━ 2s 11ms/step - loss: 0.0014 - val_loss: 0.0014
Epoch 8/50
147/147 ━━━━━━━━━━━━━━━━━━━━ 2s 10ms/step - loss: 0.0012 - val_loss: 0.0013
Epoch 9/50
147/147 ━━━━━━━━━━━━━━━━━━━━ 3s 11ms/step - loss: 0.0012 - val_loss: 0.0013
Epoch 10/50
147/147 ━━━━━━━━━━━━━━━━━━━━ 2s 10ms/step - loss: 0.0014 - val_loss: 0.0012
Epoch 11/50
147/147 ━━━━━━━━━━━━━━━━━━━━ 1s 10ms/step - loss: 0.0013 - val_loss: 0.0012
Epoch 12/50
147/147 ━━━━━━━━━━━━━━━━━━━━ 3s 10ms/step - loss: 0.0011 - val_loss: 0.0014
Epoch 13/50
147/147 ━━━━━━━━━━━━━━━━━━━━ 2s 11ms/step - loss: 0.0011 - val_loss: 0.0011
Epoch 14/50
147/147 ━━━━━━━━━━━━━━━━━━━━ 3s 15ms/step - loss: 0.0010 - val_loss: 0.0012
Epoch 15/50
147/147 ━━━━━━━━━━━━━━━━━━━━ 2s 11ms/step - loss: 0.0011 - val_loss: 0.0010
Epoch 16/50
147/147 ━━━━━━━━━━━━━━━━━━━━ 1s 10ms/step - loss: 0.0010 - val_loss: 9.6526e-04
Epoch 17/50
147/147 ━━━━━━━━━━━━━━━━━━━━ 1s 10ms/step - loss: 9.5687e-04 - val_loss: 9.3281e-04
Epoch 18/50
147/147 ━━━━━━━━━━━━━━━━━━━━ 3s 10ms/step - loss: 9.0310e-04 - val_loss: 9.1952e-04
Epoch 19/50
147/147 ━━━━━━━━━━━━━━━━━━━━ 1s 10ms/step - loss: 9.5236e-04 - val_loss: 9.6126e-04
Epoch 20/50
147/147 ━━━━━━━━━━━━━━━━━━━━ 5s 24ms/step - loss: 9.2343e-04 - val_loss: 8.8346e-04
Epoch 21/50
147/147 ━━━━━━━━━━━━━━━━━━━━ 3s 11ms/step - loss: 8.8864e-04 - val_loss: 8.9902e-04
Epoch 22/50
147/147 ━━━━━━━━━━━━━━━━━━━━ 2s 11ms/step - loss: 8.3975e-04 - val_loss: 8.6102e-04
Epoch 23/50
147/147 ━━━━━━━━━━━━━━━━━━━━ 1s 10ms/step - loss: 8.2916e-04 - val_loss: 8.4827e-04
Epoch 24/50
147/147 ━━━━━━━━━━━━━━━━━━━━ 3s 11ms/step - loss: 7.5349e-04 - val_loss: 8.6975e-04
Epoch 25/50
147/147 ━━━━━━━━━━━━━━━━━━━━ 3s 15ms/step - loss: 7.9103e-04 - val_loss: 8.9787e-04
Epoch 26/50
147/147 ━━━━━━━━━━━━━━━━━━━━ 2s 10ms/step - loss: 7.1480e-04 - val_loss: 8.0116e-04
Epoch 27/50
147/147 ━━━━━━━━━━━━━━━━━━━━ 3s 10ms/step - loss: 7.1564e-04 - val_loss: 7.6288e-04
[illegible]
```

```
Epoch 28/50
147/147 ━━━━━━━━━━━━━━━━━━━━ 1s 10ms/step - loss: 6.8315e-04 - val_loss: 7.6569e-04
Epoch 29/50
147/147 ━━━━━━━━━━━━━━━━━━━━ 2s 11ms/step - loss: 6.5697e-04 - val_loss: 7.2552e-04
Epoch 30/50
147/147 ━━━━━━━━━━━━━━━━━━━━ 3s 12ms/step - loss: 6.4902e-04 - val_loss: 8.0438e-04
Epoch 31/50
147/147 ━━━━━━━━━━━━━━━━━━━━ 2s 14ms/step - loss: 6.4652e-04 - val_loss: 7.7577e-04
Epoch 32/50
147/147 ━━━━━━━━━━━━━━━━━━━━ 2s 10ms/step - loss: 6.5484e-04 - val_loss: 7.7076e-04
Epoch 33/50
147/147 ━━━━━━━━━━━━━━━━━━━━ 2s 11ms/step - loss: 5.6836e-04 - val_loss: 7.7560e-04
Epoch 34/50
147/147 ━━━━━━━━━━━━━━━━━━━━ 2s 11ms/step - loss: 6.2028e-04 - val_loss: 6.7884e-04
Epoch 35/50
147/147 ━━━━━━━━━━━━━━━━━━━━ 1s 10ms/step - loss: 5.8084e-04 - val_loss: 7.0083e-04
Epoch 36/50
147/147 ━━━━━━━━━━━━━━━━━━━━ 1s 10ms/step - loss: 5.4505e-04 - val_loss: 6.8947e-04
Epoch 37/50
147/147 ━━━━━━━━━━━━━━━━━━━━ 3s 14ms/step - loss: 5.7341e-04 - val_loss: 7.0743e-04
Epoch 38/50
147/147 ━━━━━━━━━━━━━━━━━━━━ 2s 12ms/step - loss: 5.4499e-04 - val_loss: 6.8666e-04
Epoch 39/50
147/147 ━━━━━━━━━━━━━━━━━━━━ 2s 11ms/step - loss: 5.1884e-04 - val_loss: 6.3949e-04
Epoch 40/50
147/147 ━━━━━━━━━━━━━━━━━━━━ 2s 11ms/step - loss: 5.2138e-04 - val_loss: 6.6032e-04
Epoch 41/50
147/147 ━━━━━━━━━━━━━━━━━━━━ 2s 10ms/step - loss: 5.0695e-04 - val_loss: 6.0819e-04
Epoch 42/50
147/147 ━━━━━━━━━━━━━━━━━━━━ 1s 10ms/step - loss: 5.0357e-04 - val_loss: 6.4788e-04
Epoch 43/50
147/147 ━━━━━━━━━━━━━━━━━━━━ 2s 12ms/step - loss: 4.7796e-04 - val_loss: 6.6161e-04
Epoch 44/50
147/147 ━━━━━━━━━━━━━━━━━━━━ 2s 12ms/step - loss: 4.9097e-04 - val_loss: 5.8568e-04
Epoch 45/50
147/147 ━━━━━━━━━━━━━━━━━━━━ 2s 11ms/step - loss: 5.0084e-04 - val_loss: 6.4351e-04
Epoch 46/50
147/147 ━━━━━━━━━━━━━━━━━━━━ 3s 11ms/step - loss: 4.4269e-04 - val_loss: 6.8195e-04
Epoch 47/50
147/147 ━━━━━━━━━━━━━━━━━━━━ 2s 11ms/step - loss: 4.5654e-04 - val_loss: 6.2308e-04
Epoch 48/50
147/147 ━━━━━━━━━━━━━━━━━━━━ 3s 12ms/step - loss: 4.8472e-04 - val_loss: 5.7101e-04
Epoch 49/50
147/147 ━━━━━━━━━━━━━━━━━━━━ 2s 13ms/step - loss: 4.4050e-04 - val_loss: 6.0716e-04
Epoch 50/50
147/147 ━━━━━━━━━━━━━━━━━━━━ 2s 10ms/step - loss: 4.4041e-04 - val_loss: 6.0926e-04
74/74 ━━━━━━━━━━━━━━━━━━━━ 1s 7ms/step
```

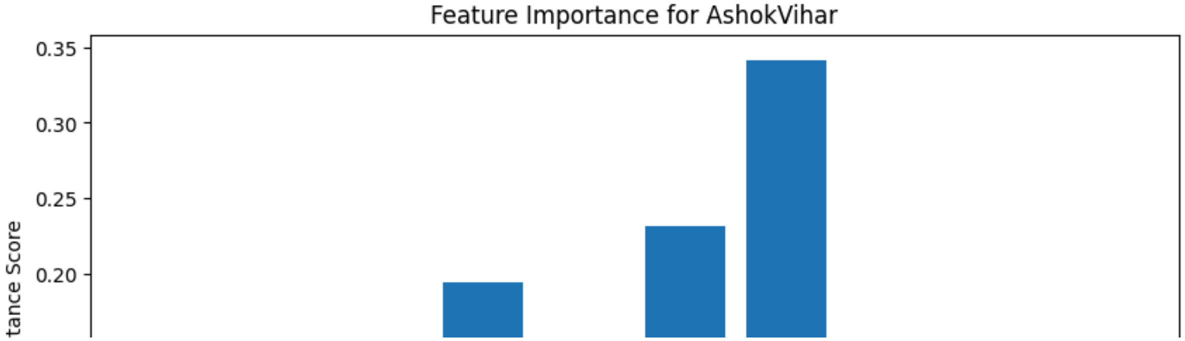

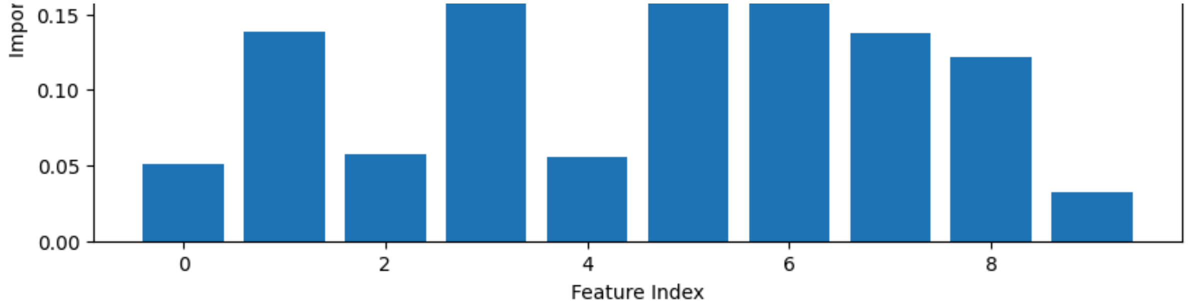

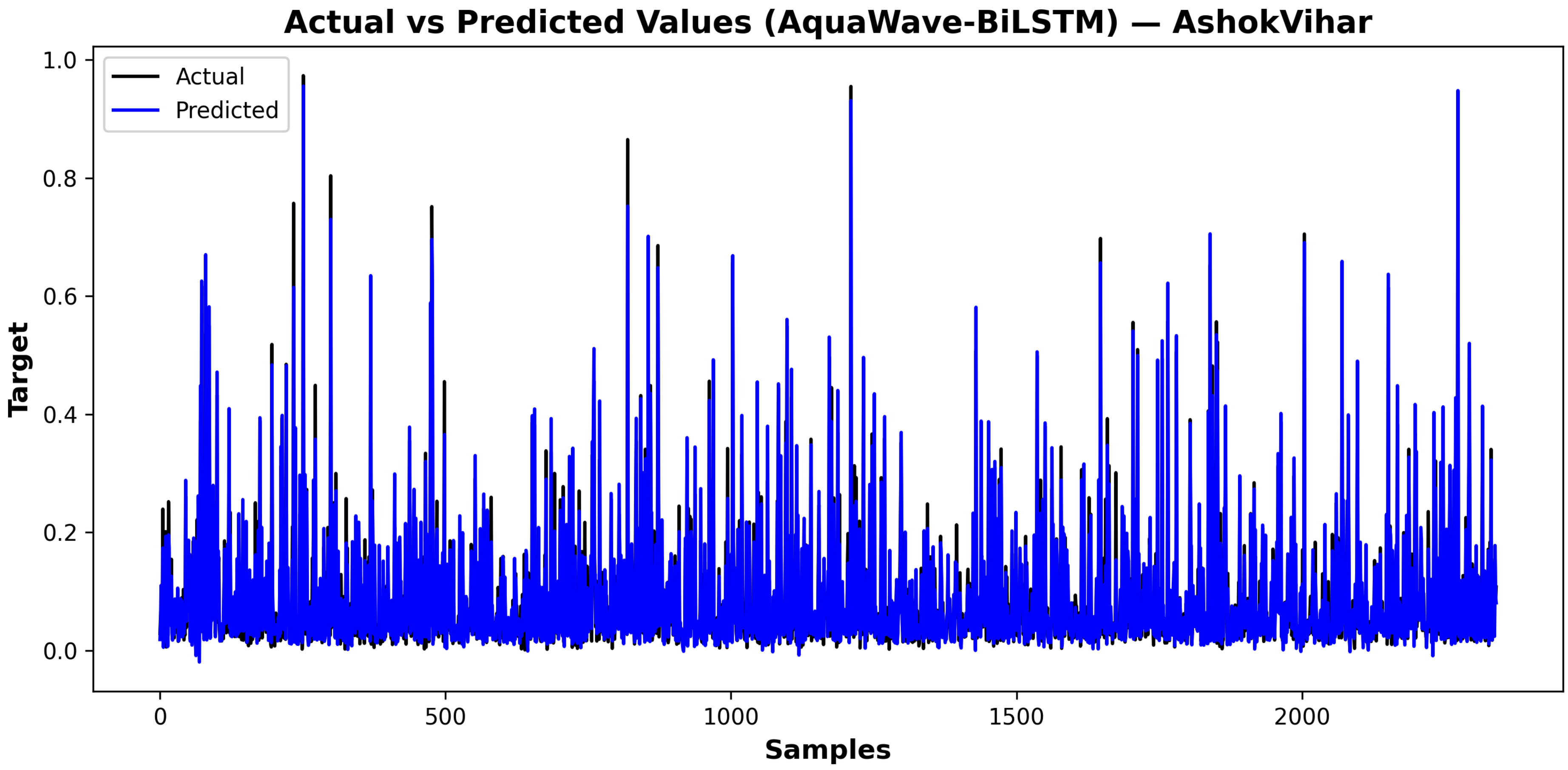


```
Processing Station: DCStadium
/usr/local/lib/python3.11/dist-packages/pywt/_multilevel.py:43: UserWarning: Level value of 3 is too high: all coefficients will experience boundary effects.
  warnings.warn(
Epoch 1/50
/usr/local/lib/python3.11/dist-packages/keras/src/layers/rnn/rnn.py:200: UserWarning: Do not pass an `input_shape`/`input_dim` argument to a layer. When using Sequential mod
  super().__init__(**kwargs)
147/147 ━━━━━━━━━━━━━━━━━━━━ 6s 17ms/step - loss: 0.0240 - val_loss: 0.0102
Epoch 2/50
147/147 ━━━━━━━━━━━━━━━━━━━━ 4s 11ms/step - loss: 0.0079 - val_loss: 0.0030
```

```
Epoch 3/50
147/147 ━━━━━━━━━━━━━━━━━━━━ 3s 11ms/step - loss: 0.0029 - val_loss: 0.0023
Epoch 4/50
147/147 ━━━━━━━━━━━━━━━━━━━━ 3s 11ms/step - loss: 0.0022 - val_loss: 0.0021
Epoch 5/50
147/147 ━━━━━━━━━━━━━━━━━━━━ 2s 15ms/step - loss: 0.0021 - val_loss: 0.0025
Epoch 6/50
147/147 ━━━━━━━━━━━━━━━━━━━━ 2s 11ms/step - loss: 0.0022 - val_loss: 0.0018
Epoch 7/50
147/147 ━━━━━━━━━━━━━━━━━━━━ 2s 10ms/step - loss: 0.0020 - val_loss: 0.0018
Epoch 8/50
147/147 ━━━━━━━━━━━━━━━━━━━━ 2s 11ms/step - loss: 0.0019 - val_loss: 0.0016
Epoch 9/50
147/147 ━━━━━━━━━━━━━━━━━━━━ 2s 10ms/step - loss: 0.0017 - val_loss: 0.0020
Epoch 10/50
147/147 ━━━━━━━━━━━━━━━━━━━━ 2s 11ms/step - loss: 0.0018 - val_loss: 0.0015
Epoch 11/50
147/147 ━━━━━━━━━━━━━━━━━━━━ 2s 11ms/step - loss: 0.0016 - val_loss: 0.0016
Epoch 12/50
147/147 ━━━━━━━━━━━━━━━━━━━━ 3s 14ms/step - loss: 0.0016 - val_loss: 0.0015
Epoch 13/50
147/147 ━━━━━━━━━━━━━━━━━━━━ 2s 10ms/step - loss: 0.0015 - val_loss: 0.0015
Epoch 14/50
147/147 ━━━━━━━━━━━━━━━━━━━━ 2s 11ms/step - loss: 0.0016 - val_loss: 0.0015
Epoch 15/50
147/147 ━━━━━━━━━━━━━━━━━━━━ 2s 10ms/step - loss: 0.0014 - val_loss: 0.0015
Epoch 16/50
147/147 ━━━━━━━━━━━━━━━━━━━━ 2s 11ms/step - loss: 0.0013 - val_loss: 0.0015
Epoch 17/50
147/147 ━━━━━━━━━━━━━━━━━━━━ 2s 11ms/step - loss: 0.0015 - val_loss: 0.0014
Epoch 18/50
147/147 ━━━━━━━━━━━━━━━━━━━━ 3s 15ms/step - loss: 0.0013 - val_loss: 0.0014
Epoch 19/50
147/147 ━━━━━━━━━━━━━━━━━━━━ 2s 11ms/step - loss: 0.0015 - val_loss: 0.0013
Epoch 20/50
147/147 ━━━━━━━━━━━━━━━━━━━━ 2s 10ms/step - loss: 0.0013 - val_loss: 0.0014
Epoch 21/50
147/147 ━━━━━━━━━━━━━━━━━━━━ 2s 10ms/step - loss: 0.0012 - val_loss: 0.0012
Epoch 22/50
147/147 ━━━━━━━━━━━━━━━━━━━━ 2s 10ms/step - loss: 0.0013 - val_loss: 0.0012
Epoch 23/50
147/147 ━━━━━━━━━━━━━━━━━━━━ 2s 12ms/step - loss: 0.0012 - val_loss: 0.0011
Epoch 24/50
147/147 ━━━━━━━━━━━━━━━━━━━━ 3s 15ms/step - loss: 0.0013 - val_loss: 0.0011
Epoch 25/50
147/147 ━━━━━━━━━━━━━━━━━━━━ 2s 10ms/step - loss: 0.0012 - val_loss: 0.0011
Epoch 26/50
147/147 ━━━━━━━━━━━━━━━━━━━━ 3s 11ms/step - loss: 0.0011 - val_loss: 0.0011
Epoch 27/50
147/147 ━━━━━━━━━━━━━━━━━━━━ 2s 10ms/step - loss: 0.0011 - val_loss: 0.0011
Epoch 28/50
147/147 ━━━━━━━━━━━━━━━━━━━━ 2s 11ms/step - loss: 0.0011 - val_loss: 0.0010
Epoch 29/50
147/147 ━━━━━━━━━━━━━━━━━━━━ 3s 11ms/step - loss: 0.0010 - val_loss: 9.5706e-04
Epoch 30/50
147/147 ━━━━━━━━━━━━━━━━━━━━ 3s 13ms/step - loss: 0.0011 - val_loss: 0.0010
Epoch 31/50
147/147 ━━━━━━━━━━━━━━━━━━━━ 2s 10ms/step - loss: 0.0010 - val_loss: 0.0011
Epoch 32/50
147/147 ━━━━━━━━━━━━━━━━━━━━ 2s 11ms/step - loss: 0.0011 - val_loss: 0.0010
Epoch 33/50
```

```
147/147 ━━━━━━━━━━━━━━━━━━━━ 2s 10ms/step - loss: 9.5586e-04 - val_loss: 9.9304e-04
Epoch 34/50
147/147 ━━━━━━━━━━━━━━━━━━━━ 2s 11ms/step - loss: 9.1685e-04 - val_loss: 0.0011
Epoch 35/50
147/147 ━━━━━━━━━━━━━━━━━━━━ 3s 12ms/step - loss: 9.5118e-04 - val_loss: 9.8264e-04
Epoch 36/50
147/147 ━━━━━━━━━━━━━━━━━━━━ 3s 12ms/step - loss: 9.6729e-04 - val_loss: 9.3751e-04
Epoch 37/50
147/147 ━━━━━━━━━━━━━━━━━━━━ 2s 10ms/step - loss: 8.5755e-04 - val_loss: 9.5756e-04
Epoch 38/50
147/147 ━━━━━━━━━━━━━━━━━━━━ 2s 11ms/step - loss: 9.9093e-04 - val_loss: 9.3977e-04
Epoch 39/50
147/147 ━━━━━━━━━━━━━━━━━━━━ 3s 11ms/step - loss: 0.0011 - val_loss: 9.1480e-04
Epoch 40/50
147/147 ━━━━━━━━━━━━━━━━━━━━ 2s 11ms/step - loss: 8.8225e-04 - val_loss: 9.2910e-04
Epoch 41/50
147/147 ━━━━━━━━━━━━━━━━━━━━ 3s 15ms/step - loss: 8.5641e-04 - val_loss: 9.1718e-04
Epoch 42/50
147/147 ━━━━━━━━━━━━━━━━━━━━ 2s 11ms/step - loss: 9.0843e-04 - val_loss: 9.8813e-04
Epoch 43/50
147/147 ━━━━━━━━━━━━━━━━━━━━ 2s 11ms/step - loss: 7.8963e-04 - val_loss: 0.0010
Epoch 44/50
147/147 ━━━━━━━━━━━━━━━━━━━━ 2s 11ms/step - loss: 8.0808e-04 - val_loss: 8.8600e-04
Epoch 45/50
147/147 ━━━━━━━━━━━━━━━━━━━━ 2s 11ms/step - loss: 8.2692e-04 - val_loss: 9.2593e-04
Epoch 46/50
147/147 ━━━━━━━━━━━━━━━━━━━━ 2s 10ms/step - loss: 8.8112e-04 - val_loss: 8.7196e-04
Epoch 47/50
147/147 ━━━━━━━━━━━━━━━━━━━━ 2s 10ms/step - loss: 7.8822e-04 - val_loss: 9.1197e-04
Epoch 48/50
147/147 ━━━━━━━━━━━━━━━━━━━━ 3s 15ms/step - loss: 7.4743e-04 - val_loss: 8.6277e-04
Epoch 49/50
147/147 ━━━━━━━━━━━━━━━━━━━━ 2s 11ms/step - loss: 7.2300e-04 - val_loss: 9.3604e-04
Epoch 50/50
147/147 ━━━━━━━━━━━━━━━━━━━━ 2s 11ms/step - loss: 7.6066e-04 - val_loss: 8.1831e-04
74/74 ━━━━━━━━━━━━━━━━━━━━ 1s 8ms/step
```

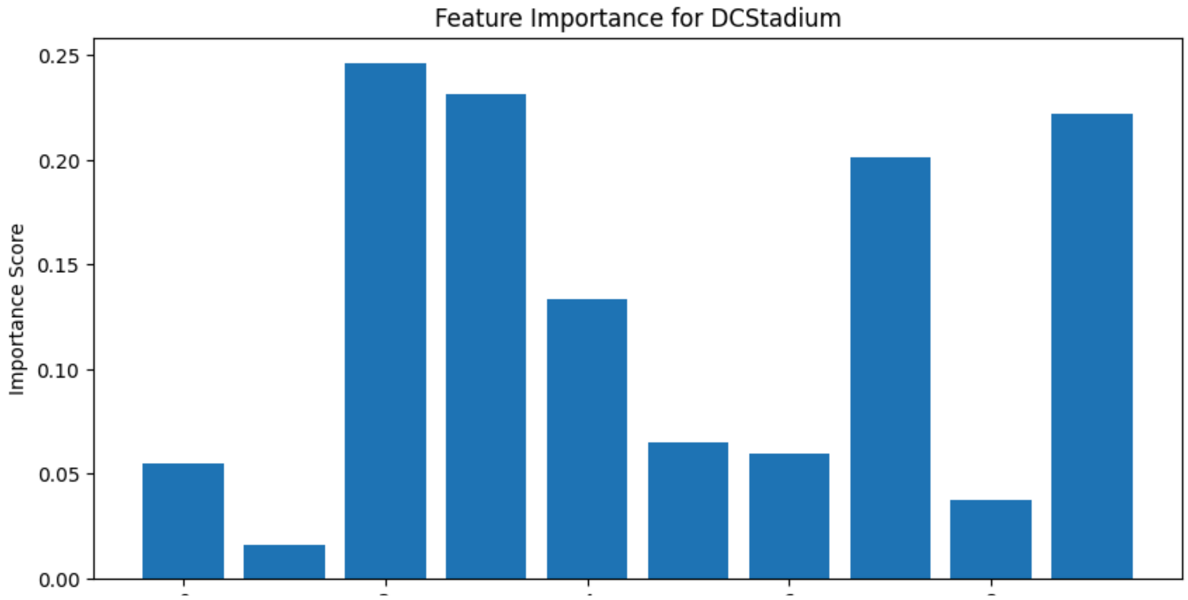

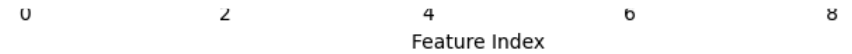

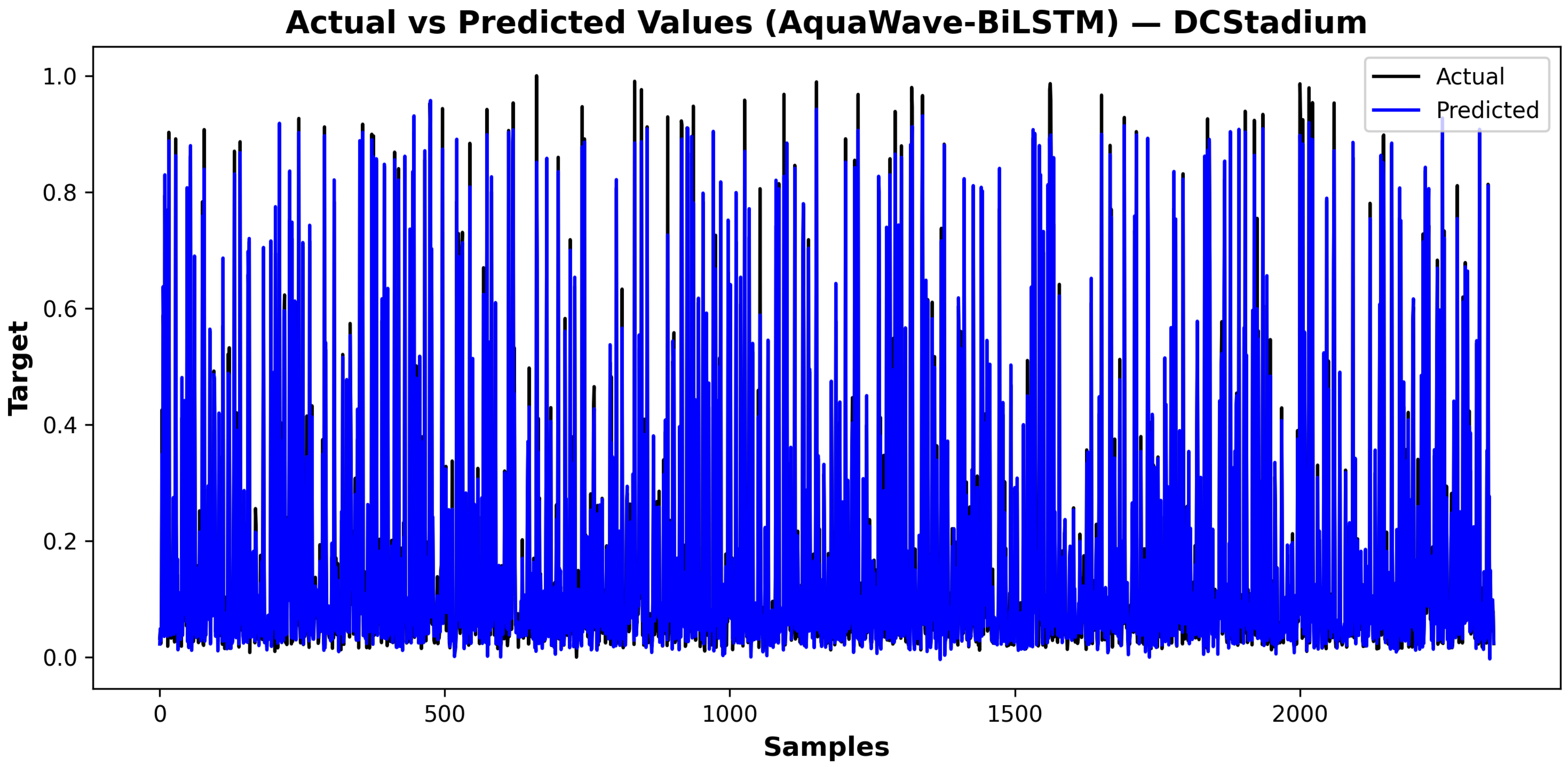


```
Processing Station: DwarkaSec8
/usr/local/lib/python3.11/dist-packages/pywt/_multilevel.py:43: UserWarning: Level value of 3 is too high: all coefficients will experience boundary effects.
  warnings.warn(
Epoch 1/50
/usr/local/lib/python3.11/dist-packages/keras/src/layers/rnn/rnn.py:200: UserWarning: Do not pass an `input_shape`/`input_dim` argument to a layer. When using Sequential mod
  super().__init__(**kwargs)
147/147 ━━━━━━━━━━━━━━━━━━━━ 6s 15ms/step - loss: 0.0079 - val_loss: 0.0035
Epoch 2/50
147/147 ━━━━━━━━━━━━━━━━━━━━ 2s 11ms/step - loss: 0.0030 - val_loss: 0.0021
Epoch 3/50
147/147 ━━━━━━━━━━━━━━━━━━━━ 2s 12ms/step - loss: 0.0019 - val_loss: 0.0018
Epoch 4/50
147/147 ━━━━━━━━━━━━━━━━━━━━ 2s 11ms/step - loss: 0.0017 - val_loss: 0.0015
Epoch 5/50
147/147 ━━━━━━━━━━━━━━━━━━━━ 2s 12ms/step - loss: 0.0015 - val_loss: 0.0014
Epoch 6/50
147/147 ━━━━━━━━━━━━━━━━━━━━ 2s 15ms/step - loss: 0.0016 - val_loss: 0.0014
Epoch 7/50
147/147 ━━━━━━━━━━━━━━━━━━━━ 2s 11ms/step - loss: 0.0013 - val_loss: 0.0013
Epoch 8/50
```

```
Epoch 8/50
147/147 ━━━━━━━━━━━━━━━━━━━━ 3s 12ms/step - loss: 0.0012 - val_loss: 0.0014
Epoch 9/50
147/147 ━━━━━━━━━━━━━━━━━━━━ 2s 11ms/step - loss: 0.0012 - val_loss: 0.0012
Epoch 10/50
147/147 ━━━━━━━━━━━━━━━━━━━━ 2s 11ms/step - loss: 0.0012 - val_loss: 0.0012
Epoch 11/50
147/147 ━━━━━━━━━━━━━━━━━━━━ 3s 15ms/step - loss: 0.0012 - val_loss: 0.0013
Epoch 12/50
147/147 ━━━━━━━━━━━━━━━━━━━━ 2s 11ms/step - loss: 0.0011 - val_loss: 0.0012
Epoch 13/50
147/147 ━━━━━━━━━━━━━━━━━━━━ 2s 11ms/step - loss: 0.0011 - val_loss: 0.0012
Epoch 14/50
147/147 ━━━━━━━━━━━━━━━━━━━━ 2s 11ms/step - loss: 0.0012 - val_loss: 0.0011
Epoch 15/50
147/147 ━━━━━━━━━━━━━━━━━━━━ 3s 12ms/step - loss: 0.0011 - val_loss: 0.0011
Epoch 16/50
147/147 ━━━━━━━━━━━━━━━━━━━━ 2s 12ms/step - loss: 0.0011 - val_loss: 0.0013
Epoch 17/50
147/147 ━━━━━━━━━━━━━━━━━━━━ 2s 14ms/step - loss: 0.0010 - val_loss: 0.0010
Epoch 18/50
147/147 ━━━━━━━━━━━━━━━━━━━━ 2s 14ms/step - loss: 9.5366e-04 - val_loss: 0.0010
Epoch 19/50
147/147 ━━━━━━━━━━━━━━━━━━━━ 2s 11ms/step - loss: 9.5810e-04 - val_loss: 0.0010
Epoch 20/50
147/147 ━━━━━━━━━━━━━━━━━━━━ 3s 11ms/step - loss: 9.3303e-04 - val_loss: 0.0011
Epoch 21/50
147/147 ━━━━━━━━━━━━━━━━━━━━ 3s 12ms/step - loss: 9.4835e-04 - val_loss: 0.0010
Epoch 22/50
147/147 ━━━━━━━━━━━━━━━━━━━━ 2s 10ms/step - loss: 9.2532e-04 - val_loss: 9.5033e-04
Epoch 23/50
147/147 ━━━━━━━━━━━━━━━━━━━━ 3s 13ms/step - loss: 7.9981e-04 - val_loss: 9.9242e-04
Epoch 24/50
147/147 ━━━━━━━━━━━━━━━━━━━━ 2s 11ms/step - loss: 9.1784e-04 - val_loss: 8.9366e-04
Epoch 25/50
147/147 ━━━━━━━━━━━━━━━━━━━━ 2s 11ms/step - loss: 8.4033e-04 - val_loss: 9.5961e-04
Epoch 26/50
147/147 ━━━━━━━━━━━━━━━━━━━━ 3s 11ms/step - loss: 8.0283e-04 - val_loss: 9.2446e-04
Epoch 27/50
147/147 ━━━━━━━━━━━━━━━━━━━━ 2s 11ms/step - loss: 8.3330e-04 - val_loss: 0.0010
Epoch 28/50
147/147 ━━━━━━━━━━━━━━━━━━━━ 2s 13ms/step - loss: 8.5294e-04 - val_loss: 8.9030e-04
Epoch 29/50
147/147 ━━━━━━━━━━━━━━━━━━━━ 2s 12ms/step - loss: 7.6789e-04 - val_loss: 9.4120e-04
Epoch 30/50
147/147 ━━━━━━━━━━━━━━━━━━━━ 2s 12ms/step - loss: 7.9267e-04 - val_loss: 9.3479e-04
Epoch 31/50
147/147 ━━━━━━━━━━━━━━━━━━━━ 2s 11ms/step - loss: 7.4607e-04 - val_loss: 9.0949e-04
Epoch 32/50
147/147 ━━━━━━━━━━━━━━━━━━━━ 3s 12ms/step - loss: 7.4210e-04 - val_loss: 8.4407e-04
Epoch 33/50
147/147 ━━━━━━━━━━━━━━━━━━━━ 2s 11ms/step - loss: 7.5090e-04 - val_loss: 9.0248e-04
Epoch 34/50
147/147 ━━━━━━━━━━━━━━━━━━━━ 2s 13ms/step - loss: 7.2519e-04 - val_loss: 8.8671e-04
Epoch 35/50
147/147 ━━━━━━━━━━━━━━━━━━━━ 2s 14ms/step - loss: 7.4260e-04 - val_loss: 8.3358e-04
Epoch 36/50
147/147 ━━━━━━━━━━━━━━━━━━━━ 2s 11ms/step - loss: 7.0418e-04 - val_loss: 8.0379e-04
Epoch 37/50
147/147 ━━━━━━━━━━━━━━━━━━━━ 2s 12ms/step - loss: 7.1060e-04 - val_loss: 8.4288e-04
Epoch 38/50
[illegible]
```

```
147/147 ━━━━━━━━━━━━━━━━━━━━ 2s 11ms/step - loss: 6.2371e-04 - val_loss: 8.0337e-04
Epoch 39/50
147/147 ━━━━━━━━━━━━━━━━━━━━ 2s 11ms/step - loss: 6.8085e-04 - val_loss: 8.5773e-04
Epoch 40/50
147/147 ━━━━━━━━━━━━━━━━━━━━ 2s 11ms/step - loss: 7.3067e-04 - val_loss: 8.3688e-04
Epoch 41/50
147/147 ━━━━━━━━━━━━━━━━━━━━ 2s 13ms/step - loss: 6.6540e-04 - val_loss: 8.6025e-04
Epoch 42/50
147/147 ━━━━━━━━━━━━━━━━━━━━ 3s 13ms/step - loss: 6.6035e-04 - val_loss: 7.6417e-04
Epoch 43/50
147/147 ━━━━━━━━━━━━━━━━━━━━ 2s 12ms/step - loss: 6.0980e-04 - val_loss: 8.1245e-04
Epoch 44/50
147/147 ━━━━━━━━━━━━━━━━━━━━ 2s 12ms/step - loss: 6.1043e-04 - val_loss: 9.2302e-04
Epoch 45/50
147/147 ━━━━━━━━━━━━━━━━━━━━ 2s 11ms/step - loss: 6.6146e-04 - val_loss: 7.7822e-04
Epoch 46/50
147/147 ━━━━━━━━━━━━━━━━━━━━ 2s 11ms/step - loss: 6.1102e-04 - val_loss: 7.7020e-04
Epoch 47/50
147/147 ━━━━━━━━━━━━━━━━━━━━ 2s 11ms/step - loss: 6.2339e-04 - val_loss: 7.4463e-04
Epoch 48/50
147/147 ━━━━━━━━━━━━━━━━━━━━ 3s 16ms/step - loss: 6.2545e-04 - val_loss: 8.4060e-04
Epoch 49/50
147/147 ━━━━━━━━━━━━━━━━━━━━ 2s 12ms/step - loss: 6.1862e-04 - val_loss: 7.2221e-04
Epoch 50/50
147/147 ━━━━━━━━━━━━━━━━━━━━ 2s 12ms/step - loss: 5.6658e-04 - val_loss: 8.0936e-04
74/74 ━━━━━━━━━━━━━━━━━━━━ 11s 140ms/step
```

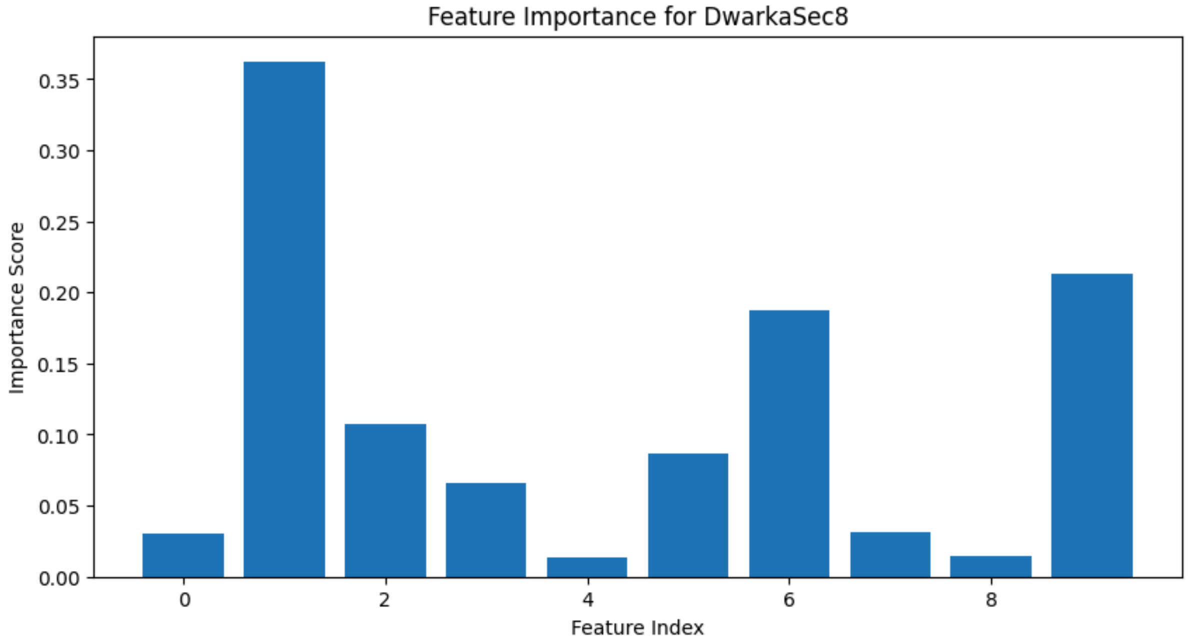

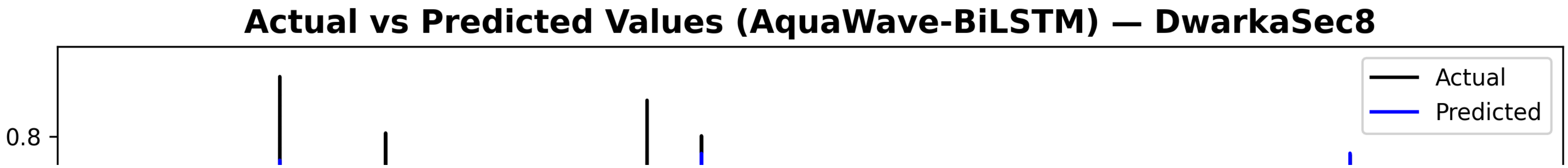

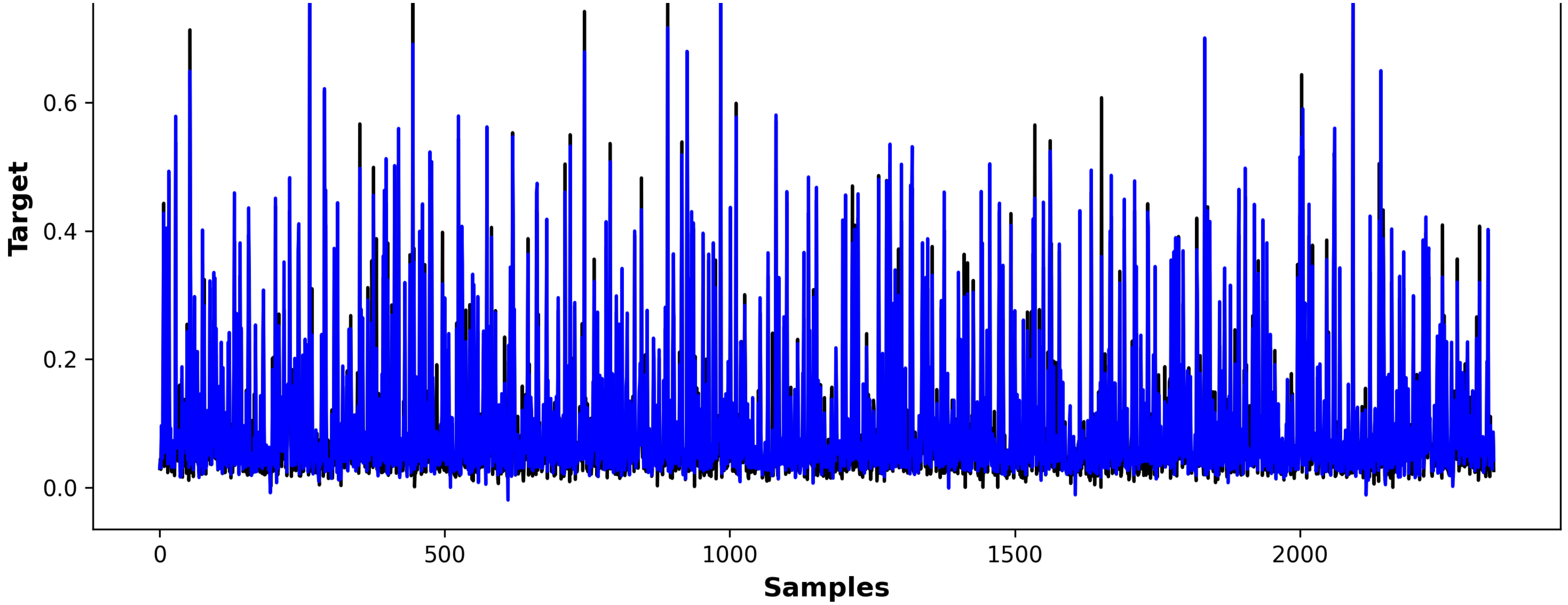


```
Processing Station: NehruNagar
/usr/local/lib/python3.11/dist-packages/pywt/_multilevel.py:43: UserWarning: Level value of 3 is too high: all coefficients will experience boundary effects.
  warnings.warn(
Epoch 1/50
/usr/local/lib/python3.11/dist-packages/keras/src/layers/rnn/rnn.py:200: UserWarning: Do not pass an `input_shape`/`input_dim` argument to a layer. When using Sequential mod
  super().__init__(**kwargs)
147/147 ━━━━━━━━━━━━━━━━━━━━ 6s 20ms/step - loss: 0.0155 - val_loss: 0.0052
Epoch 2/50
147/147 ━━━━━━━━━━━━━━━━━━━━ 4s 12ms/step - loss: 0.0046 - val_loss: 0.0030
Epoch 3/50
147/147 ━━━━━━━━━━━━━━━━━━━━ 3s 13ms/step - loss: 0.0026 - val_loss: 0.0023
Epoch 4/50
147/147 ━━━━━━━━━━━━━━━━━━━━ 2s 11ms/step - loss: 0.0020 - val_loss: 0.0020
Epoch 5/50
147/147 ━━━━━━━━━━━━━━━━━━━━ 2s 13ms/step - loss: 0.0018 - val_loss: 0.0021
Epoch 6/50
147/147 ━━━━━━━━━━━━━━━━━━━━ 3s 13ms/step - loss: 0.0017 - val_loss: 0.0019
Epoch 7/50
147/147 ━━━━━━━━━━━━━━━━━━━━ 2s 11ms/step - loss: 0.0017 - val_loss: 0.0017
Epoch 8/50
147/147 ━━━━━━━━━━━━━━━━━━━━ 3s 12ms/step - loss: 0.0016 - val_loss: 0.0017
Epoch 9/50
147/147 ━━━━━━━━━━━━━━━━━━━━ 2s 11ms/step - loss: 0.0015 - val_loss: 0.0020
Epoch 10/50
147/147 ━━━━━━━━━━━━━━━━━━━━ 2s 12ms/step - loss: 0.0016 - val_loss: 0.0015
Epoch 11/50
147/147 ━━━━━━━━━━━━━━━━━━━━ 2s 15ms/step - loss: 0.0015 - val_loss: 0.0016
Epoch 12/50
147/147 ━━━━━━━━━━━━━━━━━━━━ 2s 15ms/step - loss: 0.0014 - val_loss: 0.0014
Epoch 13/50
```

```
147/147 ━━━━━━━━━━━━━━━━━━━━ 2s 12ms/step - loss: 0.0013 - val_loss: 0.0015
Epoch 14/50
147/147 ━━━━━━━━━━━━━━━━━━━━ 2s 11ms/step - loss: 0.0013 - val_loss: 0.0015
Epoch 15/50
147/147 ━━━━━━━━━━━━━━━━━━━━ 3s 13ms/step - loss: 0.0013 - val_loss: 0.0014
Epoch 16/50
147/147 ━━━━━━━━━━━━━━━━━━━━ 2s 12ms/step - loss: 0.0012 - val_loss: 0.0013
Epoch 17/50
147/147 ━━━━━━━━━━━━━━━━━━━━ 2s 16ms/step - loss: 0.0011 - val_loss: 0.0011
Epoch 18/50
147/147 ━━━━━━━━━━━━━━━━━━━━ 2s 12ms/step - loss: 0.0010 - val_loss: 0.0011
Epoch 19/50
147/147 ━━━━━━━━━━━━━━━━━━━━ 2s 12ms/step - loss: 9.8821e-04 - val_loss: 0.0012
Epoch 20/50
147/147 ━━━━━━━━━━━━━━━━━━━━ 2s 12ms/step - loss: 9.3904e-04 - val_loss: 0.0010
Epoch 21/50
147/147 ━━━━━━━━━━━━━━━━━━━━ 3s 13ms/step - loss: 8.5555e-04 - val_loss: 9.1993e-04
Epoch 22/50
147/147 ━━━━━━━━━━━━━━━━━━━━ 2s 12ms/step - loss: 9.0505e-04 - val_loss: 8.5472e-04
Epoch 23/50
147/147 ━━━━━━━━━━━━━━━━━━━━ 3s 16ms/step - loss: 7.6004e-04 - val_loss: 0.0010
Epoch 24/50
147/147 ━━━━━━━━━━━━━━━━━━━━ 2s 13ms/step - loss: 7.7434e-04 - val_loss: 9.3562e-04
Epoch 25/50
147/147 ━━━━━━━━━━━━━━━━━━━━ 2s 12ms/step - loss: 7.7800e-04 - val_loss: 8.4200e-04
Epoch 26/50
147/147 ━━━━━━━━━━━━━━━━━━━━ 2s 11ms/step - loss: 7.1634e-04 - val_loss: 8.0029e-04
Epoch 27/50
147/147 ━━━━━━━━━━━━━━━━━━━━ 2s 11ms/step - loss: 7.6188e-04 - val_loss: 7.6871e-04
Epoch 28/50
147/147 ━━━━━━━━━━━━━━━━━━━━ 2s 13ms/step - loss: 7.4852e-04 - val_loss: 7.6345e-04
Epoch 29/50
147/147 ━━━━━━━━━━━━━━━━━━━━ 2s 16ms/step - loss: 7.0796e-04 - val_loss: 8.0650e-04
Epoch 30/50
147/147 ━━━━━━━━━━━━━━━━━━━━ 2s 12ms/step - loss: 7.4001e-04 - val_loss: 7.9073e-04
Epoch 31/50
147/147 ━━━━━━━━━━━━━━━━━━━━ 2s 12ms/step - loss: 6.5534e-04 - val_loss: 7.0603e-04
Epoch 32/50
147/147 ━━━━━━━━━━━━━━━━━━━━ 3s 12ms/step - loss: 6.1438e-04 - val_loss: 7.4213e-04
Epoch 33/50
147/147 ━━━━━━━━━━━━━━━━━━━━ 3s 12ms/step - loss: 6.0709e-04 - val_loss: 7.5112e-04
Epoch 34/50
147/147 ━━━━━━━━━━━━━━━━━━━━ 3s 15ms/step - loss: 6.4207e-04 - val_loss: 7.2726e-04
Epoch 35/50
147/147 ━━━━━━━━━━━━━━━━━━━━ 2s 14ms/step - loss: 5.9039e-04 - val_loss: 7.1557e-04
Epoch 36/50
147/147 ━━━━━━━━━━━━━━━━━━━━ 2s 11ms/step - loss: 6.2288e-04 - val_loss: 6.5707e-04
Epoch 37/50
147/147 ━━━━━━━━━━━━━━━━━━━━ 2s 11ms/step - loss: 5.6954e-04 - val_loss: 6.9753e-04
Epoch 38/50
147/147 ━━━━━━━━━━━━━━━━━━━━ 2s 12ms/step - loss: 5.5687e-04 - val_loss: 7.1965e-04
Epoch 39/50
147/147 ━━━━━━━━━━━━━━━━━━━━ 2s 12ms/step - loss: 6.5143e-04 - val_loss: 6.1995e-04
Epoch 40/50
147/147 ━━━━━━━━━━━━━━━━━━━━ 3s 13ms/step - loss: 5.7413e-04 - val_loss: 6.4295e-04
Epoch 41/50
147/147 ━━━━━━━━━━━━━━━━━━━━ 2s 16ms/step - loss: 5.3995e-04 - val_loss: 6.5550e-04
Epoch 42/50
147/147 ━━━━━━━━━━━━━━━━━━━━ 2s 12ms/step - loss: 5.4497e-04 - val_loss: 6.5221e-04
Epoch 43/50
147/147 ━━━━━━━━━━━━━━━━━━━━ 2s 11ms/step - loss: 5.3993e-04 - val_loss: 7.2756e-04
```

```
Epoch 44/50
147/147 ━━━━━━━━━━━━━━━━━━━━ 2s 12ms/step - loss: 5.6236e-04 - val_loss: 6.3477e-04
Epoch 45/50
147/147 ━━━━━━━━━━━━━━━━━━━━ 2s 12ms/step - loss: 5.2460e-04 - val_loss: 5.6959e-04
Epoch 46/50
147/147 ━━━━━━━━━━━━━━━━━━━━ 2s 11ms/step - loss: 4.7059e-04 - val_loss: 5.6275e-04
Epoch 47/50
147/147 ━━━━━━━━━━━━━━━━━━━━ 3s 16ms/step - loss: 4.9726e-04 - val_loss: 6.3593e-04
Epoch 48/50
147/147 ━━━━━━━━━━━━━━━━━━━━ 2s 12ms/step - loss: 4.7922e-04 - val_loss: 6.5915e-04
Epoch 49/50
147/147 ━━━━━━━━━━━━━━━━━━━━ 2s 11ms/step - loss: 4.9453e-04 - val_loss: 5.6943e-04
Epoch 50/50
147/147 ━━━━━━━━━━━━━━━━━━━━ 3s 12ms/step - loss: 5.0217e-04 - val_loss: 5.6885e-04
74/74 ━━━━━━━━━━━━━━━━━━━━ 1s 8ms/step
```

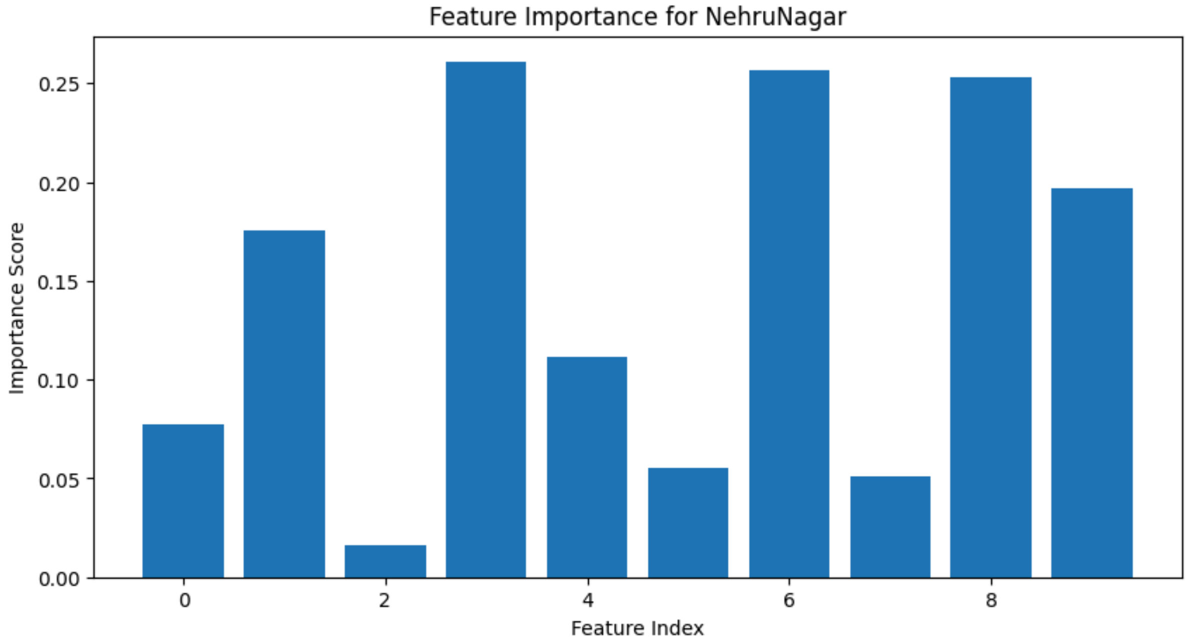

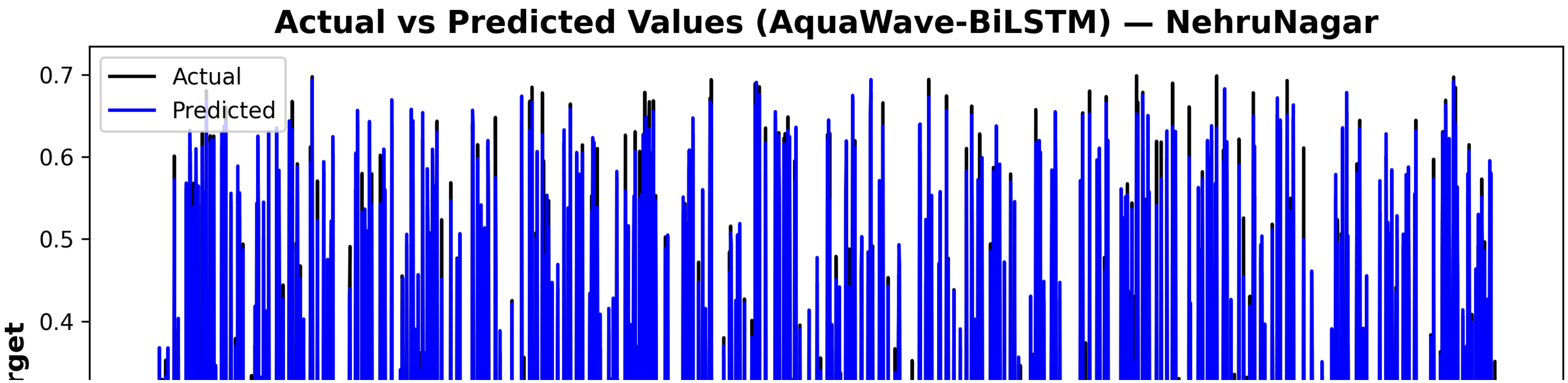

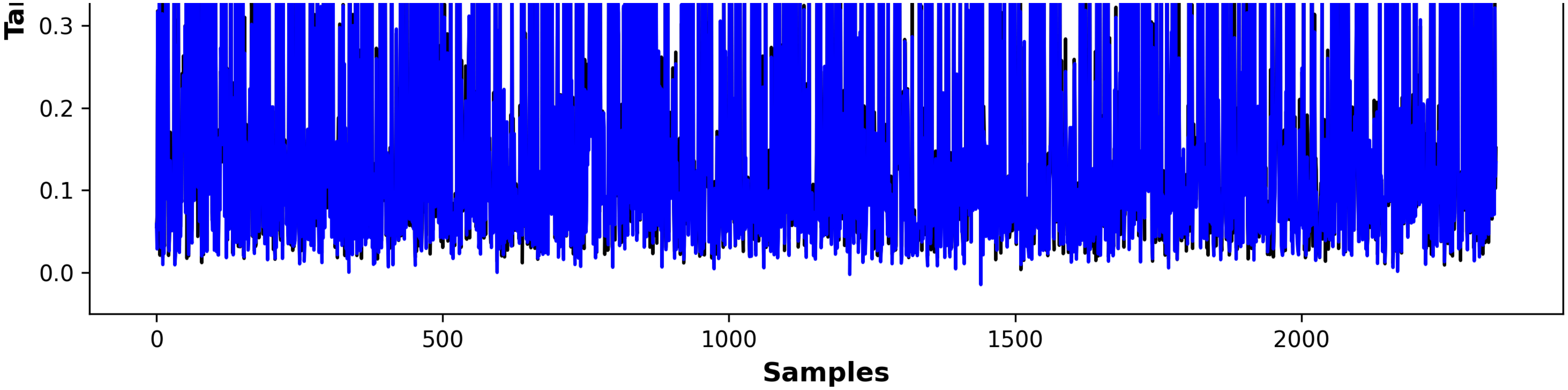


```
Processing Station: Najafgarh
/usr/local/lib/python3.11/dist-packages/pywt/_multilevel.py:43: UserWarning: Level value of 3 is too high: all coefficients will experience boundary effects.
  warnings.warn(
Epoch 1/50
/usr/local/lib/python3.11/dist-packages/keras/src/layers/rnn/rnn.py:200: UserWarning: Do not pass an `input_shape`/`input_dim` argument to a layer. When using Sequential mod
  super().__init__(**kwargs)
147/147 ━━━━━━━━━━━━━━━━━━━━ 6s 15ms/step - loss: 0.0046 - val_loss: 0.0021
Epoch 2/50
147/147 ━━━━━━━━━━━━━━━━━━━━ 2s 13ms/step - loss: 0.0021 - val_loss: 0.0016
Epoch 3/50
147/147 ━━━━━━━━━━━━━━━━━━━━ 3s 13ms/step - loss: 0.0016 - val_loss: 0.0016
Epoch 4/50
147/147 ━━━━━━━━━━━━━━━━━━━━ 3s 17ms/step - loss: 0.0014 - val_loss: 0.0013
Epoch 5/50
147/147 ━━━━━━━━━━━━━━━━━━━━ 2s 12ms/step - loss: 0.0012 - val_loss: 0.0012
Epoch 6/50
147/147 ━━━━━━━━━━━━━━━━━━━━ 2s 12ms/step - loss: 0.0013 - val_loss: 0.0011
Epoch 7/50
147/147 ━━━━━━━━━━━━━━━━━━━━ 3s 13ms/step - loss: 0.0012 - val_loss: 0.0011
Epoch 8/50
147/147 ━━━━━━━━━━━━━━━━━━━━ 3s 13ms/step - loss: 0.0012 - val_loss: 0.0011
Epoch 9/50
147/147 ━━━━━━━━━━━━━━━━━━━━ 2s 15ms/step - loss: 0.0010 - val_loss: 0.0011
Epoch 10/50
147/147 ━━━━━━━━━━━━━━━━━━━━ 2s 15ms/step - loss: 0.0011 - val_loss: 0.0011
Epoch 11/50
147/147 ━━━━━━━━━━━━━━━━━━━━ 2s 13ms/step - loss: 0.0011 - val_loss: 0.0010
Epoch 12/50
147/147 ━━━━━━━━━━━━━━━━━━━━ 2s 13ms/step - loss: 9.5443e-04 - val_loss: 9.4883e-04
Epoch 13/50
147/147 ━━━━━━━━━━━━━━━━━━━━ 2s 13ms/step - loss: 9.7756e-04 - val_loss: 9.5327e-04
Epoch 14/50
147/147 ━━━━━━━━━━━━━━━━━━━━ 2s 13ms/step - loss: 8.6286e-04 - val_loss: 0.0012
Epoch 15/50
147/147 ━━━━━━━━━━━━━━━━━━━━ 3s 13ms/step - loss: 9.1430e-04 - val_loss: 8.6186e-04
Epoch 16/50
147/147 ━━━━━━━━━━━━━━━━━━━━ 3s 15ms/step - loss: 9.2921e-04 - val_loss: 8.2499e-04
Epoch 17/50
147/147 ━━━━━━━━━━━━━━━━━━━━ 2s 12ms/step - loss: 8.0772e-04 - val_loss: 9.6771e-04
Epoch 18/50
147/147 ━━━━━━━━━━━━━━━━━━━━ 3s 13ms/step - loss: 7.7517e-04 - val_loss: 7.6392e-04
```

```
[illegible]
Epoch 19/50
147/147 ━━━━━━━━━━━━━━━━━━━━ 2s 12ms/step - loss: 7.3053e-04 - val_loss: 9.1903e-04
Epoch 20/50
147/147 ━━━━━━━━━━━━━━━━━━━━ 3s 14ms/step - loss: 7.8389e-04 - val_loss: 7.2917e-04
Epoch 21/50
147/147 ━━━━━━━━━━━━━━━━━━━━ 2s 16ms/step - loss: 6.3983e-04 - val_loss: 7.4550e-04
Epoch 22/50
147/147 ━━━━━━━━━━━━━━━━━━━━ 2s 12ms/step - loss: 6.5064e-04 - val_loss: 6.8244e-04
Epoch 23/50
147/147 ━━━━━━━━━━━━━━━━━━━━ 3s 13ms/step - loss: 6.0221e-04 - val_loss: 6.8698e-04
Epoch 24/50
147/147 ━━━━━━━━━━━━━━━━━━━━ 2s 13ms/step - loss: 6.9526e-04 - val_loss: 6.4670e-04
Epoch 25/50
147/147 ━━━━━━━━━━━━━━━━━━━━ 3s 13ms/step - loss: 6.1947e-04 - val_loss: 5.6383e-04
Epoch 26/50
147/147 ━━━━━━━━━━━━━━━━━━━━ 2s 14ms/step - loss: 6.1416e-04 - val_loss: 5.8954e-04
Epoch 27/50
147/147 ━━━━━━━━━━━━━━━━━━━━ 2s 15ms/step - loss: 5.3959e-04 - val_loss: 5.3227e-04
Epoch 28/50
147/147 ━━━━━━━━━━━━━━━━━━━━ 2s 13ms/step - loss: 5.3671e-04 - val_loss: 5.2313e-04
Epoch 29/50
147/147 ━━━━━━━━━━━━━━━━━━━━ 2s 12ms/step - loss: 4.4350e-04 - val_loss: 5.2080e-04
Epoch 30/50
147/147 ━━━━━━━━━━━━━━━━━━━━ 2s 13ms/step - loss: 4.8910e-04 - val_loss: 5.0422e-04
Epoch 31/50
147/147 ━━━━━━━━━━━━━━━━━━━━ 2s 12ms/step - loss: 4.8922e-04 - val_loss: 4.6540e-04
Epoch 32/50
147/147 ━━━━━━━━━━━━━━━━━━━━ 2s 13ms/step - loss: 5.0905e-04 - val_loss: 5.0049e-04
Epoch 33/50
147/147 ━━━━━━━━━━━━━━━━━━━━ 3s 17ms/step - loss: 4.7241e-04 - val_loss: 4.8364e-04
Epoch 34/50
147/147 ━━━━━━━━━━━━━━━━━━━━ 4s 13ms/step - loss: 4.6074e-04 - val_loss: 4.6975e-04
Epoch 35/50
147/147 ━━━━━━━━━━━━━━━━━━━━ 2s 12ms/step - loss: 4.5763e-04 - val_loss: 4.2442e-04
Epoch 36/50
147/147 ━━━━━━━━━━━━━━━━━━━━ 3s 13ms/step - loss: 4.4678e-04 - val_loss: 4.0820e-04
Epoch 37/50
147/147 ━━━━━━━━━━━━━━━━━━━━ 2s 14ms/step - loss: 4.2929e-04 - val_loss: 4.4113e-04
Epoch 38/50
147/147 ━━━━━━━━━━━━━━━━━━━━ 3s 17ms/step - loss: 4.0477e-04 - val_loss: 4.3271e-04
Epoch 39/50
147/147 ━━━━━━━━━━━━━━━━━━━━ 4s 13ms/step - loss: 4.0161e-04 - val_loss: 4.6158e-04
Epoch 40/50
147/147 ━━━━━━━━━━━━━━━━━━━━ 2s 12ms/step - loss: 4.5319e-04 - val_loss: 5.2616e-04
Epoch 41/50
147/147 ━━━━━━━━━━━━━━━━━━━━ 2s 12ms/step - loss: 4.5227e-04 - val_loss: 4.1394e-04
Epoch 42/50
147/147 ━━━━━━━━━━━━━━━━━━━━ 2s 14ms/step - loss: 4.0820e-04 - val_loss: 4.5397e-04
Epoch 43/50
147/147 ━━━━━━━━━━━━━━━━━━━━ 3s 17ms/step - loss: 4.0340e-04 - val_loss: 4.0213e-04
Epoch 44/50
147/147 ━━━━━━━━━━━━━━━━━━━━ 4s 12ms/step - loss: 3.8766e-04 - val_loss: 3.6890e-04
Epoch 45/50
147/147 ━━━━━━━━━━━━━━━━━━━━ 3s 13ms/step - loss: 3.7797e-04 - val_loss: 3.7869e-04
Epoch 46/50
147/147 ━━━━━━━━━━━━━━━━━━━━ 2s 12ms/step - loss: 3.6552e-04 - val_loss: 4.0109e-04
Epoch 47/50
147/147 ━━━━━━━━━━━━━━━━━━━━ 2s 16ms/step - loss: 3.4156e-04 - val_loss: 3.7577e-04
Epoch 48/50
147/147 ━━━━━━━━━━━━━━━━━━━━ 2s 13ms/step - loss: 3.5166e-04 - val_loss: 3.6536e-04
[illegible]
```

```
Epoch 49/50
147/147 ━━━━━━━━━━━━━━━━━━━━ 2s 13ms/step - loss: 3.4061e-04 - val_loss: 3.9649e-04
Epoch 50/50
147/147 ━━━━━━━━━━━━━━━━━━━━ 2s 12ms/step - loss: 3.4738e-04 - val_loss: 3.7799e-04
74/74 ━━━━━━━━━━━━━━━━━━━━ 1s 8ms/step
```

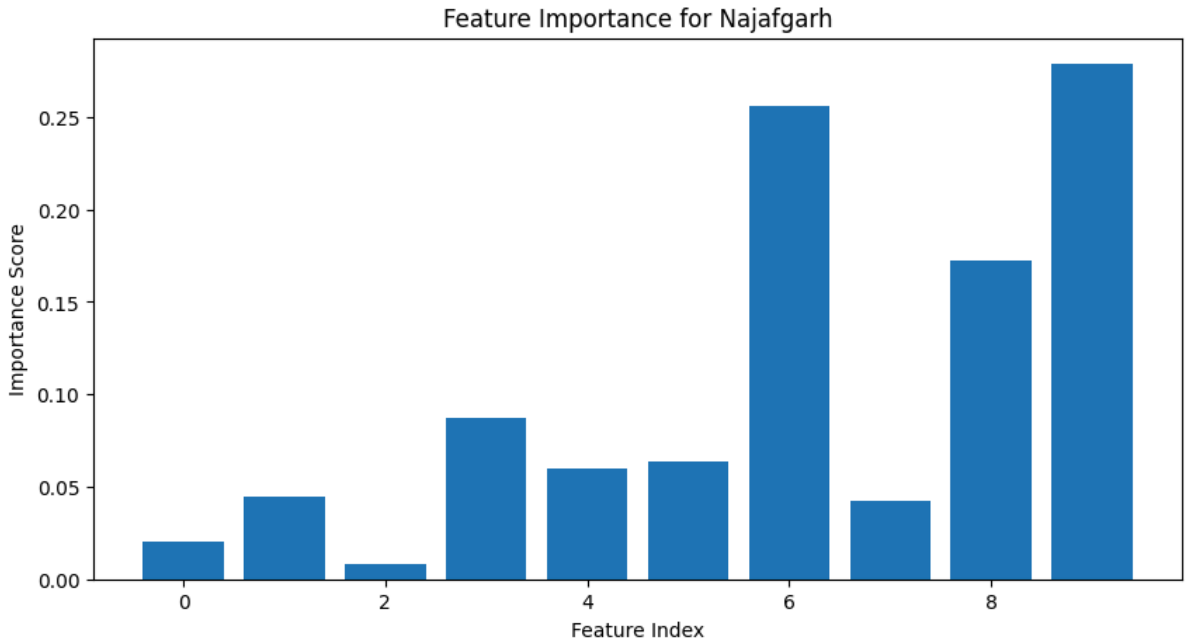

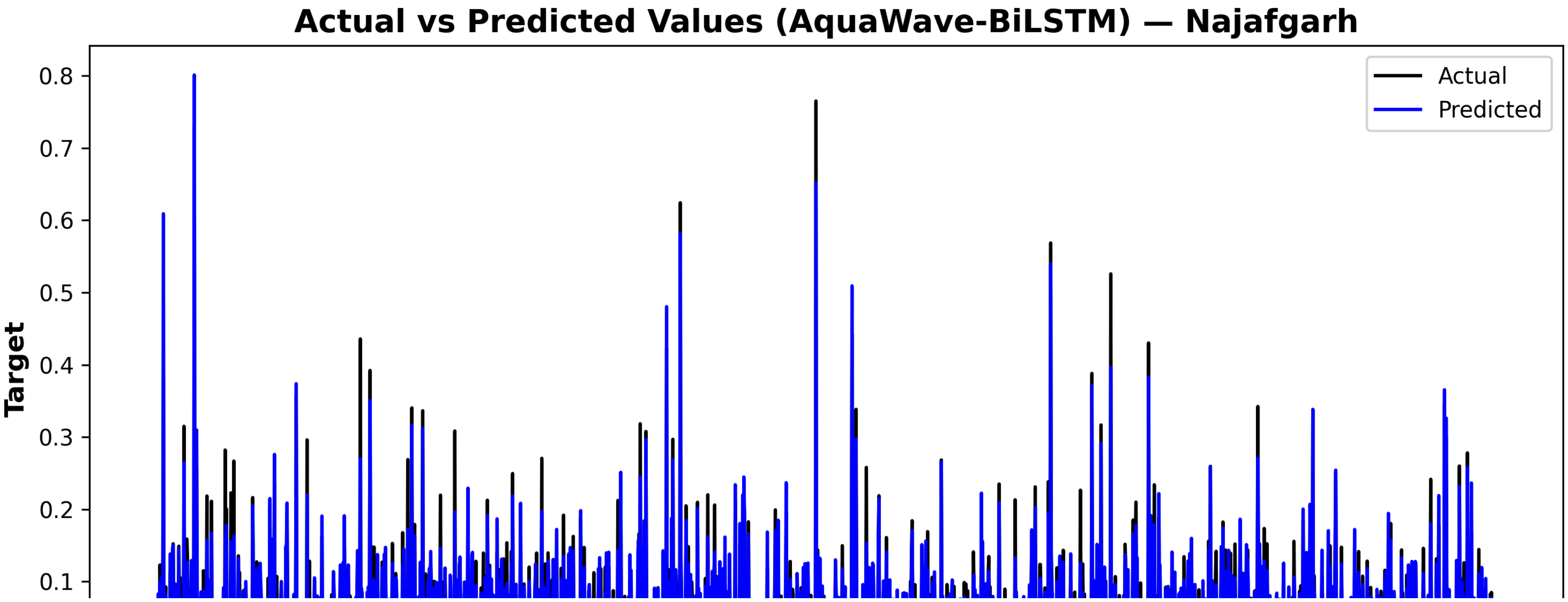

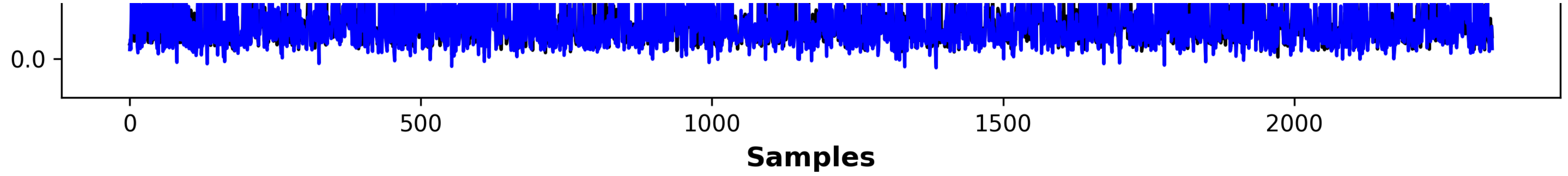


```
Processing Station: Okhla
/usr/local/lib/python3.11/dist-packages/pywt/_multilevel.py:43: UserWarning: Level value of 3 is too high: all coefficients will experience boundary effects.
  warnings.warn(
Epoch 1/50
/usr/local/lib/python3.11/dist-packages/keras/src/layers/rnn/rnn.py:200: UserWarning: Do not pass an `input_shape`/`input_dim` argument to a layer. When using Sequential mod
  super().__init__(**kwargs)
147/147 ━━━━━━━━━━━━━━━━━━━━ 6s 16ms/step - loss: 0.0106 - val_loss: 0.0066
Epoch 2/50
147/147 ━━━━━━━━━━━━━━━━━━━━ 2s 12ms/step - loss: 0.0060 - val_loss: 0.0034
Epoch 3/50
147/147 ━━━━━━━━━━━━━━━━━━━━ 3s 12ms/step - loss: 0.0033 - val_loss: 0.0024
Epoch 4/50
147/147 ━━━━━━━━━━━━━━━━━━━━ 3s 13ms/step - loss: 0.0023 - val_loss: 0.0019
Epoch 5/50
147/147 ━━━━━━━━━━━━━━━━━━━━ 3s 15ms/step - loss: 0.0020 - val_loss: 0.0019
Epoch 6/50
147/147 ━━━━━━━━━━━━━━━━━━━━ 2s 15ms/step - loss: 0.0017 - val_loss: 0.0016
Epoch 7/50
147/147 ━━━━━━━━━━━━━━━━━━━━ 2s 12ms/step - loss: 0.0017 - val_loss: 0.0014
Epoch 8/50
147/147 ━━━━━━━━━━━━━━━━━━━━ 3s 12ms/step - loss: 0.0016 - val_loss: 0.0012
Epoch 9/50
147/147 ━━━━━━━━━━━━━━━━━━━━ 2s 12ms/step - loss: 0.0013 - val_loss: 0.0011
Epoch 10/50
147/147 ━━━━━━━━━━━━━━━━━━━━ 3s 13ms/step - loss: 0.0013 - val_loss: 0.0012
Epoch 11/50
147/147 ━━━━━━━━━━━━━━━━━━━━ 3s 18ms/step - loss: 0.0012 - val_loss: 9.6053e-04
Epoch 12/50
147/147 ━━━━━━━━━━━━━━━━━━━━ 4s 13ms/step - loss: 0.0011 - val_loss: 0.0011
Epoch 13/50
147/147 ━━━━━━━━━━━━━━━━━━━━ 2s 12ms/step - loss: 0.0010 - val_loss: 9.0048e-04
Epoch 14/50
147/147 ━━━━━━━━━━━━━━━━━━━━ 3s 12ms/step - loss: 9.3276e-04 - val_loss: 8.6533e-04
Epoch 15/50
147/147 ━━━━━━━━━━━━━━━━━━━━ 3s 18ms/step - loss: 9.4585e-04 - val_loss: 8.2470e-04
Epoch 16/50
147/147 ━━━━━━━━━━━━━━━━━━━━ 2s 13ms/step - loss: 8.9604e-04 - val_loss: 7.9977e-04
Epoch 17/50
147/147 ━━━━━━━━━━━━━━━━━━━━ 2s 12ms/step - loss: 8.5644e-04 - val_loss: 8.4408e-04
Epoch 18/50
147/147 ━━━━━━━━━━━━━━━━━━━━ 3s 12ms/step - loss: 8.4209e-04 - val_loss: 8.4336e-04
Epoch 19/50
147/147 ━━━━━━━━━━━━━━━━━━━━ 3s 13ms/step - loss: 8.3249e-04 - val_loss: 7.5880e-04
Epoch 20/50
147/147 ━━━━━━━━━━━━━━━━━━━━ 3s 18ms/step - loss: 7.5692e-04 - val_loss: 7.2364e-04
Epoch 21/50
147/147 ━━━━━━━━━━━━━━━━━━━━ 2s 16ms/step - loss: 7.4803e-04 - val_loss: 7.2146e-04
Epoch 22/50
147/147 ━━━━━━━━━━━━━━━━━━━━ 2s 13ms/step - loss: 7.3577e-04 - val_loss: 7.8803e-04
Epoch 23/50
147/147 ━━━━━━━━━━━━━━━━━━━━ 2s 12ms/step - loss: 6.9856e-04 - val_loss: 8.4918e-04
```

```
Epoch 24/50
147/147 ━━━━━━━━━━━━━━━━━━━━ 3s 12ms/step - loss: 7.0563e-04 - val_loss: 7.3343e-04
Epoch 25/50
147/147 ━━━━━━━━━━━━━━━━━━━━ 2s 12ms/step - loss: 7.7629e-04 - val_loss: 7.7509e-04
Epoch 26/50
147/147 ━━━━━━━━━━━━━━━━━━━━ 2s 14ms/step - loss: 6.8019e-04 - val_loss: 7.8864e-04
Epoch 27/50
147/147 ━━━━━━━━━━━━━━━━━━━━ 3s 18ms/step - loss: 6.2399e-04 - val_loss: 8.0725e-04
Epoch 28/50
147/147 ━━━━━━━━━━━━━━━━━━━━ 4s 13ms/step - loss: 7.2396e-04 - val_loss: 6.5254e-04
Epoch 29/50
147/147 ━━━━━━━━━━━━━━━━━━━━ 3s 13ms/step - loss: 6.5074e-04 - val_loss: 6.4903e-04
Epoch 30/50
147/147 ━━━━━━━━━━━━━━━━━━━━ 2s 12ms/step - loss: 5.9552e-04 - val_loss: 6.8700e-04
Epoch 31/50
147/147 ━━━━━━━━━━━━━━━━━━━━ 2s 15ms/step - loss: 6.3768e-04 - val_loss: 7.2915e-04
Epoch 32/50
147/147 ━━━━━━━━━━━━━━━━━━━━ 2s 16ms/step - loss: 6.3757e-04 - val_loss: 6.5173e-04
Epoch 33/50
147/147 ━━━━━━━━━━━━━━━━━━━━ 2s 13ms/step - loss: 5.9498e-04 - val_loss: 6.6444e-04
Epoch 34/50
147/147 ━━━━━━━━━━━━━━━━━━━━ 2s 13ms/step - loss: 6.2480e-04 - val_loss: 5.9914e-04
Epoch 35/50
147/147 ━━━━━━━━━━━━━━━━━━━━ 2s 13ms/step - loss: 5.7592e-04 - val_loss: 6.1285e-04
Epoch 36/50
147/147 ━━━━━━━━━━━━━━━━━━━━ 3s 13ms/step - loss: 5.4019e-04 - val_loss: 5.9296e-04
Epoch 37/50
147/147 ━━━━━━━━━━━━━━━━━━━━ 3s 15ms/step - loss: 5.1815e-04 - val_loss: 6.2393e-04
Epoch 38/50
147/147 ━━━━━━━━━━━━━━━━━━━━ 2s 16ms/step - loss: 5.5353e-04 - val_loss: 6.1782e-04
Epoch 39/50
147/147 ━━━━━━━━━━━━━━━━━━━━ 2s 12ms/step - loss: 5.4445e-04 - val_loss: 6.0134e-04
Epoch 40/50
147/147 ━━━━━━━━━━━━━━━━━━━━ 3s 13ms/step - loss: 5.4823e-04 - val_loss: 5.8230e-04
Epoch 41/50
147/147 ━━━━━━━━━━━━━━━━━━━━ 2s 12ms/step - loss: 5.3239e-04 - val_loss: 5.6821e-04
Epoch 42/50
147/147 ━━━━━━━━━━━━━━━━━━━━ 3s 13ms/step - loss: 5.0806e-04 - val_loss: 5.9319e-04
Epoch 43/50
147/147 ━━━━━━━━━━━━━━━━━━━━ 2s 16ms/step - loss: 5.4348e-04 - val_loss: 5.8073e-04
Epoch 44/50
147/147 ━━━━━━━━━━━━━━━━━━━━ 2s 14ms/step - loss: 4.7125e-04 - val_loss: 6.0765e-04
Epoch 45/50
147/147 ━━━━━━━━━━━━━━━━━━━━ 2s 12ms/step - loss: 4.7239e-04 - val_loss: 6.1642e-04
Epoch 46/50
147/147 ━━━━━━━━━━━━━━━━━━━━ 3s 13ms/step - loss: 4.9720e-04 - val_loss: 5.6178e-04
Epoch 47/50
147/147 ━━━━━━━━━━━━━━━━━━━━ 2s 12ms/step - loss: 4.8311e-04 - val_loss: 7.1889e-04
Epoch 48/50
147/147 ━━━━━━━━━━━━━━━━━━━━ 3s 16ms/step - loss: 5.2474e-04 - val_loss: 5.3711e-04
Epoch 49/50
147/147 ━━━━━━━━━━━━━━━━━━━━ 2s 15ms/step - loss: 4.6240e-04 - val_loss: 5.8692e-04
Epoch 50/50
147/147 ━━━━━━━━━━━━━━━━━━━━ 2s 12ms/step - loss: 4.8067e-04 - val_loss: 5.8258e-04
74/74 ━━━━━━━━━━━━━━━━━━━━ 1s 8ms/step
```

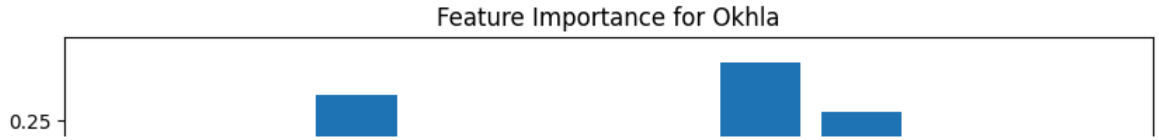

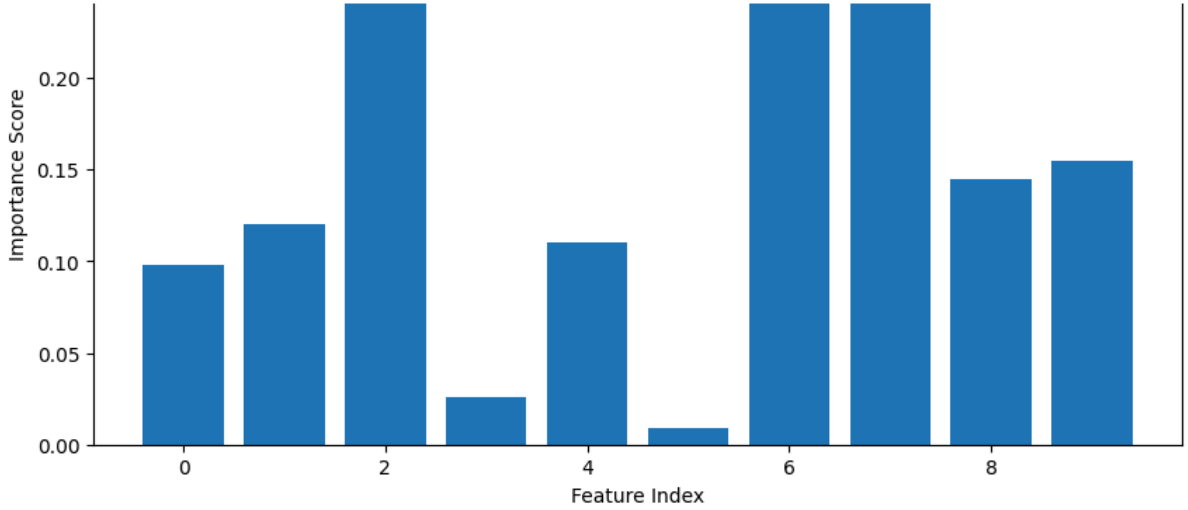

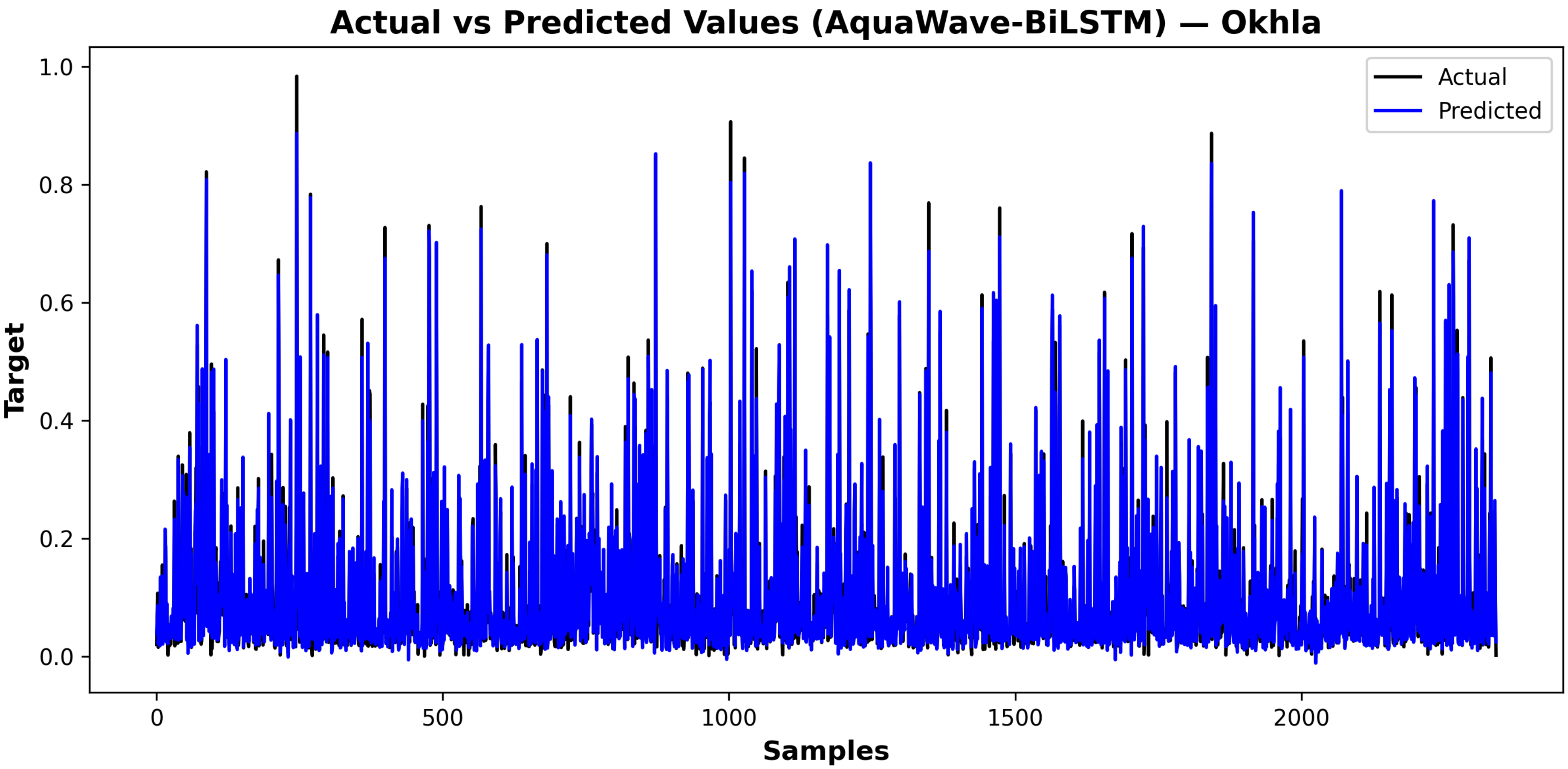


```
Final Model Evaluation Across Stations:
```

```
Station: AshokVihar
MSE: 0.0006
MAE: 0.0155
RMSE: 0.0247
R² Score: 0.9441

Station: DCStadium
MSE: 0.0008
MAE: 0.0184
RMSE: 0.0286
R² Score: 0.9840

Station: DwarkaSec8
MSE: 0.0008
MAE: 0.0190
RMSE: 0.0284
R² Score: 0.9359

Station: NehruNagar
MSE: 0.0006
MAE: 0.0172
RMSE: 0.0239
R² Score: 0.9816

Station: Najafgarh
MSE: 0.0004
MAE: 0.0129
RMSE: 0.0194
R² Score: 0.8848

Station: Okhla
MSE: 0.0006
MAE: 0.0153
RMSE: 0.0241
R² Score: 0.9655
```

```
from PIL import Image

# Open the image you already have
img = Image.open("/content/Picture22.png")

# Save it at higher DPI
img.save("22_HighDPI.png", dpi=(600,600))  # or dpi=(300,300))
```

```
Start coding or generate with AI.
```

SHAP visualizations and interpretability analysis related to PM2.5 prediction.

```
import numpy as np
import pandas as pd
import matplotlib.pyplot as plt
import os
import pywt
import shap

from sklearn.decomposition import PCA
from sklearn.preprocessing import StandardScaler, MinMaxScaler
from sklearn.model_selection import train_test_split
from sklearn.metrics import mean_squared_error, mean_absolute_error, r2_score
from sklearn.ensemble import RandomForestRegressor

import tensorflow as tf
from tensorflow.keras.models import Sequential
from tensorflow.keras.layers import LSTM, Dense, Bidirectional

# ============================================
# Hybrid AOAOA Optimizer
# ============================================
class HybridOptimizer:
    def __init__(self, objective_function, lower_bound, upper_bound, population_size, iterations):
        self.objective_function = objective_function
        self.lower_bound = np.array(lower_bound)
        self.upper_bound = np.array(upper_bound)
        self.population_size = population_size
        self.iterations = iterations
        self.population = np.random.uniform(low=self.lower_bound, high=self.upper_bound, size=(population_size, len(lower_bound)))
        self.best_solution = None
        self.best_fitness = float('inf')

    def optimize(self):
        for _ in range(self.iterations):
            for i in range(self.population_size):
                perturbation = np.random.uniform(-0.1, 0.1, size=self.population.shape[1])
                candidate_solution_aquila = self.population[i] + perturbation
                candidate_solution_aquila = np.clip(candidate_solution_aquila, self.lower_bound, self.upper_bound)
                fitness_aquila = self.objective_function(candidate_solution_aquila)

                if fitness_aquila < self.best_fitness:
```

```
                    self.best_fitness = fitness_aquila
                    self.best_solution = candidate_solution_aquila

            for i in range(self.population_size):
                partner_idx = np.random.randint(self.population_size)
                partner = self.population[partner_idx]
                candidate_solution_arithmetic = (self.population[i] + partner) / 2
                candidate_solution_arithmetic = np.clip(candidate_solution_arithmetic, self.lower_bound, self.upper_bound)
                fitness_arithmetic = self.objective_function(candidate_solution_arithmetic)

                if fitness_arithmetic < self.best_fitness:
                    self.best_fitness = fitness_arithmetic
                    self.best_solution = candidate_solution_arithmetic

        return self.best_solution

# ============================================
# Feature Extraction
# ============================================
def extract_wavelet_features(X, wavelet='db4', level=3, num_features=50):
    features = []
    for sample in X:
        coeffs = pywt.wavedec(sample, wavelet, level=level)
        flattened_coeffs = np.concatenate([c.flatten() for c in coeffs])
        features.append(flattened_coeffs[:num_features])
    return np.array(features)

def apply_pca(X, n_components=10):
    scaler = StandardScaler()
    X_scaled = scaler.fit_transform(X)
    pca = PCA(n_components=n_components)
    return pca.fit_transform(X_scaled)

def extract_combined_features(X):
    X_wavelet = extract_wavelet_features(X)
    X_pca = apply_pca(X_wavelet)
    return X_pca

# ============================================
# Bi-LSTM Model
# ============================================
def build_lstm_model(input_shape):
    model = Sequential([
        Bidirectional(LSTM(50, return_sequences=True, input_shape=input_shape)),
        Bidirectional(LSTM(50, return_sequences=False)),
        Dense(1)
    ])
    model.compile(optimizer='adam', loss='mean_squared_error')
    return model

# ============================================
# Training & Evaluation
# ============================================
def evaluate_model(X, y):
    if X.shape[1] == 0:
        [illegible]
```

```
        raise ValueError("No features selected! Adjust AOAOA feature selection.")
    X_train, X_test, y_train, y_test = train_test_split(X, y, test_size=0.2, random_state=42)
    X_train = X_train.reshape(X_train.shape[0], X_train.shape[1], 1)
    X_test = X_test.reshape(X_test.shape[0], X_test.shape[1], 1)

    model = build_lstm_model((X_train.shape[1], 1))
    model.fit(X_train, y_train, epochs=50, batch_size=64, validation_data=(X_test, y_test), verbose=1)
    y_pred = model.predict(X_test)

    mse = mean_squared_error(y_test, y_pred)
    mae = mean_absolute_error(y_test, y_pred)
    rmse = np.sqrt(mse)
    r2 = r2_score(y_test, y_pred)

    return mse, mae, rmse, r2, y_test, y_pred

# ============================================
# Run Across All Stations
# ============================================
stations = {
    'AshokVihar': '/content/AshokVihar_Hourly.csv',
    'DCStadium': '/content/DCStadium_Hourly.csv',
    'DwarkaSec8': '/content/DwarkaSec8_Hourly.csv',
    'NehruNagar': '/content/NehruNagar_Hourly.csv',
    'Najafgarh': '/content/Najafgarh_Hourly.csv',
    'Okhla': '/content/Okhla_Hourly.csv'
}

threshold = 0.40
results = {}
os.makedirs("shap_plots", exist_ok=True)

for station, file_path in stations.items():
    print(f"\nProcessing Station: {station}")

    df = pd.read_csv(file_path)

    scaler = MinMaxScaler()
    X_full = scaler.fit_transform(df.iloc[:, :-1].values)
    y = scaler.fit_transform(df.iloc[:, -1].values.reshape(-1, 1)).ravel()

    # Feature Extraction
    X_extracted = extract_combined_features(X_full)

    # Feature Selection with Hybrid AOAOA
    objective_function = lambda x: np.sum(x**2)
    hybrid_optimizer = HybridOptimizer(objective_function, lower_bound=[-1]*X_extracted.shape[1],
                                       upper_bound=[1]*X_extracted.shape[1],
                                       population_size=50, iterations=100)
    selected_features = hybrid_optimizer.optimize()

    X_selected = X_extracted[:, selected_features > threshold]
    if X_selected.shape[1] > 0:
        X_final = X_selected
        selected_feature_indices = np.where(selected_features > threshold)[0]
    else:
```

```
            X_final = X_extracted
            selected_feature_indices = np.arange(X_extracted.shape[1])

        # Dummy feature names for components (post PCA)
        selected_feature_names = [f"Feature {i+1}" for i in range(X_final.shape[1])]
        print(f"Selected features ({len(selected_feature_names)}): {selected_feature_names}")

        # Final Model Evaluation
        mse, mae, rmse, r2, y_test, y_pred = evaluate_model(X_final, y)

        results[station] = {"MSE": mse, "MAE": mae, "RMSE": rmse, "R² Score": r2}

        # AOAOA Feature Importance Plot
        plt.figure(figsize=(10, 5))
        feature_importance = np.abs(selected_features)
        plt.bar(range(len(feature_importance)), feature_importance)
        plt.xlabel('Feature Index')
        plt.ylabel('Importance Score')
        plt.title(f'Feature Importance (AOAOA) - {station}')
        plt.tight_layout()
        plt.savefig(f"shap_plots/{station}_AOAOA_FeatureImportance.png")
        plt.close()

        # SHAP with RF surrogate
        print(f"🔷 Computing SHAP for {station}...")
        rf = RandomForestRegressor(n_estimators=100, random_state=42)
        rf.fit(X_final, y)

        explainer = shap.Explainer(rf, X_final)
        shap_values = explainer(X_final, check_additivity=False)

        # SHAP Summary Plot (interactive, high DPI)
        plt.figure(dpi=300)
        plt.title(f"SHAP Feature Importance - {station}", fontsize=14, fontweight='bold')
        shap.summary_plot(
            shap_values,
            X_final,
            feature_names=selected_feature_names,
            show=True
        )


        # Actual vs Predicted Plot
        plt.figure(figsize=(10, 5))
        plt.plot(y_test, label="Actual", color='black')
        plt.plot(y_pred, label="Predicted", color='blue')
        plt.xlabel('Samples')
        plt.ylabel('PM2.5')
        plt.title(f'Actual vs Predicted (Bi-LSTM) - {station}')
        plt.legend()
        plt.tight_layout()
        plt.savefig(f"shap_plots/{station}_Actual_vs_Predicted.png")
        plt.close()

    # ============================================
    # Results Summary
```

```
    # Results Summary
    # ============================================
    print("\nFinal Model Evaluation Across Stations:")
    for station, metrics in results.items():
        print(f"\nStation: {station}")
        for metric, value in metrics.items():
            print(f"{metric}: {value:.4f}")
```

```
Processing Station: AshokVihar
/usr/local/lib/python3.11/dist-packages/pywt/_multilevel.py:43: UserWarning: Level value of 3 is too high: all coefficients will experience boundary effects.
  warnings.warn(
Selected features (10): ['Feature 1', 'Feature 2', 'Feature 3', 'Feature 4', 'Feature 5', 'Feature 6', 'Feature 7', 'Feature 8', 'Feature 9', 'Feature 10']
Epoch 1/50
/usr/local/lib/python3.11/dist-packages/keras/src/layers/rnn/rnn.py:200: UserWarning: Do not pass an `input_shape`/`input_dim` argument to a layer. When using Sequential mod
  super().__init__(**kwargs)
147/147 ━━━━━━━━━━━━━━━━━━━━ 7s 15ms/step - loss: 0.0054 - val_loss: 0.0027
Epoch 2/50
147/147 ━━━━━━━━━━━━━━━━━━━━ 1s 9ms/step - loss: 0.0026 - val_loss: 0.0027
Epoch 3/50
147/147 ━━━━━━━━━━━━━━━━━━━━ 1s 9ms/step - loss: 0.0024 - val_loss: 0.0018
Epoch 4/50
147/147 ━━━━━━━━━━━━━━━━━━━━ 3s 9ms/step - loss: 0.0016 - val_loss: 0.0021
Epoch 5/50
147/147 ━━━━━━━━━━━━━━━━━━━━ 3s 12ms/step - loss: 0.0016 - val_loss: 0.0016
Epoch 6/50
147/147 ━━━━━━━━━━━━━━━━━━━━ 2s 12ms/step - loss: 0.0017 - val_loss: 0.0014
Epoch 7/50
147/147 ━━━━━━━━━━━━━━━━━━━━ 1s 9ms/step - loss: 0.0014 - val_loss: 0.0013
Epoch 8/50
147/147 ━━━━━━━━━━━━━━━━━━━━ 1s 9ms/step - loss: 0.0013 - val_loss: 0.0012
Epoch 9/50
147/147 ━━━━━━━━━━━━━━━━━━━━ 3s 10ms/step - loss: 0.0013 - val_loss: 0.0012
Epoch 10/50
147/147 ━━━━━━━━━━━━━━━━━━━━ 1s 9ms/step - loss: 0.0013 - val_loss: 0.0011
Epoch 11/50
147/147 ━━━━━━━━━━━━━━━━━━━━ 1s 9ms/step - loss: 0.0012 - val_loss: 0.0011
Epoch 12/50
147/147 ━━━━━━━━━━━━━━━━━━━━ 2s 11ms/step - loss: 0.0012 - val_loss: 0.0012
Epoch 13/50
147/147 ━━━━━━━━━━━━━━━━━━━━ 2s 9ms/step - loss: 0.0011 - val_loss: 0.0011
Epoch 14/50
147/147 ━━━━━━━━━━━━━━━━━━━━ 1s 9ms/step - loss: 0.0011 - val_loss: 0.0011
Epoch 15/50
147/147 ━━━━━━━━━━━━━━━━━━━━ 1s 9ms/step - loss: 0.0011 - val_loss: 0.0011
Epoch 16/50
147/147 ━━━━━━━━━━━━━━━━━━━━ 3s 10ms/step - loss: 0.0011 - val_loss: 9.7906e-04
Epoch 17/50
147/147 ━━━━━━━━━━━━━━━━━━━━ 2s 9ms/step - loss: 9.5802e-04 - val_loss: 0.0010
Epoch 18/50
147/147 ━━━━━━━━━━━━━━━━━━━━ 3s 14ms/step - loss: 0.0010 - val_loss: 9.5495e-04
Epoch 19/50
147/147 ━━━━━━━━━━━━━━━━━━━━ 1s 9ms/step - loss: 9.8499e-04 - val_loss: 8.9628e-04
Epoch 20/50
147/147 ━━━━━━━━━━━━━━━━━━━━ 3s 9ms/step - loss: 9.6969e-04 - val_loss: 8.8573e-04
Epoch 21/50
147/147 ━━━━━━━━━━━━━━━━━━━━ 3s 9ms/step - loss: 8.7529e-04 - val_loss: 9.3434e-04
Epoch 22/50
147/147 ━━━━━━━━━━━━━━━━━━━━ 1s 9ms/step - loss: 8.7942e-04 - val_loss: 9.2282e-04
Epoch 23/50
147/147 ━━━━━━━━━━━━━━━━━━━━ 3s 12ms/step - loss: 8.1179e-04 - val_loss: 8.6923e-04
Epoch 24/50
147/147 ━━━━━━━━━━━━━━━━━━━━ 2s 10ms/step - loss: 8.4947e-04 - val_loss: 8.8595e-04
Epoch 25/50
147/147 ━━━━━━━━━━━━━━━━━━━━ 2s 9ms/step - loss: 8.1588e-04 - val_loss: 9.0769e-04
Epoch 26/50
147/147 ━━━━━━━━━━━━━━━━━━━━ 3s 9ms/step - loss: 7.7480e-04 - val_loss: 9.0716e-04
Epoch 27/50
[illegible]
```

```
147/147 ━━━━━━━━━━━━━━━━━━━━ 3s 10ms/step - loss: 7.0204e-04 - val_loss: 9.1465e-04
Epoch 28/50
147/147 ━━━━━━━━━━━━━━━━━━━━ 2s 12ms/step - loss: 7.3676e-04 - val_loss: 8.3192e-04
Epoch 29/50
147/147 ━━━━━━━━━━━━━━━━━━━━ 2s 10ms/step - loss: 7.1970e-04 - val_loss: 7.5018e-04
Epoch 30/50
147/147 ━━━━━━━━━━━━━━━━━━━━ 2s 9ms/step - loss: 6.8546e-04 - val_loss: 7.2944e-04
Epoch 31/50
147/147 ━━━━━━━━━━━━━━━━━━━━ 2s 10ms/step - loss: 6.0820e-04 - val_loss: 7.4848e-04
Epoch 32/50
147/147 ━━━━━━━━━━━━━━━━━━━━ 2s 9ms/step - loss: 6.2384e-04 - val_loss: 7.4618e-04
Epoch 33/50
147/147 ━━━━━━━━━━━━━━━━━━━━ 1s 9ms/step - loss: 6.6273e-04 - val_loss: 7.7665e-04
Epoch 34/50
147/147 ━━━━━━━━━━━━━━━━━━━━ 2s 11ms/step - loss: 6.4276e-04 - val_loss: 7.4428e-04
Epoch 35/50
147/147 ━━━━━━━━━━━━━━━━━━━━ 2s 9ms/step - loss: 6.0953e-04 - val_loss: 7.7169e-04
Epoch 36/50
147/147 ━━━━━━━━━━━━━━━━━━━━ 3s 9ms/step - loss: 5.7399e-04 - val_loss: 7.0597e-04
Epoch 37/50
147/147 ━━━━━━━━━━━━━━━━━━━━ 1s 9ms/step - loss: 5.3942e-04 - val_loss: 6.7751e-04
Epoch 38/50
147/147 ━━━━━━━━━━━━━━━━━━━━ 3s 9ms/step - loss: 5.3503e-04 - val_loss: 6.5636e-04
Epoch 39/50
147/147 ━━━━━━━━━━━━━━━━━━━━ 1s 9ms/step - loss: 5.8092e-04 - val_loss: 7.8981e-04
Epoch 40/50
147/147 ━━━━━━━━━━━━━━━━━━━━ 3s 12ms/step - loss: 5.6306e-04 - val_loss: 6.8309e-04
Epoch 41/50
147/147 ━━━━━━━━━━━━━━━━━━━━ 2s 10ms/step - loss: 5.0637e-04 - val_loss: 6.4545e-04
Epoch 42/50
147/147 ━━━━━━━━━━━━━━━━━━━━ 2s 9ms/step - loss: 4.9104e-04 - val_loss: 6.7514e-04
Epoch 43/50
147/147 ━━━━━━━━━━━━━━━━━━━━ 3s 9ms/step - loss: 4.7904e-04 - val_loss: 6.8276e-04
Epoch 44/50
147/147 ━━━━━━━━━━━━━━━━━━━━ 2s 9ms/step - loss: 4.7268e-04 - val_loss: 6.2833e-04
Epoch 45/50
147/147 ━━━━━━━━━━━━━━━━━━━━ 2s 12ms/step - loss: 4.8374e-04 - val_loss: 6.4740e-04
Epoch 46/50
147/147 ━━━━━━━━━━━━━━━━━━━━ 2s 9ms/step - loss: 4.4524e-04 - val_loss: 6.3471e-04
Epoch 47/50
147/147 ━━━━━━━━━━━━━━━━━━━━ 1s 9ms/step - loss: 4.5730e-04 - val_loss: 6.4952e-04
Epoch 48/50
147/147 ━━━━━━━━━━━━━━━━━━━━ 3s 9ms/step - loss: 4.4881e-04 - val_loss: 6.2541e-04
Epoch 49/50
147/147 ━━━━━━━━━━━━━━━━━━━━ 1s 9ms/step - loss: 4.4152e-04 - val_loss: 6.3631e-04
Epoch 50/50
147/147 ━━━━━━━━━━━━━━━━━━━━ 3s 9ms/step - loss: 4.4634e-04 - val_loss: 5.9634e-04
74/74 ━━━━━━━━━━━━━━━━━━━━ 1s 10ms/step
🔷 Computing SHAP for AshokVihar...
100%|===================| 11698/11705 [17:07<00:00]
```

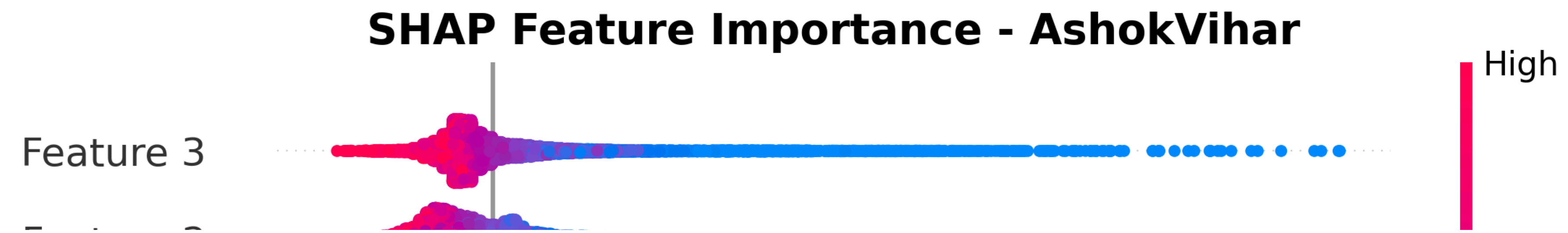

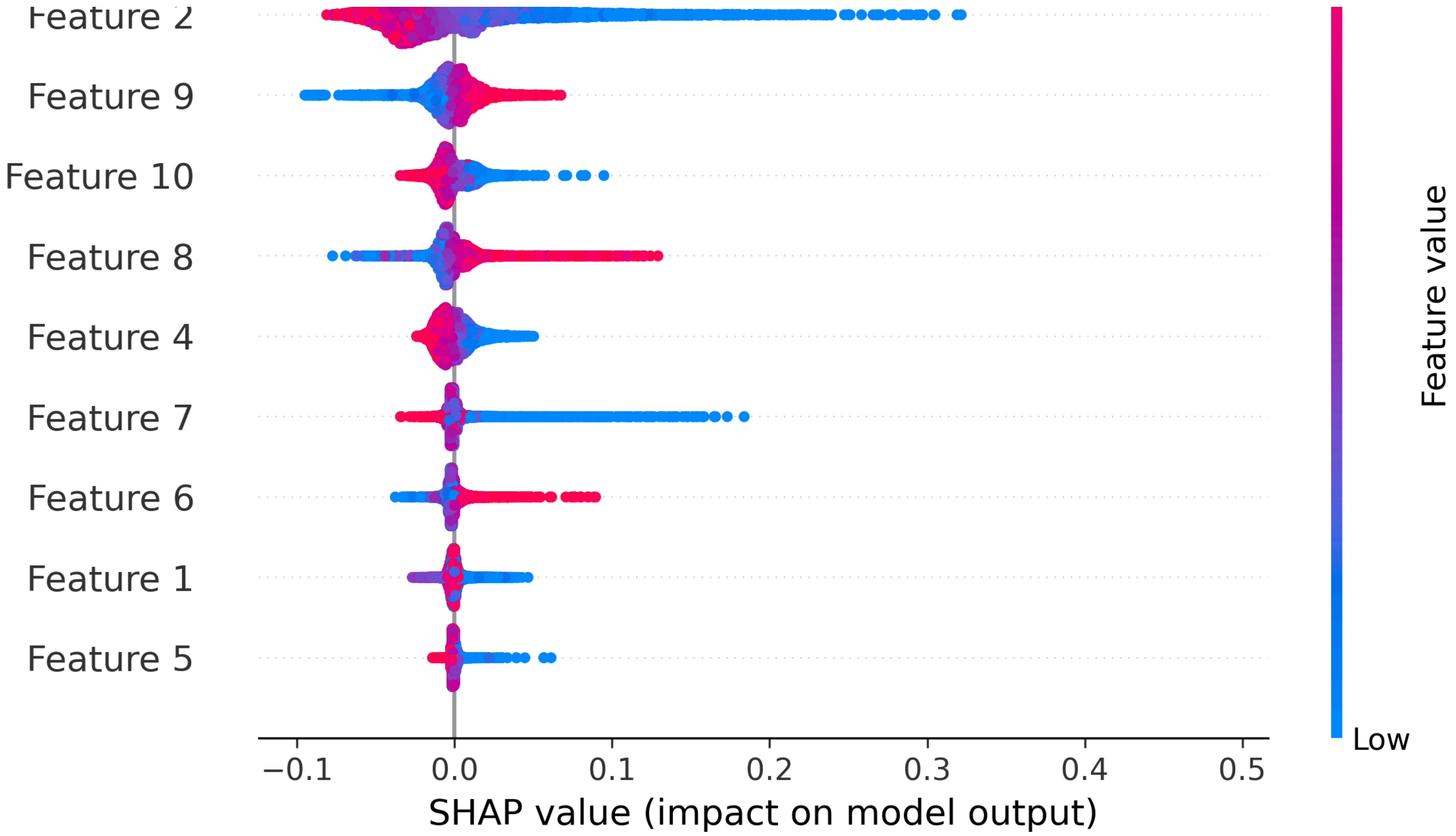


```
Processing Station: DCStadium
/usr/local/lib/python3.11/dist-packages/pywt/_multilevel.py:43: UserWarning: Level value of 3 is too high: all coefficients will experience boundary effects.
  warnings.warn(
Selected features (10): ['Feature 1', 'Feature 2', 'Feature 3', 'Feature 4', 'Feature 5', 'Feature 6', 'Feature 7', 'Feature 8', 'Feature 9', 'Feature 10']
Epoch 1/50
/usr/local/lib/python3.11/dist-packages/keras/src/layers/rnn/rnn.py:200: UserWarning: Do not pass an `input_shape`/`input_dim` argument to a layer. When using Sequential mod
  super().__init__(**kwargs)
147/147 ━━━━━━━━━━━━━━━━━━━━ 5s 12ms/step - loss: 0.0242 - val_loss: 0.0099
Epoch 2/50
147/147 ━━━━━━━━━━━━━━━━━━━━ 2s 10ms/step - loss: 0.0077 - val_loss: 0.0029
Epoch 3/50
147/147 ━━━━━━━━━━━━━━━━━━━━ 2s 14ms/step - loss: 0.0029 - val_loss: 0.0023
Epoch 4/50
147/147 ━━━━━━━━━━━━━━━━━━━━ 1s 9ms/step - loss: 0.0024 - val_loss: 0.0023
Epoch 5/50
147/147 ━━━━━━━━━━━━━━━━━━━━ 3s 10ms/step - loss: 0.0022 - val_loss: 0.0027
[illegible]
```

```
Epoch 6/50
147/147 ━━━━━━━━━━━━━━━━━━━━ 3s 9ms/step - loss: 0.0023 - val_loss: 0.0020
Epoch 7/50
147/147 ━━━━━━━━━━━━━━━━━━━━ 1s 9ms/step - loss: 0.0021 - val_loss: 0.0021
Epoch 8/50
147/147 ━━━━━━━━━━━━━━━━━━━━ 1s 9ms/step - loss: 0.0020 - val_loss: 0.0021
Epoch 9/50
147/147 ━━━━━━━━━━━━━━━━━━━━ 2s 12ms/step - loss: 0.0019 - val_loss: 0.0017
Epoch 10/50
147/147 ━━━━━━━━━━━━━━━━━━━━ 2s 10ms/step - loss: 0.0019 - val_loss: 0.0017
Epoch 11/50
147/147 ━━━━━━━━━━━━━━━━━━━━ 3s 9ms/step - loss: 0.0018 - val_loss: 0.0016
Epoch 12/50
147/147 ━━━━━━━━━━━━━━━━━━━━ 3s 10ms/step - loss: 0.0018 - val_loss: 0.0016
Epoch 13/50
147/147 ━━━━━━━━━━━━━━━━━━━━ 2s 9ms/step - loss: 0.0017 - val_loss: 0.0017
Epoch 14/50
147/147 ━━━━━━━━━━━━━━━━━━━━ 2s 11ms/step - loss: 0.0018 - val_loss: 0.0019
Epoch 15/50
147/147 ━━━━━━━━━━━━━━━━━━━━ 2s 13ms/step - loss: 0.0016 - val_loss: 0.0015
Epoch 16/50
147/147 ━━━━━━━━━━━━━━━━━━━━ 2s 9ms/step - loss: 0.0017 - val_loss: 0.0015
Epoch 17/50
147/147 ━━━━━━━━━━━━━━━━━━━━ 3s 9ms/step - loss: 0.0016 - val_loss: 0.0018
Epoch 18/50
147/147 ━━━━━━━━━━━━━━━━━━━━ 1s 9ms/step - loss: 0.0015 - val_loss: 0.0014
Epoch 19/50
147/147 ━━━━━━━━━━━━━━━━━━━━ 3s 10ms/step - loss: 0.0014 - val_loss: 0.0013
Epoch 20/50
147/147 ━━━━━━━━━━━━━━━━━━━━ 3s 14ms/step - loss: 0.0014 - val_loss: 0.0013
Epoch 21/50
147/147 ━━━━━━━━━━━━━━━━━━━━ 2s 9ms/step - loss: 0.0014 - val_loss: 0.0014
Epoch 22/50
147/147 ━━━━━━━━━━━━━━━━━━━━ 3s 9ms/step - loss: 0.0014 - val_loss: 0.0012
Epoch 23/50
147/147 ━━━━━━━━━━━━━━━━━━━━ 3s 10ms/step - loss: 0.0012 - val_loss: 0.0012
Epoch 24/50
147/147 ━━━━━━━━━━━━━━━━━━━━ 2s 10ms/step - loss: 0.0013 - val_loss: 0.0012
Epoch 25/50
147/147 ━━━━━━━━━━━━━━━━━━━━ 3s 13ms/step - loss: 0.0012 - val_loss: 0.0012
Epoch 26/50
147/147 ━━━━━━━━━━━━━━━━━━━━ 2s 9ms/step - loss: 0.0011 - val_loss: 0.0012
Epoch 27/50
147/147 ━━━━━━━━━━━━━━━━━━━━ 1s 9ms/step - loss: 0.0012 - val_loss: 0.0012
Epoch 28/50
147/147 ━━━━━━━━━━━━━━━━━━━━ 3s 9ms/step - loss: 0.0011 - val_loss: 0.0013
Epoch 29/50
147/147 ━━━━━━━━━━━━━━━━━━━━ 1s 9ms/step - loss: 0.0011 - val_loss: 0.0011
Epoch 30/50
147/147 ━━━━━━━━━━━━━━━━━━━━ 1s 9ms/step - loss: 9.9729e-04 - val_loss: 0.0012
Epoch 31/50
147/147 ━━━━━━━━━━━━━━━━━━━━ 3s 13ms/step - loss: 0.0011 - val_loss: 0.0011
Epoch 32/50
147/147 ━━━━━━━━━━━━━━━━━━━━ 2s 11ms/step - loss: 0.0010 - val_loss: 0.0012
Epoch 33/50
147/147 ━━━━━━━━━━━━━━━━━━━━ 1s 9ms/step - loss: 0.0010 - val_loss: 0.0012
Epoch 34/50
147/147 ━━━━━━━━━━━━━━━━━━━━ 3s 9ms/step - loss: 9.9673e-04 - val_loss: 0.0011
Epoch 35/50
147/147 ━━━━━━━━━━━━━━━━━━━━ 1s 9ms/step - loss: 9.6557e-04 - val_loss: 0.0011
Epoch 36/50
```

```
147/147 ━━━━━━━━━━━━━━━━━━━━ 3s 9ms/step - loss: 9.4367e-04 - val_loss: 0.0010
Epoch 37/50
147/147 ━━━━━━━━━━━━━━━━━━━━ 1s 10ms/step - loss: 8.7678e-04 - val_loss: 0.0011
Epoch 38/50
147/147 ━━━━━━━━━━━━━━━━━━━━ 2s 14ms/step - loss: 9.3268e-04 - val_loss: 0.0011
Epoch 39/50
147/147 ━━━━━━━━━━━━━━━━━━━━ 2s 9ms/step - loss: 9.0294e-04 - val_loss: 9.5952e-04
Epoch 40/50
147/147 ━━━━━━━━━━━━━━━━━━━━ 1s 9ms/step - loss: 9.5064e-04 - val_loss: 0.0011
Epoch 41/50
147/147 ━━━━━━━━━━━━━━━━━━━━ 3s 10ms/step - loss: 9.1058e-04 - val_loss: 9.8161e-04
Epoch 42/50
147/147 ━━━━━━━━━━━━━━━━━━━━ 3s 10ms/step - loss: 8.4398e-04 - val_loss: 0.0010
Epoch 43/50
147/147 ━━━━━━━━━━━━━━━━━━━━ 3s 12ms/step - loss: 8.7071e-04 - val_loss: 9.0153e-04
Epoch 44/50
147/147 ━━━━━━━━━━━━━━━━━━━━ 2s 11ms/step - loss: 8.5684e-04 - val_loss: 9.5657e-04
Epoch 45/50
147/147 ━━━━━━━━━━━━━━━━━━━━ 1s 9ms/step - loss: 8.4166e-04 - val_loss: 9.0994e-04
Epoch 46/50
147/147 ━━━━━━━━━━━━━━━━━━━━ 1s 9ms/step - loss: 8.2849e-04 - val_loss: 8.8332e-04
Epoch 47/50
147/147 ━━━━━━━━━━━━━━━━━━━━ 3s 9ms/step - loss: 8.5811e-04 - val_loss: 9.0370e-04
Epoch 48/50
147/147 ━━━━━━━━━━━━━━━━━━━━ 1s 9ms/step - loss: 8.3572e-04 - val_loss: 8.6717e-04
Epoch 49/50
147/147 ━━━━━━━━━━━━━━━━━━━━ 1s 9ms/step - loss: 8.2878e-04 - val_loss: 9.4465e-04
Epoch 50/50
147/147 ━━━━━━━━━━━━━━━━━━━━ 2s 11ms/step - loss: 7.7092e-04 - val_loss: 9.6039e-04
74/74 ━━━━━━━━━━━━━━━━━━━━ 1s 10ms/step
🔷 Computing SHAP for DCStadium...
100%|===================| 11692/11704 [15:33<00:00]
```

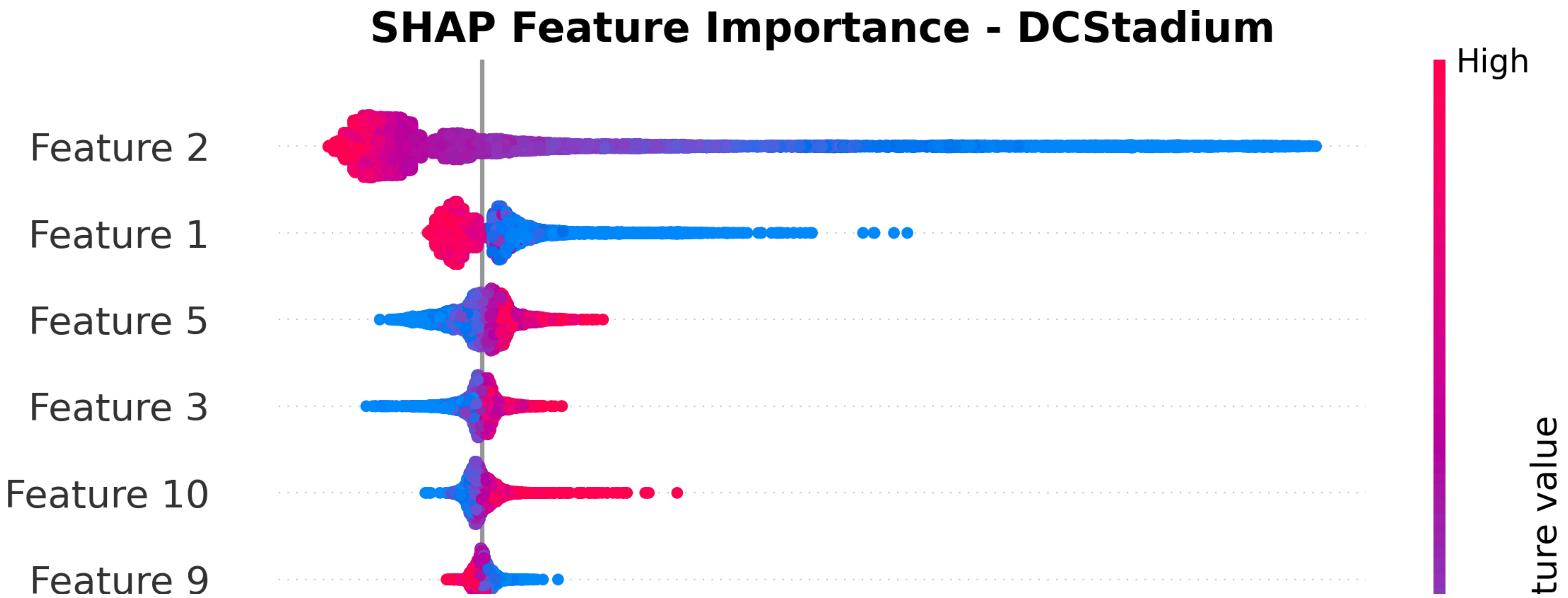

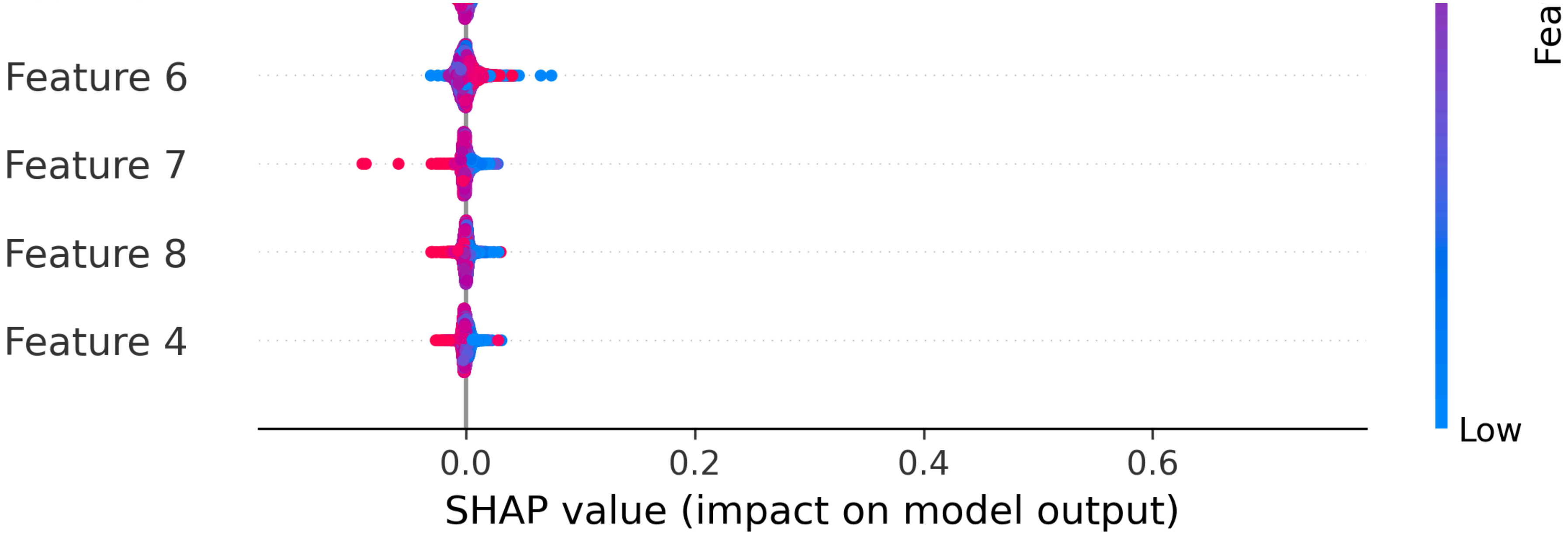


```
Processing Station: DwarkaSec8
/usr/local/lib/python3.11/dist-packages/pywt/_multilevel.py:43: UserWarning: Level value of 3 is too high: all coefficients will experience boundary effects.
  warnings.warn(
Selected features (10): ['Feature 1', 'Feature 2', 'Feature 3', 'Feature 4', 'Feature 5', 'Feature 6', 'Feature 7', 'Feature 8', 'Feature 9', 'Feature 10']
Epoch 1/50
/usr/local/lib/python3.11/dist-packages/keras/src/layers/rnn/rnn.py:200: UserWarning: Do not pass an `input_shape`/`input_dim` argument to a layer. When using Sequential mod
  super().__init__(**kwargs)
147/147 ━━━━━━━━━━━━━━━━━━━━ 5s 13ms/step - loss: 0.0079 - val_loss: 0.0041
Epoch 2/50
147/147 ━━━━━━━━━━━━━━━━━━━━ 2s 12ms/step - loss: 0.0032 - val_loss: 0.0023
Epoch 3/50
147/147 ━━━━━━━━━━━━━━━━━━━━ 1s 9ms/step - loss: 0.0019 - val_loss: 0.0019
Epoch 4/50
147/147 ━━━━━━━━━━━━━━━━━━━━ 2s 10ms/step - loss: 0.0017 - val_loss: 0.0015
Epoch 5/50
147/147 ━━━━━━━━━━━━━━━━━━━━ 1s 9ms/step - loss: 0.0015 - val_loss: 0.0015
Epoch 6/50
147/147 ━━━━━━━━━━━━━━━━━━━━ 1s 9ms/step - loss: 0.0014 - val_loss: 0.0015
Epoch 7/50
147/147 ━━━━━━━━━━━━━━━━━━━━ 1s 9ms/step - loss: 0.0013 - val_loss: 0.0014
Epoch 8/50
147/147 ━━━━━━━━━━━━━━━━━━━━ 1s 9ms/step - loss: 0.0012 - val_loss: 0.0013
Epoch 9/50
147/147 ━━━━━━━━━━━━━━━━━━━━ 2s 12ms/step - loss: 0.0013 - val_loss: 0.0013
Epoch 10/50
147/147 ━━━━━━━━━━━━━━━━━━━━ 2s 9ms/step - loss: 0.0011 - val_loss: 0.0012
Epoch 11/50
147/147 ━━━━━━━━━━━━━━━━━━━━ 1s 9ms/step - loss: 0.0011 - val_loss: 0.0013
Epoch 12/50
147/147 ━━━━━━━━━━━━━━━━━━━━ 1s 10ms/step - loss: 0.0011 - val_loss: 0.0013
Epoch 13/50
147/147 ━━━━━━━━━━━━━━━━━━━━ 2s 9ms/step - loss: 0.0011 - val_loss: 0.0012
Epoch 14/50
147/147 ━━━━━━━━━━━━━━━━━━━━ 3s 10ms/step - loss: 0.0011 - val_loss: 0.0012
```

```
Epoch 15/50
147/147 ━━━━━━━━━━━━━━━━━━━━ 3s 13ms/step - loss: 0.0011 - val_loss: 0.0010
Epoch 16/50
147/147 ━━━━━━━━━━━━━━━━━━━━ 2s 11ms/step - loss: 9.5966e-04 - val_loss: 0.0012
Epoch 17/50
147/147 ━━━━━━━━━━━━━━━━━━━━ 1s 9ms/step - loss: 0.0010 - val_loss: 0.0010
Epoch 18/50
147/147 ━━━━━━━━━━━━━━━━━━━━ 3s 11ms/step - loss: 9.5206e-04 - val_loss: 0.0011
Epoch 19/50
147/147 ━━━━━━━━━━━━━━━━━━━━ 1s 10ms/step - loss: 9.3203e-04 - val_loss: 0.0011
Epoch 20/50
147/147 ━━━━━━━━━━━━━━━━━━━━ 3s 11ms/step - loss: 9.9616e-04 - val_loss: 0.0010
Epoch 21/50
147/147 ━━━━━━━━━━━━━━━━━━━━ 2s 14ms/step - loss: 9.4257e-04 - val_loss: 9.9842e-04
Epoch 22/50
147/147 ━━━━━━━━━━━━━━━━━━━━ 1s 9ms/step - loss: 9.1910e-04 - val_loss: 9.9040e-04
Epoch 23/50
147/147 ━━━━━━━━━━━━━━━━━━━━ 1s 9ms/step - loss: 8.9677e-04 - val_loss: 0.0011
Epoch 24/50
147/147 ━━━━━━━━━━━━━━━━━━━━ 1s 10ms/step - loss: 9.0719e-04 - val_loss: 9.1682e-04
Epoch 25/50
147/147 ━━━━━━━━━━━━━━━━━━━━ 3s 9ms/step - loss: 8.6379e-04 - val_loss: 9.7049e-04
Epoch 26/50
147/147 ━━━━━━━━━━━━━━━━━━━━ 3s 11ms/step - loss: 8.5960e-04 - val_loss: 9.6894e-04
Epoch 27/50
147/147 ━━━━━━━━━━━━━━━━━━━━ 3s 12ms/step - loss: 8.5917e-04 - val_loss: 0.0010
Epoch 28/50
147/147 ━━━━━━━━━━━━━━━━━━━━ 2s 11ms/step - loss: 8.8776e-04 - val_loss: 9.9107e-04
Epoch 29/50
147/147 ━━━━━━━━━━━━━━━━━━━━ 1s 9ms/step - loss: 8.4433e-04 - val_loss: 9.7217e-04
Epoch 30/50
147/147 ━━━━━━━━━━━━━━━━━━━━ 3s 10ms/step - loss: 8.0477e-04 - val_loss: 8.9791e-04
Epoch 31/50
147/147 ━━━━━━━━━━━━━━━━━━━━ 1s 9ms/step - loss: 8.0897e-04 - val_loss: 9.3000e-04
Epoch 32/50
147/147 ━━━━━━━━━━━━━━━━━━━━ 1s 9ms/step - loss: 8.4611e-04 - val_loss: 8.8575e-04
Epoch 33/50
147/147 ━━━━━━━━━━━━━━━━━━━━ 3s 11ms/step - loss: 7.8329e-04 - val_loss: 9.6201e-04
Epoch 34/50
147/147 ━━━━━━━━━━━━━━━━━━━━ 1s 9ms/step - loss: 7.6829e-04 - val_loss: 9.5084e-04
Epoch 35/50
147/147 ━━━━━━━━━━━━━━━━━━━━ 1s 10ms/step - loss: 8.1269e-04 - val_loss: 9.6333e-04
Epoch 36/50
147/147 ━━━━━━━━━━━━━━━━━━━━ 1s 9ms/step - loss: 7.8091e-04 - val_loss: 8.6733e-04
Epoch 37/50
147/147 ━━━━━━━━━━━━━━━━━━━━ 1s 9ms/step - loss: 7.3097e-04 - val_loss: 8.8527e-04
Epoch 38/50
147/147 ━━━━━━━━━━━━━━━━━━━━ 1s 9ms/step - loss: 7.2758e-04 - val_loss: 9.2844e-04
Epoch 39/50
147/147 ━━━━━━━━━━━━━━━━━━━━ 1s 10ms/step - loss: 7.0672e-04 - val_loss: 8.7165e-04
Epoch 40/50
147/147 ━━━━━━━━━━━━━━━━━━━━ 2s 12ms/step - loss: 7.1785e-04 - val_loss: 8.3017e-04
Epoch 41/50
147/147 ━━━━━━━━━━━━━━━━━━━━ 2s 10ms/step - loss: 7.2186e-04 - val_loss: 9.8508e-04
Epoch 42/50
147/147 ━━━━━━━━━━━━━━━━━━━━ 3s 10ms/step - loss: 7.7559e-04 - val_loss: 8.4407e-04
Epoch 43/50
147/147 ━━━━━━━━━━━━━━━━━━━━ 2s 9ms/step - loss: 6.7108e-04 - val_loss: 8.6630e-04
Epoch 44/50
147/147 ━━━━━━━━━━━━━━━━━━━━ 3s 9ms/step - loss: 7.1601e-04 - val_loss: 9.2305e-04
Epoch 45/50
```

```
147/147 ━━━━━━━━━━━━━━━━━━━━ 3s 14ms/step - loss: 6.8876e-04 - val_loss: 8.2636e-04
Epoch 46/50
147/147 ━━━━━━━━━━━━━━━━━━━━ 2s 10ms/step - loss: 6.6196e-04 - val_loss: 8.3225e-04
Epoch 47/50
147/147 ━━━━━━━━━━━━━━━━━━━━ 1s 9ms/step - loss: 6.7613e-04 - val_loss: 8.2498e-04
Epoch 48/50
147/147 ━━━━━━━━━━━━━━━━━━━━ 3s 9ms/step - loss: 6.6551e-04 - val_loss: 8.3928e-04
Epoch 49/50
147/147 ━━━━━━━━━━━━━━━━━━━━ 3s 11ms/step - loss: 6.7972e-04 - val_loss: 8.3770e-04
Epoch 50/50
147/147 ━━━━━━━━━━━━━━━━━━━━ 3s 13ms/step - loss: 6.3390e-04 - val_loss: 7.9362e-04
74/74 ━━━━━━━━━━━━━━━━━━━━ 1s 10ms/step
🔷 Computing SHAP for DwarkaSec8...
100%|===================| 11692/11704 [15:41<00:00]
```

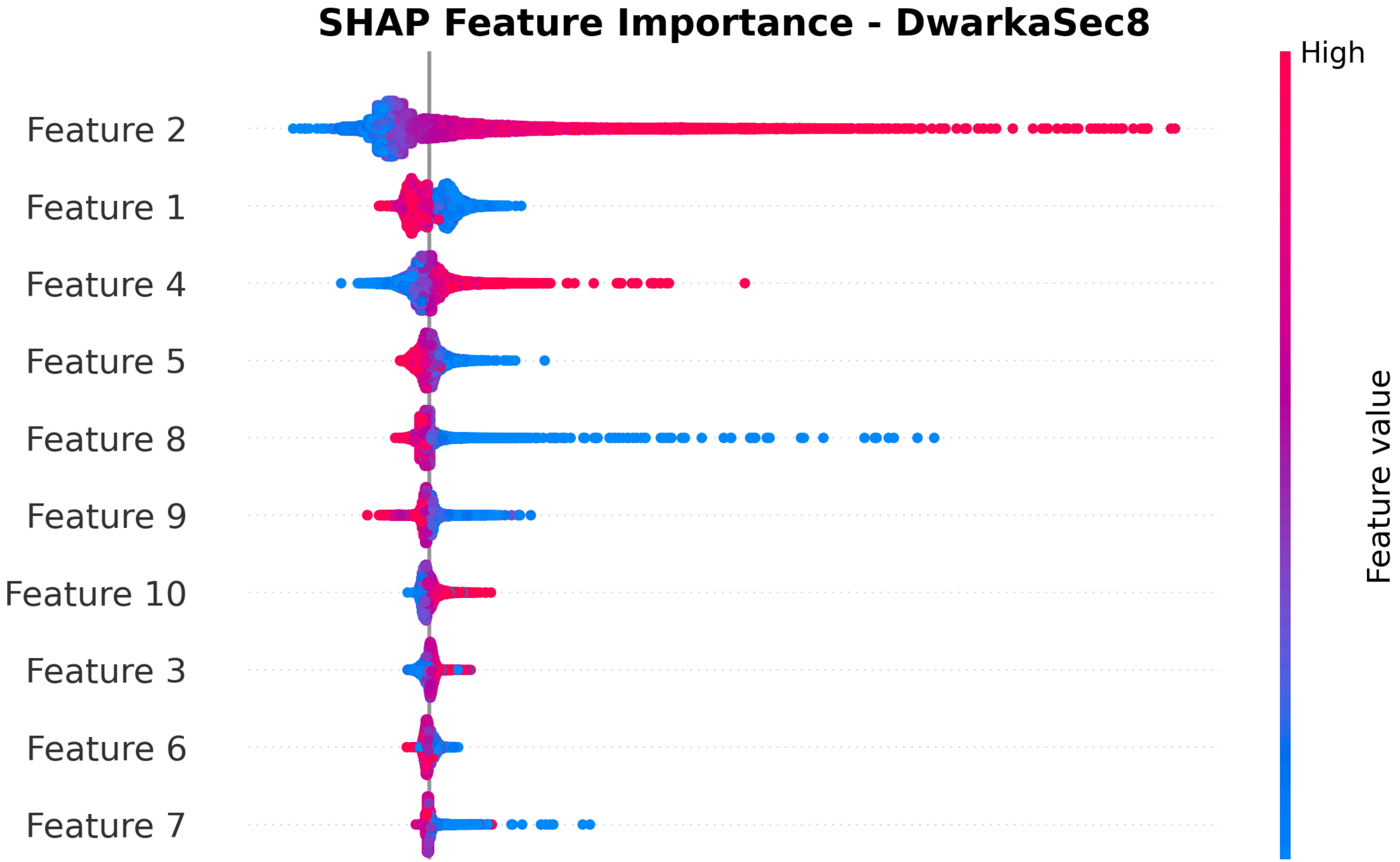

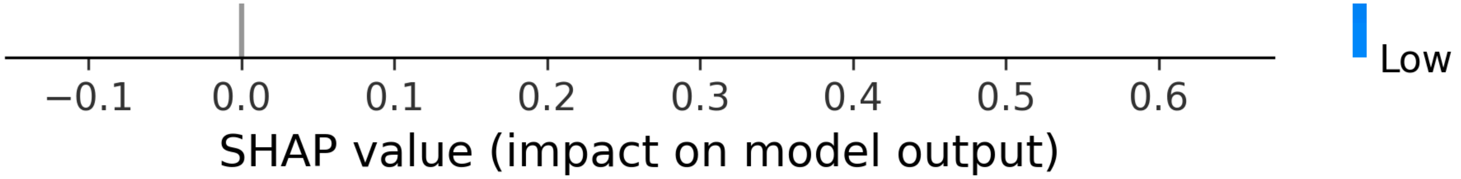


```
Processing Station: NehruNagar
/usr/local/lib/python3.11/dist-packages/pywt/_multilevel.py:43: UserWarning: Level value of 3 is too high: all coefficients will experience boundary effects.
  warnings.warn(
Selected features (10): ['Feature 1', 'Feature 2', 'Feature 3', 'Feature 4', 'Feature 5', 'Feature 6', 'Feature 7', 'Feature 8', 'Feature 9', 'Feature 10']
Epoch 1/50
/usr/local/lib/python3.11/dist-packages/keras/src/layers/rnn/rnn.py:200: UserWarning: Do not pass an `input_shape`/`input_dim` argument to a layer. When using Sequential mod
  super().__init__(**kwargs)
147/147 ━━━━━━━━━━━━━━━━━━━━ 6s 14ms/step - loss: 0.0160 - val_loss: 0.0055
Epoch 2/50
147/147 ━━━━━━━━━━━━━━━━━━━━ 2s 10ms/step - loss: 0.0040 - val_loss: 0.0025
Epoch 3/50
147/147 ━━━━━━━━━━━━━━━━━━━━ 1s 10ms/step - loss: 0.0022 - val_loss: 0.0022
Epoch 4/50
147/147 ━━━━━━━━━━━━━━━━━━━━ 2s 15ms/step - loss: 0.0020 - val_loss: 0.0019
Epoch 5/50
147/147 ━━━━━━━━━━━━━━━━━━━━ 2s 11ms/step - loss: 0.0018 - val_loss: 0.0019
Epoch 6/50
147/147 ━━━━━━━━━━━━━━━━━━━━ 1s 10ms/step - loss: 0.0017 - val_loss: 0.0019
Epoch 7/50
147/147 ━━━━━━━━━━━━━━━━━━━━ 3s 10ms/step - loss: 0.0017 - val_loss: 0.0017
Epoch 8/50
147/147 ━━━━━━━━━━━━━━━━━━━━ 1s 10ms/step - loss: 0.0016 - val_loss: 0.0017
Epoch 9/50
147/147 ━━━━━━━━━━━━━━━━━━━━ 1s 10ms/step - loss: 0.0016 - val_loss: 0.0016
Epoch 10/50
147/147 ━━━━━━━━━━━━━━━━━━━━ 3s 14ms/step - loss: 0.0015 - val_loss: 0.0015
Epoch 11/50
147/147 ━━━━━━━━━━━━━━━━━━━━ 2s 11ms/step - loss: 0.0014 - val_loss: 0.0015
Epoch 12/50
147/147 ━━━━━━━━━━━━━━━━━━━━ 2s 10ms/step - loss: 0.0013 - val_loss: 0.0013
Epoch 13/50
147/147 ━━━━━━━━━━━━━━━━━━━━ 2s 11ms/step - loss: 0.0012 - val_loss: 0.0013
Epoch 14/50
147/147 ━━━━━━━━━━━━━━━━━━━━ 3s 11ms/step - loss: 0.0012 - val_loss: 0.0011
Epoch 15/50
147/147 ━━━━━━━━━━━━━━━━━━━━ 2s 11ms/step - loss: 0.0011 - val_loss: 0.0010
Epoch 16/50
147/147 ━━━━━━━━━━━━━━━━━━━━ 2s 14ms/step - loss: 9.6888e-04 - val_loss: 0.0011
Epoch 17/50
147/147 ━━━━━━━━━━━━━━━━━━━━ 2s 11ms/step - loss: 9.3115e-04 - val_loss: 9.4134e-04
Epoch 18/50
147/147 ━━━━━━━━━━━━━━━━━━━━ 2s 11ms/step - loss: 8.7871e-04 - val_loss: 9.7186e-04
Epoch 19/50
147/147 ━━━━━━━━━━━━━━━━━━━━ 2s 10ms/step - loss: 8.8865e-04 - val_loss: 8.9043e-04
Epoch 20/50
147/147 ━━━━━━━━━━━━━━━━━━━━ 2s 10ms/step - loss: 8.5471e-04 - val_loss: 8.8041e-04
Epoch 21/50
147/147 ━━━━━━━━━━━━━━━━━━━━ 2s 11ms/step - loss: 8.3435e-04 - val_loss: 9.0028e-04
Epoch 22/50
147/147 ━━━━━━━━━━━━━━━━━━━━ 2s 14ms/step - loss: 8.0965e-04 - val_loss: 8.6119e-04
Epoch 23/50
147/147 ━━━━━━━━━━━━━━━━━━━━ 2s 12ms/step - loss: 7.8188e-04 - val_loss: 8.5608e-04
```

```
[illegible]
Epoch 24/50
147/147 ━━━━━━━━━━━━━━━━━━━━ 2s 11ms/step - loss: 7.7887e-04 - val_loss: 8.6121e-04
Epoch 25/50
147/147 ━━━━━━━━━━━━━━━━━━━━ 2s 10ms/step - loss: 7.6534e-04 - val_loss: 8.4389e-04
Epoch 26/50
147/147 ━━━━━━━━━━━━━━━━━━━━ 2s 10ms/step - loss: 8.2624e-04 - val_loss: 8.0844e-04
Epoch 27/50
147/147 ━━━━━━━━━━━━━━━━━━━━ 2s 10ms/step - loss: 7.5788e-04 - val_loss: 9.1931e-04
Epoch 28/50
147/147 ━━━━━━━━━━━━━━━━━━━━ 2s 11ms/step - loss: 7.1898e-04 - val_loss: 8.1534e-04
Epoch 29/50
147/147 ━━━━━━━━━━━━━━━━━━━━ 3s 11ms/step - loss: 7.2901e-04 - val_loss: 7.9702e-04
Epoch 30/50
147/147 ━━━━━━━━━━━━━━━━━━━━ 2s 10ms/step - loss: 6.7764e-04 - val_loss: 7.4511e-04
Epoch 31/50
147/147 ━━━━━━━━━━━━━━━━━━━━ 2s 11ms/step - loss: 6.8142e-04 - val_loss: 7.3562e-04
Epoch 32/50
147/147 ━━━━━━━━━━━━━━━━━━━━ 2s 11ms/step - loss: 6.6463e-04 - val_loss: 7.1834e-04
Epoch 33/50
147/147 ━━━━━━━━━━━━━━━━━━━━ 3s 11ms/step - loss: 7.0944e-04 - val_loss: 8.2817e-04
Epoch 34/50
147/147 ━━━━━━━━━━━━━━━━━━━━ 3s 14ms/step - loss: 7.0913e-04 - val_loss: 6.8719e-04
Epoch 35/50
147/147 ━━━━━━━━━━━━━━━━━━━━ 2s 10ms/step - loss: 6.3247e-04 - val_loss: 8.3587e-04
Epoch 36/50
147/147 ━━━━━━━━━━━━━━━━━━━━ 2s 10ms/step - loss: 6.4238e-04 - val_loss: 6.6148e-04
Epoch 37/50
147/147 ━━━━━━━━━━━━━━━━━━━━ 3s 10ms/step - loss: 6.1425e-04 - val_loss: 6.7403e-04
Epoch 38/50
147/147 ━━━━━━━━━━━━━━━━━━━━ 2s 11ms/step - loss: 5.7200e-04 - val_loss: 6.7871e-04
Epoch 39/50
147/147 ━━━━━━━━━━━━━━━━━━━━ 2s 10ms/step - loss: 6.0181e-04 - val_loss: 7.0436e-04
Epoch 40/50
147/147 ━━━━━━━━━━━━━━━━━━━━ 3s 14ms/step - loss: 6.3197e-04 - val_loss: 6.6876e-04
Epoch 41/50
147/147 ━━━━━━━━━━━━━━━━━━━━ 2s 12ms/step - loss: 5.5077e-04 - val_loss: 6.6267e-04
Epoch 42/50
147/147 ━━━━━━━━━━━━━━━━━━━━ 3s 12ms/step - loss: 5.1988e-04 - val_loss: 7.1633e-04
Epoch 43/50
147/147 ━━━━━━━━━━━━━━━━━━━━ 2s 11ms/step - loss: 5.8081e-04 - val_loss: 6.5465e-04
Epoch 44/50
147/147 ━━━━━━━━━━━━━━━━━━━━ 2s 10ms/step - loss: 5.3919e-04 - val_loss: 6.4603e-04
Epoch 45/50
147/147 ━━━━━━━━━━━━━━━━━━━━ 2s 10ms/step - loss: 5.1385e-04 - val_loss: 6.4663e-04
Epoch 46/50
147/147 ━━━━━━━━━━━━━━━━━━━━ 2s 13ms/step - loss: 5.1779e-04 - val_loss: 5.9376e-04
Epoch 47/50
147/147 ━━━━━━━━━━━━━━━━━━━━ 2s 13ms/step - loss: 5.0461e-04 - val_loss: 6.2568e-04
Epoch 48/50
147/147 ━━━━━━━━━━━━━━━━━━━━ 2s 10ms/step - loss: 4.8246e-04 - val_loss: 5.8052e-04
Epoch 49/50
147/147 ━━━━━━━━━━━━━━━━━━━━ 3s 11ms/step - loss: 4.9714e-04 - val_loss: 6.3296e-04
Epoch 50/50
147/147 ━━━━━━━━━━━━━━━━━━━━ 2s 10ms/step - loss: 5.2628e-04 - val_loss: 6.1286e-04
74/74 ━━━━━━━━━━━━━━━━━━━━ 1s 7ms/step
🔷 Computing SHAP for NehruNagar...
100%|===================| 11691/11705 [14:51<00:01]
```

SHAP Feature Importance - NehruNagar

High

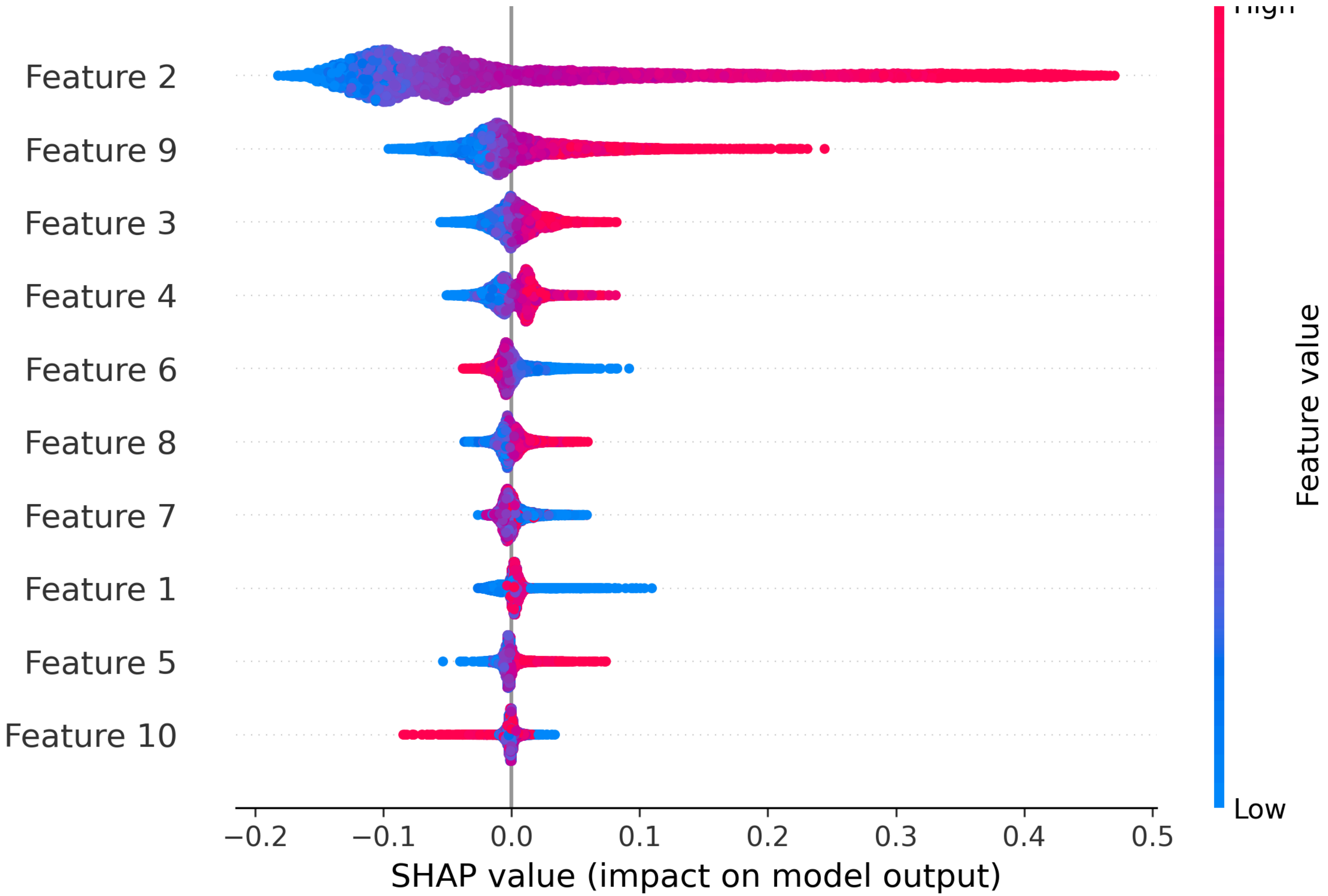


```
Processing Station: Najafgarh
/usr/local/lib/python3.11/dist-packages/pywt/_multilevel.py:43: UserWarning: Level value of 3 is too high: all coefficients will experience boundary effects.
  warnings.warn(
Selected features (10): ['Feature 1', 'Feature 2', 'Feature 3', 'Feature 4', 'Feature 5', 'Feature 6', 'Feature 7', 'Feature 8', 'Feature 9', 'Feature 10']
Epoch 1/50
/usr/local/lib/python3.11/dist-packages/keras/src/layers/rnn/rnn.py:200: UserWarning: Do not pass an `input_shape`/`input_dim` argument to a layer. When using Sequential mod
  super().__init__(**kwargs)
147/147 ━━━━━━━━━━━━━━━━━━━━ 6s 17ms/step - loss: 0.0036 - val_loss: 0.0019
Epoch 2/50
```

```
[illegible]
147/147 ━━━━━━━━━━━━━━━━━━━━ 2s 12ms/step - loss: 0.0023 - val_loss: 0.0017
Epoch 3/50
147/147 ━━━━━━━━━━━━━━━━━━━━ 2s 10ms/step - loss: 0.0022 - val_loss: 0.0014
Epoch 4/50
147/147 ━━━━━━━━━━━━━━━━━━━━ 3s 10ms/step - loss: 0.0017 - val_loss: 0.0014
Epoch 5/50
147/147 ━━━━━━━━━━━━━━━━━━━━ 3s 10ms/step - loss: 0.0015 - val_loss: 0.0013
Epoch 6/50
147/147 ━━━━━━━━━━━━━━━━━━━━ 2s 10ms/step - loss: 0.0013 - val_loss: 0.0012
Epoch 7/50
147/147 ━━━━━━━━━━━━━━━━━━━━ 2s 12ms/step - loss: 0.0011 - val_loss: 0.0011
Epoch 8/50
147/147 ━━━━━━━━━━━━━━━━━━━━ 2s 14ms/step - loss: 0.0012 - val_loss: 0.0010
Epoch 9/50
147/147 ━━━━━━━━━━━━━━━━━━━━ 2s 11ms/step - loss: 0.0012 - val_loss: 9.6392e-04
Epoch 10/50
147/147 ━━━━━━━━━━━━━━━━━━━━ 3s 11ms/step - loss: 9.8210e-04 - val_loss: 0.0010
Epoch 11/50
147/147 ━━━━━━━━━━━━━━━━━━━━ 2s 10ms/step - loss: 0.0010 - val_loss: 8.9508e-04
Epoch 12/50
147/147 ━━━━━━━━━━━━━━━━━━━━ 3s 10ms/step - loss: 9.3317e-04 - val_loss: 0.0010
Epoch 13/50
147/147 ━━━━━━━━━━━━━━━━━━━━ 2s 13ms/step - loss: 9.3773e-04 - val_loss: 9.0124e-04
Epoch 14/50
147/147 ━━━━━━━━━━━━━━━━━━━━ 3s 12ms/step - loss: 8.7785e-04 - val_loss: 8.6074e-04
Epoch 15/50
147/147 ━━━━━━━━━━━━━━━━━━━━ 2s 11ms/step - loss: 7.4162e-04 - val_loss: 7.4576e-04
Epoch 16/50
147/147 ━━━━━━━━━━━━━━━━━━━━ 2s 10ms/step - loss: 7.8252e-04 - val_loss: 7.0737e-04
Epoch 17/50
147/147 ━━━━━━━━━━━━━━━━━━━━ 2s 10ms/step - loss: 6.7027e-04 - val_loss: 7.9784e-04
Epoch 18/50
147/147 ━━━━━━━━━━━━━━━━━━━━ 2s 11ms/step - loss: 6.9906e-04 - val_loss: 6.4793e-04
Epoch 19/50
147/147 ━━━━━━━━━━━━━━━━━━━━ 3s 11ms/step - loss: 6.7527e-04 - val_loss: 6.4949e-04
Epoch 20/50
147/147 ━━━━━━━━━━━━━━━━━━━━ 2s 14ms/step - loss: 6.1487e-04 - val_loss: 5.9636e-04
Epoch 21/50
147/147 ━━━━━━━━━━━━━━━━━━━━ 2s 11ms/step - loss: 5.9353e-04 - val_loss: 5.4973e-04
Epoch 22/50
147/147 ━━━━━━━━━━━━━━━━━━━━ 2s 10ms/step - loss: 5.7205e-04 - val_loss: 6.5379e-04
Epoch 23/50
147/147 ━━━━━━━━━━━━━━━━━━━━ 3s 11ms/step - loss: 5.9515e-04 - val_loss: 5.6004e-04
Epoch 24/50
147/147 ━━━━━━━━━━━━━━━━━━━━ 2s 10ms/step - loss: 5.4549e-04 - val_loss: 5.0189e-04
Epoch 25/50
147/147 ━━━━━━━━━━━━━━━━━━━━ 3s 14ms/step - loss: 4.8935e-04 - val_loss: 5.4762e-04
Epoch 26/50
147/147 ━━━━━━━━━━━━━━━━━━━━ 2s 13ms/step - loss: 5.1524e-04 - val_loss: 5.8938e-04
Epoch 27/50
147/147 ━━━━━━━━━━━━━━━━━━━━ 2s 10ms/step - loss: 5.1665e-04 - val_loss: 4.8729e-04
Epoch 28/50
147/147 ━━━━━━━━━━━━━━━━━━━━ 3s 10ms/step - loss: 4.7192e-04 - val_loss: 4.7287e-04
Epoch 29/50
147/147 ━━━━━━━━━━━━━━━━━━━━ 2s 10ms/step - loss: 4.5263e-04 - val_loss: 5.3098e-04
Epoch 30/50
147/147 ━━━━━━━━━━━━━━━━━━━━ 2s 10ms/step - loss: 4.6260e-04 - val_loss: 4.4675e-04
Epoch 31/50
147/147 ━━━━━━━━━━━━━━━━━━━━ 2s 10ms/step - loss: 4.4438e-04 - val_loss: 4.3032e-04
Epoch 32/50
[illegible]
```

```
147/147 ━━━━━━━━━━━━━━━━━━━━ 2s 13ms/step - loss: 4.4518e-04 - val_loss: 4.1364e-04
Epoch 33/50
147/147 ━━━━━━━━━━━━━━━━━━━━ 2s 13ms/step - loss: 4.1150e-04 - val_loss: 4.7927e-04
Epoch 34/50
147/147 ━━━━━━━━━━━━━━━━━━━━ 2s 10ms/step - loss: 4.4333e-04 - val_loss: 4.0968e-04
Epoch 35/50
147/147 ━━━━━━━━━━━━━━━━━━━━ 2s 10ms/step - loss: 4.0412e-04 - val_loss: 4.6108e-04
Epoch 36/50
147/147 ━━━━━━━━━━━━━━━━━━━━ 3s 12ms/step - loss: 4.0462e-04 - val_loss: 4.0909e-04
Epoch 37/50
147/147 ━━━━━━━━━━━━━━━━━━━━ 2s 11ms/step - loss: 3.7658e-04 - val_loss: 4.0261e-04
Epoch 38/50
147/147 ━━━━━━━━━━━━━━━━━━━━ 3s 15ms/step - loss: 3.4843e-04 - val_loss: 4.0397e-04
Epoch 39/50
147/147 ━━━━━━━━━━━━━━━━━━━━ 2s 12ms/step - loss: 3.5568e-04 - val_loss: 3.8457e-04
Epoch 40/50
147/147 ━━━━━━━━━━━━━━━━━━━━ 3s 12ms/step - loss: 3.3814e-04 - val_loss: 3.8318e-04
Epoch 41/50
147/147 ━━━━━━━━━━━━━━━━━━━━ 2s 10ms/step - loss: 3.7477e-04 - val_loss: 4.1665e-04
Epoch 42/50
147/147 ━━━━━━━━━━━━━━━━━━━━ 2s 11ms/step - loss: 3.8512e-04 - val_loss: 3.8212e-04
Epoch 43/50
147/147 ━━━━━━━━━━━━━━━━━━━━ 2s 10ms/step - loss: 3.5281e-04 - val_loss: 4.0316e-04
Epoch 44/50
147/147 ━━━━━━━━━━━━━━━━━━━━ 2s 14ms/step - loss: 3.4502e-04 - val_loss: 3.5243e-04
Epoch 45/50
147/147 ━━━━━━━━━━━━━━━━━━━━ 2s 14ms/step - loss: 3.3058e-04 - val_loss: 3.7926e-04
Epoch 46/50
147/147 ━━━━━━━━━━━━━━━━━━━━ 2s 10ms/step - loss: 3.4793e-04 - val_loss: 3.5965e-04
Epoch 47/50
147/147 ━━━━━━━━━━━━━━━━━━━━ 3s 11ms/step - loss: 3.2101e-04 - val_loss: 3.6273e-04
Epoch 48/50
147/147 ━━━━━━━━━━━━━━━━━━━━ 2s 10ms/step - loss: 3.2030e-04 - val_loss: 3.7272e-04
Epoch 49/50
147/147 ━━━━━━━━━━━━━━━━━━━━ 2s 10ms/step - loss: 3.0625e-04 - val_loss: 4.0282e-04
Epoch 50/50
147/147 ━━━━━━━━━━━━━━━━━━━━ 3s 13ms/step - loss: 3.2363e-04 - val_loss: 3.5175e-04
74/74 ━━━━━━━━━━━━━━━━━━━━ 1s 10ms/step
🔷 Computing SHAP for Najafgarh...
100%|===================| 11694/11704 [17:41<00:00]
```

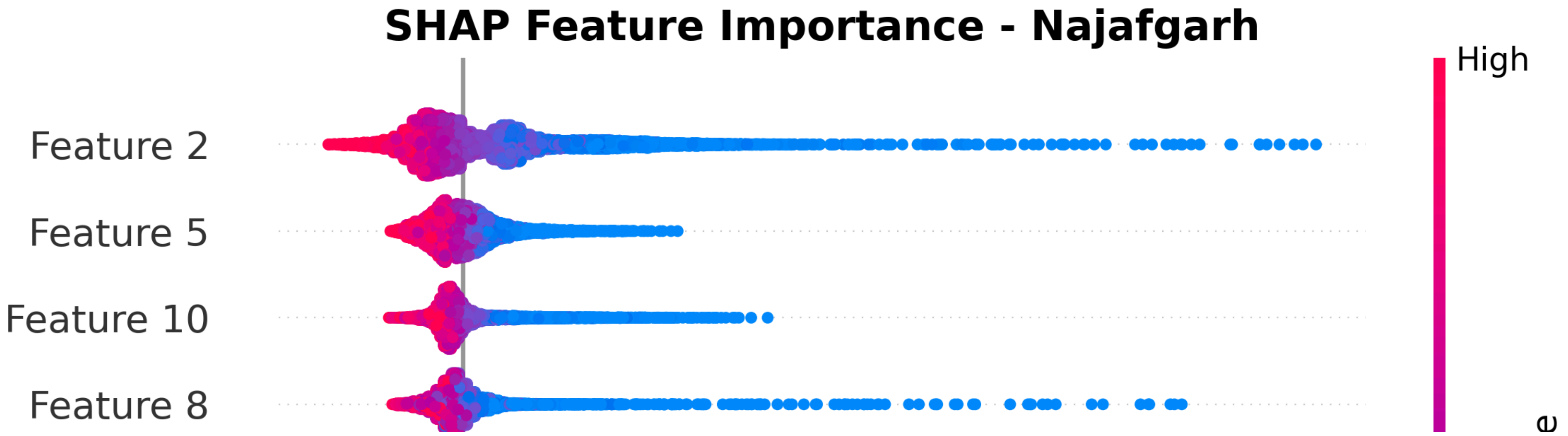

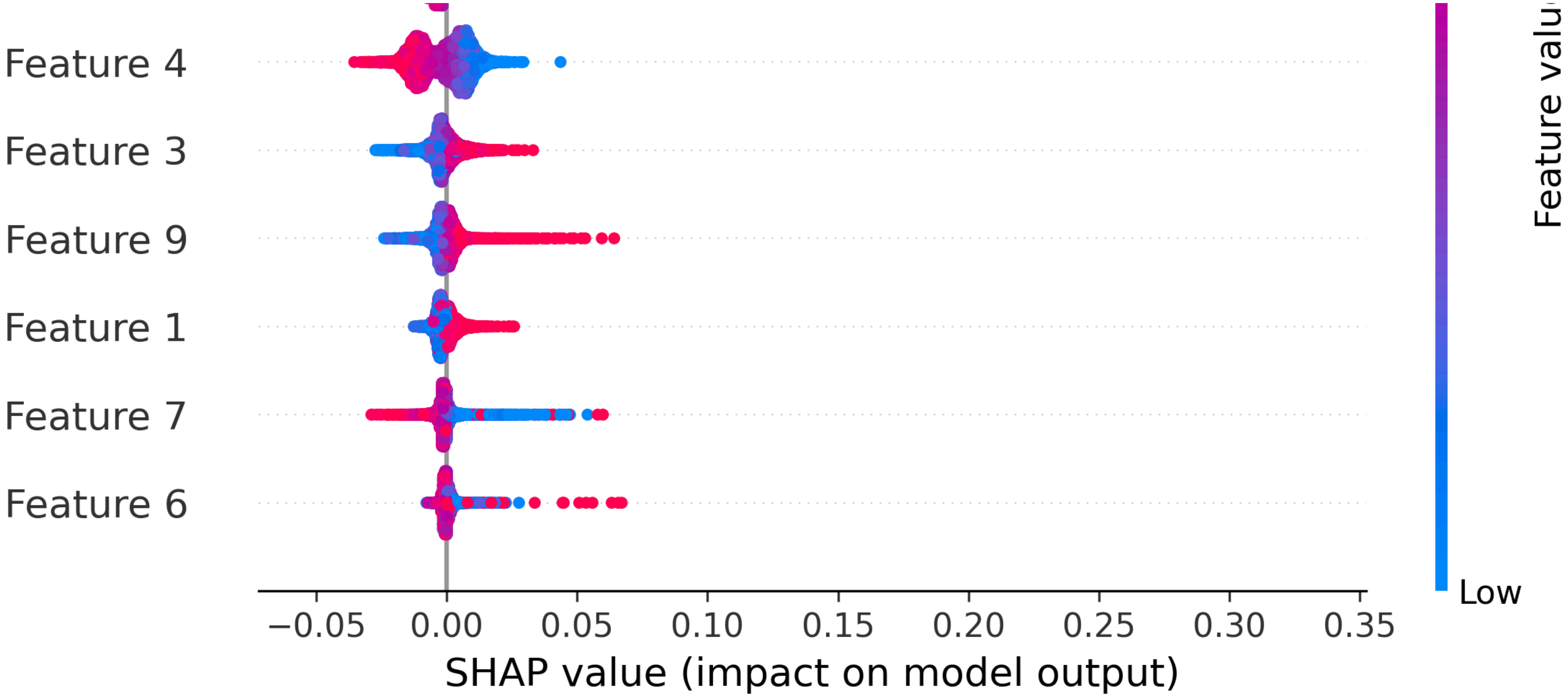


```
Processing Station: Okhla
/usr/local/lib/python3.11/dist-packages/pywt/_multilevel.py:43: UserWarning: Level value of 3 is too high: all coefficients will experience boundary effects.
  warnings.warn(
Selected features (10): ['Feature 1', 'Feature 2', 'Feature 3', 'Feature 4', 'Feature 5', 'Feature 6', 'Feature 7', 'Feature 8', 'Feature 9', 'Feature 10']
Epoch 1/50
/usr/local/lib/python3.11/dist-packages/keras/src/layers/rnn/rnn.py:200: UserWarning: Do not pass an `input_shape`/`input_dim` argument to a layer. When using Sequential mod
  super().__init__(**kwargs)
147/147 ━━━━━━━━━━━━━━━━━━━━ 6s 14ms/step - loss: 0.0113 - val_loss: 0.0068
Epoch 2/50
147/147 ━━━━━━━━━━━━━━━━━━━━ 2s 11ms/step - loss: 0.0061 - val_loss: 0.0041
Epoch 3/50
147/147 ━━━━━━━━━━━━━━━━━━━━ 2s 10ms/step - loss: 0.0038 - val_loss: 0.0024
Epoch 4/50
147/147 ━━━━━━━━━━━━━━━━━━━━ 3s 11ms/step - loss: 0.0026 - val_loss: 0.0021
Epoch 5/50
147/147 ━━━━━━━━━━━━━━━━━━━━ 3s 12ms/step - loss: 0.0023 - val_loss: 0.0018
Epoch 6/50
147/147 ━━━━━━━━━━━━━━━━━━━━ 2s 14ms/step - loss: 0.0019 - val_loss: 0.0017
Epoch 7/50
147/147 ━━━━━━━━━━━━━━━━━━━━ 2s 11ms/step - loss: 0.0018 - val_loss: 0.0017
Epoch 8/50
147/147 ━━━━━━━━━━━━━━━━━━━━ 2s 12ms/step - loss: 0.0016 - val_loss: 0.0015
Epoch 9/50
113/147 ━━━━━━━━━━━━━━━━━━━━ 0s 10ms/step - loss: 0.0015
```

```
import numpy as np
import pandas as pd
import matplotlib.pyplot as plt
import os
import pywt
import shap

from sklearn.decomposition import PCA
from sklearn.preprocessing import StandardScaler, MinMaxScaler
from sklearn.model_selection import train_test_split
from sklearn.metrics import mean_squared_error, mean_absolute_error, r2_score
from sklearn.ensemble import RandomForestRegressor

import tensorflow as tf
from tensorflow.keras.models import Sequential
from tensorflow.keras.layers import LSTM, Dense, Bidirectional

# ============================================
# Hybrid AOAOA Optimizer
# ============================================
class HybridOptimizer:
    def __init__(self, objective_function, lower_bound, upper_bound, population_size, iterations):
        self.objective_function = objective_function
        self.lower_bound = np.array(lower_bound)
        self.upper_bound = np.array(upper_bound)
        self.population_size = population_size
        self.iterations = iterations
        self.population = np.random.uniform(
            low=self.lower_bound, high=self.upper_bound,
            size=(population_size, len(lower_bound))
        )
        self.best_solution = None
        self.best_fitness = float('inf')

    def optimize(self):
        for _ in range(self.iterations):
            for i in range(self.population_size):
                perturb = np.random.uniform(-0.1, 0.1, size=self.population.shape[1])
                candidate_aquila = self.population[i] + perturb
                candidate_aquila = np.clip(candidate_aquila, self.lower_bound, self.upper_bound)
                fitness_aquila = self.objective_function(candidate_aquila)

                if fitness_aquila < self.best_fitness:
                    self.best_fitness = fitness_aquila
                    self.best_solution = candidate_aquila

            for i in range(self.population_size):
                partner_idx = np.random.randint(self.population_size)
                partner = self.population[partner_idx]
                candidate_arithmetic = (self.population[i] + partner) / 2
                candidate_arithmetic = np.clip(candidate_arithmetic, self.lower_bound, self.upper_bound)
                fitness_arithmetic = self.objective_function(candidate_arithmetic)

                if fitness_arithmetic < self.best_fitness:
                    self.best_fitness = fitness_arithmetic
```

```
                    self.best_solution = candidate_arithmetic

        return self.best_solution

# ============================================
# Feature Extraction
# ============================================
def extract_wavelet_features(X, wavelet='db4', level=3, num_features=50):
    features = []
    for sample in X:
        coeffs = pywt.wavedec(sample, wavelet, level=level)
        flattened = np.concatenate([c.flatten() for c in coeffs])
        features.append(flattened[:num_features])
    return np.array(features)

def apply_pca(X, n_components=10):
    scaler = StandardScaler()
    X_scaled = scaler.fit_transform(X)
    pca = PCA(n_components=n_components)
    return pca.fit_transform(X_scaled)

def extract_combined_features(X):
    X_wavelet = extract_wavelet_features(X)
    X_pca = apply_pca(X_wavelet)
    return X_pca

# ============================================
# Bi-LSTM Model
# ============================================
def build_lstm_model(input_shape):
    model = Sequential([
        Bidirectional(LSTM(50, return_sequences=True, input_shape=input_shape)),
        Bidirectional(LSTM(50, return_sequences=False)),
        Dense(1)
    ])
    model.compile(optimizer='adam', loss='mean_squared_error')
    return model

# ============================================
# Training & Evaluation
# ============================================
def evaluate_model(X, y):
    if X.shape[1] == 0:
        raise ValueError("No features selected! Adjust AOAOA feature selection.")
    X_train, X_test, y_train, y_test = train_test_split(X, y, test_size=0.2, random_state=42)
    X_train = X_train.reshape(X_train.shape[0], X_train.shape[1], 1)
    X_test = X_test.reshape(X_test.shape[0], X_test.shape[1], 1)

    model = build_lstm_model((X_train.shape[1], 1))
    model.fit(X_train, y_train, epochs=50, batch_size=64, validation_data=(X_test, y_test), verbose=1)
    y_pred = model.predict(X_test)

    mse = mean_squared_error(y_test, y_pred)
    mae = mean_absolute_error(y_test, y_pred)
    rmse = np.sqrt(mse)
```

```
        r2 = r2_score(y_test, y_pred)

        return mse, mae, rmse, r2, y_test, y_pred

    # ============================================
    # Run Okhla Station
    # ============================================
    station = 'Okhla'
    file_path = '/content/Okhla_Hourly.csv'

    threshold = 0.40

    print(f"\n📊 Processing Station: {station}")

    df = pd.read_csv(file_path)

    scaler = MinMaxScaler()
    X_full = scaler.fit_transform(df.iloc[:, :-1].values)
    y = scaler.fit_transform(df.iloc[:, -1].values.reshape(-1, 1)).ravel()

    # Feature Extraction
    X_extracted = extract_combined_features(X_full)

    # Feature Selection with Hybrid AOAOA
    objective_function = lambda x: np.sum(x**2)
    hybrid_optimizer = HybridOptimizer(
        objective_function, lower_bound=[-1]*X_extracted.shape[1],
        upper_bound=[1]*X_extracted.shape[1],
        population_size=50, iterations=100
    )
    selected_features = hybrid_optimizer.optimize()

    X_selected = X_extracted[:, selected_features > threshold]
    if X_selected.shape[1] > 0:
        X_final = X_selected
    else:
        X_final = X_extracted

    feature_names = [f"Feature {i+1}" for i in range(X_final.shape[1])]
    print(f"✅ Selected features: {feature_names}")

    # Final Model Evaluation
    mse, mae, rmse, r2, y_test, y_pred = evaluate_model(X_final, y)

    print("\n📋 Evaluation Metrics:")
    print(f"MSE: {mse:.4f}, MAE: {mae:.4f}, RMSE: {rmse:.4f}, R²: {r2:.4f}")

    # SHAP with RF surrogate
    print(f"🔷 Computing SHAP for {station}...")
    rf = RandomForestRegressor(n_estimators=100, random_state=42)
    rf.fit(X_final, y)

    explainer = shap.Explainer(rf, X_final)
    shap_values = explainer(X_final, check_additivity=False)
```

```
# SHAP Summary Plot (beeswarm)
plt.figure(dpi=300)
plt.title(f"SHAP Feature Importance - {station}", fontsize=14, fontweight='bold')
shap.summary_plot(
    shap_values,
    X_final,
    feature_names=feature_names,
    show=True
)
```

```
📊 Processing Station: Okhla
/usr/local/lib/python3.11/dist-packages/pywt/_multilevel.py:43: UserWarning: Level value of 3 is too high: all coefficients will experience boundary effects.
  warnings.warn(
✅ Selected features: ['Feature 1', 'Feature 2', 'Feature 3', 'Feature 4', 'Feature 5', 'Feature 6', 'Feature 7', 'Feature 8', 'Feature 9', 'Feature 10']
Epoch 1/50
/usr/local/lib/python3.11/dist-packages/keras/src/layers/rnn/rnn.py:200: UserWarning: Do not pass an `input_shape`/`input_dim` argument to a layer. When using Sequential mod
  super().__init__(**kwargs)
147/147 ━━━━━━━━━━━━━━━━━━━━ 8s 20ms/step - loss: 0.0108 - val_loss: 0.0064
Epoch 2/50
147/147 ━━━━━━━━━━━━━━━━━━━━ 3s 17ms/step - loss: 0.0054 - val_loss: 0.0036
Epoch 3/50
147/147 ━━━━━━━━━━━━━━━━━━━━ 5s 17ms/step - loss: 0.0032 - val_loss: 0.0023
Epoch 4/50
147/147 ━━━━━━━━━━━━━━━━━━━━ 2s 17ms/step - loss: 0.0024 - val_loss: 0.0023
Epoch 5/50
147/147 ━━━━━━━━━━━━━━━━━━━━ 3s 18ms/step - loss: 0.0022 - val_loss: 0.0021
Epoch 6/50
147/147 ━━━━━━━━━━━━━━━━━━━━ 3s 22ms/step - loss: 0.0017 - val_loss: 0.0016
Epoch 7/50
147/147 ━━━━━━━━━━━━━━━━━━━━ 2s 17ms/step - loss: 0.0017 - val_loss: 0.0015
Epoch 8/50
147/147 ━━━━━━━━━━━━━━━━━━━━ 3s 19ms/step - loss: 0.0016 - val_loss: 0.0013
Epoch 9/50
147/147 ━━━━━━━━━━━━━━━━━━━━ 6s 23ms/step - loss: 0.0015 - val_loss: 0.0012
Epoch 10/50
147/147 ━━━━━━━━━━━━━━━━━━━━ 4s 19ms/step - loss: 0.0012 - val_loss: 0.0012
Epoch 11/50
147/147 ━━━━━━━━━━━━━━━━━━━━ 5s 17ms/step - loss: 0.0011 - val_loss: 0.0011
Epoch 12/50
147/147 ━━━━━━━━━━━━━━━━━━━━ 4s 24ms/step - loss: 0.0010 - val_loss: 9.1109e-04
Epoch 13/50
147/147 ━━━━━━━━━━━━━━━━━━━━ 4s 18ms/step - loss: 0.0011 - val_loss: 8.6759e-04
Epoch 14/50
147/147 ━━━━━━━━━━━━━━━━━━━━ 6s 22ms/step - loss: 0.0011 - val_loss: 8.2079e-04
Epoch 15/50
147/147 ━━━━━━━━━━━━━━━━━━━━ 3s 18ms/step - loss: 8.7602e-04 - val_loss: 9.8163e-04
Epoch 16/50
147/147 ━━━━━━━━━━━━━━━━━━━━ 3s 18ms/step - loss: 9.4375e-04 - val_loss: 7.9730e-04
Epoch 17/50
147/147 ━━━━━━━━━━━━━━━━━━━━ 6s 21ms/step - loss: 8.6960e-04 - val_loss: 7.8126e-04
Epoch 18/50
147/147 ━━━━━━━━━━━━━━━━━━━━ 5s 18ms/step - loss: 8.3220e-04 - val_loss: 7.3331e-04
Epoch 19/50
147/147 ━━━━━━━━━━━━━━━━━━━━ 3s 19ms/step - loss: 8.5638e-04 - val_loss: 7.5884e-04
Epoch 20/50
147/147 ━━━━━━━━━━━━━━━━━━━━ 6s 22ms/step - loss: 7.4071e-04 - val_loss: 7.5670e-04
Epoch 21/50
147/147 ━━━━━━━━━━━━━━━━━━━━ 3s 18ms/step - loss: 7.4795e-04 - val_loss: 7.4278e-04
Epoch 22/50
147/147 ━━━━━━━━━━━━━━━━━━━━ 2s 17ms/step - loss: 7.5108e-04 - val_loss: 7.3392e-04
Epoch 23/50
147/147 ━━━━━━━━━━━━━━━━━━━━ 3s 18ms/step - loss: 7.3483e-04 - val_loss: 7.0068e-04
Epoch 24/50
147/147 ━━━━━━━━━━━━━━━━━━━━ 4s 26ms/step - loss: 6.4459e-04 - val_loss: 7.9426e-04
Epoch 25/50
147/147 ━━━━━━━━━━━━━━━━━━━━ 3s 17ms/step - loss: 6.4860e-04 - val_loss: 6.7235e-04
Epoch 26/50
147/147 ━━━━━━━━━━━━━━━━━━━━ 3s 18ms/step - loss: 6.7237e-04 - val_loss: 6.2329e-04
Epoch 27/50
[illegible]
```

```
147/147 ━━━━━━━━━━━━━━━━━━━━ 6s 22ms/step - loss: 6.5721e-04 - val_loss: 6.8612e-04
Epoch 28/50
147/147 ━━━━━━━━━━━━━━━━━━━━ 3s 19ms/step - loss: 6.2065e-04 - val_loss: 6.5758e-04
Epoch 29/50
147/147 ━━━━━━━━━━━━━━━━━━━━ 2s 17ms/step - loss: 5.9472e-04 - val_loss: 6.9195e-04
Epoch 30/50
147/147 ━━━━━━━━━━━━━━━━━━━━ 3s 18ms/step - loss: 6.4730e-04 - val_loss: 6.3914e-04
Epoch 31/50
147/147 ━━━━━━━━━━━━━━━━━━━━ 3s 23ms/step - loss: 6.0549e-04 - val_loss: 6.1053e-04
Epoch 32/50
147/147 ━━━━━━━━━━━━━━━━━━━━ 4s 17ms/step - loss: 5.7057e-04 - val_loss: 6.5777e-04
Epoch 33/50
147/147 ━━━━━━━━━━━━━━━━━━━━ 3s 18ms/step - loss: 5.6520e-04 - val_loss: 6.3420e-04
Epoch 34/50
147/147 ━━━━━━━━━━━━━━━━━━━━ 6s 23ms/step - loss: 5.5509e-04 - val_loss: 6.2457e-04
Epoch 35/50
147/147 ━━━━━━━━━━━━━━━━━━━━ 4s 18ms/step - loss: 5.4366e-04 - val_loss: 6.1086e-04
Epoch 36/50
147/147 ━━━━━━━━━━━━━━━━━━━━ 3s 19ms/step - loss: 5.7212e-04 - val_loss: 6.0025e-04
Epoch 37/50
147/147 ━━━━━━━━━━━━━━━━━━━━ 5s 19ms/step - loss: 5.3510e-04 - val_loss: 6.5680e-04
Epoch 38/50
147/147 ━━━━━━━━━━━━━━━━━━━━ 5s 18ms/step - loss: 5.3137e-04 - val_loss: 6.1901e-04
Epoch 39/50
147/147 ━━━━━━━━━━━━━━━━━━━━ 6s 23ms/step - loss: 5.3683e-04 - val_loss: 6.3036e-04
Epoch 40/50
147/147 ━━━━━━━━━━━━━━━━━━━━ 3s 19ms/step - loss: 5.4717e-04 - val_loss: 5.9631e-04
Epoch 41/50
147/147 ━━━━━━━━━━━━━━━━━━━━ 5s 19ms/step - loss: 5.1804e-04 - val_loss: 5.6965e-04
Epoch 42/50
147/147 ━━━━━━━━━━━━━━━━━━━━ 3s 23ms/step - loss: 4.8499e-04 - val_loss: 6.4022e-04
Epoch 43/50
147/147 ━━━━━━━━━━━━━━━━━━━━ 4s 18ms/step - loss: 4.9204e-04 - val_loss: 5.6821e-04
Epoch 44/50
147/147 ━━━━━━━━━━━━━━━━━━━━ 5s 20ms/step - loss: 4.8151e-04 - val_loss: 5.8584e-04
Epoch 45/50
147/147 ━━━━━━━━━━━━━━━━━━━━ 5s 19ms/step - loss: 4.4125e-04 - val_loss: 6.0236e-04
Epoch 46/50
147/147 ━━━━━━━━━━━━━━━━━━━━ 3s 18ms/step - loss: 4.9221e-04 - val_loss: 6.7488e-04
Epoch 47/50
147/147 ━━━━━━━━━━━━━━━━━━━━ 6s 22ms/step - loss: 4.6600e-04 - val_loss: 5.2596e-04
Epoch 48/50
147/147 ━━━━━━━━━━━━━━━━━━━━ 3s 18ms/step - loss: 4.8753e-04 - val_loss: 5.7113e-04
Epoch 49/50
147/147 ━━━━━━━━━━━━━━━━━━━━ 6s 21ms/step - loss: 4.5092e-04 - val_loss: 5.8502e-04
Epoch 50/50
147/147 ━━━━━━━━━━━━━━━━━━━━ 4s 17ms/step - loss: 4.0301e-04 - val_loss: 7.1454e-04
74/74 ━━━━━━━━━━━━━━━━━━━━ 1s 11ms/step

📋 Evaluation Metrics:
MSE: 0.0007, MAE: 0.0182, RMSE: 0.0267, R²: 0.9577
🔷 Computing SHAP for Okhla...
100%|==================| 11703/11705 [19:25<00:00]
```

SHAP Feature Importance - Okhla

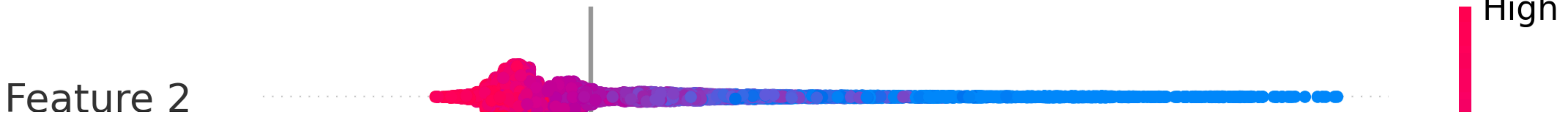

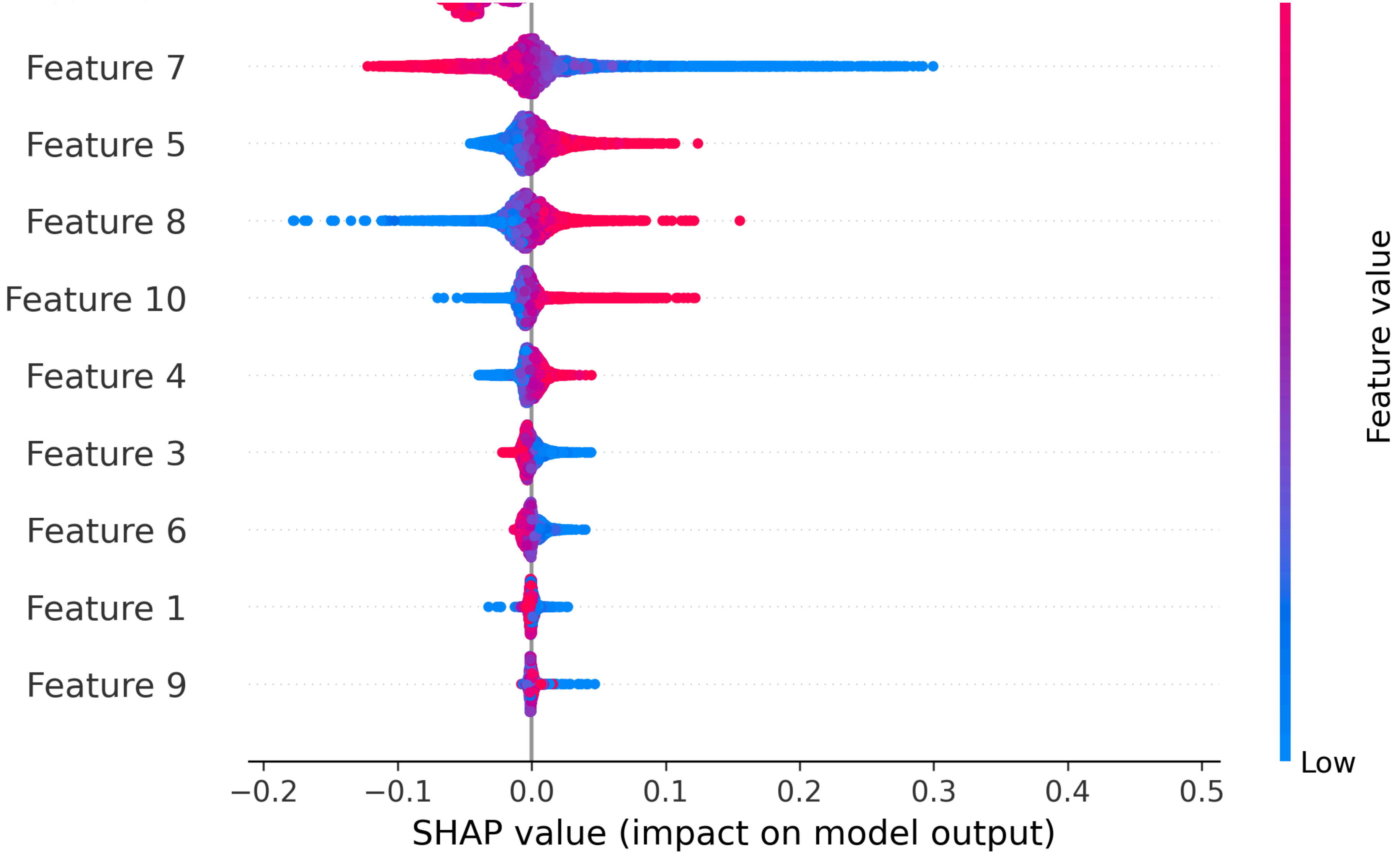
Feature 7
Feature 5
Feature 8
Feature 10
Feature 4
Feature 3
Feature 6
Feature 1
Feature 9
−0.2
−0.1
0.0
0.1
0.2
0.3
0.4
0.5
SHAP value (impact on model output)
Feature value
Low

```
import pandas as pd

df = pd.read_csv('/content/Najafgarh_Hourly.csv')

print(df.head())               # see the first rows
print(df.columns.tolist())    # check column names
print(df['PM2.5'].describe()) # check the stats
print(df['PM2.5'].unique())   # check unique values
```

```
   Unnamed: 0  PM2.5    year  month  day  hour   PM10     AT      BP   SR  \
0           0   58.0  2018.0    6.0  1.0   0.0  277.0  33.75  978.08  4.0
1           1   64.0  2018.0    6.0  1.0   1.0  260.0  33.56  978.00  4.0
2           2   52.0  2018.0    6.0  1.0   2.0  204.0  32.83  978.00  4.0
3           3   55.0  2018.0    6.0  1.0   3.0  201.0  32.17  978.00  4.0
4           4   61.0  2018.0    6.0  1.0   4.0  200.0  32.11  978.33  4.0

   ...    WS      WD    NO    NO2    SO2  Ozone    CO  Benzene    NH3    NOx
0  ...  2.62  126.75  0.32  31.91  10.62  53.20  1.17     0.10  54.61  32.21
1  ...  2.37  118.17  4.80  19.65   5.68  65.37  1.00     0.18  57.48  23.22
2  ...  3.18  126.00  0.28  18.73   2.17  69.28  0.40     0.55  19.45   6.34
3  ...  3.35  131.42  0.40  19.48   5.97  61.73  1.20     0.10  56.64  19.86
4  ...  5.53  141.08  0.32  16.17   5.02  64.41  1.22     0.10  46.60  16.51

[5 rows x 21 columns]
['Unnamed: 0', 'PM2.5', 'year', 'month', 'day', 'hour', 'PM10', 'AT', 'BP', 'SR', 'RH', 'WS', 'WD', 'NO', 'NO2', 'SO2', 'Ozone', 'CO', 'Benzene', 'NH3', 'NOx']
count    11704.000000
mean        76.825051
std         71.132566
min          0.030000
25%         27.250000
50%         51.000000
75%        106.511364
max        550.750000
Name: PM2.5, dtype: float64
[58.       64.       52.       ...  5.428571  5.285714  5.142857]
```

Impact of Feature Extraction on Model Performance

```
import matplotlib.pyplot as plt
import numpy as np
import pandas as pd

# Station names
stations = ['AshokVihar', 'DCStadium', 'DwarkaSec8', 'NehruNagar', 'Najafgarh', 'Okhla']

# R² and MSE values
r2_with = [0.9049, 0.9758, 0.9218, 0.9679, 0.8128, 0.9552]
r2_without = [0.3450, 0.9672, 0.9087, 0.6257, 0.5127, 0.4463]
mse_with = [0.0006, 0.0009, 0.0008, 0.0006, 0.0004, 0.0007]
mse_without = [0.0071, 0.0017, 0.0011, 0.0040, 0.0032, 0.0093]

x = np.arange(len(stations))
bar_width = 0.35

# Create subplot
```

```
    fig, axes = plt.subplots(1, 2, figsize=(16, 6))

    # --- R² subplot ---
    bars1 = axes[0].bar(x - bar_width/2, r2_with, bar_width, label='With FE', color='skyblue')
    bars2 = axes[0].bar(x + bar_width/2, r2_without, bar_width, label='Without FE', color='salmon')
    axes[0].set_xlabel('Station')
    axes[0].set_ylabel('R² Score')
    axes[0].set_title('R² Score Comparison')
    axes[0].set_xticks(x)
    axes[0].set_xticklabels(stations)
    axes[0].set_ylim(0, 1.1)
    axes[0].legend()
    axes[0].grid(axis='y', linestyle='--', alpha=0.7)

    for bar in bars1:
        height = bar.get_height()
        axes[0].text(bar.get_x() + bar.get_width()/2, height + 0.03, f'{height:.2f}', ha='center', fontsize=9)
    for bar in bars2:
        height = bar.get_height()
        axes[0].text(bar.get_x() + bar.get_width()/2, height + 0.03, f'{height:.2f}', ha='center', fontsize=9)

    # --- MSE subplot ---
    bars3 = axes[1].bar(x - bar_width/2, mse_with, bar_width, label='With FE', color='lightgreen')
    bars4 = axes[1].bar(x + bar_width/2, mse_without, bar_width, label='Without FE', color='orange')
    axes[1].set_xlabel('Station')
    axes[1].set_ylabel('Mean Squared Error (MSE)')
    axes[1].set_title('MSE Comparison')
    axes[1].set_xticks(x)
    axes[1].set_xticklabels(stations)
    axes[1].set_ylim(0, max(max(mse_with), max(mse_without)) + 0.005)
    axes[1].legend()
    axes[1].grid(axis='y', linestyle='--', alpha=0.7)

    for bar in bars3:
        height = bar.get_height()
        axes[1].text(bar.get_x() + bar.get_width()/2, height + 0.0005, f'{height:.4f}', ha='center', fontsize=9)
    for bar in bars4:
        height = bar.get_height()
        axes[1].text(bar.get_x() + bar.get_width()/2, height + 0.0005, f'{height:.4f}', ha='center', fontsize=9)

    # Title and layout
    plt.suptitle('Performance Comparison Across Stations (With vs Without Feature Extraction)', fontsize=16,fontweight='bold')
    plt.tight_layout(rect=[0, 0, 1, 0.95])
    plt.show()

    # --- Data Table Output ---
    comparison_df = pd.DataFrame({
        'Station': stations,
        'R² With FE': r2_with,
        'R² Without FE': r2_without,
        'MSE With FE': mse_with,
        'MSE Without FE': mse_without
    })

    print("\nComparison of R² and MSE (With vs Without Feature Extraction):")
```

```
print(comparison_df.to_string(index=False))
```

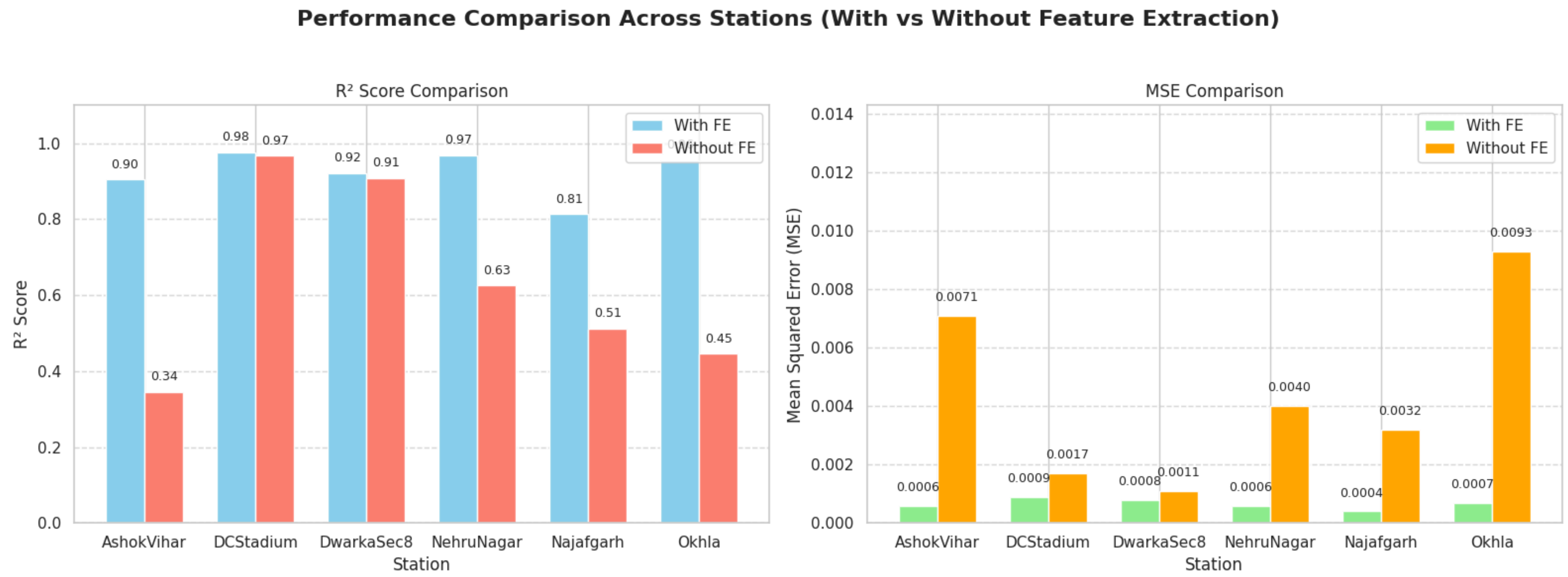


```
Comparison of R² and MSE (With vs Without Feature Extraction):
   Station  R² With FE  R² Without FE  MSE With FE  MSE Without FE
AshokVihar      0.9049         0.3450       0.0006          0.0071
 DCStadium      0.9758         0.9672       0.0009          0.0017
DwarkaSec8      0.9218         0.9087       0.0008          0.0011
NehruNagar      0.9679         0.6257       0.0006          0.0040
 Najafgarh      0.8128         0.5127       0.0004          0.0032
     Okhla      0.9552         0.4463       0.0007          0.0093
```

Feature Selection Comparison for Air Quality Prediction: AOAOA vs. Other Optimization Techniques

```
# Replot with all text (titles, labels, tick labels, legend) in bold font

sns.set_style("whitegrid")
fig, axes = plt.subplots(1, 2, figsize=(18, 6))
fig.subplots_adjust(top=0.82)
fig.suptitle("Multi-Station Performance Comparison", fontsize=16, fontweight='bold')

colors = sns.color_palette("tab10") + sns.color_palette("Set2")
bar_width = 0.06
x_indexes = np.arange(len(stations))

# RMSE Plot
```

```
    for i, method in enumerate(methods):
        axes[0].bar(x_indexes + i * bar_width, rmse_values[method], width=bar_width,
                    color=colors[i], edgecolor='black', label=method)

    axes[0].set_title('RMSE Comparison (Lower is Better)', fontsize=12, fontweight='bold')
    axes[0].set_xlabel('Stations', fontsize=11, fontweight='bold')
    axes[0].set_ylabel('RMSE', fontsize=11, fontweight='bold')
    axes[0].set_xticks(x_indexes + bar_width * (len(methods) / 2))
    axes[0].set_xticklabels(stations, rotation=30, ha='right', fontsize=10, fontweight='bold')
    axes[0].tick_params(axis='y', labelsize=10)
    axes[0].grid(axis='y', linestyle='--', alpha=0.7)

    # R² Score Plot
    for i, method in enumerate(methods):
        axes[1].bar(x_indexes + i * bar_width, r2_values[method], width=bar_width,
                    color=colors[i], edgecolor='black', label=method)

    axes[1].set_title('R² Score Comparison (Higher is Better)', fontsize=12, fontweight='bold')
    axes[1].set_xlabel('Stations', fontsize=11, fontweight='bold')
    axes[1].set_ylabel('R² Score', fontsize=11, fontweight='bold')
    axes[1].set_xticks(x_indexes + bar_width * (len(methods) / 2))
    axes[1].set_xticklabels(stations, rotation=30, ha='right', fontsize=10, fontweight='bold')
    axes[1].tick_params(axis='y', labelsize=10)
    axes[1].grid(axis='y', linestyle='--', alpha=0.7)

    # Bold Legend
    handles = [plt.Rectangle((0, 0), 1, 1, color=colors[i]) for i in range(len(methods))]
    legend = fig.legend(handles, methods, loc='upper center', bbox_to_anchor=(0.5, 1.10),
                        ncol=6, fontsize=10, frameon=False, title="Optimization Methods")
    plt.setp(legend.get_texts(), fontweight='bold')
    plt.setp(legend.get_title(), fontweight='bold')

    plt.tight_layout(rect=[0, 0, 1, 0.94])
    plt.show()
```
